# Causal effects of potential risk factors on postpartum depression: a Mendelian randomization study

**DOI:** 10.3389/fpsyt.2023.1275834

**Published:** 2023-12-20

**Authors:** Mingrong Zuo, Zhihao Wang, Wenhao Li, Siliang Chen, Yunbo Yuan, Yuan Yang, Qing Mao, Yanhui Liu

**Affiliations:** Department of Neurosurgery, West China Hospital, Sichuan University, Chengdu, China

**Keywords:** postpartum depression, causal factors, mental disorder, Mendelian randomization, linkage disequilibrium score regression

## Abstract

**Background:**

Postpartum depression (PPD) is a type of depressive episode related to parents after childbirth, which causes a variety of symptoms not only for parents but also affects the development of children. The causal relationship between potential risk factors and PPD remains comprehensively elucidated.

**Methods:**

Linkage disequilibrium score regression (LDSC) analysis was conducted to screen the heritability of each instrumental variant (IV) and to calculate the genetic correlations between effective causal factors and PPD. To search for the causal effect of multiple potential risk factors on the incidence of PPD, random effects of the inverse variance weighted (IVW) method were applied. Sensitivity analyses, including weighted median, MR-Egger regression, Cochrane’s Q test, and MR Pleiotropy Residual Sum and Outlier (MR-PRESSO), were performed to detect potential Mendelian randomization (MR) assumption violations. Multivariable MR (MVMR) was conducted to control potential multicollinearity.

**Results:**

A total of 40 potential risk factors were investigated in this study. LDSC regression analysis reported a significant genetic correlation of potential traits with PPD. MR analysis showed that higher body mass index (BMI) (Benjamini and Hochberg (BH) corrected *p* = 0.05), major depression (MD) (BH corrected *p* = 5.04E-19), and schizophrenia (SCZ) (BH corrected *p* = 1.64E-05) were associated with the increased risk of PPD, whereas increased age at first birth (BH corrected *p* = 2.11E-04), older age at first sexual intercourse (BH corrected *p* = 3.02E-15), increased average total household income before tax (BH corrected *p* = 4.57E-02), and increased years of schooling (BH corrected *p* = 1.47E-11) led to a decreased probability of PPD. MVMR analysis suggested that MD (*p* = 3.25E-08) and older age at first birth (*p* = 8.18E-04) were still associated with an increased risk of PPD.

**Conclusion:**

In our MR study, we found multiple risk factors, including MD and younger age at first birth, to be deleterious causal risk factors for PPD.

## 1 Introduction

Postpartum depression (PPD), also termed postnatal depression, is a type of depressive episode related to childbirth with a variety of symptoms of mood changes, negative attitudes to life, and mental and psychiatric changes, with few severe cases showing suicidality (1). The diagnostic criteria of PPD comprise five or more symptoms, such as depressed mood, loss of previous interest, weight or appetite alteration, somnipathy, fatigue, attention deficit, and feeling worthless or guilty (2). PPD occurs in approximately 13% of women, which may cause severe consequences for mothers and their children (3). Moreover, the reported overall incidence of paternal PPD between 3 months and 12 months after birth was about 10.4% (4), which suggested that both new parents may suffer from this common psychiatric disorder. Apart from causing mental disorders or even suicides of parents, the risks of PPD on children have been well-investigated, and it suggested that children with parents experiencing PPD are more likely to suffer from depressive disorders (5). Regarding the risk factors associated with PPD, a variety of research found that multiple factors, including a history of mental illness, hormonal changes, and social factors, were related to PPD (1, 6). The causal relationship between potential risk factors and PPD is still unelucidated, and a comprehensive exploration of the causal effects of factors on PPD would be helpful for the intervention of PPD.

Mendelian randomization (MR) is a useful way to infer a causal relationship, using genetic variants as instrumental variants (IVs) to estimate the causal assumption between exposure and outcome (7). Due to the random allocation of genetic variants, MR is less susceptible to other confounding factors and reverse causation that can impede causal inference in conventional observational studies (8). Multivariable MR (MVMR) takes pleiotropy among multiple exposures into account, which is an extension of univariable MR and is important for pleiotropic pathways (9). Thus, accumulating research has taken advantage of MR to gain insights into the causes of multiple diseases (10). Recently, MR analysis initially demonstrated a potential causal relationship between opioid use and the risk of PPD. In turn, PPD was also associated with a higher risk of opioid and non-opioid analgesic use (11). Another recent study investigated the causal association between PPD and cerebrovascular diseases and cognitive impairment, and the result recommended that cognitive impairment was a significant outcome induced by PPD (12). In addition, modifiable risk factors, such as body mass index (BMI) and glucose, are usually considered exposures that may be associated with diseases in MR analysis (13). However, the causal association of other potential risk factors, particularly modifiable risk factors with PPD, still remains unclear.

In the present study, we focused on the association of various potential risk factors and modifiable factors with PPD. Based on the summarized data of the genome-wide association study (GWAS), we investigated the genetic correlations between 40 potential risk factors and PPD by linkage disequilibrium score regression (LDSC) analysis and then performed a two-sample MR analysis.

## 2 Materials and methods

### 2.1 Study design

**Figure 1:**
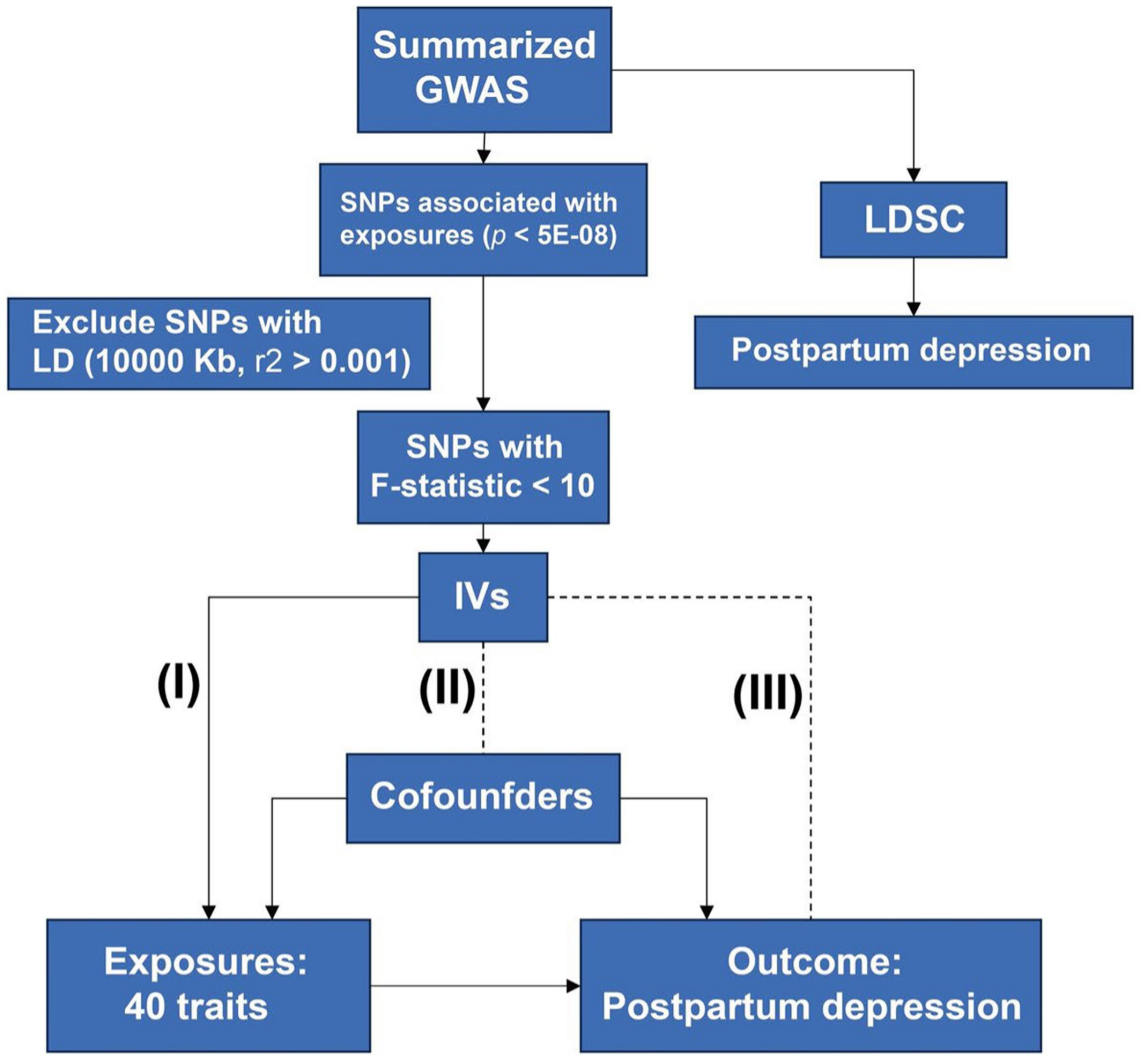
Design of the study. Procedures of MR are shown in this flow chart. Three principles of MR were listed: the IVs must be associated with exposure (I) and not be associated with confounders (II) and outcome (III). MR, mendelian randomization; GWAS, genome-wide association studies; SNP, single nucleotide polymorphism; IV, instrumental variant; LDSC, linkage disequilibrium score regression.

A procedure of MR analysis was established to investigate a causal relationship between potential risk factors and PPD. Figure 1 shows the procedures of our study. The principles of selecting potential risk factors were as follows: First, we sought out a review of the PPD (14), which mentioned the risk factors of PPD, including history of mental illness (psychiatric disorders), hormonal changes (sex hormones), and social factors (socioeconomic Factors). Another study reported the association between thyroid function and PPD (15). Second, we investigated the modifiable risk factors frequently used as exposures in MR analysis, including BMI, blood pressure, glucose and lipids, and diet hobbies such as smoking, drinking alcohol, tea, and coffee. Third, we added some risk factors that may be involved in the pathogenesis of depression, including disorder of sleep (16) and inflammatory processes (inflammatory biomarkers) (17). In total, there were 40 potential risk factors.

Three major assumptions were utilized. First, there existed a direct correlation between the IVs of potential exposures and the PPD (Relevance). Second, the confounders cannot confound the IVs of exposures (Exchangeability). Finally, the IVs have no direct connection to the outcome except through exposure (the exclusion restriction).

### 2.2 Sources of exposures and PPD in GWAS data

We obtained data on exposures from the GWAS data. The exposure factors of this study are as follows: BMI, total testosterone, bioavailable testosterone, sex hormone binding globulin, estradiol, coffee intake, tea intake, average total household income before tax, age at menarche^1^ (18–20), Schizophrenia (SCZ) (21), autism spectrum disorder (22), bipolar disorder (23), major depression (MD) (24), attention-deficit/hyperactivity disorder (25), anxiety (26), diastolic blood pressure, pulse pressure, systolic blood pressure (27), glycated hemoglobin, fasting glucose, fasting insulin, 2-h glucose (28), triglycerides, low-density lipoprotein cholesterol-c, high-density lipoprotein cholesterol-c, apolipoprotein A-I, apolipoprotein B (29), free thyroxine4, thyrotropin (TSH), incTSH/hypothyroidism, decTSH/hyperthyroidism (30), 25 hydroxyvitamin D (31), C-reactive protein (32), smoking initiation (SI), cigarettes per day, alcohol consumption (33), years of schooling (33), age at first sexual intercourse, age at first birth (34), and insomnia (35). Next, the GWAS data of PPD were obtained from the European cohort: the FinnGen study round 8 (36). The FinnGen study is dedicated to combining genome information with digital healthcare data (37). The criterion in FinnGen was based on the 10th edition of the International Classification of Diseases criteria, in which participants with delivery history (O15) diagnosed with F32, F33, or F530 were identified as PPD. A total of 13,657 PPD cases and 236,178 controls were enrolled in the dataset. GWAS summary data of PPD and all risk factors are presented in Table 1. It is worth noting that the ethnicity of the population of attention deficit/hyperactivity disorder was European, North American, and Chinese, and the ethnicity of other GWAS data was European. Given that the diagnostic criteria of the PPD included a history of delivery, we speculated that the gender of this study was restricted to biological females. We elected GWAS of sex hormones, free thyroxine, and TSH of female-only participants as the rest of the studies did not provide gender-specified data.

**Table 1:** Description of GWAS statistics included in the present study.

Outcome and potential risk factors	Traits	Sample size	PMID or data source	Population	Sex
	PPD	249,835 (13,657 cases and 236,178 controls)	https://risteys.finregistry.fi/endpoints/O15_POSTPART_DEPR	European	-
Psychiatric disorders	SCZ	127,906 (52,017 cases and 75,889 controls)	35396580	European	M/F
	Autism Spectrum Disorder	46,350 (18,381 cases and 27,969 controls)	30804558	European	M/F
	Bipolar disorder	34,950 (7,647 cases and 27,303 controls)	27329760	European	M/F
	MD	500,199 (170,756 cases and 329,443 controls)	30718901	European	M/F
	Attention deficit/hyperactivity disorder	55,374 [20,183 cases (1,084 non-European cases) and 35,191 controls (997 non-European cases)]	30478444	European, North American, and Chinese	M/F
	Anxiety	17,310	26754954	European	M/F
Overweight	BMI	454,884	MRC-IEU (https://gwas.mrcieu.ac.uk/, GWAS ID: ukb-b-2303)	European	M/F
Blood pressure	Diastolic blood pressure	810,865	33230300	European	M/F
	Pulse pressure	810,865		European	M/F
	Systolic blood pressure	810,865		European	M/F
Glucose	Glycated hemoglobin	146,806	34059833	European	M/F
	Fasting glucose	200,622		European	M/F
	Fasting insulin	151,013		European	M/F
	2-h glucose	63,396		European	M/F
Lipids	Triglycerides	441,016	32203549	European	M/F
	LDL-c	440,546		European	M/F
	HDL-c	403,943		European	M/F
	Apolipoprotein A-I	393,193		European	M/F
	Apolipoprotein B	439,214		European	M/F
Sex hormones	Total Testosterone	199,569	MRC-IEU (https://gwas.mrcieu.ac.uk/, GWAS ID: ieu-b-4864)	European	F
	Bioavailable Testosterone	180,386	MRC-IEU (https://gwas.mrcieu.ac.uk/, GWAS ID: ieu-b-4869)	European	F
	Sex hormone-binding globulin	214,989	MRC-IEU (https://gwas.mrcieu.ac.uk/, GWAS ID: ieu-b-4870)	European	F
	Estradiol	53,391	MRC-IEU (https://gwas.mrcieu.ac.uk/, GWAS ID: ieu-b-4872)	European	F
Thyroid function	Free thyroxine	26,954	30367059	European	F
	TSH	29,670		European	F
	incTSH/hypothyroidism	53,323 (3,340 cases and 49,983 controls)		European	M/F
	decTSH/hyperthyroidism	51,823 (1,840 cases and 49,983 controls)		European	M/F
Inflammatory biomarkers	Serum 25-Hydroxyvitamin D levels adjusted BMI	417,580	32242144	European	M/F
	C-reactive protein	575,531	35459240	European	M/F
Habits	SI	632,802 (311,629 cases and 321,173 controls)	30643251	European	M/F
	Cigarettes per day	337,334		European	M/F
	Alcohol consumption	941,280		European	M/F
	Coffee intake	428,860	MRC-IEU (https://gwas.mrcieu.ac.uk/, GWAS ID: ukb-b-5237)	European	M/F
	Tea intake	447,485	MRC-IEU (https://gwas.mrcieu.ac.uk/, GWAS ID: ukb-b-6066)	European	M/F
Socioeconomic Factors	Years of schooling	766,345	30038396	European	M/F
	Average total household income before tax	397,751	MRC-IEU (https://gwas.mrcieu.ac.uk/, GWAS ID: ukb-b-7408)	European	M/F
	Age at first sexual intercourse	397,338	34211149	European	M/F
	Age at first birth	542,901		European	M/F
	Age at menarche	243,944	MRC-IEU (https://gwas.mrcieu.ac.uk/, GWAS ID: ukb-b-3768)	European	M/F
Sleep	Insomnia	453,379	30804566	European	M/F

F, female; M, male; MRC-IEU, the Medical Research Council Integrative Epidemiology Unit; HDL-c, high-density lipoprotein cholesterol-c; LDL-c, low-density lipoprotein cholesterol-c; MD, major depression; BMI, body mass index; PPD, postpartum depression; SCZ, schizophrenia; SI, smoking initiation; TSH, thyrotropin.

### 2.3 Linkage disequilibrium score regression

To screen the heritability of each trait and the genetic correlations between effective causal factors and PPD, LDSC regression was conducted by the regression slope using GWAS summary data (38). The European Ancestry 1,000 Genomes LD reference panel was used as a reference.

### 2.4 Genetic instruments selection

To select the qualified IVs, only single nucleotide polymorphisms (SNPs) associated with each exposure at a genome-wide threshold (*p* < 5E-08) were elected. A less strict threshold of 5E-06 was applied if less than four IVs were involved in the inverse variance weighted (IVW) analysis. SNPs with linkage disequilibrium were filtered based on the European ancestry 1,000 Genomes LD reference panel, with r2 > 0.001 on the clump window of 10,000 kb to keep the IVs independent from each other. The F-statistic of each SNP was calculated, and any SNP with a low F-statistic (< 10) was removed to avoid weak instrument bias. SNPs significantly associated with outcome (*p* < 5E-08) were excluded as they violated the third principle of MR assumption. SNPs that passed these precedes and existed in the GWAS of outcome were harmonized to avoid a mismatch of alleles based on allele frequencies for palindromes (19).

### 2.5 Mendelian randomization analysis

We used IVW to analyze the causal relationship of 40 potential risk factors in the PPD (7). In addition, weighted median, MR-Egger regression, heterogeneity test, Cochrane’s Q test, and MR-PRESSO were utilized to assess the robustness of IVW results. We appraised the heterogeneity using Cochran’s Q statistics and I^2^ statistics (39), and if heterogeneity exists (*p* < 0.05), the results should coincide with results estimated by IVW (39). In the pleiotropy test, intercepts calculated by MR-PRESSO were used to assess the horizontal pleiotropy of valid IVs; *p* > 0.05 meant a lower probability of horizontal pleiotropy existence (40). Outliners were detected and removed by MR-PRESSO. The validation of causal inference between exposures and PPD depended on the same tendency of IVW and weighted median and MR-Egger analyses, besides no horizontal pleiotropic effect existing. To control potential multicollinearity, multivariable MVMR was conducted with significant results of the IVW method as exposures. For MVMR, the IVs must be associated with at least one of the exposures, independent of all confounders of any exposure and independent of the outcome (41). Least absolute shrinkage and selection operator regression were utilized to avoid potential bias caused by multicollinearity.

### 2.6 Statistical analysis

All the statistical analyses were performed using R-4.2.3^2^ with R packages. The R packages included the two-sample MR package (19), MR-PRESSO package (40), and LDSC package^3^ (38, 42, 43). To control type I error, BH (Benjamini and Hochberg) correction was employed in both LDSC and IVW analyses. The BH-adjusted *p* < 0.05 was considered strong evidence, while the standard *p* < 0.05 was identified as suggestive evidence. Scatter plots and forest plots were used for the visualization of MR results.

### 2.7 Ethical approval

The data from public sources of our study had been granted ethical approval by their own institutional review boards and thus it did not require ethical approval from our institutional review board.

## 3 Results

### 3.1 Linkage disequilibrium score regression

We performed LDSC to analyze genetic correlations between potential causal factors and PPD. The heritability of SNPs for each exposure factor with a range of 2 to 36% is listed in Supplementary Table S1. Among 40 factors, half of them were associated with PPD even following BH correction, as listed in Table 2.

**Table 2:** Genetic correlation between PPD and potential risk factors.

P1	P2	rg	se	*p*-value	BH-corrected *p*-value
PPD	SCZ	0.285	0.038	1.11E-13	4.93E-13
	Autism Spectrum Disorder	0.516	0.064	9.02E-16	5.15E-15
	Bipolar disorder	0.267	0.058	4.58E-06	1.31E-05
	MD	0.623	0.053	3.98E-32	7.96E-31
	Attention deficit/hyperactivity disorder	0.516	0.064	9.02E-16	5.15E-15
	Anxiety	0.075	0.062	0.227	0.350
	BMI	0.162	0.034	1.41E-06	4.34E-06
	Diastolic blood pressure	−0.029	0.077	0.701	0.802
	Pulse pressure	0.037	0.070	0.596	0.711
	Systolic blood pressure	0.013	0.065	0.837	0.881
	Glycated hemoglobin	0.015	0.055	0.793	0.858
	Fasting glucose	0.039	0.057	0.499	0.689
	Fasting insulin	0.092	0.061	0.134	0.223
	2-h glucose	0.033	0.093	0.721	0.802
	Triglycerides	0.120	0.033	2.67E-04	6.67E-04
	LDL-c	−0.024	0.039	0.551	0.710
	HDL-c	−0.088	0.035	0.012	0.027
	Apolipoprotein A-I	−0.064	0.036	0.072	0.125
	Apolipoprotein B	0.020	0.038	0.604	0.711
	Total Testosterone	−0.028	0.044	0.523	0.698
	Bioavailable Testosterone	−0.004	0.045	0.931	0.955
	Sex hormone-binding globulin	−0.051	0.045	0.251	0.372
	Estradiol	−0.268	0.259	0.301	0.430
	Free thyroxine	−0.062	0.032	0.058	0.105
	Thyrotropin	0.560	0.234	0.017	0.034
	Hypothyroidism	0.550	0.230	0.017	0.034
	Hyperthyroidism^a^	NA	NA	NA	NA
	Serum 25-Hydroxyvitamin D levels adjusted BMI	0.083	0.040	0.037	0.071
	C-reactive protein levels	0.208	0.037	2.01E-08	6.70E-08
	SI	0.353	0.042	5.14E-17	4.11E-16
	Cigarettes per day	0.257	0.043	2.75E-09	1.00E-08
	Alcohol consumption	0.133	0.043	0.002	0.005
	Coffee intake	−0.184	0.050	2.18E-04	5.83E-04
	Tea intake	0.069	0.048	0.151	0.241
	Years of schooling	−0.348	0.038	2.34E-20	2.34E-19
	Average total household income before tax	−0.359	0.055	5.04E-11	2.02E-10
	Age at first sexual intercourse	−0.480	0.042	2.18E-30	2.91E-29
	Age at first birth	−0.646	0.053	3.23E-34	1.29E-32
	Age at menarche	0.018	0.034	0.589	0.711
	Insomnia	0.370	0.047	2.86E-15	1.43E-14

a Error occurred in performing linkage disequilibrium score regression, where a negative heritability existed in the genetic covariance matrix.

BH, Benjamini and Hochberg; PPD, postpartum depression; MD, major depression; BMI, body mass index; SCZ, schizophrenia; SI, smoking initiation; HDL-c, high-density lipoprotein cholesterol-c; LDL-c, low-density lipoprotein cholesterol-c.

### 3.2 IVs selection

SNPs were selected for each pair of exposure and outcome, and detailed information on all SNPs was listed in the Supplementary Table S2. We defined the qualified IVs as only SNPs associated with each exposure at a genome-wide threshold (*p* < 5E-08). Due to the lack of IVs at the genome-wide significance with anxiety, autism spectrum disorder, and estradiol as exposure, the threshold of 5E-06 was applied in the three analyses. The total F-statistic of all pairs was above 10, indicating strong IVs.

### 3.3 IVW analysis of potential risk factors for PPD

**Figure 2:**
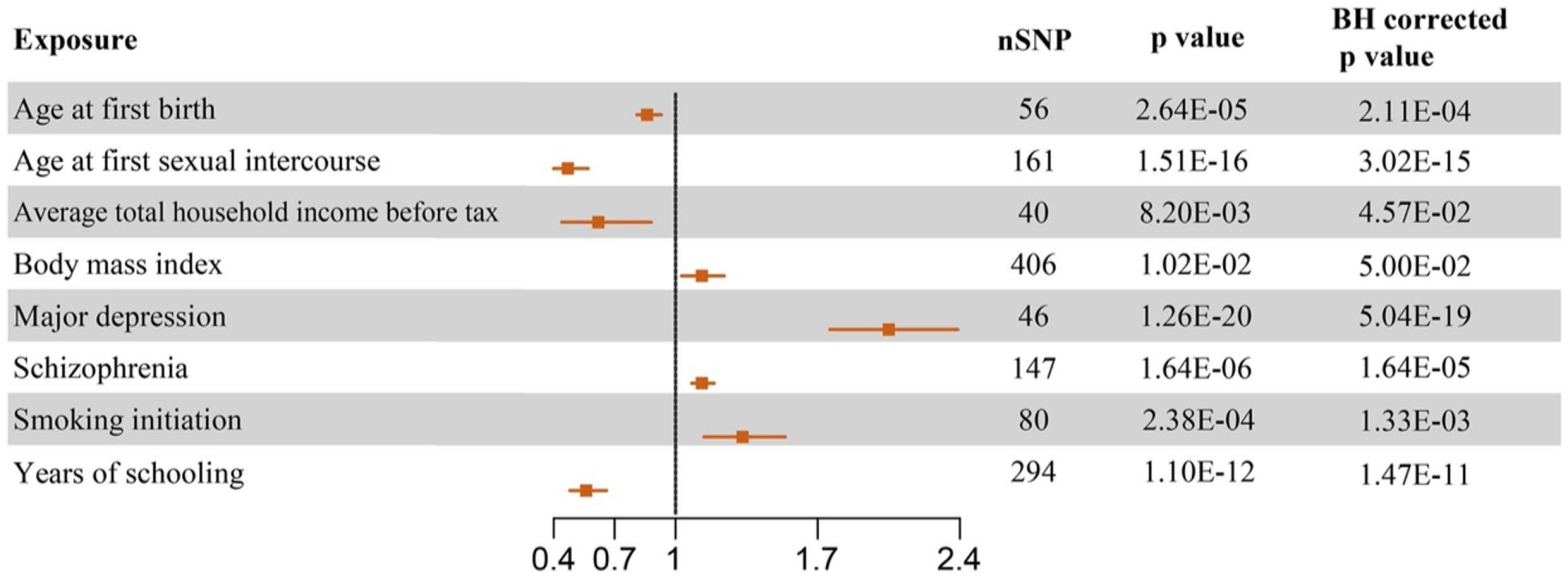
Forest plots of causal effect estimates based on Mendelian analysis. The significant results of causal inference of eight potential risk factors on PPD in random effect IVW analysis were plotted. SNP, single nucleotide polymorphism; OR, odds ratio; CI, confidence interval.

Our results of MR analyses indicated that among 40 potential risk factors, eight of them had a significant causal relationship with the PPD. The functionalities of SNPs, such as higher BMI (odds ratio (OR) = 1.13 (1.03, 1.24), BH corrected *p* = 0.05), MD (OR = 2.05 (1.76, 2.39), BH corrected *p* = 5.04E-19), SCZ (OR = 1.13 (1.08, 1.19), BH corrected *p* = 1.64E-05), and earlier SI (OR = 1.33 (1.14, 1.54), BH corrected *p* = 1.33E-03), were associated with the increased risk of PPD. On the contrary, we also found that increased age at first birth (OR = 0.86 (0.81, 0.93), BH corrected *p* = 2.11E-04), older age at first sexual intercourse (OR = 0.47 (0.4, 0.57), BH corrected *p* = 3.02E-15), increased average total household income before tax (OR = 0.62 (0.44, 0.88), BH corrected *p* = 4.57E-02), and increased years of schooling (OR = 0.56 (0.48, 0.66), BH corrected *p* = 1.47E-11) led to a decreased probability of PPD. While no significant causal relationship was found between the other 32 exposures and PPD. Complete results are available in Figure 2 and Supplementary Table S3.

### 3.4 Sensitivity analysis of MR

**Figure 3:**
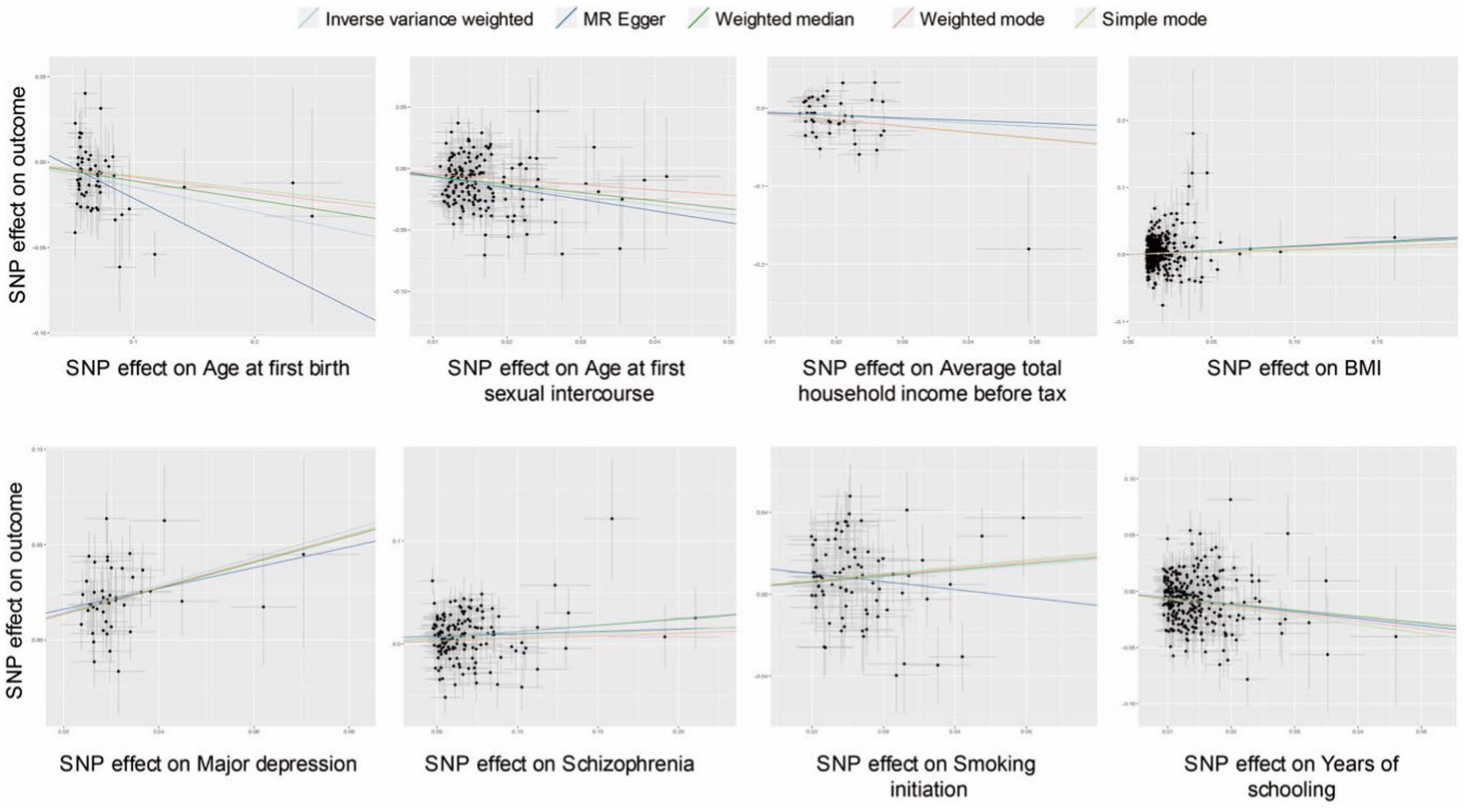
Scatter plots of causal effect inference for effective causal factors on PPD, respectively. SNP, single nucleotide polymorphism; BMI, body mass index.

The scatter plots of effective exposures with causal effects on PPD are presented in Figure 3. Apart from SI showing a discrepant correlation with PPD between MR Egger and IVW analysis and weighted median analysis (Figure 3), the rest of the effective causal effectors all presented a clear causal relationship with a similar tendency, which was in line with each corresponding forest plot (Figures 2, 3). Heterogeneity was detected in six causal factors identified by IVW, which are as follows: age at first sexual intercourse, average total household income before tax, BMI, SCZ, SI, and years of schooling. The pleiotropy test showed no horizontal pleiotropy in all analyses (*p* > 0.05). MR-PRESSO analysis suggested outliners were presented in the following exposures: average total household income before tax, BMI, SCZ, and years of schooling, deleting those that did not affect the results. The complete results of the sensitivity analysis are available in Supplementary Table S4.

### 3.5 Multivariable Mendelian randomization

We conducted an MVMR analysis with eight potential causal factors identified by IVW analysis. IVs were selected from shared SNPs of all exposure GWAS (Supplementary Table S5). None of these exposures were excluded after the least absolute shrinkage and selection operator regression. Taking all eight factors into account, genetically predicted MD was still associated with an increased risk of PPD (OR = 1.63 (1.37, 1.94), *p* = 3.25E-08). In contrast, older age at first birth was associated with a lower risk of PPD (OR = 0.81 (0.72, 0.92), *p* = 8.18E-04). The rest of the factors were not significant. Detailed information is listed in Table 3.

**Table 3:** MVMR analysis of eight significant causal factors.

Exposure	n(SNP)	OR (95% CI)	*p*-value
Age at first sexual intercourse	55	1.05 (0.73–1.52)	0.790
Age at first birth	11	0.81 (0.72–0.92)	8.18E-04
Years of schooling	123	0.89 (0.60–1.32)	0.568
MD	18	1.63 (1.37–1.94)	3.25E-08
SI	18	0.92 (0.75–1.14)	0.4598
SCZ	43	1.05 (0.99–1.12)	0.112
BMI	227	0.90 (0.79–1.01)	0.081
Average total household income before tax	11	0.97 (0.63–1.47)	0.869

MVMR, multivariable Mendelian randomization; SNP, single nucleotide polymorphism; MD, major depression; BMI, body mass index; SCZ, schizophrenia; SI, smoking initiation.

## 4 Discussion

In the present MR study, we found that higher BMI, MD, SCZ, and earlier SI increased the risk of PPD. On the contrary, the risk factors of increased age at first birth, older age at first sexual intercourse, increased income, and higher education level led to a decreased probability of PPD. In sensitivity analysis, contradictory results of SI were reported, which should be interpreted with caution. The other 32 risk factors did not show a statistically causal correlation with PPD. Adjustment for all eight significant traits in the MVMR model further found that MD and older age at first birth were still associated with an increased risk of PPD. Thus, our study shed fresh light on multiple causal factors associated with PPD.

Younger maternal age was reported to be associated with an increased risk of PPD in women (44). There was an MR analysis illustrating that the older age of first birth was a protective factor for mental disorders (45). Consistently, we found a negative causal relationship between age at first birth and PPD; thus, we suggested that relatively late-to-be parents may be less likely to develop PPD. Early sexual intercourse was associated with mental health outcomes (46). A novel MR study established a risky causal relationship between early sexual intercourse and MD (47). In our study, we initially brought forward that the experience of early sexual intercourse exerted a deleterious effect on PPD. As one of the social determinants, higher education level is identified as a protective factor for PPD (48). Our study also suggested that longer years of schooling were negatively associated with the risks of PPD, which was readily understandable as the higher the level of education, the higher the capability to withstand health and wealth risks. Whether income level or urbanization is associated with depression remains controversial (49). Here, we found that a lower average total household income before tax was associated with an increased risk of PPD. This conclusion needs further investigation using genetic biobanks of middle- and low-income countries. Based on these results, clinicians and parents should take advantage of these viable socioeconomic factors to prevent PPD, such as avoiding earlier first birth and immature sexual intercourse and acquiring a higher education level and a higher income.

Regarding the negative causalities of PPD, we found that modifiable risk factors, both higher BMI and earlier SI, significantly increased the likelihood of PPD. Recently, MR analysis has already found a promoting role of BMI in the risk of MD. In addition, it suggested that depression increases the genetic susceptibility to high BMI (50), which means a positive feedback between BMI and depression and needs the clinician’s close attention and intervention. Combined with our findings, further clinical trials should investigate the effect of preventing PPD by getting rid of excess weight. Non-smoking has been considered a protective causal factor in the incidence of MD (51). This was consistent with our MR results, which indicated a direct effect of earlier SI on an increased risk for PPD. As the risk factors above are relatively easy to modify, we postulate that reducing BMI and postponing SI may decrease the risk of PPD. Although insomnia was associated with an increased risk of perinatal depression (52), we did not conclude a causal relationship between insomnia and PPD. An observational study reported that 11% of women and 8% of men had PPD after the first birth, and it was partly attributed to postpartum sleep deprivation (53). In contrast, our finding suggested no causal effect of insomnia on PPD. Although insomnia is prevalently comorbid with depression, and treating insomnia is beneficial to the mood of patients (54, 55), further research is warranted to identify the role of insomnia in the pathogenesis of PPD.

Finally, we reported that a history of MD increased the risk of PPD, which was in line with previous findings (56, 57). In addition, there was evidence of a positive causal effect between SCZ and PPD in our study. An observational study suggested that PPD and postpartum psychosis were related to a higher risk of SCZ (58). Our MR analysis further strengthened the robustness of the positive causal connection between mental disorders, such as SCZ and MD, and the risk of PPD. More than half of women with PPD have a comorbidity of bipolar disorder, which usually being misdiagnosed or neglected (59). In our study, we did not conclude a causal relationship between these two diseases. We speculated that the negative result may be related to limited cases, distinct diagnostic criteria, and sequencing depth for bipolar disorder. Anyway, future studies should try to illustrate the association between bipolar disorder and PPD. Subsequently, we conducted an MVMR analysis to control potential multicollinearity. We included all eight significant traits from the IVW analysis, of which the MR Egger analysis showed that SI had an opposite direction compared to IVW and weighted median analysis, which should be interpreted with caution. In addition, BMI did not reach a significance following BH correcting. Importantly, compared to univariable MR, the effect of MD and older age at first birth on the increased risks of PPD were still observed. Thus, our study brought forward the most important causal factors of PPD, which may help future studies to better prevent and treat PPD.

Our study has limitations. First, the data sources are mainly from participants with European ancestry; thus, our result inevitably may not adapt to different ethnic groups, and the non-European participants in the GWAS of attention-deficit/hyperactivity disorder might lead to biases in results due to variations in allele frequencies and genetic associations among different populations and ancestry-specific effects. Second, the causal relationship of our findings does not conduct external verification as we only obtained one GWAS data of PPD. Therefore, we utilize more approaches to promote the robustness of our results, such as using the MR egger and weighted median analysis. In addition, one of the limitations of the methodology of MR is the potential confounding of the genetic variants and the outcome (Violation of IV condition two: exchangeability). Other than that, another bias of MR analysis that is uneasily corrected is the selection bias (10). In all, we should be prudent with our findings of MR because of the limitations above.

In summary, our MR analysis provided suggestive evidence of the protective effect of older age at first birth and older age at first sexual intercourse, higher education, and higher income on the risk of PPD. We also suggested that higher BMI and a history of MD and SCZ were hazardous causal factors for PPD. In addition, MD and older age at first birth were independently related to the increased risks of PPD. These novel shreds of evidence may help to guide the prevention and intervention strategies for PPD.

## Data availability statement

Publicly available datasets were analyzed in this study. See Table 1 for detailed information. Further inquiries can be directed to the corresponding author.

## Ethics statement

Ethical approval was not required for the study involving humans in accordance with the local legislation and institutional requirements. Written informed consent to participate in this study was not required from the participants or the participants’ legal guardians/next of kin in accordance with the national legislation and the institutional requirements.

## Author contributions

MZ: Conceptualization, Data curation, Investigation, Methodology, Resources, Supervision, Validation, Visualization, Writing – original draft, Writing – review & editing. ZW: Conceptualization, Data curation, Formal analysis, Investigation, Methodology, Resources, Software, Validation, Visualization, Writing – original draft, Writing – review & editing. WL: Data curation, Methodology, Validation, Visualization, Writing – review & editing. SC: Formal analysis, Investigation, Validation, Writing – review & editing. YunY: Data curation, Formal analysis, Investigation, Project administration, Validation, Writing – review & editing. YuaY: Funding acquisition, Supervision, Validation, Writing – review & editing. QM: Data curation, Supervision, Validation, Writing – review & editing. YL: Conceptualization, Funding acquisition, Investigation, Supervision, Validation, Writing – review & editing.

## Figures and Tables

**Table:** 

BMI	body mass index
BH	Benjamini and Hochberg
IV	instrumental variant
GWAS	genome-wide association study
IVW	inverse variance weighted
LDSC	linkage disequilibrium score regression
MD	Major depression
MR	mendelian randomization
MVMR	multivariable MR
OR	odds ratio
PPD	postpartum depression
SCZ	schizophrenia
SI	smoking initiation
SNP	single nucleotide polymorphism
TSH	Thyrotropin

## Footnotes

^1^ https://gwas.mrcieu.ac.uk/

^2^ https://www.R-project.org/

^3^ https://github.com/mglev1n/ldscr
