# Supplementary material for: Causal effects of potential risk factors on postpartum depression: a Mendelian randomization study
Source: Front Psychiatry. 2023 Dec 20;14:1275834. doi: 10.3389/fpsyt.2023.1275834 (PMC10761415; doi:10.3389/fpsyt.2023.1275834)
Supplement: Supplementary file 1 [file Table_1.docx]

**Supplementary Table 1. The heritability of PPD and 40 potential risk factors.**

| Traits | | Mean_chisq | Lambda_gc | Intercept | Intercept_se | Ratio | | Ratio_se | | H2_observed | H2_observed_se | H2_Z | H2_*p* |
| --- | --- | --- | --- | --- | --- | --- | --- | --- | --- | --- | --- | --- | --- |
| Outcome | PPD | 1.169 | 1.148 | 1.063 | 0.009 | | 0.371 | | 0.056 | 0.020 | 0.003 | 7.860 | 3.83E-15 |
| Psychiatric disorders | SCZ | 2.059 | 1.753 | 1.096 | 0.018 | | 0.090 | | 0.017 | 0.362 | 0.015 | 24.297 | 2.13E-130 |
|  | Autism Spectrum Disorder | 1.292 | 1.245 | 1.054 | 0.010 | | 0.185 | | 0.035 | 0.237 | 0.017 | 14.016 | 1.24E-44 |
|  | Bipolar disorder | 1.149 | 1.125 | 1.004 | 0.008 | | 0.027 | | 0.051 | 0.197 | 0.017 | 11.682 | 1.57E-31 |
|  | MD | 1.601 | 1.456 | 1.027 | 0.012 | | 0.045 | | 0.020 | 0.056 | 0.002 | 22.748 | 1.50E-114 |
|  | Attention deficit/hyperactivity disorder | 1.292 | 1.245 | 1.054 | 0.010 | | 0.185 | | 0.035 | 0.199 | 0.014 | 14.016 | 1.24E-44 |
|  | Anxiety | 1.029 | 1.035 | 1.006 | 0.006 | | 0.204 | | 0.221 | 0.058 | 0.026 | 2.253 | 0.024 |
| Overweight | BMI | 3.372 | 2.486 | 1.131 | 0.025 | | 0.055 | | 0.010 | 0.247 | 0.008 | 31.823 | 3.15E-222 |
| Blood pressure | Diastolic blood pressure | 5.196 | 2.742 | 1.951 | 0.307 | | 0.227 | | 0.073 | 0.183 | 0.034 | 5.381 | 7.40E-08 |
|  | Pulse pressure | 4.596 | 2.727 | 1.452 | 0.120 | | 0.126 | | 0.033 | 0.203 | 0.016 | 12.705 | 5.57E-37 |
|  | Systolic blood pressure | 4.944 | 2.836 | 1.710 | 0.164 | | 0.180 | | 0.042 | 0.204 | 0.020 | 10.388 | 2.80E-25 |
| Glucose | Glycated hemoglobin levels | 1.294 | 1.147 | 1.026 | 0.016 | | 0.088 | | 0.053 | 0.086 | 0.010 | 8.786 | 1.55E-18 |
|  | Fasting glucose | 1.295 | 1.153 | 1.034 | 0.021 | | 0.115 | | 0.070 | 0.063 | 0.008 | 8.379 | 5.33E-17 |
|  | Fasting insulin | 1.217 | 1.139 | 0.995 | 0.013 | | -0.021 | | 0.058 | 0.070 | 0.008 | 8.668 | 4.41E-18 |
|  | 2-hour glucose | 1.082 | 1.063 | 1.009 | 0.007 | | 0.109 | | 0.091 | 0.055 | 0.008 | 6.772 | 1.27E-11 |
| Lipids | Triglycerides | 2.583 | 1.800 | 1.231 | 0.060 | | 0.146 | | 0.038 | 0.150 | 0.013 | 11.895 | 1.26E-32 |
|  | LDL-c | 1.854 | 1.365 | 1.123 | 0.042 | | 0.144 | | 0.049 | 0.080 | 0.009 | 9.378 | 6.74E-21 |
|  | HDL-c | 2.875 | 1.932 | 1.307 | 0.083 | | 0.164 | | 0.044 | 0.192 | 0.014 | 13.971 | 2.36E-44 |
|  | Apolipoprotein A-I | 2.577 | 1.788 | 1.244 | 0.070 | | 0.154 | | 0.045 | 0.168 | 0.013 | 12.428 | 1.84E-35 |
|  | Apolipoprotein B | 1.938 | 1.388 | 1.152 | 0.045 | | 0.162 | | 0.048 | 0.088 | 0.010 | 9.255 | 2.14E-20 |
| Sex-hormones | Total Testosterone | 1.430 | 1.254 | 1.071 | 0.018 | | 0.166 | | 0.041 | 0.088 | 0.008 | 11.502 | 1.28E-30 |
|  | Bioavailable Testosterone | 1.537 | 1.346 | 1.088 | 0.018 | | 0.164 | | 0.033 | 0.121 | 0.009 | 13.690 | 1.16E-42 |
|  | Sex hormone binding globulin | 1.866 | 1.486 | 1.131 | 0.045 | | 0.151 | | 0.051 | 0.168 | 0.014 | 11.731 | 8.85E-32 |
|  | Oestradiol | 1.017 | 1.020 | 1.009 | 0.007 | | 0.522 | | 0.411 | 0.007 | 0.008 | 0.853 | 0.394 |
| Thyroid function | Free thyroxine | 1.077 | 1.065 | 1.026 | 0.008 | | 0.332 | | 0.104 | 0.090 | 0.020 | 4.390 | 1.14E-05 |
|  | Thyrotropin | 1.054 | 1.045 | 1.052 | 0.008 | | 0.958 | | 0.151 | 0.001 | 0.009 | 0.104 | 0.917 |
|  | Hypothyroidism | 1.054 | 1.045 | 1.052 | 0.008 | | 0.957 | | 0.151 | 0.001 | 0.010 | 0.108 | 0.914 |
|  | Hyperthyroidism^1^ | 1.039 | 1.028 | 1.059 | 0.008 | | 1.527 | | 0.211 | -0.020 | 0.009 | -2.313 | 0.021 |
| Inflammatory biomarkers | Serum 25-Hydroxyvitamin D levels adjusted BMI | 1.790 | 1.482 | 1.077 | 0.027 | | 0.097 | | 0.034 | 0.080 | 0.009 | 8.685 | 3.78E-18 |
|  | C-reactive protein levels | 2.443 | 1.683 | 1.028 | 0.051 | | 0.019 | | 0.035 | 0.121 | 0.010 | 12.110 | 9.30E-34 |
| Habits | SI | 1.749 | 1.497 | 0.945 | 0.013 | | -0.073 | | 0.017 | 0.063 | 0.002 | 27.495 | 2.03E-166 |
|  | Cigarettes per day | 1.310 | 1.224 | 0.976 | 0.012 | | -0.076 | | 0.038 | 0.048 | 0.003 | 14.980 | 9.95E-51 |
|  | Alcohol consumption | 1.457 | 1.317 | 0.943 | 0.011 | | -0.124 | | 0.024 | 0.026 | 0.001 | 19.725 | 1.32E-86 |
|  | Coffee intake | 1.467 | 1.357 | 1.073 | 0.012 | | 0.155 | | 0.027 | 0.045 | 0.004 | 12.236 | 2.00E-34 |
|  | Tea intake | 1.566 | 1.438 | 1.079 | 0.012 | | 0.139 | | 0.021 | 0.052 | 0.003 | 15.739 | 8.22E-56 |
| Socioeconomic Factors | Years of schooling | 2.671 | 2.110 | 1.062 | 0.022 | | 0.037 | | 0.013 | 0.106 | 0.003 | 30.707 | 4.53E-207 |
|  | Average total household income before tax | 1.634 | 1.507 | 1.124 | 0.012 | | 0.195 | | 0.019 | 0.063 | 0.003 | 19.954 | 1.39E-88 |
|  | Age at first sexual intercourse | 2.218 | 1.832 | 1.086 | 0.017 | | 0.071 | | 0.014 | 0.140 | 0.005 | 26.578 | 1.23E-155 |
|  | Age at first birth | 1.630 | 1.475 | 1.055 | 0.013 | | 0.087 | | 0.020 | 0.051 | 0.002 | 21.322 | 7.06E-101 |
|  | Age at menarche | 2.007 | 1.640 | 1.052 | 0.017 | | 0.052 | | 0.017 | 0.196 | 0.010 | 20.570 | 5.08E-94 |
| Sleep | Insomnia | 1.564 | 1.443 | 1.053 | 0.012 | | 0.094 | | 0.020 | 0.055 | 0.003 | 21.249 | 3.40E-100 |

Abbreviations: PPD = Postpartum depression. H2 = Heritability. MD = Major depression. BMI = Body mass index. SCZ = Schizophrenia. se = standard error. SI = Smoking initiation.
