# Supplementary material for: Causal effects of potential risk factors on postpartum depression: a Mendelian randomization study
Source: Front Psychiatry. 2023 Dec 20;14:1275834. doi: 10.3389/fpsyt.2023.1275834 (PMC10761415; doi:10.3389/fpsyt.2023.1275834)
Supplement: Supplementary file 2 [file Table_2.docx]

**Supplementary table 2.1. Detailed information of instrumental SNPs for SCZ in the analysis of PPD.**

| **Chr** | **Position** | **SNP** | **EA** | **OA** | **beta.exposure** | **se.exposure** | **pval.exposure** | **R2** | **F_val** | **beta.outcome** | **se.outcome** | **pval.outcome** | **Eaf.outcome** |
| --- | --- | --- | --- | --- | --- | --- | --- | --- | --- | --- | --- | --- | --- |
| 19 | 19518316 | rs1000237 | A | T | 0.073 | 0.009 | 2.80027e-16 | 0.0025 | 67.656 | -0.023 | 0.015 | 0.123 | 0.33700 |
| 5 | 45252500 | rs10035564 | G | A | 0.067 | 0.009 | 4.38026e-13 | 0.0020 | 52.724 | 0.000 | 0.014 | 0.983 | 0.33900 |
| 8 | 111580570 | rs10086619 | G | A | 0.072 | 0.012 | 4.96798e-10 | 0.0014 | 38.745 | -0.003 | 0.020 | 0.865 | 0.13820 |
| 5 | 137892170 | rs10117 | A | G | -0.055 | 0.009 | 4.65897e-10 | 0.0014 | 39.062 | 0.000 | 0.014 | 0.981 | 0.43640 |
| 12 | 104631552 | rs10861176 | A | G | 0.056 | 0.010 | 1.58800e-08 | 0.0012 | 32.075 | -0.009 | 0.016 | 0.573 | 0.74750 |
| 12 | 53760710 | rs10876446 | C | G | 0.054 | 0.009 | 1.02601e-08 | 0.0013 | 33.004 | 0.004 | 0.014 | 0.784 | 0.31810 |
| 8 | 65605878 | rs10957321 | A | G | 0.048 | 0.009 | 3.48498e-08 | 0.0011 | 30.628 | 0.015 | 0.014 | 0.269 | 0.50800 |
| 11 | 24389235 | rs11027839 | C | A | 0.052 | 0.009 | 2.39800e-09 | 0.0013 | 35.866 | -0.002 | 0.014 | 0.869 | 0.55170 |
| 8 | 144870701 | rs11136325 | A | G | -0.054 | 0.009 | 3.04600e-09 | 0.0014 | 34.948 | -0.005 | 0.013 | 0.718 | 0.57950 |
| 1 | 97878068 | rs11165867 | T | C | 0.074 | 0.012 | 1.29999e-10 | 0.0015 | 41.030 | -0.005 | 0.018 | 0.787 | 0.16100 |
| 10 | 104906211 | rs11191580 | C | T | -0.132 | 0.016 | 1.77215e-17 | 0.0028 | 72.198 | -0.030 | 0.024 | 0.208 | 0.08748 |
| 1 | 44100084 | rs11210892 | A | G | -0.064 | 0.009 | 2.67793e-12 | 0.0018 | 48.694 | -0.014 | 0.014 | 0.313 | 0.63820 |
| 20 | 62150128 | rs113264400 | C | T | 0.112 | 0.020 | 2.86497e-08 | 0.0012 | 30.905 | -0.025 | 0.019 | 0.205 | 0.05567 |
| 1 | 239198959 | rs11587347 | G | C | 0.104 | 0.015 | 1.52897e-12 | 0.0018 | 49.952 | -0.009 | 0.027 | 0.751 | 0.09642 |
| 18 | 77578986 | rs11664298 | A | G | 0.077 | 0.011 | 8.93511e-13 | 0.0019 | 51.360 | 0.037 | 0.016 | 0.020 | 0.16200 |
| 2 | 185601420 | rs11693094 | T | C | -0.054 | 0.009 | 4.29200e-10 | 0.0015 | 39.103 | -0.023 | 0.013 | 0.084 | 0.42250 |
| 20 | 48105317 | rs11696755 | C | T | 0.064 | 0.011 | 7.26006e-09 | 0.0012 | 33.531 | -0.020 | 0.019 | 0.313 | 0.19580 |
| 5 | 153680747 | rs11740474 | T | A | 0.054 | 0.009 | 1.12800e-09 | 0.0014 | 37.232 | 0.028 | 0.014 | 0.036 | 0.41150 |
| 4 | 31202669 | rs11941714 | A | G | -0.052 | 0.009 | 3.07298e-08 | 0.0012 | 30.779 | -0.011 | 0.014 | 0.437 | 0.38170 |
| 1 | 98552832 | rs1198588 | T | A | 0.103 | 0.011 | 1.73101e-21 | 0.0035 | 90.246 | 0.001 | 0.015 | 0.933 | 0.77040 |
| 1 | 73768366 | rs12129573 | A | C | 0.078 | 0.009 | 2.28192e-18 | 0.0028 | 76.414 | 0.048 | 0.014 | 0.000 | 0.39660 |
| 1 | 150115398 | rs12138231 | A | T | 0.067 | 0.012 | 7.99006e-09 | 0.0013 | 33.356 | -0.005 | 0.019 | 0.780 | 0.81410 |
| 2 | 198274929 | rs12151767 | A | G | -0.061 | 0.009 | 1.30707e-12 | 0.0019 | 50.484 | -0.014 | 0.014 | 0.293 | 0.51090 |
| 11 | 46343189 | rs12285419 | A | C | 0.085 | 0.011 | 1.04809e-14 | 0.0022 | 59.577 | 0.009 | 0.019 | 0.620 | 0.19180 |
| 11 | 124612932 | rs12293670 | G | A | -0.070 | 0.009 | 1.55704e-14 | 0.0022 | 58.715 | -0.017 | 0.014 | 0.228 | 0.35090 |
| 12 | 72259954 | rs12303743 | C | G | 0.087 | 0.015 | 1.59001e-09 | 0.0013 | 36.414 | -0.040 | 0.023 | 0.089 | 0.07555 |
| 3 | 16875823 | rs12489270 | C | T | 0.058 | 0.009 | 7.46793e-11 | 0.0016 | 42.330 | 0.008 | 0.014 | 0.540 | 0.36480 |
| 5 | 155775075 | rs12652777 | C | T | -0.049 | 0.009 | 1.52300e-08 | 0.0012 | 32.199 | -0.019 | 0.013 | 0.159 | 0.53880 |
| 2 | 22749726 | rs12712510 | C | T | -0.057 | 0.009 | 5.14399e-11 | 0.0016 | 43.531 | -0.008 | 0.013 | 0.540 | 0.52680 |
| 10 | 54063083 | rs12771371 | A | G | -0.052 | 0.009 | 1.93999e-08 | 0.0012 | 31.750 | 0.023 | 0.015 | 0.130 | 0.30320 |
| 12 | 124476873 | rs12833624 | T | C | 0.050 | 0.009 | 2.77300e-08 | 0.0011 | 31.111 | 0.027 | 0.015 | 0.063 | 0.36780 |
| 13 | 74325499 | rs12877581 | C | G | 0.060 | 0.010 | 1.80302e-09 | 0.0014 | 36.245 | 0.005 | 0.014 | 0.702 | 0.30720 |
| 14 | 33303540 | rs12883788 | T | C | 0.061 | 0.009 | 1.86209e-12 | 0.0019 | 49.648 | 0.017 | 0.013 | 0.218 | 0.47420 |
| 2 | 57961602 | rs13011472 | G | C | 0.070 | 0.009 | 4.28154e-16 | 0.0025 | 65.482 | 0.021 | 0.013 | 0.108 | 0.47020 |
| 2 | 145183851 | rs13016542 | C | T | -0.088 | 0.013 | 8.28133e-12 | 0.0018 | 46.858 | -0.027 | 0.019 | 0.152 | 0.14120 |
| 4 | 103188709 | rs13107325 | T | C | 0.159 | 0.017 | 2.90001e-21 | 0.0031 | 89.238 | 0.122 | 0.058 | 0.035 | 0.07952 |
| 6 | 27509493 | rs13195636 | C | A | -0.211 | 0.016 | 6.54636e-40 | 0.0073 | 175.278 | -0.025 | 0.031 | 0.412 | 0.07058 |
| 7 | 87244960 | rs13233308 | T | C | -0.049 | 0.009 | 1.74900e-08 | 0.0012 | 32.073 | -0.008 | 0.013 | 0.522 | 0.46620 |
| 22 | 39988175 | rs132582 | T | C | -0.051 | 0.009 | 3.25897e-09 | 0.0013 | 35.164 | 0.026 | 0.013 | 0.052 | 0.54370 |
| 4 | 167974142 | rs1427633 | C | G | -0.048 | 0.009 | 4.09704e-08 | 0.0011 | 30.130 | -0.019 | 0.013 | 0.169 | 0.60740 |
| 3 | 17868759 | rs1430894 | T | C | 0.053 | 0.009 | 6.15404e-10 | 0.0014 | 38.404 | 0.005 | 0.013 | 0.692 | 0.48110 |
| 1 | 243793012 | rs145071536 | C | T | 0.085 | 0.012 | 1.61510e-12 | 0.0022 | 50.292 | 0.012 | 0.018 | 0.512 | 0.19580 |
| 2 | 199990107 | rs1451488 | G | A | 0.071 | 0.009 | 4.47198e-16 | 0.0025 | 66.403 | 0.035 | 0.014 | 0.011 | 0.52580 |
| 16 | 58659307 | rs149165 | G | T | -0.048 | 0.009 | 3.00497e-08 | 0.0011 | 30.694 | -0.015 | 0.013 | 0.265 | 0.42640 |
| 14 | 99733384 | rs1540840 | C | G | -0.056 | 0.009 | 2.21197e-09 | 0.0015 | 35.871 | 0.011 | 0.013 | 0.427 | 0.47510 |
| 7 | 131619847 | rs1593304 | G | A | 0.064 | 0.011 | 7.44801e-09 | 0.0013 | 33.349 | -0.022 | 0.020 | 0.267 | 0.81710 |
| 3 | 117772036 | rs1604060 | G | A | 0.077 | 0.014 | 3.24101e-08 | 0.0012 | 30.411 | -0.001 | 0.016 | 0.964 | 0.87870 |
| 6 | 29748690 | rs1611236 | A | G | -0.055 | 0.010 | 8.47403e-09 | 0.0013 | 32.947 | 0.052 | 0.016 | 0.001 | 0.28230 |
| 12 | 123650335 | rs1615350 | T | C | -0.074 | 0.010 | 4.91813e-14 | 0.0021 | 56.409 | 0.029 | 0.016 | 0.078 | 0.72070 |
| 3 | 107379837 | rs167924 | G | A | 0.050 | 0.009 | 2.33900e-08 | 0.0012 | 31.111 | 0.026 | 0.013 | 0.056 | 0.63720 |
| 1 | 177276006 | rs16851048 | C | T | 0.074 | 0.011 | 4.14859e-12 | 0.0017 | 48.475 | -0.038 | 0.019 | 0.043 | 0.21670 |
| 5 | 88743219 | rs16867571 | G | A | -0.066 | 0.010 | 2.67602e-10 | 0.0016 | 39.913 | -0.010 | 0.016 | 0.548 | 0.21070 |
| 3 | 2547786 | rs17194490 | T | G | 0.078 | 0.012 | 1.79515e-11 | 0.0017 | 45.445 | 0.032 | 0.019 | 0.099 | 0.16000 |
| 10 | 3821561 | rs17731 | A | G | 0.052 | 0.009 | 4.36999e-09 | 0.0013 | 34.663 | 0.012 | 0.015 | 0.417 | 0.34890 |
| 12 | 2413803 | rs1860002 | T | C | -0.084 | 0.009 | 1.03896e-21 | 0.0035 | 92.776 | -0.016 | 0.013 | 0.226 | 0.54170 |
| 5 | 106766340 | rs187557 | T | C | -0.067 | 0.012 | 2.02899e-08 | 0.0011 | 31.412 | -0.044 | 0.021 | 0.037 | 0.84190 |
| 2 | 156835793 | rs1881046 | T | G | -0.051 | 0.009 | 3.39203e-08 | 0.0012 | 30.373 | 0.013 | 0.015 | 0.362 | 0.33500 |
| 1 | 66331478 | rs1892346 | A | T | 0.048 | 0.009 | 3.55697e-08 | 0.0012 | 30.253 | -0.007 | 0.013 | 0.619 | 0.58050 |
| 5 | 101723875 | rs1901512 | C | T | -0.058 | 0.009 | 5.72295e-10 | 0.0014 | 38.600 | -0.041 | 0.015 | 0.006 | 0.67890 |
| 7 | 71770973 | rs1914399 | G | C | -0.049 | 0.009 | 1.40101e-08 | 0.0012 | 31.857 | 0.024 | 0.013 | 0.072 | 0.57060 |
| 8 | 89283689 | rs1915019 | G | A | -0.057 | 0.010 | 6.57400e-09 | 0.0012 | 33.947 | -0.014 | 0.016 | 0.399 | 0.74060 |
| 14 | 30300361 | rs1953205 | A | T | 0.050 | 0.009 | 2.21498e-08 | 0.0012 | 31.442 | 0.005 | 0.013 | 0.687 | 0.46320 |
| 19 | 30987423 | rs2053079 | G | A | 0.060 | 0.010 | 3.00698e-09 | 0.0013 | 35.171 | -0.005 | 0.015 | 0.755 | 0.25940 |
| 9 | 138378856 | rs2078266 | G | A | -0.070 | 0.013 | 2.94097e-08 | 0.0014 | 30.513 | 0.024 | 0.018 | 0.191 | 0.84190 |
| 4 | 23423586 | rs215412 | A | G | 0.058 | 0.009 | 2.69197e-10 | 0.0015 | 40.209 | -0.005 | 0.014 | 0.714 | 0.30420 |
| 6 | 84341803 | rs217336 | A | C | -0.050 | 0.009 | 8.05100e-09 | 0.0012 | 33.431 | -0.024 | 0.013 | 0.077 | 0.44430 |
| 14 | 72417326 | rs2332700 | G | C | -0.075 | 0.010 | 3.87525e-14 | 0.0021 | 57.542 | -0.016 | 0.015 | 0.300 | 0.75350 |
| 4 | 176859992 | rs2333321 | G | A | -0.071 | 0.011 | 1.24796e-11 | 0.0017 | 45.986 | -0.043 | 0.017 | 0.010 | 0.79620 |
| 9 | 36319928 | rs2381411 | C | T | 0.050 | 0.009 | 1.25000e-08 | 0.0012 | 32.800 | -0.002 | 0.013 | 0.861 | 0.39860 |
| 15 | 78868398 | rs2456020 | T | C | -0.082 | 0.010 | 1.12512e-15 | 0.0024 | 63.997 | -0.010 | 0.015 | 0.494 | 0.24650 |
| 11 | 113392994 | rs2514218 | T | C | -0.070 | 0.009 | 1.34803e-14 | 0.0022 | 58.715 | -0.022 | 0.017 | 0.184 | 0.35190 |
| 17 | 44289832 | rs2696466 | G | A | -0.061 | 0.009 | 2.63694e-11 | 0.0018 | 44.249 | 0.040 | 0.014 | 0.004 | 0.44040 |
| 3 | 52815905 | rs2710323 | C | T | -0.078 | 0.009 | 1.22914e-19 | 0.0031 | 83.116 | -0.025 | 0.013 | 0.058 | 0.50500 |
| 6 | 73155285 | rs2815731 | A | C | -0.060 | 0.009 | 4.38834e-11 | 0.0017 | 43.478 | 0.003 | 0.013 | 0.821 | 0.34290 |
| 2 | 162845855 | rs2909457 | A | G | -0.049 | 0.009 | 1.48201e-08 | 0.0012 | 31.721 | 0.023 | 0.014 | 0.092 | 0.58350 |
| 14 | 51655145 | rs2999392 | T | C | 0.052 | 0.009 | 3.05000e-08 | 0.0011 | 30.366 | -0.001 | 0.015 | 0.925 | 0.68390 |
| 3 | 161487491 | rs308697 | A | C | -0.050 | 0.009 | 8.83202e-09 | 0.0012 | 33.166 | -0.025 | 0.014 | 0.075 | 0.45430 |
| 15 | 61872197 | rs35351411 | C | A | 0.064 | 0.009 | 2.21106e-13 | 0.0020 | 53.281 | 0.023 | 0.013 | 0.084 | 0.57260 |
| 7 | 110988593 | rs35426637 | T | G | -0.062 | 0.009 | 2.15080e-11 | 0.0017 | 44.873 | 0.014 | 0.014 | 0.318 | 0.34990 |
| 4 | 706700 | rs35734242 | C | T | 0.051 | 0.009 | 1.36801e-08 | 0.0013 | 32.457 | 0.010 | 0.013 | 0.460 | 0.44140 |
| 2 | 201253769 | rs3739118 | A | G | -0.057 | 0.010 | 2.36401e-09 | 0.0013 | 36.005 | 0.003 | 0.015 | 0.850 | 0.28330 |
| 2 | 37575381 | rs3770754 | G | C | -0.053 | 0.009 | 5.35205e-09 | 0.0013 | 33.788 | 0.004 | 0.013 | 0.747 | 0.37570 |
| 2 | 212290048 | rs3791710 | C | T | -0.060 | 0.011 | 3.02002e-08 | 0.0012 | 30.868 | -0.018 | 0.017 | 0.280 | 0.20080 |
| 1 | 8431607 | rs3795310 | T | C | -0.051 | 0.009 | 5.75003e-09 | 0.0013 | 34.360 | -0.007 | 0.014 | 0.607 | 0.49500 |
| 11 | 133827733 | rs3802924 | C | A | -0.074 | 0.011 | 9.58297e-12 | 0.0018 | 46.446 | -0.016 | 0.015 | 0.269 | 0.20280 |
| 16 | 29994922 | rs3814883 | T | C | -0.067 | 0.009 | 1.57616e-14 | 0.0022 | 59.481 | -0.032 | 0.013 | 0.019 | 0.46020 |
| 9 | 101071522 | rs3824451 | C | T | 0.066 | 0.012 | 2.53898e-08 | 0.0011 | 30.901 | 0.032 | 0.019 | 0.103 | 0.16100 |
| 8 | 143312933 | rs4129585 | C | A | -0.075 | 0.009 | 5.10858e-18 | 0.0028 | 74.309 | -0.034 | 0.014 | 0.012 | 0.55470 |
| 16 | 89559297 | rs4575535 | G | A | 0.056 | 0.010 | 5.77006e-09 | 0.0013 | 33.783 | 0.002 | 0.014 | 0.879 | 0.71370 |
| 18 | 50746748 | rs4632195 | T | C | 0.047 | 0.009 | 4.58596e-08 | 0.0011 | 30.118 | 0.061 | 0.013 | 0.000 | 0.49900 |
| 11 | 28642653 | rs4636654 | A | G | -0.048 | 0.009 | 4.89204e-08 | 0.0011 | 29.457 | -0.039 | 0.013 | 0.003 | 0.39460 |
| 1 | 36627542 | rs4653164 | T | C | 0.051 | 0.009 | 3.07603e-08 | 0.0012 | 30.855 | -0.007 | 0.014 | 0.589 | 0.65310 |
| 5 | 60621839 | rs4700418 | G | C | 0.070 | 0.009 | 5.36661e-16 | 0.0025 | 65.103 | -0.036 | 0.013 | 0.006 | 0.48010 |
| 15 | 91426560 | rs4702 | A | G | -0.084 | 0.009 | 2.79383e-21 | 0.0035 | 89.726 | -0.034 | 0.013 | 0.010 | 0.56260 |
| 12 | 110723245 | rs4766428 | T | C | 0.075 | 0.009 | 3.93097e-17 | 0.0028 | 71.021 | 0.033 | 0.013 | 0.014 | 0.45330 |
| 15 | 83368738 | rs4779050 | G | T | -0.058 | 0.009 | 7.27445e-11 | 0.0016 | 42.463 | 0.014 | 0.013 | 0.284 | 0.59640 |
| 20 | 37485458 | rs4812325 | A | G | 0.072 | 0.009 | 8.95983e-16 | 0.0024 | 65.272 | -0.014 | 0.014 | 0.316 | 0.57460 |
| 8 | 17070926 | rs4921741 | G | A | 0.056 | 0.010 | 1.20501e-08 | 0.0012 | 32.652 | -0.006 | 0.014 | 0.664 | 0.27930 |
| 9 | 14509105 | rs498591 | T | A | 0.072 | 0.012 | 2.11398e-09 | 0.0013 | 35.896 | 0.007 | 0.019 | 0.722 | 0.13920 |
| 9 | 77358745 | rs500102 | C | T | -0.052 | 0.009 | 4.87102e-09 | 0.0013 | 34.516 | 0.035 | 0.013 | 0.009 | 0.60340 |
| 9 | 22767164 | rs505061 | A | C | 0.053 | 0.009 | 5.80403e-10 | 0.0014 | 38.694 | 0.001 | 0.013 | 0.932 | 0.47120 |
| 15 | 40567237 | rs56205728 | A | G | 0.063 | 0.010 | 1.00700e-10 | 0.0016 | 42.188 | 0.020 | 0.016 | 0.210 | 0.28830 |
| 1 | 30427639 | rs56335113 | G | A | -0.065 | 0.009 | 6.01728e-12 | 0.0018 | 47.377 | -0.008 | 0.015 | 0.596 | 0.68990 |
| 17 | 19141582 | rs57433322 | G | C | -0.083 | 0.014 | 1.99200e-09 | 0.0015 | 35.741 | 0.028 | 0.018 | 0.128 | 0.11030 |
| 22 | 42370991 | rs5751191 | C | T | 0.066 | 0.009 | 3.00400e-14 | 0.0022 | 58.176 | 0.020 | 0.013 | 0.134 | 0.50990 |
| 7 | 2029867 | rs58120505 | C | T | -0.090 | 0.009 | 2.23512e-24 | 0.0039 | 103.676 | 0.013 | 0.014 | 0.348 | 0.42050 |
| 22 | 39247750 | rs6001259 | T | C | 0.192 | 0.035 | 3.69598e-08 | 0.0012 | 30.282 | 0.007 | 0.032 | 0.827 | 0.01491 |
| 22 | 51103091 | rs6010045 | C | T | 0.055 | 0.010 | 7.44492e-09 | 0.0013 | 33.396 | -0.007 | 0.016 | 0.656 | 0.70970 |
| 3 | 71563777 | rs60135207 | T | G | -0.050 | 0.009 | 1.52598e-08 | 0.0012 | 31.768 | -0.017 | 0.014 | 0.203 | 0.43640 |
| 12 | 57682956 | rs61937595 | T | C | -0.130 | 0.016 | 1.14710e-15 | 0.0028 | 64.493 | 0.004 | 0.019 | 0.825 | 0.09046 |
| 15 | 44080737 | rs62018952 | C | T | 0.058 | 0.010 | 1.94102e-09 | 0.0014 | 36.251 | 0.028 | 0.018 | 0.120 | 0.71870 |
| 2 | 172956449 | rs62183855 | C | A | -0.066 | 0.011 | 2.65797e-09 | 0.0014 | 35.458 | 0.029 | 0.026 | 0.254 | 0.19980 |
| 6 | 93077500 | rs634940 | T | G | 0.066 | 0.010 | 1.78402e-11 | 0.0017 | 44.980 | 0.029 | 0.016 | 0.073 | 0.24350 |
| 10 | 18726326 | rs6482437 | C | A | 0.099 | 0.014 | 3.32583e-12 | 0.0019 | 48.512 | -0.007 | 0.019 | 0.731 | 0.89560 |
| 12 | 95195293 | rs6538539 | T | G | -0.057 | 0.009 | 4.42792e-11 | 0.0016 | 43.615 | -0.004 | 0.013 | 0.779 | 0.53980 |
| 2 | 73837955 | rs6546857 | G | A | 0.060 | 0.010 | 2.74397e-09 | 0.0013 | 35.062 | -0.021 | 0.016 | 0.181 | 0.22370 |
| 3 | 30044778 | rs6549963 | C | T | -0.048 | 0.009 | 4.30804e-08 | 0.0011 | 30.130 | 0.001 | 0.014 | 0.926 | 0.40760 |
| 1 | 2373168 | rs6673880 | G | A | 0.062 | 0.009 | 7.19449e-12 | 0.0019 | 46.871 | 0.000 | 0.013 | 0.978 | 0.49200 |
| 2 | 2327295 | rs6715366 | A | G | 0.054 | 0.010 | 2.49201e-08 | 0.0011 | 31.103 | -0.003 | 0.015 | 0.858 | 0.24450 |
| 2 | 79426855 | rs6721531 | T | A | -0.052 | 0.009 | 1.46801e-08 | 0.0012 | 32.278 | -0.009 | 0.018 | 0.591 | 0.33900 |
| 3 | 63903759 | rs6798742 | G | A | 0.061 | 0.009 | 4.56878e-11 | 0.0016 | 43.162 | -0.004 | 0.014 | 0.789 | 0.33200 |
| 7 | 86403263 | rs6943762 | C | T | -0.105 | 0.013 | 1.56892e-15 | 0.0025 | 63.393 | 0.004 | 0.020 | 0.840 | 0.12920 |
| 7 | 110056000 | rs6974218 | C | A | -0.055 | 0.009 | 6.80393e-10 | 0.0014 | 38.044 | -0.006 | 0.013 | 0.676 | 0.42250 |
| 8 | 60700469 | rs6984242 | A | G | -0.055 | 0.009 | 3.85496e-10 | 0.0014 | 39.526 | -0.026 | 0.013 | 0.049 | 0.60340 |
| 11 | 57585662 | rs708228 | T | C | 0.053 | 0.009 | 6.55602e-09 | 0.0012 | 33.665 | 0.004 | 0.014 | 0.791 | 0.31310 |
| 11 | 130805334 | rs7112616 | C | T | -0.052 | 0.009 | 1.51702e-09 | 0.0014 | 36.847 | -0.024 | 0.013 | 0.067 | 0.49400 |
| 11 | 134247187 | rs7113199 | C | A | -0.052 | 0.009 | 2.80098e-08 | 0.0011 | 30.954 | 0.007 | 0.014 | 0.619 | 0.73560 |
| 19 | 50162909 | rs7251 | G | C | -0.064 | 0.009 | 8.29278e-12 | 0.0018 | 46.502 | -0.037 | 0.015 | 0.014 | 0.33300 |
| 5 | 152235215 | rs72802868 | T | G | -0.069 | 0.010 | 4.55407e-13 | 0.0020 | 51.959 | -0.024 | 0.015 | 0.099 | 0.28230 |
| 7 | 137072531 | rs728055 | A | T | -0.067 | 0.009 | 8.84912e-14 | 0.0021 | 56.078 | -0.022 | 0.014 | 0.111 | 0.35790 |
| 11 | 81178838 | rs72943392 | C | G | 0.053 | 0.010 | 2.39299e-08 | 0.0012 | 31.052 | -0.034 | 0.014 | 0.018 | 0.27440 |
| 19 | 11849736 | rs72986630 | T | C | 0.112 | 0.018 | 3.59402e-10 | 0.0016 | 39.357 | 0.016 | 0.027 | 0.558 | 0.05865 |
| 8 | 27442127 | rs73229090 | A | C | -0.103 | 0.014 | 4.33711e-13 | 0.0021 | 52.208 | 0.042 | 0.023 | 0.063 | 0.11330 |
| 17 | 12875908 | rs73292401 | A | T | 0.068 | 0.011 | 5.47999e-10 | 0.0014 | 38.468 | -0.002 | 0.018 | 0.928 | 0.17790 |
| 1 | 200414959 | rs7515363 | T | C | -0.054 | 0.009 | 1.83599e-09 | 0.0014 | 36.139 | -0.018 | 0.013 | 0.171 | 0.61630 |
| 2 | 97746526 | rs7575796 | G | A | -0.096 | 0.017 | 2.06500e-08 | 0.0016 | 31.347 | 0.003 | 0.016 | 0.872 | 0.07455 |
| 3 | 136398387 | rs7634476 | G | A | 0.058 | 0.009 | 5.46261e-11 | 0.0016 | 42.997 | 0.017 | 0.013 | 0.195 | 0.56560 |
| 3 | 180733150 | rs7647398 | T | C | -0.077 | 0.011 | 1.07399e-12 | 0.0019 | 50.551 | -0.015 | 0.019 | 0.425 | 0.20280 |
| 2 | 233743109 | rs778371 | G | A | 0.081 | 0.010 | 1.49486e-17 | 0.0027 | 71.987 | 0.000 | 0.015 | 0.987 | 0.29520 |
| 7 | 133128127 | rs7798283 | G | T | -0.074 | 0.013 | 3.48698e-08 | 0.0013 | 30.499 | 0.018 | 0.022 | 0.403 | 0.15810 |
| 7 | 24717969 | rs79210963 | C | T | 0.086 | 0.014 | 4.14200e-10 | 0.0014 | 39.041 | 0.009 | 0.022 | 0.663 | 0.10830 |
| 8 | 33863561 | rs79445414 | C | T | 0.123 | 0.022 | 2.79801e-08 | 0.0012 | 30.898 | 0.057 | 0.026 | 0.026 | 0.05169 |
| 16 | 13753384 | rs8055219 | A | G | 0.067 | 0.010 | 5.69246e-11 | 0.0016 | 43.355 | 0.008 | 0.016 | 0.589 | 0.25940 |
| 22 | 50290678 | rs8138941 | A | G | 0.058 | 0.011 | 4.46396e-08 | 0.0011 | 30.038 | 0.004 | 0.018 | 0.814 | 0.21070 |
| 18 | 27500959 | rs9304548 | A | C | -0.057 | 0.010 | 1.59401e-08 | 0.0012 | 32.151 | -0.015 | 0.015 | 0.307 | 0.74250 |
| 13 | 79930079 | rs9318627 | C | A | -0.061 | 0.009 | 4.35211e-12 | 0.0018 | 48.363 | -0.042 | 0.014 | 0.003 | 0.39360 |
| 6 | 70003389 | rs9454727 | G | A | -0.054 | 0.010 | 3.34996e-08 | 0.0012 | 30.817 | -0.029 | 0.015 | 0.047 | 0.26940 |
| 6 | 33796794 | rs9461916 | C | T | 0.053 | 0.009 | 1.64101e-09 | 0.0014 | 36.679 | 0.011 | 0.013 | 0.404 | 0.63620 |
| 18 | 53200117 | rs9636107 | G | A | 0.070 | 0.009 | 5.11446e-16 | 0.0024 | 66.057 | 0.036 | 0.013 | 0.007 | 0.49010 |
| 5 | 139065988 | rs9687282 | G | T | 0.053 | 0.009 | 7.32808e-09 | 0.0012 | 33.410 | -0.010 | 0.014 | 0.491 | 0.33400 |
| 3 | 36848316 | rs9876421 | T | C | 0.063 | 0.009 | 9.19179e-12 | 0.0018 | 46.156 | 0.004 | 0.014 | 0.787 | 0.34990 |

**Supplementary table 2.2. Detailed information of instrumental SNPs for Autism Spectrum Disorder** **in the analysis of PPD.**

| **Chr** | **Position** | **SNP** | **EA** | **OA** | **beta.exposure** | **se.exposure** | **pval.exposure** | **R2** | **F_val** | **beta.outcome** | **se.outcome** | **pval.outcome** | **Eaf.outcome** |
| --- | --- | --- | --- | --- | --- | --- | --- | --- | --- | --- | --- | --- | --- |
| 8 | 10576775 | rs10099100 | C | G | 0.084 | 0.015 | 1.06500e-08 | 0.0032 | 32.890 | -0.004 | 0.015 | 0.794 | 0.34100 |
| 8 | 131472047 | rs10110094 | G | A | -0.091 | 0.019 | 2.04998e-06 | 0.0020 | 22.550 | 0.007 | 0.020 | 0.726 | 0.85490 |
| 1 | 104792257 | rs11185408 | A | G | -0.069 | 0.014 | 6.98297e-07 | 0.0024 | 24.781 | -0.031 | 0.013 | 0.021 | 0.50890 |
| 14 | 94838142 | rs112635299 | T | G | 0.221 | 0.043 | 3.04397e-07 | 0.0016 | 26.170 | -0.004 | 0.048 | 0.941 | 0.01690 |
| 8 | 142615222 | rs11787216 | T | C | -0.069 | 0.015 | 2.58702e-06 | 0.0021 | 22.160 | -0.006 | 0.014 | 0.679 | 0.33300 |
| 6 | 23767038 | rs12203328 | C | G | 0.070 | 0.015 | 4.91496e-06 | 0.0018 | 20.755 | -0.012 | 0.015 | 0.439 | 0.24450 |
| 21 | 37255329 | rs144911765 | C | T | 0.190 | 0.040 | 2.36401e-06 | 0.0019 | 22.250 | -0.007 | 0.030 | 0.805 | 0.02684 |
| 3 | 62481063 | rs1452075 | T | C | 0.081 | 0.016 | 2.06900e-07 | 0.0027 | 27.110 | -0.017 | 0.015 | 0.235 | 0.71570 |
| 19 | 37980494 | rs149923766 | G | T | 0.237 | 0.048 | 9.61192e-07 | 0.0013 | 24.040 | 0.014 | 0.044 | 0.749 | 0.01193 |
| 6 | 16753147 | rs16879023 | A | G | -0.096 | 0.020 | 1.76498e-06 | 0.0024 | 22.714 | -0.006 | 0.021 | 0.757 | 0.15210 |
| 20 | 14760747 | rs2224274 | T | C | 0.071 | 0.014 | 2.85799e-07 | 0.0025 | 26.469 | -0.021 | 0.013 | 0.121 | 0.50200 |
| 1 | 96978961 | rs2391769 | G | A | 0.077 | 0.015 | 1.13501e-07 | 0.0026 | 28.128 | 0.005 | 0.015 | 0.727 | 0.67000 |
| 9 | 76179384 | rs28729902 | G | A | 0.084 | 0.018 | 2.34498e-06 | 0.0022 | 22.219 | 0.019 | 0.016 | 0.236 | 0.19580 |
| 18 | 55872558 | rs292441 | A | G | -0.072 | 0.015 | 1.12401e-06 | 0.0023 | 23.673 | -0.015 | 0.015 | 0.308 | 0.67300 |
| 5 | 103995368 | rs325485 | G | A | -0.073 | 0.014 | 3.25402e-07 | 0.0025 | 25.920 | -0.037 | 0.014 | 0.008 | 0.62620 |
| 12 | 73196902 | rs35404050 | T | C | 0.084 | 0.018 | 1.60602e-06 | 0.0024 | 22.944 | -0.015 | 0.017 | 0.396 | 0.21170 |
| 10 | 16691399 | rs45595836 | T | C | 0.139 | 0.027 | 3.13098e-07 | 0.0033 | 26.114 | 0.004 | 0.030 | 0.883 | 0.09344 |
| 10 | 130488026 | rs4750990 | C | T | 0.068 | 0.014 | 1.37101e-06 | 0.0022 | 23.325 | -0.029 | 0.013 | 0.030 | 0.38570 |
| 5 | 87854395 | rs4916723 | C | A | 0.067 | 0.014 | 1.92398e-06 | 0.0022 | 22.785 | -0.007 | 0.013 | 0.576 | 0.41350 |
| 11 | 102735140 | rs644552 | A | G | 0.159 | 0.035 | 4.21105e-06 | 0.0030 | 21.225 | 0.038 | 0.032 | 0.240 | 0.06262 |
| 1 | 193502609 | rs6692705 | G | A | -0.066 | 0.014 | 3.26302e-06 | 0.0020 | 21.646 | -0.018 | 0.014 | 0.201 | 0.60930 |
| 6 | 29575405 | rs740883 | T | A | 0.114 | 0.024 | 1.69399e-06 | 0.0023 | 22.821 | -0.016 | 0.029 | 0.596 | 0.09742 |
| 8 | 60390318 | rs76397219 | G | A | 0.140 | 0.030 | 3.56599e-06 | 0.0030 | 21.439 | -0.003 | 0.032 | 0.921 | 0.08250 |
| 13 | 66970212 | rs77691144 | C | T | 0.207 | 0.044 | 1.90999e-06 | 0.0026 | 22.733 | 0.008 | 0.062 | 0.896 | 0.03181 |
| 7 | 71646872 | rs7783557 | C | T | -0.067 | 0.015 | 4.36305e-06 | 0.0020 | 21.062 | -0.003 | 0.014 | 0.853 | 0.32110 |
| 15 | 93953737 | rs78058104 | A | G | 0.188 | 0.040 | 2.22101e-06 | 0.0022 | 22.401 | -0.010 | 0.040 | 0.804 | 0.03280 |
| 1 | 147183927 | rs78653484 | T | C | -0.176 | 0.038 | 4.67498e-06 | 0.0030 | 20.968 | -0.052 | 0.028 | 0.068 | 0.05070 |
| 10 | 72749037 | rs78827416 | A | G | 0.131 | 0.027 | 8.99995e-07 | 0.0023 | 24.070 | -0.020 | 0.022 | 0.351 | 0.07455 |
| 3 | 191838169 | rs79940520 | G | A | 0.095 | 0.021 | 4.26000e-06 | 0.0023 | 21.240 | 0.033 | 0.021 | 0.116 | 0.14810 |
| 20 | 21248116 | rs910805 | A | G | -0.096 | 0.016 | 2.04099e-09 | 0.0033 | 35.773 | 0.012 | 0.015 | 0.453 | 0.76440 |
| 6 | 11730878 | rs9366877 | G | A | -0.068 | 0.014 | 9.05295e-07 | 0.0023 | 24.285 | -0.013 | 0.014 | 0.339 | 0.42740 |
| 6 | 135035609 | rs9389208 | T | C | 0.067 | 0.014 | 3.12097e-06 | 0.0021 | 21.778 | 0.009 | 0.013 | 0.517 | 0.37670 |

**Supplementary table 2.3. Detailed information of instrumental SNPs for Bipolar disorder in the analysis of PPD.**

| **Chr** | **Position** | **SNP** | **EA** | **OA** | **beta.exposure** | **se.exposure** | **pval.exposure** | **R2** | **F_val** | **beta.outcome** | **se.outcome** | **pval.outcome** | **Eaf.outcome** |
| --- | --- | --- | --- | --- | --- | --- | --- | --- | --- | --- | --- | --- | --- |
| 3 | 36856030 | rs9834970 | C | T | 0.120 | 0.021 | 4.83404e-10 | 0.0072 | 33.775 | 0.004 | 0.014 | 0.769 | 0.417 |
| 6 | 98553894 | rs1487441 | A | G | 0.108 | 0.020 | 2.58297e-08 | 0.0059 | 28.184 | -0.010 | 0.013 | 0.444 | 0.480 |
| 7 | 1896413 | rs4236274 | G | A | -0.139 | 0.021 | 8.48790e-12 | 0.0092 | 41.978 | 0.010 | 0.014 | 0.467 | 0.305 |
| 8 | 19381917 | rs17480050 | G | T | 0.360 | 0.031 | 3.27567e-39 | 0.0366 | 135.885 | 0.031 | 0.017 | 0.069 | 0.178 |
| 9 | 23347865 | rs12553324 | G | C | 0.128 | 0.021 | 5.86894e-09 | 0.0079 | 37.216 | 0.008 | 0.013 | 0.544 | 0.441 |
| 12 | 49389320 | rs1054442 | C | A | 0.126 | 0.021 | 1.19600e-08 | 0.0075 | 34.887 | -0.010 | 0.013 | 0.434 | 0.430 |
| 17 | 37846512 | rs2517959 | T | A | -0.115 | 0.024 | 4.53304e-09 | 0.0058 | 23.621 | -0.028 | 0.015 | 0.051 | 0.700 |

**Supplementary table 2.4. Detailed information of instrumental SNPs for MD in the analysis of PPD.**

| **Chr** | **Position** | **SNP** | **EA** | **OA** | **beta.exposure** | **se.exposure** | **pval.exposure** | **R2** | **F_val** | **beta.outcome** | **se.outcome** | **pval.outcome** | **Eaf.outcome** |
| --- | --- | --- | --- | --- | --- | --- | --- | --- | --- | --- | --- | --- | --- |
| 10 | 106610839 | rs1021363 | G | A | -0.030 | 0.004 | 2.28718e-11 | 4e-04 | 44.444 | -0.038 | 0.015 | 0.011 | 0.66200 |
| 7 | 2086814 | rs10235664 | C | T | -0.027 | 0.005 | 4.67703e-08 | 3e-04 | 30.362 | -0.016 | 0.014 | 0.246 | 0.26940 |
| 1 | 175913828 | rs10913112 | T | C | -0.026 | 0.004 | 4.52501e-09 | 3e-04 | 33.898 | -0.017 | 0.014 | 0.211 | 0.39860 |
| 16 | 13800430 | rs12919291 | C | G | 0.033 | 0.005 | 3.09200e-09 | 3e-04 | 35.348 | 0.018 | 0.018 | 0.318 | 0.20180 |
| 18 | 53099012 | rs12967143 | C | G | -0.034 | 0.005 | 2.52697e-13 | 5e-04 | 53.882 | -0.033 | 0.015 | 0.030 | 0.69780 |
| 20 | 44692598 | rs13037326 | T | C | 0.031 | 0.005 | 2.39800e-10 | 4e-04 | 40.025 | 0.022 | 0.015 | 0.138 | 0.26140 |
| 18 | 50861409 | rs1367635 | C | T | 0.025 | 0.004 | 4.35402e-09 | 3e-04 | 34.618 | 0.044 | 0.013 | 0.001 | 0.49900 |
| 6 | 27182377 | rs150186873 | C | A | 0.070 | 0.012 | 4.51305e-09 | 3e-04 | 34.418 | 0.045 | 0.050 | 0.367 | 0.01690 |
| 7 | 117625599 | rs150346963 | T | C | 0.028 | 0.004 | 1.15699e-10 | 4e-04 | 41.368 | 0.015 | 0.013 | 0.271 | 0.39860 |
| 1 | 197704717 | rs17641524 | T | C | -0.030 | 0.005 | 1.50200e-08 | 3e-04 | 32.040 | -0.007 | 0.019 | 0.716 | 0.18290 |
| 9 | 11203149 | rs1931388 | G | A | -0.029 | 0.004 | 1.67996e-11 | 4e-04 | 44.951 | -0.019 | 0.013 | 0.148 | 0.40460 |
| 14 | 42097937 | rs1950829 | G | A | -0.030 | 0.004 | 4.73805e-12 | 4e-04 | 47.706 | -0.023 | 0.013 | 0.077 | 0.51890 |
| 11 | 61471678 | rs198457 | T | C | -0.032 | 0.006 | 1.89998e-08 | 3e-04 | 31.641 | 0.017 | 0.022 | 0.460 | 0.19090 |
| 2 | 208049581 | rs2111592 | A | G | 0.026 | 0.005 | 1.34999e-08 | 3e-04 | 32.689 | -0.001 | 0.014 | 0.939 | 0.32410 |
| 6 | 67000001 | rs2214123 | G | A | -0.026 | 0.004 | 8.55598e-09 | 3e-04 | 33.640 | -0.003 | 0.014 | 0.801 | 0.63020 |
| 6 | 28366151 | rs2232423 | G | A | -0.062 | 0.007 | 1.13501e-18 | 7e-04 | 78.449 | -0.017 | 0.031 | 0.573 | 0.06859 |
| 9 | 119731359 | rs2418449 | C | T | -0.028 | 0.005 | 4.24502e-09 | 3e-04 | 34.271 | -0.026 | 0.014 | 0.070 | 0.29030 |
| 5 | 87630769 | rs247910 | G | A | 0.024 | 0.004 | 4.71205e-08 | 3e-04 | 30.378 | 0.008 | 0.013 | 0.541 | 0.48610 |
| 7 | 82448100 | rs2522831 | C | T | 0.024 | 0.004 | 2.11300e-08 | 3e-04 | 31.152 | 0.024 | 0.013 | 0.072 | 0.46920 |
| 1 | 72765116 | rs2568958 | A | G | 0.038 | 0.004 | 2.90202e-18 | 7e-04 | 75.374 | 0.026 | 0.014 | 0.065 | 0.64210 |
| 15 | 88945878 | rs28541419 | G | C | -0.029 | 0.005 | 1.75598e-08 | 3e-04 | 31.533 | -0.044 | 0.018 | 0.015 | 0.20680 |
| 6 | 142996618 | rs2876520 | G | C | 0.026 | 0.004 | 2.24099e-09 | 3e-04 | 36.560 | 0.022 | 0.013 | 0.103 | 0.48710 |
| 5 | 103972357 | rs30266 | A | G | 0.037 | 0.005 | 1.42791e-15 | 6e-04 | 63.306 | 0.037 | 0.015 | 0.012 | 0.31610 |
| 1 | 49675276 | rs354155 | C | G | -0.045 | 0.007 | 1.75102e-09 | 3e-04 | 35.840 | -0.020 | 0.018 | 0.262 | 0.10930 |
| 7 | 12250402 | rs3807865 | A | G | 0.031 | 0.004 | 1.09295e-12 | 5e-04 | 49.638 | 0.022 | 0.014 | 0.106 | 0.40160 |
| 1 | 18122009 | rs4141983 | C | T | -0.026 | 0.005 | 9.69192e-09 | 3e-04 | 32.938 | 0.011 | 0.014 | 0.415 | 0.32900 |
| 11 | 88756779 | rs4497414 | C | T | 0.029 | 0.004 | 2.92685e-11 | 4e-04 | 43.740 | 0.003 | 0.013 | 0.816 | 0.45530 |
| 7 | 109100414 | rs4730387 | A | T | 0.024 | 0.004 | 4.12003e-08 | 3e-04 | 30.635 | 0.003 | 0.013 | 0.850 | 0.47710 |
| 18 | 35155910 | rs4799949 | T | C | -0.029 | 0.005 | 1.40301e-10 | 4e-04 | 40.295 | -0.021 | 0.015 | 0.159 | 0.66100 |
| 11 | 113365141 | rs4936276 | C | G | 0.028 | 0.004 | 3.57001e-10 | 4e-04 | 39.919 | 0.019 | 0.017 | 0.239 | 0.63820 |
| 13 | 80921519 | rs508502 | T | C | -0.026 | 0.005 | 3.55599e-08 | 3e-04 | 30.250 | -0.025 | 0.014 | 0.075 | 0.28530 |
| 7 | 38724868 | rs59082935 | T | C | 0.036 | 0.007 | 3.07100e-08 | 3e-04 | 30.250 | 0.025 | 0.019 | 0.178 | 0.14810 |
| 12 | 52352301 | rs61914045 | A | G | 0.031 | 0.005 | 7.96398e-09 | 3e-04 | 32.744 | 0.021 | 0.017 | 0.219 | 0.20970 |
| 9 | 37182655 | rs62535714 | A | G | 0.034 | 0.006 | 4.68598e-09 | 3e-04 | 34.162 | 0.045 | 0.015 | 0.003 | 0.14710 |
| 3 | 117515519 | rs66511648 | C | T | 0.030 | 0.005 | 6.03004e-10 | 4e-04 | 38.285 | -0.006 | 0.016 | 0.715 | 0.23860 |
| 14 | 75125540 | rs7152906 | C | T | 0.026 | 0.004 | 1.87301e-09 | 3e-04 | 36.000 | 0.018 | 0.013 | 0.165 | 0.53380 |
| 18 | 77580712 | rs7241572 | A | G | 0.032 | 0.005 | 2.43299e-09 | 3e-04 | 35.778 | 0.038 | 0.016 | 0.020 | 0.16000 |
| 2 | 212618440 | rs72948506 | A | G | 0.026 | 0.005 | 1.71498e-08 | 3e-04 | 31.790 | 0.041 | 0.014 | 0.004 | 0.26040 |
| 1 | 67132262 | rs7538938 | C | T | 0.025 | 0.004 | 7.28903e-09 | 3e-04 | 34.073 | 0.015 | 0.013 | 0.245 | 0.55770 |
| 14 | 103997525 | rs754287 | A | T | -0.029 | 0.004 | 1.31099e-10 | 4e-04 | 41.245 | -0.010 | 0.014 | 0.486 | 0.36980 |
| 1 | 52274078 | rs7551758 | G | T | 0.028 | 0.004 | 5.10740e-11 | 4e-04 | 43.315 | 0.022 | 0.013 | 0.094 | 0.55570 |
| 3 | 115977242 | rs76954012 | A | T | 0.041 | 0.007 | 2.41002e-08 | 3e-04 | 30.998 | 0.063 | 0.029 | 0.030 | 0.09940 |
| 5 | 164487555 | rs7725715 | A | G | 0.029 | 0.004 | 1.60694e-11 | 4e-04 | 45.484 | 0.064 | 0.014 | 0.000 | 0.52390 |
| 3 | 61255413 | rs843812 | A | G | 0.025 | 0.004 | 1.40501e-08 | 3e-04 | 31.769 | 0.031 | 0.013 | 0.020 | 0.39360 |
| 6 | 165117329 | rs9364755 | G | A | 0.028 | 0.005 | 3.48602e-08 | 3e-04 | 30.792 | 0.005 | 0.015 | 0.737 | 0.23660 |
| 13 | 31790053 | rs9529218 | T | C | -0.034 | 0.005 | 2.23100e-10 | 4e-04 | 39.643 | -0.004 | 0.015 | 0.772 | 0.20180 |
| 13 | 53860655 | rs9536381 | T | C | 0.025 | 0.005 | 2.61698e-08 | 3e-04 | 30.730 | 0.019 | 0.014 | 0.183 | 0.31910 |
| 3 | 49214303 | rs9831648 | T | G | -0.029 | 0.005 | 1.58599e-08 | 3e-04 | 31.533 | -0.042 | 0.018 | 0.022 | 0.78830 |

**Supplementary table 2.5. Detailed information of instrumental SNPs for Attention deficit/hyperactivity disorder** **in the analysis of PPD.**

| **Chr** | **Position** | **SNP** | **EA** | **OA** | **beta.exposure** | **se.exposure** | **pval.exposure** | **R2** | **F_val** | **beta.outcome** | **se.outcome** | **pval.outcome** | **Eaf.outcome** |
| --- | --- | --- | --- | --- | --- | --- | --- | --- | --- | --- | --- | --- | --- |
| 7 | 114091753 | rs10262192 | A | G | 0.073 | 0.013 | 2.88702e-08 | 0.0026 | 30.755 | -0.010 | 0.013 | 0.473 | 0.42640 |
| 1 | 44173423 | rs112984125 | A | G | -0.106 | 0.015 | 3.58096e-13 | 0.0046 | 52.717 | 0.001 | 0.014 | 0.957 | 0.29130 |
| 10 | 106747354 | rs11591402 | A | T | -0.093 | 0.016 | 1.33601e-08 | 0.0031 | 32.092 | -0.042 | 0.015 | 0.006 | 0.22960 |
| 12 | 89760744 | rs1427829 | G | A | -0.080 | 0.013 | 1.82201e-09 | 0.0031 | 36.091 | -0.022 | 0.014 | 0.112 | 0.55770 |
| 16 | 72578131 | rs212178 | A | G | -0.115 | 0.020 | 7.67697e-09 | 0.0024 | 33.293 | 0.011 | 0.028 | 0.684 | 0.89860 |
| 3 | 20669071 | rs4858241 | G | T | -0.079 | 0.014 | 1.74000e-08 | 0.0028 | 31.764 | 0.011 | 0.014 | 0.419 | 0.33000 |
| 5 | 87854395 | rs4916723 | C | A | 0.077 | 0.014 | 1.57601e-08 | 0.0028 | 32.195 | -0.007 | 0.013 | 0.576 | 0.41350 |
| 8 | 34352610 | rs74760947 | G | A | 0.180 | 0.032 | 1.34800e-08 | 0.0036 | 32.170 | 0.057 | 0.026 | 0.028 | 0.05964 |
| 2 | 215181889 | rs9677504 | A | G | 0.117 | 0.021 | 1.39101e-08 | 0.0023 | 32.205 | 0.007 | 0.021 | 0.751 | 0.09245 |

**Supplementary table 2.6. Detailed information of instrumental SNPs for Anixety in the analysis of PPD.**

| **Chr** | **Position** | **SNP** | **EA** | **OA** | **beta.exposure** | **se.exposure** | **pval.exposure** | **R2** | **F_val** | **beta.outcome** | **se.outcome** | **pval.outcome** | **Eaf.outcome** |
| --- | --- | --- | --- | --- | --- | --- | --- | --- | --- | --- | --- | --- | --- |
| 3 | 101699154 | rs1709393 | C | T | -0.151 | 0.027 | 1.651e-08 | 0.0111 | 31.942 | 0.022 | 0.013 | 0.092 | 0.58150 |
| 6 | 11167111 | rs2146346 | G | A | 0.144 | 0.030 | 1.375e-06 | 0.0101 | 23.383 | 0.012 | 0.014 | 0.372 | 0.42840 |
| 6 | 47941339 | rs2753188 | G | A | 0.160 | 0.033 | 1.417e-06 | 0.0102 | 23.313 | -0.006 | 0.014 | 0.675 | 0.71670 |
| 16 | 88815473 | rs28373923 | G | A | 0.419 | 0.092 | 4.558e-06 | 0.0221 | 20.999 | -0.001 | 0.026 | 0.964 | 0.07555 |
| 11 | 116509339 | rs58990403 | G | A | -0.185 | 0.039 | 2.163e-06 | 0.0113 | 22.411 | -0.013 | 0.016 | 0.418 | 0.20870 |
| 22 | 25617602 | rs739315 | G | A | -0.154 | 0.033 | 3.027e-06 | 0.0116 | 21.825 | -0.002 | 0.013 | 0.886 | 0.42840 |

**Supplementary table 2.7. Detailed information of instrumental SNPs for BMI in the analysis of PPD.**

| **Chr** | **Position** | **SNP** | **EA** | **OA** | **beta.exposure** | **se.exposure** | **pval.exposure** | **R2** | **F_val** | **beta.outcome** | **se.outcome** | **pval.outcome** | **Eaf.outcome** |
| --- | --- | --- | --- | --- | --- | --- | --- | --- | --- | --- | --- | --- | --- |
| 5 | 140990108 | rs10063055 | T | C | 0.013 | 0.002 | 1.40001e-08 | 0.0001 | 32.184 | 0.013 | 0.016 | 0.418 | 0.23760 |
| 8 | 143383694 | rs10099330 | G | A | 0.012 | 0.002 | 3.69999e-09 | 0.0001 | 34.751 | -0.019 | 0.013 | 0.152 | 0.43840 |
| 11 | 76474827 | rs10160769 | C | G | -0.015 | 0.002 | 4.00000e-10 | 0.0001 | 39.100 | -0.017 | 0.016 | 0.293 | 0.24850 |
| 2 | 41637688 | rs10169594 | C | T | 0.012 | 0.002 | 1.79999e-08 | 0.0001 | 31.656 | -0.026 | 0.014 | 0.056 | 0.37080 |
| 2 | 208307761 | rs10172070 | T | C | 0.016 | 0.003 | 9.59997e-09 | 0.0001 | 32.930 | 0.008 | 0.021 | 0.694 | 0.12820 |
| 17 | 27912415 | rs1017529 | A | C | 0.015 | 0.003 | 1.20000e-08 | 0.0001 | 32.484 | -0.001 | 0.019 | 0.947 | 0.17000 |
| 2 | 104242992 | rs10182416 | G | A | 0.013 | 0.002 | 1.80011e-11 | 0.0001 | 45.151 | 0.000 | 0.013 | 0.995 | 0.56560 |
| 19 | 1950042 | rs10417386 | C | T | 0.012 | 0.002 | 1.29999e-08 | 0.0001 | 32.341 | 0.021 | 0.014 | 0.142 | 0.67990 |
| 19 | 46182304 | rs10423928 | A | T | -0.033 | 0.003 | 2.19989e-40 | 0.0004 | 176.960 | 0.005 | 0.015 | 0.738 | 0.21370 |
| 11 | 115044850 | rs1048932 | A | C | -0.015 | 0.002 | 2.09991e-14 | 0.0001 | 58.476 | -0.017 | 0.013 | 0.213 | 0.42940 |
| 12 | 19288508 | rs10505836 | C | A | 0.018 | 0.003 | 7.59994e-10 | 0.0001 | 37.848 | -0.015 | 0.019 | 0.455 | 0.84590 |
| 10 | 118650996 | rs10510025 | T | C | 0.018 | 0.002 | 5.00035e-15 | 0.0001 | 61.275 | -0.020 | 0.015 | 0.196 | 0.26040 |
| 2 | 198950240 | rs1064213 | A | G | 0.015 | 0.002 | 6.59933e-14 | 0.0001 | 56.180 | -0.025 | 0.014 | 0.067 | 0.52390 |
| 11 | 45438374 | rs10742752 | C | T | 0.012 | 0.002 | 1.00000e-08 | 0.0001 | 32.757 | -0.038 | 0.013 | 0.005 | 0.61930 |
| 9 | 16726119 | rs10756792 | T | C | -0.019 | 0.002 | 2.39994e-17 | 0.0001 | 71.760 | -0.024 | 0.016 | 0.130 | 0.74160 |
| 9 | 126093999 | rs10760277 | T | C | 0.014 | 0.002 | 7.89951e-12 | 0.0001 | 46.781 | 0.013 | 0.014 | 0.340 | 0.38770 |
| 10 | 88096047 | rs10887578 | C | G | 0.013 | 0.002 | 8.40040e-11 | 0.0001 | 42.158 | -0.013 | 0.013 | 0.335 | 0.47610 |
| 10 | 2569491 | rs10903791 | A | G | 0.011 | 0.002 | 4.49997e-08 | 0.0001 | 29.931 | -0.016 | 0.014 | 0.238 | 0.60740 |
| 1 | 243557659 | rs10927006 | C | T | -0.017 | 0.003 | 1.60000e-09 | 0.0001 | 36.356 | -0.019 | 0.018 | 0.283 | 0.12820 |
| 4 | 45186139 | rs10938398 | A | G | 0.029 | 0.002 | 4.00037e-48 | 0.0004 | 212.480 | -0.022 | 0.013 | 0.098 | 0.42050 |
| 7 | 71630362 | rs10950301 | A | G | -0.016 | 0.003 | 3.79997e-10 | 0.0001 | 39.198 | -0.019 | 0.017 | 0.247 | 0.18990 |
| 10 | 76084111 | rs11000993 | C | T | 0.021 | 0.003 | 1.80011e-12 | 0.0001 | 49.641 | -0.018 | 0.026 | 0.490 | 0.11830 |
| 10 | 21830104 | rs11012732 | G | A | 0.021 | 0.002 | 3.29989e-24 | 0.0002 | 103.033 | 0.005 | 0.014 | 0.713 | 0.35190 |
| 10 | 132954191 | rs11017771 | C | G | -0.014 | 0.002 | 2.00000e-08 | 0.0001 | 31.489 | -0.003 | 0.020 | 0.889 | 0.19580 |
| 11 | 17395540 | rs11024271 | C | T | 0.012 | 0.002 | 2.00000e-08 | 0.0001 | 31.517 | -0.009 | 0.013 | 0.507 | 0.64120 |
| 8 | 142619234 | rs1106761 | A | G | 0.015 | 0.002 | 1.69981e-12 | 0.0001 | 49.778 | -0.008 | 0.014 | 0.570 | 0.35490 |
| 15 | 62273994 | rs11071646 | A | G | -0.040 | 0.007 | 4.79999e-09 | 0.0001 | 34.254 | -0.048 | 0.028 | 0.091 | 0.02485 |
| 17 | 47090785 | rs11079849 | T | C | -0.020 | 0.002 | 6.29941e-22 | 0.0002 | 92.642 | 0.005 | 0.015 | 0.752 | 0.29030 |
| 4 | 130724902 | rs11099020 | T | C | -0.014 | 0.002 | 4.90004e-12 | 0.0001 | 47.745 | 0.017 | 0.014 | 0.214 | 0.62720 |
| 12 | 82424100 | rs11115160 | A | G | -0.013 | 0.002 | 2.39999e-08 | 0.0001 | 31.138 | -0.020 | 0.016 | 0.221 | 0.21870 |
| 1 | 230301811 | rs11122450 | G | T | -0.012 | 0.002 | 1.40001e-08 | 0.0001 | 32.144 | 0.010 | 0.013 | 0.452 | 0.60930 |
| 5 | 170623391 | rs11134679 | G | A | 0.019 | 0.002 | 5.50047e-18 | 0.0001 | 74.679 | -0.004 | 0.014 | 0.777 | 0.67990 |
| 17 | 78757626 | rs11150745 | G | A | -0.021 | 0.002 | 2.29985e-22 | 0.0002 | 94.634 | -0.002 | 0.015 | 0.906 | 0.31510 |
| 1 | 96924097 | rs11165643 | T | C | 0.020 | 0.002 | 7.89951e-23 | 0.0002 | 96.735 | -0.005 | 0.014 | 0.693 | 0.58250 |
| 5 | 27175962 | rs111689389 | C | G | -0.014 | 0.002 | 7.39997e-10 | 0.0001 | 37.912 | -0.008 | 0.016 | 0.600 | 0.27440 |
| 8 | 10802001 | rs11250094 | C | G | -0.021 | 0.002 | 4.10015e-25 | 0.0002 | 107.169 | 0.001 | 0.013 | 0.967 | 0.53280 |
| 12 | 49399132 | rs1126930 | C | G | 0.032 | 0.005 | 4.60002e-09 | 0.0001 | 34.339 | -0.017 | 0.038 | 0.652 | 0.02386 |
| 4 | 147354089 | rs113079574 | T | C | -0.016 | 0.003 | 2.19999e-10 | 0.0001 | 40.275 | -0.007 | 0.016 | 0.662 | 0.20580 |
| 1 | 39564930 | rs113603865 | T | C | 0.019 | 0.002 | 2.49977e-14 | 0.0001 | 58.110 | 0.017 | 0.017 | 0.309 | 0.20180 |
| 14 | 88326386 | rs113624107 | A | G | 0.014 | 0.002 | 1.50000e-09 | 0.0001 | 36.553 | -0.020 | 0.016 | 0.225 | 0.22960 |
| 7 | 138817193 | rs11525873 | C | T | -0.024 | 0.003 | 8.49963e-13 | 0.0001 | 51.168 | -0.023 | 0.019 | 0.212 | 0.09642 |
| 12 | 121671133 | rs11610621 | A | T | 0.016 | 0.003 | 5.00000e-09 | 0.0001 | 34.180 | -0.002 | 0.019 | 0.934 | 0.12130 |
| 5 | 50723410 | rs116374395 | A | G | 0.032 | 0.005 | 3.59998e-09 | 0.0001 | 34.820 | -0.041 | 0.038 | 0.282 | 0.04076 |
| 1 | 110131521 | rs116377258 | G | A | 0.067 | 0.006 | 1.59993e-26 | 0.0002 | 113.567 | 0.001 | 0.029 | 0.978 | 0.02982 |
| 15 | 66600096 | rs11639144 | A | G | -0.014 | 0.002 | 8.10009e-09 | 0.0001 | 33.259 | 0.007 | 0.015 | 0.633 | 0.24060 |
| 1 | 49996959 | rs1167311 | A | G | -0.019 | 0.002 | 1.59993e-19 | 0.0002 | 81.631 | 0.000 | 0.014 | 0.993 | 0.69780 |
| 2 | 204053742 | rs11675464 | G | A | 0.012 | 0.002 | 2.80001e-09 | 0.0001 | 35.336 | 0.026 | 0.013 | 0.049 | 0.55470 |
| 2 | 100805996 | rs11691869 | A | C | -0.020 | 0.002 | 1.90020e-21 | 0.0002 | 90.483 | -0.028 | 0.014 | 0.042 | 0.35190 |
| 3 | 131551027 | rs11709402 | G | A | 0.023 | 0.002 | 9.49948e-25 | 0.0002 | 105.506 | 0.012 | 0.015 | 0.437 | 0.26540 |
| 10 | 131783328 | rs117118217 | C | G | 0.044 | 0.008 | 2.30001e-08 | 0.0001 | 31.263 | -0.034 | 0.047 | 0.472 | 0.01491 |
| 16 | 54267868 | rs117342986 | T | C | 0.037 | 0.007 | 1.79999e-08 | 0.0001 | 31.646 | -0.037 | 0.062 | 0.546 | 0.02982 |
| 6 | 13180454 | rs11757278 | C | T | -0.015 | 0.002 | 1.00000e-11 | 0.0001 | 46.248 | -0.028 | 0.014 | 0.038 | 0.32110 |
| 17 | 2168104 | rs118136827 | T | G | -0.014 | 0.002 | 1.09999e-09 | 0.0001 | 37.175 | 0.020 | 0.015 | 0.165 | 0.28030 |
| 13 | 112191837 | rs1183668 | G | C | -0.016 | 0.002 | 1.90020e-14 | 0.0001 | 58.663 | -0.005 | 0.014 | 0.697 | 0.37670 |
| 13 | 31042452 | rs11842871 | T | G | -0.015 | 0.002 | 1.99986e-11 | 0.0001 | 44.948 | 0.007 | 0.014 | 0.609 | 0.26140 |
| 3 | 85880768 | rs11914525 | G | A | -0.018 | 0.002 | 3.69999e-18 | 0.0001 | 75.471 | -0.009 | 0.013 | 0.514 | 0.38470 |
| 3 | 48085349 | rs11919665 | T | A | -0.013 | 0.002 | 1.09999e-09 | 0.0001 | 37.209 | 0.001 | 0.015 | 0.941 | 0.67100 |
| 9 | 34074476 | rs12001437 | C | T | 0.012 | 0.002 | 2.19999e-09 | 0.0001 | 35.779 | 0.003 | 0.014 | 0.838 | 0.38270 |
| 1 | 98315893 | rs12072739 | G | A | 0.016 | 0.002 | 1.20005e-11 | 0.0001 | 46.042 | 0.017 | 0.014 | 0.244 | 0.21970 |
| 1 | 91189933 | rs12089815 | A | G | -0.013 | 0.002 | 1.89998e-10 | 0.0001 | 40.534 | -0.027 | 0.014 | 0.046 | 0.50100 |
| 1 | 62579891 | rs12140153 | T | G | -0.034 | 0.003 | 5.70033e-22 | 0.0002 | 92.813 | -0.018 | 0.025 | 0.478 | 0.06660 |
| 16 | 70309237 | rs12149660 | A | G | -0.022 | 0.003 | 1.59993e-12 | 0.0001 | 49.980 | 0.021 | 0.021 | 0.325 | 0.11030 |
| 8 | 67218772 | rs12156160 | G | A | 0.016 | 0.003 | 1.60000e-08 | 0.0001 | 31.893 | 0.069 | 0.022 | 0.002 | 0.11430 |
| 10 | 53680099 | rs12259464 | A | G | 0.013 | 0.002 | 5.70033e-11 | 0.0001 | 42.919 | 0.001 | 0.013 | 0.936 | 0.44230 |
| 4 | 100239319 | rs1229984 | C | T | 0.039 | 0.006 | 1.09999e-10 | 0.0001 | 41.555 | 0.180 | 0.096 | 0.060 | 0.97120 |
| 11 | 134601012 | rs12364470 | G | T | 0.019 | 0.003 | 1.10002e-12 | 0.0001 | 50.671 | 0.015 | 0.016 | 0.342 | 0.15510 |
| 9 | 11813799 | rs12375985 | A | G | -0.015 | 0.002 | 1.50003e-12 | 0.0001 | 50.014 | -0.023 | 0.015 | 0.117 | 0.33000 |
| 12 | 90213070 | rs12427047 | T | C | -0.017 | 0.002 | 1.90020e-13 | 0.0001 | 54.138 | -0.006 | 0.014 | 0.648 | 0.25550 |
| 19 | 18459377 | rs12459368 | G | A | -0.017 | 0.002 | 1.20005e-14 | 0.0001 | 59.490 | 0.004 | 0.016 | 0.785 | 0.24550 |
| 19 | 30272202 | rs12462975 | A | G | 0.019 | 0.002 | 8.49963e-20 | 0.0002 | 82.919 | -0.008 | 0.014 | 0.573 | 0.28830 |
| 6 | 51779638 | rs1266874 | G | A | 0.014 | 0.002 | 1.20005e-11 | 0.0001 | 45.946 | -0.019 | 0.013 | 0.163 | 0.37380 |
| 8 | 62054463 | rs12681792 | A | C | 0.015 | 0.003 | 4.39997e-09 | 0.0001 | 34.424 | -0.004 | 0.014 | 0.787 | 0.18690 |
| 2 | 161265910 | rs12692596 | T | C | 0.014 | 0.002 | 1.90020e-11 | 0.0001 | 45.096 | -0.009 | 0.014 | 0.506 | 0.35980 |
| 10 | 33969931 | rs12762034 | C | T | 0.028 | 0.004 | 1.29987e-13 | 0.0001 | 54.846 | 0.010 | 0.023 | 0.657 | 0.07952 |
| 14 | 91458523 | rs1286058 | A | T | 0.015 | 0.002 | 1.69981e-11 | 0.0001 | 45.311 | 0.038 | 0.015 | 0.012 | 0.73660 |
| 14 | 101146413 | rs12881629 | G | A | 0.022 | 0.004 | 8.30004e-10 | 0.0001 | 37.687 | 0.027 | 0.025 | 0.282 | 0.07256 |
| 14 | 47303114 | rs12885458 | G | T | -0.016 | 0.002 | 1.80011e-16 | 0.0001 | 67.828 | -0.027 | 0.013 | 0.043 | 0.52190 |
| 14 | 103860206 | rs12889639 | A | G | 0.016 | 0.002 | 5.79963e-14 | 0.0001 | 56.445 | -0.001 | 0.014 | 0.951 | 0.64120 |
| 18 | 63429049 | rs12956148 | A | C | 0.014 | 0.002 | 7.90005e-10 | 0.0001 | 37.787 | 0.000 | 0.014 | 0.989 | 0.32600 |
| 4 | 137083193 | rs1296328 | C | A | -0.018 | 0.002 | 2.09991e-19 | 0.0002 | 81.163 | -0.017 | 0.013 | 0.194 | 0.54870 |
| 19 | 1814025 | rs12977787 | A | G | 0.013 | 0.002 | 1.69981e-11 | 0.0001 | 45.247 | 0.003 | 0.013 | 0.827 | 0.51490 |
| 2 | 35447243 | rs13012070 | A | G | -0.014 | 0.002 | 1.79999e-09 | 0.0001 | 36.141 | -0.020 | 0.015 | 0.175 | 0.20480 |
| 3 | 44456573 | rs13076052 | G | C | 0.014 | 0.002 | 3.09999e-10 | 0.0001 | 39.589 | -0.016 | 0.014 | 0.234 | 0.29030 |
| 4 | 103188709 | rs13107325 | T | C | 0.047 | 0.004 | 5.40008e-36 | 0.0003 | 156.907 | 0.122 | 0.058 | 0.035 | 0.07952 |
| 5 | 43152216 | rs13176429 | C | T | 0.015 | 0.002 | 6.09958e-12 | 0.0001 | 47.291 | 0.013 | 0.013 | 0.331 | 0.66300 |
| 17 | 21264396 | rs1320251 | T | C | -0.018 | 0.002 | 1.50003e-18 | 0.0002 | 77.246 | 0.011 | 0.013 | 0.426 | 0.49500 |
| 6 | 120173501 | rs13218383 | G | C | -0.015 | 0.002 | 7.39946e-13 | 0.0001 | 51.438 | -0.012 | 0.014 | 0.381 | 0.35390 |
| 6 | 20488897 | rs1322842 | G | A | -0.013 | 0.002 | 1.09999e-10 | 0.0001 | 41.681 | -0.012 | 0.014 | 0.382 | 0.64910 |
| 8 | 14336834 | rs13248187 | C | T | 0.016 | 0.002 | 1.50003e-12 | 0.0001 | 50.078 | 0.002 | 0.016 | 0.907 | 0.25750 |
| 6 | 51177811 | rs1327259 | G | A | -0.015 | 0.002 | 1.50003e-13 | 0.0001 | 54.536 | -0.015 | 0.013 | 0.271 | 0.41550 |
| 9 | 15910044 | rs13292699 | C | A | -0.021 | 0.002 | 4.00037e-26 | 0.0002 | 111.786 | 0.008 | 0.013 | 0.569 | 0.45130 |
| 9 | 27760946 | rs1330199 | T | G | -0.012 | 0.002 | 2.80001e-09 | 0.0001 | 35.334 | -0.003 | 0.013 | 0.836 | 0.44330 |
| 2 | 50751414 | rs13420048 | A | C | -0.016 | 0.002 | 3.10027e-14 | 0.0001 | 57.692 | -0.001 | 0.013 | 0.929 | 0.39760 |
| 2 | 213414265 | rs13427822 | G | A | -0.018 | 0.002 | 4.70002e-16 | 0.0001 | 65.938 | 0.022 | 0.014 | 0.123 | 0.26640 |
| 4 | 65651730 | rs1346841 | A | G | -0.013 | 0.002 | 2.90001e-10 | 0.0001 | 39.728 | 0.020 | 0.014 | 0.166 | 0.42050 |
| 9 | 73796450 | rs1360201 | T | C | 0.013 | 0.002 | 4.00037e-11 | 0.0001 | 43.623 | 0.006 | 0.013 | 0.643 | 0.46420 |
| 11 | 30432220 | rs13642 | T | A | -0.016 | 0.002 | 1.80011e-15 | 0.0001 | 63.236 | -0.003 | 0.014 | 0.842 | 0.35290 |
| 15 | 73765586 | rs140159717 | T | C | -0.025 | 0.004 | 3.90032e-11 | 0.0001 | 43.668 | -0.018 | 0.022 | 0.407 | 0.06262 |
| 9 | 28425515 | rs1412239 | G | C | 0.025 | 0.002 | 6.40030e-31 | 0.0003 | 133.699 | -0.008 | 0.014 | 0.578 | 0.30420 |
| 3 | 104612668 | rs1436348 | G | A | 0.016 | 0.002 | 6.49980e-15 | 0.0001 | 60.756 | 0.017 | 0.014 | 0.221 | 0.59050 |
| 5 | 152510937 | rs1438945 | A | T | -0.014 | 0.002 | 4.90004e-10 | 0.0001 | 38.730 | 0.014 | 0.015 | 0.377 | 0.73060 |
| 13 | 79580919 | rs1441264 | A | G | 0.018 | 0.002 | 3.69999e-18 | 0.0002 | 75.500 | 0.013 | 0.014 | 0.351 | 0.62620 |
| 2 | 136407479 | rs1446585 | G | A | -0.014 | 0.002 | 9.69996e-10 | 0.0001 | 37.386 | -0.049 | 0.014 | 0.001 | 0.46120 |
| 14 | 41350367 | rs1451963 | T | G | 0.023 | 0.004 | 3.09999e-10 | 0.0001 | 39.623 | -0.024 | 0.037 | 0.525 | 0.06759 |
| 3 | 94038085 | rs1454687 | G | C | -0.021 | 0.002 | 5.50047e-26 | 0.0002 | 111.138 | 0.006 | 0.013 | 0.628 | 0.54770 |
| 12 | 41887940 | rs1458156 | T | C | 0.014 | 0.002 | 3.10027e-12 | 0.0001 | 48.643 | 0.011 | 0.013 | 0.410 | 0.44930 |
| 5 | 64070962 | rs1459190 | A | G | -0.014 | 0.002 | 6.29941e-13 | 0.0001 | 51.756 | 0.025 | 0.014 | 0.071 | 0.53480 |
| 3 | 108031094 | rs1471093 | A | G | 0.013 | 0.002 | 5.40008e-11 | 0.0001 | 43.044 | -0.004 | 0.013 | 0.739 | 0.62430 |
| 10 | 93061851 | rs147568678 | C | T | -0.014 | 0.002 | 1.40001e-09 | 0.0001 | 36.607 | 0.014 | 0.015 | 0.375 | 0.23260 |
| 5 | 87988934 | rs1477290 | C | T | 0.034 | 0.003 | 4.19952e-31 | 0.0003 | 134.500 | -0.008 | 0.020 | 0.685 | 0.15900 |
| 12 | 123024476 | rs147730268 | T | G | -0.035 | 0.004 | 9.79941e-22 | 0.0002 | 91.766 | -0.012 | 0.024 | 0.623 | 0.07058 |
| 5 | 63020706 | rs1503526 | C | T | 0.016 | 0.002 | 5.30029e-15 | 0.0001 | 61.140 | 0.014 | 0.014 | 0.294 | 0.51490 |
| 6 | 104847441 | rs156201 | C | G | 0.013 | 0.002 | 2.50000e-08 | 0.0001 | 31.057 | -0.009 | 0.014 | 0.495 | 0.73360 |
| 5 | 122657199 | rs1582931 | A | G | -0.013 | 0.002 | 1.60000e-10 | 0.0001 | 40.872 | 0.004 | 0.013 | 0.789 | 0.49800 |
| 5 | 94197477 | rs159037 | C | T | 0.013 | 0.002 | 2.59998e-08 | 0.0001 | 30.991 | -0.002 | 0.016 | 0.913 | 0.26440 |
| 8 | 77227464 | rs1609010 | G | A | 0.022 | 0.002 | 7.10068e-27 | 0.0002 | 115.193 | -0.005 | 0.013 | 0.690 | 0.55570 |
| 2 | 212292521 | rs16846140 | G | A | 0.014 | 0.002 | 6.09958e-11 | 0.0001 | 42.804 | -0.010 | 0.014 | 0.488 | 0.33900 |
| 7 | 93206264 | rs16868443 | C | G | 0.012 | 0.002 | 7.90005e-09 | 0.0001 | 33.302 | -0.033 | 0.015 | 0.028 | 0.34000 |
| 9 | 30823761 | rs16916303 | G | A | -0.020 | 0.003 | 3.09999e-10 | 0.0001 | 39.611 | -0.010 | 0.020 | 0.603 | 0.08946 |
| 17 | 39573713 | rs16966801 | G | A | 0.015 | 0.003 | 6.69993e-09 | 0.0001 | 33.614 | 0.002 | 0.021 | 0.933 | 0.19980 |
| 5 | 158271680 | rs17056301 | C | T | 0.013 | 0.002 | 8.00000e-09 | 0.0001 | 33.271 | 0.025 | 0.016 | 0.112 | 0.23960 |
| 7 | 2108036 | rs17132130 | C | G | -0.018 | 0.002 | 2.70023e-13 | 0.0001 | 53.443 | -0.010 | 0.014 | 0.472 | 0.24450 |
| 21 | 38885506 | rs17193211 | T | C | -0.025 | 0.004 | 1.09999e-09 | 0.0001 | 37.088 | 0.001 | 0.026 | 0.980 | 0.08549 |
| 4 | 140774684 | rs17289010 | G | A | -0.014 | 0.002 | 1.70000e-10 | 0.0001 | 40.736 | -0.021 | 0.015 | 0.176 | 0.31210 |
| 10 | 87490850 | rs17399739 | G | A | 0.028 | 0.004 | 1.99986e-12 | 0.0001 | 49.497 | 0.017 | 0.023 | 0.467 | 0.06561 |
| 13 | 40762556 | rs17446299 | G | C | 0.016 | 0.003 | 1.89998e-09 | 0.0001 | 36.040 | -0.013 | 0.017 | 0.455 | 0.17590 |
| 3 | 61251635 | rs17639546 | A | G | -0.023 | 0.003 | 6.70039e-17 | 0.0001 | 69.761 | 0.038 | 0.019 | 0.047 | 0.14810 |
| 1 | 156489974 | rs1778830 | A | G | 0.014 | 0.002 | 8.80035e-12 | 0.0001 | 46.571 | -0.001 | 0.015 | 0.938 | 0.35690 |
| 18 | 21090023 | rs1788808 | G | A | -0.021 | 0.002 | 8.80035e-25 | 0.0002 | 105.650 | -0.017 | 0.013 | 0.197 | 0.47420 |
| 11 | 131934926 | rs1793636 | C | G | -0.013 | 0.002 | 5.99998e-10 | 0.0001 | 38.325 | 0.019 | 0.016 | 0.239 | 0.28830 |
| 18 | 40744790 | rs1834144 | A | C | -0.014 | 0.002 | 8.10028e-12 | 0.0001 | 46.744 | 0.010 | 0.013 | 0.437 | 0.37480 |
| 14 | 73309738 | rs1860750 | A | T | 0.012 | 0.002 | 4.00000e-09 | 0.0001 | 34.628 | -0.008 | 0.013 | 0.532 | 0.53080 |
| 20 | 6612832 | rs1884897 | G | A | 0.020 | 0.002 | 1.69981e-21 | 0.0002 | 90.724 | -0.003 | 0.014 | 0.815 | 0.61430 |
| 1 | 193036952 | rs1890660 | A | G | -0.012 | 0.002 | 4.00000e-08 | 0.0001 | 30.140 | 0.022 | 0.014 | 0.113 | 0.68290 |
| 5 | 88778861 | rs1919243 | C | T | 0.012 | 0.002 | 7.10003e-09 | 0.0001 | 33.515 | 0.027 | 0.013 | 0.047 | 0.50200 |
| 9 | 81371441 | rs1999433 | T | C | -0.012 | 0.002 | 3.69999e-09 | 0.0001 | 34.769 | -0.019 | 0.013 | 0.158 | 0.44630 |
| 3 | 141298124 | rs2035936 | T | G | 0.036 | 0.004 | 9.39940e-17 | 0.0001 | 69.081 | 0.102 | 0.031 | 0.001 | 0.05268 |
| 4 | 3298800 | rs2051559 | C | T | 0.021 | 0.003 | 2.70023e-12 | 0.0001 | 48.899 | 0.032 | 0.021 | 0.131 | 0.11930 |
| 1 | 17319011 | rs2076603 | A | G | -0.012 | 0.002 | 2.80001e-09 | 0.0001 | 35.289 | -0.018 | 0.013 | 0.183 | 0.61530 |
| 4 | 52818664 | rs2102278 | G | A | 0.012 | 0.002 | 2.69998e-08 | 0.0001 | 30.888 | -0.024 | 0.014 | 0.092 | 0.28030 |
| 7 | 109228343 | rs2103123 | T | A | 0.011 | 0.002 | 4.30002e-08 | 0.0001 | 29.996 | 0.011 | 0.014 | 0.430 | 0.48310 |
| 8 | 95595162 | rs2114210 | A | G | 0.014 | 0.002 | 7.10068e-11 | 0.0001 | 42.500 | 0.004 | 0.016 | 0.809 | 0.30820 |
| 5 | 139086651 | rs2133561 | T | A | -0.014 | 0.002 | 5.90065e-12 | 0.0001 | 47.351 | 0.011 | 0.014 | 0.417 | 0.57850 |
| 7 | 26941065 | rs213518 | C | T | 0.016 | 0.003 | 8.40001e-09 | 0.0001 | 33.174 | -0.045 | 0.021 | 0.030 | 0.14310 |
| 20 | 2126089 | rs2153740 | G | A | -0.011 | 0.002 | 1.20000e-08 | 0.0001 | 32.522 | -0.014 | 0.013 | 0.302 | 0.50200 |
| 7 | 32369148 | rs215634 | G | A | -0.015 | 0.002 | 4.10015e-14 | 0.0001 | 57.097 | -0.011 | 0.015 | 0.456 | 0.63220 |
| 4 | 67813017 | rs2164300 | T | C | -0.012 | 0.002 | 8.60003e-10 | 0.0001 | 37.610 | -0.014 | 0.013 | 0.311 | 0.49200 |
| 10 | 133978962 | rs2172131 | C | T | -0.015 | 0.002 | 6.70039e-14 | 0.0001 | 56.158 | 0.003 | 0.013 | 0.822 | 0.52680 |
| 6 | 31606756 | rs2178899 | T | A | -0.026 | 0.003 | 4.79954e-18 | 0.0001 | 74.962 | 0.039 | 0.027 | 0.149 | 0.11630 |
| 7 | 39378580 | rs2190887 | T | C | -0.011 | 0.002 | 4.09996e-08 | 0.0001 | 30.118 | -0.004 | 0.013 | 0.768 | 0.56160 |
| 4 | 55505360 | rs2192158 | G | A | -0.015 | 0.002 | 1.39991e-14 | 0.0001 | 59.185 | 0.008 | 0.013 | 0.546 | 0.53080 |
| 2 | 181599070 | rs2216931 | A | C | 0.017 | 0.002 | 2.39994e-15 | 0.0001 | 62.732 | -0.003 | 0.015 | 0.837 | 0.64810 |
| 11 | 65639374 | rs2234458 | T | C | -0.021 | 0.002 | 1.10002e-23 | 0.0002 | 100.735 | -0.009 | 0.014 | 0.549 | 0.64810 |
| 6 | 108888593 | rs2253310 | G | C | 0.018 | 0.002 | 2.39994e-18 | 0.0002 | 76.355 | 0.013 | 0.013 | 0.336 | 0.56660 |
| 12 | 56494991 | rs2271189 | A | G | -0.016 | 0.002 | 2.60016e-15 | 0.0001 | 62.581 | 0.005 | 0.014 | 0.711 | 0.40360 |
| 6 | 33771673 | rs2281819 | A | T | -0.016 | 0.002 | 3.59998e-11 | 0.0001 | 43.813 | -0.003 | 0.014 | 0.843 | 0.26840 |
| 7 | 44804225 | rs2289379 | T | C | -0.015 | 0.002 | 1.80011e-13 | 0.0001 | 54.166 | 0.005 | 0.013 | 0.692 | 0.43040 |
| 17 | 34866546 | rs2306593 | T | C | -0.017 | 0.002 | 1.69981e-17 | 0.0001 | 72.430 | -0.011 | 0.013 | 0.410 | 0.51190 |
| 5 | 75003678 | rs2307111 | C | T | -0.028 | 0.002 | 1.80011e-42 | 0.0004 | 186.543 | 0.010 | 0.013 | 0.448 | 0.39560 |
| 16 | 24540806 | rs2342892 | G | T | -0.013 | 0.002 | 1.70000e-10 | 0.0001 | 40.818 | -0.017 | 0.013 | 0.199 | 0.54570 |
| 2 | 144035442 | rs2381404 | C | T | 0.014 | 0.002 | 5.49997e-09 | 0.0001 | 34.012 | -0.003 | 0.016 | 0.863 | 0.25450 |
| 7 | 113028634 | rs2396625 | A | T | -0.020 | 0.002 | 4.40048e-22 | 0.0002 | 93.344 | -0.017 | 0.014 | 0.214 | 0.40060 |
| 9 | 96430747 | rs2398861 | G | A | 0.018 | 0.002 | 1.59993e-15 | 0.0001 | 63.495 | -0.003 | 0.014 | 0.850 | 0.30620 |
| 20 | 44895075 | rs2425816 | A | G | 0.012 | 0.002 | 7.90005e-10 | 0.0001 | 37.795 | 0.019 | 0.014 | 0.175 | 0.39660 |
| 2 | 230816703 | rs2433733 | A | G | -0.018 | 0.002 | 1.00000e-16 | 0.0001 | 68.910 | 0.017 | 0.014 | 0.233 | 0.65610 |
| 9 | 94178371 | rs2482356 | C | T | -0.011 | 0.002 | 2.80001e-08 | 0.0001 | 30.823 | -0.023 | 0.014 | 0.093 | 0.43140 |
| 16 | 9412222 | rs249293 | C | G | 0.013 | 0.002 | 2.10000e-09 | 0.0001 | 35.891 | 0.016 | 0.014 | 0.278 | 0.68390 |
| 11 | 131451862 | rs2512892 | C | T | 0.013 | 0.002 | 5.50047e-11 | 0.0001 | 42.978 | -0.010 | 0.013 | 0.438 | 0.56460 |
| 5 | 77380723 | rs252761 | T | G | -0.011 | 0.002 | 2.69998e-08 | 0.0001 | 30.876 | -0.007 | 0.013 | 0.601 | 0.59940 |
| 1 | 72765116 | rs2568958 | A | G | 0.022 | 0.002 | 1.50003e-28 | 0.0002 | 122.830 | 0.026 | 0.014 | 0.065 | 0.64210 |
| 3 | 12926096 | rs2569993 | C | T | 0.013 | 0.002 | 1.20000e-09 | 0.0001 | 36.986 | -0.003 | 0.014 | 0.837 | 0.33800 |
| 3 | 183537759 | rs2606228 | C | A | -0.013 | 0.002 | 1.50000e-10 | 0.0001 | 41.059 | -0.002 | 0.014 | 0.863 | 0.67500 |
| 8 | 20632022 | rs2616143 | A | G | -0.014 | 0.002 | 1.60000e-10 | 0.0001 | 40.957 | 0.000 | 0.014 | 0.987 | 0.32800 |
| 1 | 201800511 | rs2678204 | G | T | 0.024 | 0.002 | 8.99912e-31 | 0.0003 | 133.018 | 0.017 | 0.015 | 0.250 | 0.30520 |
| 8 | 30854033 | rs2725371 | G | A | -0.016 | 0.002 | 3.50026e-13 | 0.0001 | 52.894 | -0.007 | 0.015 | 0.636 | 0.71170 |
| 6 | 131897278 | rs2781668 | T | C | 0.015 | 0.003 | 3.20000e-08 | 0.0001 | 30.570 | -0.007 | 0.016 | 0.671 | 0.17000 |
| 1 | 11207269 | rs2791643 | T | C | -0.013 | 0.002 | 2.80001e-08 | 0.0001 | 30.816 | -0.002 | 0.016 | 0.901 | 0.76040 |
| 5 | 80874229 | rs28404639 | T | C | -0.012 | 0.002 | 5.80003e-09 | 0.0001 | 33.886 | -0.015 | 0.015 | 0.323 | 0.32800 |
| 15 | 58885035 | rs28457680 | A | T | 0.018 | 0.003 | 6.59994e-10 | 0.0001 | 38.126 | -0.020 | 0.015 | 0.189 | 0.15900 |
| 22 | 41804716 | rs28489620 | A | G | -0.015 | 0.002 | 7.70016e-12 | 0.0001 | 46.831 | -0.022 | 0.015 | 0.132 | 0.28030 |
| 2 | 67837553 | rs2861685 | C | T | -0.017 | 0.002 | 4.10015e-17 | 0.0001 | 70.714 | -0.005 | 0.013 | 0.711 | 0.40360 |
| 9 | 140363045 | rs28670671 | C | T | -0.013 | 0.002 | 4.00000e-08 | 0.0001 | 30.136 | 0.037 | 0.014 | 0.010 | 0.33100 |
| 6 | 124925032 | rs2875762 | C | G | 0.015 | 0.002 | 9.60064e-11 | 0.0001 | 41.898 | -0.050 | 0.021 | 0.019 | 0.23860 |
| 3 | 12324230 | rs2920503 | T | C | -0.014 | 0.002 | 1.40001e-10 | 0.0001 | 41.126 | -0.002 | 0.014 | 0.871 | 0.28430 |
| 8 | 76544012 | rs2922757 | T | A | 0.013 | 0.002 | 4.49997e-10 | 0.0001 | 38.891 | -0.002 | 0.014 | 0.897 | 0.60140 |
| 5 | 86879056 | rs2962334 | T | G | 0.043 | 0.007 | 8.40001e-10 | 0.0001 | 37.654 | -0.041 | 0.046 | 0.371 | 0.02286 |
| 12 | 69681101 | rs317656 | A | T | -0.014 | 0.002 | 1.20000e-10 | 0.0001 | 41.515 | -0.011 | 0.014 | 0.419 | 0.68690 |
| 16 | 2089006 | rs3211995 | A | G | -0.015 | 0.003 | 1.70000e-08 | 0.0001 | 31.772 | 0.011 | 0.017 | 0.517 | 0.17300 |
| 5 | 133861663 | rs329118 | T | C | -0.017 | 0.002 | 1.00000e-16 | 0.0001 | 68.914 | -0.001 | 0.013 | 0.954 | 0.42540 |
| 11 | 133767622 | rs329651 | T | G | 0.016 | 0.003 | 4.79999e-10 | 0.0001 | 38.748 | -0.018 | 0.023 | 0.433 | 0.82010 |
| 7 | 137405295 | rs34025316 | T | C | 0.012 | 0.002 | 2.59998e-08 | 0.0001 | 30.985 | 0.001 | 0.014 | 0.956 | 0.31810 |
| 6 | 40369081 | rs34045288 | T | C | 0.024 | 0.002 | 6.90081e-29 | 0.0002 | 124.409 | 0.016 | 0.014 | 0.237 | 0.32010 |
| 15 | 41339697 | rs34153025 | C | T | -0.038 | 0.007 | 1.79999e-08 | 0.0001 | 31.687 | -0.121 | 0.039 | 0.002 | 0.01988 |
| 2 | 175166636 | rs34234296 | A | G | -0.015 | 0.002 | 2.80027e-13 | 0.0001 | 53.315 | 0.016 | 0.014 | 0.280 | 0.35390 |
| 20 | 47501038 | rs34481751 | A | C | -0.019 | 0.003 | 2.19989e-12 | 0.0001 | 49.332 | -0.012 | 0.018 | 0.503 | 0.11630 |
| 1 | 78450517 | rs34517439 | A | C | 0.038 | 0.003 | 1.29987e-35 | 0.0003 | 155.103 | 0.011 | 0.020 | 0.578 | 0.09145 |
| 5 | 119389031 | rs347551 | G | C | 0.014 | 0.002 | 8.40040e-12 | 0.0001 | 46.662 | 0.003 | 0.013 | 0.813 | 0.50000 |
| 4 | 25408838 | rs34811474 | A | G | -0.028 | 0.002 | 3.29989e-33 | 0.0003 | 144.161 | -0.030 | 0.016 | 0.063 | 0.21670 |
| 11 | 84776849 | rs349071 | A | G | -0.013 | 0.002 | 9.20026e-11 | 0.0001 | 41.980 | -0.009 | 0.014 | 0.525 | 0.51890 |
| 16 | 24862414 | rs35154326 | G | A | -0.013 | 0.002 | 2.80001e-09 | 0.0001 | 35.305 | 0.017 | 0.014 | 0.231 | 0.27240 |
| 15 | 74278126 | rs35364449 | T | C | 0.022 | 0.003 | 6.70039e-12 | 0.0001 | 47.120 | 0.059 | 0.019 | 0.002 | 0.10240 |
| 15 | 46592396 | rs355393 | G | A | -0.012 | 0.002 | 8.50002e-09 | 0.0001 | 33.164 | 0.012 | 0.014 | 0.382 | 0.49400 |
| 3 | 154034950 | rs355777 | C | G | 0.016 | 0.002 | 2.09991e-14 | 0.0001 | 58.441 | 0.018 | 0.013 | 0.185 | 0.42250 |
| 2 | 47019521 | rs35809007 | A | G | -0.017 | 0.002 | 4.10015e-17 | 0.0001 | 70.741 | 0.009 | 0.014 | 0.494 | 0.34000 |
| 17 | 79047278 | rs35867081 | G | A | -0.015 | 0.002 | 1.99986e-13 | 0.0001 | 53.979 | 0.007 | 0.013 | 0.608 | 0.50000 |
| 8 | 73440371 | rs35957544 | T | G | -0.020 | 0.002 | 1.90020e-22 | 0.0002 | 94.989 | -0.021 | 0.014 | 0.113 | 0.56960 |
| 10 | 75519691 | rs35972789 | A | C | -0.029 | 0.005 | 2.90001e-08 | 0.0001 | 30.766 | -0.061 | 0.040 | 0.128 | 0.03280 |
| 6 | 163009335 | rs36007635 | A | G | -0.021 | 0.003 | 1.20005e-12 | 0.0001 | 50.431 | 0.005 | 0.023 | 0.832 | 0.14910 |
| 8 | 38329650 | rs36061954 | T | C | 0.013 | 0.002 | 2.50000e-10 | 0.0001 | 40.053 | 0.003 | 0.014 | 0.816 | 0.38070 |
| 19 | 49649051 | rs3764625 | G | T | -0.011 | 0.002 | 1.50000e-08 | 0.0001 | 32.069 | 0.021 | 0.013 | 0.121 | 0.58550 |
| 15 | 68072458 | rs3784710 | C | T | -0.030 | 0.002 | 2.29985e-35 | 0.0003 | 153.994 | -0.004 | 0.018 | 0.800 | 0.23760 |
| 14 | 103246470 | rs3803286 | G | A | -0.018 | 0.002 | 3.10027e-18 | 0.0002 | 75.855 | -0.028 | 0.014 | 0.043 | 0.68090 |
| 7 | 50564204 | rs3807566 | T | G | -0.013 | 0.002 | 4.30002e-10 | 0.0001 | 38.980 | 0.026 | 0.014 | 0.054 | 0.40460 |
| 16 | 29994922 | rs3814883 | T | C | 0.024 | 0.002 | 2.49977e-33 | 0.0003 | 144.733 | -0.032 | 0.013 | 0.019 | 0.46020 |
| 1 | 75001480 | rs3845344 | T | C | 0.017 | 0.002 | 7.39946e-17 | 0.0001 | 69.556 | 0.018 | 0.013 | 0.177 | 0.39360 |
| 3 | 131876605 | rs3851998 | G | C | -0.014 | 0.002 | 1.20000e-09 | 0.0001 | 36.961 | -0.017 | 0.017 | 0.317 | 0.76440 |
| 1 | 6657424 | rs3866805 | A | C | 0.012 | 0.002 | 1.20000e-08 | 0.0001 | 32.426 | -0.012 | 0.014 | 0.382 | 0.36780 |
| 12 | 123492112 | rs3897102 | T | C | 0.012 | 0.002 | 1.89998e-09 | 0.0001 | 36.082 | -0.005 | 0.013 | 0.715 | 0.42740 |
| 7 | 99107727 | rs3901286 | A | C | -0.023 | 0.003 | 3.69999e-16 | 0.0001 | 66.399 | 0.001 | 0.016 | 0.970 | 0.16300 |
| 21 | 46581798 | rs394608 | C | T | 0.019 | 0.002 | 2.29985e-20 | 0.0002 | 85.478 | 0.021 | 0.013 | 0.117 | 0.54470 |
| 5 | 104022239 | rs396755 | G | C | -0.012 | 0.002 | 6.90001e-10 | 0.0001 | 38.051 | -0.040 | 0.014 | 0.003 | 0.58950 |
| 5 | 107496102 | rs40071 | C | T | -0.026 | 0.003 | 1.29987e-23 | 0.0002 | 100.276 | 0.002 | 0.016 | 0.894 | 0.19280 |
| 3 | 44028764 | rs4017425 | T | C | -0.013 | 0.002 | 1.60000e-10 | 0.0001 | 40.862 | 0.002 | 0.014 | 0.901 | 0.46920 |
| 13 | 59266053 | rs4055791 | T | C | -0.018 | 0.002 | 4.40048e-19 | 0.0002 | 79.696 | 0.015 | 0.014 | 0.253 | 0.38370 |
| 22 | 18226997 | rs406388 | G | C | 0.016 | 0.003 | 1.40001e-09 | 0.0001 | 36.700 | -0.008 | 0.016 | 0.630 | 0.16300 |
| 4 | 89054667 | rs4148155 | G | A | -0.022 | 0.003 | 7.80010e-13 | 0.0001 | 51.327 | -0.029 | 0.025 | 0.251 | 0.09245 |
| 4 | 31003636 | rs4261944 | G | T | 0.014 | 0.002 | 7.89951e-12 | 0.0001 | 46.791 | 0.007 | 0.014 | 0.615 | 0.33900 |
| 12 | 60966740 | rs4267103 | C | T | 0.016 | 0.003 | 5.39995e-10 | 0.0001 | 38.544 | 0.044 | 0.017 | 0.011 | 0.19680 |
| 15 | 31843528 | rs4284600 | C | T | 0.012 | 0.002 | 1.29999e-09 | 0.0001 | 36.838 | -0.017 | 0.013 | 0.198 | 0.46820 |
| 2 | 147903382 | rs429343 | G | A | -0.017 | 0.002 | 8.40040e-18 | 0.0001 | 73.852 | 0.002 | 0.013 | 0.863 | 0.56260 |
| 19 | 45411941 | rs429358 | C | T | -0.027 | 0.003 | 5.90065e-22 | 0.0002 | 92.753 | 0.032 | 0.017 | 0.069 | 0.15510 |
| 7 | 24354300 | rs4307239 | G | A | 0.012 | 0.002 | 4.60002e-10 | 0.0001 | 38.819 | -0.012 | 0.013 | 0.381 | 0.47510 |
| 4 | 96150044 | rs4419475 | T | A | 0.012 | 0.002 | 4.90004e-09 | 0.0001 | 34.244 | 0.011 | 0.013 | 0.402 | 0.37570 |
| 20 | 25190777 | rs4456769 | T | C | 0.015 | 0.002 | 3.80014e-12 | 0.0001 | 48.218 | 0.012 | 0.014 | 0.383 | 0.34490 |
| 13 | 54104968 | rs4477562 | T | C | 0.030 | 0.003 | 3.69999e-23 | 0.0002 | 98.254 | -0.011 | 0.019 | 0.559 | 0.11030 |
| 2 | 205375909 | rs4482463 | A | C | -0.031 | 0.004 | 1.39991e-16 | 0.0001 | 68.307 | 0.031 | 0.028 | 0.266 | 0.91950 |
| 16 | 19942527 | rs4613074 | C | T | -0.024 | 0.003 | 3.69999e-20 | 0.0002 | 84.556 | -0.025 | 0.018 | 0.152 | 0.18290 |
| 2 | 236762851 | rs4625852 | A | G | -0.014 | 0.002 | 2.00000e-08 | 0.0001 | 31.477 | -0.001 | 0.016 | 0.970 | 0.19580 |
| 1 | 2723214 | rs4648450 | A | C | -0.015 | 0.002 | 1.29987e-13 | 0.0001 | 54.839 | -0.007 | 0.013 | 0.598 | 0.47610 |
| 1 | 243832560 | rs4658403 | T | C | -0.019 | 0.003 | 2.70023e-12 | 0.0001 | 48.881 | 0.008 | 0.017 | 0.633 | 0.82210 |
| 2 | 60217457 | rs4672338 | T | C | 0.013 | 0.002 | 2.39999e-10 | 0.0001 | 40.095 | 0.011 | 0.013 | 0.418 | 0.33200 |
| 7 | 70038969 | rs4718964 | T | G | 0.011 | 0.002 | 2.59998e-08 | 0.0001 | 30.998 | -0.020 | 0.014 | 0.139 | 0.40660 |
| 7 | 3125220 | rs4722398 | T | C | 0.019 | 0.003 | 1.40001e-10 | 0.0001 | 41.201 | 0.005 | 0.026 | 0.838 | 0.13420 |
| 8 | 64756657 | rs4737188 | T | A | -0.013 | 0.002 | 7.39946e-11 | 0.0001 | 42.418 | -0.003 | 0.013 | 0.820 | 0.48710 |
| 11 | 13331226 | rs4757144 | A | G | 0.016 | 0.002 | 1.50003e-15 | 0.0001 | 63.613 | 0.023 | 0.013 | 0.083 | 0.58750 |
| 12 | 103658096 | rs4764949 | G | A | -0.018 | 0.002 | 3.10027e-17 | 0.0001 | 71.292 | 0.009 | 0.014 | 0.509 | 0.36080 |
| 17 | 1824305 | rs4790292 | A | C | -0.026 | 0.003 | 6.90081e-21 | 0.0002 | 87.891 | -0.001 | 0.018 | 0.945 | 0.14410 |
| 2 | 86764004 | rs4832298 | T | C | -0.016 | 0.002 | 8.19974e-14 | 0.0001 | 55.750 | 0.013 | 0.014 | 0.368 | 0.66700 |
| 8 | 116671848 | rs4876611 | G | A | 0.019 | 0.002 | 3.50026e-18 | 0.0002 | 75.564 | 0.016 | 0.014 | 0.262 | 0.76140 |
| 6 | 126052729 | rs4895799 | T | C | 0.012 | 0.002 | 1.09999e-08 | 0.0001 | 32.620 | -0.018 | 0.013 | 0.180 | 0.57360 |
| 10 | 100043147 | rs4919197 | T | C | 0.011 | 0.002 | 4.79999e-08 | 0.0001 | 29.791 | -0.005 | 0.014 | 0.704 | 0.46220 |
| 11 | 8639200 | rs4929923 | C | T | 0.019 | 0.002 | 3.40017e-20 | 0.0002 | 84.728 | 0.005 | 0.014 | 0.711 | 0.62330 |
| 16 | 69187318 | rs5011579 | G | C | 0.014 | 0.002 | 1.29999e-10 | 0.0001 | 41.245 | -0.012 | 0.014 | 0.403 | 0.69580 |
| 18 | 7548501 | rs512121 | C | T | -0.016 | 0.003 | 6.69993e-10 | 0.0001 | 38.100 | -0.004 | 0.019 | 0.818 | 0.20680 |
| 3 | 173114305 | rs529200 | G | A | 0.017 | 0.002 | 5.79963e-18 | 0.0001 | 74.579 | 0.016 | 0.013 | 0.246 | 0.52190 |
| 1 | 177889025 | rs539515 | C | A | 0.050 | 0.002 | 7.00003e-91 | 0.0008 | 408.701 | -0.009 | 0.017 | 0.607 | 0.18390 |
| 2 | 219284215 | rs55658481 | A | G | 0.013 | 0.002 | 1.79999e-10 | 0.0001 | 40.632 | -0.004 | 0.014 | 0.790 | 0.32900 |
| 19 | 18207397 | rs55714539 | C | A | 0.018 | 0.002 | 3.50026e-17 | 0.0001 | 71.050 | -0.028 | 0.014 | 0.038 | 0.34790 |
| 12 | 991306 | rs55726687 | A | G | 0.025 | 0.002 | 2.70023e-24 | 0.0002 | 103.395 | -0.021 | 0.016 | 0.198 | 0.18790 |
| 20 | 62567684 | rs55886426 | G | C | -0.028 | 0.005 | 1.89998e-09 | 0.0001 | 36.101 | -0.028 | 0.033 | 0.398 | 0.03976 |
| 11 | 28712741 | rs558887 | G | A | -0.013 | 0.002 | 8.00000e-10 | 0.0001 | 37.753 | 0.004 | 0.016 | 0.787 | 0.30120 |
| 4 | 171609715 | rs55920177 | T | A | -0.018 | 0.003 | 8.70001e-10 | 0.0001 | 37.607 | -0.016 | 0.026 | 0.536 | 0.10830 |
| 18 | 39644247 | rs559231 | T | G | 0.013 | 0.002 | 3.20000e-10 | 0.0001 | 39.529 | -0.016 | 0.013 | 0.226 | 0.37570 |
| 12 | 97584357 | rs55966114 | T | C | 0.015 | 0.003 | 3.79997e-09 | 0.0001 | 34.706 | 0.013 | 0.017 | 0.435 | 0.19580 |
| 3 | 69925128 | rs56038322 | A | G | 0.014 | 0.002 | 6.79986e-11 | 0.0001 | 42.588 | 0.028 | 0.014 | 0.050 | 0.31210 |
| 16 | 53806453 | rs56094641 | G | A | 0.073 | 0.002 | 1.00000e-200 | 0.0026 | 1306.497 | 0.008 | 0.013 | 0.527 | 0.43440 |
| 2 | 172818467 | rs56133507 | G | T | 0.014 | 0.002 | 1.40001e-08 | 0.0001 | 32.252 | -0.010 | 0.020 | 0.613 | 0.17890 |
| 17 | 46288649 | rs56161855 | T | A | 0.023 | 0.003 | 1.10002e-14 | 0.0001 | 59.777 | -0.006 | 0.018 | 0.733 | 0.15710 |
| 9 | 131040874 | rs56203622 | C | T | 0.018 | 0.003 | 2.00000e-10 | 0.0001 | 40.461 | -0.015 | 0.017 | 0.381 | 0.16600 |
| 19 | 19352155 | rs56352336 | C | T | -0.017 | 0.003 | 1.79999e-09 | 0.0001 | 36.217 | -0.014 | 0.020 | 0.502 | 0.16100 |
| 19 | 4064057 | rs56356382 | C | T | -0.023 | 0.003 | 6.70039e-19 | 0.0002 | 78.861 | -0.008 | 0.018 | 0.640 | 0.19180 |
| 13 | 33381721 | rs56399737 | T | C | -0.016 | 0.002 | 3.59998e-15 | 0.0001 | 61.921 | -0.005 | 0.013 | 0.729 | 0.48110 |
| 15 | 99222509 | rs56803094 | G | A | -0.015 | 0.002 | 5.00000e-10 | 0.0001 | 38.673 | 0.021 | 0.015 | 0.147 | 0.20180 |
| 13 | 86511730 | rs56858768 | A | G | 0.016 | 0.002 | 5.60015e-13 | 0.0001 | 51.995 | -0.001 | 0.014 | 0.949 | 0.30020 |
| 8 | 25662655 | rs56893062 | G | T | 0.013 | 0.002 | 2.39999e-09 | 0.0001 | 35.582 | 0.012 | 0.014 | 0.376 | 0.28830 |
| 15 | 79403002 | rs57488047 | C | T | -0.015 | 0.002 | 4.49987e-14 | 0.0001 | 56.948 | -0.009 | 0.013 | 0.486 | 0.44140 |
| 18 | 58048295 | rs57636386 | C | T | -0.041 | 0.004 | 1.00000e-29 | 0.0003 | 128.189 | 0.005 | 0.030 | 0.858 | 0.07455 |
| 10 | 99769388 | rs577525 | C | T | 0.019 | 0.002 | 3.69999e-22 | 0.0002 | 93.659 | 0.002 | 0.013 | 0.866 | 0.55860 |
| 6 | 100629078 | rs57989773 | C | T | 0.013 | 0.002 | 1.60000e-08 | 0.0001 | 31.945 | 0.014 | 0.017 | 0.398 | 0.24350 |
| 7 | 75081418 | rs58862095 | T | C | -0.023 | 0.002 | 4.90004e-29 | 0.0002 | 125.070 | -0.022 | 0.013 | 0.105 | 0.44430 |
| 2 | 25145173 | rs59086897 | A | T | 0.033 | 0.002 | 6.59933e-64 | 0.0006 | 284.857 | -0.004 | 0.013 | 0.768 | 0.46620 |
| 11 | 43692423 | rs59227842 | G | A | 0.023 | 0.002 | 1.90020e-26 | 0.0002 | 113.210 | 0.006 | 0.014 | 0.684 | 0.34890 |
| 17 | 31479035 | rs59237168 | C | T | -0.016 | 0.002 | 8.19974e-11 | 0.0001 | 42.215 | 0.016 | 0.015 | 0.298 | 0.22960 |
| 22 | 40697377 | rs5995843 | G | A | -0.018 | 0.002 | 5.00035e-17 | 0.0001 | 70.329 | 0.014 | 0.014 | 0.300 | 0.32210 |
| 20 | 53470583 | rs6023649 | G | A | -0.014 | 0.002 | 1.50000e-09 | 0.0001 | 36.517 | -0.024 | 0.016 | 0.132 | 0.75350 |
| 18 | 1839911 | rs60764613 | T | G | 0.020 | 0.003 | 1.59993e-12 | 0.0001 | 49.965 | -0.007 | 0.018 | 0.690 | 0.14120 |
| 20 | 1409757 | rs6134916 | T | C | -0.011 | 0.002 | 4.60002e-08 | 0.0001 | 29.886 | 0.000 | 0.013 | 0.977 | 0.49110 |
| 1 | 156049877 | rs61813324 | T | C | 0.029 | 0.003 | 6.20012e-23 | 0.0002 | 97.221 | 0.003 | 0.018 | 0.870 | 0.13020 |
| 1 | 174768522 | rs61828088 | A | G | 0.022 | 0.003 | 2.90001e-12 | 0.0001 | 48.761 | 0.007 | 0.024 | 0.760 | 0.10830 |
| 11 | 89922417 | rs61903695 | G | A | 0.017 | 0.002 | 2.70023e-13 | 0.0001 | 53.438 | -0.011 | 0.015 | 0.473 | 0.26540 |
| 14 | 101531854 | rs61992671 | G | A | -0.016 | 0.002 | 1.20005e-14 | 0.0001 | 59.475 | 0.042 | 0.013 | 0.002 | 0.49200 |
| 2 | 422144 | rs62107261 | C | T | -0.092 | 0.005 | 1.69981e-86 | 0.0008 | 388.514 | -0.004 | 0.048 | 0.932 | 0.04473 |
| 2 | 166190881 | rs62176243 | T | A | -0.015 | 0.002 | 6.20012e-11 | 0.0001 | 42.748 | -0.023 | 0.015 | 0.110 | 0.25050 |
| 2 | 157606799 | rs62176993 | A | G | 0.011 | 0.002 | 3.40001e-08 | 0.0001 | 30.486 | -0.007 | 0.013 | 0.628 | 0.37770 |
| 2 | 182566998 | rs62190049 | C | G | -0.011 | 0.002 | 3.69999e-08 | 0.0001 | 30.293 | -0.020 | 0.014 | 0.146 | 0.41050 |
| 3 | 20466465 | rs62241847 | G | A | -0.012 | 0.002 | 5.80003e-09 | 0.0001 | 33.887 | -0.001 | 0.014 | 0.918 | 0.31610 |
| 3 | 9498143 | rs62246311 | A | G | 0.021 | 0.003 | 2.90001e-10 | 0.0001 | 39.735 | -0.076 | 0.028 | 0.006 | 0.08847 |
| 5 | 105870033 | rs62379271 | G | T | 0.012 | 0.002 | 7.29995e-09 | 0.0001 | 33.461 | -0.034 | 0.013 | 0.010 | 0.56360 |
| 11 | 27679916 | rs6265 | T | C | -0.040 | 0.003 | 5.90065e-56 | 0.0005 | 248.356 | 0.040 | 0.018 | 0.031 | 0.19680 |
| 3 | 88263487 | rs6419869 | G | T | 0.022 | 0.003 | 1.99986e-12 | 0.0001 | 49.502 | 0.008 | 0.022 | 0.712 | 0.89260 |
| 2 | 145627927 | rs6430068 | A | G | 0.019 | 0.003 | 5.60003e-09 | 0.0001 | 33.980 | 0.025 | 0.022 | 0.259 | 0.11330 |
| 3 | 170602073 | rs6444950 | A | G | 0.016 | 0.002 | 3.90032e-12 | 0.0001 | 48.177 | -0.007 | 0.016 | 0.669 | 0.21870 |
| 4 | 38556399 | rs6531639 | A | G | -0.014 | 0.002 | 2.99999e-09 | 0.0001 | 35.216 | 0.016 | 0.015 | 0.308 | 0.23260 |
| 2 | 59307725 | rs6545714 | A | G | -0.021 | 0.002 | 2.49977e-24 | 0.0002 | 103.604 | -0.009 | 0.014 | 0.499 | 0.61830 |
| 12 | 133414054 | rs6560906 | C | T | -0.012 | 0.002 | 1.70000e-08 | 0.0001 | 31.770 | 0.007 | 0.015 | 0.629 | 0.71970 |
| 13 | 58257667 | rs6561937 | A | T | -0.016 | 0.002 | 6.59933e-12 | 0.0001 | 47.130 | -0.016 | 0.016 | 0.335 | 0.79620 |
| 18 | 57829135 | rs6567160 | C | T | 0.054 | 0.002 | 2.50035e-114 | 0.0010 | 516.422 | -0.022 | 0.017 | 0.187 | 0.23960 |
| 14 | 94023972 | rs6575340 | A | G | 0.021 | 0.002 | 9.09913e-24 | 0.0002 | 101.023 | 0.005 | 0.014 | 0.734 | 0.62330 |
| 4 | 18351898 | rs66679256 | T | C | 0.015 | 0.002 | 1.90020e-14 | 0.0001 | 58.596 | -0.003 | 0.014 | 0.821 | 0.44730 |
| 1 | 47678458 | rs6669341 | G | A | -0.017 | 0.002 | 9.20026e-18 | 0.0001 | 73.678 | -0.023 | 0.014 | 0.096 | 0.59840 |
| 1 | 33784146 | rs6682438 | C | T | 0.013 | 0.002 | 1.79999e-09 | 0.0001 | 36.147 | 0.035 | 0.014 | 0.012 | 0.63120 |
| 1 | 80812329 | rs6688826 | C | T | 0.014 | 0.002 | 7.70016e-11 | 0.0001 | 42.324 | -0.013 | 0.017 | 0.439 | 0.30220 |
| 2 | 100123030 | rs6707827 | G | A | 0.012 | 0.002 | 1.70000e-08 | 0.0001 | 31.756 | -0.004 | 0.015 | 0.815 | 0.71070 |
| 2 | 239597 | rs6710091 | G | C | -0.012 | 0.002 | 2.69998e-08 | 0.0001 | 30.889 | 0.005 | 0.014 | 0.705 | 0.32900 |
| 2 | 40291940 | rs6713781 | C | G | -0.013 | 0.002 | 1.40001e-10 | 0.0001 | 41.200 | -0.009 | 0.014 | 0.539 | 0.38070 |
| 2 | 628504 | rs6744646 | G | A | 0.055 | 0.003 | 1.69981e-98 | 0.0009 | 443.708 | 0.018 | 0.018 | 0.317 | 0.82900 |
| 2 | 81741750 | rs6752979 | A | G | 0.012 | 0.002 | 1.09999e-08 | 0.0001 | 32.586 | 0.023 | 0.014 | 0.107 | 0.28930 |
| 10 | 126640936 | rs67609008 | C | T | 0.018 | 0.002 | 2.80027e-15 | 0.0001 | 62.389 | -0.013 | 0.017 | 0.434 | 0.26040 |
| 3 | 196116393 | rs6774894 | A | T | 0.013 | 0.002 | 4.30002e-10 | 0.0001 | 38.991 | -0.020 | 0.014 | 0.176 | 0.36380 |
| 3 | 62376645 | rs6777784 | T | G | 0.012 | 0.002 | 1.29999e-09 | 0.0001 | 36.763 | 0.001 | 0.014 | 0.919 | 0.62230 |
| 4 | 162132758 | rs6843852 | T | C | 0.013 | 0.002 | 1.69981e-11 | 0.0001 | 45.312 | 0.020 | 0.013 | 0.134 | 0.52090 |
| 6 | 97753952 | rs6909685 | T | C | -0.015 | 0.002 | 6.20012e-12 | 0.0001 | 47.275 | -0.020 | 0.014 | 0.156 | 0.32500 |
| 6 | 142703483 | rs6922607 | G | A | 0.014 | 0.003 | 1.50000e-08 | 0.0001 | 32.071 | 0.040 | 0.018 | 0.023 | 0.22470 |
| 6 | 98421721 | rs6938973 | C | T | 0.018 | 0.002 | 1.90020e-19 | 0.0002 | 81.384 | 0.010 | 0.014 | 0.502 | 0.59150 |
| 7 | 1270699 | rs6950388 | A | G | 0.016 | 0.002 | 9.20026e-11 | 0.0001 | 41.990 | 0.002 | 0.016 | 0.875 | 0.78030 |
| 5 | 3513485 | rs698147 | G | A | -0.013 | 0.002 | 2.90001e-11 | 0.0001 | 44.225 | -0.015 | 0.013 | 0.250 | 0.50300 |
| 9 | 109072075 | rs7024334 | G | T | -0.014 | 0.002 | 1.70000e-08 | 0.0001 | 31.793 | -0.006 | 0.016 | 0.712 | 0.77340 |
| 9 | 129408290 | rs7027304 | T | C | 0.015 | 0.002 | 1.69981e-12 | 0.0001 | 49.753 | -0.008 | 0.015 | 0.585 | 0.64910 |
| 9 | 37081301 | rs7034554 | G | A | -0.014 | 0.002 | 4.70002e-11 | 0.0001 | 43.294 | -0.016 | 0.015 | 0.275 | 0.36380 |
| 9 | 120377178 | rs7038943 | C | T | -0.014 | 0.002 | 1.50003e-11 | 0.0001 | 45.587 | -0.023 | 0.014 | 0.084 | 0.35880 |
| 9 | 6959840 | rs7042372 | G | A | -0.013 | 0.002 | 2.69998e-09 | 0.0001 | 35.379 | 0.011 | 0.014 | 0.426 | 0.33400 |
| 10 | 125226178 | rs705145 | A | C | 0.014 | 0.002 | 1.59993e-11 | 0.0001 | 45.398 | 0.012 | 0.014 | 0.398 | 0.34190 |
| 10 | 61842645 | rs7070670 | T | C | -0.013 | 0.002 | 2.69998e-09 | 0.0001 | 35.415 | 0.020 | 0.015 | 0.178 | 0.31910 |
| 11 | 47529947 | rs7124681 | A | C | 0.026 | 0.002 | 1.39991e-37 | 0.0003 | 164.137 | 0.000 | 0.014 | 0.976 | 0.42150 |
| 12 | 50263148 | rs7132908 | A | G | 0.029 | 0.002 | 1.10002e-46 | 0.0004 | 205.857 | -0.022 | 0.014 | 0.102 | 0.35690 |
| 14 | 102780553 | rs7159965 | G | C | 0.015 | 0.003 | 3.59998e-08 | 0.0001 | 30.374 | -0.002 | 0.017 | 0.899 | 0.16600 |
| 15 | 95272920 | rs7169847 | T | G | -0.014 | 0.002 | 1.99986e-11 | 0.0001 | 44.930 | -0.002 | 0.013 | 0.864 | 0.61930 |
| 16 | 407723 | rs7201895 | A | G | -0.015 | 0.002 | 8.00018e-13 | 0.0001 | 51.284 | 0.004 | 0.015 | 0.804 | 0.34100 |
| 16 | 82872628 | rs7206608 | G | C | 0.013 | 0.002 | 2.59998e-10 | 0.0001 | 39.935 | 0.011 | 0.014 | 0.415 | 0.31310 |
| 17 | 65832016 | rs7218014 | C | T | 0.019 | 0.003 | 7.80010e-14 | 0.0001 | 55.849 | 0.051 | 0.015 | 0.001 | 0.21870 |
| 18 | 31251221 | rs7232171 | T | G | 0.013 | 0.002 | 1.20000e-10 | 0.0001 | 41.464 | 0.002 | 0.014 | 0.891 | 0.57850 |
| 12 | 2161561 | rs723672 | T | C | 0.011 | 0.002 | 4.00000e-08 | 0.0001 | 30.172 | 0.002 | 0.013 | 0.855 | 0.46320 |
| 19 | 33937277 | rs7250833 | T | C | 0.014 | 0.002 | 7.10003e-10 | 0.0001 | 37.984 | 0.018 | 0.017 | 0.287 | 0.27440 |
| 19 | 47562509 | rs7259070 | C | T | 0.022 | 0.002 | 3.80014e-26 | 0.0002 | 111.891 | 0.005 | 0.013 | 0.715 | 0.58650 |
| 2 | 229019109 | rs72617140 | C | A | 0.019 | 0.002 | 1.10002e-14 | 0.0001 | 59.625 | -0.023 | 0.017 | 0.168 | 0.20970 |
| 1 | 1601052 | rs72634826 | A | G | -0.021 | 0.002 | 2.09991e-19 | 0.0002 | 81.147 | 0.000 | 0.016 | 0.991 | 0.25250 |
| 6 | 50816887 | rs72892910 | T | G | 0.039 | 0.003 | 6.59933e-49 | 0.0004 | 216.041 | 0.013 | 0.016 | 0.437 | 0.17300 |
| 6 | 69761994 | rs72910629 | G | A | 0.016 | 0.003 | 2.50000e-08 | 0.0001 | 31.026 | 0.000 | 0.020 | 0.984 | 0.12230 |
| 6 | 73910108 | rs72948836 | G | A | -0.024 | 0.004 | 7.00003e-09 | 0.0001 | 33.549 | -0.031 | 0.039 | 0.429 | 0.05765 |
| 3 | 185828465 | rs73052033 | C | T | -0.030 | 0.003 | 1.00000e-31 | 0.0003 | 137.277 | 0.006 | 0.018 | 0.744 | 0.18590 |
| 20 | 51195932 | rs73142879 | T | C | -0.027 | 0.003 | 9.30037e-27 | 0.0002 | 114.674 | -0.016 | 0.018 | 0.367 | 0.19280 |
| 12 | 108294381 | rs73193736 | G | A | -0.018 | 0.002 | 3.59998e-15 | 0.0001 | 61.917 | -0.040 | 0.015 | 0.008 | 0.25750 |
| 4 | 28489339 | rs73213484 | T | A | -0.023 | 0.003 | 1.00000e-15 | 0.0001 | 64.432 | -0.011 | 0.019 | 0.564 | 0.18490 |
| 13 | 99236471 | rs7331420 | A | G | -0.014 | 0.002 | 1.60000e-10 | 0.0001 | 40.854 | -0.028 | 0.015 | 0.054 | 0.29520 |
| 9 | 92207308 | rs7357754 | G | A | 0.014 | 0.002 | 5.79963e-13 | 0.0001 | 51.912 | 0.009 | 0.013 | 0.503 | 0.51590 |
| 10 | 18549889 | rs73601548 | T | C | 0.017 | 0.003 | 4.90004e-08 | 0.0001 | 29.763 | 0.048 | 0.027 | 0.076 | 0.08052 |
| 4 | 80718632 | rs7442137 | T | C | -0.012 | 0.002 | 1.60000e-09 | 0.0001 | 36.442 | 0.007 | 0.014 | 0.642 | 0.62430 |
| 5 | 87682877 | rs7442885 | G | C | -0.023 | 0.002 | 5.19996e-21 | 0.0002 | 88.454 | -0.025 | 0.017 | 0.145 | 0.23560 |
| 2 | 105460333 | rs745249 | T | C | 0.018 | 0.002 | 3.29989e-16 | 0.0001 | 66.597 | 0.010 | 0.014 | 0.494 | 0.26840 |
| 15 | 92573639 | rs7498044 | A | G | -0.017 | 0.002 | 4.70002e-12 | 0.0001 | 47.808 | -0.017 | 0.015 | 0.267 | 0.21370 |
| 1 | 174973726 | rs75035127 | G | A | -0.041 | 0.006 | 1.20005e-12 | 0.0001 | 50.435 | -0.025 | 0.059 | 0.671 | 0.03479 |
| 1 | 210301331 | rs7516554 | T | C | 0.012 | 0.002 | 1.50000e-09 | 0.0001 | 36.499 | -0.033 | 0.014 | 0.015 | 0.42350 |
| 1 | 66434743 | rs7519259 | A | G | 0.014 | 0.002 | 5.79963e-12 | 0.0001 | 47.394 | -0.008 | 0.013 | 0.536 | 0.52980 |
| 1 | 209208033 | rs7539903 | A | T | -0.014 | 0.002 | 1.20005e-11 | 0.0001 | 46.030 | 0.001 | 0.013 | 0.962 | 0.61030 |
| 3 | 42305131 | rs754635 | G | C | 0.023 | 0.003 | 5.19996e-13 | 0.0001 | 52.139 | -0.016 | 0.022 | 0.484 | 0.89560 |
| 1 | 115252609 | rs7549358 | C | G | -0.011 | 0.002 | 3.29997e-08 | 0.0001 | 30.508 | 0.011 | 0.014 | 0.435 | 0.67000 |
| 6 | 26145217 | rs75499503 | T | C | -0.018 | 0.002 | 3.10027e-13 | 0.0001 | 53.160 | -0.013 | 0.016 | 0.404 | 0.21370 |
| 3 | 84321469 | rs75557510 | G | A | -0.031 | 0.004 | 1.59993e-13 | 0.0001 | 54.398 | 0.004 | 0.031 | 0.904 | 0.05169 |
| 2 | 6169351 | rs7571496 | G | A | -0.015 | 0.002 | 1.10002e-11 | 0.0001 | 46.223 | 0.014 | 0.014 | 0.293 | 0.28830 |
| 3 | 114371939 | rs76183894 | C | T | -0.022 | 0.004 | 2.69998e-09 | 0.0001 | 35.397 | 0.031 | 0.027 | 0.266 | 0.07356 |
| 3 | 25110415 | rs7619139 | A | T | 0.013 | 0.002 | 1.29999e-10 | 0.0001 | 41.338 | 0.014 | 0.013 | 0.293 | 0.56960 |
| 8 | 85685773 | rs76387394 | G | A | 0.027 | 0.004 | 1.00000e-09 | 0.0001 | 37.284 | 0.044 | 0.034 | 0.200 | 0.05268 |
| 6 | 143185557 | rs765874 | A | T | -0.012 | 0.002 | 3.59998e-09 | 0.0001 | 34.842 | 0.008 | 0.013 | 0.569 | 0.49800 |
| 1 | 195148296 | rs76702514 | G | C | -0.017 | 0.002 | 1.10002e-11 | 0.0001 | 46.189 | 0.011 | 0.016 | 0.473 | 0.23160 |
| 5 | 112448114 | rs7704382 | G | C | 0.012 | 0.002 | 1.29999e-09 | 0.0001 | 36.769 | -0.005 | 0.013 | 0.684 | 0.41250 |
| 6 | 153380228 | rs7762794 | G | A | 0.015 | 0.002 | 1.99986e-11 | 0.0001 | 44.946 | -0.010 | 0.014 | 0.483 | 0.29520 |
| 17 | 4801163 | rs7774 | A | C | 0.015 | 0.002 | 1.50003e-12 | 0.0001 | 50.068 | 0.003 | 0.014 | 0.849 | 0.35790 |
| 7 | 78121458 | rs7805441 | T | C | 0.013 | 0.002 | 2.60016e-11 | 0.0001 | 44.494 | 0.023 | 0.013 | 0.083 | 0.53180 |
| 12 | 24024639 | rs78086698 | C | T | 0.032 | 0.005 | 2.30001e-10 | 0.0001 | 40.173 | 0.047 | 0.048 | 0.327 | 0.03678 |
| 8 | 132875320 | rs7828631 | T | C | 0.018 | 0.003 | 1.20000e-08 | 0.0001 | 32.466 | 0.013 | 0.028 | 0.633 | 0.10140 |
| 18 | 53397199 | rs784257 | C | T | 0.018 | 0.003 | 2.39994e-12 | 0.0001 | 49.105 | -0.011 | 0.018 | 0.535 | 0.81310 |
| 9 | 103121547 | rs7852189 | G | A | 0.017 | 0.002 | 1.10002e-15 | 0.0001 | 64.252 | 0.006 | 0.014 | 0.674 | 0.32600 |
| 10 | 16750129 | rs7893571 | T | G | 0.014 | 0.002 | 9.39940e-12 | 0.0001 | 46.458 | 0.019 | 0.014 | 0.196 | 0.67400 |
| 10 | 102470432 | rs7916385 | T | C | -0.024 | 0.003 | 1.80011e-15 | 0.0001 | 63.245 | -0.004 | 0.019 | 0.856 | 0.15810 |
| 15 | 53402758 | rs79212998 | G | T | -0.023 | 0.004 | 2.80001e-09 | 0.0001 | 35.286 | -0.034 | 0.027 | 0.204 | 0.07157 |
| 10 | 65191645 | rs7924036 | T | G | -0.014 | 0.002 | 3.50026e-13 | 0.0001 | 52.890 | -0.008 | 0.013 | 0.566 | 0.50800 |
| 11 | 118941596 | rs7925100 | A | G | 0.014 | 0.002 | 1.10002e-12 | 0.0001 | 50.578 | -0.013 | 0.015 | 0.359 | 0.34890 |
| 11 | 130795698 | rs7944782 | G | T | 0.016 | 0.002 | 1.39991e-15 | 0.0001 | 63.732 | -0.011 | 0.013 | 0.410 | 0.51490 |
| 11 | 64090422 | rs7947143 | A | G | -0.018 | 0.003 | 3.59998e-11 | 0.0001 | 43.794 | 0.017 | 0.018 | 0.353 | 0.16200 |
| 11 | 892089 | rs7952102 | C | T | -0.014 | 0.002 | 2.80027e-12 | 0.0001 | 48.813 | 0.007 | 0.014 | 0.616 | 0.36780 |
| 10 | 104952499 | rs79780963 | T | C | 0.024 | 0.004 | 2.50000e-10 | 0.0001 | 40.017 | -0.028 | 0.024 | 0.249 | 0.96620 |
| 13 | 97019090 | rs7996639 | A | G | 0.014 | 0.002 | 1.20005e-12 | 0.0001 | 50.537 | 0.031 | 0.013 | 0.021 | 0.49600 |
| 14 | 25930988 | rs8015400 | A | C | 0.021 | 0.002 | 9.60064e-24 | 0.0002 | 100.913 | 0.005 | 0.014 | 0.715 | 0.65210 |
| 14 | 79937216 | rs8020365 | A | T | 0.025 | 0.002 | 6.70039e-25 | 0.0002 | 106.204 | -0.014 | 0.015 | 0.367 | 0.20970 |
| 15 | 35837297 | rs8024137 | T | A | 0.016 | 0.003 | 2.39999e-08 | 0.0001 | 31.142 | -0.023 | 0.027 | 0.391 | 0.89170 |
| 17 | 15888448 | rs8076669 | C | T | 0.014 | 0.002 | 5.90065e-12 | 0.0001 | 47.368 | 0.020 | 0.014 | 0.144 | 0.56560 |
| 21 | 40644170 | rs8134638 | C | T | 0.012 | 0.002 | 1.89998e-09 | 0.0001 | 36.092 | 0.001 | 0.013 | 0.957 | 0.41250 |
| 1 | 190294726 | rs815163 | C | T | -0.017 | 0.002 | 4.60045e-17 | 0.0001 | 70.494 | -0.003 | 0.013 | 0.808 | 0.57160 |
| 16 | 69651866 | rs862320 | T | C | -0.023 | 0.002 | 2.19989e-30 | 0.0003 | 131.227 | 0.037 | 0.013 | 0.006 | 0.42150 |
| 16 | 4015729 | rs879620 | T | C | 0.024 | 0.002 | 5.70033e-32 | 0.0003 | 138.483 | -0.002 | 0.013 | 0.905 | 0.61130 |
| 20 | 41982698 | rs909892 | A | G | -0.018 | 0.003 | 8.60003e-10 | 0.0001 | 37.611 | 0.009 | 0.022 | 0.684 | 0.15010 |
| 6 | 31880480 | rs9267671 | A | G | 0.026 | 0.004 | 5.30005e-10 | 0.0001 | 38.554 | 0.061 | 0.046 | 0.184 | 0.07256 |
| 6 | 83433228 | rs9294260 | A | G | 0.015 | 0.002 | 1.99986e-13 | 0.0001 | 53.960 | 0.009 | 0.013 | 0.480 | 0.46420 |
| 6 | 42524937 | rs9296389 | C | G | 0.012 | 0.002 | 1.09999e-08 | 0.0001 | 32.594 | 0.011 | 0.013 | 0.424 | 0.40460 |
| 2 | 26949366 | rs935166 | A | G | -0.016 | 0.002 | 5.90065e-16 | 0.0001 | 65.475 | -0.029 | 0.013 | 0.035 | 0.48110 |
| 6 | 34688946 | rs9366863 | C | T | -0.028 | 0.002 | 2.60016e-40 | 0.0003 | 176.685 | -0.003 | 0.014 | 0.809 | 0.66300 |
| 20 | 17171373 | rs947088 | T | G | 0.013 | 0.002 | 9.80009e-09 | 0.0001 | 32.882 | -0.008 | 0.014 | 0.557 | 0.72470 |
| 6 | 154333183 | rs9478496 | C | T | 0.018 | 0.003 | 6.59933e-11 | 0.0001 | 42.630 | 0.008 | 0.017 | 0.647 | 0.16500 |
| 13 | 111926794 | rs9522173 | T | A | -0.012 | 0.002 | 1.29999e-09 | 0.0001 | 36.832 | 0.001 | 0.014 | 0.921 | 0.39360 |
| 13 | 67472713 | rs9571687 | A | C | -0.013 | 0.002 | 4.60002e-10 | 0.0001 | 38.858 | -0.006 | 0.016 | 0.684 | 0.31610 |
| 12 | 107713511 | rs961498 | C | G | 0.012 | 0.002 | 2.19999e-09 | 0.0001 | 35.783 | 0.000 | 0.013 | 0.991 | 0.49200 |
| 7 | 14645949 | rs9638713 | G | A | -0.036 | 0.006 | 2.39999e-08 | 0.0001 | 31.151 | -0.075 | 0.068 | 0.271 | 0.98010 |
| 16 | 76895693 | rs9673839 | G | A | 0.013 | 0.002 | 8.80035e-11 | 0.0001 | 42.068 | 0.014 | 0.013 | 0.290 | 0.50100 |
| 17 | 80191995 | rs9674487 | G | C | 0.160 | 0.029 | 2.90001e-08 | 0.0001 | 30.769 | 0.025 | 0.064 | 0.689 | 0.13520 |
| 3 | 49920571 | rs9843653 | C | T | 0.029 | 0.002 | 7.70016e-49 | 0.0004 | 215.733 | 0.052 | 0.014 | 0.000 | 0.49200 |
| 3 | 45373442 | rs9852062 | A | T | -0.014 | 0.002 | 3.59998e-12 | 0.0001 | 48.313 | -0.035 | 0.014 | 0.010 | 0.58050 |
| 3 | 35683104 | rs9860326 | G | C | 0.015 | 0.002 | 7.50067e-12 | 0.0001 | 46.887 | 0.030 | 0.015 | 0.046 | 0.31210 |
| 13 | 107854612 | rs9888533 | T | C | 0.012 | 0.002 | 2.19999e-09 | 0.0001 | 35.768 | 0.002 | 0.013 | 0.863 | 0.54170 |
| 18 | 56882326 | rs9951619 | G | T | 0.015 | 0.002 | 2.90001e-10 | 0.0001 | 39.746 | 0.002 | 0.014 | 0.877 | 0.75840 |
| 4 | 112686354 | rs9991259 | A | G | 0.011 | 0.002 | 4.39997e-08 | 0.0001 | 29.975 | -0.008 | 0.014 | 0.581 | 0.63420 |

**Supplementary table 2.8. Detailed information of instrumental SNPs for Diastolic blood pressure** **in the analysis of PPD.**

| **Chr** | **Position** | **SNP** | **EA** | **OA** | **beta.exposure** | **se.exposure** | **pval.exposure** | **R2** | **F_val** | **beta.outcome** | **se.outcome** | **pval.outcome** | **Eaf.outcome** |
| --- | --- | --- | --- | --- | --- | --- | --- | --- | --- | --- | --- | --- | --- |
| 1 | 172346548 | rs1011731 | A | G | -0.014 | 0.002 | 1.000e-200 | 1e-04 | 80.789 | -0.017 | 0.013 | 0.210 | 0.559 |
| 1 | 41486245 | rs1138293 | T | C | -0.013 | 0.002 | 1.282e-10 | 1e-04 | 41.336 | 0.001 | 0.017 | 0.966 | 0.198 |
| 1 | 27138393 | rs12748152 | T | C | 0.016 | 0.003 | 1.533e-08 | 0e+00 | 32.011 | -0.029 | 0.025 | 0.249 | 0.077 |
| 1 | 78623626 | rs17391694 | T | C | -0.019 | 0.002 | 5.000e-17 | 1e-04 | 70.288 | -0.003 | 0.020 | 0.873 | 0.132 |
| 1 | 201860626 | rs2250377 | A | G | 0.011 | 0.002 | 2.329e-10 | 0e+00 | 40.169 | 0.017 | 0.015 | 0.250 | 0.295 |
| 1 | 3328659 | rs2493292 | T | C | 0.023 | 0.002 | 1.000e-200 | 1e-04 | 102.066 | -0.006 | 0.021 | 0.783 | 0.109 |
| 1 | 228126470 | rs2527614 | A | G | 0.013 | 0.002 | 2.000e-17 | 1e-04 | 71.646 | -0.007 | 0.013 | 0.606 | 0.537 |
| 1 | 43886494 | rs2782643 | T | C | 0.012 | 0.002 | 2.139e-14 | 1e-04 | 58.400 | -0.004 | 0.014 | 0.755 | 0.398 |
| 1 | 13802325 | rs3013105 | T | C | 0.010 | 0.002 | 1.256e-10 | 1e-04 | 41.375 | 0.002 | 0.014 | 0.886 | 0.633 |
| 1 | 153662423 | rs35479618 | A | G | 0.064 | 0.006 | 1.000e-200 | 1e-04 | 106.087 | 0.050 | 0.066 | 0.453 | 0.011 |
| 1 | 25044111 | rs4601530 | T | C | 0.018 | 0.002 | 1.000e-200 | 1e-04 | 97.883 | 0.005 | 0.015 | 0.722 | 0.280 |
| 1 | 113098534 | rs6658555 | T | C | -0.021 | 0.002 | 1.000e-200 | 2e-04 | 140.995 | -0.012 | 0.016 | 0.431 | 0.230 |
| 1 | 243385307 | rs6662118 | A | G | -0.024 | 0.002 | 1.000e-200 | 3e-04 | 189.835 | -0.001 | 0.014 | 0.918 | 0.636 |
| 1 | 230845794 | rs699 | A | G | -0.023 | 0.002 | 1.000e-200 | 3e-04 | 209.220 | -0.001 | 0.013 | 0.962 | 0.572 |
| 1 | 16259813 | rs848210 | A | G | 0.011 | 0.002 | 7.847e-12 | 1e-04 | 46.804 | 0.000 | 0.014 | 0.990 | 0.397 |
| 1 | 10796866 | rs880315 | T | C | -0.023 | 0.002 | 1.000e-200 | 2e-04 | 193.393 | -0.015 | 0.013 | 0.263 | 0.587 |
| 2 | 165047746 | rs10204405 | A | G | -0.020 | 0.002 | 1.000e-200 | 2e-04 | 154.570 | 0.002 | 0.014 | 0.908 | 0.376 |
| 2 | 46353166 | rs10495928 | A | G | 0.013 | 0.002 | 4.300e-16 | 1e-04 | 66.072 | 0.005 | 0.015 | 0.731 | 0.719 |
| 2 | 239877148 | rs12477314 | T | C | 0.012 | 0.002 | 3.553e-09 | 0e+00 | 34.855 | 0.005 | 0.016 | 0.758 | 0.211 |
| 2 | 219563602 | rs12993599 | A | G | -0.025 | 0.004 | 7.885e-13 | 1e-04 | 51.311 | -0.015 | 0.032 | 0.648 | 0.046 |
| 2 | 96795608 | rs1657502 | T | G | -0.017 | 0.002 | 1.000e-200 | 1e-04 | 107.468 | 0.030 | 0.013 | 0.022 | 0.503 |
| 2 | 37248015 | rs17020136 | T | C | 0.014 | 0.002 | 3.948e-12 | 1e-04 | 48.150 | -0.010 | 0.016 | 0.548 | 0.774 |
| 2 | 179721046 | rs17362588 | A | G | 0.031 | 0.003 | 1.000e-200 | 2e-04 | 127.344 | 0.005 | 0.025 | 0.830 | 0.076 |
| 2 | 190827023 | rs2053163 | A | G | 0.011 | 0.002 | 2.783e-10 | 0e+00 | 39.822 | 0.016 | 0.014 | 0.272 | 0.306 |
| 2 | 145739556 | rs2381683 | A | G | 0.015 | 0.002 | 1.000e-200 | 1e-04 | 89.883 | -0.026 | 0.013 | 0.051 | 0.434 |
| 2 | 86135154 | rs2679722 | A | G | 0.010 | 0.002 | 4.027e-09 | 0e+00 | 34.611 | 0.000 | 0.013 | 0.986 | 0.582 |
| 2 | 43625184 | rs33979934 | A | T | -0.012 | 0.002 | 4.888e-11 | 1e-04 | 43.222 | -0.002 | 0.015 | 0.895 | 0.274 |
| 2 | 43015719 | rs3816183 | T | C | 0.012 | 0.002 | 5.753e-11 | 1e-04 | 42.903 | 0.005 | 0.015 | 0.734 | 0.254 |
| 2 | 72707874 | rs653220 | A | G | 0.012 | 0.002 | 2.520e-10 | 0e+00 | 40.015 | 0.002 | 0.017 | 0.917 | 0.802 |
| 2 | 135650766 | rs6714498 | T | C | 0.010 | 0.002 | 7.522e-09 | 0e+00 | 33.395 | 0.018 | 0.014 | 0.188 | 0.604 |
| 2 | 25116977 | rs7586879 | T | C | -0.015 | 0.002 | 1.000e-200 | 1e-04 | 80.724 | -0.006 | 0.014 | 0.690 | 0.344 |
| 2 | 218674697 | rs918949 | T | C | -0.015 | 0.002 | 1.000e-200 | 1e-04 | 89.328 | -0.013 | 0.013 | 0.339 | 0.556 |
| 3 | 85460131 | rs1549979 | T | C | 0.011 | 0.002 | 1.107e-11 | 1e-04 | 46.130 | -0.015 | 0.015 | 0.317 | 0.708 |
| 3 | 101238259 | rs16843947 | A | G | -0.010 | 0.002 | 1.611e-09 | 0e+00 | 36.395 | -0.026 | 0.013 | 0.051 | 0.565 |
| 3 | 141134818 | rs16851397 | A | G | -0.039 | 0.004 | 1.000e-200 | 1e-04 | 113.429 | 0.057 | 0.037 | 0.121 | 0.965 |
| 3 | 158366900 | rs2303909 | A | G | 0.010 | 0.002 | 1.049e-09 | 0e+00 | 37.232 | -0.007 | 0.013 | 0.605 | 0.513 |
| 3 | 11400249 | rs2606736 | T | C | 0.010 | 0.002 | 9.338e-10 | 0e+00 | 37.459 | 0.016 | 0.013 | 0.247 | 0.577 |
| 3 | 14899778 | rs294636 | T | G | -0.015 | 0.002 | 6.796e-14 | 1e-04 | 56.126 | 0.009 | 0.019 | 0.650 | 0.857 |
| 3 | 122060404 | rs34173813 | T | C | -0.014 | 0.002 | 1.943e-09 | 0e+00 | 36.030 | -0.029 | 0.020 | 0.152 | 0.123 |
| 3 | 27472936 | rs3755652 | T | C | 0.016 | 0.002 | 1.000e-200 | 1e-04 | 77.487 | 0.010 | 0.017 | 0.566 | 0.183 |
| 3 | 56771251 | rs3772219 | A | C | 0.015 | 0.002 | 1.000e-200 | 1e-04 | 75.743 | 0.019 | 0.015 | 0.187 | 0.715 |
| 3 | 169100899 | rs448378 | A | G | -0.025 | 0.002 | 1.000e-200 | 3e-04 | 256.796 | 0.002 | 0.013 | 0.870 | 0.573 |
| 3 | 185307363 | rs4686683 | T | G | -0.016 | 0.002 | 1.000e-200 | 1e-04 | 102.358 | -0.010 | 0.014 | 0.458 | 0.626 |
| 3 | 153839959 | rs59508481 | T | C | -0.014 | 0.002 | 2.991e-14 | 1e-04 | 57.740 | -0.009 | 0.015 | 0.555 | 0.730 |
| 3 | 41756965 | rs61744388 | T | C | 0.029 | 0.002 | 1.000e-200 | 2e-04 | 184.922 | -0.001 | 0.016 | 0.973 | 0.208 |
| 3 | 111513720 | rs6438013 | T | G | -0.012 | 0.002 | 3.037e-12 | 1e-04 | 48.664 | 0.000 | 0.014 | 0.973 | 0.359 |
| 3 | 64705365 | rs6795735 | T | C | -0.013 | 0.002 | 1.190e-15 | 1e-04 | 64.075 | 0.019 | 0.014 | 0.176 | 0.360 |
| 4 | 2195024 | rs10018786 | T | G | -0.020 | 0.003 | 4.226e-14 | 1e-04 | 57.060 | -0.002 | 0.023 | 0.914 | 0.903 |
| 4 | 103188709 | rs13107325 | T | C | -0.060 | 0.003 | 1.000e-200 | 5e-04 | 374.333 | 0.122 | 0.058 | 0.035 | 0.014 |
| 4 | 111445587 | rs1448808 | A | G | 0.010 | 0.002 | 4.962e-11 | 1e-04 | 43.192 | -0.001 | 0.014 | 0.969 | 0.399 |
| 4 | 81184341 | rs16998073 | A | T | -0.042 | 0.002 | 1.000e-200 | 7e-04 | 587.407 | -0.010 | 0.014 | 0.479 | 0.686 |
| 4 | 17972372 | rs2320299 | A | G | 0.011 | 0.002 | 1.352e-10 | 1e-04 | 41.232 | 0.008 | 0.014 | 0.552 | 0.670 |
| 4 | 157720124 | rs4691380 | T | C | -0.011 | 0.002 | 5.861e-11 | 1e-04 | 42.866 | -0.021 | 0.015 | 0.178 | 0.249 |
| 4 | 144060464 | rs7666785 | A | G | 0.009 | 0.002 | 4.012e-08 | 0e+00 | 30.144 | -0.007 | 0.014 | 0.614 | 0.356 |
| 4 | 156635309 | rs7692387 | A | G | -0.024 | 0.002 | 1.000e-200 | 2e-04 | 141.849 | -0.026 | 0.016 | 0.093 | 0.237 |
| 4 | 119951647 | rs7698598 | A | G | 0.016 | 0.002 | 9.196e-12 | 1e-04 | 46.493 | 0.014 | 0.017 | 0.414 | 0.171 |
| 4 | 109066366 | rs922163 | T | C | -0.010 | 0.002 | 1.328e-10 | 1e-04 | 41.266 | -0.017 | 0.013 | 0.196 | 0.582 |
| 5 | 32830521 | rs1173727 | T | C | -0.029 | 0.002 | 1.000e-200 | 4e-04 | 335.986 | 0.005 | 0.013 | 0.700 | 0.414 |
| 5 | 157528319 | rs13155377 | T | G | 0.012 | 0.002 | 7.314e-13 | 1e-04 | 51.458 | -0.008 | 0.013 | 0.553 | 0.556 |
| 5 | 122657199 | rs1582931 | A | G | 0.024 | 0.002 | 1.000e-200 | 3e-04 | 156.315 | 0.004 | 0.013 | 0.789 | 0.513 |
| 5 | 131447104 | rs247008 | A | G | 0.009 | 0.002 | 2.783e-08 | 0e+00 | 30.853 | -0.007 | 0.014 | 0.598 | 0.385 |
| 5 | 96244549 | rs2549794 | T | C | -0.012 | 0.002 | 1.916e-12 | 1e-04 | 49.568 | -0.010 | 0.014 | 0.473 | 0.621 |
| 5 | 172196752 | rs34471628 | A | G | 0.024 | 0.004 | 6.188e-09 | 0e+00 | 33.775 | -0.001 | 0.040 | 0.986 | 0.973 |
| 5 | 74967386 | rs40060 | T | C | -0.015 | 0.002 | 1.000e-200 | 1e-04 | 84.232 | -0.014 | 0.013 | 0.299 | 0.594 |
| 5 | 112366678 | rs4705752 | A | G | 0.009 | 0.002 | 3.955e-08 | 0e+00 | 30.172 | 0.000 | 0.013 | 0.973 | 0.489 |
| 5 | 173362458 | rs6861681 | A | G | -0.017 | 0.002 | 1.000e-200 | 1e-04 | 99.978 | 0.023 | 0.014 | 0.089 | 0.413 |
| 5 | 56177743 | rs832582 | A | G | 0.012 | 0.002 | 5.293e-09 | 0e+00 | 34.079 | 0.014 | 0.017 | 0.395 | 0.809 |
| 6 | 79655477 | rs10943605 | A | G | 0.015 | 0.002 | 1.000e-200 | 1e-04 | 96.787 | -0.008 | 0.013 | 0.567 | 0.456 |
| 6 | 118667522 | rs11153730 | T | C | 0.013 | 0.002 | 6.000e-17 | 1e-04 | 69.798 | -0.024 | 0.013 | 0.073 | 0.533 |
| 6 | 55924962 | rs12209452 | A | G | 0.029 | 0.004 | 6.176e-14 | 1e-04 | 56.314 | -0.007 | 0.027 | 0.809 | 0.934 |
| 6 | 32913246 | rs1480380 | T | C | -0.028 | 0.003 | 1.000e-200 | 1e-04 | 100.762 | 0.023 | 0.026 | 0.370 | 0.074 |
| 6 | 147699123 | rs2328813 | A | C | -0.010 | 0.002 | 2.320e-10 | 1e-04 | 40.177 | -0.007 | 0.013 | 0.587 | 0.516 |
| 6 | 51611470 | rs2397060 | T | C | -0.016 | 0.002 | 4.558e-12 | 1e-04 | 47.869 | -0.033 | 0.018 | 0.064 | 0.832 |
| 6 | 117210052 | rs339331 | T | C | -0.010 | 0.002 | 8.565e-09 | 0e+00 | 33.142 | 0.006 | 0.014 | 0.692 | 0.668 |
| 6 | 22125964 | rs4712653 | T | C | -0.012 | 0.002 | 5.475e-14 | 1e-04 | 56.552 | 0.015 | 0.013 | 0.253 | 0.432 |
| 6 | 127208390 | rs4897193 | A | G | 0.022 | 0.002 | 1.000e-200 | 2e-04 | 192.285 | 0.013 | 0.013 | 0.321 | 0.555 |
| 6 | 139829666 | rs605066 | T | C | -0.011 | 0.002 | 5.733e-10 | 1e-04 | 38.410 | 0.000 | 0.013 | 0.982 | 0.519 |
| 6 | 43758873 | rs6905288 | A | G | 0.015 | 0.002 | 1.000e-200 | 1e-04 | 94.169 | -0.002 | 0.013 | 0.908 | 0.566 |
| 6 | 20679709 | rs7756992 | A | G | -0.011 | 0.002 | 1.058e-09 | 0e+00 | 37.214 | 0.001 | 0.014 | 0.940 | 0.667 |
| 6 | 43806609 | rs881858 | A | G | 0.014 | 0.002 | 4.000e-17 | 1e-04 | 70.355 | 0.003 | 0.014 | 0.854 | 0.679 |
| 6 | 25641200 | rs932316 | T | C | -0.023 | 0.002 | 1.000e-200 | 2e-04 | 122.662 | 0.001 | 0.017 | 0.961 | 0.824 |
| 6 | 97063522 | rs9373985 | C | G | -0.009 | 0.002 | 1.948e-08 | 0e+00 | 31.546 | 0.022 | 0.014 | 0.119 | 0.700 |
| 6 | 117522156 | rs961764 | C | G | -0.013 | 0.002 | 8.720e-15 | 1e-04 | 60.165 | 0.007 | 0.013 | 0.582 | 0.416 |
| 7 | 151415041 | rs10224002 | A | G | -0.019 | 0.002 | 1.000e-200 | 1e-04 | 120.088 | 0.006 | 0.016 | 0.719 | 0.766 |
| 7 | 7268431 | rs10259085 | T | C | 0.010 | 0.002 | 1.507e-09 | 0e+00 | 36.526 | 0.030 | 0.013 | 0.024 | 0.457 |
| 7 | 129663496 | rs11556924 | T | C | -0.016 | 0.002 | 1.000e-200 | 1e-04 | 94.656 | 0.007 | 0.014 | 0.642 | 0.328 |
| 7 | 46008110 | rs11977526 | A | G | 0.010 | 0.002 | 6.296e-10 | 0e+00 | 38.228 | -0.008 | 0.014 | 0.596 | 0.326 |
| 7 | 111580166 | rs144867634 | T | C | 0.029 | 0.005 | 5.233e-09 | 0e+00 | 34.101 | -0.014 | 0.048 | 0.765 | 0.980 |
| 7 | 96410250 | rs1449592 | A | G | -0.010 | 0.002 | 5.255e-11 | 1e-04 | 43.080 | 0.009 | 0.013 | 0.484 | 0.544 |
| 7 | 89854446 | rs194520 | T | G | 0.011 | 0.002 | 8.420e-11 | 1e-04 | 42.158 | 0.008 | 0.013 | 0.531 | 0.420 |
| 7 | 100453208 | rs314370 | T | C | -0.017 | 0.002 | 4.000e-17 | 1e-04 | 70.716 | -0.005 | 0.016 | 0.745 | 0.780 |
| 7 | 73020337 | rs3812316 | C | G | 0.014 | 0.002 | 4.979e-09 | 0e+00 | 34.198 | 0.028 | 0.020 | 0.152 | 0.872 |
| 7 | 27159136 | rs6969780 | C | G | 0.017 | 0.003 | 1.854e-10 | 1e-04 | 40.615 | -0.050 | 0.020 | 0.012 | 0.126 |
| 8 | 38095662 | rs2306899 | T | C | 0.012 | 0.002 | 2.638e-11 | 1e-04 | 44.429 | -0.014 | 0.017 | 0.416 | 0.190 |
| 8 | 120353267 | rs2469997 | C | G | 0.019 | 0.002 | 1.000e-200 | 1e-04 | 84.908 | 0.020 | 0.020 | 0.317 | 0.879 |
| 8 | 142367087 | rs34591516 | T | C | 0.029 | 0.004 | 1.030e-15 | 1e-04 | 64.363 | -0.075 | 0.028 | 0.008 | 0.061 |
| 8 | 145108151 | rs61732533 | A | G | -0.021 | 0.004 | 3.632e-08 | 0e+00 | 30.336 | -0.029 | 0.034 | 0.392 | 0.041 |
| 8 | 81426196 | rs76767219 | A | C | -0.029 | 0.004 | 3.425e-11 | 1e-04 | 43.918 | 0.024 | 0.044 | 0.576 | 0.025 |
| 8 | 9649769 | rs7830613 | T | C | 0.014 | 0.002 | 1.550e-15 | 1e-04 | 63.561 | 0.023 | 0.014 | 0.086 | 0.421 |
| 8 | 129316014 | rs975730 | A | G | -0.009 | 0.002 | 1.531e-08 | 0e+00 | 32.013 | 0.019 | 0.015 | 0.210 | 0.279 |
| 9 | 113169775 | rs111245230 | T | C | -0.033 | 0.004 | 4.409e-14 | 1e-04 | 56.977 | 0.029 | 0.061 | 0.635 | 0.988 |
| 9 | 117166246 | rs2274159 | A | G | -0.012 | 0.002 | 5.430e-15 | 1e-04 | 61.097 | -0.026 | 0.013 | 0.051 | 0.526 |
| 9 | 139368953 | rs3812594 | A | G | 0.010 | 0.002 | 3.482e-08 | 0e+00 | 30.418 | 0.033 | 0.015 | 0.032 | 0.249 |
| 9 | 9261737 | rs4626664 | A | G | 0.013 | 0.002 | 4.955e-10 | 0e+00 | 38.695 | 0.000 | 0.018 | 1.000 | 0.163 |
| 9 | 128369224 | rs534214 | A | G | 0.011 | 0.002 | 2.115e-11 | 1e-04 | 44.861 | -0.009 | 0.013 | 0.519 | 0.508 |
| 9 | 136153875 | rs651007 | T | C | -0.026 | 0.002 | 1.000e-200 | 2e-04 | 176.426 | -0.027 | 0.016 | 0.086 | 0.224 |
| 9 | 21816758 | rs7023954 | A | G | 0.010 | 0.002 | 1.224e-09 | 0e+00 | 36.931 | 0.005 | 0.013 | 0.732 | 0.433 |
| 9 | 35906471 | rs76452347 | T | C | -0.021 | 0.002 | 1.000e-200 | 1e-04 | 109.469 | 0.002 | 0.018 | 0.927 | 0.179 |
| 10 | 64564934 | rs10995311 | C | G | 0.020 | 0.002 | 1.000e-200 | 2e-04 | 153.650 | 0.004 | 0.014 | 0.767 | 0.611 |
| 10 | 18708798 | rs11014166 | A | T | 0.027 | 0.002 | 1.000e-200 | 3e-04 | 267.062 | 0.006 | 0.015 | 0.691 | 0.721 |
| 10 | 134459388 | rs1133400 | A | G | -0.011 | 0.002 | 2.196e-08 | 0e+00 | 31.313 | 0.011 | 0.018 | 0.525 | 0.826 |
| 10 | 115457264 | rs11555408 | T | G | 0.014 | 0.003 | 3.133e-08 | 0e+00 | 30.623 | 0.028 | 0.027 | 0.285 | 0.934 |
| 10 | 70332672 | rs12773594 | A | T | 0.013 | 0.002 | 5.999e-09 | 0e+00 | 33.835 | -0.012 | 0.016 | 0.435 | 0.240 |
| 10 | 45399088 | rs1408814 | A | G | 0.009 | 0.002 | 1.962e-08 | 0e+00 | 31.532 | 0.018 | 0.013 | 0.170 | 0.464 |
| 10 | 63524591 | rs1530440 | T | C | -0.033 | 0.002 | 1.000e-200 | 3e-04 | 278.207 | -0.003 | 0.017 | 0.862 | 0.198 |
| 10 | 104591393 | rs17115100 | T | G | -0.040 | 0.003 | 1.000e-200 | 3e-04 | 210.976 | -0.025 | 0.023 | 0.277 | 0.095 |
| 10 | 121429633 | rs2234962 | T | C | 0.019 | 0.002 | 1.000e-200 | 1e-04 | 98.004 | -0.006 | 0.016 | 0.702 | 0.778 |
| 10 | 30316072 | rs3739998 | C | G | 0.012 | 0.002 | 1.343e-14 | 1e-04 | 59.316 | 0.000 | 0.013 | 0.980 | 0.557 |
| 10 | 75415677 | rs60632610 | T | C | 0.015 | 0.002 | 4.204e-11 | 1e-04 | 43.516 | -0.006 | 0.019 | 0.763 | 0.148 |
| 10 | 115789375 | rs7076938 | T | C | 0.030 | 0.002 | 1.000e-200 | 4e-04 | 286.368 | -0.006 | 0.015 | 0.696 | 0.750 |
| 10 | 28228865 | rs7893462 | A | G | -0.010 | 0.002 | 5.926e-11 | 1e-04 | 42.845 | -0.025 | 0.013 | 0.055 | 0.479 |
| 10 | 96013705 | rs9419788 | A | G | -0.019 | 0.002 | 1.000e-200 | 2e-04 | 138.162 | -0.012 | 0.013 | 0.380 | 0.511 |
| 11 | 27487992 | rs11030016 | T | C | -0.011 | 0.002 | 1.262e-10 | 1e-04 | 41.367 | -0.023 | 0.014 | 0.103 | 0.678 |
| 11 | 130275749 | rs11222085 | T | C | 0.015 | 0.002 | 1.283e-13 | 1e-04 | 54.877 | 0.009 | 0.019 | 0.639 | 0.852 |
| 11 | 58207203 | rs11229457 | T | C | -0.011 | 0.002 | 8.097e-09 | 0e+00 | 33.252 | 0.006 | 0.015 | 0.693 | 0.247 |
| 11 | 100470306 | rs1145415 | A | G | 0.015 | 0.002 | 1.000e-200 | 1e-04 | 91.059 | 0.009 | 0.013 | 0.488 | 0.471 |
| 11 | 65391317 | rs12801636 | A | G | -0.023 | 0.002 | 1.000e-200 | 2e-04 | 143.272 | -0.013 | 0.016 | 0.424 | 0.204 |
| 11 | 31210771 | rs16921914 | A | G | 0.011 | 0.002 | 7.384e-09 | 0e+00 | 33.431 | 0.025 | 0.014 | 0.074 | 0.365 |
| 11 | 77583266 | rs2186564 | A | G | 0.016 | 0.003 | 1.426e-10 | 1e-04 | 41.128 | 0.028 | 0.017 | 0.090 | 0.193 |
| 11 | 14264916 | rs2303973 | A | G | 0.009 | 0.002 | 2.830e-09 | 0e+00 | 35.298 | 0.000 | 0.013 | 0.989 | 0.583 |
| 11 | 78091150 | rs2373115 | A | C | -0.012 | 0.002 | 1.680e-08 | 0e+00 | 31.833 | 0.001 | 0.017 | 0.956 | 0.197 |
| 11 | 16274295 | rs4757388 | A | G | 0.014 | 0.002 | 1.000e-200 | 1e-04 | 78.626 | -0.007 | 0.015 | 0.637 | 0.284 |
| 11 | 10673739 | rs4909945 | T | C | 0.018 | 0.002 | 1.000e-200 | 1e-04 | 117.923 | 0.004 | 0.014 | 0.779 | 0.331 |
| 11 | 47336320 | rs7944584 | A | T | -0.022 | 0.002 | 1.000e-200 | 2e-04 | 149.031 | -0.030 | 0.017 | 0.082 | 0.815 |
| 11 | 1874072 | rs907611 | A | G | 0.018 | 0.002 | 1.000e-200 | 1e-04 | 100.434 | -0.001 | 0.014 | 0.920 | 0.321 |
| 11 | 1941946 | rs909116 | T | C | -0.015 | 0.002 | 1.000e-200 | 1e-04 | 92.224 | -0.012 | 0.013 | 0.383 | 0.458 |
| 11 | 27667202 | rs925946 | T | G | -0.015 | 0.002 | 1.000e-200 | 1e-04 | 81.780 | 0.006 | 0.014 | 0.655 | 0.338 |
| 11 | 69462910 | rs9344 | A | G | -0.010 | 0.002 | 1.704e-10 | 0e+00 | 40.779 | 0.013 | 0.013 | 0.334 | 0.475 |
| 12 | 123806219 | rs1060105 | T | C | -0.020 | 0.002 | 1.000e-200 | 1e-04 | 103.359 | -0.021 | 0.016 | 0.207 | 0.209 |
| 12 | 20154110 | rs10743347 | A | G | 0.021 | 0.003 | 2.000e-17 | 1e-04 | 72.102 | -0.025 | 0.030 | 0.402 | 0.948 |
| 12 | 24770878 | rs17287293 | A | G | 0.014 | 0.002 | 7.053e-10 | 0e+00 | 38.006 | -0.033 | 0.022 | 0.130 | 0.894 |
| 12 | 56482180 | rs2292239 | T | G | -0.011 | 0.002 | 6.441e-11 | 1e-04 | 42.682 | 0.033 | 0.014 | 0.023 | 0.307 |
| 12 | 90008959 | rs2681472 | A | G | 0.034 | 0.002 | 1.000e-200 | 3e-04 | 263.678 | 0.058 | 0.025 | 0.021 | 0.925 |
| 12 | 57109931 | rs2926747 | A | T | 0.013 | 0.002 | 9.250e-15 | 1e-04 | 60.048 | 0.029 | 0.015 | 0.046 | 0.285 |
| 12 | 33510115 | rs4587807 | A | G | -0.009 | 0.002 | 1.513e-08 | 0e+00 | 32.037 | 0.021 | 0.014 | 0.113 | 0.494 |
| 12 | 111385296 | rs6489844 | A | G | 0.017 | 0.002 | 1.000e-200 | 1e-04 | 111.875 | 0.010 | 0.013 | 0.447 | 0.483 |
| 12 | 69842643 | rs6581895 | T | C | -0.010 | 0.002 | 4.083e-08 | 0e+00 | 30.110 | -0.042 | 0.015 | 0.006 | 0.750 |
| 12 | 20473758 | rs7134375 | A | C | -0.010 | 0.002 | 6.630e-11 | 1e-04 | 42.625 | 0.012 | 0.014 | 0.374 | 0.407 |
| 12 | 50537815 | rs7302981 | A | G | 0.026 | 0.002 | 1.000e-200 | 3e-04 | 262.375 | -0.009 | 0.014 | 0.528 | 0.370 |
| 13 | 28624294 | rs1933437 | A | G | 0.009 | 0.002 | 1.725e-08 | 0e+00 | 31.782 | 0.001 | 0.014 | 0.936 | 0.609 |
| 13 | 51105334 | rs3118905 | A | G | 0.011 | 0.002 | 1.979e-09 | 0e+00 | 35.994 | 0.003 | 0.014 | 0.857 | 0.311 |
| 14 | 23313633 | rs17880989 | A | G | 0.032 | 0.005 | 4.324e-11 | 1e-04 | 43.462 | 0.011 | 0.044 | 0.793 | 0.024 |
| 14 | 24830850 | rs1950500 | T | C | 0.013 | 0.002 | 7.206e-14 | 1e-04 | 56.011 | 0.003 | 0.015 | 0.835 | 0.284 |
| 14 | 69278204 | rs4899260 | T | C | 0.012 | 0.002 | 1.668e-11 | 1e-04 | 45.326 | -0.018 | 0.016 | 0.263 | 0.233 |
| 15 | 43816917 | rs1060939 | A | G | 0.016 | 0.002 | 1.000e-200 | 1e-04 | 79.149 | -0.036 | 0.016 | 0.027 | 0.214 |
| 15 | 91416550 | rs17514846 | A | C | 0.026 | 0.002 | 1.000e-200 | 3e-04 | 267.929 | -0.016 | 0.014 | 0.224 | 0.394 |
| 15 | 42115747 | rs3959569 | C | G | 0.017 | 0.002 | 1.000e-200 | 1e-04 | 79.445 | 0.035 | 0.014 | 0.010 | 0.362 |
| 15 | 95378243 | rs6496047 | T | C | -0.013 | 0.002 | 3.000e-17 | 1e-04 | 71.271 | -0.002 | 0.013 | 0.856 | 0.560 |
| 15 | 67528374 | rs7173826 | T | G | 0.010 | 0.002 | 1.812e-09 | 0e+00 | 36.166 | -0.001 | 0.014 | 0.969 | 0.648 |
| 15 | 75393720 | rs8040061 | A | C | 0.020 | 0.002 | 1.000e-200 | 2e-04 | 145.197 | 0.006 | 0.013 | 0.672 | 0.486 |
| 16 | 4942099 | rs1049205 | T | C | 0.015 | 0.002 | 1.000e-200 | 1e-04 | 88.599 | 0.005 | 0.014 | 0.691 | 0.589 |
| 16 | 89704365 | rs1126464 | C | G | 0.020 | 0.002 | 1.000e-200 | 1e-04 | 112.025 | 0.011 | 0.014 | 0.445 | 0.311 |
| 16 | 69224615 | rs1424114 | T | C | -0.011 | 0.002 | 9.730e-12 | 1e-04 | 46.382 | 0.033 | 0.014 | 0.017 | 0.328 |
| 16 | 2070568 | rs2286472 | A | C | -0.013 | 0.002 | 4.120e-15 | 1e-04 | 61.638 | -0.003 | 0.015 | 0.824 | 0.727 |
| 16 | 75390316 | rs2865531 | A | T | 0.011 | 0.002 | 2.123e-09 | 1e-04 | 35.857 | -0.028 | 0.013 | 0.033 | 0.571 |
| 16 | 30936081 | rs35675346 | A | G | 0.015 | 0.002 | 2.560e-15 | 1e-04 | 62.578 | -0.018 | 0.015 | 0.223 | 0.271 |
| 16 | 27480797 | rs61739285 | T | C | 0.028 | 0.004 | 1.174e-10 | 1e-04 | 41.509 | -0.005 | 0.030 | 0.869 | 0.052 |
| 16 | 20410547 | rs9926580 | T | C | -0.017 | 0.002 | 1.000e-200 | 1e-04 | 93.382 | 0.022 | 0.015 | 0.127 | 0.294 |
| 17 | 19239432 | rs1043809 | T | C | 0.014 | 0.002 | 3.453e-11 | 1e-04 | 43.902 | -0.016 | 0.016 | 0.333 | 0.794 |
| 17 | 43183108 | rs1053739 | A | G | 0.015 | 0.002 | 3.000e-17 | 1e-04 | 71.209 | 0.008 | 0.014 | 0.574 | 0.348 |
| 17 | 73949540 | rs1135640 | C | G | 0.009 | 0.002 | 1.981e-08 | 0e+00 | 31.513 | 0.005 | 0.015 | 0.736 | 0.754 |
| 17 | 47440466 | rs16948048 | A | G | -0.017 | 0.002 | 1.000e-200 | 1e-04 | 117.197 | 0.002 | 0.013 | 0.905 | 0.573 |
| 17 | 2203025 | rs1885987 | T | G | 0.011 | 0.002 | 2.434e-11 | 1e-04 | 44.586 | -0.029 | 0.014 | 0.031 | 0.603 |
| 17 | 29247715 | rs3760318 | A | G | -0.009 | 0.002 | 1.935e-08 | 0e+00 | 31.558 | -0.012 | 0.014 | 0.373 | 0.373 |
| 17 | 7163739 | rs4562 | A | G | -0.013 | 0.002 | 7.976e-14 | 1e-04 | 55.812 | -0.003 | 0.014 | 0.815 | 0.342 |
| 17 | 61055390 | rs4968639 | A | G | 0.011 | 0.002 | 2.144e-08 | 0e+00 | 31.360 | -0.020 | 0.016 | 0.199 | 0.772 |
| 17 | 59483766 | rs8068318 | T | C | 0.016 | 0.002 | 1.000e-200 | 1e-04 | 84.587 | 0.027 | 0.015 | 0.064 | 0.721 |
| 18 | 72999359 | rs55679337 | T | C | -0.010 | 0.002 | 3.922e-09 | 0e+00 | 34.662 | 0.043 | 0.014 | 0.003 | 0.691 |
| 18 | 10752666 | rs7234309 | T | C | -0.011 | 0.002 | 7.428e-10 | 0e+00 | 37.905 | 0.001 | 0.016 | 0.974 | 0.228 |
| 19 | 17170885 | rs1130222 | T | C | 0.012 | 0.002 | 1.282e-14 | 1e-04 | 59.408 | -0.006 | 0.013 | 0.662 | 0.446 |
| 19 | 19789528 | rs2304130 | A | G | -0.026 | 0.003 | 1.000e-200 | 1e-04 | 83.993 | 0.042 | 0.029 | 0.141 | 0.942 |
| 19 | 47569003 | rs3810291 | A | G | -0.011 | 0.002 | 9.055e-12 | 1e-04 | 46.523 | -0.003 | 0.014 | 0.822 | 0.633 |
| 19 | 11285390 | rs440677 | A | G | 0.010 | 0.002 | 1.045e-09 | 0e+00 | 37.239 | -0.007 | 0.013 | 0.619 | 0.543 |
| 19 | 31863085 | rs7248171 | A | G | 0.014 | 0.002 | 1.000e-17 | 1e-04 | 73.410 | -0.022 | 0.013 | 0.091 | 0.467 |
| 19 | 30296853 | rs8102137 | T | C | -0.018 | 0.002 | 1.000e-200 | 2e-04 | 122.640 | 0.013 | 0.014 | 0.356 | 0.649 |
| 19 | 41038574 | rs814501 | A | G | -0.009 | 0.002 | 6.058e-09 | 0e+00 | 33.816 | 0.010 | 0.013 | 0.451 | 0.470 |
| 20 | 4101800 | rs1741344 | T | C | 0.010 | 0.002 | 1.534e-10 | 0e+00 | 40.985 | -0.019 | 0.014 | 0.174 | 0.654 |
| 20 | 10965998 | rs1887320 | A | G | 0.029 | 0.002 | 1.000e-200 | 4e-04 | 354.476 | 0.002 | 0.013 | 0.864 | 0.434 |
| 20 | 62326110 | rs3208008 | A | C | -0.013 | 0.002 | 2.072e-12 | 1e-04 | 49.415 | -0.013 | 0.017 | 0.442 | 0.199 |
| 20 | 57769140 | rs55786258 | C | G | 0.021 | 0.002 | 1.000e-200 | 1e-04 | 115.333 | 0.021 | 0.015 | 0.175 | 0.250 |
| 20 | 10603750 | rs6077853 | A | G | -0.019 | 0.002 | 1.000e-200 | 1e-04 | 80.545 | 0.011 | 0.021 | 0.583 | 0.118 |
| 20 | 47308798 | rs6095241 | A | G | -0.014 | 0.002 | 1.000e-200 | 1e-04 | 75.700 | -0.012 | 0.014 | 0.387 | 0.387 |
| 20 | 48301146 | rs6512586 | A | G | -0.009 | 0.002 | 9.354e-09 | 0e+00 | 32.971 | -0.015 | 0.013 | 0.262 | 0.499 |
| 20 | 30108533 | rs879597 | A | G | 0.015 | 0.002 | 1.835e-10 | 1e-04 | 40.635 | 0.016 | 0.017 | 0.346 | 0.794 |
| 21 | 45107562 | rs9306160 | T | C | -0.013 | 0.002 | 2.700e-16 | 1e-04 | 66.946 | -0.019 | 0.014 | 0.183 | 0.315 |
| 21 | 39972727 | rs9636639 | A | C | 0.011 | 0.002 | 7.055e-13 | 1e-04 | 51.529 | -0.010 | 0.013 | 0.438 | 0.522 |
| 22 | 31998612 | rs2006771 | A | G | 0.009 | 0.002 | 4.581e-08 | 0e+00 | 29.886 | -0.015 | 0.013 | 0.252 | 0.418 |
| 22 | 29451671 | rs4823006 | A | G | 0.014 | 0.002 | 1.000e-200 | 1e-04 | 79.364 | -0.028 | 0.013 | 0.038 | 0.570 |

**Supplementary table 2.9. Detailed information of instrumental SNPs for Pulse pressure in the analysis of PPD.**

| **Chr** | **Position** | **SNP** | **EA** | **OA** | **beta.exposure** | **se.exposure** | **pval.exposure** | **R2** | **F_val** | **beta.outcome** | **se.outcome** | **pval.outcome** | **Eaf.outcome** |
| --- | --- | --- | --- | --- | --- | --- | --- | --- | --- | --- | --- | --- | --- |
| 4 | 10140259 | rs10025456 | T | C | 0.010 | 0.002 | 2.333e-10 | 0e+00 | 40.165 | 0.001 | 0.013 | 0.937 | 0.47910 |
| 7 | 151415041 | rs10224002 | A | G | -0.014 | 0.002 | 1.200e-16 | 1e-04 | 68.485 | 0.006 | 0.016 | 0.719 | 0.29820 |
| 15 | 48702873 | rs1042078 | A | G | 0.016 | 0.002 | 1.000e-200 | 1e-04 | 84.955 | -0.024 | 0.015 | 0.100 | 0.72860 |
| 10 | 124214251 | rs10490923 | A | G | 0.013 | 0.002 | 1.890e-08 | 0e+00 | 31.604 | -0.022 | 0.023 | 0.345 | 0.13020 |
| 16 | 4942099 | rs1049205 | T | C | 0.010 | 0.002 | 9.411e-11 | 1e-04 | 41.940 | 0.005 | 0.014 | 0.691 | 0.53880 |
| 2 | 46353166 | rs10495928 | A | G | -0.012 | 0.002 | 1.844e-13 | 1e-04 | 54.164 | 0.005 | 0.015 | 0.731 | 0.31710 |
| 2 | 179839888 | rs10497529 | A | G | -0.037 | 0.004 | 1.000e-200 | 1e-04 | 78.429 | 0.023 | 0.047 | 0.618 | 0.02883 |
| 14 | 93103309 | rs10498635 | T | C | -0.011 | 0.002 | 3.397e-08 | 0e+00 | 30.466 | 0.015 | 0.019 | 0.449 | 0.18090 |
| 13 | 27879526 | rs10507380 | A | G | 0.014 | 0.002 | 1.507e-09 | 0e+00 | 36.526 | 0.006 | 0.016 | 0.701 | 0.15010 |
| 11 | 47306585 | rs1051006 | A | G | -0.019 | 0.002 | 1.000e-200 | 1e-04 | 83.905 | -0.028 | 0.015 | 0.068 | 0.16200 |
| 10 | 61552692 | rs1053266 | T | G | 0.010 | 0.002 | 1.798e-09 | 0e+00 | 36.181 | -0.005 | 0.013 | 0.687 | 0.45030 |
| 11 | 89228425 | rs10765211 | A | G | -0.018 | 0.002 | 1.000e-200 | 1e-04 | 115.246 | 0.005 | 0.013 | 0.711 | 0.65010 |
| 12 | 20230639 | rs10770612 | A | G | 0.026 | 0.002 | 1.000e-200 | 2e-04 | 172.059 | 0.005 | 0.016 | 0.756 | 0.17590 |
| 10 | 123033549 | rs10788160 | A | G | 0.011 | 0.002 | 1.313e-10 | 1e-04 | 41.289 | 0.016 | 0.014 | 0.276 | 0.26140 |
| 11 | 92708710 | rs10830963 | C | G | -0.011 | 0.002 | 8.698e-10 | 0e+00 | 37.597 | -0.033 | 0.014 | 0.018 | 0.28830 |
| 1 | 169099483 | rs10919071 | A | G | -0.018 | 0.002 | 6.930e-15 | 1e-04 | 60.618 | -0.016 | 0.021 | 0.467 | 0.13020 |
| 3 | 169514585 | rs10936600 | A | T | 0.013 | 0.002 | 2.142e-11 | 1e-04 | 44.836 | -0.009 | 0.015 | 0.527 | 0.24250 |
| 10 | 75902177 | rs11000889 | A | G | -0.010 | 0.002 | 2.002e-09 | 0e+00 | 35.972 | -0.015 | 0.013 | 0.265 | 0.62520 |
| 10 | 18708798 | rs11014166 | A | T | 0.018 | 0.002 | 1.000e-200 | 1e-04 | 117.621 | 0.006 | 0.015 | 0.691 | 0.32210 |
| 12 | 427575 | rs11062385 | A | G | -0.011 | 0.002 | 1.609e-10 | 0e+00 | 40.892 | -0.005 | 0.014 | 0.748 | 0.23260 |
| 9 | 113169775 | rs111245230 | T | C | -0.032 | 0.004 | 7.455e-14 | 1e-04 | 55.944 | 0.029 | 0.061 | 0.635 | 0.03181 |
| 10 | 104906211 | rs11191580 | T | C | 0.048 | 0.003 | 1.000e-200 | 3e-04 | 274.286 | 0.030 | 0.024 | 0.208 | 0.08748 |
| 11 | 130275749 | rs11222085 | T | C | -0.025 | 0.002 | 1.000e-200 | 2e-04 | 155.602 | 0.009 | 0.019 | 0.639 | 0.17500 |
| 12 | 49399132 | rs1126930 | C | G | 0.033 | 0.004 | 5.890e-15 | 1e-04 | 60.938 | -0.017 | 0.038 | 0.652 | 0.02386 |
| 9 | 127975654 | rs1128362 | T | C | 0.029 | 0.002 | 1.000e-200 | 2e-04 | 163.691 | -0.012 | 0.020 | 0.560 | 0.13620 |
| 10 | 134459388 | rs1133400 | A | G | -0.012 | 0.002 | 5.891e-10 | 0e+00 | 38.357 | 0.011 | 0.018 | 0.525 | 0.21370 |
| 7 | 89938680 | rs1134956 | T | C | 0.010 | 0.002 | 3.671e-11 | 1e-04 | 43.782 | -0.007 | 0.014 | 0.617 | 0.55770 |
| 17 | 73949540 | rs1135640 | C | G | 0.010 | 0.002 | 1.428e-08 | 0e+00 | 32.149 | 0.005 | 0.015 | 0.736 | 0.64020 |
| 11 | 100470306 | rs1145415 | A | G | 0.011 | 0.002 | 7.442e-12 | 1e-04 | 46.907 | 0.009 | 0.013 | 0.488 | 0.45130 |
| 3 | 99886662 | rs11537816 | A | G | -0.015 | 0.002 | 5.983e-13 | 1e-04 | 51.853 | 0.008 | 0.017 | 0.648 | 0.16200 |
| 17 | 7462555 | rs11552708 | A | G | -0.026 | 0.003 | 1.000e-200 | 1e-04 | 79.512 | -0.008 | 0.025 | 0.740 | 0.10040 |
| 11 | 118486067 | rs11603023 | T | C | -0.010 | 0.002 | 5.576e-11 | 1e-04 | 42.964 | 0.027 | 0.013 | 0.048 | 0.56260 |
| 16 | 24788645 | rs11639856 | A | T | -0.020 | 0.002 | 1.000e-200 | 1e-04 | 101.224 | 0.011 | 0.019 | 0.584 | 0.16200 |
| 5 | 32830521 | rs1173727 | T | C | -0.027 | 0.002 | 1.000e-200 | 3e-04 | 282.721 | 0.005 | 0.013 | 0.700 | 0.57950 |
| 17 | 17715101 | rs11868035 | A | G | 0.010 | 0.002 | 4.883e-09 | 0e+00 | 34.236 | -0.006 | 0.014 | 0.682 | 0.33800 |
| 7 | 46008110 | rs11977526 | A | G | -0.035 | 0.002 | 1.000e-200 | 6e-04 | 486.922 | -0.008 | 0.014 | 0.596 | 0.40260 |
| 14 | 23761094 | rs12050260 | T | C | 0.015 | 0.002 | 1.000e-200 | 1e-04 | 86.553 | 0.004 | 0.014 | 0.789 | 0.65310 |
| 1 | 16042766 | rs12091750 | T | C | 0.017 | 0.002 | 1.000e-200 | 1e-04 | 89.485 | -0.008 | 0.015 | 0.601 | 0.24450 |
| 6 | 122158270 | rs12110693 | A | G | -0.028 | 0.003 | 1.000e-200 | 1e-04 | 119.350 | 0.006 | 0.020 | 0.762 | 0.10830 |
| 1 | 176833497 | rs12118933 | C | G | -0.016 | 0.003 | 9.222e-09 | 0e+00 | 32.999 | 0.013 | 0.024 | 0.586 | 0.11030 |
| 6 | 7720059 | rs12198986 | A | G | 0.014 | 0.002 | 1.000e-200 | 1e-04 | 80.496 | 0.013 | 0.013 | 0.328 | 0.46020 |
| 10 | 114788815 | rs12243326 | T | C | -0.016 | 0.002 | 1.000e-200 | 1e-04 | 84.619 | 0.016 | 0.017 | 0.341 | 0.28530 |
| 4 | 100239319 | rs1229984 | T | C | -0.063 | 0.006 | 1.000e-200 | 2e-04 | 103.195 | -0.180 | 0.096 | 0.060 | 0.97120 |
| 2 | 216304384 | rs1250229 | T | C | 0.022 | 0.002 | 1.000e-200 | 2e-04 | 146.293 | -0.005 | 0.016 | 0.770 | 0.76840 |
| 8 | 110027347 | rs12543567 | T | C | 0.010 | 0.002 | 1.192e-10 | 1e-04 | 41.479 | 0.018 | 0.013 | 0.169 | 0.44930 |
| 9 | 139110654 | rs12684650 | T | C | 0.010 | 0.002 | 1.402e-08 | 0e+00 | 32.184 | -0.054 | 0.014 | 0.000 | 0.28730 |
| 7 | 25901639 | rs12700667 | A | G | -0.010 | 0.002 | 4.761e-09 | 0e+00 | 34.285 | -0.001 | 0.015 | 0.959 | 0.72270 |
| 1 | 208024820 | rs12731740 | T | C | -0.020 | 0.003 | 1.670e-15 | 1e-04 | 63.416 | -0.007 | 0.019 | 0.710 | 0.12030 |
| 1 | 38289383 | rs12751325 | T | C | -0.016 | 0.002 | 1.000e-200 | 1e-04 | 83.480 | 0.001 | 0.014 | 0.942 | 0.28430 |
| 21 | 47423509 | rs13051496 | T | C | -0.012 | 0.002 | 4.995e-08 | 0e+00 | 29.719 | 0.023 | 0.016 | 0.148 | 0.26040 |
| 4 | 77412140 | rs13146355 | A | G | -0.010 | 0.002 | 3.814e-10 | 1e-04 | 39.206 | 0.015 | 0.013 | 0.275 | 0.43240 |
| 6 | 12889004 | rs1332844 | T | C | -0.016 | 0.002 | 1.000e-200 | 1e-04 | 97.704 | 0.004 | 0.014 | 0.780 | 0.61630 |
| 9 | 22125503 | rs1333049 | C | G | 0.010 | 0.002 | 1.128e-09 | 0e+00 | 37.090 | 0.009 | 0.013 | 0.519 | 0.47220 |
| 6 | 7211818 | rs1334576 | A | G | -0.011 | 0.002 | 3.761e-12 | 1e-04 | 48.245 | -0.020 | 0.013 | 0.136 | 0.40760 |
| 2 | 20888265 | rs13385191 | A | G | 0.014 | 0.002 | 2.918e-14 | 1e-04 | 57.789 | -0.003 | 0.015 | 0.850 | 0.25050 |
| 2 | 165528876 | rs13389219 | T | C | -0.010 | 0.002 | 1.785e-10 | 0e+00 | 40.689 | 0.002 | 0.014 | 0.887 | 0.43540 |
| 12 | 66351826 | rs1351394 | T | C | -0.014 | 0.002 | 1.000e-200 | 1e-04 | 83.393 | 0.001 | 0.013 | 0.935 | 0.54970 |
| 17 | 6493198 | rs1443417 | T | C | -0.015 | 0.002 | 1.000e-200 | 1e-04 | 95.852 | -0.012 | 0.013 | 0.378 | 0.54770 |
| 10 | 63524591 | rs1530440 | T | C | -0.016 | 0.002 | 2.210e-15 | 1e-04 | 62.864 | -0.003 | 0.017 | 0.862 | 0.18990 |
| 3 | 85460131 | rs1549979 | T | C | 0.011 | 0.002 | 5.075e-11 | 1e-04 | 43.148 | -0.015 | 0.015 | 0.317 | 0.61930 |
| 5 | 156139569 | rs157350 | A | G | 0.015 | 0.002 | 4.269e-10 | 0e+00 | 38.986 | -0.060 | 0.023 | 0.008 | 0.86880 |
| 3 | 151899704 | rs161792 | A | T | 0.010 | 0.002 | 4.300e-09 | 0e+00 | 34.483 | -0.022 | 0.014 | 0.104 | 0.63120 |
| 5 | 122471989 | rs1644318 | T | C | -0.020 | 0.002 | 1.000e-200 | 2e-04 | 145.936 | -0.002 | 0.013 | 0.904 | 0.42350 |
| 2 | 96795608 | rs1657502 | T | G | 0.014 | 0.002 | 1.000e-200 | 1e-04 | 79.528 | 0.030 | 0.013 | 0.022 | 0.35590 |
| 19 | 11526765 | rs167479 | T | G | -0.018 | 0.002 | 1.000e-200 | 2e-04 | 132.179 | -0.012 | 0.013 | 0.376 | 0.46020 |
| 20 | 57758720 | rs16982520 | A | G | -0.020 | 0.002 | 1.000e-17 | 1e-04 | 73.210 | -0.042 | 0.018 | 0.017 | 0.12620 |
| 4 | 81184341 | rs16998073 | A | T | -0.024 | 0.002 | 1.000e-200 | 2e-04 | 201.480 | -0.010 | 0.014 | 0.479 | 0.26840 |
| 1 | 46493460 | rs1707336 | T | G | -0.010 | 0.002 | 6.022e-10 | 0e+00 | 38.314 | -0.031 | 0.013 | 0.022 | 0.44630 |
| 1 | 56962821 | rs17114036 | A | G | 0.023 | 0.003 | 1.000e-200 | 1e-04 | 75.379 | 0.025 | 0.021 | 0.247 | 0.12130 |
| 15 | 91416550 | rs17514846 | A | C | 0.020 | 0.002 | 1.000e-200 | 2e-04 | 159.484 | -0.016 | 0.014 | 0.224 | 0.46120 |
| 18 | 57851097 | rs17782313 | T | C | 0.011 | 0.002 | 1.123e-08 | 0e+00 | 32.617 | 0.023 | 0.017 | 0.180 | 0.23960 |
| 22 | 21928641 | rs181359 | A | G | -0.012 | 0.002 | 2.357e-10 | 1e-04 | 40.146 | -0.020 | 0.014 | 0.172 | 0.19380 |
| 20 | 10965998 | rs1887320 | A | G | 0.009 | 0.002 | 3.476e-09 | 0e+00 | 34.897 | 0.002 | 0.013 | 0.864 | 0.46120 |
| 6 | 56102780 | rs1925153 | T | C | -0.017 | 0.002 | 1.000e-200 | 1e-04 | 111.439 | 0.012 | 0.013 | 0.349 | 0.43240 |
| 7 | 19036775 | rs2023938 | T | C | -0.031 | 0.003 | 1.000e-200 | 2e-04 | 141.512 | -0.005 | 0.021 | 0.801 | 0.09543 |
| 10 | 75562108 | rs2075641 | A | G | 0.021 | 0.002 | 1.000e-200 | 1e-04 | 80.719 | -0.005 | 0.019 | 0.796 | 0.13420 |
| 9 | 101748356 | rs2075663 | A | G | 0.014 | 0.002 | 1.000e-200 | 1e-04 | 77.607 | -0.028 | 0.013 | 0.037 | 0.41550 |
| 14 | 54107791 | rs210381 | A | G | -0.011 | 0.002 | 4.534e-13 | 1e-04 | 52.397 | -0.024 | 0.013 | 0.067 | 0.54970 |
| 6 | 108988184 | rs2153960 | A | G | -0.011 | 0.002 | 1.581e-09 | 0e+00 | 36.432 | 0.004 | 0.014 | 0.788 | 0.64210 |
| 11 | 30394734 | rs2183848 | A | G | 0.014 | 0.002 | 2.300e-16 | 1e-04 | 67.305 | 0.003 | 0.014 | 0.817 | 0.29720 |
| 21 | 16339172 | rs2229742 | C | G | 0.016 | 0.003 | 5.956e-10 | 0e+00 | 38.336 | -0.029 | 0.021 | 0.175 | 0.09841 |
| 22 | 19969106 | rs2240717 | A | G | -0.017 | 0.002 | 1.000e-200 | 1e-04 | 106.076 | 0.021 | 0.014 | 0.114 | 0.34590 |
| 3 | 52584787 | rs2251219 | T | C | -0.014 | 0.002 | 1.000e-200 | 1e-04 | 77.217 | 0.009 | 0.013 | 0.504 | 0.41950 |
| 6 | 43270151 | rs2270860 | T | C | 0.016 | 0.002 | 1.000e-200 | 1e-04 | 85.362 | 0.032 | 0.014 | 0.025 | 0.34390 |
| 14 | 36153177 | rs2274068 | T | C | 0.011 | 0.002 | 2.167e-08 | 0e+00 | 31.339 | 0.018 | 0.016 | 0.257 | 0.19180 |
| 10 | 96039597 | rs2274224 | C | G | -0.016 | 0.002 | 1.000e-200 | 1e-04 | 79.667 | -0.016 | 0.014 | 0.247 | 0.45630 |
| 7 | 92264410 | rs2282978 | T | C | 0.027 | 0.002 | 1.000e-200 | 3e-04 | 271.248 | -0.003 | 0.014 | 0.844 | 0.35980 |
| 16 | 89613123 | rs2292954 | A | G | -0.012 | 0.002 | 1.272e-09 | 0e+00 | 36.856 | 0.003 | 0.022 | 0.893 | 0.16800 |
| 6 | 31632686 | rs2295665 | T | C | -0.027 | 0.002 | 1.000e-200 | 2e-04 | 127.487 | -0.020 | 0.015 | 0.158 | 0.16300 |
| 10 | 21074455 | rs2296613 | T | C | -0.011 | 0.002 | 5.057e-10 | 0e+00 | 38.655 | 0.040 | 0.016 | 0.015 | 0.26840 |
| 19 | 2226772 | rs2302061 | C | G | 0.023 | 0.002 | 1.000e-200 | 1e-04 | 93.135 | -0.006 | 0.017 | 0.700 | 0.14410 |
| 5 | 122682334 | rs2303720 | T | C | -0.042 | 0.005 | 1.000e-200 | 1e-04 | 75.075 | -0.004 | 0.027 | 0.871 | 0.02584 |
| 3 | 66434643 | rs2306272 | T | C | 0.015 | 0.002 | 1.000e-200 | 1e-04 | 75.959 | -0.003 | 0.015 | 0.862 | 0.29220 |
| 8 | 38095662 | rs2306899 | T | C | 0.012 | 0.002 | 1.251e-11 | 1e-04 | 45.890 | -0.014 | 0.017 | 0.416 | 0.25450 |
| 4 | 17972372 | rs2320299 | A | G | 0.010 | 0.002 | 5.721e-09 | 0e+00 | 33.927 | 0.008 | 0.014 | 0.552 | 0.70680 |
| 4 | 95496882 | rs2452600 | T | C | -0.011 | 0.002 | 1.813e-10 | 0e+00 | 40.658 | -0.029 | 0.015 | 0.050 | 0.31210 |
| 8 | 120353267 | rs2469997 | C | G | -0.044 | 0.002 | 1.000e-200 | 6e-04 | 445.876 | 0.020 | 0.020 | 0.317 | 0.85390 |
| 4 | 3451109 | rs2498323 | A | G | 0.025 | 0.003 | 1.000e-200 | 1e-04 | 88.087 | 0.012 | 0.027 | 0.644 | 0.08151 |
| 10 | 30335122 | rs2505083 | T | C | -0.016 | 0.002 | 1.000e-200 | 1e-04 | 108.403 | 0.000 | 0.013 | 0.984 | 0.42840 |
| 6 | 2355675 | rs2505675 | T | C | 0.009 | 0.002 | 4.910e-08 | 0e+00 | 29.752 | 0.020 | 0.014 | 0.160 | 0.33700 |
| 2 | 60608759 | rs2540917 | T | C | -0.011 | 0.002 | 1.455e-12 | 1e-04 | 50.108 | 0.025 | 0.014 | 0.063 | 0.41950 |
| 3 | 11400249 | rs2606736 | T | C | 0.017 | 0.002 | 1.000e-200 | 1e-04 | 106.058 | 0.016 | 0.013 | 0.247 | 0.58850 |
| 3 | 37574951 | rs267561 | A | G | -0.010 | 0.002 | 7.028e-11 | 1e-04 | 42.511 | -0.006 | 0.013 | 0.653 | 0.54270 |
| 12 | 90008959 | rs2681472 | A | G | 0.036 | 0.002 | 1.000e-200 | 4e-04 | 298.860 | 0.058 | 0.025 | 0.021 | 0.14510 |
| 2 | 217682779 | rs2712184 | A | C | 0.009 | 0.002 | 8.222e-09 | 0e+00 | 33.222 | -0.028 | 0.014 | 0.042 | 0.57160 |
| 21 | 30106926 | rs2832007 | A | G | 0.011 | 0.002 | 2.471e-10 | 0e+00 | 40.053 | -0.012 | 0.015 | 0.404 | 0.31210 |
| 16 | 75390316 | rs2865531 | A | T | 0.021 | 0.002 | 1.000e-200 | 2e-04 | 132.039 | -0.028 | 0.013 | 0.033 | 0.57360 |
| 14 | 100133942 | rs2895811 | T | C | -0.012 | 0.002 | 3.270e-15 | 1e-04 | 62.094 | 0.003 | 0.013 | 0.828 | 0.42640 |
| 17 | 76799860 | rs3088040 | T | C | 0.009 | 0.002 | 5.258e-09 | 0e+00 | 34.091 | 0.002 | 0.014 | 0.899 | 0.54770 |
| 7 | 100453208 | rs314370 | T | C | 0.021 | 0.002 | 1.000e-200 | 1e-04 | 114.210 | -0.005 | 0.016 | 0.745 | 0.18990 |
| 5 | 71519664 | rs3209157 | T | C | 0.013 | 0.002 | 3.400e-15 | 1e-04 | 62.017 | -0.021 | 0.014 | 0.117 | 0.40560 |
| 2 | 43625184 | rs33979934 | A | T | -0.011 | 0.002 | 2.751e-10 | 1e-04 | 39.844 | -0.002 | 0.015 | 0.895 | 0.27040 |
| 17 | 41004637 | rs33986943 | A | G | -0.023 | 0.003 | 1.000e-200 | 1e-04 | 81.070 | -0.009 | 0.026 | 0.726 | 0.11230 |
| 2 | 177016728 | rs34727427 | T | C | -0.016 | 0.002 | 1.000e-200 | 1e-04 | 84.933 | 0.016 | 0.015 | 0.298 | 0.30820 |
| 6 | 90397132 | rs34766278 | C | G | -0.014 | 0.002 | 2.800e-08 | 0e+00 | 30.842 | 0.006 | 0.019 | 0.756 | 0.15510 |
| 5 | 141309824 | rs351260 | A | G | 0.009 | 0.002 | 8.405e-09 | 0e+00 | 33.179 | -0.041 | 0.014 | 0.004 | 0.30620 |
| 2 | 203765756 | rs35212307 | T | C | -0.023 | 0.002 | 1.000e-200 | 1e-04 | 93.204 | -0.012 | 0.021 | 0.579 | 0.13020 |
| 12 | 115552437 | rs35444 | A | G | 0.015 | 0.002 | 1.000e-200 | 1e-04 | 93.078 | 0.007 | 0.014 | 0.604 | 0.39760 |
| 1 | 153662423 | rs35479618 | A | G | 0.045 | 0.006 | 5.538e-13 | 1e-04 | 52.004 | 0.050 | 0.066 | 0.453 | 0.01392 |
| 4 | 55139771 | rs35597368 | T | C | -0.016 | 0.003 | 8.409e-11 | 1e-04 | 42.160 | -0.003 | 0.020 | 0.877 | 0.10740 |
| 16 | 30936081 | rs35675346 | A | G | -0.012 | 0.002 | 9.592e-11 | 1e-04 | 41.903 | -0.018 | 0.015 | 0.223 | 0.25050 |
| 4 | 156289917 | rs3733391 | A | G | 0.012 | 0.002 | 5.250e-11 | 1e-04 | 43.082 | 0.030 | 0.014 | 0.036 | 0.24060 |
| 7 | 131195959 | rs3735035 | T | C | 0.009 | 0.002 | 1.933e-08 | 0e+00 | 31.561 | 0.009 | 0.013 | 0.498 | 0.43540 |
| 7 | 155531080 | rs3735576 | A | G | -0.012 | 0.002 | 3.981e-09 | 0e+00 | 34.633 | -0.008 | 0.017 | 0.618 | 0.18690 |
| 6 | 26505362 | rs3736781 | A | G | -0.011 | 0.002 | 4.225e-13 | 1e-04 | 52.536 | 0.040 | 0.013 | 0.003 | 0.44230 |
| 2 | 238672703 | rs3739038 | C | G | 0.011 | 0.002 | 1.341e-09 | 0e+00 | 36.753 | -0.012 | 0.015 | 0.433 | 0.24950 |
| 8 | 142222445 | rs3739238 | T | C | -0.011 | 0.002 | 1.753e-11 | 1e-04 | 45.229 | 0.003 | 0.014 | 0.849 | 0.58750 |
| 20 | 36841914 | rs3746471 | A | G | 0.010 | 0.002 | 2.577e-10 | 0e+00 | 39.972 | 0.016 | 0.013 | 0.242 | 0.42350 |
| 6 | 143823112 | rs3762001 | A | G | 0.012 | 0.002 | 4.955e-10 | 0e+00 | 38.695 | -0.022 | 0.015 | 0.135 | 0.22470 |
| 3 | 63982082 | rs3774729 | A | G | -0.010 | 0.002 | 1.672e-09 | 0e+00 | 36.323 | 0.005 | 0.014 | 0.711 | 0.33400 |
| 2 | 56111309 | rs3791675 | T | C | 0.012 | 0.002 | 2.332e-11 | 1e-04 | 44.670 | -0.001 | 0.016 | 0.931 | 0.23060 |
| 7 | 116186241 | rs3807989 | A | G | 0.013 | 0.002 | 5.000e-17 | 1e-04 | 70.033 | 0.001 | 0.013 | 0.962 | 0.60640 |
| 16 | 4432029 | rs3810818 | A | C | 0.012 | 0.002 | 4.738e-10 | 0e+00 | 38.782 | -0.004 | 0.015 | 0.806 | 0.77630 |
| 9 | 16915021 | rs3814113 | T | C | -0.011 | 0.002 | 1.848e-10 | 0e+00 | 40.622 | 0.007 | 0.014 | 0.599 | 0.30620 |
| 14 | 73422259 | rs3815460 | C | G | -0.020 | 0.003 | 1.308e-12 | 1e-04 | 50.318 | 0.001 | 0.027 | 0.972 | 0.11230 |
| 6 | 160745751 | rs384156 | A | C | 0.009 | 0.002 | 4.567e-08 | 0e+00 | 29.892 | 0.005 | 0.014 | 0.741 | 0.65710 |
| 13 | 47243196 | rs41284185 | C | G | 0.016 | 0.003 | 1.837e-08 | 0e+00 | 31.659 | 0.045 | 0.024 | 0.056 | 0.05964 |
| 6 | 31917540 | rs4151657 | T | C | -0.016 | 0.002 | 1.000e-200 | 1e-04 | 82.306 | 0.002 | 0.014 | 0.904 | 0.36480 |
| 6 | 159749112 | rs4388308 | T | C | -0.015 | 0.002 | 1.000e-200 | 1e-04 | 81.754 | -0.012 | 0.014 | 0.396 | 0.69280 |
| 18 | 3457606 | rs4468717 | T | C | -0.017 | 0.003 | 1.633e-09 | 0e+00 | 36.369 | -0.018 | 0.019 | 0.360 | 0.09443 |
| 3 | 169100899 | rs448378 | A | G | -0.011 | 0.002 | 1.264e-12 | 1e-04 | 50.384 | 0.002 | 0.013 | 0.870 | 0.51790 |
| 12 | 33510115 | rs4587807 | A | G | 0.010 | 0.002 | 2.738e-09 | 0e+00 | 35.362 | 0.021 | 0.014 | 0.113 | 0.53480 |
| 15 | 63359564 | rs4619348 | A | C | 0.012 | 0.002 | 9.620e-15 | 1e-04 | 59.971 | 0.024 | 0.013 | 0.072 | 0.57850 |
| 22 | 40729614 | rs470113 | A | G | -0.022 | 0.002 | 1.000e-200 | 2e-04 | 123.586 | -0.016 | 0.016 | 0.322 | 0.15610 |
| 22 | 29451671 | rs4823006 | A | G | 0.009 | 0.002 | 1.409e-08 | 0e+00 | 32.174 | -0.028 | 0.013 | 0.038 | 0.42740 |
| 1 | 219750717 | rs4846567 | T | G | -0.017 | 0.002 | 1.000e-200 | 1e-04 | 94.671 | 0.024 | 0.015 | 0.105 | 0.28630 |
| 11 | 117222592 | rs490262 | A | G | 0.019 | 0.002 | 1.000e-200 | 1e-04 | 96.767 | -0.017 | 0.019 | 0.346 | 0.17400 |
| 11 | 57381989 | rs4926 | A | G | -0.015 | 0.002 | 6.296e-14 | 1e-04 | 56.277 | 0.004 | 0.014 | 0.796 | 0.26640 |
| 3 | 27416013 | rs4973768 | T | C | 0.011 | 0.002 | 4.955e-12 | 1e-04 | 47.705 | 0.012 | 0.013 | 0.390 | 0.48110 |
| 5 | 158258461 | rs57392066 | T | C | 0.019 | 0.002 | 1.000e-200 | 2e-04 | 142.286 | 0.010 | 0.014 | 0.457 | 0.59740 |
| 20 | 33907161 | rs6060369 | T | C | -0.012 | 0.002 | 3.234e-14 | 1e-04 | 57.587 | 0.002 | 0.013 | 0.876 | 0.38470 |
| 20 | 62695931 | rs6062343 | A | G | -0.010 | 0.002 | 6.032e-11 | 1e-04 | 42.810 | 0.004 | 0.013 | 0.770 | 0.43540 |
| 20 | 10603750 | rs6077853 | A | G | 0.024 | 0.002 | 1.000e-200 | 2e-04 | 129.063 | 0.011 | 0.021 | 0.583 | 0.13220 |
| 1 | 1226889 | rs609805 | A | G | -0.030 | 0.005 | 1.130e-09 | 1e-04 | 37.087 | 0.016 | 0.028 | 0.565 | 0.03777 |
| 18 | 34289285 | rs61735998 | T | G | 0.034 | 0.005 | 7.928e-11 | 1e-04 | 42.276 | 0.013 | 0.044 | 0.762 | 0.02286 |
| 3 | 41756965 | rs61744388 | T | C | -0.043 | 0.002 | 1.000e-200 | 5e-04 | 407.336 | -0.001 | 0.016 | 0.973 | 0.18390 |
| 17 | 1372839 | rs61753655 | T | C | 0.067 | 0.008 | 1.000e-200 | 1e-04 | 77.449 | -0.021 | 0.062 | 0.742 | 0.01093 |
| 16 | 72830539 | rs62051555 | C | G | -0.039 | 0.004 | 1.000e-200 | 1e-04 | 108.903 | 0.022 | 0.027 | 0.413 | 0.04274 |
| 5 | 53298025 | rs6450176 | A | G | 0.013 | 0.002 | 3.389e-12 | 1e-04 | 48.449 | -0.031 | 0.015 | 0.042 | 0.28130 |
| 17 | 62408299 | rs6504218 | A | G | -0.012 | 0.002 | 5.340e-15 | 1e-04 | 61.130 | -0.007 | 0.013 | 0.574 | 0.56460 |
| 1 | 116278503 | rs6677074 | A | C | 0.011 | 0.002 | 6.558e-12 | 1e-04 | 47.155 | 0.026 | 0.013 | 0.056 | 0.43540 |
| 1 | 89123443 | rs6699417 | T | C | -0.015 | 0.002 | 1.000e-200 | 1e-04 | 87.913 | 0.015 | 0.014 | 0.289 | 0.62330 |
| 2 | 33361425 | rs6714546 | A | G | -0.011 | 0.002 | 6.110e-10 | 0e+00 | 38.286 | -0.020 | 0.016 | 0.197 | 0.70680 |
| 2 | 71633389 | rs6714975 | T | C | -0.012 | 0.002 | 2.881e-14 | 1e-04 | 57.814 | -0.001 | 0.013 | 0.957 | 0.55270 |
| 1 | 230845794 | rs699 | A | G | -0.010 | 0.002 | 6.033e-11 | 1e-04 | 42.810 | -0.001 | 0.013 | 0.962 | 0.41150 |
| 10 | 12277992 | rs7068966 | T | C | -0.010 | 0.002 | 6.836e-10 | 0e+00 | 38.067 | -0.003 | 0.013 | 0.836 | 0.50600 |
| 10 | 115789375 | rs7076938 | T | C | 0.012 | 0.002 | 5.942e-12 | 1e-04 | 47.349 | -0.006 | 0.015 | 0.696 | 0.67890 |
| 14 | 100625902 | rs7157599 | T | C | -0.012 | 0.002 | 6.095e-11 | 1e-04 | 42.790 | 0.006 | 0.015 | 0.674 | 0.69480 |
| 2 | 164731394 | rs717366 | T | C | 0.014 | 0.002 | 1.000e-200 | 1e-04 | 74.311 | -0.010 | 0.013 | 0.464 | 0.37280 |
| 15 | 79141784 | rs7173743 | T | C | 0.017 | 0.002 | 1.000e-200 | 1e-04 | 95.526 | -0.008 | 0.014 | 0.587 | 0.46520 |
| 16 | 58622178 | rs7188697 | A | G | -0.013 | 0.002 | 2.198e-13 | 1e-04 | 53.819 | -0.009 | 0.015 | 0.541 | 0.75750 |
| 17 | 43101382 | rs7225329 | T | C | -0.018 | 0.002 | 1.000e-200 | 2e-04 | 123.095 | -0.008 | 0.013 | 0.529 | 0.57460 |
| 19 | 7224431 | rs7248104 | A | G | -0.018 | 0.002 | 1.000e-200 | 2e-04 | 129.154 | 0.005 | 0.013 | 0.705 | 0.42150 |
| 19 | 31863085 | rs7248171 | A | G | 0.011 | 0.002 | 1.006e-12 | 1e-04 | 50.833 | -0.022 | 0.013 | 0.091 | 0.34490 |
| 12 | 115891403 | rs7315438 | T | C | 0.009 | 0.002 | 1.871e-08 | 0e+00 | 31.624 | 0.010 | 0.014 | 0.496 | 0.55270 |
| 15 | 67702907 | rs7359257 | A | C | 0.010 | 0.002 | 7.397e-10 | 0e+00 | 37.913 | -0.004 | 0.014 | 0.755 | 0.53580 |
| 19 | 45412079 | rs7412 | T | C | -0.030 | 0.003 | 1.000e-200 | 1e-04 | 91.934 | -0.026 | 0.030 | 0.373 | 0.06262 |
| 12 | 94769769 | rs74340001 | A | G | -0.027 | 0.003 | 1.000e-200 | 1e-04 | 88.432 | -0.010 | 0.029 | 0.745 | 0.06660 |
| 1 | 234726012 | rs744487 | A | C | 0.010 | 0.002 | 1.967e-09 | 0e+00 | 36.006 | -0.024 | 0.014 | 0.088 | 0.31910 |
| 7 | 139415775 | rs7456421 | C | G | -0.013 | 0.002 | 1.033e-10 | 1e-04 | 41.757 | -0.004 | 0.016 | 0.817 | 0.21270 |
| 2 | 24247514 | rs7561273 | A | G | 0.009 | 0.002 | 4.030e-08 | 0e+00 | 30.135 | -0.005 | 0.013 | 0.695 | 0.52090 |
| 11 | 17351683 | rs757081 | C | G | -0.017 | 0.002 | 1.000e-200 | 1e-04 | 105.722 | 0.002 | 0.014 | 0.911 | 0.31110 |
| 6 | 32830013 | rs7767288 | A | G | 0.010 | 0.002 | 1.884e-10 | 1e-04 | 40.584 | 0.030 | 0.014 | 0.028 | 0.39860 |
| 8 | 11045161 | rs7819412 | A | G | -0.012 | 0.002 | 5.790e-15 | 1e-04 | 60.971 | -0.015 | 0.014 | 0.283 | 0.51190 |
| 9 | 85126163 | rs7866939 | T | C | -0.009 | 0.002 | 2.654e-08 | 0e+00 | 30.945 | 0.001 | 0.014 | 0.951 | 0.34390 |
| 9 | 73080367 | rs7873409 | T | G | 0.009 | 0.002 | 1.400e-08 | 0e+00 | 32.187 | 0.016 | 0.013 | 0.221 | 0.56760 |
| 4 | 48493237 | rs79858408 | A | G | -0.012 | 0.002 | 1.920e-14 | 1e-04 | 58.613 | -0.010 | 0.013 | 0.434 | 0.51090 |
| 5 | 140115303 | rs801171 | A | G | 0.012 | 0.002 | 1.214e-13 | 1e-04 | 54.986 | -0.016 | 0.013 | 0.219 | 0.43040 |
| 17 | 59483766 | rs8068318 | T | C | 0.017 | 0.002 | 1.000e-200 | 1e-04 | 94.885 | 0.027 | 0.015 | 0.064 | 0.72760 |
| 19 | 41038574 | rs814501 | A | G | 0.010 | 0.002 | 1.519e-10 | 0e+00 | 41.005 | 0.010 | 0.013 | 0.451 | 0.47220 |
| 7 | 73483689 | rs8326 | C | G | -0.014 | 0.002 | 4.088e-09 | 0e+00 | 34.581 | -0.001 | 0.022 | 0.981 | 0.11230 |
| 1 | 59787350 | rs835409 | T | G | 0.009 | 0.002 | 9.086e-09 | 0e+00 | 33.028 | -0.005 | 0.014 | 0.720 | 0.35090 |
| 1 | 10796866 | rs880315 | T | C | -0.016 | 0.002 | 1.000e-200 | 1e-04 | 98.065 | -0.015 | 0.013 | 0.263 | 0.35690 |
| 9 | 123652898 | rs881375 | T | C | 0.014 | 0.002 | 1.000e-200 | 1e-04 | 78.326 | -0.001 | 0.013 | 0.928 | 0.63920 |
| 3 | 156798732 | rs900399 | A | G | 0.009 | 0.002 | 2.448e-08 | 0e+00 | 31.102 | -0.012 | 0.014 | 0.406 | 0.38970 |
| 11 | 1874072 | rs907611 | A | G | 0.016 | 0.002 | 1.000e-200 | 1e-04 | 78.840 | -0.001 | 0.014 | 0.920 | 0.32700 |
| 11 | 1941946 | rs909116 | T | C | -0.017 | 0.002 | 1.000e-200 | 1e-04 | 122.252 | -0.012 | 0.013 | 0.383 | 0.45830 |
| 20 | 42826350 | rs9346 | A | C | -0.017 | 0.002 | 1.390e-13 | 1e-04 | 54.720 | 0.010 | 0.017 | 0.542 | 0.15310 |
| 8 | 76776862 | rs969826 | A | G | -0.012 | 0.002 | 3.050e-15 | 1e-04 | 62.229 | -0.001 | 0.013 | 0.922 | 0.48210 |
| 11 | 103660567 | rs974819 | T | C | 0.010 | 0.002 | 1.738e-08 | 0e+00 | 31.767 | 0.030 | 0.016 | 0.059 | 0.72070 |
| 8 | 92091710 | rs9918929 | T | C | 0.009 | 0.002 | 6.050e-09 | 0e+00 | 33.819 | -0.021 | 0.013 | 0.118 | 0.59740 |
| 21 | 35599128 | rs9982601 | T | C | -0.016 | 0.002 | 3.882e-12 | 1e-04 | 48.183 | 0.021 | 0.019 | 0.280 | 0.12230 |

**Supplementary table 2.10. Detailed information of instrumental SNPs for Systolic blood pressure** **in the analysis of PPD.**

| **Chr** | **Position** | **SNP** | **EA** | **OA** | **beta.exposure** | **se.exposure** | **pval.exposure** | **R2** | **F_val** | **beta.outcome** | **se.outcome** | **pval.outcome** | **Eaf.outcome** |
| --- | --- | --- | --- | --- | --- | --- | --- | --- | --- | --- | --- | --- | --- |
| 4 | 2195024 | rs10018786 | T | G | -0.018 | 0.003 | 8.399e-12 | 1e-04 | 46.670 | -0.002 | 0.023 | 0.914 | 0.12430 |
| 3 | 50597092 | rs1034405 | A | G | -0.013 | 0.002 | 2.180e-08 | 0e+00 | 31.328 | -0.053 | 0.019 | 0.006 | 0.88470 |
| 10 | 124214251 | rs10490923 | A | G | 0.017 | 0.002 | 2.729e-12 | 1e-04 | 48.874 | -0.022 | 0.023 | 0.345 | 0.13020 |
| 16 | 4942099 | rs1049205 | T | C | 0.016 | 0.002 | 1.000e-200 | 1e-04 | 98.244 | 0.005 | 0.014 | 0.691 | 0.53880 |
| 13 | 27879526 | rs10507380 | A | G | 0.017 | 0.002 | 7.399e-13 | 1e-04 | 51.435 | 0.006 | 0.016 | 0.701 | 0.15010 |
| 17 | 45018463 | rs1052586 | T | C | 0.018 | 0.002 | 1.000e-200 | 2e-04 | 117.888 | 0.028 | 0.013 | 0.035 | 0.48910 |
| 12 | 20230639 | rs10770612 | A | G | 0.015 | 0.002 | 5.340e-13 | 1e-04 | 52.076 | 0.005 | 0.016 | 0.756 | 0.17590 |
| 10 | 123033549 | rs10788160 | A | G | 0.012 | 0.002 | 2.261e-11 | 1e-04 | 44.730 | 0.016 | 0.014 | 0.276 | 0.26140 |
| 18 | 57851763 | rs10871777 | A | G | 0.011 | 0.002 | 1.538e-09 | 0e+00 | 36.486 | 0.023 | 0.017 | 0.181 | 0.24650 |
| 12 | 50901882 | rs10876041 | T | C | 0.016 | 0.002 | 1.000e-200 | 1e-04 | 90.585 | 0.015 | 0.014 | 0.279 | 0.59940 |
| 1 | 169099483 | rs10919071 | A | G | -0.018 | 0.002 | 7.526e-14 | 1e-04 | 55.926 | -0.016 | 0.021 | 0.467 | 0.13020 |
| 3 | 169492101 | rs10936599 | T | C | -0.013 | 0.002 | 5.535e-12 | 1e-04 | 47.488 | 0.008 | 0.015 | 0.571 | 0.24250 |
| 6 | 79655477 | rs10943605 | A | G | 0.014 | 0.002 | 1.000e-200 | 1e-04 | 79.239 | -0.008 | 0.013 | 0.567 | 0.47910 |
| 10 | 64564934 | rs10995311 | C | G | 0.016 | 0.002 | 1.000e-200 | 1e-04 | 103.571 | 0.004 | 0.014 | 0.767 | 0.41450 |
| 10 | 75902177 | rs11000889 | A | G | -0.010 | 0.002 | 7.219e-11 | 1e-04 | 42.459 | -0.015 | 0.013 | 0.265 | 0.62520 |
| 10 | 18708798 | rs11014166 | A | T | 0.029 | 0.002 | 1.000e-200 | 4e-04 | 303.963 | 0.006 | 0.015 | 0.691 | 0.32210 |
| 12 | 427575 | rs11062385 | A | G | -0.010 | 0.002 | 3.250e-09 | 0e+00 | 35.028 | -0.005 | 0.014 | 0.748 | 0.23260 |
| 9 | 113169775 | rs111245230 | T | C | -0.041 | 0.004 | 1.000e-200 | 1e-04 | 89.897 | 0.029 | 0.061 | 0.635 | 0.03181 |
| 9 | 116191205 | rs111841972 | A | C | 0.019 | 0.003 | 3.793e-08 | 0e+00 | 30.253 | -0.038 | 0.030 | 0.210 | 0.05169 |
| 11 | 58207203 | rs11229457 | T | C | -0.016 | 0.002 | 5.000e-17 | 1e-04 | 70.128 | 0.006 | 0.015 | 0.693 | 0.20970 |
| 12 | 49399132 | rs1126930 | C | G | 0.037 | 0.004 | 1.000e-200 | 1e-04 | 75.677 | -0.017 | 0.038 | 0.652 | 0.02386 |
| 9 | 127975654 | rs1128362 | T | C | 0.018 | 0.002 | 5.700e-16 | 1e-04 | 65.528 | -0.012 | 0.020 | 0.560 | 0.13620 |
| 19 | 17170885 | rs1130222 | T | C | 0.009 | 0.002 | 3.697e-09 | 0e+00 | 34.777 | -0.006 | 0.013 | 0.662 | 0.39960 |
| 10 | 134459388 | rs1133400 | A | G | -0.015 | 0.002 | 1.477e-14 | 1e-04 | 59.129 | 0.011 | 0.018 | 0.525 | 0.21370 |
| 17 | 73949540 | rs1135640 | C | G | 0.012 | 0.002 | 5.161e-13 | 1e-04 | 52.143 | 0.005 | 0.015 | 0.736 | 0.64020 |
| 1 | 42657196 | rs1139978 | T | G | 0.013 | 0.002 | 1.531e-08 | 0e+00 | 32.014 | -0.001 | 0.019 | 0.969 | 0.12620 |
| 11 | 100470306 | rs1145415 | A | G | 0.016 | 0.002 | 1.000e-200 | 1e-04 | 109.025 | 0.009 | 0.013 | 0.488 | 0.45130 |
| 15 | 41245289 | rs11632640 | T | C | -0.012 | 0.002 | 1.899e-14 | 1e-04 | 58.634 | -0.019 | 0.013 | 0.149 | 0.45530 |
| 7 | 75163169 | rs1167827 | A | G | -0.010 | 0.002 | 4.933e-09 | 1e-04 | 34.216 | -0.013 | 0.013 | 0.325 | 0.53980 |
| 7 | 46008110 | rs11977526 | A | G | -0.019 | 0.002 | 1.000e-200 | 2e-04 | 144.393 | -0.008 | 0.014 | 0.596 | 0.40260 |
| 1 | 16042766 | rs12091750 | T | C | 0.017 | 0.002 | 1.000e-200 | 1e-04 | 90.587 | -0.008 | 0.015 | 0.601 | 0.24450 |
| 11 | 55999950 | rs12221615 | C | G | -0.023 | 0.002 | 1.000e-200 | 1e-04 | 87.997 | -0.009 | 0.017 | 0.621 | 0.11730 |
| 4 | 100239319 | rs1229984 | T | C | -0.048 | 0.006 | 9.000e-15 | 1e-04 | 60.102 | -0.180 | 0.096 | 0.060 | 0.97120 |
| 13 | 51106555 | rs1239947 | T | C | 0.009 | 0.002 | 2.448e-08 | 0e+00 | 31.103 | 0.005 | 0.015 | 0.732 | 0.65110 |
| 12 | 133069894 | rs12423664 | A | G | 0.014 | 0.002 | 1.357e-10 | 1e-04 | 41.225 | -0.017 | 0.020 | 0.382 | 0.13720 |
| 3 | 153839866 | rs12493885 | C | G | -0.016 | 0.002 | 1.630e-12 | 1e-04 | 49.885 | 0.002 | 0.017 | 0.902 | 0.87380 |
| 2 | 216304384 | rs1250229 | T | C | 0.015 | 0.002 | 2.500e-16 | 1e-04 | 67.147 | -0.005 | 0.016 | 0.770 | 0.76840 |
| 18 | 26719357 | rs12605558 | A | G | 0.010 | 0.002 | 2.763e-09 | 0e+00 | 35.344 | 0.004 | 0.013 | 0.772 | 0.60830 |
| 4 | 107279482 | rs12645490 | A | T | 0.014 | 0.002 | 2.876e-09 | 1e-04 | 35.266 | 0.017 | 0.017 | 0.309 | 0.17690 |
| 6 | 31105310 | rs1265100 | A | G | -0.013 | 0.002 | 1.214e-08 | 0e+00 | 32.465 | 0.022 | 0.017 | 0.188 | 0.16600 |
| 7 | 131195712 | rs12670788 | A | G | 0.011 | 0.002 | 4.434e-12 | 1e-04 | 47.923 | 0.008 | 0.013 | 0.531 | 0.43240 |
| 11 | 65391317 | rs12801636 | A | G | -0.021 | 0.002 | 1.000e-200 | 1e-04 | 120.083 | -0.013 | 0.016 | 0.424 | 0.22370 |
| 16 | 20367690 | rs12917707 | T | G | -0.018 | 0.002 | 1.000e-200 | 1e-04 | 82.012 | 0.012 | 0.016 | 0.467 | 0.20080 |
| 4 | 103188709 | rs13107325 | T | C | -0.046 | 0.003 | 1.000e-200 | 3e-04 | 221.559 | 0.122 | 0.058 | 0.035 | 0.07952 |
| 2 | 165528876 | rs13389219 | T | C | -0.014 | 0.002 | 1.000e-17 | 1e-04 | 72.770 | 0.002 | 0.014 | 0.887 | 0.43540 |
| 12 | 66351826 | rs1351394 | T | C | -0.012 | 0.002 | 2.819e-14 | 1e-04 | 57.857 | 0.001 | 0.013 | 0.935 | 0.54970 |
| 4 | 96075965 | rs1434536 | T | C | -0.009 | 0.002 | 3.028e-08 | 0e+00 | 30.690 | -0.009 | 0.013 | 0.481 | 0.55860 |
| 4 | 111445587 | rs1448808 | A | G | 0.011 | 0.002 | 2.391e-12 | 1e-04 | 49.133 | -0.001 | 0.014 | 0.969 | 0.53180 |
| 10 | 63524591 | rs1530440 | T | C | -0.031 | 0.002 | 1.000e-200 | 3e-04 | 237.211 | -0.003 | 0.017 | 0.862 | 0.18990 |
| 3 | 85460131 | rs1549979 | T | C | 0.014 | 0.002 | 1.000e-17 | 1e-04 | 73.251 | -0.015 | 0.015 | 0.317 | 0.61930 |
| 5 | 122471989 | rs1644318 | T | C | -0.019 | 0.002 | 1.000e-200 | 2e-04 | 138.605 | -0.002 | 0.013 | 0.904 | 0.42350 |
| 19 | 11526765 | rs167479 | T | G | -0.031 | 0.002 | 1.000e-200 | 5e-04 | 390.749 | -0.012 | 0.013 | 0.376 | 0.46020 |
| 3 | 141134818 | rs16851397 | A | G | -0.029 | 0.004 | 2.970e-15 | 1e-04 | 62.283 | 0.057 | 0.037 | 0.121 | 0.04573 |
| 17 | 7633692 | rs16956936 | T | C | -0.015 | 0.002 | 1.687e-10 | 1e-04 | 40.799 | -0.004 | 0.023 | 0.849 | 0.11830 |
| 20 | 57758720 | rs16982520 | A | G | -0.040 | 0.002 | 1.000e-200 | 3e-04 | 280.836 | -0.042 | 0.018 | 0.017 | 0.12620 |
| 4 | 81184341 | rs16998073 | A | T | -0.041 | 0.002 | 1.000e-200 | 7e-04 | 580.508 | -0.010 | 0.014 | 0.479 | 0.26840 |
| 10 | 104591393 | rs17115100 | T | G | -0.053 | 0.003 | 1.000e-200 | 5e-04 | 363.819 | -0.025 | 0.023 | 0.277 | 0.09344 |
| 12 | 90060586 | rs17249754 | A | G | -0.046 | 0.002 | 1.000e-200 | 6e-04 | 473.852 | -0.057 | 0.025 | 0.023 | 0.14410 |
| 15 | 91416550 | rs17514846 | A | C | 0.030 | 0.002 | 1.000e-200 | 4e-04 | 347.959 | -0.016 | 0.014 | 0.224 | 0.46120 |
| 3 | 71020490 | rs17651978 | A | G | -0.011 | 0.002 | 4.785e-09 | 0e+00 | 34.275 | 0.024 | 0.016 | 0.131 | 0.25050 |
| 15 | 42032383 | rs17677991 | C | G | -0.009 | 0.002 | 4.731e-08 | 0e+00 | 29.824 | -0.027 | 0.014 | 0.044 | 0.37970 |
| 12 | 122396395 | rs17852561 | T | C | 0.010 | 0.002 | 4.772e-09 | 0e+00 | 34.280 | -0.014 | 0.014 | 0.300 | 0.31110 |
| 19 | 46181392 | rs1800437 | C | G | 0.012 | 0.002 | 2.518e-09 | 0e+00 | 35.525 | 0.004 | 0.015 | 0.780 | 0.21370 |
| 17 | 2203025 | rs1885987 | T | G | 0.013 | 0.002 | 1.520e-15 | 1e-04 | 63.594 | -0.029 | 0.014 | 0.031 | 0.38270 |
| 20 | 10965998 | rs1887320 | A | G | 0.024 | 0.002 | 1.000e-200 | 3e-04 | 229.945 | 0.002 | 0.013 | 0.864 | 0.46120 |
| 9 | 123640500 | rs1953126 | T | C | 0.010 | 0.002 | 8.859e-09 | 0e+00 | 33.077 | 0.000 | 0.013 | 0.975 | 0.64020 |
| 6 | 143200547 | rs198683 | T | C | -0.011 | 0.002 | 7.555e-12 | 1e-04 | 46.878 | -0.012 | 0.013 | 0.367 | 0.56060 |
| 7 | 19036775 | rs2023938 | T | C | -0.019 | 0.003 | 7.379e-13 | 1e-04 | 51.441 | -0.005 | 0.021 | 0.801 | 0.09543 |
| 2 | 68385097 | rs2044693 | A | G | -0.010 | 0.002 | 7.437e-10 | 0e+00 | 37.902 | -0.013 | 0.014 | 0.376 | 0.62230 |
| 15 | 86122779 | rs2061822 | T | C | -0.011 | 0.002 | 3.465e-11 | 1e-04 | 43.895 | -0.002 | 0.013 | 0.862 | 0.65110 |
| 11 | 130281488 | rs2131535 | A | G | -0.011 | 0.002 | 2.215e-09 | 0e+00 | 35.774 | -0.021 | 0.015 | 0.156 | 0.76940 |
| 6 | 108988184 | rs2153960 | A | G | -0.012 | 0.002 | 2.330e-11 | 1e-04 | 44.671 | 0.004 | 0.014 | 0.788 | 0.64210 |
| 19 | 19329924 | rs2228603 | T | C | 0.017 | 0.003 | 4.092e-09 | 0e+00 | 34.579 | -0.017 | 0.026 | 0.525 | 0.06759 |
| 21 | 16339172 | rs2229742 | C | G | 0.018 | 0.003 | 4.004e-12 | 1e-04 | 48.123 | -0.029 | 0.021 | 0.175 | 0.09841 |
| 22 | 19969106 | rs2240717 | A | G | -0.012 | 0.002 | 5.878e-14 | 1e-04 | 56.412 | 0.021 | 0.014 | 0.114 | 0.34590 |
| 6 | 43273604 | rs2242416 | A | G | 0.014 | 0.002 | 1.000e-200 | 1e-04 | 78.171 | 0.015 | 0.014 | 0.293 | 0.57650 |
| 2 | 145801461 | rs2252641 | T | C | 0.011 | 0.002 | 7.941e-12 | 1e-04 | 46.780 | -0.006 | 0.014 | 0.641 | 0.46020 |
| 5 | 32786389 | rs2270915 | A | G | -0.019 | 0.002 | 1.000e-200 | 1e-04 | 104.218 | 0.017 | 0.016 | 0.296 | 0.18790 |
| 14 | 31381351 | rs2273171 | T | C | -0.010 | 0.002 | 1.860e-10 | 0e+00 | 40.609 | -0.007 | 0.013 | 0.579 | 0.46320 |
| 16 | 2070568 | rs2286472 | A | C | -0.013 | 0.002 | 1.272e-13 | 1e-04 | 54.894 | -0.003 | 0.015 | 0.824 | 0.32900 |
| 19 | 49228272 | rs2287921 | T | C | -0.010 | 0.002 | 4.888e-09 | 1e-04 | 34.233 | -0.016 | 0.013 | 0.221 | 0.48110 |
| 19 | 2226772 | rs2302061 | C | G | 0.016 | 0.002 | 3.398e-12 | 1e-04 | 48.444 | -0.006 | 0.017 | 0.700 | 0.14410 |
| 3 | 66434643 | rs2306272 | T | C | 0.011 | 0.002 | 8.168e-11 | 1e-04 | 42.217 | -0.003 | 0.015 | 0.862 | 0.29220 |
| 8 | 38095662 | rs2306899 | T | C | 0.016 | 0.002 | 1.000e-17 | 1e-04 | 73.237 | -0.014 | 0.017 | 0.416 | 0.25450 |
| 4 | 17972372 | rs2320299 | A | G | 0.014 | 0.002 | 4.450e-15 | 1e-04 | 61.488 | 0.008 | 0.014 | 0.552 | 0.70680 |
| 8 | 120353267 | rs2469997 | C | G | -0.019 | 0.002 | 1.000e-200 | 1e-04 | 85.080 | 0.020 | 0.020 | 0.317 | 0.85390 |
| 1 | 3328659 | rs2493292 | T | C | 0.022 | 0.002 | 1.000e-200 | 1e-04 | 98.366 | -0.006 | 0.021 | 0.783 | 0.16600 |
| 4 | 3451109 | rs2498323 | A | G | 0.017 | 0.003 | 1.282e-10 | 1e-04 | 41.336 | 0.012 | 0.027 | 0.644 | 0.08151 |
| 1 | 27709020 | rs2504779 | T | C | 0.017 | 0.003 | 3.151e-10 | 0e+00 | 39.578 | -0.033 | 0.019 | 0.085 | 0.13620 |
| 1 | 228126470 | rs2527614 | A | G | 0.013 | 0.002 | 3.000e-17 | 1e-04 | 71.156 | -0.007 | 0.013 | 0.606 | 0.47510 |
| 3 | 11400249 | rs2606736 | T | C | 0.018 | 0.002 | 1.000e-200 | 1e-04 | 119.644 | 0.016 | 0.013 | 0.247 | 0.58850 |
| 3 | 183476685 | rs262993 | A | G | -0.009 | 0.002 | 4.767e-08 | 0e+00 | 29.809 | -0.004 | 0.014 | 0.755 | 0.45330 |
| 5 | 96139250 | rs26653 | C | G | -0.012 | 0.002 | 5.708e-10 | 1e-04 | 38.419 | -0.007 | 0.015 | 0.614 | 0.71770 |
| 3 | 37574951 | rs267561 | A | G | -0.012 | 0.002 | 5.738e-14 | 1e-04 | 56.459 | -0.006 | 0.013 | 0.653 | 0.54270 |
| 2 | 86135154 | rs2679722 | A | G | 0.009 | 0.002 | 3.941e-08 | 0e+00 | 30.178 | 0.000 | 0.013 | 0.986 | 0.61530 |
| 17 | 20107773 | rs2703805 | T | C | 0.009 | 0.002 | 4.981e-08 | 0e+00 | 29.724 | -0.012 | 0.013 | 0.375 | 0.39960 |
| 1 | 43886494 | rs2782643 | T | C | 0.012 | 0.002 | 2.807e-14 | 1e-04 | 57.865 | -0.004 | 0.014 | 0.755 | 0.42050 |
| 2 | 231248261 | rs28497362 | T | C | -0.013 | 0.002 | 3.777e-08 | 0e+00 | 30.260 | -0.012 | 0.018 | 0.509 | 0.15510 |
| 5 | 1288547 | rs2853676 | T | C | 0.010 | 0.002 | 3.942e-09 | 0e+00 | 34.652 | 0.008 | 0.016 | 0.606 | 0.72470 |
| 3 | 14899778 | rs294636 | T | G | -0.017 | 0.002 | 3.000e-16 | 1e-04 | 66.794 | 0.009 | 0.019 | 0.650 | 0.80520 |
| 17 | 76799860 | rs3088040 | T | C | 0.011 | 0.002 | 1.565e-11 | 1e-04 | 45.451 | 0.002 | 0.014 | 0.899 | 0.54770 |
| 5 | 71519664 | rs3209157 | T | C | 0.009 | 0.002 | 7.306e-09 | 0e+00 | 33.452 | -0.021 | 0.014 | 0.117 | 0.40560 |
| 16 | 20810067 | rs3213646 | T | C | 0.009 | 0.002 | 8.477e-09 | 0e+00 | 33.162 | -0.011 | 0.013 | 0.412 | 0.58050 |
| 10 | 75406912 | rs34163229 | T | G | 0.023 | 0.002 | 1.000e-200 | 1e-04 | 102.535 | -0.003 | 0.019 | 0.868 | 0.13320 |
| 15 | 63414083 | rs34317102 | A | C | 0.014 | 0.002 | 8.524e-14 | 1e-04 | 55.681 | 0.007 | 0.015 | 0.622 | 0.73160 |
| 8 | 142367087 | rs34591516 | T | C | 0.026 | 0.004 | 5.164e-13 | 1e-04 | 52.142 | -0.075 | 0.028 | 0.008 | 0.05169 |
| 2 | 177016728 | rs34727427 | T | C | -0.016 | 0.002 | 1.000e-200 | 1e-04 | 90.755 | 0.016 | 0.015 | 0.298 | 0.30820 |
| 12 | 115552437 | rs35444 | A | G | 0.024 | 0.002 | 1.000e-200 | 3e-04 | 217.510 | 0.007 | 0.014 | 0.604 | 0.39760 |
| 1 | 153662423 | rs35479618 | A | G | 0.069 | 0.006 | 1.000e-200 | 2e-04 | 123.518 | 0.050 | 0.066 | 0.453 | 0.01392 |
| 17 | 43985636 | rs3785879 | A | C | 0.009 | 0.002 | 1.266e-08 | 0e+00 | 32.383 | -0.033 | 0.013 | 0.013 | 0.46520 |
| 16 | 24802325 | rs3803716 | T | C | -0.020 | 0.002 | 1.000e-200 | 1e-04 | 101.099 | 0.009 | 0.019 | 0.652 | 0.16200 |
| 7 | 116186241 | rs3807989 | A | G | 0.009 | 0.002 | 2.908e-08 | 0e+00 | 30.768 | 0.001 | 0.013 | 0.962 | 0.60640 |
| 16 | 4432029 | rs3810818 | A | C | 0.011 | 0.002 | 5.390e-09 | 0e+00 | 34.043 | -0.004 | 0.015 | 0.806 | 0.77630 |
| 6 | 7725760 | rs3812163 | A | T | -0.010 | 0.002 | 1.482e-10 | 1e-04 | 41.052 | -0.011 | 0.013 | 0.393 | 0.45230 |
| 14 | 73422259 | rs3815460 | C | G | -0.020 | 0.003 | 7.960e-13 | 1e-04 | 51.292 | 0.001 | 0.027 | 0.972 | 0.11230 |
| 11 | 1909006 | rs3817198 | T | C | 0.017 | 0.002 | 1.000e-200 | 1e-04 | 98.899 | -0.011 | 0.015 | 0.485 | 0.31410 |
| 1 | 242023898 | rs4149909 | A | G | -0.025 | 0.004 | 1.532e-08 | 0e+00 | 32.012 | -0.013 | 0.037 | 0.733 | 0.03777 |
| 6 | 32189032 | rs415929 | T | C | -0.017 | 0.002 | 1.000e-200 | 1e-04 | 89.414 | 0.005 | 0.014 | 0.745 | 0.35190 |
| 7 | 92248076 | rs42235 | T | C | -0.020 | 0.002 | 1.000e-200 | 2e-04 | 133.547 | 0.003 | 0.015 | 0.844 | 0.32210 |
| 5 | 53300662 | rs4311394 | A | G | -0.013 | 0.002 | 6.918e-13 | 1e-04 | 51.567 | 0.031 | 0.015 | 0.040 | 0.28430 |
| 2 | 55812061 | rs4312532 | A | G | 0.011 | 0.002 | 3.217e-12 | 1e-04 | 48.552 | 0.005 | 0.013 | 0.712 | 0.42250 |
| 18 | 3457606 | rs4468717 | T | C | -0.020 | 0.003 | 1.064e-11 | 1e-04 | 46.208 | -0.018 | 0.019 | 0.360 | 0.09443 |
| 3 | 169100899 | rs448378 | A | G | -0.023 | 0.002 | 1.000e-200 | 3e-04 | 210.651 | 0.002 | 0.013 | 0.870 | 0.51790 |
| 14 | 23865885 | rs452036 | A | G | -0.014 | 0.002 | 1.500e-16 | 1e-04 | 68.125 | 0.032 | 0.015 | 0.033 | 0.33600 |
| 17 | 7163739 | rs4562 | A | G | -0.014 | 0.002 | 4.000e-17 | 1e-04 | 70.436 | -0.003 | 0.014 | 0.815 | 0.64120 |
| 1 | 25044111 | rs4601530 | T | C | 0.013 | 0.002 | 7.671e-13 | 1e-04 | 51.365 | 0.005 | 0.015 | 0.722 | 0.26740 |
| 9 | 9261737 | rs4626664 | A | G | 0.016 | 0.002 | 3.754e-14 | 1e-04 | 57.293 | 0.000 | 0.018 | 1.000 | 0.14310 |
| 1 | 197126649 | rs4639796 | A | G | 0.013 | 0.002 | 2.950e-09 | 0e+00 | 35.217 | -0.008 | 0.016 | 0.603 | 0.16800 |
| 3 | 124646594 | rs4679392 | A | G | -0.011 | 0.002 | 9.464e-12 | 1e-04 | 46.436 | -0.010 | 0.013 | 0.478 | 0.42940 |
| 3 | 185307363 | rs4686683 | T | G | -0.010 | 0.002 | 6.637e-11 | 1e-04 | 42.623 | -0.010 | 0.014 | 0.458 | 0.38670 |
| 4 | 157720124 | rs4691380 | T | C | -0.012 | 0.002 | 5.740e-13 | 1e-04 | 51.934 | -0.021 | 0.015 | 0.178 | 0.35880 |
| 22 | 40729614 | rs470113 | A | G | -0.014 | 0.002 | 2.658e-12 | 1e-04 | 48.926 | -0.016 | 0.016 | 0.322 | 0.15610 |
| 5 | 112366678 | rs4705752 | A | G | 0.009 | 0.002 | 1.362e-08 | 0e+00 | 32.242 | 0.000 | 0.013 | 0.973 | 0.50890 |
| 7 | 128573967 | rs4728142 | A | G | -0.010 | 0.002 | 7.586e-10 | 0e+00 | 37.864 | 0.004 | 0.013 | 0.761 | 0.44930 |
| 11 | 16274295 | rs4757388 | A | G | 0.014 | 0.002 | 1.000e-200 | 1e-04 | 80.961 | -0.007 | 0.015 | 0.637 | 0.59540 |
| 16 | 52599188 | rs4784227 | T | C | 0.011 | 0.002 | 1.240e-09 | 0e+00 | 36.906 | -0.001 | 0.015 | 0.947 | 0.25450 |
| 20 | 11169603 | rs4813048 | T | C | -0.010 | 0.002 | 3.036e-11 | 1e-04 | 44.154 | -0.012 | 0.013 | 0.375 | 0.50400 |
| 22 | 29451671 | rs4823006 | A | G | 0.014 | 0.002 | 1.000e-200 | 1e-04 | 84.248 | -0.028 | 0.013 | 0.038 | 0.42740 |
| 4 | 77419073 | rs4859683 | T | C | -0.012 | 0.002 | 2.526e-13 | 1e-04 | 53.546 | 0.015 | 0.014 | 0.286 | 0.64810 |
| 6 | 127208390 | rs4897193 | A | G | 0.021 | 0.002 | 1.000e-200 | 2e-04 | 176.143 | 0.013 | 0.013 | 0.321 | 0.52390 |
| 3 | 27349047 | rs4973760 | A | G | 0.016 | 0.002 | 1.000e-200 | 1e-04 | 87.287 | 0.034 | 0.015 | 0.026 | 0.26340 |
| 17 | 43216281 | rs4986172 | T | C | 0.019 | 0.002 | 1.000e-200 | 2e-04 | 133.899 | -0.004 | 0.014 | 0.774 | 0.37970 |
| 18 | 72999359 | rs55679337 | T | C | -0.010 | 0.002 | 4.476e-09 | 0e+00 | 34.405 | 0.043 | 0.014 | 0.003 | 0.34990 |
| 6 | 25850845 | rs56027330 | T | C | 0.026 | 0.002 | 1.000e-200 | 1e-04 | 116.672 | -0.011 | 0.019 | 0.556 | 0.13620 |
| 15 | 50932357 | rs56170748 | T | C | -0.012 | 0.002 | 7.217e-14 | 1e-04 | 56.008 | 0.028 | 0.013 | 0.037 | 0.52090 |
| 5 | 158258461 | rs57392066 | T | C | 0.014 | 0.002 | 1.000e-200 | 1e-04 | 73.778 | 0.010 | 0.014 | 0.457 | 0.59740 |
| 16 | 75483806 | rs59155720 | A | G | -0.020 | 0.002 | 1.000e-200 | 2e-04 | 107.240 | 0.029 | 0.013 | 0.030 | 0.56760 |
| 20 | 62695931 | rs6062343 | A | G | -0.014 | 0.002 | 1.000e-200 | 1e-04 | 77.623 | 0.004 | 0.013 | 0.770 | 0.43540 |
| 20 | 47308798 | rs6095241 | A | G | -0.009 | 0.002 | 2.807e-08 | 0e+00 | 30.836 | -0.012 | 0.014 | 0.387 | 0.45630 |
| 17 | 1372839 | rs61753655 | T | C | 0.066 | 0.008 | 1.000e-200 | 1e-04 | 76.233 | -0.021 | 0.062 | 0.742 | 0.01093 |
| 9 | 136155000 | rs635634 | T | C | -0.011 | 0.002 | 3.982e-08 | 0e+00 | 30.158 | -0.019 | 0.016 | 0.242 | 0.18490 |
| 17 | 62408299 | rs6504218 | A | G | -0.014 | 0.002 | 1.000e-200 | 1e-04 | 76.306 | -0.007 | 0.013 | 0.574 | 0.56460 |
| 1 | 113098534 | rs6658555 | T | C | -0.017 | 0.002 | 1.000e-200 | 1e-04 | 94.532 | -0.012 | 0.016 | 0.431 | 0.24060 |
| 1 | 209950760 | rs669694 | C | G | -0.011 | 0.002 | 1.267e-08 | 0e+00 | 32.381 | 0.002 | 0.015 | 0.878 | 0.76740 |
| 1 | 89123443 | rs6699417 | T | C | -0.014 | 0.002 | 1.000e-200 | 1e-04 | 81.366 | 0.015 | 0.014 | 0.289 | 0.62330 |
| 2 | 135650766 | rs6714498 | T | C | 0.009 | 0.002 | 1.906e-08 | 0e+00 | 31.588 | 0.018 | 0.014 | 0.188 | 0.49900 |
| 2 | 203913552 | rs6715752 | A | G | -0.013 | 0.002 | 3.720e-15 | 1e-04 | 61.840 | 0.028 | 0.013 | 0.035 | 0.40560 |
| 2 | 37559355 | rs6734118 | A | C | -0.016 | 0.002 | 1.000e-200 | 1e-04 | 75.332 | 0.016 | 0.016 | 0.327 | 0.24350 |
| 5 | 173362458 | rs6861681 | A | G | -0.012 | 0.002 | 1.753e-12 | 1e-04 | 49.743 | 0.023 | 0.014 | 0.089 | 0.28730 |
| 6 | 43758873 | rs6905288 | A | G | 0.011 | 0.002 | 3.542e-12 | 1e-04 | 48.363 | -0.002 | 0.013 | 0.908 | 0.59640 |
| 7 | 27223563 | rs6968828 | T | G | 0.009 | 0.002 | 1.737e-08 | 0e+00 | 31.769 | 0.012 | 0.013 | 0.386 | 0.57160 |
| 1 | 230845794 | rs699 | A | G | -0.021 | 0.002 | 1.000e-200 | 2e-04 | 167.820 | -0.001 | 0.013 | 0.962 | 0.41150 |
| 17 | 26694861 | rs704 | A | G | -0.010 | 0.002 | 7.320e-11 | 1e-04 | 42.432 | 0.012 | 0.013 | 0.365 | 0.44930 |
| 10 | 12277992 | rs7068966 | T | C | -0.010 | 0.002 | 3.649e-10 | 0e+00 | 39.292 | -0.003 | 0.013 | 0.836 | 0.50600 |
| 10 | 115789375 | rs7076938 | T | C | 0.026 | 0.002 | 1.000e-200 | 3e-04 | 217.680 | -0.006 | 0.015 | 0.696 | 0.67890 |
| 14 | 100625902 | rs7157599 | T | C | -0.011 | 0.002 | 7.030e-09 | 0e+00 | 33.526 | 0.006 | 0.015 | 0.674 | 0.69480 |
| 2 | 164731394 | rs717366 | T | C | 0.014 | 0.002 | 1.000e-200 | 1e-04 | 79.861 | -0.010 | 0.013 | 0.464 | 0.37280 |
| 15 | 40360741 | rs7181230 | A | G | 0.011 | 0.002 | 1.854e-11 | 1e-04 | 45.120 | -0.012 | 0.014 | 0.375 | 0.32600 |
| 12 | 26453283 | rs718314 | A | G | -0.013 | 0.002 | 5.514e-12 | 1e-04 | 47.495 | 0.004 | 0.015 | 0.766 | 0.24350 |
| 19 | 7224431 | rs7248104 | A | G | -0.015 | 0.002 | 1.000e-200 | 1e-04 | 90.624 | 0.005 | 0.013 | 0.705 | 0.42150 |
| 19 | 31863085 | rs7248171 | A | G | 0.016 | 0.002 | 1.000e-200 | 1e-04 | 98.888 | -0.022 | 0.013 | 0.091 | 0.34490 |
| 2 | 40527128 | rs727477 | T | G | 0.012 | 0.002 | 6.936e-12 | 1e-04 | 47.046 | -0.007 | 0.014 | 0.599 | 0.28630 |
| 12 | 115891403 | rs7315438 | T | C | 0.014 | 0.002 | 1.000e-200 | 1e-04 | 75.958 | 0.010 | 0.014 | 0.496 | 0.55270 |
| 7 | 139415775 | rs7456421 | C | G | -0.017 | 0.002 | 1.000e-200 | 1e-04 | 74.483 | -0.004 | 0.016 | 0.817 | 0.21270 |
| 2 | 25116977 | rs7586879 | T | C | -0.015 | 0.002 | 1.000e-200 | 1e-04 | 79.803 | -0.006 | 0.014 | 0.690 | 0.34990 |
| 3 | 157992814 | rs7643792 | A | G | 0.009 | 0.002 | 4.283e-09 | 0e+00 | 34.491 | -0.014 | 0.013 | 0.298 | 0.44430 |
| 9 | 35906471 | rs76452347 | T | C | -0.016 | 0.002 | 3.200e-15 | 1e-04 | 62.139 | 0.002 | 0.018 | 0.927 | 0.20780 |
| 4 | 144060464 | rs7666785 | A | G | 0.011 | 0.002 | 4.831e-12 | 1e-04 | 47.754 | -0.007 | 0.014 | 0.614 | 0.60140 |
| 8 | 81426196 | rs76767219 | A | C | -0.030 | 0.004 | 3.291e-12 | 1e-04 | 48.507 | 0.024 | 0.044 | 0.576 | 0.02783 |
| 4 | 156635309 | rs7692387 | A | G | -0.022 | 0.002 | 1.000e-200 | 1e-04 | 119.366 | -0.026 | 0.016 | 0.093 | 0.20480 |
| 6 | 20679709 | rs7756992 | A | G | -0.013 | 0.002 | 1.603e-12 | 1e-04 | 49.918 | 0.001 | 0.014 | 0.940 | 0.28130 |
| 8 | 9649769 | rs7830613 | T | C | 0.015 | 0.002 | 1.000e-200 | 1e-04 | 75.066 | 0.023 | 0.014 | 0.086 | 0.29030 |
| 8 | 110007151 | rs7838433 | T | G | 0.010 | 0.002 | 7.918e-09 | 0e+00 | 33.295 | 0.014 | 0.014 | 0.336 | 0.29420 |
| 10 | 28228865 | rs7893462 | A | G | -0.011 | 0.002 | 1.569e-12 | 1e-04 | 49.960 | -0.025 | 0.013 | 0.055 | 0.54770 |
| 11 | 10669228 | rs7940646 | T | C | 0.015 | 0.002 | 1.000e-200 | 1e-04 | 85.054 | 0.004 | 0.014 | 0.763 | 0.69980 |
| 4 | 48493237 | rs79858408 | A | G | -0.012 | 0.002 | 1.370e-13 | 1e-04 | 54.748 | -0.010 | 0.013 | 0.434 | 0.51090 |
| 5 | 140115303 | rs801171 | A | G | 0.011 | 0.002 | 8.647e-13 | 1e-04 | 51.129 | -0.016 | 0.013 | 0.219 | 0.43040 |
| 15 | 75393720 | rs8040061 | A | C | 0.016 | 0.002 | 1.000e-200 | 1e-04 | 95.092 | 0.006 | 0.013 | 0.672 | 0.41350 |
| 16 | 72688650 | rs8048832 | T | C | -0.013 | 0.002 | 2.091e-08 | 0e+00 | 31.408 | 0.028 | 0.019 | 0.130 | 0.88070 |
| 17 | 59483766 | rs8068318 | T | C | 0.021 | 0.002 | 1.000e-200 | 2e-04 | 144.612 | 0.027 | 0.015 | 0.064 | 0.72760 |
| 11 | 117283676 | rs8258 | T | C | 0.011 | 0.002 | 6.011e-12 | 1e-04 | 47.326 | -0.024 | 0.014 | 0.079 | 0.63120 |
| 7 | 28196222 | rs849134 | A | G | 0.009 | 0.002 | 3.211e-09 | 0e+00 | 35.051 | -0.002 | 0.013 | 0.868 | 0.51490 |
| 1 | 10796866 | rs880315 | T | C | -0.024 | 0.002 | 1.000e-200 | 3e-04 | 219.053 | -0.015 | 0.013 | 0.263 | 0.35690 |
| 7 | 150704843 | rs891511 | A | G | -0.021 | 0.002 | 1.000e-200 | 2e-04 | 145.559 | -0.010 | 0.013 | 0.455 | 0.33500 |
| 11 | 1874072 | rs907611 | A | G | 0.022 | 0.002 | 1.000e-200 | 2e-04 | 142.906 | -0.001 | 0.014 | 0.920 | 0.32700 |
| 2 | 218674697 | rs918949 | T | C | -0.014 | 0.002 | 1.000e-200 | 1e-04 | 77.313 | -0.013 | 0.013 | 0.339 | 0.58650 |
| 11 | 27667202 | rs925946 | T | G | -0.012 | 0.002 | 3.546e-12 | 1e-04 | 48.361 | 0.006 | 0.014 | 0.655 | 0.71470 |
| 21 | 45107562 | rs9306160 | T | C | -0.013 | 0.002 | 1.290e-15 | 1e-04 | 63.918 | -0.019 | 0.014 | 0.183 | 0.61730 |
| 20 | 42826350 | rs9346 | A | C | -0.018 | 0.002 | 2.690e-15 | 1e-04 | 62.476 | 0.010 | 0.017 | 0.542 | 0.15310 |
| 6 | 12903957 | rs9349379 | A | G | 0.016 | 0.002 | 1.000e-200 | 1e-04 | 101.572 | 0.007 | 0.013 | 0.575 | 0.40060 |
| 6 | 147701133 | rs9373523 | T | G | 0.011 | 0.002 | 4.555e-11 | 1e-04 | 43.360 | 0.005 | 0.013 | 0.709 | 0.56560 |
| 6 | 97063522 | rs9373985 | C | G | -0.012 | 0.002 | 4.260e-13 | 1e-04 | 52.519 | 0.022 | 0.014 | 0.119 | 0.34790 |
| 10 | 96013705 | rs9419788 | A | G | -0.019 | 0.002 | 1.000e-200 | 2e-04 | 143.726 | -0.012 | 0.013 | 0.380 | 0.62820 |
| 8 | 129316014 | rs975730 | A | G | -0.010 | 0.002 | 3.038e-10 | 0e+00 | 39.650 | 0.019 | 0.015 | 0.210 | 0.35690 |
| 3 | 48282695 | rs9847953 | A | G | 0.015 | 0.002 | 1.000e-200 | 1e-04 | 76.818 | 0.021 | 0.016 | 0.198 | 0.29130 |
| 1 | 56965664 | rs9970807 | T | C | -0.018 | 0.003 | 6.745e-11 | 1e-04 | 42.592 | -0.023 | 0.021 | 0.276 | 0.11830 |

**Supplementary table 2.11. Detailed information of instrumental SNPs for Fasting glucose in the analysis of PPD.**

| **Chr** | **Position** | **SNP** | **EA** | **OA** | **beta.exposure** | **se.exposure** | **pval.exposure** | **R2** | **F_val** | **beta.outcome** | **se.outcome** | **pval.outcome** | **Eaf.outcome** |
| --- | --- | --- | --- | --- | --- | --- | --- | --- | --- | --- | --- | --- | --- |
| 6 | 39034095 | rs10305457 | T | C | 0.024 | 0.003 | 1.20893e-14 | 0.0001 | 53.931 | -0.032 | 0.024 | 0.180 | 0.08151 |
| 7 | 15063430 | rs10487796 | A | T | -0.026 | 0.002 | 4.61849e-52 | 0.0003 | 266.098 | -0.003 | 0.013 | 0.798 | 0.48810 |
| 2 | 27324036 | rs1057394 | A | G | -0.012 | 0.002 | 1.91117e-12 | 0.0001 | 47.457 | 0.027 | 0.014 | 0.052 | 0.60240 |
| 9 | 22134068 | rs10811660 | A | G | -0.022 | 0.002 | 7.93780e-25 | 0.0001 | 102.746 | 0.000 | 0.019 | 0.995 | 0.16800 |
| 11 | 92708710 | rs10830963 | G | C | 0.077 | 0.002 | 1.00000e-200 | 0.0024 | 1650.925 | 0.033 | 0.014 | 0.018 | 0.28830 |
| 11 | 45870177 | rs10838524 | G | A | -0.024 | 0.002 | 1.55883e-40 | 0.0003 | 221.266 | 0.004 | 0.013 | 0.746 | 0.52390 |
| 11 | 47350553 | rs10838693 | C | G | 0.018 | 0.002 | 3.44191e-23 | 0.0001 | 96.694 | -0.015 | 0.014 | 0.262 | 0.31010 |
| 9 | 4291928 | rs10974438 | C | A | 0.020 | 0.002 | 9.84691e-31 | 0.0002 | 135.654 | -0.018 | 0.014 | 0.187 | 0.38370 |
| 11 | 72460694 | rs11603349 | C | T | -0.024 | 0.002 | 3.11602e-25 | 0.0002 | 115.074 | 0.061 | 0.015 | 0.000 | 0.15110 |
| 12 | 133063768 | rs11610045 | A | G | 0.014 | 0.002 | 3.25987e-13 | 0.0001 | 57.440 | -0.007 | 0.013 | 0.619 | 0.45130 |
| 13 | 28487599 | rs11619319 | G | A | 0.017 | 0.002 | 3.41193e-20 | 0.0001 | 74.823 | -0.009 | 0.015 | 0.561 | 0.22860 |
| 3 | 123065778 | rs11708067 | G | A | -0.028 | 0.002 | 1.62592e-43 | 0.0002 | 197.403 | -0.003 | 0.018 | 0.870 | 0.17100 |
| 6 | 153431125 | rs12055786 | T | C | 0.012 | 0.002 | 1.16600e-11 | 0.0001 | 49.827 | -0.002 | 0.013 | 0.886 | 0.40160 |
| 8 | 81076874 | rs12541643 | T | C | 0.012 | 0.002 | 4.51097e-09 | 0.0001 | 38.571 | 0.030 | 0.013 | 0.024 | 0.50200 |
| 2 | 27730940 | rs1260326 | C | T | 0.028 | 0.002 | 4.48126e-65 | 0.0004 | 275.170 | 0.004 | 0.014 | 0.762 | 0.58950 |
| 10 | 113036354 | rs12784552 | G | A | -0.033 | 0.003 | 2.86286e-31 | 0.0002 | 120.268 | -0.015 | 0.023 | 0.528 | 0.07952 |
| 14 | 100830818 | rs12888855 | A | C | -0.014 | 0.002 | 6.01866e-12 | 0.0001 | 45.562 | 0.025 | 0.017 | 0.141 | 0.19090 |
| 15 | 75090349 | rs12898997 | T | C | -0.010 | 0.002 | 4.64098e-09 | 0.0000 | 33.232 | -0.016 | 0.013 | 0.225 | 0.60440 |
| 5 | 55809127 | rs157512 | C | T | -0.013 | 0.002 | 5.42600e-10 | 0.0001 | 40.717 | 0.011 | 0.015 | 0.433 | 0.27440 |
| 3 | 170709193 | rs1604038 | T | C | -0.020 | 0.002 | 4.46786e-28 | 0.0002 | 121.000 | -0.007 | 0.015 | 0.632 | 0.28930 |
| 3 | 141134818 | rs16851397 | G | A | -0.033 | 0.004 | 1.26009e-12 | 0.0001 | 60.617 | -0.057 | 0.037 | 0.121 | 0.04573 |
| 9 | 111680359 | rs16913693 | G | T | -0.039 | 0.005 | 2.81968e-16 | 0.0001 | 64.655 | 0.038 | 0.044 | 0.395 | 0.02584 |
| 7 | 14898282 | rs17168486 | T | C | 0.028 | 0.002 | 4.16965e-36 | 0.0002 | 177.778 | 0.045 | 0.016 | 0.006 | 0.16600 |
| 20 | 39832628 | rs17265513 | C | T | 0.016 | 0.002 | 5.10152e-14 | 0.0001 | 56.608 | 0.036 | 0.015 | 0.015 | 0.19480 |
| 15 | 60862500 | rs17270243 | G | A | 0.010 | 0.002 | 3.61601e-08 | 0.0000 | 24.526 | -0.024 | 0.017 | 0.148 | 0.23560 |
| 3 | 152180329 | rs17437560 | T | C | -0.018 | 0.003 | 3.33096e-08 | 0.0001 | 29.907 | -0.056 | 0.028 | 0.051 | 0.10740 |
| 11 | 61609750 | rs174583 | T | C | -0.017 | 0.002 | 3.37132e-22 | 0.0001 | 97.661 | 0.011 | 0.013 | 0.397 | 0.36280 |
| 5 | 95696585 | rs1820176 | C | T | -0.025 | 0.002 | 1.90502e-34 | 0.0003 | 152.523 | -0.012 | 0.014 | 0.410 | 0.28030 |
| 2 | 54941112 | rs189548 | A | G | -0.012 | 0.002 | 2.81099e-09 | 0.0001 | 37.822 | -0.003 | 0.016 | 0.842 | 0.72760 |
| 7 | 89853149 | rs194518 | A | G | 0.010 | 0.002 | 8.75709e-09 | 0.0001 | 32.111 | -0.008 | 0.013 | 0.533 | 0.51190 |
| 16 | 4014282 | rs2238435 | G | C | -0.011 | 0.002 | 3.82402e-09 | 0.0001 | 34.748 | -0.001 | 0.013 | 0.929 | 0.61430 |
| 2 | 169700463 | rs2461385 | C | T | -0.022 | 0.002 | 2.73023e-19 | 0.0001 | 81.752 | -0.028 | 0.021 | 0.173 | 0.84590 |
| 7 | 44148553 | rs2595701 | G | A | -0.019 | 0.002 | 4.47816e-19 | 0.0002 | 81.000 | 0.001 | 0.014 | 0.933 | 0.67990 |
| 12 | 56865338 | rs2657879 | G | A | 0.012 | 0.002 | 7.33398e-09 | 0.0000 | 29.258 | 0.003 | 0.017 | 0.841 | 0.21270 |
| 10 | 26505822 | rs2839671 | A | G | -0.016 | 0.002 | 8.37915e-14 | 0.0001 | 52.893 | 0.006 | 0.019 | 0.764 | 0.16800 |
| 1 | 229672955 | rs348330 | A | G | -0.012 | 0.002 | 3.03599e-10 | 0.0001 | 37.210 | 0.011 | 0.014 | 0.433 | 0.64210 |
| 14 | 90055468 | rs35889227 | T | G | -0.013 | 0.002 | 3.36504e-10 | 0.0001 | 46.814 | 0.014 | 0.014 | 0.300 | 0.60740 |
| 6 | 7250270 | rs3778321 | A | G | -0.019 | 0.002 | 3.15718e-17 | 0.0001 | 78.449 | -0.009 | 0.016 | 0.591 | 0.15710 |
| 9 | 139256766 | rs3829109 | A | G | -0.016 | 0.002 | 1.08693e-15 | 0.0001 | 66.422 | 0.011 | 0.014 | 0.422 | 0.28230 |
| 11 | 2181060 | rs3842753 | G | T | -0.013 | 0.002 | 2.84099e-09 | 0.0001 | 37.099 | 0.023 | 0.016 | 0.154 | 0.72560 |
| 22 | 30343186 | rs39713 | T | C | -0.017 | 0.003 | 1.76701e-08 | 0.0000 | 29.720 | -0.039 | 0.024 | 0.097 | 0.06461 |
| 12 | 57771153 | rs4760278 | A | C | -0.011 | 0.002 | 3.33096e-08 | 0.0000 | 30.250 | 0.009 | 0.016 | 0.557 | 0.18590 |
| 4 | 185726548 | rs4862423 | T | C | 0.012 | 0.002 | 4.44703e-10 | 0.0001 | 41.909 | 0.002 | 0.014 | 0.893 | 0.39460 |
| 2 | 169774646 | rs537183 | T | C | 0.066 | 0.002 | 1.00000e-200 | 0.0020 | 1521.000 | -0.024 | 0.014 | 0.090 | 0.64310 |
| 20 | 22557099 | rs6113722 | A | G | -0.042 | 0.004 | 7.65949e-25 | 0.0002 | 92.860 | 0.051 | 0.035 | 0.148 | 0.06561 |
| 12 | 121893626 | rs6489811 | G | A | 0.011 | 0.002 | 3.26701e-09 | 0.0001 | 37.346 | -0.043 | 0.014 | 0.002 | 0.50000 |
| 12 | 97848910 | rs6538804 | G | C | -0.014 | 0.002 | 9.41239e-14 | 0.0001 | 55.856 | 0.004 | 0.014 | 0.784 | 0.39260 |
| 15 | 99271135 | rs6598541 | G | A | -0.011 | 0.002 | 4.12382e-12 | 0.0001 | 44.969 | 0.017 | 0.014 | 0.223 | 0.65510 |
| 1 | 100894419 | rs6662924 | A | C | 0.014 | 0.002 | 3.34103e-10 | 0.0001 | 38.656 | 0.024 | 0.017 | 0.145 | 0.19280 |
| 3 | 187740523 | rs6808574 | C | T | 0.013 | 0.002 | 7.20941e-14 | 0.0001 | 55.810 | -0.016 | 0.014 | 0.255 | 0.60540 |
| 8 | 9173209 | rs7012637 | A | G | -0.018 | 0.002 | 9.74541e-25 | 0.0002 | 112.111 | 0.002 | 0.013 | 0.852 | 0.47320 |
| 10 | 95384152 | rs7095788 | T | C | -0.011 | 0.002 | 1.97601e-09 | 0.0001 | 34.679 | 0.031 | 0.014 | 0.026 | 0.36480 |
| 15 | 62391608 | rs7163757 | T | C | -0.022 | 0.002 | 2.64119e-36 | 0.0002 | 183.941 | -0.009 | 0.013 | 0.508 | 0.41550 |
| 15 | 77747190 | rs7178572 | G | A | 0.012 | 0.002 | 7.08598e-10 | 0.0001 | 45.188 | 0.012 | 0.014 | 0.398 | 0.69280 |
| 5 | 76425867 | rs7708285 | A | G | -0.013 | 0.002 | 1.25300e-09 | 0.0001 | 49.000 | 0.012 | 0.016 | 0.458 | 0.67790 |
| 2 | 43777964 | rs77981966 | T | C | -0.025 | 0.004 | 1.58016e-14 | 0.0001 | 49.401 | 0.015 | 0.037 | 0.689 | 0.05765 |
| 1 | 150868102 | rs78132593 | A | C | -0.015 | 0.002 | 2.59801e-10 | 0.0001 | 44.647 | -0.003 | 0.017 | 0.854 | 0.18890 |
| 10 | 114758349 | rs7903146 | T | C | 0.026 | 0.002 | 1.99480e-35 | 0.0003 | 185.820 | -0.012 | 0.017 | 0.480 | 0.31710 |
| 7 | 44255643 | rs878521 | A | G | 0.055 | 0.002 | 2.64850e-174 | 0.0011 | 753.503 | 0.008 | 0.016 | 0.641 | 0.25350 |
| 8 | 95960511 | rs896854 | C | T | -0.010 | 0.002 | 5.61203e-09 | 0.0000 | 38.285 | -0.007 | 0.013 | 0.597 | 0.53980 |
| 6 | 20680678 | rs9348441 | A | T | 0.018 | 0.002 | 4.40251e-20 | 0.0001 | 95.605 | -0.001 | 0.014 | 0.949 | 0.27830 |
| 8 | 118204020 | rs9650069 | T | C | -0.029 | 0.002 | 8.30615e-58 | 0.0003 | 252.457 | 0.006 | 0.014 | 0.652 | 0.28130 |

**Supplementary table 2.12. Detailed information of instrumental SNPs for Fasting insulin in the analysis of PPD.**

| **Chr** | **Position** | **SNP** | **EA** | **OA** | **beta.exposure** | **se.exposure** | **pval.exposure** | **R2** | **F_val** | **beta.outcome** | **se.outcome** | **pval.outcome** | **Eaf.outcome** |
| --- | --- | --- | --- | --- | --- | --- | --- | --- | --- | --- | --- | --- | --- |
| 5 | 157918946 | rs10050393 | C | T | -0.009 | 0.002 | 4.84295e-08 | 0e+00 | 22.438 | 0.010 | 0.013 | 0.461 | 0.43040 |
| 3 | 49891002 | rs10865959 | C | G | 0.014 | 0.002 | 1.99200e-08 | 1e-04 | 39.347 | 0.040 | 0.015 | 0.009 | 0.30820 |
| 6 | 34222201 | rs116141873 | T | G | 0.043 | 0.006 | 1.42298e-11 | 1e-04 | 52.624 | 0.010 | 0.040 | 0.797 | 0.02982 |
| 3 | 123065778 | rs11708067 | G | A | 0.014 | 0.002 | 1.29900e-09 | 1e-04 | 34.452 | -0.003 | 0.018 | 0.870 | 0.17100 |
| 4 | 145659064 | rs11727676 | C | T | 0.020 | 0.004 | 2.89801e-08 | 1e-04 | 27.093 | -0.011 | 0.024 | 0.652 | 0.07654 |
| 10 | 89680631 | rs118164457 | C | T | 0.034 | 0.006 | 3.85798e-10 | 1e-04 | 36.634 | 0.025 | 0.058 | 0.667 | 0.03777 |
| 20 | 45582472 | rs1206760 | A | G | -0.011 | 0.002 | 8.82104e-10 | 1e-04 | 34.748 | -0.003 | 0.013 | 0.800 | 0.54470 |
| 18 | 60845884 | rs12454712 | C | T | -0.014 | 0.002 | 1.77701e-09 | 1e-04 | 32.262 | 0.010 | 0.013 | 0.472 | 0.38570 |
| 2 | 27730940 | rs1260326 | C | T | 0.023 | 0.002 | 8.41783e-38 | 3e-04 | 147.814 | 0.004 | 0.014 | 0.762 | 0.58950 |
| 8 | 23615445 | rs13258890 | C | T | -0.013 | 0.002 | 2.76503e-08 | 1e-04 | 26.214 | 0.031 | 0.016 | 0.062 | 0.25750 |
| 2 | 165528876 | rs13389219 | T | C | -0.020 | 0.002 | 5.83848e-28 | 2e-04 | 109.698 | 0.002 | 0.014 | 0.887 | 0.43540 |
| 12 | 66351826 | rs1351394 | C | T | 0.011 | 0.002 | 2.70801e-09 | 1e-04 | 38.028 | -0.001 | 0.013 | 0.935 | 0.54970 |
| 6 | 127449246 | rs1474696 | G | A | 0.015 | 0.002 | 3.01926e-16 | 1e-04 | 66.694 | -0.036 | 0.013 | 0.007 | 0.48410 |
| 3 | 12287863 | rs17036126 | T | C | 0.021 | 0.003 | 1.27900e-10 | 1e-04 | 48.534 | 0.000 | 0.023 | 0.993 | 0.12620 |
| 3 | 52844534 | rs17331151 | T | C | -0.016 | 0.003 | 1.52398e-08 | 0e+00 | 27.309 | -0.067 | 0.024 | 0.005 | 0.10240 |
| 7 | 50786663 | rs2108349 | A | G | -0.012 | 0.002 | 1.13300e-08 | 1e-04 | 33.062 | 0.003 | 0.013 | 0.815 | 0.70580 |
| 6 | 34236973 | rs2780215 | G | A | -0.039 | 0.006 | 1.06400e-09 | 1e-04 | 38.716 | -0.022 | 0.040 | 0.591 | 0.04672 |
| 11 | 63869062 | rs2845885 | T | C | -0.020 | 0.004 | 1.17999e-08 | 1e-04 | 27.361 | 0.016 | 0.025 | 0.520 | 0.92350 |
| 2 | 227099534 | rs2943646 | G | A | 0.025 | 0.002 | 8.47227e-39 | 3e-04 | 173.130 | -0.008 | 0.014 | 0.559 | 0.62430 |
| 3 | 12351521 | rs35000407 | G | T | -0.026 | 0.003 | 1.50383e-21 | 1e-04 | 84.903 | -0.016 | 0.018 | 0.348 | 0.11930 |
| 4 | 89739808 | rs3775380 | G | A | 0.012 | 0.002 | 1.47707e-11 | 1e-04 | 43.707 | 0.005 | 0.013 | 0.693 | 0.49200 |
| 5 | 55806751 | rs459193 | G | A | 0.018 | 0.002 | 1.12305e-18 | 1e-04 | 74.288 | -0.012 | 0.014 | 0.398 | 0.70870 |
| 5 | 53272664 | rs4865796 | A | G | 0.016 | 0.002 | 7.32825e-17 | 1e-04 | 68.062 | -0.011 | 0.014 | 0.433 | 0.69680 |
| 2 | 630902 | rs5017305 | T | A | -0.014 | 0.003 | 1.06699e-08 | 1e-04 | 27.765 | 0.019 | 0.018 | 0.296 | 0.82600 |
| 3 | 150066540 | rs62271373 | A | T | 0.026 | 0.005 | 1.59599e-08 | 1e-04 | 28.444 | 0.029 | 0.032 | 0.367 | 0.06163 |
| 12 | 21699928 | rs6487237 | A | C | 0.015 | 0.003 | 4.68198e-09 | 1e-04 | 35.083 | 0.019 | 0.017 | 0.264 | 0.77140 |
| 4 | 157670537 | rs6855363 | C | T | -0.013 | 0.002 | 4.03896e-08 | 1e-04 | 39.062 | -0.019 | 0.015 | 0.225 | 0.35980 |
| 6 | 43758873 | rs6905288 | A | G | 0.011 | 0.002 | 7.74997e-09 | 1e-04 | 34.748 | -0.002 | 0.013 | 0.908 | 0.59640 |
| 8 | 9173358 | rs7012814 | A | G | -0.022 | 0.002 | 8.34257e-30 | 2e-04 | 132.856 | 0.003 | 0.013 | 0.826 | 0.47420 |
| 12 | 124409502 | rs7133378 | A | G | -0.013 | 0.002 | 5.99653e-11 | 1e-04 | 40.322 | 0.029 | 0.015 | 0.048 | 0.34990 |
| 6 | 164126233 | rs73013411 | A | C | -0.018 | 0.003 | 2.08401e-08 | 1e-04 | 31.641 | 0.014 | 0.024 | 0.561 | 0.12520 |
| 19 | 33899065 | rs731839 | A | G | -0.012 | 0.002 | 3.86456e-11 | 1e-04 | 40.557 | 0.015 | 0.014 | 0.287 | 0.66700 |
| 9 | 136132954 | rs75179845 | C | T | 0.022 | 0.004 | 6.05062e-11 | 1e-04 | 38.087 | 0.021 | 0.019 | 0.274 | 0.07753 |
| 10 | 114758349 | rs7903146 | T | C | -0.012 | 0.002 | 1.23700e-09 | 1e-04 | 30.512 | -0.012 | 0.017 | 0.480 | 0.31710 |
| 12 | 102898446 | rs860598 | A | G | 0.018 | 0.002 | 6.87860e-12 | 1e-04 | 50.126 | 0.002 | 0.016 | 0.915 | 0.82600 |
| 7 | 130466854 | rs972283 | G | A | 0.011 | 0.002 | 1.09300e-08 | 1e-04 | 30.540 | 0.002 | 0.013 | 0.890 | 0.56060 |
| 4 | 106081636 | rs9884482 | C | T | 0.013 | 0.002 | 2.87872e-11 | 1e-04 | 43.283 | -0.005 | 0.014 | 0.731 | 0.40850 |

**Supplementary table 2.13. Detailed information of instrumental SNPs for 2-hour glucose in the analysis of PPD.**

| **Chr** | **Position** | **SNP** | **EA** | **OA** | **beta.exposure** | **se.exposure** | **pval.exposure** | **R2** | **F_val** | **beta.outcome** | **se.outcome** | **pval.outcome** | **Eaf.outcome** |
| --- | --- | --- | --- | --- | --- | --- | --- | --- | --- | --- | --- | --- | --- |
| 3 | 123065778 | rs11708067 | A | G | 0.087 | 0.009 | 1.979e-22 | 0.0022 | 87.916 | 0.003 | 0.018 | 0.870 | 0.17100 |
| 17 | 7185779 | rs117643180 | A | C | 0.234 | 0.033 | 7.308e-14 | 0.0035 | 51.208 | 0.027 | 0.045 | 0.553 | 0.03082 |
| 2 | 27730940 | rs1260326 | T | C | 0.049 | 0.008 | 5.929e-12 | 0.0011 | 38.822 | -0.004 | 0.014 | 0.762 | 0.58950 |
| 2 | 165558252 | rs12692738 | T | C | 0.049 | 0.009 | 2.716e-08 | 0.0009 | 29.160 | -0.003 | 0.017 | 0.868 | 0.28330 |
| 15 | 62332980 | rs17271305 | A | G | -0.059 | 0.008 | 2.883e-14 | 0.0017 | 58.116 | 0.008 | 0.013 | 0.554 | 0.40060 |
| 19 | 46181392 | rs1800437 | C | G | 0.100 | 0.010 | 4.788e-26 | 0.0033 | 102.848 | 0.004 | 0.015 | 0.780 | 0.21370 |
| 8 | 9185146 | rs2126259 | T | C | -0.072 | 0.012 | 2.976e-10 | 0.0008 | 35.015 | -0.022 | 0.018 | 0.236 | 0.91350 |
| 12 | 121380544 | rs2649999 | T | C | 0.050 | 0.008 | 2.009e-10 | 0.0011 | 36.883 | 0.023 | 0.014 | 0.084 | 0.63520 |
| 11 | 17415190 | rs4148646 | C | G | 0.040 | 0.008 | 4.386e-08 | 0.0007 | 25.905 | 0.008 | 0.013 | 0.571 | 0.65010 |
| 10 | 114758349 | rs7903146 | T | C | 0.085 | 0.009 | 2.793e-26 | 0.0031 | 96.356 | -0.012 | 0.017 | 0.480 | 0.31710 |
| 7 | 44255643 | rs878521 | A | G | 0.099 | 0.009 | 1.254e-28 | 0.0037 | 110.921 | 0.008 | 0.016 | 0.641 | 0.25350 |

**Supplementary table 2.14. Detailed information of instrumental SNPs for Triglycerides in the analysis of PPD.**

| **Chr** | **Position** | **SNP** | **EA** | **OA** | **beta.exposure** | **se.exposure** | **pval.exposure** | **R2** | **F_val** | **beta.outcome** | **se.outcome** | **pval.outcome** | **Eaf.outcome** |
| --- | --- | --- | --- | --- | --- | --- | --- | --- | --- | --- | --- | --- | --- |
| 2 | 65276049 | rs1009360 | C | T | -0.018 | 0.002 | 3.40017e-20 | 0.0002 | 84.741 | -0.012 | 0.014 | 0.360 | 0.43840 |
| 15 | 101890913 | rs10152471 | A | G | -0.014 | 0.002 | 4.00037e-11 | 0.0001 | 43.596 | 0.010 | 0.013 | 0.434 | 0.41250 |
| 2 | 28646847 | rs10210970 | T | C | 0.023 | 0.003 | 1.99986e-15 | 0.0001 | 63.089 | -0.004 | 0.017 | 0.811 | 0.14120 |
| 7 | 17920613 | rs10242866 | T | C | 0.016 | 0.002 | 7.00003e-15 | 0.0001 | 60.599 | -0.022 | 0.014 | 0.131 | 0.40160 |
| 7 | 17284989 | rs10277582 | T | C | -0.017 | 0.003 | 1.70000e-08 | 0.0001 | 31.807 | 0.013 | 0.021 | 0.541 | 0.11630 |
| 15 | 102068658 | rs1037117 | A | G | 0.017 | 0.002 | 5.70033e-14 | 0.0001 | 56.478 | 0.007 | 0.015 | 0.646 | 0.26140 |
| 19 | 33728001 | rs10405944 | C | T | -0.013 | 0.002 | 6.20012e-11 | 0.0001 | 42.762 | 0.010 | 0.014 | 0.479 | 0.46520 |
| 1 | 230416399 | rs1043897 | T | G | -0.015 | 0.002 | 3.40017e-13 | 0.0001 | 52.952 | 0.004 | 0.014 | 0.774 | 0.40560 |
| 1 | 149871905 | rs1044808 | C | G | -0.025 | 0.004 | 8.30042e-12 | 0.0001 | 46.693 | -0.053 | 0.029 | 0.066 | 0.08648 |
| 5 | 118729286 | rs1045241 | T | C | -0.021 | 0.002 | 3.19963e-20 | 0.0002 | 84.857 | 0.012 | 0.014 | 0.408 | 0.26040 |
| 3 | 170727218 | rs10513688 | A | G | 0.025 | 0.003 | 1.39991e-13 | 0.0001 | 54.753 | -0.005 | 0.022 | 0.838 | 0.11030 |
| 11 | 65473798 | rs10750766 | A | C | 0.019 | 0.002 | 7.80010e-19 | 0.0002 | 78.540 | 0.030 | 0.015 | 0.049 | 0.70970 |
| 12 | 123736084 | rs10773000 | T | G | -0.015 | 0.002 | 1.69981e-12 | 0.0001 | 49.836 | -0.003 | 0.014 | 0.829 | 0.35880 |
| 12 | 124506631 | rs10773049 | C | T | -0.029 | 0.002 | 4.10015e-46 | 0.0004 | 203.256 | -0.003 | 0.013 | 0.827 | 0.43940 |
| 17 | 46197755 | rs10775406 | G | A | 0.021 | 0.002 | 6.59933e-19 | 0.0002 | 78.886 | 0.022 | 0.015 | 0.137 | 0.73360 |
| 15 | 58723426 | rs1077835 | G | A | 0.047 | 0.002 | 2.19989e-86 | 0.0008 | 388.053 | 0.005 | 0.015 | 0.759 | 0.21370 |
| 9 | 92202495 | rs10797119 | C | T | 0.016 | 0.002 | 4.60045e-15 | 0.0001 | 61.425 | 0.009 | 0.013 | 0.492 | 0.53580 |
| 9 | 22134253 | rs10811662 | A | G | -0.015 | 0.003 | 4.60002e-09 | 0.0001 | 34.354 | -0.001 | 0.019 | 0.968 | 0.16800 |
| 10 | 65124098 | rs10822163 | G | C | -0.032 | 0.002 | 9.70063e-59 | 0.0005 | 261.137 | -0.009 | 0.013 | 0.513 | 0.48710 |
| 10 | 99793865 | rs10883026 | T | C | -0.014 | 0.002 | 5.50047e-13 | 0.0001 | 52.030 | 0.017 | 0.014 | 0.210 | 0.52780 |
| 11 | 78105879 | rs10899490 | T | C | -0.017 | 0.003 | 2.69998e-10 | 0.0001 | 39.874 | 0.001 | 0.017 | 0.963 | 0.18290 |
| 10 | 74711376 | rs11000468 | T | C | -0.015 | 0.002 | 1.50000e-10 | 0.0001 | 41.085 | -0.013 | 0.014 | 0.326 | 0.31210 |
| 11 | 27694835 | rs11030107 | G | A | 0.016 | 0.002 | 1.20005e-12 | 0.0001 | 50.521 | -0.013 | 0.015 | 0.392 | 0.23460 |
| 17 | 1618363 | rs11078597 | C | T | 0.019 | 0.003 | 5.70033e-14 | 0.0001 | 56.462 | 0.013 | 0.017 | 0.434 | 0.16900 |
| 4 | 157682598 | rs11100083 | C | T | -0.016 | 0.002 | 1.50003e-11 | 0.0001 | 45.493 | -0.010 | 0.017 | 0.568 | 0.25550 |
| 1 | 219637671 | rs11118310 | T | A | 0.019 | 0.002 | 1.10002e-21 | 0.0002 | 91.446 | -0.013 | 0.014 | 0.349 | 0.61030 |
| 1 | 230301811 | rs11122450 | G | T | -0.048 | 0.002 | 1.30017e-123 | 0.0011 | 559.145 | 0.010 | 0.013 | 0.452 | 0.60930 |
| 3 | 196187927 | rs11185542 | C | G | -0.013 | 0.002 | 1.60000e-08 | 0.0001 | 31.883 | 0.004 | 0.014 | 0.796 | 0.71270 |
| 10 | 94248310 | rs11187019 | G | A | -0.012 | 0.002 | 6.40000e-09 | 0.0001 | 33.710 | 0.013 | 0.013 | 0.318 | 0.52680 |
| 1 | 40048009 | rs11206374 | A | G | 0.025 | 0.002 | 6.09958e-26 | 0.0002 | 110.937 | 0.016 | 0.016 | 0.313 | 0.22860 |
| 17 | 41388250 | rs112381903 | T | A | 0.023 | 0.004 | 7.49998e-09 | 0.0001 | 33.394 | -0.014 | 0.035 | 0.685 | 0.06461 |
| 1 | 205070573 | rs11240358 | A | G | 0.014 | 0.002 | 2.29985e-11 | 0.0001 | 44.674 | -0.004 | 0.014 | 0.745 | 0.37570 |
| 10 | 134459388 | rs1133400 | G | A | 0.014 | 0.002 | 9.80009e-09 | 0.0001 | 32.871 | -0.011 | 0.018 | 0.525 | 0.21370 |
| 1 | 27021913 | rs114165349 | C | G | 0.082 | 0.007 | 6.29941e-35 | 0.0003 | 151.997 | -0.015 | 0.035 | 0.675 | 0.02187 |
| 11 | 14797227 | rs11600815 | A | G | -0.032 | 0.005 | 2.09991e-12 | 0.0001 | 49.434 | -0.043 | 0.023 | 0.069 | 0.04970 |
| 15 | 40387971 | rs11637681 | G | A | 0.013 | 0.002 | 2.00000e-08 | 0.0001 | 31.503 | -0.007 | 0.016 | 0.679 | 0.26440 |
| 18 | 2846812 | rs11664106 | T | A | -0.013 | 0.002 | 2.39999e-09 | 0.0001 | 35.626 | -0.017 | 0.014 | 0.219 | 0.35590 |
| 19 | 8429323 | rs116843064 | A | G | -0.227 | 0.007 | 1.00000e-200 | 0.0019 | 986.902 | -0.033 | 0.041 | 0.430 | 0.02584 |
| 4 | 77396854 | rs11722924 | C | G | 0.013 | 0.002 | 8.90020e-11 | 0.0001 | 42.044 | 0.014 | 0.013 | 0.276 | 0.51590 |
| 12 | 4328521 | rs117233107 | A | G | -0.073 | 0.009 | 1.80011e-17 | 0.0002 | 72.305 | 0.003 | 0.057 | 0.959 | 0.01491 |
| 11 | 15090794 | rs117287238 | A | G | -0.039 | 0.006 | 1.40001e-10 | 0.0001 | 41.153 | -0.007 | 0.043 | 0.866 | 0.01193 |
| 11 | 380315 | rs117291242 | T | C | 0.030 | 0.005 | 1.70000e-08 | 0.0001 | 31.795 | -0.026 | 0.030 | 0.391 | 0.03678 |
| 19 | 45458212 | rs117316645 | A | G | 0.028 | 0.005 | 2.10000e-08 | 0.0001 | 31.443 | -0.037 | 0.035 | 0.288 | 0.03976 |
| 17 | 42355095 | rs117431393 | G | A | 0.031 | 0.005 | 6.49995e-09 | 0.0001 | 33.689 | 0.014 | 0.027 | 0.612 | 0.04672 |
| 5 | 55990342 | rs11746801 | A | G | -0.012 | 0.002 | 2.69998e-09 | 0.0001 | 35.365 | 0.003 | 0.013 | 0.839 | 0.62330 |
| 2 | 119745127 | rs11904650 | G | A | 0.041 | 0.007 | 2.99999e-09 | 0.0001 | 35.171 | 0.032 | 0.054 | 0.549 | 0.01889 |
| 17 | 47407071 | rs12185242 | C | A | 0.018 | 0.002 | 1.80011e-18 | 0.0002 | 76.873 | -0.008 | 0.013 | 0.546 | 0.45330 |
| 12 | 107186747 | rs12424054 | A | G | 0.019 | 0.002 | 4.49987e-16 | 0.0001 | 66.011 | 0.005 | 0.017 | 0.764 | 0.23060 |
| 15 | 61960302 | rs12440800 | T | A | 0.016 | 0.002 | 2.19989e-12 | 0.0001 | 49.302 | -0.007 | 0.016 | 0.670 | 0.23460 |
| 16 | 56987015 | rs12446515 | T | C | -0.033 | 0.002 | 3.50026e-55 | 0.0005 | 244.835 | -0.003 | 0.015 | 0.835 | 0.29220 |
| 2 | 227232047 | rs12475332 | G | T | -0.014 | 0.002 | 5.19996e-10 | 0.0001 | 38.587 | -0.017 | 0.016 | 0.308 | 0.26640 |
| 4 | 39646631 | rs12504746 | T | C | -0.015 | 0.003 | 1.40001e-09 | 0.0001 | 36.662 | -0.002 | 0.019 | 0.914 | 0.18590 |
| 7 | 106632113 | rs12530679 | G | A | -0.012 | 0.002 | 1.79999e-09 | 0.0001 | 36.173 | -0.010 | 0.013 | 0.451 | 0.49700 |
| 7 | 32262377 | rs12669911 | C | A | -0.012 | 0.002 | 7.90005e-09 | 0.0001 | 33.303 | -0.011 | 0.014 | 0.428 | 0.63020 |
| 14 | 64236191 | rs12880341 | C | T | 0.021 | 0.003 | 1.80011e-14 | 0.0001 | 58.722 | 0.012 | 0.020 | 0.553 | 0.14710 |
| 15 | 41915522 | rs12902047 | C | A | -0.013 | 0.002 | 1.70000e-09 | 0.0001 | 36.299 | -0.028 | 0.014 | 0.045 | 0.32600 |
| 17 | 57906288 | rs1292065 | G | C | -0.014 | 0.002 | 1.89998e-10 | 0.0001 | 40.545 | -0.015 | 0.014 | 0.313 | 0.71470 |
| 16 | 88004092 | rs12926107 | G | A | 0.013 | 0.002 | 2.69998e-10 | 0.0001 | 39.864 | 0.004 | 0.014 | 0.763 | 0.38670 |
| 16 | 15150505 | rs12928099 | A | C | -0.028 | 0.002 | 2.90001e-38 | 0.0003 | 167.268 | -0.007 | 0.015 | 0.634 | 0.28830 |
| 17 | 464962 | rs12948505 | T | C | 0.014 | 0.003 | 3.20000e-08 | 0.0001 | 30.566 | 0.014 | 0.019 | 0.464 | 0.20280 |
| 3 | 87037543 | rs13066793 | G | A | -0.022 | 0.003 | 1.00000e-10 | 0.0001 | 41.821 | -0.016 | 0.024 | 0.519 | 0.08648 |
| 4 | 103188709 | rs13107325 | T | C | 0.030 | 0.004 | 1.69981e-15 | 0.0001 | 63.389 | 0.122 | 0.058 | 0.035 | 0.07952 |
| 4 | 3443931 | rs13108218 | G | A | -0.031 | 0.002 | 9.70063e-50 | 0.0004 | 219.859 | -0.029 | 0.014 | 0.041 | 0.61030 |
| 4 | 89704367 | rs13118477 | A | G | 0.015 | 0.002 | 1.90020e-13 | 0.0001 | 54.073 | 0.001 | 0.013 | 0.941 | 0.41350 |
| 5 | 78531337 | rs1316753 | C | G | -0.015 | 0.002 | 7.00003e-13 | 0.0001 | 51.550 | 0.004 | 0.013 | 0.783 | 0.37870 |
| 8 | 18214065 | rs13264304 | G | C | 0.019 | 0.003 | 8.00018e-12 | 0.0001 | 46.771 | -0.011 | 0.016 | 0.477 | 0.15110 |
| 8 | 72459889 | rs13269725 | G | A | 0.035 | 0.004 | 1.99986e-21 | 0.0002 | 90.374 | -0.002 | 0.035 | 0.962 | 0.05666 |
| 5 | 157925297 | rs13354321 | C | T | -0.015 | 0.002 | 1.90020e-14 | 0.0001 | 58.683 | 0.010 | 0.013 | 0.454 | 0.40260 |
| 2 | 165528876 | rs13389219 | T | C | -0.038 | 0.002 | 6.59933e-77 | 0.0007 | 344.516 | 0.002 | 0.014 | 0.887 | 0.43540 |
| 13 | 29145323 | rs1340819 | C | A | -0.012 | 0.002 | 6.29999e-09 | 0.0001 | 33.729 | 0.006 | 0.014 | 0.670 | 0.33700 |
| 22 | 28807625 | rs134551 | T | C | -0.012 | 0.002 | 3.40001e-08 | 0.0001 | 30.472 | -0.005 | 0.015 | 0.737 | 0.33100 |
| 4 | 124743259 | rs1347188 | G | A | 0.014 | 0.002 | 1.89998e-09 | 0.0001 | 36.099 | 0.010 | 0.015 | 0.490 | 0.23860 |
| 12 | 34821442 | rs139386986 | T | C | -0.022 | 0.004 | 1.09999e-09 | 0.0001 | 37.105 | -0.012 | 0.024 | 0.623 | 0.08250 |
| 10 | 5267191 | rs140107293 | G | A | -0.023 | 0.003 | 1.59993e-16 | 0.0001 | 67.993 | -0.025 | 0.019 | 0.193 | 0.12030 |
| 2 | 171583876 | rs1420384 | T | G | -0.013 | 0.002 | 1.20000e-09 | 0.0001 | 36.906 | -0.002 | 0.014 | 0.905 | 0.65810 |
| 16 | 921179 | rs143076454 | A | G | 0.040 | 0.007 | 3.89996e-08 | 0.0001 | 30.183 | -0.048 | 0.057 | 0.391 | 0.02485 |
| 17 | 41809207 | rs145947882 | C | A | 0.137 | 0.006 | 3.39625e-103 | 0.0010 | 465.273 | 0.004 | 0.039 | 0.927 | 0.03579 |
| 2 | 20368519 | rs1473886 | T | G | -0.018 | 0.002 | 6.90081e-20 | 0.0002 | 83.329 | -0.008 | 0.013 | 0.542 | 0.45730 |
| 4 | 110766064 | rs148827772 | G | A | 0.047 | 0.007 | 5.70033e-11 | 0.0001 | 42.912 | 0.117 | 0.044 | 0.008 | 0.02386 |
| 20 | 32188142 | rs149142833 | T | C | 0.017 | 0.003 | 9.09997e-10 | 0.0001 | 37.514 | -0.020 | 0.018 | 0.261 | 0.18090 |
| 15 | 57023457 | rs150460588 | C | T | 0.033 | 0.005 | 1.59993e-11 | 0.0001 | 45.451 | -0.033 | 0.038 | 0.384 | 0.04175 |
| 11 | 116697081 | rs150555490 | T | C | -0.038 | 0.004 | 2.49977e-19 | 0.0002 | 80.766 | 0.027 | 0.035 | 0.445 | 0.04573 |
| 20 | 569164 | rs151235402 | T | C | 0.052 | 0.008 | 1.50000e-10 | 0.0001 | 40.982 | -0.020 | 0.053 | 0.708 | 0.01690 |
| 8 | 10677981 | rs1544980 | C | T | 0.024 | 0.002 | 7.59976e-22 | 0.0002 | 92.262 | -0.011 | 0.017 | 0.512 | 0.20580 |
| 9 | 1033773 | rs1567353 | G | C | 0.015 | 0.002 | 7.39946e-12 | 0.0001 | 46.920 | -0.006 | 0.014 | 0.658 | 0.33700 |
| 15 | 63792486 | rs17184382 | C | A | -0.022 | 0.002 | 1.10002e-27 | 0.0002 | 118.939 | -0.006 | 0.014 | 0.678 | 0.39960 |
| 2 | 48962291 | rs17326656 | T | G | 0.017 | 0.002 | 7.80010e-14 | 0.0001 | 55.846 | -0.042 | 0.018 | 0.020 | 0.21970 |
| 11 | 61592362 | rs174566 | G | A | 0.049 | 0.002 | 2.79898e-120 | 0.0011 | 543.834 | 0.011 | 0.013 | 0.412 | 0.35590 |
| 6 | 139835498 | rs17585887 | C | T | -0.029 | 0.002 | 1.29987e-45 | 0.0004 | 200.933 | 0.000 | 0.013 | 0.975 | 0.57260 |
| 1 | 154259650 | rs1760801 | A | G | -0.020 | 0.002 | 1.50003e-20 | 0.0002 | 86.345 | -0.010 | 0.014 | 0.466 | 0.32700 |
| 7 | 44199142 | rs1799831 | T | C | 0.025 | 0.003 | 3.69999e-19 | 0.0002 | 80.019 | 0.006 | 0.017 | 0.717 | 0.17000 |
| 6 | 161162290 | rs1835346 | G | A | -0.039 | 0.007 | 2.19999e-09 | 0.0001 | 35.832 | 0.062 | 0.055 | 0.259 | 0.01491 |
| 6 | 161111700 | rs186696265 | T | C | -0.104 | 0.008 | 2.39994e-36 | 0.0003 | 158.530 | 0.041 | 0.053 | 0.435 | 0.01292 |
| 19 | 19396616 | rs188247550 | T | C | -0.134 | 0.009 | 5.90065e-49 | 0.0005 | 216.271 | 0.002 | 0.030 | 0.957 | 0.01292 |
| 5 | 130672958 | rs193735 | A | G | 0.033 | 0.005 | 4.90004e-10 | 0.0001 | 38.734 | -0.051 | 0.026 | 0.050 | 0.03479 |
| 1 | 98478981 | rs1938566 | T | C | -0.021 | 0.003 | 1.99986e-15 | 0.0001 | 63.095 | -0.015 | 0.016 | 0.321 | 0.83100 |
| 15 | 58680954 | rs2043085 | C | T | -0.031 | 0.002 | 1.90020e-51 | 0.0005 | 227.642 | 0.022 | 0.013 | 0.102 | 0.63220 |
| 10 | 94839642 | rs2068888 | A | G | -0.032 | 0.002 | 3.19963e-57 | 0.0005 | 254.197 | -0.014 | 0.013 | 0.299 | 0.48610 |
| 14 | 24545375 | rs2070341 | T | C | 0.011 | 0.002 | 2.80001e-08 | 0.0001 | 30.867 | 0.016 | 0.014 | 0.244 | 0.61930 |
| 22 | 38879010 | rs2071887 | A | T | 0.016 | 0.002 | 7.29962e-15 | 0.0001 | 60.525 | -0.010 | 0.014 | 0.474 | 0.36680 |
| 19 | 49263477 | rs2081194 | C | G | -0.021 | 0.002 | 6.29941e-25 | 0.0002 | 106.299 | -0.007 | 0.014 | 0.594 | 0.63320 |
| 8 | 59388565 | rs2081687 | C | T | -0.026 | 0.002 | 7.59976e-36 | 0.0003 | 156.216 | 0.018 | 0.014 | 0.178 | 0.63520 |
| 20 | 39858545 | rs2092203 | T | C | 0.014 | 0.002 | 6.79986e-12 | 0.0001 | 47.074 | 0.023 | 0.013 | 0.089 | 0.46020 |
| 1 | 40435999 | rs2131311 | G | A | -0.012 | 0.002 | 2.50000e-08 | 0.0001 | 31.079 | -0.006 | 0.015 | 0.695 | 0.72560 |
| 9 | 95283887 | rs2131919 | G | A | 0.017 | 0.003 | 1.00000e-10 | 0.0001 | 41.793 | -0.010 | 0.017 | 0.553 | 0.17200 |
| 1 | 54877103 | rs213494 | T | C | 0.016 | 0.002 | 5.50047e-14 | 0.0001 | 56.556 | 0.003 | 0.015 | 0.832 | 0.68990 |
| 3 | 188439236 | rs2137557 | C | T | 0.012 | 0.002 | 1.60000e-08 | 0.0001 | 31.978 | 0.005 | 0.014 | 0.708 | 0.66300 |
| 18 | 70517838 | rs2187114 | A | G | -0.019 | 0.003 | 2.10000e-08 | 0.0001 | 31.408 | -0.033 | 0.020 | 0.108 | 0.10240 |
| 4 | 55535336 | rs2237029 | A | G | -0.014 | 0.002 | 8.30042e-12 | 0.0001 | 46.685 | 0.006 | 0.014 | 0.685 | 0.59740 |
| 7 | 72856269 | rs2240466 | A | G | -0.123 | 0.003 | 1.00000e-200 | 0.0032 | 1654.249 | -0.029 | 0.020 | 0.141 | 0.11430 |
| 14 | 71541026 | rs2240533 | C | T | -0.013 | 0.002 | 1.60000e-09 | 0.0001 | 36.423 | 0.015 | 0.015 | 0.315 | 0.33700 |
| 9 | 107661129 | rs2244278 | A | C | -0.027 | 0.003 | 1.20005e-18 | 0.0002 | 77.736 | 0.021 | 0.023 | 0.361 | 0.11030 |
| 22 | 38600542 | rs2267373 | T | C | 0.022 | 0.002 | 1.39991e-26 | 0.0002 | 113.795 | 0.012 | 0.013 | 0.357 | 0.57550 |
| 11 | 64021821 | rs2302263 | T | C | 0.044 | 0.003 | 8.00018e-36 | 0.0003 | 156.106 | -0.016 | 0.020 | 0.427 | 0.09940 |
| 17 | 4692559 | rs2304969 | T | G | -0.016 | 0.003 | 1.50000e-08 | 0.0001 | 32.097 | -0.006 | 0.015 | 0.669 | 0.15900 |
| 19 | 35556300 | rs2305746 | G | A | 0.028 | 0.004 | 1.20005e-12 | 0.0001 | 50.539 | 0.026 | 0.027 | 0.341 | 0.93840 |
| 2 | 219184275 | rs2382825 | T | C | -0.013 | 0.002 | 4.79954e-11 | 0.0001 | 43.256 | -0.023 | 0.015 | 0.128 | 0.66000 |
| 8 | 37399290 | rs2407278 | G | A | -0.034 | 0.006 | 5.39995e-09 | 0.0001 | 34.022 | -0.057 | 0.030 | 0.056 | 0.03976 |
| 10 | 113937941 | rs2487294 | T | G | 0.018 | 0.002 | 1.59993e-16 | 0.0001 | 68.041 | -0.017 | 0.014 | 0.235 | 0.69090 |
| 4 | 15102518 | rs2604568 | A | T | 0.012 | 0.002 | 2.80001e-08 | 0.0001 | 30.840 | -0.043 | 0.015 | 0.004 | 0.68590 |
| 7 | 26394297 | rs2699805 | A | G | -0.020 | 0.002 | 5.10035e-23 | 0.0002 | 97.624 | -0.003 | 0.014 | 0.810 | 0.40360 |
| 15 | 39444679 | rs275184 | G | T | -0.017 | 0.003 | 2.90001e-10 | 0.0001 | 39.723 | 0.009 | 0.017 | 0.581 | 0.15900 |
| 10 | 115798895 | rs2773469 | G | A | -0.019 | 0.002 | 5.70033e-17 | 0.0001 | 70.083 | -0.006 | 0.015 | 0.688 | 0.67790 |
| 4 | 40428010 | rs278981 | C | T | 0.013 | 0.002 | 3.89996e-08 | 0.0001 | 30.197 | -0.005 | 0.016 | 0.776 | 0.78430 |
| 13 | 50707087 | rs2812208 | C | G | -0.048 | 0.007 | 3.50026e-12 | 0.0001 | 48.366 | -0.013 | 0.045 | 0.771 | 0.03082 |
| 6 | 32587213 | rs28383314 | C | T | 0.038 | 0.002 | 1.50003e-76 | 0.0007 | 342.908 | -0.009 | 0.014 | 0.515 | 0.66100 |
| 8 | 64664690 | rs28439112 | A | T | 0.013 | 0.002 | 2.69998e-08 | 0.0001 | 30.884 | -0.004 | 0.017 | 0.796 | 0.25350 |
| 16 | 4488191 | rs28577186 | A | G | -0.016 | 0.002 | 1.20005e-14 | 0.0001 | 59.611 | 0.021 | 0.014 | 0.135 | 0.64610 |
| 6 | 31303922 | rs28752924 | G | T | 0.024 | 0.003 | 1.10002e-18 | 0.0002 | 77.870 | -0.011 | 0.013 | 0.417 | 0.06759 |
| 16 | 81534790 | rs2925979 | C | T | -0.032 | 0.002 | 8.49963e-50 | 0.0004 | 220.120 | 0.007 | 0.014 | 0.607 | 0.71170 |
| 16 | 69862109 | rs2937124 | T | C | -0.018 | 0.002 | 8.10028e-18 | 0.0002 | 73.921 | 0.040 | 0.014 | 0.003 | 0.37280 |
| 2 | 227099180 | rs2943645 | T | C | 0.040 | 0.002 | 2.90001e-84 | 0.0007 | 378.320 | -0.008 | 0.014 | 0.558 | 0.62430 |
| 6 | 97029871 | rs2983896 | A | G | 0.014 | 0.002 | 1.20000e-08 | 0.0001 | 32.429 | -0.013 | 0.017 | 0.455 | 0.23660 |
| 8 | 19817476 | rs308 | G | T | -0.159 | 0.007 | 2.60016e-115 | 0.0010 | 520.939 | -0.039 | 0.039 | 0.320 | 0.02485 |
| 3 | 12473045 | rs3103310 | G | A | 0.020 | 0.002 | 1.10002e-17 | 0.0002 | 73.255 | 0.004 | 0.018 | 0.825 | 0.21570 |
| 1 | 118143517 | rs320369 | G | A | -0.013 | 0.002 | 5.19996e-09 | 0.0001 | 34.116 | -0.020 | 0.014 | 0.145 | 0.70280 |
| 5 | 103995368 | rs325485 | G | A | -0.012 | 0.002 | 8.60003e-09 | 0.0001 | 33.125 | -0.037 | 0.014 | 0.008 | 0.62620 |
| 11 | 47259668 | rs326222 | C | T | 0.025 | 0.002 | 1.39991e-31 | 0.0003 | 136.713 | 0.015 | 0.014 | 0.261 | 0.69780 |
| 8 | 19810787 | rs343 | A | C | -0.141 | 0.004 | 1.00000e-200 | 0.0031 | 1535.785 | -0.014 | 0.027 | 0.600 | 0.09344 |
| 16 | 72096227 | rs34682685 | A | G | 0.034 | 0.003 | 5.79963e-25 | 0.0002 | 106.488 | -0.014 | 0.029 | 0.618 | 0.09443 |
| 12 | 46215895 | rs35763453 | C | T | 0.028 | 0.004 | 6.20012e-11 | 0.0001 | 42.748 | 0.024 | 0.036 | 0.506 | 0.04672 |
| 1 | 203511492 | rs36043408 | A | G | -0.013 | 0.002 | 1.09999e-10 | 0.0001 | 41.606 | 0.000 | 0.013 | 0.981 | 0.48110 |
| 2 | 203431804 | rs3731696 | G | A | 0.022 | 0.003 | 5.10035e-13 | 0.0001 | 52.150 | 0.020 | 0.020 | 0.325 | 0.14210 |
| 10 | 17268839 | rs3758413 | C | T | 0.011 | 0.002 | 3.50002e-08 | 0.0001 | 30.392 | -0.011 | 0.013 | 0.398 | 0.44930 |
| 4 | 87985166 | rs3775228 | T | C | 0.034 | 0.002 | 5.79963e-62 | 0.0005 | 275.915 | 0.008 | 0.014 | 0.579 | 0.38070 |
| 8 | 116670347 | rs3808477 | T | C | -0.013 | 0.002 | 1.20000e-09 | 0.0001 | 36.904 | -0.016 | 0.014 | 0.257 | 0.23860 |
| 16 | 29994922 | rs3814883 | T | C | 0.015 | 0.002 | 8.69961e-14 | 0.0001 | 55.638 | -0.032 | 0.013 | 0.019 | 0.46020 |
| 2 | 3642361 | rs3820897 | C | T | 0.020 | 0.003 | 3.50026e-14 | 0.0001 | 57.432 | 0.022 | 0.014 | 0.112 | 0.78130 |
| 21 | 46582100 | rs394872 | T | C | 0.011 | 0.002 | 2.39999e-08 | 0.0001 | 31.163 | 0.022 | 0.013 | 0.105 | 0.54370 |
| 11 | 49161967 | rs3974807 | T | C | 0.016 | 0.003 | 2.80001e-10 | 0.0001 | 39.843 | -0.016 | 0.018 | 0.393 | 0.18790 |
| 2 | 165467068 | rs4128205 | C | A | 0.012 | 0.002 | 8.10009e-09 | 0.0001 | 33.244 | 0.015 | 0.013 | 0.273 | 0.49500 |
| 6 | 20486798 | rs4134963 | T | C | -0.019 | 0.003 | 8.99912e-14 | 0.0001 | 55.584 | -0.025 | 0.017 | 0.140 | 0.20080 |
| 7 | 116486020 | rs41785 | A | C | -0.015 | 0.002 | 8.19974e-14 | 0.0001 | 55.749 | 0.002 | 0.014 | 0.855 | 0.42150 |
| 16 | 85198965 | rs4471666 | G | T | -0.022 | 0.004 | 1.50000e-08 | 0.0001 | 32.058 | 0.018 | 0.024 | 0.446 | 0.08250 |
| 2 | 27598097 | rs4665972 | C | T | -0.100 | 0.002 | 1.00000e-200 | 0.0048 | 2424.998 | 0.006 | 0.014 | 0.636 | 0.58450 |
| 2 | 242395674 | rs4675812 | A | G | -0.014 | 0.002 | 1.50003e-12 | 0.0001 | 50.037 | 0.005 | 0.014 | 0.725 | 0.61430 |
| 7 | 130430930 | rs4731701 | T | C | -0.033 | 0.002 | 2.70023e-60 | 0.0005 | 268.256 | -0.002 | 0.013 | 0.871 | 0.44930 |
| 12 | 57766392 | rs4760254 | C | G | -0.028 | 0.002 | 1.00000e-33 | 0.0003 | 146.518 | 0.007 | 0.016 | 0.672 | 0.19090 |
| 12 | 69732105 | rs4761234 | C | T | -0.014 | 0.002 | 1.99986e-12 | 0.0001 | 49.474 | -0.013 | 0.013 | 0.339 | 0.46920 |
| 12 | 124478637 | rs4765148 | T | G | -0.025 | 0.002 | 9.70063e-32 | 0.0003 | 137.438 | 0.026 | 0.015 | 0.071 | 0.34890 |
| 11 | 116525730 | rs480823 | C | T | 0.156 | 0.004 | 1.00000e-200 | 0.0035 | 1752.523 | -0.006 | 0.018 | 0.752 | 0.10440 |
| 19 | 45416178 | rs483082 | T | G | 0.086 | 0.002 | 1.00000e-200 | 0.0027 | 1351.901 | 0.017 | 0.016 | 0.265 | 0.21870 |
| 19 | 7957481 | rs483808 | T | C | -0.014 | 0.002 | 4.00000e-10 | 0.0001 | 39.098 | 0.027 | 0.014 | 0.054 | 0.69090 |
| 8 | 11517977 | rs4841580 | C | T | -0.025 | 0.002 | 1.39991e-34 | 0.0003 | 150.392 | 0.013 | 0.013 | 0.333 | 0.42350 |
| 17 | 76391454 | rs4969179 | G | T | -0.018 | 0.002 | 2.80027e-18 | 0.0002 | 75.993 | 0.003 | 0.015 | 0.813 | 0.62230 |
| 5 | 67714246 | rs4976033 | G | A | 0.018 | 0.002 | 4.30031e-18 | 0.0002 | 75.186 | 0.018 | 0.014 | 0.185 | 0.40950 |
| 11 | 30504660 | rs499293 | A | G | -0.012 | 0.002 | 1.70000e-08 | 0.0001 | 31.859 | -0.003 | 0.014 | 0.809 | 0.66100 |
| 19 | 45430280 | rs5112 | G | C | 0.068 | 0.002 | 1.00000e-200 | 0.0023 | 1028.752 | -0.013 | 0.014 | 0.325 | 0.55770 |
| 11 | 66074483 | rs551243 | C | G | 0.014 | 0.002 | 4.90004e-13 | 0.0001 | 52.234 | 0.008 | 0.013 | 0.558 | 0.48210 |
| 5 | 173324971 | rs55646464 | T | G | 0.012 | 0.002 | 1.89998e-08 | 0.0001 | 31.559 | 0.022 | 0.014 | 0.097 | 0.28630 |
| 20 | 45599090 | rs55966194 | G | C | -0.018 | 0.002 | 7.10068e-16 | 0.0001 | 65.099 | -0.026 | 0.016 | 0.106 | 0.26440 |
| 10 | 114042643 | rs56397607 | G | A | 0.017 | 0.003 | 1.10002e-11 | 0.0001 | 46.070 | 0.005 | 0.018 | 0.768 | 0.17690 |
| 14 | 23733114 | rs56902258 | A | T | -0.015 | 0.003 | 1.70000e-09 | 0.0001 | 36.316 | -0.014 | 0.017 | 0.411 | 0.20970 |
| 22 | 36012864 | rs5755799 | G | C | 0.012 | 0.002 | 2.99999e-09 | 0.0001 | 35.198 | -0.007 | 0.013 | 0.599 | 0.44430 |
| 9 | 15305378 | rs581080 | C | G | 0.018 | 0.003 | 1.10002e-11 | 0.0001 | 46.078 | -0.019 | 0.019 | 0.326 | 0.81710 |
| 19 | 19379549 | rs58542926 | T | C | -0.103 | 0.004 | 5.29663e-164 | 0.0015 | 744.854 | -0.014 | 0.027 | 0.602 | 0.06759 |
| 20 | 38547459 | rs6028716 | A | G | -0.013 | 0.002 | 2.00000e-08 | 0.0001 | 31.541 | 0.007 | 0.017 | 0.684 | 0.23560 |
| 20 | 44551855 | rs6073958 | C | T | 0.056 | 0.002 | 1.59956e-110 | 0.0010 | 498.928 | 0.005 | 0.017 | 0.793 | 0.22070 |
| 17 | 65892343 | rs60856912 | T | G | 0.025 | 0.003 | 4.79954e-20 | 0.0002 | 84.075 | 0.052 | 0.016 | 0.001 | 0.18190 |
| 1 | 221001142 | rs61830291 | C | A | 0.029 | 0.003 | 1.80011e-17 | 0.0001 | 72.380 | -0.028 | 0.021 | 0.187 | 0.08648 |
| 11 | 116596309 | rs61905078 | C | A | 0.200 | 0.004 | 1.00000e-200 | 0.0055 | 2774.643 | 0.017 | 0.022 | 0.441 | 0.07753 |
| 14 | 100765823 | rs61993685 | C | T | -0.023 | 0.004 | 3.89996e-10 | 0.0001 | 39.176 | -0.003 | 0.024 | 0.887 | 0.05666 |
| 19 | 33891013 | rs62102718 | T | A | 0.020 | 0.002 | 3.90032e-20 | 0.0002 | 84.487 | -0.012 | 0.015 | 0.420 | 0.27930 |
| 19 | 7220013 | rs62112763 | G | C | 0.019 | 0.002 | 5.60015e-22 | 0.0002 | 92.874 | -0.008 | 0.013 | 0.558 | 0.43740 |
| 19 | 48341814 | rs62128802 | T | C | -0.016 | 0.003 | 7.39997e-10 | 0.0001 | 37.923 | -0.034 | 0.018 | 0.051 | 0.16700 |
| 2 | 50731010 | rs62135012 | A | G | -0.012 | 0.002 | 1.50000e-08 | 0.0001 | 32.054 | -0.003 | 0.013 | 0.800 | 0.39660 |
| 3 | 150066540 | rs62271373 | A | T | 0.042 | 0.004 | 7.80010e-23 | 0.0002 | 96.769 | 0.029 | 0.032 | 0.367 | 0.06163 |
| 3 | 156810578 | rs62274099 | T | C | 0.012 | 0.002 | 2.30001e-09 | 0.0001 | 35.661 | 0.005 | 0.014 | 0.722 | 0.41050 |
| 5 | 176750688 | rs62397245 | G | C | 0.015 | 0.002 | 3.89996e-10 | 0.0001 | 39.151 | 0.022 | 0.015 | 0.154 | 0.23760 |
| 6 | 107437166 | rs62427982 | T | C | -0.013 | 0.002 | 4.20001e-10 | 0.0001 | 39.007 | -0.024 | 0.014 | 0.087 | 0.30320 |
| 7 | 130583442 | rs62473520 | C | T | -0.021 | 0.004 | 2.80001e-08 | 0.0001 | 30.813 | 0.036 | 0.027 | 0.174 | 0.07256 |
| 2 | 161340270 | rs6432622 | G | A | -0.011 | 0.002 | 3.79997e-08 | 0.0001 | 30.230 | -0.033 | 0.013 | 0.014 | 0.45920 |
| 18 | 289209 | rs6506033 | T | C | -0.023 | 0.004 | 2.50000e-09 | 0.0001 | 35.566 | -0.039 | 0.022 | 0.070 | 0.08946 |
| 21 | 40553845 | rs6517522 | C | T | -0.013 | 0.002 | 9.39940e-11 | 0.0001 | 41.940 | -0.010 | 0.014 | 0.466 | 0.43840 |
| 4 | 100054827 | rs6532798 | T | C | 0.014 | 0.002 | 1.79999e-10 | 0.0001 | 40.638 | 0.012 | 0.014 | 0.405 | 0.69680 |
| 13 | 74188935 | rs6562773 | G | A | -0.012 | 0.002 | 2.00000e-09 | 0.0001 | 35.943 | -0.018 | 0.013 | 0.183 | 0.49010 |
| 14 | 52480621 | rs6572807 | G | A | 0.012 | 0.002 | 2.90001e-08 | 0.0001 | 30.773 | -0.006 | 0.015 | 0.675 | 0.25650 |
| 2 | 37095139 | rs6752845 | C | G | -0.014 | 0.002 | 6.09958e-12 | 0.0001 | 47.296 | 0.020 | 0.013 | 0.140 | 0.43240 |
| 2 | 21231524 | rs676210 | A | G | -0.074 | 0.002 | 7.09578e-198 | 0.0018 | 900.649 | 0.028 | 0.015 | 0.058 | 0.21770 |
| 3 | 24520283 | rs6792725 | G | A | -0.015 | 0.002 | 6.20012e-12 | 0.0001 | 47.263 | -0.015 | 0.013 | 0.253 | 0.67100 |
| 12 | 21343886 | rs67981690 | G | A | 0.030 | 0.003 | 8.30042e-24 | 0.0002 | 101.194 | -0.015 | 0.017 | 0.359 | 0.14210 |
| 3 | 52516293 | rs6800707 | G | C | 0.030 | 0.003 | 5.30029e-32 | 0.0003 | 138.614 | 0.014 | 0.023 | 0.530 | 0.83800 |
| 3 | 69879670 | rs6805924 | T | G | 0.011 | 0.002 | 4.49997e-08 | 0.0001 | 29.905 | 0.005 | 0.013 | 0.712 | 0.41250 |
| 3 | 135956305 | rs684773 | C | A | 0.029 | 0.002 | 2.49977e-35 | 0.0003 | 153.847 | -0.041 | 0.019 | 0.026 | 0.76540 |
| 5 | 156390297 | rs6882076 | C | T | 0.033 | 0.002 | 4.90004e-58 | 0.0005 | 257.891 | -0.002 | 0.014 | 0.896 | 0.64710 |
| 6 | 127435106 | rs6916318 | T | A | 0.027 | 0.002 | 4.19952e-41 | 0.0004 | 180.273 | 0.041 | 0.013 | 0.002 | 0.48610 |
| 9 | 86583076 | rs696825 | T | C | -0.020 | 0.002 | 7.50067e-19 | 0.0002 | 78.619 | 0.019 | 0.014 | 0.188 | 0.28630 |
| 1 | 93836218 | rs698927 | C | A | -0.018 | 0.003 | 9.20026e-13 | 0.0001 | 51.013 | 0.003 | 0.017 | 0.847 | 0.16900 |
| 8 | 126475770 | rs6999569 | G | A | -0.086 | 0.002 | 1.00000e-200 | 0.0037 | 1889.263 | 0.002 | 0.013 | 0.887 | 0.45330 |
| 8 | 19873502 | rs7000494 | C | G | 0.137 | 0.006 | 7.99834e-121 | 0.0011 | 546.317 | 0.020 | 0.048 | 0.671 | 0.02783 |
| 10 | 81096071 | rs7077812 | C | T | 0.014 | 0.003 | 1.40001e-08 | 0.0001 | 32.234 | -0.005 | 0.017 | 0.779 | 0.19780 |
| 12 | 20473758 | rs7134375 | A | C | -0.017 | 0.002 | 1.20005e-17 | 0.0001 | 73.106 | 0.012 | 0.014 | 0.374 | 0.40160 |
| 12 | 22765676 | rs7135509 | C | T | -0.012 | 0.002 | 4.09996e-08 | 0.0001 | 30.091 | -0.018 | 0.014 | 0.203 | 0.29820 |
| 19 | 56087586 | rs71368855 | T | C | 0.026 | 0.003 | 7.80010e-17 | 0.0001 | 69.467 | -0.007 | 0.026 | 0.795 | 0.10930 |
| 13 | 114544024 | rs7140110 | C | T | 0.028 | 0.002 | 1.20005e-38 | 0.0003 | 169.021 | -0.028 | 0.015 | 0.057 | 0.31510 |
| 7 | 1010801 | rs71538127 | G | C | 0.018 | 0.003 | 6.19998e-09 | 0.0001 | 33.769 | -0.015 | 0.023 | 0.502 | 0.11330 |
| 4 | 18034463 | rs71603401 | G | A | 0.026 | 0.003 | 1.10002e-19 | 0.0002 | 82.496 | 0.011 | 0.025 | 0.664 | 0.10830 |
| 17 | 17458353 | rs7215055 | G | A | 0.039 | 0.004 | 2.29985e-21 | 0.0002 | 90.099 | 0.039 | 0.025 | 0.113 | 0.07455 |
| 18 | 21120035 | rs7239575 | C | T | -0.016 | 0.002 | 6.79986e-16 | 0.0001 | 65.179 | -0.013 | 0.013 | 0.312 | 0.45920 |
| 5 | 153800513 | rs7244 | A | G | 0.015 | 0.003 | 6.40000e-09 | 0.0001 | 33.697 | 0.002 | 0.017 | 0.904 | 0.18090 |
| 7 | 73123473 | rs72555385 | G | A | 0.065 | 0.005 | 1.69981e-45 | 0.0004 | 200.442 | 0.011 | 0.045 | 0.801 | 0.06163 |
| 5 | 55783832 | rs72644085 | C | T | -0.020 | 0.003 | 3.19963e-12 | 0.0001 | 48.586 | -0.007 | 0.020 | 0.720 | 0.15510 |
| 20 | 56113783 | rs7274718 | A | G | 0.016 | 0.002 | 3.80014e-15 | 0.0001 | 61.777 | -0.016 | 0.013 | 0.226 | 0.61030 |
| 2 | 28489549 | rs72784786 | A | G | 0.026 | 0.004 | 2.59998e-10 | 0.0001 | 39.974 | 0.040 | 0.026 | 0.128 | 0.06262 |
| 5 | 132444128 | rs72801474 | A | G | -0.031 | 0.003 | 3.59998e-19 | 0.0002 | 80.061 | -0.042 | 0.029 | 0.147 | 0.06859 |
| 6 | 43804571 | rs729761 | G | T | 0.018 | 0.002 | 9.49948e-16 | 0.0001 | 64.521 | 0.009 | 0.015 | 0.559 | 0.72660 |
| 6 | 160562481 | rs73025562 | A | G | 0.014 | 0.002 | 2.00000e-09 | 0.0001 | 35.926 | -0.012 | 0.014 | 0.405 | 0.25450 |
| 12 | 6706744 | rs7308584 | A | G | 0.015 | 0.003 | 5.30005e-09 | 0.0001 | 34.086 | -0.023 | 0.015 | 0.130 | 0.16900 |
| 13 | 114524944 | rs7400002 | G | A | 0.014 | 0.002 | 3.59998e-09 | 0.0001 | 34.845 | 0.004 | 0.015 | 0.812 | 0.24160 |
| 1 | 61705898 | rs74090351 | A | G | -0.025 | 0.004 | 2.10000e-10 | 0.0001 | 40.363 | 0.016 | 0.025 | 0.507 | 0.06859 |
| 16 | 960725 | rs742036 | A | G | -0.014 | 0.002 | 2.49977e-12 | 0.0001 | 49.037 | -0.017 | 0.014 | 0.234 | 0.37570 |
| 2 | 59313974 | rs7424120 | T | C | -0.012 | 0.002 | 1.20000e-09 | 0.0001 | 37.025 | -0.008 | 0.014 | 0.544 | 0.61730 |
| 11 | 18301915 | rs75268115 | G | A | -0.021 | 0.004 | 6.80002e-09 | 0.0001 | 33.577 | 0.036 | 0.023 | 0.114 | 0.11330 |
| 8 | 19871080 | rs75609851 | A | G | -0.199 | 0.010 | 1.59993e-87 | 0.0008 | 393.311 | -0.028 | 0.059 | 0.636 | 0.01392 |
| 7 | 150296604 | rs75721796 | A | G | 0.021 | 0.002 | 1.20005e-17 | 0.0001 | 73.219 | -0.002 | 0.014 | 0.871 | 0.19880 |
| 12 | 62787720 | rs75942983 | T | A | -0.020 | 0.004 | 2.50000e-08 | 0.0001 | 31.059 | -0.009 | 0.031 | 0.781 | 0.10240 |
| 6 | 161004972 | rs77009508 | G | A | 0.045 | 0.004 | 1.80011e-32 | 0.0003 | 140.807 | -0.010 | 0.028 | 0.714 | 0.05467 |
| 5 | 90255685 | rs7704653 | G | A | 0.016 | 0.002 | 2.29985e-12 | 0.0001 | 49.203 | -0.010 | 0.015 | 0.527 | 0.71770 |
| 5 | 112490629 | rs7714361 | C | A | 0.014 | 0.002 | 4.39997e-09 | 0.0001 | 34.437 | 0.006 | 0.015 | 0.702 | 0.20870 |
| 5 | 53310139 | rs7735249 | G | C | 0.027 | 0.003 | 1.90020e-17 | 0.0001 | 72.270 | 0.005 | 0.020 | 0.799 | 0.11430 |
| 7 | 72139093 | rs7786339 | T | C | 0.016 | 0.003 | 7.59994e-10 | 0.0001 | 37.864 | 0.014 | 0.017 | 0.417 | 0.18390 |
| 2 | 219699999 | rs78058190 | A | G | 0.082 | 0.005 | 1.69981e-57 | 0.0006 | 255.394 | 0.020 | 0.024 | 0.413 | 0.06262 |
| 9 | 13723175 | rs7847285 | C | T | -0.012 | 0.002 | 1.00000e-08 | 0.0001 | 32.833 | 0.007 | 0.014 | 0.620 | 0.61730 |
| 11 | 116598065 | rs78484485 | A | G | -0.076 | 0.004 | 1.20005e-66 | 0.0006 | 297.378 | -0.005 | 0.023 | 0.826 | 0.06064 |
| 6 | 7249460 | rs78588343 | A | G | -0.016 | 0.003 | 2.50000e-09 | 0.0001 | 35.550 | -0.007 | 0.016 | 0.640 | 0.15610 |
| 9 | 123375589 | rs7861679 | T | C | 0.012 | 0.002 | 1.79999e-08 | 0.0001 | 31.697 | 0.001 | 0.015 | 0.942 | 0.69780 |
| 3 | 172294500 | rs79287178 | A | G | 0.050 | 0.006 | 7.19946e-17 | 0.0002 | 69.607 | -0.040 | 0.033 | 0.226 | 0.02584 |
| 11 | 116902639 | rs79357714 | G | A | -0.029 | 0.005 | 1.20000e-09 | 0.0001 | 36.910 | -0.012 | 0.033 | 0.718 | 0.03280 |
| 11 | 13356030 | rs7947951 | G | A | 0.019 | 0.002 | 1.39991e-19 | 0.0002 | 81.936 | 0.026 | 0.013 | 0.048 | 0.70280 |
| 10 | 52373245 | rs80276949 | A | G | 0.046 | 0.007 | 8.00018e-12 | 0.0001 | 46.778 | 0.079 | 0.061 | 0.192 | 0.02087 |
| 19 | 57488423 | rs8102873 | T | C | 0.012 | 0.002 | 9.40005e-10 | 0.0001 | 37.454 | 0.025 | 0.013 | 0.056 | 0.57650 |
| 20 | 62711459 | rs8126001 | T | C | -0.016 | 0.002 | 2.39994e-16 | 0.0001 | 67.273 | -0.004 | 0.013 | 0.755 | 0.48210 |
| 7 | 5574239 | rs852388 | C | G | 0.016 | 0.002 | 1.60000e-10 | 0.0001 | 40.928 | 0.009 | 0.020 | 0.672 | 0.17400 |
| 18 | 19911690 | rs867939 | A | G | -0.014 | 0.002 | 1.80011e-11 | 0.0001 | 45.130 | -0.013 | 0.013 | 0.344 | 0.58150 |
| 1 | 10796866 | rs880315 | C | T | -0.012 | 0.002 | 2.19999e-08 | 0.0001 | 31.276 | 0.015 | 0.013 | 0.263 | 0.35690 |
| 18 | 57861663 | rs921971 | C | T | 0.016 | 0.002 | 4.00037e-12 | 0.0001 | 48.122 | -0.013 | 0.017 | 0.438 | 0.26840 |
| 6 | 27303745 | rs9368503 | T | A | -0.012 | 0.002 | 1.79999e-09 | 0.0001 | 36.203 | -0.015 | 0.014 | 0.274 | 0.52980 |
| 6 | 133797208 | rs9373056 | T | C | -0.012 | 0.002 | 7.19996e-09 | 0.0001 | 33.472 | -0.008 | 0.015 | 0.589 | 0.31310 |
| 6 | 140611419 | rs9376511 | G | A | -0.015 | 0.002 | 3.20000e-10 | 0.0001 | 39.532 | 0.022 | 0.016 | 0.189 | 0.20680 |
| 1 | 172355276 | rs9425589 | A | G | -0.014 | 0.002 | 6.20012e-12 | 0.0001 | 47.261 | -0.016 | 0.013 | 0.232 | 0.58250 |
| 1 | 62904575 | rs9436661 | G | T | -0.078 | 0.002 | 1.00000e-200 | 0.0028 | 1399.394 | 0.021 | 0.015 | 0.171 | 0.30520 |
| 6 | 109189021 | rs9480889 | G | C | 0.016 | 0.002 | 1.29987e-11 | 0.0001 | 45.779 | 0.010 | 0.019 | 0.590 | 0.77630 |
| 2 | 121309231 | rs954244 | G | C | 0.015 | 0.002 | 1.59993e-11 | 0.0001 | 45.396 | 0.026 | 0.017 | 0.121 | 0.22370 |
| 13 | 95253131 | rs9561643 | C | A | 0.017 | 0.002 | 6.49980e-15 | 0.0001 | 60.734 | -0.001 | 0.014 | 0.952 | 0.32500 |
| 13 | 99245866 | rs9584870 | C | T | -0.012 | 0.002 | 4.30002e-09 | 0.0001 | 34.496 | -0.044 | 0.014 | 0.002 | 0.36180 |
| 5 | 140888248 | rs970069 | T | C | 0.016 | 0.002 | 2.60016e-11 | 0.0001 | 44.455 | 0.005 | 0.017 | 0.753 | 0.20080 |
| 3 | 37025661 | rs9831084 | C | T | -0.012 | 0.002 | 2.90001e-09 | 0.0001 | 35.236 | -0.007 | 0.014 | 0.608 | 0.44140 |
| 3 | 127397553 | rs9859117 | C | G | 0.015 | 0.002 | 2.39999e-09 | 0.0001 | 35.610 | 0.018 | 0.015 | 0.216 | 0.22370 |
| 17 | 73364216 | rs9889402 | A | G | 0.012 | 0.002 | 3.59998e-08 | 0.0001 | 30.358 | 0.005 | 0.016 | 0.768 | 0.69780 |
| 17 | 7440984 | rs9902027 | T | C | -0.015 | 0.002 | 2.10000e-10 | 0.0001 | 40.359 | -0.011 | 0.015 | 0.471 | 0.76240 |
| 6 | 43757896 | rs998584 | A | C | 0.040 | 0.002 | 2.19989e-90 | 0.0008 | 406.426 | -0.004 | 0.013 | 0.791 | 0.50400 |

**Supplementary table 2.15. Detailed information of instrumental SNPs for LDL-c in the analysis of PPD.**

| **Chr** | **Position** | **SNP** | **EA** | **OA** | **beta.exposure** | **se.exposure** | **pval.exposure** | **R2** | **F_val** | **beta.outcome** | **se.outcome** | **pval.outcome** | **Eaf.outcome** |
| --- | --- | --- | --- | --- | --- | --- | --- | --- | --- | --- | --- | --- | --- |
| 20 | 34147630 | rs1010759 | A | G | -0.023 | 0.003 | 1.50003e-14 | 0.0001 | 59.124 | 0.022 | 0.023 | 0.336 | 0.14910 |
| 5 | 131744574 | rs1016988 | C | T | -0.017 | 0.003 | 4.79954e-11 | 0.0001 | 43.271 | -0.037 | 0.016 | 0.022 | 0.20180 |
| 7 | 100532540 | rs10231941 | C | T | 0.020 | 0.003 | 3.29989e-13 | 0.0001 | 53.016 | -0.007 | 0.017 | 0.694 | 0.16200 |
| 9 | 139320069 | rs10448340 | G | T | -0.015 | 0.002 | 1.69981e-11 | 0.0001 | 45.304 | 0.027 | 0.014 | 0.064 | 0.29720 |
| 11 | 18664241 | rs10832963 | G | T | 0.017 | 0.002 | 5.10035e-13 | 0.0001 | 52.162 | -0.011 | 0.014 | 0.421 | 0.71970 |
| 1 | 234734956 | rs10910476 | T | C | 0.012 | 0.002 | 3.69999e-09 | 0.0001 | 34.767 | 0.013 | 0.013 | 0.324 | 0.54570 |
| 10 | 18720845 | rs11014204 | T | C | 0.014 | 0.002 | 3.20000e-09 | 0.0001 | 35.038 | -0.004 | 0.016 | 0.785 | 0.26740 |
| 12 | 121423386 | rs11065385 | G | A | -0.024 | 0.002 | 1.99986e-27 | 0.0003 | 117.695 | -0.022 | 0.014 | 0.121 | 0.66700 |
| 4 | 81167309 | rs11099097 | T | C | -0.018 | 0.002 | 2.80027e-15 | 0.0001 | 62.406 | 0.003 | 0.014 | 0.820 | 0.26540 |
| 11 | 103870755 | rs11226108 | C | G | -0.016 | 0.003 | 1.60000e-09 | 0.0001 | 36.469 | -0.007 | 0.017 | 0.687 | 0.19580 |
| 3 | 132217703 | rs113177823 | A | G | -0.041 | 0.005 | 1.00000e-18 | 0.0002 | 78.054 | 0.085 | 0.027 | 0.002 | 0.05070 |
| 1 | 27021913 | rs114165349 | C | G | 0.056 | 0.007 | 6.20012e-16 | 0.0001 | 65.358 | -0.015 | 0.035 | 0.675 | 0.02187 |
| 2 | 234665498 | rs11568318 | A | C | 0.026 | 0.004 | 7.79992e-10 | 0.0001 | 37.815 | 0.050 | 0.039 | 0.197 | 0.04871 |
| 1 | 55505647 | rs11591147 | T | G | -0.348 | 0.008 | 1.00000e-200 | 0.0042 | 1930.426 | 0.065 | 0.035 | 0.063 | 0.02087 |
| 11 | 5701074 | rs11601507 | A | C | 0.032 | 0.004 | 1.80011e-15 | 0.0001 | 63.223 | -0.048 | 0.025 | 0.051 | 0.06759 |
| 14 | 24871926 | rs11621792 | T | C | 0.019 | 0.002 | 1.10002e-19 | 0.0002 | 82.335 | 0.050 | 0.014 | 0.000 | 0.47020 |
| 5 | 52095024 | rs116734477 | T | C | -0.047 | 0.005 | 3.19963e-19 | 0.0002 | 80.319 | 0.006 | 0.039 | 0.878 | 0.02883 |
| 6 | 160922870 | rs117733303 | G | A | 0.084 | 0.008 | 2.39994e-27 | 0.0003 | 117.318 | 0.070 | 0.064 | 0.279 | 0.01193 |
| 6 | 160985526 | rs118039278 | A | G | 0.084 | 0.004 | 1.79887e-102 | 0.0010 | 461.929 | -0.009 | 0.032 | 0.768 | 0.07356 |
| 1 | 62958658 | rs1183851 | C | T | 0.024 | 0.002 | 4.49987e-30 | 0.0003 | 129.827 | -0.025 | 0.013 | 0.060 | 0.43540 |
| 1 | 16512586 | rs12078100 | G | C | 0.013 | 0.002 | 1.00000e-09 | 0.0001 | 37.240 | 0.007 | 0.015 | 0.648 | 0.61130 |
| 22 | 50853626 | rs12162782 | G | T | 0.013 | 0.002 | 3.50002e-09 | 0.0001 | 34.904 | 0.008 | 0.013 | 0.548 | 0.34000 |
| 6 | 160543148 | rs12208357 | T | C | 0.057 | 0.004 | 5.40008e-44 | 0.0004 | 193.533 | -0.012 | 0.029 | 0.682 | 0.06262 |
| 10 | 124705307 | rs12246352 | G | A | 0.026 | 0.003 | 6.00067e-14 | 0.0001 | 56.356 | 0.025 | 0.028 | 0.372 | 0.10040 |
| 4 | 100239319 | rs1229984 | C | T | 0.053 | 0.006 | 1.00000e-16 | 0.0001 | 68.919 | 0.180 | 0.096 | 0.060 | 0.97120 |
| 16 | 11706100 | rs12445804 | A | G | 0.023 | 0.004 | 9.49992e-09 | 0.0001 | 32.945 | -0.035 | 0.021 | 0.101 | 0.06759 |
| 2 | 64928603 | rs12471768 | C | T | 0.014 | 0.002 | 2.50000e-09 | 0.0001 | 35.545 | -0.024 | 0.016 | 0.134 | 0.71370 |
| 2 | 216300185 | rs1250258 | T | C | 0.014 | 0.002 | 5.99998e-09 | 0.0001 | 33.845 | 0.006 | 0.016 | 0.712 | 0.76640 |
| 2 | 27730940 | rs1260326 | C | T | -0.035 | 0.002 | 5.10035e-60 | 0.0006 | 266.994 | 0.004 | 0.014 | 0.762 | 0.58950 |
| 5 | 74656539 | rs12916 | C | T | 0.062 | 0.002 | 1.69824e-187 | 0.0019 | 852.850 | 0.010 | 0.013 | 0.461 | 0.41250 |
| 2 | 44327463 | rs13020929 | A | G | 0.015 | 0.002 | 3.40017e-12 | 0.0001 | 48.456 | 0.008 | 0.013 | 0.524 | 0.47420 |
| 3 | 12327431 | rs13076933 | G | T | -0.021 | 0.002 | 1.59993e-18 | 0.0002 | 77.143 | 0.018 | 0.015 | 0.221 | 0.25650 |
| 4 | 103188709 | rs13107325 | T | C | -0.025 | 0.004 | 3.79997e-10 | 0.0001 | 39.238 | 0.122 | 0.058 | 0.035 | 0.07952 |
| 4 | 3443931 | rs13108218 | G | A | -0.018 | 0.002 | 1.80011e-16 | 0.0001 | 67.824 | -0.029 | 0.014 | 0.041 | 0.61030 |
| 4 | 7212389 | rs13121616 | G | A | 0.013 | 0.002 | 1.29999e-08 | 0.0001 | 32.294 | 0.004 | 0.015 | 0.805 | 0.70380 |
| 8 | 9367743 | rs1350559 | G | C | 0.014 | 0.002 | 1.09999e-10 | 0.0001 | 41.601 | -0.001 | 0.013 | 0.914 | 0.37280 |
| 19 | 11187324 | rs143020224 | G | C | -0.169 | 0.003 | 1.00000e-200 | 0.0060 | 2773.120 | -0.021 | 0.022 | 0.336 | 0.11530 |
| 2 | 118845121 | rs150474434 | A | G | -0.035 | 0.003 | 1.29987e-23 | 0.0002 | 100.323 | -0.058 | 0.025 | 0.019 | 0.08946 |
| 19 | 45231821 | rs1551891 | A | G | -0.174 | 0.004 | 1.00000e-200 | 0.0048 | 2250.135 | -0.018 | 0.026 | 0.488 | 0.06561 |
| 1 | 93034023 | rs1556562 | T | G | 0.019 | 0.002 | 2.09991e-14 | 0.0001 | 58.423 | 0.007 | 0.016 | 0.639 | 0.80720 |
| 2 | 121306440 | rs17050272 | A | G | -0.021 | 0.002 | 2.49977e-22 | 0.0002 | 94.452 | -0.030 | 0.013 | 0.026 | 0.45030 |
| 11 | 61588305 | rs174564 | G | A | -0.032 | 0.002 | 4.49987e-48 | 0.0005 | 212.221 | 0.011 | 0.013 | 0.431 | 0.35290 |
| 10 | 71094504 | rs17476364 | C | T | -0.022 | 0.003 | 1.20000e-10 | 0.0001 | 41.502 | 0.040 | 0.028 | 0.162 | 0.09940 |
| 20 | 62896110 | rs17569873 | T | C | 0.017 | 0.003 | 4.79954e-11 | 0.0001 | 43.244 | -0.025 | 0.019 | 0.204 | 0.19380 |
| 16 | 56991363 | rs183130 | T | C | -0.033 | 0.002 | 2.09991e-49 | 0.0005 | 218.312 | -0.002 | 0.015 | 0.882 | 0.29320 |
| 20 | 39179822 | rs1883711 | C | G | 0.103 | 0.006 | 7.50067e-64 | 0.0006 | 284.595 | -0.002 | 0.028 | 0.954 | 0.03976 |
| 15 | 58680954 | rs2043085 | C | T | -0.017 | 0.002 | 1.90020e-15 | 0.0001 | 63.135 | 0.022 | 0.013 | 0.102 | 0.63220 |
| 9 | 107586753 | rs2066714 | C | T | 0.021 | 0.003 | 1.39991e-11 | 0.0001 | 45.671 | 0.013 | 0.023 | 0.552 | 0.13520 |
| 10 | 94839642 | rs2068888 | A | G | -0.019 | 0.002 | 4.60045e-20 | 0.0002 | 84.126 | -0.014 | 0.013 | 0.299 | 0.48610 |
| 7 | 44582331 | rs2073547 | G | A | 0.036 | 0.003 | 2.29985e-40 | 0.0004 | 176.896 | -0.012 | 0.014 | 0.391 | 0.19480 |
| 12 | 50650057 | rs2160994 | C | T | 0.018 | 0.002 | 6.29941e-17 | 0.0002 | 69.878 | -0.010 | 0.014 | 0.466 | 0.62030 |
| 13 | 32959199 | rs2238162 | T | C | -0.017 | 0.002 | 2.80027e-15 | 0.0001 | 62.424 | 0.020 | 0.013 | 0.145 | 0.51590 |
| 10 | 113921354 | rs2250802 | A | G | -0.018 | 0.002 | 6.09958e-15 | 0.0001 | 60.856 | -0.017 | 0.014 | 0.246 | 0.69180 |
| 20 | 62373983 | rs2256814 | A | G | 0.015 | 0.003 | 6.10000e-09 | 0.0001 | 33.800 | -0.020 | 0.017 | 0.240 | 0.16800 |
| 2 | 169830328 | rs2287622 | G | A | -0.021 | 0.002 | 2.90001e-23 | 0.0002 | 98.728 | -0.011 | 0.013 | 0.423 | 0.60440 |
| 13 | 111035483 | rs2391825 | A | G | -0.013 | 0.002 | 2.59998e-08 | 0.0001 | 30.962 | -0.022 | 0.016 | 0.181 | 0.29030 |
| 20 | 17844684 | rs2618566 | T | G | -0.025 | 0.002 | 9.60064e-30 | 0.0003 | 128.313 | 0.031 | 0.014 | 0.029 | 0.61630 |
| 1 | 220970028 | rs2642438 | G | A | 0.025 | 0.002 | 8.00018e-29 | 0.0003 | 124.097 | -0.016 | 0.015 | 0.267 | 0.72170 |
| 8 | 116667634 | rs2737265 | G | A | -0.020 | 0.002 | 2.49977e-18 | 0.0002 | 76.237 | -0.016 | 0.014 | 0.272 | 0.23960 |
| 19 | 11227480 | rs2738447 | C | A | 0.042 | 0.002 | 2.90001e-89 | 0.0009 | 401.301 | 0.017 | 0.014 | 0.222 | 0.56460 |
| 9 | 107661742 | rs2740488 | C | A | -0.025 | 0.002 | 1.29987e-26 | 0.0002 | 113.942 | 0.018 | 0.017 | 0.294 | 0.24650 |
| 6 | 127452935 | rs2745353 | T | C | 0.013 | 0.002 | 1.09999e-09 | 0.0001 | 37.139 | -0.036 | 0.013 | 0.007 | 0.48410 |
| 6 | 35045281 | rs2820226 | A | G | -0.013 | 0.002 | 8.60003e-10 | 0.0001 | 37.621 | 0.025 | 0.013 | 0.062 | 0.47810 |
| 7 | 21449451 | rs28406917 | T | C | 0.012 | 0.002 | 1.79999e-08 | 0.0001 | 31.708 | 0.013 | 0.013 | 0.323 | 0.41350 |
| 5 | 71932487 | rs28590710 | A | G | -0.018 | 0.003 | 1.40001e-10 | 0.0001 | 41.116 | 0.006 | 0.016 | 0.686 | 0.16200 |
| 8 | 126500031 | rs28601761 | G | C | -0.062 | 0.002 | 2.89734e-185 | 0.0019 | 842.617 | 0.008 | 0.013 | 0.533 | 0.40760 |
| 8 | 55451193 | rs28615248 | C | T | 0.018 | 0.003 | 2.70023e-12 | 0.0001 | 48.920 | 0.018 | 0.016 | 0.258 | 0.16100 |
| 1 | 235109214 | rs28631087 | C | T | -0.016 | 0.003 | 2.00000e-10 | 0.0001 | 40.440 | 0.014 | 0.018 | 0.439 | 0.19680 |
| 6 | 32585967 | rs3104412 | G | A | -0.019 | 0.002 | 1.80011e-19 | 0.0002 | 81.500 | 0.015 | 0.013 | 0.261 | 0.45530 |
| 16 | 72101525 | rs34042070 | G | C | 0.049 | 0.003 | 2.90001e-73 | 0.0007 | 327.782 | 0.015 | 0.017 | 0.379 | 0.20180 |
| 8 | 41542093 | rs34265667 | A | G | -0.032 | 0.006 | 2.59998e-08 | 0.0001 | 30.995 | -0.070 | 0.054 | 0.189 | 0.02187 |
| 19 | 58665817 | rs35511051 | A | C | -0.022 | 0.003 | 1.10002e-17 | 0.0002 | 73.254 | 0.009 | 0.016 | 0.592 | 0.22070 |
| 12 | 623129 | rs35882350 | G | A | 0.014 | 0.002 | 4.00000e-09 | 0.0001 | 34.615 | 0.000 | 0.015 | 0.977 | 0.25150 |
| 3 | 119536429 | rs3732359 | A | G | -0.017 | 0.003 | 5.70033e-12 | 0.0001 | 47.418 | -0.011 | 0.014 | 0.458 | 0.79720 |
| 9 | 2640759 | rs3780181 | G | A | -0.028 | 0.004 | 2.60016e-11 | 0.0001 | 44.477 | 0.026 | 0.029 | 0.380 | 0.05268 |
| 6 | 116316882 | rs3822855 | T | G | 0.018 | 0.002 | 4.49987e-17 | 0.0002 | 70.530 | -0.011 | 0.013 | 0.398 | 0.42450 |
| 7 | 87074419 | rs4148826 | C | T | -0.016 | 0.003 | 6.69993e-09 | 0.0001 | 33.625 | 0.020 | 0.017 | 0.233 | 0.19180 |
| 19 | 45438643 | rs4263041 | G | A | -0.070 | 0.003 | 4.89779e-171 | 0.0020 | 777.197 | -0.013 | 0.015 | 0.376 | 0.28830 |
| 11 | 126244955 | rs4307732 | A | G | 0.045 | 0.003 | 9.79941e-40 | 0.0004 | 174.017 | -0.030 | 0.018 | 0.093 | 0.10640 |
| 20 | 12958687 | rs438568 | G | A | 0.012 | 0.002 | 5.49997e-09 | 0.0001 | 34.002 | -0.029 | 0.014 | 0.035 | 0.63720 |
| 19 | 11285390 | rs440677 | A | G | -0.016 | 0.002 | 1.50003e-13 | 0.0001 | 54.634 | -0.007 | 0.013 | 0.619 | 0.62920 |
| 2 | 20575012 | rs4666384 | G | A | -0.017 | 0.002 | 1.10002e-13 | 0.0001 | 55.136 | 0.023 | 0.015 | 0.115 | 0.66100 |
| 1 | 55521313 | rs472495 | T | G | 0.043 | 0.002 | 7.29962e-85 | 0.0008 | 381.076 | -0.015 | 0.013 | 0.277 | 0.62520 |
| 16 | 83980529 | rs4782568 | G | C | -0.017 | 0.002 | 4.90004e-15 | 0.0001 | 61.308 | 0.008 | 0.013 | 0.561 | 0.46120 |
| 11 | 65591348 | rs4930163 | A | G | 0.017 | 0.003 | 9.20005e-10 | 0.0001 | 37.477 | 0.002 | 0.019 | 0.923 | 0.15610 |
| 2 | 135632981 | rs4954192 | T | C | 0.015 | 0.002 | 6.70039e-12 | 0.0001 | 47.100 | -0.026 | 0.013 | 0.058 | 0.51290 |
| 1 | 109814880 | rs4970834 | T | C | -0.105 | 0.003 | 1.00000e-200 | 0.0034 | 1541.814 | -0.008 | 0.017 | 0.616 | 0.18590 |
| 19 | 45430280 | rs5112 | G | C | 0.028 | 0.002 | 9.39940e-36 | 0.0004 | 155.790 | -0.013 | 0.014 | 0.325 | 0.55770 |
| 19 | 49206145 | rs516316 | C | G | 0.030 | 0.002 | 1.50003e-46 | 0.0004 | 205.203 | -0.001 | 0.014 | 0.962 | 0.44140 |
| 1 | 234853059 | rs556107 | T | C | 0.035 | 0.002 | 1.20005e-63 | 0.0006 | 283.610 | 0.007 | 0.013 | 0.609 | 0.51890 |
| 1 | 55466303 | rs55637835 | T | C | -0.019 | 0.003 | 8.40001e-09 | 0.0001 | 33.186 | 0.041 | 0.022 | 0.064 | 0.10240 |
| 17 | 7080316 | rs55714927 | T | C | -0.026 | 0.003 | 2.39994e-23 | 0.0002 | 99.071 | -0.015 | 0.015 | 0.310 | 0.18390 |
| 8 | 145031968 | rs55831924 | T | C | 0.016 | 0.002 | 6.89922e-13 | 0.0001 | 51.571 | 0.003 | 0.014 | 0.834 | 0.37870 |
| 7 | 21598753 | rs56130071 | C | G | 0.033 | 0.003 | 4.70002e-39 | 0.0004 | 170.914 | -0.005 | 0.016 | 0.744 | 0.18890 |
| 2 | 3636478 | rs56236159 | G | T | 0.018 | 0.003 | 8.79995e-09 | 0.0001 | 33.082 | -0.018 | 0.015 | 0.226 | 0.15710 |
| 12 | 111973358 | rs597808 | G | A | 0.027 | 0.002 | 2.09991e-38 | 0.0004 | 167.969 | 0.010 | 0.013 | 0.448 | 0.53380 |
| 20 | 43038249 | rs6031587 | T | C | -0.026 | 0.004 | 5.69994e-10 | 0.0001 | 38.424 | -0.022 | 0.026 | 0.406 | 0.06163 |
| 7 | 98874892 | rs60612724 | G | A | 0.032 | 0.005 | 2.30001e-09 | 0.0001 | 35.714 | 0.065 | 0.038 | 0.085 | 0.03579 |
| 20 | 44700166 | rs6074012 | C | T | 0.012 | 0.002 | 2.39999e-08 | 0.0001 | 31.102 | -0.018 | 0.013 | 0.166 | 0.53380 |
| 7 | 36189798 | rs61003864 | C | T | 0.016 | 0.003 | 5.60003e-09 | 0.0001 | 33.965 | -0.009 | 0.016 | 0.584 | 0.18290 |
| 14 | 73439258 | rs61988556 | C | T | -0.022 | 0.004 | 1.70000e-09 | 0.0001 | 36.288 | -0.039 | 0.025 | 0.115 | 0.07157 |
| 16 | 53811788 | rs62033400 | G | A | -0.014 | 0.002 | 1.39991e-11 | 0.0001 | 45.620 | 0.007 | 0.013 | 0.624 | 0.41550 |
| 9 | 22081850 | rs6475606 | T | C | -0.020 | 0.002 | 2.29985e-22 | 0.0002 | 94.624 | 0.018 | 0.013 | 0.191 | 0.48510 |
| 15 | 75125645 | rs6495122 | C | A | 0.014 | 0.002 | 1.20005e-11 | 0.0001 | 46.053 | -0.013 | 0.013 | 0.330 | 0.52980 |
| 2 | 44073881 | rs6544713 | C | T | -0.054 | 0.002 | 5.00035e-129 | 0.0013 | 584.006 | 0.030 | 0.016 | 0.063 | 0.69090 |
| 9 | 78730766 | rs6560499 | A | G | -0.012 | 0.002 | 8.90000e-09 | 0.0001 | 33.073 | 0.010 | 0.014 | 0.483 | 0.55070 |
| 13 | 114546549 | rs6602912 | G | T | 0.022 | 0.002 | 7.19946e-22 | 0.0002 | 92.356 | -0.020 | 0.015 | 0.190 | 0.32500 |
| 1 | 109798914 | rs6680227 | A | G | -0.075 | 0.006 | 4.30031e-40 | 0.0004 | 175.647 | 0.020 | 0.031 | 0.523 | 0.03479 |
| 2 | 44080324 | rs6709904 | G | A | -0.043 | 0.003 | 1.10002e-39 | 0.0004 | 173.709 | 0.029 | 0.022 | 0.181 | 0.11230 |
| 2 | 21094034 | rs6732741 | A | T | -0.028 | 0.003 | 2.90001e-18 | 0.0002 | 75.948 | 0.026 | 0.021 | 0.202 | 0.14020 |
| 5 | 156391628 | rs6874202 | C | T | 0.032 | 0.002 | 1.59993e-50 | 0.0005 | 223.438 | -0.002 | 0.014 | 0.905 | 0.64710 |
| 11 | 5677158 | rs7108486 | C | T | -0.039 | 0.007 | 2.39999e-08 | 0.0001 | 31.117 | -0.044 | 0.046 | 0.330 | 0.02187 |
| 3 | 58420613 | rs71311871 | G | A | -0.028 | 0.004 | 7.50067e-14 | 0.0001 | 55.945 | 0.017 | 0.025 | 0.496 | 0.08350 |
| 16 | 72217113 | rs7202323 | G | T | -0.026 | 0.002 | 6.49980e-25 | 0.0002 | 106.257 | 0.018 | 0.017 | 0.275 | 0.23060 |
| 18 | 47160953 | rs7241918 | T | G | 0.016 | 0.003 | 1.00000e-08 | 0.0001 | 32.804 | -0.010 | 0.018 | 0.578 | 0.83700 |
| 17 | 67191270 | rs72631343 | G | C | -0.029 | 0.003 | 4.60045e-21 | 0.0002 | 88.676 | -0.009 | 0.019 | 0.637 | 0.12720 |
| 17 | 76391005 | rs72911393 | T | C | -0.018 | 0.003 | 5.30005e-10 | 0.0001 | 38.552 | -0.044 | 0.022 | 0.048 | 0.15310 |
| 2 | 203527979 | rs7569317 | C | T | 0.018 | 0.002 | 8.90020e-18 | 0.0002 | 73.738 | -0.003 | 0.013 | 0.821 | 0.51490 |
| 10 | 116033892 | rs76468627 | T | C | -0.022 | 0.004 | 3.69999e-08 | 0.0001 | 30.288 | 0.018 | 0.024 | 0.439 | 0.08052 |
| 5 | 122848876 | rs7734476 | A | G | 0.019 | 0.002 | 3.29989e-19 | 0.0002 | 80.257 | 0.007 | 0.013 | 0.620 | 0.58750 |
| 6 | 16126934 | rs7746081 | A | G | -0.023 | 0.002 | 4.00037e-25 | 0.0002 | 107.204 | 0.021 | 0.014 | 0.122 | 0.33600 |
| 6 | 135418916 | rs7776054 | G | A | -0.016 | 0.002 | 8.19974e-12 | 0.0001 | 46.711 | 0.002 | 0.014 | 0.898 | 0.26440 |
| 6 | 26098474 | rs79220007 | C | T | -0.057 | 0.004 | 1.99986e-48 | 0.0005 | 213.795 | 0.008 | 0.035 | 0.817 | 0.04274 |
| 10 | 52352431 | rs79828839 | T | C | 0.014 | 0.003 | 2.90001e-08 | 0.0001 | 30.741 | 0.026 | 0.017 | 0.129 | 0.17990 |
| 19 | 19388500 | rs8107974 | T | A | -0.105 | 0.004 | 2.69774e-158 | 0.0015 | 718.564 | -0.016 | 0.027 | 0.552 | 0.06859 |
| 7 | 1074134 | rs869412 | C | T | -0.014 | 0.003 | 1.29999e-08 | 0.0001 | 32.381 | -0.017 | 0.015 | 0.253 | 0.22070 |
| 1 | 10796866 | rs880315 | C | T | -0.015 | 0.002 | 5.30029e-12 | 0.0001 | 47.583 | 0.015 | 0.013 | 0.263 | 0.35690 |
| 3 | 122268506 | rs9289196 | C | T | 0.017 | 0.003 | 2.99999e-10 | 0.0001 | 39.703 | 0.025 | 0.019 | 0.177 | 0.16300 |
| 2 | 21267461 | rs934197 | A | G | 0.083 | 0.002 | 1.00000e-200 | 0.0031 | 1427.542 | -0.034 | 0.015 | 0.022 | 0.29720 |
| 6 | 42905235 | rs9471968 | G | A | -0.012 | 0.002 | 2.90001e-08 | 0.0001 | 30.782 | -0.004 | 0.013 | 0.742 | 0.55270 |
| 6 | 100602753 | rs9496567 | A | G | -0.017 | 0.002 | 7.80010e-13 | 0.0001 | 51.344 | 0.013 | 0.016 | 0.422 | 0.23760 |
| 22 | 41393520 | rs960596 | T | C | 0.014 | 0.002 | 1.09999e-09 | 0.0001 | 37.098 | -0.033 | 0.014 | 0.016 | 0.35090 |
| 11 | 116648917 | rs964184 | C | G | -0.058 | 0.003 | 8.19974e-79 | 0.0008 | 353.285 | -0.022 | 0.019 | 0.248 | 0.83800 |
| 3 | 142649110 | rs9832727 | G | C | -0.015 | 0.002 | 2.09991e-11 | 0.0001 | 44.856 | -0.017 | 0.014 | 0.217 | 0.38570 |
| 3 | 32535382 | rs9834932 | G | A | -0.032 | 0.004 | 1.50003e-18 | 0.0002 | 77.312 | 0.049 | 0.024 | 0.039 | 0.11230 |
| 4 | 69373407 | rs9884390 | C | T | 0.025 | 0.002 | 5.90065e-24 | 0.0002 | 101.876 | 0.000 | 0.016 | 0.984 | 0.17790 |
| 17 | 7571080 | rs9894946 | G | A | -0.017 | 0.003 | 2.30001e-09 | 0.0001 | 35.692 | -0.029 | 0.019 | 0.122 | 0.82900 |
| 4 | 156507678 | rs990619 | G | C | -0.012 | 0.002 | 1.50000e-08 | 0.0001 | 32.030 | 0.010 | 0.013 | 0.468 | 0.53380 |
| 16 | 71639053 | rs9929977 | A | T | 0.017 | 0.002 | 1.00000e-14 | 0.0001 | 59.897 | -0.025 | 0.014 | 0.065 | 0.33800 |
| 8 | 9183358 | rs9987289 | G | A | 0.045 | 0.004 | 5.10035e-36 | 0.0003 | 157.019 | 0.021 | 0.019 | 0.270 | 0.92540 |

**Supplementary table 2.16. Detailed information of instrumental SNPs for Apolipoprotein A-I in the analysis of PPD.**

| **Chr** | **Position** | **SNP** | **EA** | **OA** | **beta.exposure** | **se.exposure** | **pval.exposure** | **R2** | **F_val** | **beta.outcome** | **se.outcome** | **pval.outcome** | **Eaf.outcome** |
| --- | --- | --- | --- | --- | --- | --- | --- | --- | --- | --- | --- | --- | --- |
| 4 | 177959511 | rs10023962 | G | T | 0.018 | 0.003 | 3.80014e-12 | 0.0001 | 48.249 | -0.009 | 0.021 | 0.653 | 0.81010 |
| 2 | 238699970 | rs10170658 | T | G | -0.013 | 0.002 | 1.70000e-08 | 0.0001 | 31.755 | 0.013 | 0.015 | 0.375 | 0.24450 |
| 7 | 1081562 | rs10271082 | C | G | -0.019 | 0.002 | 1.69981e-21 | 0.0002 | 90.622 | -0.010 | 0.013 | 0.439 | 0.40660 |
| 15 | 102068658 | rs1037117 | A | G | 0.016 | 0.002 | 9.49948e-13 | 0.0001 | 50.944 | 0.007 | 0.015 | 0.646 | 0.26140 |
| 19 | 8466254 | rs10404380 | A | C | -0.026 | 0.002 | 1.59993e-37 | 0.0003 | 163.883 | 0.001 | 0.013 | 0.954 | 0.57750 |
| 2 | 211540507 | rs1047891 | A | C | -0.026 | 0.002 | 3.29989e-34 | 0.0003 | 148.711 | 0.004 | 0.014 | 0.752 | 0.30120 |
| 4 | 87976387 | rs10489044 | G | A | -0.018 | 0.002 | 1.00000e-13 | 0.0001 | 55.355 | 0.008 | 0.019 | 0.654 | 0.18590 |
| 8 | 71338185 | rs10504477 | C | T | -0.012 | 0.002 | 1.20000e-09 | 0.0001 | 36.959 | -0.007 | 0.013 | 0.607 | 0.44040 |
| 4 | 39700173 | rs1055582 | T | C | 0.016 | 0.002 | 2.39994e-15 | 0.0001 | 62.672 | -0.017 | 0.013 | 0.207 | 0.50100 |
| 12 | 103483094 | rs10745954 | G | A | -0.014 | 0.002 | 2.49977e-13 | 0.0001 | 53.563 | 0.017 | 0.014 | 0.220 | 0.51990 |
| 12 | 71372390 | rs10748165 | T | C | 0.011 | 0.002 | 2.10000e-08 | 0.0001 | 31.400 | 0.000 | 0.013 | 0.999 | 0.48410 |
| 11 | 65473798 | rs10750766 | A | C | -0.015 | 0.002 | 7.80010e-12 | 0.0001 | 46.815 | 0.030 | 0.015 | 0.049 | 0.70970 |
| 1 | 183077896 | rs10752898 | C | T | 0.013 | 0.002 | 1.89998e-10 | 0.0001 | 40.609 | -0.014 | 0.013 | 0.286 | 0.54870 |
| 12 | 6731818 | rs10774439 | A | G | 0.016 | 0.003 | 1.29999e-10 | 0.0001 | 41.370 | 0.025 | 0.016 | 0.111 | 0.82900 |
| 1 | 178513895 | rs10798615 | G | T | -0.015 | 0.002 | 6.20012e-15 | 0.0001 | 60.842 | -0.005 | 0.013 | 0.692 | 0.46320 |
| 3 | 154088411 | rs1086056 | G | T | -0.019 | 0.003 | 3.69999e-12 | 0.0001 | 48.299 | 0.000 | 0.017 | 0.986 | 0.86780 |
| 12 | 53770941 | rs10876447 | A | G | -0.019 | 0.003 | 9.30037e-13 | 0.0001 | 50.981 | 0.015 | 0.020 | 0.444 | 0.15010 |
| 10 | 101924418 | rs10883451 | C | T | 0.022 | 0.002 | 1.80011e-29 | 0.0002 | 127.019 | -0.018 | 0.014 | 0.177 | 0.48010 |
| 1 | 23788994 | rs10917383 | G | A | 0.020 | 0.003 | 5.70033e-12 | 0.0001 | 47.430 | -0.012 | 0.021 | 0.554 | 0.87970 |
| 11 | 95320808 | rs11021232 | C | T | -0.016 | 0.003 | 1.29999e-10 | 0.0001 | 41.311 | 0.008 | 0.018 | 0.660 | 0.19780 |
| 12 | 20470221 | rs11045172 | C | A | 0.022 | 0.002 | 2.70023e-19 | 0.0002 | 80.658 | -0.015 | 0.016 | 0.359 | 0.19280 |
| 12 | 124383813 | rs11057390 | G | T | 0.019 | 0.002 | 1.20005e-18 | 0.0002 | 77.637 | 0.049 | 0.015 | 0.001 | 0.33000 |
| 12 | 112072424 | rs11065987 | G | A | -0.014 | 0.002 | 1.50003e-12 | 0.0001 | 50.114 | -0.019 | 0.014 | 0.172 | 0.41950 |
| 12 | 109993603 | rs11067231 | A | C | 0.022 | 0.002 | 3.59998e-29 | 0.0002 | 125.693 | 0.013 | 0.013 | 0.327 | 0.54470 |
| 22 | 21922456 | rs11089620 | G | C | -0.032 | 0.003 | 1.69981e-37 | 0.0003 | 163.792 | -0.018 | 0.015 | 0.228 | 0.18190 |
| 14 | 52581893 | rs111849006 | A | G | 0.015 | 0.003 | 1.70000e-08 | 0.0001 | 31.865 | -0.024 | 0.018 | 0.182 | 0.15110 |
| 11 | 118462554 | rs11216920 | G | A | 0.025 | 0.004 | 1.59993e-12 | 0.0001 | 49.972 | -0.005 | 0.024 | 0.830 | 0.07952 |
| 11 | 103870755 | rs11226108 | C | G | -0.015 | 0.002 | 4.20001e-09 | 0.0001 | 34.536 | -0.007 | 0.017 | 0.687 | 0.19580 |
| 9 | 107721423 | rs1125873 | T | A | 0.012 | 0.002 | 5.10000e-10 | 0.0001 | 38.633 | -0.007 | 0.013 | 0.621 | 0.50890 |
| 8 | 19979846 | rs112928559 | T | C | -0.037 | 0.005 | 2.60016e-12 | 0.0001 | 48.982 | -0.087 | 0.057 | 0.127 | 0.03678 |
| 20 | 17596155 | rs1132274 | A | C | -0.029 | 0.003 | 3.19963e-26 | 0.0002 | 112.215 | 0.006 | 0.019 | 0.761 | 0.13420 |
| 5 | 53405314 | rs116006942 | A | G | -0.028 | 0.004 | 9.39940e-12 | 0.0001 | 46.457 | -0.001 | 0.034 | 0.981 | 0.05567 |
| 15 | 58724706 | rs11632618 | A | G | 0.108 | 0.004 | 1.09901e-173 | 0.0015 | 789.319 | 0.013 | 0.031 | 0.660 | 0.07356 |
| 16 | 88039447 | rs11641548 | C | A | -0.013 | 0.002 | 3.59998e-11 | 0.0001 | 43.840 | 0.003 | 0.015 | 0.823 | 0.37280 |
| 1 | 63139730 | rs1168124 | T | C | 0.043 | 0.002 | 1.20005e-95 | 0.0008 | 430.572 | -0.021 | 0.015 | 0.157 | 0.69480 |
| 2 | 100796182 | rs11691486 | C | T | 0.014 | 0.002 | 8.30004e-10 | 0.0001 | 37.696 | -0.015 | 0.015 | 0.336 | 0.27040 |
| 15 | 59264999 | rs117399007 | T | C | -0.029 | 0.005 | 2.50000e-09 | 0.0001 | 35.503 | 0.027 | 0.032 | 0.396 | 0.03181 |
| 11 | 408174 | rs117739035 | T | G | -0.031 | 0.005 | 6.59994e-09 | 0.0001 | 33.642 | -0.023 | 0.029 | 0.436 | 0.03579 |
| 8 | 20056640 | rs117853493 | A | G | 0.057 | 0.008 | 1.69981e-12 | 0.0001 | 49.783 | 0.032 | 0.042 | 0.451 | 0.01889 |
| 17 | 481604 | rs11870735 | T | C | -0.015 | 0.003 | 2.19999e-09 | 0.0001 | 35.758 | 0.015 | 0.020 | 0.462 | 0.19380 |
| 1 | 219679967 | rs12044156 | C | G | -0.012 | 0.002 | 1.50000e-09 | 0.0001 | 36.525 | -0.007 | 0.013 | 0.591 | 0.58450 |
| 12 | 125339990 | rs12229372 | C | T | 0.030 | 0.003 | 2.39994e-19 | 0.0002 | 80.872 | 0.007 | 0.018 | 0.695 | 0.09940 |
| 11 | 27744859 | rs12273363 | C | T | -0.020 | 0.002 | 3.90032e-16 | 0.0001 | 66.284 | -0.015 | 0.016 | 0.325 | 0.17990 |
| 14 | 24634825 | rs12436555 | A | G | -0.015 | 0.003 | 1.79999e-08 | 0.0001 | 31.686 | 0.005 | 0.021 | 0.795 | 0.13120 |
| 19 | 52343496 | rs12462109 | T | C | -0.013 | 0.002 | 6.40000e-10 | 0.0001 | 38.196 | 0.033 | 0.014 | 0.022 | 0.29030 |
| 2 | 27730940 | rs1260326 | C | T | -0.026 | 0.002 | 4.49987e-38 | 0.0003 | 166.396 | 0.004 | 0.014 | 0.762 | 0.58950 |
| 1 | 109817590 | rs12740374 | T | G | 0.044 | 0.002 | 1.10002e-78 | 0.0007 | 352.708 | -0.017 | 0.016 | 0.307 | 0.21270 |
| 15 | 58639710 | rs12898210 | G | T | -0.049 | 0.003 | 6.40030e-60 | 0.0005 | 266.539 | -0.015 | 0.022 | 0.482 | 0.13520 |
| 17 | 4083923 | rs12943517 | A | G | -0.012 | 0.002 | 2.39999e-08 | 0.0001 | 31.120 | 0.014 | 0.015 | 0.345 | 0.30020 |
| 19 | 45442528 | rs12977604 | G | C | 0.037 | 0.002 | 1.99986e-71 | 0.0007 | 319.346 | 0.012 | 0.013 | 0.368 | 0.49200 |
| 2 | 188323311 | rs12987470 | T | A | 0.012 | 0.002 | 2.19999e-08 | 0.0001 | 31.308 | -0.017 | 0.014 | 0.242 | 0.29720 |
| 2 | 111710863 | rs13024140 | C | G | -0.012 | 0.002 | 6.29999e-09 | 0.0001 | 33.744 | 0.020 | 0.013 | 0.127 | 0.42150 |
| 3 | 152062241 | rs13059175 | T | C | 0.015 | 0.002 | 3.59998e-12 | 0.0001 | 48.324 | 0.014 | 0.017 | 0.396 | 0.28830 |
| 4 | 103188709 | rs13107325 | T | C | -0.072 | 0.004 | 1.69981e-82 | 0.0007 | 370.189 | 0.122 | 0.058 | 0.035 | 0.07952 |
| 4 | 3443931 | rs13108218 | G | A | -0.013 | 0.002 | 5.30029e-11 | 0.0001 | 43.055 | -0.029 | 0.014 | 0.041 | 0.61030 |
| 15 | 58586129 | rs1318175 | T | C | -0.078 | 0.003 | 3.39625e-186 | 0.0016 | 846.918 | 0.021 | 0.017 | 0.230 | 0.18490 |
| 22 | 38572526 | rs133015 | G | C | 0.020 | 0.002 | 5.60015e-24 | 0.0002 | 101.971 | -0.017 | 0.013 | 0.217 | 0.43440 |
| 3 | 52532118 | rs13326165 | G | A | -0.020 | 0.002 | 8.00018e-16 | 0.0001 | 64.864 | 0.025 | 0.017 | 0.155 | 0.80820 |
| 14 | 74250126 | rs13379043 | C | T | 0.022 | 0.002 | 1.50003e-22 | 0.0002 | 95.467 | 0.009 | 0.017 | 0.584 | 0.29820 |
| 4 | 24626903 | rs1395221 | T | G | -0.013 | 0.002 | 3.90032e-11 | 0.0001 | 43.658 | -0.002 | 0.013 | 0.894 | 0.40850 |
| 3 | 185883562 | rs1400362 | C | T | 0.013 | 0.002 | 3.29997e-08 | 0.0001 | 30.504 | -0.019 | 0.016 | 0.247 | 0.71470 |
| 11 | 117221862 | rs141368429 | T | C | -0.057 | 0.005 | 1.90020e-35 | 0.0003 | 154.416 | -0.010 | 0.043 | 0.810 | 0.04672 |
| 20 | 571467 | rs144033177 | C | A | -0.049 | 0.008 | 8.90000e-10 | 0.0001 | 37.555 | -0.022 | 0.053 | 0.673 | 0.01590 |
| 2 | 136407479 | rs1446585 | G | A | 0.019 | 0.002 | 9.20026e-17 | 0.0001 | 69.132 | -0.049 | 0.014 | 0.001 | 0.46120 |
| 17 | 41809207 | rs145947882 | C | A | -0.136 | 0.006 | 6.90240e-105 | 0.0009 | 473.072 | 0.004 | 0.039 | 0.927 | 0.03579 |
| 9 | 107732530 | rs147772065 | C | G | 0.031 | 0.005 | 5.99998e-09 | 0.0001 | 33.832 | -0.024 | 0.043 | 0.587 | 0.03579 |
| 4 | 110766064 | rs148827772 | G | A | -0.057 | 0.007 | 3.69999e-16 | 0.0001 | 66.372 | 0.117 | 0.044 | 0.008 | 0.02386 |
| 18 | 46781014 | rs150237291 | C | T | 0.049 | 0.007 | 2.29985e-13 | 0.0001 | 53.750 | 0.038 | 0.039 | 0.324 | 0.01690 |
| 7 | 106957161 | rs150483923 | A | C | 0.020 | 0.002 | 5.30029e-17 | 0.0001 | 70.206 | 0.009 | 0.018 | 0.589 | 0.20080 |
| 5 | 111246489 | rs1540687 | T | A | 0.012 | 0.002 | 2.80001e-08 | 0.0001 | 30.863 | 0.020 | 0.014 | 0.146 | 0.65110 |
| 15 | 58671559 | rs1601933 | T | C | -0.084 | 0.002 | 1.00000e-200 | 0.0035 | 1781.378 | 0.019 | 0.013 | 0.150 | 0.45530 |
| 11 | 2942593 | rs1661052 | A | G | 0.029 | 0.003 | 6.20012e-18 | 0.0001 | 74.462 | 0.005 | 0.026 | 0.855 | 0.91150 |
| 3 | 71756272 | rs17008972 | A | G | 0.019 | 0.003 | 1.00000e-10 | 0.0001 | 41.774 | 0.012 | 0.017 | 0.494 | 0.13420 |
| 7 | 17920253 | rs17138358 | C | G | -0.026 | 0.002 | 6.59933e-39 | 0.0003 | 170.215 | -0.022 | 0.014 | 0.128 | 0.40160 |
| 2 | 48962291 | rs17326656 | T | G | -0.018 | 0.002 | 3.10027e-15 | 0.0001 | 62.233 | -0.042 | 0.018 | 0.020 | 0.21970 |
| 11 | 61592362 | rs174566 | G | A | -0.035 | 0.002 | 1.00000e-64 | 0.0006 | 288.606 | 0.011 | 0.013 | 0.412 | 0.35590 |
| 7 | 94974185 | rs17883513 | G | A | 0.030 | 0.005 | 2.69998e-08 | 0.0001 | 30.890 | -0.063 | 0.036 | 0.078 | 0.02485 |
| 4 | 100256984 | rs1789896 | A | G | 0.015 | 0.002 | 2.60016e-14 | 0.0001 | 58.008 | 0.007 | 0.013 | 0.604 | 0.54470 |
| 20 | 43042364 | rs1800961 | T | C | -0.149 | 0.006 | 1.30017e-152 | 0.0013 | 692.422 | 0.000 | 0.032 | 0.997 | 0.03678 |
| 3 | 70936712 | rs1852922 | A | G | 0.012 | 0.002 | 6.40000e-09 | 0.0001 | 33.717 | -0.020 | 0.015 | 0.164 | 0.68290 |
| 5 | 108656635 | rs1862205 | A | G | 0.011 | 0.002 | 3.20000e-08 | 0.0001 | 30.603 | 0.003 | 0.013 | 0.832 | 0.40460 |
| 5 | 131121165 | rs188502504 | C | T | -0.029 | 0.005 | 2.99999e-08 | 0.0001 | 30.690 | -0.052 | 0.026 | 0.046 | 0.03479 |
| 11 | 14029854 | rs1919309 | C | T | 0.012 | 0.002 | 2.80001e-09 | 0.0001 | 35.309 | -0.006 | 0.014 | 0.683 | 0.49900 |
| 10 | 126696496 | rs1970811 | C | T | -0.012 | 0.002 | 4.00000e-09 | 0.0001 | 34.631 | 0.019 | 0.014 | 0.156 | 0.45920 |
| 1 | 55491851 | rs199717562 | G | A | 0.015 | 0.003 | 6.40000e-09 | 0.0001 | 33.694 | 0.022 | 0.015 | 0.156 | 0.19580 |
| 9 | 107586753 | rs2066714 | C | T | 0.054 | 0.003 | 7.89951e-77 | 0.0007 | 344.156 | 0.013 | 0.023 | 0.552 | 0.13520 |
| 10 | 94839642 | rs2068888 | A | G | 0.013 | 0.002 | 9.79941e-12 | 0.0001 | 46.364 | -0.014 | 0.013 | 0.299 | 0.48610 |
| 17 | 26695832 | rs2071379 | G | A | -0.021 | 0.002 | 8.10028e-27 | 0.0002 | 114.934 | 0.010 | 0.013 | 0.441 | 0.57850 |
| 14 | 75261641 | rs2111705 | A | G | -0.015 | 0.002 | 3.69999e-14 | 0.0001 | 57.319 | 0.013 | 0.013 | 0.344 | 0.54170 |
| 4 | 55521017 | rs2159935 | A | G | 0.011 | 0.002 | 5.60003e-09 | 0.0001 | 33.984 | 0.010 | 0.013 | 0.465 | 0.46920 |
| 10 | 122857596 | rs2245095 | T | C | -0.026 | 0.003 | 1.10002e-13 | 0.0001 | 55.096 | 0.005 | 0.019 | 0.806 | 0.08549 |
| 8 | 103876780 | rs2247355 | T | C | 0.018 | 0.003 | 2.99985e-12 | 0.0001 | 48.717 | 0.031 | 0.015 | 0.043 | 0.19780 |
| 20 | 46279531 | rs2256720 | C | T | 0.012 | 0.002 | 7.39997e-10 | 0.0001 | 37.907 | -0.017 | 0.014 | 0.236 | 0.46520 |
| 11 | 47360412 | rs2269434 | C | T | 0.035 | 0.002 | 1.69981e-63 | 0.0005 | 282.921 | -0.022 | 0.013 | 0.101 | 0.34690 |
| 1 | 230297659 | rs2281719 | T | C | 0.053 | 0.002 | 1.49968e-155 | 0.0014 | 705.910 | 0.008 | 0.013 | 0.544 | 0.60830 |
| 22 | 44340904 | rs2294915 | T | C | -0.020 | 0.002 | 1.00000e-17 | 0.0001 | 73.504 | -0.001 | 0.015 | 0.948 | 0.25250 |
| 9 | 107576246 | rs2297409 | A | G | -0.037 | 0.002 | 1.50003e-49 | 0.0004 | 218.990 | 0.009 | 0.020 | 0.654 | 0.15810 |
| 1 | 935222 | rs2298214 | A | C | -0.013 | 0.002 | 4.70002e-11 | 0.0001 | 43.298 | -0.008 | 0.013 | 0.542 | 0.58950 |
| 18 | 47429022 | rs2298624 | T | C | 0.032 | 0.003 | 1.99986e-28 | 0.0002 | 122.325 | 0.030 | 0.018 | 0.085 | 0.13720 |
| 11 | 64021821 | rs2302263 | T | C | -0.029 | 0.003 | 1.50003e-16 | 0.0001 | 68.168 | -0.016 | 0.020 | 0.427 | 0.09940 |
| 16 | 57157206 | rs2305696 | T | C | -0.018 | 0.002 | 3.90032e-21 | 0.0002 | 89.035 | 0.022 | 0.013 | 0.104 | 0.53180 |
| 21 | 46271452 | rs235314 | T | C | -0.021 | 0.002 | 1.50003e-27 | 0.0002 | 118.251 | -0.018 | 0.013 | 0.166 | 0.53280 |
| 2 | 30478453 | rs2362541 | G | T | -0.011 | 0.002 | 9.09997e-09 | 0.0001 | 33.016 | -0.019 | 0.013 | 0.152 | 0.51390 |
| 6 | 32374595 | rs2395158 | G | A | -0.034 | 0.003 | 1.00000e-39 | 0.0003 | 173.931 | -0.006 | 0.019 | 0.733 | 0.11730 |
| 10 | 113978097 | rs2419605 | G | A | -0.031 | 0.003 | 2.19989e-29 | 0.0002 | 126.645 | 0.007 | 0.020 | 0.732 | 0.13920 |
| 20 | 33104592 | rs2424993 | C | G | -0.016 | 0.002 | 4.90004e-14 | 0.0001 | 56.774 | -0.004 | 0.015 | 0.766 | 0.66100 |
| 14 | 105258437 | rs2494747 | T | G | -0.032 | 0.002 | 4.30031e-57 | 0.0005 | 253.601 | 0.009 | 0.014 | 0.527 | 0.61030 |
| 9 | 136919416 | rs2520096 | G | A | 0.014 | 0.002 | 2.30001e-10 | 0.0001 | 40.208 | 0.024 | 0.014 | 0.075 | 0.30420 |
| 2 | 65276736 | rs2540951 | G | A | 0.012 | 0.002 | 2.00000e-09 | 0.0001 | 35.961 | -0.003 | 0.014 | 0.814 | 0.38670 |
| 5 | 72888280 | rs2544654 | T | G | 0.013 | 0.002 | 3.69999e-09 | 0.0001 | 34.759 | -0.004 | 0.016 | 0.783 | 0.73160 |
| 5 | 134444982 | rs254559 | A | C | -0.017 | 0.002 | 1.39991e-17 | 0.0001 | 72.814 | 0.028 | 0.014 | 0.035 | 0.40950 |
| 1 | 220970028 | rs2642438 | G | A | 0.029 | 0.002 | 3.29989e-42 | 0.0004 | 185.360 | -0.016 | 0.015 | 0.267 | 0.72170 |
| 1 | 150940625 | rs267738 | G | T | 0.034 | 0.002 | 7.50067e-48 | 0.0004 | 211.216 | -0.001 | 0.017 | 0.959 | 0.19090 |
| 7 | 36178753 | rs2726112 | G | A | -0.014 | 0.002 | 1.99986e-12 | 0.0001 | 49.465 | -0.001 | 0.013 | 0.922 | 0.43540 |
| 9 | 107661742 | rs2740488 | C | A | -0.080 | 0.002 | 1.00000e-200 | 0.0025 | 1306.880 | 0.018 | 0.017 | 0.294 | 0.24650 |
| 10 | 113934384 | rs2803619 | C | G | -0.037 | 0.002 | 2.09991e-65 | 0.0006 | 291.708 | -0.017 | 0.014 | 0.232 | 0.69090 |
| 10 | 33647091 | rs2804894 | A | G | 0.016 | 0.002 | 4.79954e-13 | 0.0001 | 52.288 | -0.002 | 0.016 | 0.897 | 0.73760 |
| 15 | 74712937 | rs28362901 | A | C | -0.025 | 0.003 | 1.00000e-12 | 0.0001 | 50.792 | 0.006 | 0.021 | 0.764 | 0.08250 |
| 5 | 153360230 | rs286965 | C | T | -0.016 | 0.002 | 3.19963e-15 | 0.0001 | 62.110 | -0.022 | 0.014 | 0.112 | 0.62820 |
| 16 | 81534790 | rs2925979 | C | T | 0.029 | 0.002 | 6.09958e-41 | 0.0003 | 179.548 | 0.007 | 0.014 | 0.607 | 0.71170 |
| 2 | 227099180 | rs2943645 | T | C | -0.034 | 0.002 | 1.80011e-61 | 0.0005 | 273.635 | -0.008 | 0.014 | 0.558 | 0.62430 |
| 5 | 158003020 | rs2963468 | G | A | -0.017 | 0.002 | 8.99912e-14 | 0.0001 | 55.574 | -0.003 | 0.019 | 0.867 | 0.23460 |
| 19 | 45251156 | rs2965169 | C | A | 0.019 | 0.002 | 7.59976e-21 | 0.0002 | 87.692 | 0.000 | 0.014 | 0.989 | 0.43740 |
| 3 | 12316339 | rs2972166 | A | G | 0.015 | 0.002 | 1.10002e-11 | 0.0001 | 46.165 | -0.028 | 0.014 | 0.051 | 0.28930 |
| 1 | 23832103 | rs2999141 | C | A | -0.012 | 0.002 | 4.90004e-08 | 0.0001 | 29.750 | 0.002 | 0.015 | 0.872 | 0.29320 |
| 8 | 19819439 | rs326 | G | A | 0.083 | 0.002 | 1.00000e-200 | 0.0028 | 1483.151 | -0.009 | 0.015 | 0.548 | 0.32700 |
| 16 | 28139077 | rs33042 | A | G | 0.017 | 0.002 | 2.49977e-13 | 0.0001 | 53.597 | -0.013 | 0.015 | 0.399 | 0.24750 |
| 10 | 33967489 | rs34045894 | A | G | -0.015 | 0.003 | 4.79999e-08 | 0.0001 | 29.799 | 0.034 | 0.017 | 0.052 | 0.16600 |
| 17 | 40781561 | rs34138141 | T | G | -0.018 | 0.002 | 1.29987e-16 | 0.0001 | 68.466 | -0.022 | 0.016 | 0.160 | 0.25650 |
| 15 | 75370012 | rs34180494 | C | A | -0.013 | 0.002 | 4.79999e-09 | 0.0001 | 34.277 | 0.007 | 0.014 | 0.595 | 0.31110 |
| 1 | 27092322 | rs34397747 | C | T | -0.039 | 0.004 | 2.19989e-28 | 0.0002 | 122.085 | -0.029 | 0.025 | 0.249 | 0.08151 |
| 3 | 123051019 | rs34642857 | C | T | -0.013 | 0.002 | 4.49997e-09 | 0.0001 | 34.391 | 0.000 | 0.017 | 0.979 | 0.18190 |
| 7 | 50271064 | rs34767118 | G | A | 0.017 | 0.002 | 8.40040e-16 | 0.0001 | 64.776 | 0.015 | 0.014 | 0.297 | 0.36080 |
| 16 | 15139594 | rs34955778 | C | T | -0.012 | 0.002 | 3.69999e-10 | 0.0001 | 39.241 | -0.011 | 0.013 | 0.422 | 0.41950 |
| 2 | 20363666 | rs35135293 | T | C | -0.022 | 0.002 | 2.70023e-28 | 0.0002 | 121.657 | -0.007 | 0.013 | 0.605 | 0.48010 |
| 5 | 176541370 | rs351862 | T | C | 0.018 | 0.003 | 2.59998e-09 | 0.0001 | 35.428 | 0.004 | 0.017 | 0.795 | 0.12030 |
| 7 | 6451080 | rs35745599 | T | C | 0.028 | 0.002 | 4.00037e-35 | 0.0003 | 152.917 | 0.008 | 0.016 | 0.628 | 0.20970 |
| 16 | 72156666 | rs35909200 | G | T | 0.019 | 0.003 | 1.69981e-12 | 0.0001 | 49.804 | 0.009 | 0.018 | 0.602 | 0.16500 |
| 8 | 121887377 | rs36024006 | A | G | -0.018 | 0.002 | 1.59993e-19 | 0.0002 | 81.705 | -0.001 | 0.013 | 0.937 | 0.44430 |
| 8 | 72399623 | rs36096231 | T | C | -0.021 | 0.004 | 4.00000e-08 | 0.0001 | 30.129 | -0.018 | 0.037 | 0.630 | 0.05467 |
| 19 | 54800500 | rs367070 | G | A | 0.039 | 0.002 | 8.40040e-62 | 0.0005 | 275.183 | 0.008 | 0.014 | 0.569 | 0.17990 |
| 3 | 119529113 | rs3732356 | T | G | -0.037 | 0.004 | 2.70023e-20 | 0.0002 | 85.178 | 0.023 | 0.026 | 0.382 | 0.94230 |
| 11 | 47380340 | rs3740688 | T | G | 0.016 | 0.002 | 2.60016e-15 | 0.0001 | 62.579 | 0.003 | 0.013 | 0.822 | 0.54270 |
| 1 | 205677148 | rs3747973 | G | A | 0.015 | 0.002 | 9.89920e-14 | 0.0001 | 55.390 | -0.025 | 0.014 | 0.068 | 0.58950 |
| 5 | 127350549 | rs3749748 | T | C | 0.022 | 0.002 | 4.30031e-22 | 0.0002 | 93.402 | 0.013 | 0.018 | 0.462 | 0.21070 |
| 17 | 1994071 | rs3760230 | G | C | -0.014 | 0.002 | 7.29962e-12 | 0.0001 | 46.947 | 0.040 | 0.014 | 0.003 | 0.59840 |
| 1 | 40035928 | rs3768321 | T | G | -0.042 | 0.002 | 6.20012e-66 | 0.0006 | 294.150 | 0.033 | 0.018 | 0.066 | 0.18790 |
| 6 | 116317092 | rs3798233 | C | A | 0.018 | 0.002 | 2.29985e-19 | 0.0002 | 80.924 | -0.011 | 0.013 | 0.398 | 0.42350 |
| 10 | 45952745 | rs3802548 | A | T | 0.034 | 0.002 | 9.39940e-50 | 0.0004 | 219.923 | 0.024 | 0.015 | 0.106 | 0.27040 |
| 17 | 16851834 | rs3818716 | T | C | -0.013 | 0.002 | 5.19996e-10 | 0.0001 | 38.606 | -0.022 | 0.014 | 0.115 | 0.34590 |
| 7 | 135194613 | rs3828960 | G | A | -0.015 | 0.002 | 1.70000e-09 | 0.0001 | 36.248 | -0.036 | 0.016 | 0.025 | 0.20680 |
| 10 | 80941936 | rs3915932 | C | G | 0.016 | 0.002 | 2.19989e-15 | 0.0001 | 62.905 | 0.031 | 0.014 | 0.025 | 0.41150 |
| 6 | 161008646 | rs41272086 | A | G | -0.037 | 0.003 | 1.39991e-31 | 0.0003 | 136.682 | 0.003 | 0.022 | 0.884 | 0.08549 |
| 19 | 45411941 | rs429358 | C | T | -0.103 | 0.003 | 1.00000e-200 | 0.0027 | 1426.265 | 0.032 | 0.017 | 0.069 | 0.15510 |
| 16 | 57235644 | rs4330777 | A | G | -0.017 | 0.002 | 2.80027e-17 | 0.0001 | 71.446 | 0.019 | 0.013 | 0.162 | 0.49400 |
| 3 | 36892717 | rs4441609 | C | T | 0.011 | 0.002 | 4.39997e-08 | 0.0001 | 29.959 | -0.008 | 0.013 | 0.568 | 0.63020 |
| 18 | 19641009 | rs4632228 | T | G | -0.020 | 0.002 | 1.59993e-16 | 0.0001 | 68.046 | -0.009 | 0.016 | 0.551 | 0.22370 |
| 1 | 42407229 | rs4660586 | T | C | 0.013 | 0.002 | 1.79999e-08 | 0.0001 | 31.684 | 0.008 | 0.014 | 0.597 | 0.72760 |
| 3 | 39214256 | rs4676609 | T | C | 0.015 | 0.002 | 1.60000e-09 | 0.0001 | 36.456 | -0.004 | 0.016 | 0.806 | 0.17690 |
| 1 | 20917108 | rs472629 | A | G | -0.018 | 0.002 | 1.29987e-18 | 0.0001 | 77.502 | -0.006 | 0.016 | 0.695 | 0.30120 |
| 12 | 20597577 | rs4762756 | C | T | 0.014 | 0.002 | 4.60002e-10 | 0.0001 | 38.826 | -0.031 | 0.017 | 0.069 | 0.71170 |
| 16 | 56711375 | rs4784709 | A | T | -0.062 | 0.005 | 7.39946e-36 | 0.0003 | 156.279 | 0.036 | 0.049 | 0.462 | 0.96320 |
| 17 | 37746847 | rs4795386 | G | A | 0.026 | 0.002 | 5.10035e-31 | 0.0003 | 134.143 | -0.027 | 0.015 | 0.077 | 0.71570 |
| 19 | 3408665 | rs4807462 | T | A | -0.011 | 0.002 | 2.50000e-08 | 0.0001 | 31.042 | -0.020 | 0.014 | 0.147 | 0.35590 |
| 22 | 39125074 | rs4820346 | G | C | 0.013 | 0.002 | 1.09999e-09 | 0.0001 | 37.070 | -0.008 | 0.014 | 0.551 | 0.69090 |
| 8 | 144496772 | rs4875043 | C | A | -0.015 | 0.002 | 2.90001e-10 | 0.0001 | 39.750 | -0.003 | 0.016 | 0.868 | 0.23360 |
| 11 | 66066993 | rs4930352 | T | G | 0.017 | 0.002 | 5.30029e-17 | 0.0001 | 70.221 | -0.017 | 0.013 | 0.191 | 0.48910 |
| 8 | 9151688 | rs49675 | A | G | 0.021 | 0.003 | 1.29999e-10 | 0.0001 | 41.258 | -0.017 | 0.027 | 0.519 | 0.08350 |
| 7 | 100698648 | rs55710224 | A | G | 0.012 | 0.002 | 6.10000e-10 | 0.0001 | 38.292 | -0.013 | 0.013 | 0.341 | 0.48310 |
| 1 | 234853268 | rs557933 | C | A | 0.019 | 0.002 | 3.19963e-22 | 0.0002 | 93.991 | 0.006 | 0.013 | 0.667 | 0.51690 |
| 5 | 158622532 | rs55801554 | A | C | 0.014 | 0.002 | 1.70000e-09 | 0.0001 | 36.297 | -0.028 | 0.015 | 0.070 | 0.25550 |
| 7 | 44790807 | rs55812947 | C | T | -0.016 | 0.003 | 4.00000e-08 | 0.0001 | 30.162 | -0.024 | 0.026 | 0.343 | 0.10930 |
| 11 | 75451281 | rs559355 | T | A | -0.045 | 0.003 | 8.80035e-62 | 0.0005 | 275.091 | 0.003 | 0.016 | 0.866 | 0.17300 |
| 1 | 66110292 | rs57274629 | G | A | 0.015 | 0.002 | 3.29989e-13 | 0.0001 | 52.995 | 0.004 | 0.013 | 0.755 | 0.36780 |
| 2 | 242237902 | rs59104589 | T | C | 0.017 | 0.002 | 7.59976e-17 | 0.0001 | 69.500 | 0.026 | 0.014 | 0.057 | 0.33900 |
| 8 | 19750044 | rs59347135 | G | C | -0.081 | 0.005 | 1.39991e-63 | 0.0006 | 283.353 | -0.064 | 0.034 | 0.065 | 0.04076 |
| 20 | 30061695 | rs6057911 | G | C | 0.019 | 0.003 | 2.00000e-10 | 0.0001 | 40.473 | -0.012 | 0.017 | 0.493 | 0.13220 |
| 20 | 62372148 | rs6062510 | C | G | -0.015 | 0.002 | 3.50026e-13 | 0.0001 | 52.901 | 0.011 | 0.015 | 0.469 | 0.71970 |
| 12 | 57839173 | rs61352607 | T | G | 0.028 | 0.002 | 1.10002e-33 | 0.0003 | 146.314 | 0.006 | 0.016 | 0.703 | 0.19480 |
| 11 | 116710968 | rs613808 | G | A | -0.085 | 0.002 | 1.00000e-200 | 0.0029 | 1514.941 | -0.017 | 0.014 | 0.219 | 0.69880 |
| 8 | 95997165 | rs61596977 | T | C | -0.016 | 0.003 | 3.79997e-08 | 0.0001 | 30.227 | 0.017 | 0.018 | 0.350 | 0.12720 |
| 1 | 182154990 | rs61805076 | C | T | -0.015 | 0.002 | 1.50003e-13 | 0.0001 | 54.605 | 0.000 | 0.015 | 0.999 | 0.32210 |
| 14 | 81676844 | rs61980899 | C | T | -0.014 | 0.002 | 1.50003e-12 | 0.0001 | 50.016 | 0.010 | 0.013 | 0.435 | 0.54470 |
| 2 | 242793 | rs62114506 | C | G | 0.014 | 0.002 | 2.69998e-10 | 0.0001 | 39.867 | 0.009 | 0.014 | 0.536 | 0.27630 |
| 2 | 48755477 | rs62135193 | T | C | -0.011 | 0.002 | 4.20001e-08 | 0.0001 | 30.055 | -0.004 | 0.013 | 0.748 | 0.54770 |
| 15 | 91423543 | rs6224 | T | G | 0.013 | 0.002 | 1.79999e-10 | 0.0001 | 40.675 | -0.017 | 0.013 | 0.195 | 0.47120 |
| 5 | 43815578 | rs62369502 | C | T | -0.021 | 0.004 | 6.80002e-09 | 0.0001 | 33.579 | -0.060 | 0.028 | 0.033 | 0.07853 |
| 5 | 180225919 | rs62405458 | T | C | -0.015 | 0.003 | 6.49995e-09 | 0.0001 | 33.669 | 0.012 | 0.016 | 0.442 | 0.22470 |
| 4 | 26066863 | rs6448429 | T | C | -0.022 | 0.003 | 6.40030e-17 | 0.0001 | 69.857 | 0.006 | 0.019 | 0.744 | 0.18290 |
| 8 | 116601894 | rs6469605 | T | C | 0.035 | 0.002 | 1.00000e-69 | 0.0006 | 311.485 | 0.014 | 0.015 | 0.333 | 0.58150 |
| 17 | 28661769 | rs6505176 | G | T | -0.017 | 0.002 | 8.60003e-17 | 0.0001 | 69.273 | -0.022 | 0.014 | 0.127 | 0.34190 |
| 6 | 153433701 | rs6557267 | T | C | -0.012 | 0.002 | 2.80001e-09 | 0.0001 | 35.344 | -0.002 | 0.013 | 0.888 | 0.40060 |
| 2 | 66709468 | rs6705285 | T | G | 0.011 | 0.002 | 1.70000e-08 | 0.0001 | 31.860 | -0.002 | 0.014 | 0.914 | 0.63020 |
| 2 | 21231524 | rs676210 | A | G | 0.060 | 0.002 | 8.70964e-136 | 0.0012 | 615.101 | 0.028 | 0.015 | 0.058 | 0.21770 |
| 3 | 50041313 | rs6765484 | T | C | 0.020 | 0.002 | 2.49977e-25 | 0.0002 | 108.173 | -0.044 | 0.013 | 0.001 | 0.48710 |
| 3 | 15702641 | rs6772763 | C | T | 0.013 | 0.002 | 8.49963e-11 | 0.0001 | 42.144 | -0.022 | 0.014 | 0.097 | 0.58350 |
| 3 | 141114293 | rs6807935 | G | A | -0.013 | 0.002 | 8.19993e-10 | 0.0001 | 37.708 | 0.000 | 0.014 | 0.978 | 0.31610 |
| 18 | 2981398 | rs681869 | T | C | -0.017 | 0.002 | 1.50003e-14 | 0.0001 | 59.114 | -0.018 | 0.015 | 0.235 | 0.70280 |
| 9 | 15304782 | rs686030 | A | C | 0.056 | 0.003 | 4.70002e-89 | 0.0008 | 400.307 | -0.019 | 0.021 | 0.361 | 0.86980 |
| 7 | 150542711 | rs6977416 | A | G | -0.016 | 0.002 | 5.70033e-14 | 0.0001 | 56.457 | 0.000 | 0.014 | 0.976 | 0.30120 |
| 1 | 93836218 | rs698927 | C | A | 0.025 | 0.003 | 2.80027e-22 | 0.0002 | 94.252 | 0.003 | 0.017 | 0.847 | 0.16900 |
| 9 | 117134945 | rs7032795 | C | T | 0.015 | 0.002 | 1.29987e-13 | 0.0001 | 54.915 | 0.008 | 0.013 | 0.538 | 0.48410 |
| 12 | 125326153 | rs7136506 | C | T | -0.032 | 0.002 | 2.09991e-39 | 0.0003 | 172.511 | -0.016 | 0.016 | 0.314 | 0.21970 |
| 14 | 100794818 | rs7147511 | T | C | -0.015 | 0.002 | 1.50003e-13 | 0.0001 | 54.512 | -0.005 | 0.013 | 0.738 | 0.47320 |
| 15 | 41888918 | rs7170463 | G | A | 0.016 | 0.002 | 2.19989e-14 | 0.0001 | 58.386 | -0.033 | 0.014 | 0.016 | 0.33000 |
| 17 | 66827491 | rs7216643 | T | C | -0.022 | 0.002 | 2.90001e-21 | 0.0002 | 89.577 | -0.019 | 0.016 | 0.223 | 0.21970 |
| 18 | 57735552 | rs7238484 | T | G | -0.021 | 0.002 | 5.30029e-22 | 0.0002 | 92.966 | -0.010 | 0.017 | 0.533 | 0.26840 |
| 19 | 18614935 | rs7251640 | C | T | 0.015 | 0.002 | 1.29999e-09 | 0.0001 | 36.787 | 0.007 | 0.016 | 0.668 | 0.20280 |
| 4 | 154208278 | rs72729623 | T | C | -0.015 | 0.003 | 4.39997e-08 | 0.0001 | 29.957 | -0.017 | 0.018 | 0.346 | 0.11730 |
| 1 | 243740445 | rs72761606 | C | T | -0.016 | 0.003 | 2.80001e-08 | 0.0001 | 30.840 | -0.008 | 0.018 | 0.642 | 0.14120 |
| 2 | 203477868 | rs72926946 | A | C | -0.015 | 0.002 | 6.20012e-13 | 0.0001 | 51.776 | 0.033 | 0.015 | 0.024 | 0.30320 |
| 6 | 127454893 | rs72959041 | A | G | -0.036 | 0.005 | 4.19952e-15 | 0.0001 | 61.594 | -0.051 | 0.027 | 0.060 | 0.05070 |
| 12 | 71114400 | rs7304603 | C | T | -0.013 | 0.002 | 1.09999e-10 | 0.0001 | 41.654 | -0.014 | 0.014 | 0.310 | 0.55960 |
| 3 | 185828465 | rs73052033 | C | T | -0.018 | 0.003 | 3.19963e-13 | 0.0001 | 53.102 | 0.006 | 0.018 | 0.744 | 0.18590 |
| 3 | 108865293 | rs73216701 | G | A | 0.016 | 0.002 | 9.39940e-17 | 0.0001 | 69.083 | -0.039 | 0.013 | 0.004 | 0.41250 |
| 19 | 11347657 | rs737338 | T | C | -0.108 | 0.005 | 2.29985e-91 | 0.0008 | 410.939 | -0.017 | 0.027 | 0.525 | 0.04274 |
| 16 | 68878721 | rs74025321 | G | A | -0.030 | 0.004 | 1.10002e-15 | 0.0001 | 64.177 | 0.048 | 0.026 | 0.071 | 0.07157 |
| 17 | 8107979 | rs7503353 | T | G | -0.014 | 0.002 | 7.29962e-12 | 0.0001 | 46.958 | 0.024 | 0.014 | 0.078 | 0.53580 |
| 6 | 34190104 | rs75104038 | A | G | -0.039 | 0.004 | 1.29987e-21 | 0.0002 | 91.202 | -0.012 | 0.037 | 0.741 | 0.04473 |
| 16 | 56579961 | rs75152587 | T | G | -0.080 | 0.009 | 1.20005e-19 | 0.0002 | 82.276 | 0.019 | 0.062 | 0.757 | 0.01093 |
| 2 | 165518799 | rs75265117 | G | C | 0.032 | 0.003 | 1.20005e-25 | 0.0002 | 109.682 | 0.003 | 0.022 | 0.902 | 0.15510 |
| 10 | 5257647 | rs75406471 | A | G | -0.025 | 0.003 | 1.29987e-19 | 0.0002 | 82.023 | -0.025 | 0.019 | 0.190 | 0.12130 |
| 19 | 11269893 | rs76213248 | T | C | 0.021 | 0.002 | 1.80011e-26 | 0.0002 | 113.323 | 0.001 | 0.014 | 0.955 | 0.43240 |
| 13 | 28604007 | rs76428106 | C | T | -0.061 | 0.009 | 1.29987e-11 | 0.0001 | 45.791 | 0.029 | 0.062 | 0.641 | 0.01392 |
| 7 | 25827967 | rs76456334 | C | T | -0.037 | 0.005 | 7.39946e-13 | 0.0001 | 51.442 | 0.030 | 0.037 | 0.408 | 0.03082 |
| 1 | 224427133 | rs76962725 | A | G | -0.030 | 0.005 | 1.29999e-08 | 0.0001 | 32.338 | -0.013 | 0.052 | 0.797 | 0.03380 |
| 5 | 39546706 | rs7700617 | A | C | -0.013 | 0.002 | 9.89920e-11 | 0.0001 | 41.834 | 0.001 | 0.014 | 0.929 | 0.53680 |
| 2 | 219699999 | rs78058190 | A | G | -0.066 | 0.005 | 2.60016e-39 | 0.0004 | 172.077 | 0.020 | 0.024 | 0.413 | 0.06262 |
| 8 | 144302570 | rs7817574 | C | T | 0.036 | 0.003 | 7.39946e-47 | 0.0004 | 206.639 | 0.008 | 0.015 | 0.602 | 0.20380 |
| 11 | 116648776 | rs78296522 | A | C | 0.068 | 0.005 | 8.99912e-47 | 0.0004 | 206.252 | -0.040 | 0.033 | 0.227 | 0.05169 |
| 6 | 43761091 | rs78807370 | A | G | 0.016 | 0.003 | 8.60003e-09 | 0.0001 | 33.138 | -0.022 | 0.019 | 0.248 | 0.13720 |
| 18 | 46617871 | rs78965095 | T | C | 0.044 | 0.007 | 3.50002e-10 | 0.0001 | 39.397 | 0.001 | 0.056 | 0.985 | 0.02584 |
| 13 | 103553567 | rs79506257 | A | G | 0.028 | 0.005 | 1.20000e-09 | 0.0001 | 36.949 | 0.026 | 0.024 | 0.281 | 0.05567 |
| 11 | 116385354 | rs7952521 | A | G | 0.027 | 0.003 | 2.80027e-16 | 0.0001 | 66.938 | -0.032 | 0.021 | 0.124 | 0.12720 |
| 16 | 56834234 | rs79984435 | A | G | -0.086 | 0.003 | 4.19759e-142 | 0.0012 | 644.155 | -0.015 | 0.023 | 0.510 | 0.09841 |
| 15 | 58972699 | rs80236739 | G | A | -0.032 | 0.005 | 8.19974e-13 | 0.0001 | 51.231 | -0.102 | 0.041 | 0.014 | 0.02286 |
| 15 | 43130646 | rs8028785 | C | T | 0.015 | 0.003 | 7.69999e-09 | 0.0001 | 33.357 | -0.003 | 0.017 | 0.883 | 0.16800 |
| 18 | 47171888 | rs8086351 | G | C | 0.096 | 0.003 | 1.00000e-200 | 0.0027 | 1392.506 | -0.010 | 0.018 | 0.584 | 0.83500 |
| 18 | 21069269 | rs8098618 | T | C | 0.012 | 0.002 | 5.10000e-09 | 0.0001 | 34.155 | 0.013 | 0.013 | 0.325 | 0.50100 |
| 19 | 33900257 | rs8103728 | G | C | 0.023 | 0.002 | 7.29962e-28 | 0.0002 | 119.724 | 0.014 | 0.014 | 0.314 | 0.66800 |
| 12 | 125265201 | rs921919 | A | G | -0.037 | 0.002 | 2.49977e-68 | 0.0006 | 305.103 | -0.001 | 0.013 | 0.961 | 0.67300 |
| 6 | 163740322 | rs9347737 | G | A | -0.011 | 0.002 | 2.90001e-08 | 0.0001 | 30.751 | -0.006 | 0.013 | 0.647 | 0.43140 |
| 1 | 154589965 | rs9426827 | C | T | 0.017 | 0.002 | 2.80027e-18 | 0.0001 | 76.008 | -0.005 | 0.013 | 0.697 | 0.47120 |
| 6 | 42915021 | rs9471972 | A | G | 0.027 | 0.002 | 1.80011e-42 | 0.0004 | 186.560 | -0.009 | 0.013 | 0.491 | 0.53980 |
| 13 | 113927208 | rs9604045 | T | G | 0.015 | 0.002 | 5.79963e-11 | 0.0001 | 42.880 | 0.023 | 0.018 | 0.197 | 0.25650 |
| 22 | 30931307 | rs9608972 | C | T | -0.017 | 0.002 | 2.80027e-14 | 0.0001 | 57.856 | -0.005 | 0.016 | 0.740 | 0.23660 |
| 3 | 135880410 | rs9647335 | T | A | 0.032 | 0.002 | 8.60003e-37 | 0.0003 | 160.538 | 0.035 | 0.021 | 0.095 | 0.19780 |
| 4 | 69343287 | rs976002 | G | A | 0.023 | 0.002 | 4.40048e-24 | 0.0002 | 102.463 | 0.018 | 0.016 | 0.251 | 0.18790 |
| 3 | 156795414 | rs9817452 | T | G | 0.017 | 0.002 | 5.19996e-18 | 0.0001 | 74.820 | 0.012 | 0.014 | 0.395 | 0.38670 |
| 21 | 46907287 | rs9977268 | T | C | -0.018 | 0.002 | 1.50003e-13 | 0.0001 | 54.619 | 0.002 | 0.018 | 0.914 | 0.19780 |
| 8 | 9183358 | rs9987289 | G | A | 0.085 | 0.003 | 1.00000e-138 | 0.0012 | 628.525 | 0.021 | 0.019 | 0.270 | 0.92540 |
| 16 | 56985139 | rs9989419 | G | A | 0.115 | 0.002 | 1.00000e-200 | 0.0063 | 3325.539 | 0.000 | 0.014 | 0.996 | 0.60440 |

**Supplementary table 2.17. Detailed information of instrumental SNPs for Apolipoprotein B in the analysis of PPD.**

| **Chr** | **Position** | **SNP** | **EA** | **OA** | **beta.exposure** | **se.exposure** | **pval.exposure** | **R2** | **F_val** | **beta.outcome** | **se.outcome** | **pval.outcome** | **Eaf.outcome** |
| --- | --- | --- | --- | --- | --- | --- | --- | --- | --- | --- | --- | --- | --- |
| 5 | 131755651 | rs1003533 | T | C | -0.020 | 0.003 | 5.30029e-14 | 0.0001 | 56.621 | -0.038 | 0.016 | 0.019 | 0.19780 |
| 8 | 55433151 | rs10087526 | G | T | 0.019 | 0.003 | 6.20012e-13 | 0.0001 | 51.770 | 0.010 | 0.016 | 0.519 | 0.16700 |
| 14 | 73616095 | rs10151436 | T | A | -0.019 | 0.003 | 9.80009e-09 | 0.0001 | 32.874 | -0.038 | 0.022 | 0.085 | 0.10240 |
| 2 | 158464849 | rs10201242 | A | G | -0.023 | 0.004 | 1.20000e-08 | 0.0001 | 32.455 | 0.036 | 0.029 | 0.205 | 0.05765 |
| 9 | 139320069 | rs10448340 | G | T | -0.017 | 0.002 | 1.99986e-14 | 0.0001 | 58.488 | 0.027 | 0.014 | 0.064 | 0.29720 |
| 10 | 124686656 | rs10794579 | C | T | 0.014 | 0.002 | 6.79986e-11 | 0.0001 | 42.590 | 0.014 | 0.014 | 0.303 | 0.60140 |
| 11 | 18664241 | rs10832963 | G | T | 0.022 | 0.002 | 3.80014e-20 | 0.0002 | 84.533 | -0.011 | 0.014 | 0.421 | 0.71970 |
| 7 | 100216773 | rs10953298 | T | C | -0.017 | 0.002 | 3.69999e-12 | 0.0001 | 48.289 | -0.008 | 0.015 | 0.605 | 0.24850 |
| 12 | 124419728 | rs11057397 | T | C | -0.018 | 0.002 | 7.80010e-17 | 0.0002 | 69.458 | 0.024 | 0.014 | 0.091 | 0.37180 |
| 12 | 125311720 | rs11057837 | T | C | 0.022 | 0.003 | 2.50000e-10 | 0.0001 | 40.050 | -0.016 | 0.020 | 0.446 | 0.11830 |
| 4 | 81167309 | rs11099097 | T | C | -0.016 | 0.002 | 1.80011e-12 | 0.0001 | 49.700 | 0.003 | 0.014 | 0.820 | 0.26540 |
| 17 | 40522713 | rs112220485 | C | T | 0.021 | 0.004 | 3.89996e-08 | 0.0001 | 30.189 | -0.052 | 0.028 | 0.058 | 0.07256 |
| 7 | 97977268 | rs112758337 | A | G | -0.021 | 0.003 | 2.09991e-15 | 0.0001 | 62.984 | 0.018 | 0.019 | 0.327 | 0.18190 |
| 3 | 132217703 | rs113177823 | A | G | -0.041 | 0.005 | 7.19946e-19 | 0.0002 | 78.709 | 0.085 | 0.027 | 0.002 | 0.05070 |
| 1 | 27021913 | rs114165349 | C | G | 0.090 | 0.007 | 7.70016e-39 | 0.0004 | 169.913 | -0.015 | 0.035 | 0.675 | 0.02187 |
| 2 | 234665498 | rs11568318 | A | C | 0.029 | 0.004 | 4.19952e-12 | 0.0001 | 48.013 | 0.050 | 0.039 | 0.197 | 0.04871 |
| 2 | 21077208 | rs115692156 | G | A | -0.112 | 0.012 | 5.10035e-21 | 0.0002 | 88.506 | 0.022 | 0.042 | 0.592 | 0.01093 |
| 1 | 55505647 | rs11591147 | T | G | -0.346 | 0.008 | 1.00000e-200 | 0.0041 | 1917.296 | 0.065 | 0.035 | 0.063 | 0.02087 |
| 11 | 5701074 | rs11601507 | A | C | 0.041 | 0.004 | 1.10002e-24 | 0.0002 | 105.282 | -0.048 | 0.025 | 0.051 | 0.06759 |
| 14 | 24871926 | rs11621792 | T | C | 0.021 | 0.002 | 1.29987e-23 | 0.0002 | 100.384 | 0.050 | 0.014 | 0.000 | 0.47020 |
| 5 | 52095024 | rs116734477 | T | C | -0.063 | 0.005 | 2.49977e-33 | 0.0003 | 144.694 | 0.006 | 0.039 | 0.878 | 0.02883 |
| 19 | 45225423 | rs11673631 | C | G | 0.039 | 0.005 | 3.19963e-14 | 0.0001 | 57.622 | 0.056 | 0.033 | 0.085 | 0.03181 |
| 3 | 142648844 | rs11709868 | T | G | -0.016 | 0.002 | 9.49948e-12 | 0.0001 | 46.421 | -0.021 | 0.015 | 0.174 | 0.33800 |
| 6 | 160922870 | rs117733303 | G | A | 0.086 | 0.008 | 3.59998e-29 | 0.0003 | 125.682 | 0.070 | 0.064 | 0.279 | 0.01193 |
| 6 | 160985526 | rs118039278 | A | G | 0.087 | 0.004 | 2.19786e-111 | 0.0011 | 502.947 | -0.009 | 0.032 | 0.768 | 0.07356 |
| 2 | 43850522 | rs11901691 | A | C | 0.014 | 0.002 | 1.50003e-11 | 0.0001 | 45.567 | -0.010 | 0.013 | 0.472 | 0.57850 |
| 1 | 10799577 | rs12046278 | C | T | -0.016 | 0.002 | 6.40030e-13 | 0.0001 | 51.707 | 0.020 | 0.013 | 0.129 | 0.36280 |
| 3 | 122064369 | rs12054451 | G | T | 0.016 | 0.002 | 2.09991e-11 | 0.0001 | 44.855 | 0.003 | 0.016 | 0.857 | 0.29720 |
| 1 | 16512586 | rs12078100 | G | C | 0.015 | 0.002 | 1.20005e-12 | 0.0001 | 50.563 | 0.007 | 0.015 | 0.648 | 0.61130 |
| 6 | 160543148 | rs12208357 | T | C | 0.063 | 0.004 | 1.00000e-53 | 0.0005 | 238.062 | -0.012 | 0.029 | 0.682 | 0.06262 |
| 4 | 100239319 | rs1229984 | C | T | 0.039 | 0.006 | 1.09999e-09 | 0.0001 | 37.226 | 0.180 | 0.096 | 0.060 | 0.97120 |
| 2 | 135629927 | rs12469941 | T | C | 0.013 | 0.002 | 1.50000e-10 | 0.0001 | 40.969 | -0.031 | 0.013 | 0.018 | 0.55170 |
| 2 | 64928603 | rs12471768 | C | T | 0.014 | 0.002 | 8.79995e-10 | 0.0001 | 37.570 | -0.024 | 0.016 | 0.134 | 0.71370 |
| 2 | 216300185 | rs1250258 | T | C | 0.014 | 0.002 | 1.89998e-09 | 0.0001 | 36.083 | 0.006 | 0.016 | 0.712 | 0.76640 |
| 16 | 71855220 | rs12597418 | A | G | 0.018 | 0.002 | 2.90001e-17 | 0.0002 | 71.391 | -0.025 | 0.014 | 0.073 | 0.33200 |
| 2 | 27730940 | rs1260326 | C | T | -0.050 | 0.002 | 1.90108e-121 | 0.0012 | 549.149 | 0.004 | 0.014 | 0.762 | 0.58950 |
| 17 | 45650196 | rs12603290 | C | T | -0.029 | 0.002 | 9.79941e-44 | 0.0004 | 192.333 | 0.010 | 0.013 | 0.468 | 0.53780 |
| 19 | 45418486 | rs12691088 | A | G | 0.246 | 0.008 | 1.00000e-200 | 0.0025 | 1022.709 | 0.041 | 0.037 | 0.272 | 0.02187 |
| 10 | 18494666 | rs1277762 | T | C | 0.017 | 0.003 | 2.19999e-09 | 0.0001 | 35.796 | 0.018 | 0.017 | 0.282 | 0.83300 |
| 5 | 74656539 | rs12916 | C | T | 0.055 | 0.002 | 5.19996e-148 | 0.0014 | 671.287 | 0.010 | 0.013 | 0.461 | 0.41250 |
| 3 | 12327431 | rs13076933 | G | T | -0.018 | 0.002 | 1.80011e-14 | 0.0001 | 58.757 | 0.018 | 0.015 | 0.221 | 0.25650 |
| 4 | 3443931 | rs13108218 | G | A | -0.023 | 0.002 | 1.59993e-26 | 0.0002 | 113.534 | -0.029 | 0.014 | 0.041 | 0.61030 |
| 7 | 130437124 | rs13230111 | G | A | -0.014 | 0.002 | 2.80027e-11 | 0.0001 | 44.300 | -0.002 | 0.013 | 0.870 | 0.44730 |
| 7 | 73010442 | rs13247874 | T | C | -0.022 | 0.003 | 1.39991e-16 | 0.0001 | 68.275 | -0.032 | 0.017 | 0.069 | 0.18990 |
| 14 | 74250126 | rs13379043 | C | T | -0.014 | 0.002 | 1.09999e-09 | 0.0001 | 37.102 | 0.009 | 0.017 | 0.584 | 0.29820 |
| 2 | 165528876 | rs13389219 | T | C | -0.016 | 0.002 | 2.60016e-14 | 0.0001 | 57.986 | 0.002 | 0.014 | 0.887 | 0.43540 |
| 6 | 43764551 | rs1358980 | T | C | 0.015 | 0.002 | 1.10002e-12 | 0.0001 | 50.632 | -0.008 | 0.013 | 0.564 | 0.49700 |
| 8 | 19824492 | rs13702 | C | T | -0.030 | 0.002 | 7.80010e-38 | 0.0004 | 165.318 | -0.007 | 0.015 | 0.665 | 0.32010 |
| 2 | 21086334 | rs138026891 | T | C | -0.069 | 0.010 | 1.59993e-11 | 0.0001 | 45.379 | 0.131 | 0.053 | 0.014 | 0.01193 |
| 22 | 41272143 | rs138354 | C | T | -0.012 | 0.002 | 6.90001e-09 | 0.0001 | 33.551 | 0.034 | 0.013 | 0.010 | 0.52680 |
| 19 | 45467132 | rs138692741 | T | C | 0.071 | 0.006 | 2.99985e-36 | 0.0004 | 158.092 | 0.008 | 0.037 | 0.831 | 0.03380 |
| 19 | 11187324 | rs143020224 | G | C | -0.165 | 0.003 | 1.00000e-200 | 0.0057 | 2703.568 | -0.021 | 0.022 | 0.336 | 0.11530 |
| 6 | 11839042 | rs147539187 | G | C | -0.023 | 0.004 | 1.09999e-08 | 0.0001 | 32.628 | 0.030 | 0.044 | 0.495 | 0.05169 |
| 19 | 45302504 | rs148933445 | A | G | -0.573 | 0.008 | 1.00000e-200 | 0.0139 | 5701.635 | 0.002 | 0.047 | 0.969 | 0.01491 |
| 2 | 118845121 | rs150474434 | A | G | -0.034 | 0.003 | 2.80027e-23 | 0.0002 | 98.789 | -0.058 | 0.025 | 0.019 | 0.08946 |
| 19 | 45579242 | rs150503754 | C | T | -0.052 | 0.009 | 2.39999e-09 | 0.0001 | 35.617 | -0.010 | 0.039 | 0.790 | 0.01789 |
| 19 | 45227155 | rs150820726 | T | A | 0.072 | 0.011 | 1.10002e-11 | 0.0001 | 46.148 | 0.006 | 0.032 | 0.858 | 0.01988 |
| 1 | 93034023 | rs1556562 | T | G | 0.016 | 0.002 | 1.09999e-10 | 0.0001 | 41.667 | 0.007 | 0.016 | 0.639 | 0.80720 |
| 16 | 56852822 | rs1561139 | T | G | -0.013 | 0.002 | 4.20001e-10 | 0.0001 | 39.008 | -0.007 | 0.014 | 0.617 | 0.38970 |
| 1 | 109834039 | rs17036085 | G | A | -0.068 | 0.009 | 4.00037e-13 | 0.0001 | 52.664 | -0.051 | 0.033 | 0.129 | 0.01292 |
| 2 | 121306440 | rs17050272 | A | G | -0.024 | 0.002 | 3.80014e-31 | 0.0003 | 134.739 | -0.030 | 0.013 | 0.026 | 0.45030 |
| 11 | 61588305 | rs174564 | G | A | -0.045 | 0.002 | 1.00000e-94 | 0.0009 | 426.380 | 0.011 | 0.013 | 0.431 | 0.35290 |
| 10 | 71094504 | rs17476364 | C | T | -0.021 | 0.003 | 4.00000e-10 | 0.0001 | 39.108 | 0.040 | 0.028 | 0.162 | 0.09940 |
| 20 | 62896110 | rs17569873 | T | C | 0.018 | 0.003 | 1.59993e-12 | 0.0001 | 49.978 | -0.025 | 0.019 | 0.204 | 0.19380 |
| 16 | 56991363 | rs183130 | T | C | -0.048 | 0.002 | 1.30017e-102 | 0.0010 | 462.635 | -0.002 | 0.015 | 0.882 | 0.29320 |
| 1 | 109725404 | rs188608977 | T | G | 0.062 | 0.010 | 2.10000e-09 | 0.0001 | 35.894 | -0.007 | 0.062 | 0.906 | 0.01590 |
| 21 | 40686521 | rs1888488 | T | C | 0.017 | 0.002 | 1.50003e-16 | 0.0001 | 68.161 | -0.006 | 0.014 | 0.684 | 0.59240 |
| 15 | 58680954 | rs2043085 | C | T | -0.019 | 0.002 | 1.20005e-19 | 0.0002 | 82.176 | 0.022 | 0.013 | 0.102 | 0.63220 |
| 10 | 94839642 | rs2068888 | A | G | -0.024 | 0.002 | 5.19996e-31 | 0.0003 | 134.098 | -0.014 | 0.013 | 0.299 | 0.48610 |
| 7 | 44582331 | rs2073547 | G | A | 0.031 | 0.003 | 1.10002e-31 | 0.0003 | 137.185 | -0.012 | 0.014 | 0.391 | 0.19480 |
| 4 | 26080549 | rs2137234 | C | T | 0.015 | 0.003 | 5.69994e-09 | 0.0001 | 33.936 | 0.004 | 0.019 | 0.810 | 0.22660 |
| 12 | 50650057 | rs2160994 | C | T | 0.017 | 0.002 | 2.09991e-15 | 0.0001 | 62.927 | -0.010 | 0.014 | 0.466 | 0.62030 |
| 17 | 73868288 | rs2199048 | G | A | -0.013 | 0.002 | 3.79997e-09 | 0.0001 | 34.733 | -0.001 | 0.016 | 0.961 | 0.33100 |
| 13 | 32959199 | rs2238162 | T | C | -0.023 | 0.002 | 1.50003e-27 | 0.0003 | 118.305 | 0.020 | 0.013 | 0.145 | 0.51590 |
| 20 | 34107160 | rs224391 | C | G | -0.017 | 0.002 | 1.99986e-12 | 0.0001 | 49.445 | -0.005 | 0.016 | 0.770 | 0.25050 |
| 20 | 62373983 | rs2256814 | A | G | 0.015 | 0.003 | 2.90001e-08 | 0.0001 | 30.792 | -0.020 | 0.017 | 0.240 | 0.16800 |
| 2 | 169830328 | rs2287622 | G | A | -0.020 | 0.002 | 7.70016e-22 | 0.0002 | 92.233 | -0.011 | 0.013 | 0.423 | 0.60440 |
| 12 | 53778650 | rs2446066 | T | G | 0.016 | 0.003 | 1.20000e-08 | 0.0001 | 32.411 | -0.001 | 0.018 | 0.944 | 0.16600 |
| 20 | 17844684 | rs2618566 | T | G | -0.030 | 0.002 | 1.99986e-43 | 0.0004 | 190.906 | 0.031 | 0.014 | 0.029 | 0.61630 |
| 8 | 116667539 | rs2737263 | T | G | -0.019 | 0.002 | 1.20005e-15 | 0.0001 | 64.128 | -0.016 | 0.014 | 0.267 | 0.23960 |
| 19 | 11227480 | rs2738447 | C | A | 0.044 | 0.002 | 9.89920e-97 | 0.0009 | 435.587 | 0.017 | 0.014 | 0.222 | 0.56460 |
| 10 | 95052021 | rs2761311 | T | C | 0.012 | 0.002 | 1.00000e-08 | 0.0001 | 32.757 | 0.005 | 0.013 | 0.680 | 0.56560 |
| 4 | 40428010 | rs278981 | C | T | 0.015 | 0.002 | 6.10000e-10 | 0.0001 | 38.299 | -0.005 | 0.016 | 0.776 | 0.78430 |
| 1 | 221029841 | rs2807854 | C | T | 0.016 | 0.002 | 1.39991e-13 | 0.0001 | 54.642 | -0.009 | 0.014 | 0.501 | 0.65410 |
| 7 | 21449451 | rs28406917 | T | C | 0.013 | 0.002 | 2.00000e-10 | 0.0001 | 40.436 | 0.013 | 0.013 | 0.323 | 0.41350 |
| 5 | 71932487 | rs28590710 | A | G | -0.018 | 0.003 | 9.09913e-11 | 0.0001 | 42.007 | 0.006 | 0.016 | 0.686 | 0.16200 |
| 8 | 126500031 | rs28601761 | G | C | -0.073 | 0.002 | 1.00000e-200 | 0.0026 | 1162.066 | 0.008 | 0.013 | 0.533 | 0.40760 |
| 16 | 72101525 | rs34042070 | G | C | 0.049 | 0.003 | 1.90020e-76 | 0.0007 | 342.376 | 0.015 | 0.017 | 0.379 | 0.20180 |
| 19 | 58662235 | rs35081008 | T | C | -0.031 | 0.003 | 7.80010e-27 | 0.0002 | 115.013 | -0.003 | 0.020 | 0.886 | 0.15310 |
| 8 | 18254509 | rs35583283 | C | G | 0.016 | 0.003 | 2.30001e-08 | 0.0001 | 31.192 | -0.008 | 0.017 | 0.624 | 0.15410 |
| 2 | 21477029 | rs35764292 | G | A | 0.051 | 0.005 | 1.29987e-21 | 0.0002 | 91.256 | -0.020 | 0.042 | 0.634 | 0.04573 |
| 12 | 623129 | rs35882350 | G | A | 0.014 | 0.002 | 1.00000e-08 | 0.0001 | 32.801 | 0.000 | 0.015 | 0.977 | 0.25150 |
| 20 | 25208272 | rs3746337 | T | C | 0.016 | 0.002 | 5.10035e-14 | 0.0001 | 56.698 | -0.005 | 0.013 | 0.689 | 0.46420 |
| 9 | 2640759 | rs3780181 | G | A | -0.026 | 0.004 | 4.70002e-10 | 0.0001 | 38.794 | 0.026 | 0.029 | 0.380 | 0.05268 |
| 6 | 116316882 | rs3822855 | T | G | 0.015 | 0.002 | 2.39994e-12 | 0.0001 | 49.149 | -0.011 | 0.013 | 0.398 | 0.42450 |
| 11 | 126244955 | rs4307732 | A | G | 0.048 | 0.003 | 5.79963e-46 | 0.0004 | 202.537 | -0.030 | 0.018 | 0.093 | 0.10640 |
| 7 | 21604916 | rs4470903 | G | C | 0.035 | 0.003 | 2.60016e-42 | 0.0004 | 185.857 | -0.004 | 0.016 | 0.788 | 0.18890 |
| 19 | 49114236 | rs454715 | G | T | -0.024 | 0.002 | 2.39994e-29 | 0.0003 | 126.518 | 0.012 | 0.015 | 0.432 | 0.62920 |
| 7 | 87110640 | rs45537841 | T | C | -0.016 | 0.003 | 4.09996e-09 | 0.0001 | 34.566 | 0.017 | 0.017 | 0.311 | 0.19090 |
| 2 | 62988169 | rs4671050 | T | G | -0.020 | 0.002 | 1.00000e-18 | 0.0002 | 78.045 | 0.006 | 0.014 | 0.664 | 0.35190 |
| 3 | 52492085 | rs4687614 | A | G | 0.027 | 0.005 | 3.69999e-09 | 0.0001 | 34.769 | 0.057 | 0.036 | 0.110 | 0.93540 |
| 4 | 7222253 | rs4689088 | A | G | 0.012 | 0.002 | 5.89997e-09 | 0.0001 | 33.867 | -0.025 | 0.014 | 0.066 | 0.63420 |
| 1 | 55521313 | rs472495 | T | G | 0.041 | 0.002 | 1.29987e-78 | 0.0008 | 352.317 | -0.015 | 0.013 | 0.277 | 0.62520 |
| 16 | 83980529 | rs4782568 | G | C | -0.018 | 0.002 | 3.19963e-18 | 0.0002 | 75.765 | 0.008 | 0.013 | 0.561 | 0.46120 |
| 1 | 234773373 | rs478975 | A | G | 0.014 | 0.002 | 7.89951e-11 | 0.0001 | 42.281 | 0.006 | 0.013 | 0.629 | 0.62430 |
| 19 | 45914283 | rs4803818 | T | C | 0.042 | 0.007 | 1.29999e-09 | 0.0001 | 36.880 | -0.016 | 0.034 | 0.631 | 0.01988 |
| 11 | 30533622 | rs546240 | T | C | -0.012 | 0.002 | 3.09999e-08 | 0.0001 | 30.616 | -0.002 | 0.013 | 0.878 | 0.59150 |
| 1 | 234853059 | rs556107 | T | C | 0.036 | 0.002 | 1.29987e-66 | 0.0006 | 297.210 | 0.007 | 0.013 | 0.609 | 0.51890 |
| 1 | 55466303 | rs55637835 | T | C | -0.019 | 0.003 | 6.10000e-09 | 0.0001 | 33.799 | 0.041 | 0.022 | 0.064 | 0.10240 |
| 17 | 7080316 | rs55714927 | T | C | -0.032 | 0.003 | 5.10035e-34 | 0.0003 | 147.841 | -0.015 | 0.015 | 0.310 | 0.18390 |
| 8 | 145031968 | rs55831924 | T | C | 0.018 | 0.002 | 4.79954e-17 | 0.0002 | 70.421 | 0.003 | 0.014 | 0.834 | 0.37870 |
| 9 | 15305378 | rs581080 | C | G | 0.019 | 0.003 | 6.40030e-12 | 0.0001 | 47.189 | -0.019 | 0.019 | 0.326 | 0.81710 |
| 4 | 124758773 | rs58148580 | T | C | 0.021 | 0.003 | 9.60064e-11 | 0.0001 | 41.907 | 0.019 | 0.021 | 0.360 | 0.11630 |
| 8 | 21928227 | rs59328596 | A | G | -0.021 | 0.003 | 6.59933e-13 | 0.0001 | 51.667 | 0.021 | 0.017 | 0.217 | 0.15110 |
| 12 | 111973358 | rs597808 | G | A | 0.022 | 0.002 | 2.39994e-25 | 0.0002 | 108.205 | 0.010 | 0.013 | 0.448 | 0.53380 |
| 20 | 39755154 | rs6072279 | A | G | 0.029 | 0.002 | 8.69961e-45 | 0.0004 | 197.151 | 0.023 | 0.013 | 0.080 | 0.46020 |
| 20 | 44551855 | rs6073958 | C | T | 0.042 | 0.003 | 2.49977e-59 | 0.0006 | 263.802 | 0.005 | 0.017 | 0.793 | 0.22070 |
| 20 | 39144286 | rs6129620 | A | T | -0.025 | 0.002 | 8.69961e-30 | 0.0003 | 128.498 | 0.005 | 0.015 | 0.755 | 0.30520 |
| 2 | 21216815 | rs62122481 | A | C | 0.084 | 0.002 | 1.00000e-200 | 0.0033 | 1537.840 | -0.021 | 0.014 | 0.144 | 0.30120 |
| 1 | 246891260 | rs6426328 | T | G | 0.012 | 0.002 | 4.00000e-09 | 0.0001 | 34.638 | -0.007 | 0.014 | 0.610 | 0.48510 |
| 9 | 22081850 | rs6475606 | T | C | -0.017 | 0.002 | 7.19946e-17 | 0.0002 | 69.620 | 0.018 | 0.013 | 0.191 | 0.48510 |
| 9 | 78730766 | rs6560499 | A | G | -0.012 | 0.002 | 8.99995e-09 | 0.0001 | 33.057 | 0.010 | 0.014 | 0.483 | 0.55070 |
| 13 | 114551993 | rs6602909 | C | T | 0.021 | 0.002 | 4.60045e-22 | 0.0002 | 93.254 | -0.022 | 0.014 | 0.110 | 0.36280 |
| 1 | 109807283 | rs6657811 | T | A | -0.128 | 0.003 | 1.00000e-200 | 0.0037 | 1713.258 | -0.012 | 0.022 | 0.588 | 0.11130 |
| 21 | 33096103 | rs67038483 | T | C | 0.028 | 0.005 | 2.99999e-08 | 0.0001 | 30.691 | 0.007 | 0.024 | 0.764 | 0.05666 |
| 5 | 156391628 | rs6874202 | C | T | 0.031 | 0.002 | 1.59993e-47 | 0.0005 | 209.709 | -0.002 | 0.014 | 0.905 | 0.64710 |
| 6 | 42924932 | rs6940814 | G | A | -0.020 | 0.002 | 2.99985e-21 | 0.0002 | 89.525 | -0.001 | 0.013 | 0.954 | 0.58950 |
| 8 | 9173209 | rs7012637 | A | G | 0.020 | 0.002 | 1.29987e-21 | 0.0002 | 91.254 | 0.002 | 0.013 | 0.852 | 0.47320 |
| 11 | 5677158 | rs7108486 | C | T | -0.043 | 0.007 | 5.19996e-10 | 0.0001 | 38.613 | -0.044 | 0.046 | 0.330 | 0.02187 |
| 3 | 58420613 | rs71311871 | G | A | -0.026 | 0.004 | 2.90001e-12 | 0.0001 | 48.760 | 0.017 | 0.025 | 0.496 | 0.08350 |
| 19 | 11302807 | rs7249565 | A | G | 0.015 | 0.002 | 2.90001e-12 | 0.0001 | 48.779 | -0.002 | 0.013 | 0.894 | 0.38370 |
| 17 | 67191270 | rs72631343 | G | C | -0.028 | 0.003 | 2.29985e-19 | 0.0002 | 80.949 | -0.009 | 0.019 | 0.637 | 0.12720 |
| 4 | 74177397 | rs72663045 | G | T | 0.042 | 0.007 | 8.00000e-09 | 0.0001 | 33.268 | 0.009 | 0.050 | 0.853 | 0.02187 |
| 10 | 115786233 | rs72823013 | A | G | -0.023 | 0.003 | 3.19963e-13 | 0.0001 | 53.062 | 0.017 | 0.023 | 0.463 | 0.11430 |
| 17 | 17432220 | rs74454529 | A | G | 0.028 | 0.004 | 1.29999e-10 | 0.0001 | 41.325 | -0.026 | 0.024 | 0.284 | 0.07256 |
| 2 | 44069772 | rs75331444 | A | G | -0.090 | 0.004 | 1.99986e-103 | 0.0010 | 466.392 | 0.035 | 0.024 | 0.143 | 0.07952 |
| 2 | 203527979 | rs7569317 | C | T | 0.018 | 0.002 | 2.60016e-18 | 0.0002 | 76.163 | -0.003 | 0.013 | 0.821 | 0.51490 |
| 2 | 44077225 | rs7590687 | C | T | -0.043 | 0.004 | 3.40017e-27 | 0.0003 | 116.668 | 0.019 | 0.027 | 0.478 | 0.92840 |
| 2 | 204317553 | rs7603427 | T | C | 0.014 | 0.002 | 6.59933e-11 | 0.0001 | 42.639 | 0.027 | 0.013 | 0.042 | 0.53080 |
| 1 | 109840169 | rs76186504 | T | C | -0.116 | 0.007 | 3.29989e-69 | 0.0007 | 309.175 | -0.086 | 0.051 | 0.094 | 0.02187 |
| 1 | 62930106 | rs77083979 | A | G | -0.019 | 0.003 | 2.00000e-09 | 0.0001 | 35.969 | 0.016 | 0.023 | 0.487 | 0.11130 |
| 5 | 122848876 | rs7734476 | A | G | 0.020 | 0.002 | 1.20005e-21 | 0.0002 | 91.317 | 0.007 | 0.013 | 0.620 | 0.58750 |
| 6 | 16126934 | rs7746081 | A | G | -0.023 | 0.002 | 6.70039e-24 | 0.0002 | 101.622 | 0.021 | 0.014 | 0.122 | 0.33600 |
| 11 | 66183801 | rs77498041 | A | G | -0.016 | 0.003 | 8.50002e-10 | 0.0001 | 37.648 | 0.001 | 0.017 | 0.961 | 0.16600 |
| 19 | 45513854 | rs79149284 | T | C | 0.034 | 0.005 | 3.20000e-10 | 0.0001 | 39.545 | -0.096 | 0.039 | 0.014 | 0.02485 |
| 6 | 26098474 | rs79220007 | C | T | -0.054 | 0.004 | 4.19952e-43 | 0.0004 | 189.459 | 0.008 | 0.035 | 0.817 | 0.04274 |
| 14 | 70798289 | rs8016418 | A | T | 0.020 | 0.002 | 4.60045e-20 | 0.0002 | 84.135 | -0.010 | 0.013 | 0.474 | 0.36380 |
| 10 | 52373245 | rs80276949 | A | G | 0.042 | 0.007 | 2.10000e-09 | 0.0001 | 35.893 | 0.079 | 0.061 | 0.192 | 0.02087 |
| 19 | 19388500 | rs8107974 | T | A | -0.091 | 0.004 | 4.49780e-121 | 0.0012 | 547.478 | -0.016 | 0.027 | 0.552 | 0.06859 |
| 8 | 59392324 | rs9297994 | A | G | -0.029 | 0.002 | 6.20012e-40 | 0.0004 | 174.932 | 0.019 | 0.014 | 0.169 | 0.63220 |
| 6 | 127446790 | rs9482772 | C | T | 0.018 | 0.002 | 2.19989e-17 | 0.0002 | 71.956 | -0.040 | 0.013 | 0.003 | 0.41050 |
| 6 | 100602753 | rs9496567 | A | G | -0.019 | 0.002 | 3.80014e-15 | 0.0001 | 61.790 | 0.013 | 0.016 | 0.422 | 0.23760 |
| 22 | 50840573 | rs9616822 | A | G | 0.016 | 0.002 | 2.70023e-13 | 0.0001 | 53.396 | 0.014 | 0.013 | 0.292 | 0.34490 |
| 11 | 116648917 | rs964184 | C | G | -0.077 | 0.003 | 2.50035e-138 | 0.0014 | 626.776 | -0.022 | 0.019 | 0.248 | 0.83800 |
| 20 | 17792323 | rs969075 | C | T | 0.014 | 0.002 | 4.39997e-10 | 0.0001 | 38.912 | 0.013 | 0.015 | 0.382 | 0.67990 |
| 3 | 32535382 | rs9834932 | G | A | -0.032 | 0.004 | 3.90032e-18 | 0.0002 | 75.394 | 0.049 | 0.024 | 0.039 | 0.11230 |
| 4 | 69373407 | rs9884390 | C | T | 0.023 | 0.002 | 7.80010e-21 | 0.0002 | 87.651 | 0.000 | 0.016 | 0.984 | 0.17790 |
| 17 | 7571080 | rs9894946 | G | A | -0.017 | 0.003 | 3.09999e-09 | 0.0001 | 35.139 | -0.029 | 0.019 | 0.122 | 0.82900 |

**Supplementary table 2.18. Detailed information of instrumental SNPs for Testosterone in the analysis of PPD.**

| **Chr** | **Position** | **SNP** | **EA** | **OA** | **beta.exposure** | **se.exposure** | **pval.exposure** | **R2** | **F_val** | **beta.outcome** | **se.outcome** | **pval.outcome** | **Eaf.outcome** |
| --- | --- | --- | --- | --- | --- | --- | --- | --- | --- | --- | --- | --- | --- |
| 2 | 20277070 | rs10184382 | T | C | 0.020 | 0.003 | 2.00000e-10 | 0.0002 | 40.477 | -0.014 | 0.014 | 0.313 | 0.43340 |
| 9 | 114828332 | rs10817260 | T | C | -0.038 | 0.004 | 3.59998e-20 | 0.0004 | 84.647 | 0.000 | 0.015 | 0.989 | 0.19880 |
| 10 | 64934548 | rs10822145 | T | C | 0.024 | 0.003 | 5.79963e-14 | 0.0003 | 56.434 | -0.013 | 0.013 | 0.339 | 0.49500 |
| 17 | 53372566 | rs10852974 | C | G | -0.019 | 0.003 | 5.30005e-09 | 0.0002 | 34.071 | 0.016 | 0.014 | 0.243 | 0.59540 |
| 8 | 23401534 | rs10866828 | T | C | 0.023 | 0.004 | 4.90004e-10 | 0.0002 | 38.730 | 0.000 | 0.015 | 0.985 | 0.25840 |
| 10 | 96825332 | rs10882525 | T | A | -0.019 | 0.003 | 5.69994e-09 | 0.0002 | 33.943 | 0.015 | 0.014 | 0.303 | 0.35590 |
| 11 | 30226356 | rs11031005 | C | T | 0.027 | 0.005 | 1.29999e-09 | 0.0002 | 36.880 | 0.004 | 0.018 | 0.841 | 0.14020 |
| 11 | 118308499 | rs111903103 | T | C | -0.044 | 0.008 | 2.19999e-08 | 0.0002 | 31.341 | 0.086 | 0.039 | 0.027 | 0.03678 |
| 5 | 76488613 | rs1119208 | T | C | -0.026 | 0.003 | 3.59998e-15 | 0.0003 | 61.921 | -0.021 | 0.013 | 0.116 | 0.36380 |
| 4 | 69946371 | rs11249524 | C | T | -0.026 | 0.003 | 5.90065e-16 | 0.0003 | 65.467 | -0.014 | 0.013 | 0.299 | 0.51490 |
| 8 | 143954290 | rs1134095 | A | G | -0.036 | 0.003 | 8.60003e-31 | 0.0007 | 133.097 | -0.005 | 0.014 | 0.710 | 0.59340 |
| 19 | 41985931 | rs11673591 | T | A | -0.027 | 0.004 | 1.00000e-13 | 0.0003 | 55.357 | 0.000 | 0.015 | 0.988 | 0.74650 |
| 2 | 43282963 | rs11676745 | C | G | 0.021 | 0.003 | 2.99999e-10 | 0.0002 | 39.699 | -0.005 | 0.014 | 0.735 | 0.65510 |
| 2 | 62538681 | rs11683263 | C | T | -0.018 | 0.003 | 1.79999e-08 | 0.0002 | 31.695 | -0.011 | 0.014 | 0.427 | 0.56660 |
| 2 | 112380559 | rs11683361 | G | C | 0.026 | 0.004 | 3.19963e-11 | 0.0002 | 44.068 | 0.008 | 0.016 | 0.629 | 0.78330 |
| 10 | 61467182 | rs1171617 | T | G | 0.041 | 0.004 | 2.39994e-28 | 0.0006 | 121.953 | -0.020 | 0.018 | 0.261 | 0.75650 |
| 8 | 105978368 | rs11774829 | A | T | 0.047 | 0.005 | 7.39946e-19 | 0.0004 | 78.650 | 0.015 | 0.018 | 0.408 | 0.09940 |
| 2 | 24990082 | rs11892043 | G | A | -0.025 | 0.004 | 3.50026e-12 | 0.0002 | 48.367 | 0.008 | 0.014 | 0.568 | 0.28030 |
| 1 | 155929562 | rs12091273 | T | C | -0.022 | 0.004 | 2.80001e-08 | 0.0002 | 30.844 | -0.014 | 0.017 | 0.408 | 0.18190 |
| 1 | 113138110 | rs12143068 | G | A | -0.025 | 0.004 | 8.69961e-12 | 0.0002 | 46.605 | -0.013 | 0.016 | 0.412 | 0.24060 |
| 6 | 43352980 | rs1214759 | G | A | 0.032 | 0.003 | 5.40008e-22 | 0.0005 | 92.931 | -0.028 | 0.014 | 0.054 | 0.62920 |
| 14 | 98550490 | rs12436785 | C | T | 0.029 | 0.003 | 7.19946e-20 | 0.0004 | 83.247 | 0.016 | 0.013 | 0.241 | 0.44040 |
| 2 | 27730940 | rs1260326 | C | T | 0.028 | 0.003 | 2.80027e-18 | 0.0004 | 75.995 | 0.004 | 0.014 | 0.762 | 0.58950 |
| 5 | 124205385 | rs12658172 | C | G | -0.042 | 0.004 | 2.19989e-21 | 0.0005 | 90.188 | 0.020 | 0.018 | 0.274 | 0.16500 |
| 9 | 16252807 | rs12683780 | C | A | -0.027 | 0.003 | 1.29987e-15 | 0.0003 | 63.865 | 0.010 | 0.014 | 0.467 | 0.34890 |
| 14 | 68685460 | rs1296527 | C | T | -0.025 | 0.004 | 1.79999e-08 | 0.0002 | 31.685 | 0.005 | 0.020 | 0.804 | 0.17100 |
| 2 | 12567937 | rs13022088 | G | A | 0.028 | 0.004 | 2.80027e-15 | 0.0003 | 62.422 | 0.016 | 0.014 | 0.233 | 0.29030 |
| 3 | 27563686 | rs13091026 | T | C | 0.056 | 0.010 | 2.59998e-08 | 0.0002 | 30.959 | 0.057 | 0.032 | 0.074 | 0.02883 |
| 5 | 176782218 | rs13153019 | C | T | 0.023 | 0.004 | 3.50002e-10 | 0.0002 | 39.384 | 0.034 | 0.015 | 0.023 | 0.26440 |
| 1 | 51243374 | rs1416685 | C | G | -0.018 | 0.003 | 1.40001e-08 | 0.0002 | 32.223 | -0.019 | 0.015 | 0.212 | 0.41250 |
| 2 | 178221668 | rs1453373 | A | G | -0.020 | 0.003 | 2.50000e-09 | 0.0002 | 35.535 | -0.002 | 0.014 | 0.862 | 0.64910 |
| 13 | 73131694 | rs17245822 | C | A | 0.023 | 0.003 | 3.80014e-12 | 0.0002 | 48.237 | -0.014 | 0.014 | 0.299 | 0.35880 |
| 1 | 218548521 | rs17558745 | T | C | -0.020 | 0.003 | 3.89996e-09 | 0.0002 | 34.690 | -0.018 | 0.014 | 0.188 | 0.29520 |
| 6 | 127226814 | rs17649719 | C | G | -0.021 | 0.003 | 1.29999e-10 | 0.0002 | 41.284 | 0.009 | 0.013 | 0.508 | 0.40850 |
| 5 | 128015370 | rs17677724 | T | C | -0.025 | 0.004 | 3.59998e-09 | 0.0002 | 34.816 | 0.004 | 0.020 | 0.858 | 0.16600 |
| 8 | 101330957 | rs1788174 | C | G | -0.019 | 0.003 | 5.69994e-09 | 0.0002 | 33.937 | -0.006 | 0.014 | 0.658 | 0.37770 |
| 20 | 48905967 | rs1889236 | T | C | -0.033 | 0.004 | 3.29989e-18 | 0.0004 | 75.702 | -0.001 | 0.014 | 0.952 | 0.24650 |
| 3 | 169179876 | rs1918975 | T | C | -0.021 | 0.003 | 9.09913e-11 | 0.0002 | 42.015 | -0.012 | 0.013 | 0.383 | 0.60440 |
| 1 | 7909373 | rs1989147 | T | C | -0.026 | 0.004 | 1.60000e-10 | 0.0002 | 40.954 | -0.026 | 0.016 | 0.100 | 0.18990 |
| 21 | 18289770 | rs2142361 | G | C | -0.028 | 0.005 | 3.69999e-09 | 0.0002 | 34.765 | -0.023 | 0.023 | 0.320 | 0.87180 |
| 6 | 31603591 | rs2261033 | G | A | 0.027 | 0.003 | 3.19963e-17 | 0.0003 | 71.235 | -0.024 | 0.014 | 0.082 | 0.51290 |
| 2 | 48921375 | rs2293275 | C | T | -0.019 | 0.003 | 3.09999e-09 | 0.0002 | 35.147 | -0.005 | 0.013 | 0.737 | 0.64020 |
| 3 | 195942487 | rs2342307 | G | A | -0.029 | 0.003 | 2.29985e-18 | 0.0004 | 76.441 | -0.015 | 0.014 | 0.266 | 0.63920 |
| 6 | 170588559 | rs2344744 | G | T | 0.018 | 0.003 | 3.20000e-08 | 0.0002 | 30.604 | 0.020 | 0.013 | 0.129 | 0.44730 |
| 5 | 142424022 | rs246660 | T | C | 0.022 | 0.004 | 2.99999e-09 | 0.0002 | 35.199 | -0.004 | 0.015 | 0.789 | 0.22170 |
| 10 | 5048014 | rs2801885 | G | A | -0.027 | 0.004 | 4.90004e-14 | 0.0003 | 56.777 | 0.030 | 0.015 | 0.048 | 0.26540 |
| 1 | 68839242 | rs3118386 | C | T | 0.018 | 0.003 | 1.70000e-08 | 0.0002 | 31.844 | -0.019 | 0.013 | 0.163 | 0.37570 |
| 11 | 68097049 | rs312023 | A | G | -0.024 | 0.003 | 4.60045e-14 | 0.0003 | 56.910 | -0.006 | 0.014 | 0.661 | 0.53880 |
| 7 | 73037956 | rs34060476 | G | A | 0.044 | 0.005 | 1.20005e-21 | 0.0005 | 91.426 | -0.025 | 0.020 | 0.210 | 0.12330 |
| 17 | 66879927 | rs34931250 | T | C | -0.044 | 0.007 | 3.90032e-11 | 0.0002 | 43.655 | -0.016 | 0.027 | 0.548 | 0.04970 |
| 3 | 23614855 | rs35169377 | C | A | -0.019 | 0.003 | 2.80001e-08 | 0.0002 | 30.840 | -0.010 | 0.014 | 0.469 | 0.33900 |
| 10 | 5062752 | rs36032941 | A | C | -0.056 | 0.003 | 5.10035e-57 | 0.0013 | 253.256 | -0.008 | 0.015 | 0.578 | 0.29520 |
| 6 | 119167949 | rs36182456 | A | G | 0.052 | 0.004 | 2.70023e-42 | 0.0009 | 185.745 | -0.013 | 0.018 | 0.464 | 0.79920 |
| 11 | 72288843 | rs371862 | T | C | -0.025 | 0.003 | 1.20005e-13 | 0.0003 | 54.986 | 0.001 | 0.014 | 0.941 | 0.31010 |
| 12 | 49167683 | rs3730074 | A | C | -0.025 | 0.004 | 1.70000e-08 | 0.0002 | 31.756 | -0.030 | 0.022 | 0.184 | 0.12520 |
| 18 | 3813758 | rs3786432 | T | C | 0.021 | 0.004 | 5.99998e-09 | 0.0002 | 33.839 | -0.014 | 0.014 | 0.322 | 0.71770 |
| 17 | 7499502 | rs3933469 | A | G | 0.026 | 0.004 | 7.29962e-13 | 0.0003 | 51.469 | -0.016 | 0.014 | 0.266 | 0.26840 |
| 12 | 21331549 | rs4149056 | C | T | 0.027 | 0.004 | 8.90000e-10 | 0.0002 | 37.560 | -0.017 | 0.016 | 0.312 | 0.16100 |
| 4 | 109038654 | rs4245930 | A | G | -0.023 | 0.003 | 5.90065e-13 | 0.0003 | 51.882 | -0.016 | 0.013 | 0.240 | 0.61630 |
| 1 | 41453453 | rs4453027 | T | G | -0.019 | 0.003 | 5.60003e-09 | 0.0002 | 33.972 | -0.018 | 0.013 | 0.180 | 0.45230 |
| 15 | 79840557 | rs4464040 | T | C | -0.041 | 0.004 | 5.90065e-20 | 0.0004 | 83.637 | 0.047 | 0.024 | 0.050 | 0.13620 |
| 7 | 99332948 | rs45446698 | G | T | -0.312 | 0.008 | 1.00000e-200 | 0.0069 | 1395.268 | -0.040 | 0.030 | 0.182 | 0.04374 |
| 19 | 12509536 | rs4804181 | A | C | -0.034 | 0.004 | 2.70023e-19 | 0.0004 | 80.632 | 0.003 | 0.016 | 0.830 | 0.76540 |
| 1 | 66738623 | rs505237 | G | A | 0.018 | 0.003 | 2.19999e-08 | 0.0002 | 31.289 | -0.006 | 0.014 | 0.672 | 0.62620 |
| 7 | 156311364 | rs55911644 | A | T | -0.023 | 0.003 | 5.70033e-12 | 0.0002 | 47.446 | 0.002 | 0.013 | 0.868 | 0.38470 |
| 16 | 81590541 | rs58072681 | C | T | 0.090 | 0.006 | 7.59976e-49 | 0.0011 | 215.772 | -0.008 | 0.032 | 0.798 | 0.06561 |
| 5 | 35267743 | rs58282071 | G | A | -0.051 | 0.007 | 1.10002e-11 | 0.0002 | 46.088 | 0.043 | 0.024 | 0.072 | 0.04672 |
| 2 | 111934107 | rs590097 | G | T | 0.047 | 0.003 | 5.70033e-47 | 0.0010 | 207.157 | -0.024 | 0.014 | 0.085 | 0.62130 |
| 22 | 46633782 | rs6008259 | A | G | -0.029 | 0.004 | 2.29985e-12 | 0.0002 | 49.185 | 0.005 | 0.019 | 0.813 | 0.17990 |
| 9 | 34130435 | rs61237993 | A | G | 0.037 | 0.005 | 5.79963e-15 | 0.0003 | 60.967 | -0.026 | 0.019 | 0.189 | 0.13620 |
| 13 | 109924283 | rs61964100 | T | G | -0.054 | 0.009 | 1.60000e-09 | 0.0002 | 36.381 | 0.033 | 0.055 | 0.550 | 0.01988 |
| 13 | 22318506 | rs629042 | C | G | 0.046 | 0.003 | 2.49977e-45 | 0.0010 | 199.616 | -0.002 | 0.013 | 0.869 | 0.60240 |
| 16 | 30672097 | rs67128646 | C | A | 0.023 | 0.004 | 2.39999e-10 | 0.0002 | 40.115 | 0.005 | 0.015 | 0.723 | 0.27040 |
| 3 | 41107173 | rs6788984 | G | A | -0.041 | 0.005 | 1.59993e-19 | 0.0004 | 81.732 | 0.041 | 0.025 | 0.112 | 0.14510 |
| 11 | 64016284 | rs7111278 | A | G | -0.024 | 0.004 | 6.29999e-09 | 0.0002 | 33.732 | 0.005 | 0.017 | 0.770 | 0.17890 |
| 2 | 198492316 | rs72916919 | G | T | 0.017 | 0.003 | 3.50002e-08 | 0.0002 | 30.401 | -0.013 | 0.013 | 0.342 | 0.53680 |
| 1 | 22086167 | rs7521867 | C | T | -0.022 | 0.004 | 1.70000e-08 | 0.0002 | 31.797 | 0.028 | 0.016 | 0.076 | 0.74550 |
| 19 | 49517140 | rs75287599 | T | C | 0.037 | 0.006 | 4.20001e-10 | 0.0002 | 39.001 | -0.012 | 0.019 | 0.517 | 0.09642 |
| 2 | 43515427 | rs7575635 | T | C | -0.032 | 0.004 | 3.90032e-16 | 0.0003 | 66.300 | -0.016 | 0.017 | 0.339 | 0.22170 |
| 7 | 75607155 | rs76021834 | G | A | 0.020 | 0.003 | 1.60000e-08 | 0.0002 | 31.953 | 0.008 | 0.014 | 0.576 | 0.30620 |
| 3 | 10545125 | rs7618363 | G | C | -0.031 | 0.004 | 1.59993e-12 | 0.0002 | 49.929 | -0.033 | 0.021 | 0.107 | 0.12520 |
| 3 | 152084243 | rs7633673 | A | G | -0.021 | 0.003 | 2.90001e-11 | 0.0002 | 44.237 | -0.003 | 0.015 | 0.846 | 0.40660 |
| 4 | 1008212 | rs77822621 | T | C | 0.045 | 0.008 | 1.20000e-08 | 0.0002 | 32.451 | -0.041 | 0.063 | 0.513 | 0.03082 |
| 5 | 77987524 | rs784420 | G | A | 0.028 | 0.003 | 1.29987e-15 | 0.0003 | 63.976 | 0.015 | 0.015 | 0.302 | 0.28630 |
| 6 | 1373614 | rs78816356 | A | G | 0.033 | 0.005 | 3.09999e-10 | 0.0002 | 39.618 | 0.013 | 0.020 | 0.523 | 0.08151 |
| 16 | 4156809 | rs78865787 | G | A | -0.058 | 0.009 | 2.19989e-11 | 0.0002 | 44.774 | 0.010 | 0.028 | 0.715 | 0.02982 |
| 11 | 62913582 | rs79586456 | A | G | 0.074 | 0.006 | 1.80011e-31 | 0.0007 | 136.226 | -0.023 | 0.039 | 0.544 | 0.05567 |
| 16 | 81603771 | rs8044524 | A | G | -0.024 | 0.003 | 8.69961e-14 | 0.0003 | 55.630 | 0.011 | 0.014 | 0.420 | 0.59440 |
| 18 | 72036182 | rs8093038 | C | T | -0.044 | 0.005 | 4.40048e-17 | 0.0004 | 70.583 | 0.010 | 0.023 | 0.679 | 0.07753 |
| 19 | 10471462 | rs8111359 | T | C | -0.061 | 0.005 | 6.59933e-29 | 0.0006 | 124.490 | 0.045 | 0.018 | 0.013 | 0.09742 |
| 11 | 123437669 | rs850294 | T | C | 0.045 | 0.005 | 9.89920e-20 | 0.0004 | 82.624 | -0.013 | 0.026 | 0.625 | 0.08350 |
| 6 | 158195695 | rs928008 | G | A | 0.018 | 0.003 | 2.59998e-08 | 0.0002 | 30.953 | -0.002 | 0.013 | 0.867 | 0.50890 |
| 6 | 144318529 | rs9399469 | A | T | 0.026 | 0.003 | 4.00037e-15 | 0.0003 | 61.703 | 0.005 | 0.013 | 0.716 | 0.61930 |
| 10 | 104623578 | rs9527 | T | C | -0.033 | 0.004 | 2.70023e-19 | 0.0004 | 80.627 | 0.006 | 0.016 | 0.705 | 0.25150 |
| 13 | 73203811 | rs9599996 | G | T | -0.018 | 0.003 | 2.69998e-08 | 0.0002 | 30.920 | -0.012 | 0.014 | 0.385 | 0.37480 |
| 7 | 137799323 | rs9642092 | T | C | 0.043 | 0.004 | 1.10002e-28 | 0.0006 | 123.485 | 0.001 | 0.017 | 0.942 | 0.78330 |
| 5 | 55861786 | rs9686661 | T | C | -0.023 | 0.004 | 7.49998e-09 | 0.0002 | 33.405 | -0.007 | 0.019 | 0.736 | 0.18590 |
| 4 | 106071064 | rs974801 | G | A | 0.023 | 0.003 | 8.00018e-13 | 0.0003 | 51.284 | -0.005 | 0.014 | 0.741 | 0.40660 |

**Supplementary table 2.19. Detailed information of instrumental SNPs for Bioavailable Testosterone in the analysis of PPD.**

| **Chr** | **Position** | **SNP** | **EA** | **OA** | **beta.exposure** | **se.exposure** | **pval.exposure** | **R2** | **F_val** | **beta.outcome** | **se.outcome** | **pval.outcome** | **Eaf.outcome** |
| --- | --- | --- | --- | --- | --- | --- | --- | --- | --- | --- | --- | --- | --- |
| 2 | 112375425 | rs10168169 | T | C | 0.032 | 0.004 | 1.80011e-13 | 0.0003 | 54.201 | 0.009 | 0.015 | 0.550 | 0.80520 |
| 17 | 7560835 | rs1050541 | G | T | -0.051 | 0.003 | 5.10035e-51 | 0.0013 | 225.738 | -0.014 | 0.013 | 0.299 | 0.52580 |
| 12 | 103537266 | rs10778215 | A | T | 0.032 | 0.003 | 1.29987e-21 | 0.0005 | 91.250 | -0.015 | 0.014 | 0.282 | 0.51190 |
| 9 | 114828332 | rs10817260 | T | C | -0.030 | 0.004 | 1.20005e-12 | 0.0003 | 50.541 | 0.000 | 0.015 | 0.989 | 0.19880 |
| 1 | 235258183 | rs10925196 | G | A | -0.025 | 0.004 | 4.70002e-08 | 0.0002 | 29.851 | -0.011 | 0.020 | 0.568 | 0.14910 |
| 9 | 102182414 | rs10988819 | T | A | -0.021 | 0.004 | 2.90001e-08 | 0.0002 | 30.780 | -0.010 | 0.015 | 0.537 | 0.21970 |
| 11 | 30226356 | rs11031005 | C | T | 0.031 | 0.005 | 3.19963e-11 | 0.0002 | 44.080 | 0.004 | 0.018 | 0.841 | 0.14020 |
| 5 | 76488311 | rs1119209 | T | C | -0.021 | 0.003 | 1.29999e-09 | 0.0002 | 36.776 | -0.021 | 0.013 | 0.117 | 0.36480 |
| 6 | 119611059 | rs111984076 | G | C | -0.063 | 0.011 | 1.79999e-08 | 0.0002 | 31.671 | -0.021 | 0.047 | 0.658 | 0.01889 |
| 11 | 72947934 | rs11235688 | A | G | -0.022 | 0.003 | 1.00000e-10 | 0.0002 | 41.782 | -0.004 | 0.013 | 0.768 | 0.41150 |
| 14 | 94838142 | rs112635299 | T | G | -0.102 | 0.012 | 2.90001e-18 | 0.0004 | 75.931 | -0.004 | 0.048 | 0.941 | 0.01690 |
| 8 | 143954290 | rs1134095 | A | G | -0.026 | 0.003 | 2.49977e-15 | 0.0003 | 62.624 | -0.005 | 0.014 | 0.710 | 0.59340 |
| 1 | 27021913 | rs114165349 | C | G | 0.114 | 0.011 | 1.99986e-25 | 0.0006 | 108.545 | -0.015 | 0.035 | 0.675 | 0.02187 |
| 14 | 24871926 | rs11621792 | T | C | 0.030 | 0.003 | 7.00003e-19 | 0.0004 | 78.762 | 0.050 | 0.014 | 0.000 | 0.47020 |
| 2 | 62538681 | rs11683263 | C | T | -0.020 | 0.003 | 8.90000e-10 | 0.0002 | 37.552 | -0.011 | 0.014 | 0.427 | 0.56660 |
| 10 | 61467182 | rs1171617 | T | G | 0.040 | 0.004 | 3.19963e-24 | 0.0006 | 103.085 | -0.020 | 0.018 | 0.261 | 0.75650 |
| 8 | 105978368 | rs11774829 | A | T | 0.040 | 0.006 | 2.70023e-13 | 0.0003 | 53.437 | 0.015 | 0.018 | 0.408 | 0.09940 |
| 15 | 75925592 | rs11856606 | A | G | -0.036 | 0.005 | 2.60016e-13 | 0.0003 | 53.464 | -0.007 | 0.023 | 0.741 | 0.13320 |
| 19 | 46193351 | rs11879227 | G | A | -0.025 | 0.004 | 2.30001e-09 | 0.0002 | 35.734 | 0.000 | 0.016 | 0.995 | 0.19880 |
| 2 | 24990082 | rs11892043 | G | A | -0.025 | 0.004 | 2.09991e-11 | 0.0002 | 44.856 | 0.008 | 0.014 | 0.568 | 0.28030 |
| 6 | 43352980 | rs1214759 | G | A | 0.025 | 0.004 | 1.50003e-12 | 0.0003 | 50.081 | -0.028 | 0.014 | 0.054 | 0.62920 |
| 5 | 35239886 | rs12189146 | A | G | -0.041 | 0.008 | 3.89996e-08 | 0.0002 | 30.180 | 0.044 | 0.023 | 0.054 | 0.05467 |
| 2 | 178167261 | rs12474344 | G | C | -0.021 | 0.003 | 2.50000e-10 | 0.0002 | 40.050 | 0.008 | 0.014 | 0.536 | 0.57950 |
| 1 | 168234645 | rs12564492 | A | G | 0.022 | 0.004 | 1.50000e-09 | 0.0002 | 36.485 | -0.012 | 0.014 | 0.374 | 0.68790 |
| 5 | 124205385 | rs12658172 | C | G | -0.042 | 0.005 | 3.40017e-20 | 0.0005 | 84.770 | 0.020 | 0.018 | 0.274 | 0.16500 |
| 9 | 16252807 | rs12683780 | C | A | -0.024 | 0.004 | 2.70023e-11 | 0.0003 | 44.354 | 0.010 | 0.014 | 0.467 | 0.34890 |
| 2 | 12567937 | rs13022088 | G | A | 0.024 | 0.004 | 1.09999e-10 | 0.0002 | 41.666 | 0.016 | 0.014 | 0.233 | 0.29030 |
| 2 | 43508205 | rs13030651 | A | G | -0.021 | 0.003 | 4.39997e-10 | 0.0002 | 38.935 | -0.012 | 0.014 | 0.358 | 0.59940 |
| 4 | 88203828 | rs13150068 | G | A | 0.020 | 0.003 | 1.20000e-09 | 0.0002 | 36.905 | -0.015 | 0.014 | 0.291 | 0.40850 |
| 5 | 176782218 | rs13153019 | C | T | 0.021 | 0.004 | 3.50002e-08 | 0.0002 | 30.426 | 0.034 | 0.015 | 0.023 | 0.26440 |
| 7 | 73030175 | rs13229619 | A | G | 0.038 | 0.005 | 8.60003e-15 | 0.0003 | 60.184 | -0.029 | 0.020 | 0.151 | 0.11830 |
| 2 | 12312061 | rs143851281 | C | G | -0.037 | 0.006 | 9.90011e-09 | 0.0002 | 32.853 | 0.022 | 0.027 | 0.431 | 0.05169 |
| 2 | 227073854 | rs1515098 | T | C | 0.022 | 0.004 | 3.40001e-10 | 0.0002 | 39.414 | -0.009 | 0.014 | 0.532 | 0.66900 |
| 5 | 77974427 | rs168189 | T | C | 0.025 | 0.004 | 5.70033e-12 | 0.0003 | 47.422 | 0.012 | 0.015 | 0.420 | 0.28130 |
| 19 | 35552079 | rs1688044 | G | C | -0.039 | 0.007 | 2.30001e-09 | 0.0002 | 35.670 | 0.026 | 0.028 | 0.340 | 0.93840 |
| 5 | 128015370 | rs17677724 | T | C | -0.024 | 0.004 | 4.30002e-08 | 0.0002 | 30.028 | 0.004 | 0.020 | 0.858 | 0.16600 |
| 14 | 98546911 | rs17698580 | C | T | 0.025 | 0.004 | 5.30029e-11 | 0.0002 | 43.067 | 0.045 | 0.016 | 0.004 | 0.26040 |
| 15 | 40655873 | rs1898882 | C | G | 0.022 | 0.003 | 1.90020e-11 | 0.0002 | 45.023 | 0.025 | 0.013 | 0.064 | 0.41850 |
| 17 | 7261866 | rs192287715 | A | G | 0.047 | 0.006 | 2.09991e-14 | 0.0003 | 58.419 | -0.032 | 0.023 | 0.159 | 0.07256 |
| 1 | 7909373 | rs1989147 | T | C | -0.030 | 0.004 | 1.80011e-12 | 0.0003 | 49.668 | -0.026 | 0.016 | 0.100 | 0.18990 |
| 1 | 179293511 | rs2152318 | T | C | 0.043 | 0.004 | 5.50047e-29 | 0.0007 | 124.846 | 0.015 | 0.015 | 0.309 | 0.24350 |
| 2 | 64909166 | rs2160348 | C | T | 0.038 | 0.004 | 5.00035e-24 | 0.0006 | 102.210 | -0.029 | 0.016 | 0.069 | 0.73360 |
| 10 | 122873944 | rs2243612 | C | T | 0.036 | 0.005 | 8.90020e-13 | 0.0003 | 51.074 | 0.000 | 0.018 | 0.995 | 0.11630 |
| 3 | 195942487 | rs2342307 | G | A | -0.022 | 0.003 | 5.60015e-11 | 0.0002 | 42.947 | -0.015 | 0.014 | 0.266 | 0.63920 |
| 6 | 52650183 | rs2608666 | A | C | 0.019 | 0.003 | 1.40001e-08 | 0.0002 | 32.145 | 0.022 | 0.013 | 0.097 | 0.56260 |
| 8 | 81424801 | rs272607 | G | T | -0.026 | 0.004 | 5.30029e-13 | 0.0003 | 52.082 | 0.047 | 0.015 | 0.002 | 0.33900 |
| 10 | 5048014 | rs2801885 | G | A | -0.023 | 0.004 | 1.70000e-09 | 0.0002 | 36.264 | 0.030 | 0.015 | 0.048 | 0.26540 |
| 15 | 40364263 | rs28479712 | G | A | 0.035 | 0.003 | 1.10002e-24 | 0.0006 | 105.269 | 0.012 | 0.014 | 0.373 | 0.32600 |
| 12 | 122134415 | rs2954111 | C | T | 0.020 | 0.003 | 3.59998e-09 | 0.0002 | 34.830 | 0.004 | 0.014 | 0.798 | 0.30820 |
| 11 | 68097049 | rs312023 | A | G | -0.022 | 0.003 | 4.30031e-11 | 0.0002 | 43.494 | -0.006 | 0.014 | 0.661 | 0.53880 |
| 4 | 69335293 | rs34072207 | G | T | -0.028 | 0.004 | 8.99912e-13 | 0.0003 | 51.050 | 0.016 | 0.016 | 0.319 | 0.17500 |
| 17 | 66879927 | rs34931250 | T | C | -0.052 | 0.007 | 1.00000e-13 | 0.0003 | 55.350 | -0.016 | 0.027 | 0.548 | 0.04970 |
| 1 | 113054659 | rs351370 | T | C | 0.021 | 0.003 | 5.69994e-10 | 0.0002 | 38.414 | 0.009 | 0.014 | 0.516 | 0.59940 |
| 8 | 81655099 | rs35339118 | T | C | -0.020 | 0.003 | 9.49992e-09 | 0.0002 | 32.949 | -0.007 | 0.014 | 0.589 | 0.41350 |
| 18 | 3813758 | rs3786432 | T | C | 0.025 | 0.004 | 2.09991e-11 | 0.0002 | 44.882 | -0.014 | 0.014 | 0.322 | 0.71770 |
| 17 | 45750596 | rs3809868 | A | G | -0.033 | 0.003 | 1.29987e-23 | 0.0005 | 100.316 | 0.009 | 0.013 | 0.498 | 0.53780 |
| 16 | 4136418 | rs387212 | C | T | -0.021 | 0.004 | 3.79997e-09 | 0.0002 | 34.701 | 0.019 | 0.014 | 0.168 | 0.31210 |
| 5 | 55804552 | rs40270 | C | A | 0.025 | 0.004 | 3.40001e-10 | 0.0002 | 39.402 | -0.013 | 0.015 | 0.378 | 0.73860 |
| 7 | 75612803 | rs41301394 | T | C | 0.024 | 0.004 | 6.20012e-11 | 0.0002 | 42.743 | 0.014 | 0.014 | 0.304 | 0.29320 |
| 12 | 21331549 | rs4149056 | C | T | 0.053 | 0.005 | 1.29987e-30 | 0.0007 | 132.217 | -0.017 | 0.016 | 0.312 | 0.16100 |
| 6 | 119128633 | rs415441 | T | C | 0.045 | 0.004 | 4.00037e-30 | 0.0007 | 130.039 | -0.016 | 0.018 | 0.371 | 0.78830 |
| 4 | 109038654 | rs4245930 | A | G | -0.019 | 0.003 | 1.29999e-08 | 0.0002 | 32.317 | -0.016 | 0.013 | 0.240 | 0.61630 |
| 3 | 156852141 | rs4368453 | T | C | 0.023 | 0.004 | 2.00000e-10 | 0.0002 | 40.419 | 0.021 | 0.016 | 0.188 | 0.30220 |
| 15 | 79840557 | rs4464040 | T | C | -0.034 | 0.005 | 2.90001e-13 | 0.0003 | 53.287 | 0.047 | 0.024 | 0.050 | 0.13620 |
| 7 | 97913196 | rs4506131 | C | G | -0.034 | 0.004 | 2.99985e-20 | 0.0005 | 84.975 | -0.018 | 0.016 | 0.266 | 0.71770 |
| 7 | 99332948 | rs45446698 | G | T | -0.219 | 0.009 | 2.79898e-138 | 0.0034 | 626.597 | -0.040 | 0.030 | 0.182 | 0.04374 |
| 10 | 93646268 | rs4565819 | A | G | -0.027 | 0.004 | 3.69999e-10 | 0.0002 | 39.282 | 0.028 | 0.018 | 0.122 | 0.80120 |
| 4 | 3447156 | rs4690098 | T | C | 0.030 | 0.004 | 1.29987e-14 | 0.0003 | 59.361 | 0.014 | 0.015 | 0.362 | 0.23460 |
| 6 | 25842203 | rs4712976 | T | C | -0.025 | 0.004 | 3.10027e-11 | 0.0002 | 44.104 | 0.002 | 0.014 | 0.875 | 0.26440 |
| 12 | 57766392 | rs4760254 | C | G | -0.028 | 0.004 | 1.99986e-13 | 0.0003 | 53.999 | 0.007 | 0.016 | 0.672 | 0.19090 |
| 12 | 102806305 | rs4764883 | T | C | 0.020 | 0.004 | 4.90004e-08 | 0.0002 | 29.746 | -0.001 | 0.014 | 0.914 | 0.72560 |
| 19 | 18207397 | rs55714539 | C | A | 0.021 | 0.003 | 1.50000e-09 | 0.0002 | 36.561 | -0.028 | 0.014 | 0.038 | 0.34790 |
| 12 | 2908330 | rs56196860 | A | C | -0.064 | 0.010 | 1.50003e-11 | 0.0002 | 45.567 | -0.078 | 0.060 | 0.194 | 0.02883 |
| 15 | 96714816 | rs56332871 | A | C | -0.027 | 0.004 | 2.39994e-13 | 0.0003 | 53.681 | 0.008 | 0.015 | 0.601 | 0.30220 |
| 3 | 10534247 | rs573833 | C | T | -0.018 | 0.003 | 4.30002e-08 | 0.0002 | 29.997 | -0.018 | 0.014 | 0.195 | 0.51990 |
| 16 | 81590541 | rs58072681 | C | T | 0.077 | 0.006 | 1.10002e-33 | 0.0008 | 146.333 | -0.008 | 0.032 | 0.798 | 0.06561 |
| 2 | 111934107 | rs590097 | G | T | 0.045 | 0.003 | 2.39994e-38 | 0.0009 | 167.671 | -0.024 | 0.014 | 0.085 | 0.62130 |
| 22 | 46633782 | rs6008259 | A | G | -0.028 | 0.004 | 4.00037e-11 | 0.0002 | 43.601 | 0.005 | 0.019 | 0.813 | 0.17990 |
| 20 | 48909667 | rs6020423 | T | C | -0.030 | 0.004 | 6.79986e-15 | 0.0003 | 60.655 | 0.000 | 0.014 | 0.973 | 0.25250 |
| 9 | 34130435 | rs61237993 | A | G | 0.035 | 0.005 | 1.39991e-12 | 0.0003 | 50.138 | -0.026 | 0.019 | 0.189 | 0.13620 |
| 10 | 5063382 | rs61856128 | A | C | -0.043 | 0.004 | 1.00000e-31 | 0.0008 | 137.308 | -0.009 | 0.015 | 0.540 | 0.29620 |
| 21 | 40683740 | rs62223042 | G | A | 0.020 | 0.003 | 4.60002e-09 | 0.0002 | 34.341 | 0.000 | 0.013 | 0.995 | 0.41050 |
| 3 | 48260304 | rs62263023 | A | T | 0.039 | 0.006 | 1.10002e-11 | 0.0003 | 46.112 | 0.046 | 0.023 | 0.043 | 0.07952 |
| 13 | 22318506 | rs629042 | C | G | 0.041 | 0.003 | 2.19989e-33 | 0.0008 | 144.940 | -0.002 | 0.013 | 0.869 | 0.60240 |
| 1 | 92979354 | rs6603979 | G | A | -0.030 | 0.004 | 1.99986e-13 | 0.0003 | 54.009 | 0.008 | 0.016 | 0.630 | 0.80720 |
| 1 | 218520995 | rs6658835 | G | A | -0.023 | 0.004 | 1.09999e-09 | 0.0002 | 37.139 | -0.018 | 0.014 | 0.225 | 0.26940 |
| 3 | 41107173 | rs6788984 | G | A | -0.028 | 0.005 | 2.80001e-09 | 0.0002 | 35.337 | 0.041 | 0.025 | 0.112 | 0.14510 |
| 3 | 24520283 | rs6792725 | G | A | -0.023 | 0.004 | 2.10000e-10 | 0.0002 | 40.349 | -0.015 | 0.013 | 0.253 | 0.67100 |
| 5 | 142792990 | rs6865292 | C | T | 0.022 | 0.004 | 8.30004e-09 | 0.0002 | 33.201 | -0.003 | 0.015 | 0.837 | 0.25750 |
| 3 | 135932359 | rs687339 | T | C | 0.045 | 0.004 | 5.90065e-30 | 0.0007 | 129.276 | -0.037 | 0.019 | 0.047 | 0.77040 |
| 7 | 98898149 | rs6957987 | G | A | -0.032 | 0.005 | 3.69999e-09 | 0.0002 | 34.771 | 0.017 | 0.018 | 0.344 | 0.11430 |
| 9 | 86368660 | rs7047907 | A | G | 0.030 | 0.004 | 1.59993e-15 | 0.0003 | 63.567 | -0.020 | 0.015 | 0.196 | 0.71970 |
| 12 | 121415293 | rs7139079 | A | G | -0.026 | 0.003 | 5.30029e-15 | 0.0003 | 61.133 | -0.008 | 0.013 | 0.540 | 0.56460 |
| 17 | 7537792 | rs727428 | C | T | -0.106 | 0.003 | 1.00000e-200 | 0.0056 | 1033.980 | -0.012 | 0.014 | 0.361 | 0.57260 |
| 2 | 20420078 | rs72785204 | A | G | 0.027 | 0.003 | 6.09958e-15 | 0.0003 | 60.865 | -0.010 | 0.015 | 0.513 | 0.36580 |
| 17 | 47455752 | rs73324348 | A | G | -0.074 | 0.006 | 2.99985e-37 | 0.0009 | 162.622 | -0.035 | 0.036 | 0.326 | 0.06461 |
| 11 | 66119563 | rs7395670 | T | C | -0.020 | 0.003 | 3.40001e-09 | 0.0002 | 34.929 | -0.006 | 0.013 | 0.633 | 0.39660 |
| 8 | 23403387 | rs7464081 | C | T | 0.024 | 0.004 | 3.50002e-10 | 0.0002 | 39.372 | -0.002 | 0.015 | 0.874 | 0.25250 |
| 3 | 152084243 | rs7633673 | A | G | -0.020 | 0.003 | 5.30005e-09 | 0.0002 | 34.080 | -0.003 | 0.015 | 0.846 | 0.40660 |
| 11 | 62913772 | rs80237154 | G | C | 0.064 | 0.007 | 1.10002e-21 | 0.0005 | 91.442 | -0.024 | 0.039 | 0.543 | 0.05666 |
| 15 | 53152770 | rs8031605 | C | G | 0.036 | 0.005 | 2.60016e-11 | 0.0002 | 44.450 | 0.005 | 0.028 | 0.863 | 0.08250 |
| 16 | 81603771 | rs8044524 | A | G | -0.020 | 0.003 | 6.10000e-09 | 0.0002 | 33.791 | 0.011 | 0.014 | 0.420 | 0.59440 |
| 18 | 72036182 | rs8093038 | C | T | -0.035 | 0.006 | 2.00000e-10 | 0.0002 | 40.424 | 0.010 | 0.023 | 0.679 | 0.07753 |
| 19 | 10471462 | rs8111359 | T | C | -0.055 | 0.006 | 6.09958e-22 | 0.0005 | 92.694 | 0.045 | 0.018 | 0.013 | 0.09742 |
| 11 | 123438118 | rs850293 | C | A | 0.042 | 0.005 | 1.39991e-15 | 0.0003 | 63.745 | -0.023 | 0.029 | 0.423 | 0.08052 |
| 6 | 144318529 | rs9399469 | A | T | 0.027 | 0.003 | 2.49977e-15 | 0.0003 | 62.603 | 0.005 | 0.013 | 0.716 | 0.61930 |
| 10 | 104623578 | rs9527 | T | C | -0.031 | 0.004 | 1.90020e-15 | 0.0003 | 63.173 | 0.006 | 0.016 | 0.705 | 0.25150 |
| 13 | 73136378 | rs9543004 | C | T | 0.020 | 0.003 | 3.29997e-09 | 0.0002 | 34.975 | -0.014 | 0.014 | 0.287 | 0.35980 |
| 7 | 137799323 | rs9642092 | T | C | 0.033 | 0.004 | 5.50047e-16 | 0.0004 | 65.602 | 0.001 | 0.017 | 0.942 | 0.78330 |
| 17 | 7326404 | rs9910990 | C | T | 0.047 | 0.006 | 5.40008e-14 | 0.0003 | 56.575 | -0.002 | 0.017 | 0.908 | 0.08052 |
| 16 | 29001460 | rs9929088 | C | A | -0.020 | 0.004 | 1.09999e-08 | 0.0002 | 32.570 | 0.048 | 0.014 | 0.001 | 0.73260 |
| 8 | 9183358 | rs9987289 | G | A | -0.038 | 0.006 | 2.19989e-11 | 0.0002 | 44.805 | 0.021 | 0.019 | 0.270 | 0.92540 |

**Supplementary table 2.20. Detailed information of instrumental SNPs for Sex hormone binding globulin in the analysis of PPD.**

| **Chr** | **Position** | **SNP** | **EA** | **OA** | **beta.exposure** | **se.exposure** | **pval.exposure** | **R2** | **F_val** | **beta.outcome** | **se.outcome** | **pval.outcome** | **Eaf.outcome** |
| --- | --- | --- | --- | --- | --- | --- | --- | --- | --- | --- | --- | --- | --- |
| 2 | 58884018 | rs1014291 | T | G | 0.018 | 0.003 | 2.50000e-09 | 0.0002 | 35.563 | 0.002 | 0.013 | 0.886 | 0.44140 |
| 17 | 79481772 | rs10153315 | C | T | -0.019 | 0.003 | 1.29999e-10 | 0.0002 | 41.261 | -0.004 | 0.013 | 0.762 | 0.42350 |
| 2 | 165508389 | rs10184004 | T | C | 0.033 | 0.003 | 4.00037e-28 | 0.0005 | 120.896 | 0.007 | 0.014 | 0.626 | 0.44330 |
| 7 | 99208899 | rs10238028 | G | A | 0.040 | 0.006 | 1.80011e-11 | 0.0002 | 45.177 | -0.015 | 0.026 | 0.552 | 0.06064 |
| 2 | 211540507 | rs1047891 | A | C | 0.026 | 0.003 | 5.90065e-16 | 0.0003 | 65.482 | 0.004 | 0.014 | 0.752 | 0.30120 |
| 8 | 59398461 | rs10504255 | A | G | 0.020 | 0.003 | 4.70002e-10 | 0.0002 | 38.785 | 0.018 | 0.014 | 0.190 | 0.63520 |
| 1 | 193119940 | rs10801178 | C | G | 0.019 | 0.003 | 6.19998e-09 | 0.0001 | 33.783 | 0.013 | 0.014 | 0.349 | 0.64510 |
| 10 | 65124098 | rs10822163 | G | C | 0.092 | 0.003 | 1.00000e-200 | 0.0042 | 966.064 | -0.009 | 0.013 | 0.513 | 0.48710 |
| 10 | 81094125 | rs10824744 | G | A | -0.028 | 0.004 | 5.00035e-14 | 0.0002 | 56.732 | -0.006 | 0.017 | 0.712 | 0.19880 |
| 12 | 20587192 | rs10841521 | G | A | 0.019 | 0.003 | 4.00000e-08 | 0.0001 | 30.164 | 0.015 | 0.015 | 0.307 | 0.26640 |
| 11 | 82779736 | rs10898075 | T | C | -0.025 | 0.004 | 2.30001e-10 | 0.0002 | 40.180 | 0.011 | 0.021 | 0.582 | 0.17300 |
| 1 | 23691745 | rs10917373 | T | C | 0.022 | 0.003 | 3.50026e-11 | 0.0002 | 43.848 | -0.001 | 0.016 | 0.940 | 0.73060 |
| 11 | 16246700 | rs11023881 | A | T | -0.023 | 0.003 | 2.60016e-14 | 0.0003 | 58.030 | 0.012 | 0.014 | 0.397 | 0.41850 |
| 12 | 6853766 | rs11064358 | A | G | -0.026 | 0.004 | 1.20000e-09 | 0.0002 | 36.990 | -0.020 | 0.022 | 0.371 | 0.10440 |
| 12 | 100874901 | rs11110390 | T | C | -0.023 | 0.003 | 9.30037e-14 | 0.0002 | 55.500 | -0.020 | 0.013 | 0.129 | 0.37770 |
| 12 | 57645789 | rs11172134 | A | T | 0.031 | 0.004 | 2.49977e-17 | 0.0003 | 71.711 | 0.010 | 0.016 | 0.537 | 0.15900 |
| 9 | 131591894 | rs112086410 | C | G | -0.030 | 0.004 | 1.80011e-11 | 0.0002 | 45.165 | -0.017 | 0.021 | 0.419 | 0.13420 |
| 1 | 27021913 | rs114165349 | C | G | -0.147 | 0.010 | 4.10015e-50 | 0.0010 | 221.562 | -0.015 | 0.035 | 0.675 | 0.02187 |
| 7 | 150498245 | rs114949263 | C | T | 0.030 | 0.005 | 1.09999e-10 | 0.0002 | 41.620 | 0.045 | 0.025 | 0.068 | 0.10240 |
| 7 | 129663496 | rs11556924 | T | C | 0.023 | 0.003 | 2.49977e-14 | 0.0003 | 58.095 | 0.007 | 0.014 | 0.642 | 0.37670 |
| 14 | 24871926 | rs11621792 | T | C | -0.048 | 0.003 | 6.40030e-58 | 0.0011 | 257.371 | 0.050 | 0.014 | 0.000 | 0.47020 |
| 15 | 40387728 | rs11637595 | T | C | -0.019 | 0.003 | 2.50000e-08 | 0.0001 | 31.024 | -0.009 | 0.016 | 0.603 | 0.26340 |
| 16 | 15172118 | rs11644601 | C | T | 0.024 | 0.003 | 1.10002e-13 | 0.0002 | 55.172 | -0.008 | 0.015 | 0.605 | 0.29030 |
| 12 | 4328521 | rs117233107 | A | G | 0.111 | 0.013 | 7.10068e-18 | 0.0004 | 74.178 | 0.003 | 0.057 | 0.959 | 0.01491 |
| 17 | 7651906 | rs117387630 | T | C | -0.307 | 0.011 | 1.00000e-187 | 0.0040 | 853.901 | 0.095 | 0.035 | 0.007 | 0.02286 |
| 1 | 51038327 | rs12121301 | A | T | 0.019 | 0.003 | 2.80001e-10 | 0.0002 | 39.840 | -0.025 | 0.015 | 0.097 | 0.42540 |
| 18 | 55081839 | rs1217569 | A | G | -0.024 | 0.004 | 5.00035e-11 | 0.0002 | 43.163 | 0.007 | 0.017 | 0.699 | 0.19480 |
| 1 | 214321081 | rs1223791 | A | G | -0.029 | 0.004 | 4.30031e-13 | 0.0002 | 52.501 | -0.010 | 0.018 | 0.587 | 0.83800 |
| 7 | 81568750 | rs1229498 | G | T | -0.022 | 0.003 | 1.50000e-10 | 0.0002 | 41.052 | -0.007 | 0.015 | 0.650 | 0.73060 |
| 8 | 72436958 | rs12334548 | C | G | -0.036 | 0.005 | 5.79963e-11 | 0.0002 | 42.885 | 0.001 | 0.035 | 0.987 | 0.05964 |
| 13 | 51107757 | rs1239945 | A | G | -0.016 | 0.003 | 4.49997e-08 | 0.0001 | 29.931 | 0.020 | 0.014 | 0.136 | 0.53480 |
| 16 | 30027694 | rs12444108 | A | G | -0.018 | 0.003 | 4.00000e-09 | 0.0002 | 34.630 | -0.033 | 0.014 | 0.017 | 0.38470 |
| 11 | 95311260 | rs12575636 | G | T | -0.028 | 0.004 | 1.29987e-13 | 0.0002 | 54.881 | 0.000 | 0.017 | 0.995 | 0.21170 |
| 2 | 27730940 | rs1260326 | C | T | 0.064 | 0.003 | 3.10027e-99 | 0.0019 | 447.065 | 0.004 | 0.014 | 0.762 | 0.58950 |
| 2 | 111897506 | rs12613243 | C | T | -0.036 | 0.006 | 4.39997e-09 | 0.0002 | 34.430 | 0.004 | 0.033 | 0.904 | 0.06759 |
| 11 | 69284560 | rs12805041 | T | C | 0.033 | 0.004 | 9.09913e-21 | 0.0004 | 87.342 | -0.012 | 0.016 | 0.459 | 0.21070 |
| 15 | 51054195 | rs12914895 | G | C | 0.017 | 0.003 | 2.10000e-08 | 0.0001 | 31.443 | 0.028 | 0.013 | 0.035 | 0.49400 |
| 6 | 151991200 | rs1293953 | C | G | 0.021 | 0.003 | 3.19963e-11 | 0.0002 | 44.033 | -0.029 | 0.014 | 0.032 | 0.31810 |
| 17 | 29680526 | rs12943365 | G | C | 0.022 | 0.003 | 7.70016e-13 | 0.0002 | 51.369 | -0.030 | 0.014 | 0.027 | 0.62720 |
| 17 | 7318935 | rs12947017 | T | C | 0.129 | 0.003 | 1.00000e-200 | 0.0080 | 1793.482 | -0.004 | 0.014 | 0.773 | 0.38270 |
| 3 | 49779906 | rs13086465 | T | C | -0.028 | 0.003 | 1.59993e-18 | 0.0003 | 77.167 | 0.039 | 0.015 | 0.010 | 0.30820 |
| 4 | 3443931 | rs13108218 | G | A | -0.047 | 0.003 | 7.50067e-53 | 0.0010 | 234.141 | -0.029 | 0.014 | 0.041 | 0.61030 |
| 4 | 88203828 | rs13150068 | G | A | -0.033 | 0.003 | 2.29985e-28 | 0.0005 | 122.023 | -0.015 | 0.014 | 0.291 | 0.40850 |
| 5 | 55868097 | rs13179413 | T | C | -0.023 | 0.003 | 8.60003e-12 | 0.0002 | 46.633 | -0.014 | 0.015 | 0.350 | 0.28530 |
| 8 | 145656806 | rs13273326 | G | A | -0.019 | 0.003 | 8.80035e-11 | 0.0002 | 42.076 | -0.012 | 0.013 | 0.355 | 0.53080 |
| 9 | 4305064 | rs1330307 | C | A | -0.017 | 0.003 | 4.60002e-09 | 0.0002 | 34.352 | 0.021 | 0.013 | 0.117 | 0.52390 |
| 14 | 74250126 | rs13379043 | C | T | 0.020 | 0.003 | 2.10000e-09 | 0.0002 | 35.867 | 0.009 | 0.017 | 0.584 | 0.29820 |
| 2 | 227026864 | rs13423088 | A | T | 0.029 | 0.003 | 4.70002e-20 | 0.0004 | 84.091 | 0.007 | 0.014 | 0.603 | 0.32800 |
| 2 | 191564893 | rs13427019 | A | T | -0.020 | 0.003 | 2.00000e-08 | 0.0001 | 31.468 | 0.007 | 0.014 | 0.600 | 0.25650 |
| 19 | 35553756 | rs1350292 | T | C | 0.064 | 0.006 | 2.70023e-27 | 0.0005 | 117.125 | 0.026 | 0.028 | 0.343 | 0.93840 |
| 17 | 7092533 | rs140215187 | G | A | -0.060 | 0.010 | 1.50000e-09 | 0.0002 | 36.483 | -0.009 | 0.039 | 0.807 | 0.03380 |
| 17 | 7502034 | rs142331290 | G | A | -0.173 | 0.012 | 7.50067e-49 | 0.0009 | 215.785 | 0.096 | 0.064 | 0.132 | 0.01292 |
| 8 | 81399180 | rs150539196 | G | A | 0.094 | 0.008 | 2.80027e-30 | 0.0006 | 130.773 | 0.025 | 0.043 | 0.567 | 0.03579 |
| 7 | 130585553 | rs157935 | G | T | 0.018 | 0.003 | 2.80001e-08 | 0.0001 | 30.858 | 0.004 | 0.014 | 0.785 | 0.33200 |
| 22 | 19949013 | rs165722 | T | C | -0.017 | 0.003 | 7.10003e-09 | 0.0001 | 33.505 | -0.027 | 0.013 | 0.046 | 0.50100 |
| 1 | 171079005 | rs16864042 | T | G | -0.017 | 0.003 | 6.90001e-09 | 0.0001 | 33.566 | 0.021 | 0.013 | 0.118 | 0.36680 |
| 7 | 97974851 | rs1688606 | A | G | 0.074 | 0.004 | 2.39994e-84 | 0.0017 | 378.649 | -0.018 | 0.019 | 0.327 | 0.81710 |
| 1 | 107607810 | rs1730864 | T | C | -0.043 | 0.003 | 6.09958e-42 | 0.0008 | 184.117 | -0.004 | 0.015 | 0.793 | 0.67790 |
| 11 | 61552680 | rs174537 | T | G | -0.028 | 0.003 | 6.29941e-19 | 0.0003 | 78.971 | 0.010 | 0.013 | 0.455 | 0.34890 |
| 4 | 171180561 | rs17547712 | A | G | 0.032 | 0.006 | 6.10000e-09 | 0.0001 | 33.811 | 0.024 | 0.037 | 0.510 | 0.06262 |
| 7 | 100493711 | rs17884589 | A | G | 0.036 | 0.004 | 8.99912e-21 | 0.0004 | 87.365 | 0.007 | 0.016 | 0.660 | 0.18390 |
| 4 | 77138658 | rs1866975 | T | C | -0.022 | 0.003 | 2.09991e-13 | 0.0002 | 53.899 | 0.013 | 0.013 | 0.333 | 0.39170 |
| 12 | 21352315 | rs1871395 | G | A | -0.053 | 0.004 | 2.60016e-38 | 0.0007 | 167.494 | -0.016 | 0.015 | 0.291 | 0.17790 |
| 12 | 21571656 | rs1989390 | A | C | -0.024 | 0.004 | 4.09996e-09 | 0.0002 | 34.580 | 0.004 | 0.017 | 0.803 | 0.80420 |
| 19 | 17346854 | rs202200760 | C | G | 0.155 | 0.008 | 6.70039e-78 | 0.0018 | 349.094 | 0.029 | 0.030 | 0.329 | 0.04573 |
| 10 | 94839642 | rs2068888 | A | G | 0.024 | 0.003 | 1.29987e-15 | 0.0003 | 63.884 | -0.014 | 0.013 | 0.299 | 0.48610 |
| 4 | 148988159 | rs2076947 | C | T | -0.026 | 0.004 | 5.50047e-12 | 0.0002 | 47.505 | -0.007 | 0.016 | 0.675 | 0.77730 |
| 4 | 69328988 | rs2123885 | G | T | 0.021 | 0.004 | 2.50000e-09 | 0.0002 | 35.505 | 0.015 | 0.016 | 0.330 | 0.17590 |
| 12 | 124484643 | rs2130382 | G | C | 0.021 | 0.003 | 1.00000e-10 | 0.0002 | 41.787 | 0.026 | 0.015 | 0.071 | 0.34590 |
| 2 | 64909166 | rs2160348 | C | T | -0.056 | 0.003 | 1.59993e-62 | 0.0012 | 278.506 | -0.029 | 0.016 | 0.069 | 0.73360 |
| 8 | 116999486 | rs2223054 | C | T | -0.022 | 0.003 | 3.90032e-13 | 0.0002 | 52.695 | 0.023 | 0.013 | 0.078 | 0.56460 |
| 14 | 73011885 | rs2239222 | G | A | 0.019 | 0.003 | 7.39997e-10 | 0.0002 | 37.904 | 0.019 | 0.014 | 0.173 | 0.35390 |
| 10 | 122875589 | rs2254069 | A | G | -0.033 | 0.005 | 9.49948e-13 | 0.0002 | 50.950 | -0.001 | 0.018 | 0.957 | 0.11430 |
| 3 | 105455955 | rs2289746 | C | T | 0.018 | 0.003 | 2.10000e-08 | 0.0001 | 31.372 | 0.003 | 0.014 | 0.829 | 0.66000 |
| 1 | 11124885 | rs2486922 | C | T | -0.023 | 0.003 | 8.00018e-12 | 0.0002 | 46.761 | -0.009 | 0.015 | 0.557 | 0.74160 |
| 14 | 105262368 | rs2498786 | G | C | -0.023 | 0.003 | 9.89920e-14 | 0.0002 | 55.382 | 0.012 | 0.014 | 0.390 | 0.61330 |
| 5 | 53307772 | rs255755 | G | A | -0.025 | 0.003 | 2.49977e-13 | 0.0002 | 53.535 | -0.028 | 0.015 | 0.064 | 0.29720 |
| 20 | 17844492 | rs2618567 | T | G | -0.019 | 0.003 | 5.60003e-10 | 0.0002 | 38.440 | 0.028 | 0.014 | 0.045 | 0.61330 |
| 1 | 150958836 | rs267733 | G | A | -0.027 | 0.004 | 9.89920e-12 | 0.0002 | 46.341 | -0.011 | 0.019 | 0.564 | 0.13820 |
| 19 | 18237882 | rs273492 | A | G | -0.028 | 0.003 | 1.59993e-16 | 0.0003 | 68.072 | -0.027 | 0.015 | 0.077 | 0.25450 |
| 9 | 92274389 | rs28372209 | G | A | -0.016 | 0.003 | 3.20000e-08 | 0.0001 | 30.593 | 0.004 | 0.013 | 0.745 | 0.51490 |
| 15 | 63790662 | rs28539993 | T | C | 0.027 | 0.003 | 4.70002e-19 | 0.0003 | 79.531 | -0.006 | 0.014 | 0.676 | 0.39560 |
| 14 | 94844947 | rs28929474 | T | C | 0.125 | 0.011 | 6.90081e-32 | 0.0006 | 138.100 | -0.007 | 0.048 | 0.891 | 0.01690 |
| 16 | 81534790 | rs2925979 | C | T | 0.025 | 0.003 | 4.00037e-15 | 0.0003 | 61.723 | 0.007 | 0.014 | 0.607 | 0.71170 |
| 5 | 157970432 | rs2963431 | T | C | -0.023 | 0.003 | 3.50026e-11 | 0.0002 | 43.848 | 0.003 | 0.018 | 0.870 | 0.24950 |
| 1 | 219727779 | rs3001032 | C | T | 0.021 | 0.003 | 3.59998e-11 | 0.0002 | 43.808 | 0.022 | 0.014 | 0.125 | 0.31610 |
| 11 | 65217589 | rs3015966 | C | T | -0.024 | 0.003 | 2.70023e-14 | 0.0003 | 57.914 | 0.005 | 0.017 | 0.750 | 0.31710 |
| 20 | 62212392 | rs310670 | C | G | -0.017 | 0.003 | 2.90001e-08 | 0.0001 | 30.787 | 0.017 | 0.014 | 0.208 | 0.66300 |
| 6 | 31119976 | rs3130500 | A | T | -0.027 | 0.003 | 1.80011e-15 | 0.0003 | 63.240 | 0.000 | 0.015 | 0.980 | 0.77730 |
| 14 | 23685442 | rs34210072 | A | G | 0.024 | 0.004 | 3.59998e-10 | 0.0002 | 39.292 | -0.014 | 0.017 | 0.390 | 0.18690 |
| 19 | 46384830 | rs34255979 | T | C | 0.051 | 0.005 | 3.19963e-29 | 0.0006 | 125.941 | -0.028 | 0.028 | 0.329 | 0.09245 |
| 9 | 137267991 | rs34463468 | T | C | -0.031 | 0.004 | 4.30031e-18 | 0.0003 | 75.194 | -0.003 | 0.016 | 0.825 | 0.76740 |
| 1 | 154599778 | rs35504625 | G | C | 0.030 | 0.003 | 3.10027e-24 | 0.0004 | 103.128 | -0.005 | 0.013 | 0.716 | 0.47120 |
| 22 | 44324855 | rs3747207 | A | G | 0.036 | 0.004 | 5.90065e-23 | 0.0004 | 97.324 | -0.005 | 0.016 | 0.767 | 0.22270 |
| 11 | 2179204 | rs3842763 | T | G | 0.028 | 0.003 | 2.29985e-15 | 0.0003 | 62.775 | -0.028 | 0.016 | 0.078 | 0.28430 |
| 4 | 103915618 | rs3960788 | C | T | 0.017 | 0.003 | 6.80002e-09 | 0.0001 | 33.603 | -0.004 | 0.014 | 0.765 | 0.41750 |
| 5 | 55804552 | rs40270 | C | A | -0.039 | 0.004 | 2.80027e-28 | 0.0005 | 121.611 | -0.013 | 0.015 | 0.378 | 0.73860 |
| 12 | 51144432 | rs4307773 | C | T | -0.026 | 0.003 | 9.30037e-19 | 0.0003 | 78.207 | 0.004 | 0.014 | 0.747 | 0.58650 |
| 8 | 81461974 | rs440837 | G | A | 0.052 | 0.004 | 5.19996e-46 | 0.0009 | 202.781 | 0.039 | 0.017 | 0.018 | 0.24650 |
| 1 | 91530001 | rs469721 | T | C | -0.026 | 0.004 | 3.29989e-12 | 0.0002 | 48.477 | -0.012 | 0.016 | 0.472 | 0.18790 |
| 19 | 7223848 | rs4804416 | G | T | -0.036 | 0.003 | 9.70063e-34 | 0.0006 | 146.580 | -0.009 | 0.014 | 0.489 | 0.42050 |
| 22 | 21940189 | rs4820091 | G | T | 0.024 | 0.004 | 2.59998e-10 | 0.0002 | 39.980 | -0.018 | 0.014 | 0.200 | 0.18290 |
| 9 | 123507855 | rs4837794 | C | T | -0.026 | 0.003 | 1.99986e-16 | 0.0003 | 67.582 | -0.014 | 0.014 | 0.306 | 0.66700 |
| 11 | 119075323 | rs4938637 | A | G | 0.036 | 0.006 | 1.79999e-10 | 0.0002 | 40.674 | 0.077 | 0.042 | 0.066 | 0.08648 |
| 12 | 102810269 | rs5009837 | C | T | -0.028 | 0.003 | 2.99985e-17 | 0.0003 | 71.333 | 0.000 | 0.014 | 0.971 | 0.72660 |
| 20 | 49569025 | rs55987409 | T | C | 0.032 | 0.006 | 1.70000e-08 | 0.0001 | 31.761 | 0.004 | 0.029 | 0.895 | 0.06660 |
| 3 | 122359589 | rs56298514 | C | A | -0.020 | 0.003 | 1.09999e-08 | 0.0001 | 32.740 | 0.002 | 0.015 | 0.912 | 0.24750 |
| 15 | 96714816 | rs56332871 | A | C | 0.075 | 0.003 | 3.19890e-112 | 0.0022 | 506.763 | 0.008 | 0.015 | 0.601 | 0.30220 |
| 17 | 7538323 | rs57116414 | A | G | -0.149 | 0.008 | 4.60045e-70 | 0.0014 | 313.089 | -0.043 | 0.045 | 0.340 | 0.03777 |
| 20 | 51136424 | rs6063803 | G | A | -0.019 | 0.003 | 9.60064e-11 | 0.0002 | 41.896 | 0.010 | 0.014 | 0.454 | 0.59440 |
| 20 | 43040569 | rs6073431 | T | C | 0.033 | 0.003 | 1.99986e-27 | 0.0005 | 117.748 | -0.020 | 0.013 | 0.143 | 0.52490 |
| 20 | 33081906 | rs6120663 | A | C | -0.019 | 0.003 | 8.10028e-11 | 0.0002 | 42.223 | 0.010 | 0.013 | 0.473 | 0.45230 |
| 20 | 39920014 | rs6129802 | T | C | 0.024 | 0.004 | 6.79986e-11 | 0.0002 | 42.575 | -0.002 | 0.016 | 0.895 | 0.19580 |
| 10 | 63914890 | rs61850811 | A | G | 0.031 | 0.005 | 4.70002e-10 | 0.0002 | 38.790 | -0.004 | 0.022 | 0.873 | 0.07555 |
| 16 | 53811788 | rs62033400 | G | A | -0.024 | 0.003 | 7.29962e-15 | 0.0003 | 60.510 | 0.007 | 0.013 | 0.624 | 0.41550 |
| 3 | 150066540 | rs62271373 | A | T | -0.046 | 0.006 | 6.40030e-13 | 0.0002 | 51.727 | 0.029 | 0.032 | 0.367 | 0.06163 |
| 9 | 102162570 | rs62565259 | T | C | 0.023 | 0.004 | 5.89997e-09 | 0.0002 | 33.873 | -0.005 | 0.020 | 0.806 | 0.14710 |
| 11 | 32956492 | rs62618693 | T | C | 0.043 | 0.007 | 1.70000e-09 | 0.0002 | 36.341 | -0.007 | 0.043 | 0.865 | 0.03380 |
| 6 | 139829560 | rs651837 | G | A | 0.020 | 0.003 | 7.70016e-11 | 0.0002 | 42.339 | 0.000 | 0.013 | 0.979 | 0.57060 |
| 4 | 39686332 | rs6531735 | A | G | -0.017 | 0.003 | 4.30002e-09 | 0.0002 | 34.500 | -0.014 | 0.013 | 0.291 | 0.50400 |
| 18 | 57846077 | rs663640 | T | C | -0.022 | 0.004 | 5.49997e-10 | 0.0002 | 38.481 | -0.012 | 0.018 | 0.494 | 0.22170 |
| 2 | 42510018 | rs6736913 | G | A | -0.065 | 0.010 | 4.70002e-10 | 0.0002 | 38.795 | 0.073 | 0.052 | 0.156 | 0.98810 |
| 6 | 160776653 | rs673736 | A | G | 0.031 | 0.003 | 4.40048e-25 | 0.0005 | 107.025 | -0.003 | 0.013 | 0.810 | 0.48710 |
| 2 | 70537173 | rs6758199 | T | C | -0.045 | 0.006 | 2.39994e-14 | 0.0003 | 58.171 | 0.001 | 0.022 | 0.959 | 0.07157 |
| 20 | 32308275 | rs67611724 | T | C | -0.025 | 0.004 | 1.00000e-09 | 0.0002 | 37.253 | -0.019 | 0.018 | 0.282 | 0.17790 |
| 3 | 24520283 | rs6792725 | G | A | 0.035 | 0.003 | 2.49977e-26 | 0.0005 | 112.691 | -0.015 | 0.013 | 0.253 | 0.67100 |
| 5 | 127367998 | rs6860245 | C | G | 0.025 | 0.003 | 2.09991e-13 | 0.0002 | 53.909 | 0.014 | 0.018 | 0.457 | 0.21070 |
| 5 | 142792990 | rs6865292 | C | T | -0.019 | 0.003 | 2.90001e-08 | 0.0001 | 30.754 | -0.003 | 0.015 | 0.837 | 0.25750 |
| 3 | 135932359 | rs687339 | T | C | -0.068 | 0.004 | 1.20005e-83 | 0.0016 | 375.529 | -0.037 | 0.019 | 0.047 | 0.77040 |
| 6 | 41703041 | rs6939861 | A | G | -0.037 | 0.003 | 3.69999e-28 | 0.0005 | 121.046 | 0.004 | 0.015 | 0.783 | 0.29920 |
| 9 | 86583076 | rs696825 | T | C | 0.049 | 0.003 | 1.20005e-46 | 0.0009 | 205.652 | 0.019 | 0.014 | 0.188 | 0.28630 |
| 10 | 98974849 | rs701810 | T | C | -0.019 | 0.003 | 2.59998e-09 | 0.0002 | 35.427 | 0.020 | 0.014 | 0.155 | 0.28530 |
| 9 | 5728192 | rs7039093 | G | C | 0.018 | 0.003 | 8.19993e-10 | 0.0002 | 37.711 | -0.003 | 0.013 | 0.795 | 0.43440 |
| 16 | 88013029 | rs7194995 | A | C | -0.028 | 0.004 | 3.40017e-15 | 0.0003 | 62.004 | 0.003 | 0.015 | 0.868 | 0.77240 |
| 17 | 47221643 | rs7220103 | G | A | 0.060 | 0.007 | 4.30031e-16 | 0.0003 | 66.095 | -0.016 | 0.039 | 0.683 | 0.03877 |
| 17 | 17845800 | rs7224815 | T | A | -0.033 | 0.003 | 6.49980e-28 | 0.0005 | 119.947 | -0.001 | 0.013 | 0.964 | 0.48610 |
| 4 | 17993410 | rs724577 | C | A | -0.020 | 0.003 | 2.80001e-09 | 0.0002 | 35.327 | 0.011 | 0.014 | 0.430 | 0.70870 |
| 17 | 73149850 | rs72844546 | T | C | -0.021 | 0.003 | 3.90032e-12 | 0.0002 | 48.158 | 0.004 | 0.014 | 0.774 | 0.61630 |
| 12 | 103520884 | rs7298698 | C | T | -0.056 | 0.003 | 1.99986e-79 | 0.0016 | 356.095 | 0.016 | 0.014 | 0.255 | 0.50800 |
| 12 | 111438001 | rs7304705 | G | A | 0.055 | 0.005 | 7.10068e-24 | 0.0004 | 101.507 | 0.061 | 0.029 | 0.039 | 0.07356 |
| 12 | 121424861 | rs7310409 | G | A | 0.027 | 0.003 | 4.60045e-19 | 0.0003 | 79.606 | -0.009 | 0.014 | 0.498 | 0.58450 |
| 7 | 77055836 | rs740158 | C | T | 0.016 | 0.003 | 4.49997e-08 | 0.0001 | 29.908 | -0.014 | 0.013 | 0.282 | 0.48710 |
| 19 | 45745607 | rs74444983 | C | T | -0.021 | 0.003 | 6.90001e-10 | 0.0002 | 38.043 | 0.014 | 0.015 | 0.345 | 0.26140 |
| 12 | 25410741 | rs75130744 | C | G | -0.053 | 0.006 | 4.10015e-20 | 0.0004 | 84.358 | 0.020 | 0.021 | 0.337 | 0.07256 |
| 6 | 7255610 | rs75479205 | G | A | 0.025 | 0.004 | 1.69981e-11 | 0.0002 | 45.238 | -0.014 | 0.016 | 0.381 | 0.17590 |
| 19 | 35566151 | rs75702986 | A | G | 0.030 | 0.004 | 1.50003e-14 | 0.0003 | 59.060 | -0.013 | 0.016 | 0.413 | 0.17790 |
| 22 | 30764453 | rs757869 | A | G | -0.031 | 0.003 | 5.10035e-21 | 0.0004 | 88.489 | -0.002 | 0.016 | 0.904 | 0.75350 |
| 4 | 120106348 | rs7655064 | C | T | -0.025 | 0.004 | 1.40001e-08 | 0.0001 | 32.244 | 0.014 | 0.017 | 0.399 | 0.12820 |
| 4 | 159719955 | rs76610881 | G | A | 0.045 | 0.005 | 1.90020e-21 | 0.0004 | 90.401 | -0.048 | 0.020 | 0.019 | 0.09742 |
| 10 | 63674885 | rs7921378 | C | G | -0.020 | 0.003 | 7.59976e-12 | 0.0002 | 46.875 | 0.023 | 0.013 | 0.078 | 0.49010 |
| 15 | 96667048 | rs79240050 | A | G | 0.058 | 0.009 | 2.19999e-10 | 0.0002 | 40.256 | -0.055 | 0.032 | 0.087 | 0.01392 |
| 3 | 172294500 | rs79287178 | A | G | -0.054 | 0.009 | 1.40001e-09 | 0.0002 | 36.635 | -0.040 | 0.033 | 0.226 | 0.02584 |
| 15 | 53739426 | rs79391862 | C | A | -0.148 | 0.013 | 6.79986e-31 | 0.0006 | 133.576 | 0.017 | 0.038 | 0.654 | 0.01690 |
| 11 | 13356030 | rs7947951 | G | A | -0.028 | 0.003 | 1.69981e-18 | 0.0003 | 76.961 | 0.026 | 0.013 | 0.048 | 0.70280 |
| 10 | 5262267 | rs79717793 | A | G | -0.042 | 0.004 | 9.49948e-25 | 0.0005 | 105.506 | -0.025 | 0.019 | 0.196 | 0.12030 |
| 12 | 46267260 | rs80127816 | A | G | -0.038 | 0.007 | 7.90005e-09 | 0.0001 | 33.299 | 0.044 | 0.038 | 0.250 | 0.03877 |
| 15 | 53093869 | rs8025155 | G | C | -0.045 | 0.004 | 1.39991e-25 | 0.0005 | 109.246 | 0.026 | 0.025 | 0.313 | 0.11430 |
| 6 | 26200677 | rs806794 | G | A | 0.024 | 0.003 | 4.30031e-13 | 0.0002 | 52.523 | 0.007 | 0.013 | 0.613 | 0.30120 |
| 18 | 9094245 | rs8087306 | C | G | -0.019 | 0.003 | 3.09999e-09 | 0.0002 | 35.124 | -0.010 | 0.014 | 0.497 | 0.71470 |
| 19 | 33888460 | rs8104606 | A | G | 0.018 | 0.003 | 1.50000e-08 | 0.0001 | 32.085 | 0.021 | 0.014 | 0.124 | 0.64120 |
| 12 | 124508758 | rs825453 | T | A | -0.023 | 0.003 | 1.80011e-14 | 0.0003 | 58.761 | 0.002 | 0.013 | 0.856 | 0.56160 |
| 19 | 2814181 | rs941408 | T | C | 0.045 | 0.003 | 6.70039e-44 | 0.0008 | 193.105 | -0.024 | 0.014 | 0.093 | 0.29320 |
| 6 | 34408579 | rs9766229 | C | A | 0.043 | 0.008 | 8.00000e-09 | 0.0001 | 33.281 | 0.034 | 0.024 | 0.160 | 0.04374 |
| 17 | 47395729 | rs9907478 | T | C | 0.132 | 0.005 | 4.19759e-142 | 0.0028 | 644.156 | -0.038 | 0.036 | 0.294 | 0.06362 |
| 1 | 214156165 | rs9970073 | A | G | 0.018 | 0.003 | 3.79997e-09 | 0.0002 | 34.706 | -0.003 | 0.013 | 0.837 | 0.40760 |
| 6 | 43757896 | rs998584 | A | C | -0.021 | 0.003 | 1.10002e-12 | 0.0002 | 50.672 | -0.004 | 0.013 | 0.791 | 0.50400 |
| 8 | 9183358 | rs9987289 | G | A | 0.041 | 0.005 | 1.29987e-15 | 0.0003 | 63.903 | 0.021 | 0.019 | 0.270 | 0.92540 |

**Supplementary table 1.21. Detailed information of instrumental SNPs for Oestradiol in the analysis of PPD.**

| **Chr** | **Position** | **SNP** | **EA** | **OA** | **beta.exposure** | **se.exposure** | **pval.exposure** | **R2** | **F_val** | **beta.outcome** | **se.outcome** | **pval.outcome** | **Eaf.outcome** |
| --- | --- | --- | --- | --- | --- | --- | --- | --- | --- | --- | --- | --- | --- |
| 22 | 22586747 | rs117861337 | A | C | 0.172 | 0.037 | 3.50002e-06 | 5e-04 | 21.507 | 0.053 | 0.041 | 0.199 | 0.01392 |
| 2 | 213584890 | rs12621317 | A | G | 0.045 | 0.008 | 1.29999e-07 | 5e-04 | 27.873 | 0.001 | 0.016 | 0.931 | 0.17000 |
| 1 | 180548800 | rs143808140 | A | G | 0.182 | 0.039 | 3.29997e-06 | 4e-04 | 21.618 | -0.007 | 0.048 | 0.884 | 0.01193 |
| 18 | 32151415 | rs1439128 | C | T | 0.042 | 0.008 | 5.39995e-07 | 5e-04 | 25.112 | 0.001 | 0.016 | 0.928 | 0.17790 |
| 4 | 137529847 | rs17048801 | A | T | -0.029 | 0.006 | 4.30002e-06 | 4e-04 | 21.109 | 0.016 | 0.013 | 0.235 | 0.39760 |
| 5 | 19392363 | rs17279060 | C | T | 0.104 | 0.022 | 1.70000e-06 | 4e-04 | 22.948 | -0.019 | 0.058 | 0.744 | 0.02286 |
| 17 | 33232422 | rs184032510 | C | T | 0.115 | 0.025 | 4.39997e-06 | 4e-04 | 21.098 | -0.010 | 0.027 | 0.718 | 0.02883 |
| 15 | 33691764 | rs1878302 | T | G | 0.043 | 0.009 | 1.00000e-06 | 5e-04 | 23.931 | 0.029 | 0.020 | 0.150 | 0.12230 |
| 20 | 62883134 | rs351893 | A | G | -0.028 | 0.006 | 4.30002e-06 | 4e-04 | 21.119 | 0.016 | 0.015 | 0.264 | 0.42940 |
| 11 | 107762576 | rs61906909 | C | G | 0.036 | 0.007 | 1.50000e-06 | 4e-04 | 23.112 | 0.005 | 0.016 | 0.747 | 0.25750 |
| 17 | 7537792 | rs727428 | C | T | 0.041 | 0.006 | 2.60016e-11 | 8e-04 | 44.482 | -0.012 | 0.014 | 0.361 | 0.57260 |
| 11 | 30242287 | rs74485684 | C | T | -0.044 | 0.008 | 1.40001e-07 | 5e-04 | 27.676 | 0.001 | 0.017 | 0.953 | 0.16100 |
| 14 | 25265632 | rs77075600 | T | C | -0.054 | 0.011 | 2.69998e-06 | 4e-04 | 22.002 | -0.040 | 0.026 | 0.131 | 0.08549 |
| 6 | 56237709 | rs7769615 | C | T | 0.051 | 0.011 | 1.70000e-06 | 4e-04 | 22.952 | 0.036 | 0.022 | 0.096 | 0.10540 |
| 13 | 31165823 | rs9551946 | A | G | -0.029 | 0.006 | 2.80001e-06 | 4e-04 | 21.929 | 0.007 | 0.014 | 0.632 | 0.41450 |
| 3 | 138936698 | rs9823043 | T | A | -0.031 | 0.006 | 1.20000e-06 | 5e-04 | 23.616 | 0.025 | 0.013 | 0.064 | 0.59050 |
| 16 | 7767262 | rs9929491 | C | T | -0.045 | 0.010 | 2.59998e-06 | 4e-04 | 22.113 | 0.011 | 0.017 | 0.511 | 0.11330 |

**Supplementary table 2.22. Detailed information of instrumental SNPs for FT4 in the analysis of PPD.**

| **Chr** | **Position** | **SNP** | **EA** | **OA** | **beta.exposure** | **se.exposure** | **pval.exposure** | **R2** | **F_val** | **beta.outcome** | **se.outcome** | **pval.outcome** | **Eaf.outcome** |
| --- | --- | --- | --- | --- | --- | --- | --- | --- | --- | --- | --- | --- | --- |
| 1 | 54375570 | rs2235544 | A | C | 0.138 | 0.009 | 8.232e-57 | 0.0094 | 251.605 | -0.021 | 0.013 | 0.123 | 0.545 |
| 3 | 181726693 | rs11711652 | T | C | 0.078 | 0.013 | 4.423e-10 | 0.0013 | 38.738 | 0.016 | 0.015 | 0.297 | 0.737 |
| 4 | 170992674 | rs6855450 | T | C | 0.125 | 0.015 | 5.699e-17 | 0.0011 | 70.492 | -0.032 | 0.030 | 0.283 | 0.054 |
| 6 | 100600097 | rs17789218 | T | C | -0.082 | 0.011 | 7.302e-14 | 0.0014 | 56.457 | -0.013 | 0.016 | 0.438 | 0.792 |
| 6 | 100856746 | rs36028076 | T | C | -0.073 | 0.013 | 1.257e-08 | 0.0010 | 32.526 | 0.028 | 0.023 | 0.228 | 0.908 |
| 9 | 127016876 | rs10986292 | T | G | 0.051 | 0.009 | 2.459e-08 | 0.0011 | 30.730 | 0.006 | 0.014 | 0.661 | 0.652 |
| 9 | 139092679 | rs4842131 | T | C | -0.111 | 0.010 | 2.669e-28 | 0.0062 | 121.654 | 0.030 | 0.013 | 0.026 | 0.490 |
| 9 | 100591463 | rs7045138 | T | C | 0.068 | 0.009 | 6.659e-15 | 0.0020 | 60.732 | 0.000 | 0.013 | 0.997 | 0.564 |
| 14 | 80669580 | rs225014 | T | C | 0.051 | 0.009 | 1.303e-08 | 0.0013 | 32.237 | -0.010 | 0.015 | 0.485 | 0.719 |
| 14 | 101998986 | rs7151700 | A | C | 0.059 | 0.009 | 4.049e-10 | 0.0017 | 39.396 | 0.001 | 0.014 | 0.955 | 0.581 |
| 18 | 29306737 | rs113107469 | T | C | 0.178 | 0.029 | 1.351e-09 | 0.0009 | 36.821 | 0.104 | 0.047 | 0.029 | 0.020 |

**Supplementary table 2.23. Detailed information of instrumental SNPs for TSH in the analysis of PPD.**

| **Chr** | **Position** | **SNP** | **EA** | **OA** | **beta.exposure** | **se.exposure** | **pval.exposure** | **R2** | **F_val** | **beta.outcome** | **se.outcome** | **pval.outcome** | **Eaf.outcome** |
| --- | --- | --- | --- | --- | --- | --- | --- | --- | --- | --- | --- | --- | --- |
| 19 | 7224350 | rs10410204 | T | C | 0.054 | 0.009 | 2.478e-10 | 0.0014 | 39.913 | -0.009 | 0.013 | 0.494 | 0.57360 |
| 17 | 70121339 | rs1042673 | A | G | -0.058 | 0.008 | 2.919e-12 | 0.0016 | 48.663 | -0.004 | 0.013 | 0.780 | 0.41150 |
| 10 | 101283330 | rs10748781 | A | C | -0.054 | 0.009 | 5.794e-10 | 0.0012 | 38.099 | -0.014 | 0.014 | 0.304 | 0.57550 |
| 15 | 89119711 | rs11638818 | A | G | -0.051 | 0.009 | 7.925e-09 | 0.0009 | 32.966 | -0.010 | 0.014 | 0.479 | 0.32600 |
| 14 | 36738361 | rs116909374 | T | C | -0.190 | 0.032 | 3.255e-09 | 0.0014 | 34.964 | 0.008 | 0.039 | 0.838 | 0.04175 |
| 6 | 43904780 | rs11755845 | T | C | -0.070 | 0.010 | 9.603e-13 | 0.0016 | 50.712 | 0.009 | 0.016 | 0.593 | 0.24550 |
| 1 | 19830373 | rs12083293 | A | C | 0.088 | 0.009 | 6.420e-24 | 0.0038 | 100.911 | -0.006 | 0.013 | 0.646 | 0.52190 |
| 2 | 217625523 | rs13015993 | A | G | 0.063 | 0.010 | 5.709e-10 | 0.0019 | 38.538 | -0.007 | 0.014 | 0.596 | 0.71570 |
| 1 | 108357391 | rs17020122 | T | C | 0.120 | 0.016 | 4.077e-14 | 0.0035 | 57.340 | -0.013 | 0.021 | 0.521 | 0.06759 |
| 15 | 49711185 | rs17477923 | T | C | 0.088 | 0.009 | 6.820e-21 | 0.0022 | 88.522 | 0.004 | 0.014 | 0.773 | 0.27530 |
| 5 | 76532571 | rs2127387 | A | G | 0.132 | 0.009 | 3.077e-50 | 0.0081 | 221.307 | 0.023 | 0.013 | 0.091 | 0.41250 |
| 5 | 76471463 | rs2359775 | A | T | -0.077 | 0.012 | 5.163e-11 | 0.0030 | 43.425 | 0.002 | 0.015 | 0.907 | 0.18590 |
| 6 | 43804808 | rs2396083 | C | G | 0.091 | 0.010 | 2.501e-20 | 0.0038 | 85.657 | 0.005 | 0.014 | 0.709 | 0.69380 |
| 8 | 133951991 | rs2739067 | A | G | -0.052 | 0.009 | 2.762e-09 | 0.0014 | 35.457 | 0.018 | 0.014 | 0.202 | 0.60340 |
| 14 | 93587465 | rs28709909 | T | G | -0.065 | 0.010 | 4.660e-10 | 0.0014 | 39.062 | -0.004 | 0.017 | 0.836 | 0.21770 |
| 1 | 61608473 | rs334729 | C | G | -0.176 | 0.020 | 4.946e-18 | 0.0051 | 74.742 | -0.101 | 0.046 | 0.027 | 0.95920 |
| 16 | 14412536 | rs40393 | T | C | 0.050 | 0.009 | 8.614e-09 | 0.0010 | 32.897 | -0.012 | 0.014 | 0.370 | 0.63020 |
| 4 | 149632011 | rs59840922 | A | G | -0.119 | 0.011 | 1.057e-25 | 0.0040 | 109.331 | 0.004 | 0.020 | 0.826 | 0.19480 |
| 14 | 81591396 | rs72691624 | T | C | -0.056 | 0.010 | 1.011e-08 | 0.0012 | 32.770 | 0.029 | 0.017 | 0.082 | 0.24450 |
| 16 | 79747855 | rs73575083 | A | G | 0.077 | 0.009 | 5.294e-18 | 0.0024 | 74.463 | -0.005 | 0.014 | 0.711 | 0.34190 |
| 6 | 166046483 | rs753760 | C | G | 0.095 | 0.009 | 5.615e-24 | 0.0037 | 102.139 | -0.020 | 0.015 | 0.191 | 0.32310 |
| 11 | 45227830 | rs7940871 | T | C | -0.057 | 0.009 | 3.565e-10 | 0.0010 | 39.510 | -0.035 | 0.014 | 0.016 | 0.30620 |
| 9 | 136149098 | rs8176645 | A | T | 0.058 | 0.009 | 1.160e-10 | 0.0016 | 41.674 | -0.002 | 0.013 | 0.905 | 0.39660 |
| 6 | 148521292 | rs9497965 | T | C | 0.054 | 0.009 | 1.179e-09 | 0.0014 | 37.377 | 0.018 | 0.014 | 0.192 | 0.38170 |
| 13 | 24773803 | rs9511141 | T | C | -0.055 | 0.009 | 2.214e-09 | 0.0011 | 35.610 | -0.005 | 0.014 | 0.747 | 0.35590 |

**Supplementary table 2.24. Detailed information of instrumental SNPs for incTSH in the analysis of PPD.**

| **Chr** | **Position** | **SNP** | **EA** | **OA** | **beta.exposure** | **se.exposure** | **pval.exposure** | **R2** | **F_val** | **beta.outcome** | **se.outcome** | **pval.outcome** | **Eaf.outcome** |
| --- | --- | --- | --- | --- | --- | --- | --- | --- | --- | --- | --- | --- | --- |
| 2 | 1407628 | rs11675342 | T | C | 0.160 | 0.025 | 1.477e-10 | 0.0123 | 41.165 | 0.005 | 0.013 | 0.726 | 0.45530 |
| 9 | 75458704 | rs116878894 | C | G | 0.707 | 0.120 | 4.189e-09 | 0.0064 | 34.519 | -0.001 | 0.047 | 0.991 | 0.01590 |
| 17 | 43302259 | rs12449792 | T | C | 0.157 | 0.028 | 2.409e-08 | 0.0106 | 31.058 | 0.010 | 0.014 | 0.466 | 0.41950 |
| 5 | 76521868 | rs1382879 | T | C | -0.153 | 0.025 | 2.149e-09 | 0.0106 | 35.906 | -0.023 | 0.013 | 0.087 | 0.41150 |
| 6 | 74446214 | rs148575660 | A | G | -0.703 | 0.118 | 2.526e-09 | 0.0054 | 35.513 | -0.050 | 0.050 | 0.315 | 0.01193 |
| 6 | 166050119 | rs2983514 | A | G | 0.154 | 0.027 | 1.583e-08 | 0.0097 | 31.863 | -0.007 | 0.015 | 0.608 | 0.34290 |
| 12 | 111973358 | rs597808 | A | G | 0.180 | 0.026 | 3.465e-12 | 0.0113 | 48.353 | -0.010 | 0.013 | 0.448 | 0.53380 |
| 1 | 19836221 | rs75491569 | T | C | -0.238 | 0.037 | 8.665e-11 | 0.0166 | 42.197 | 0.037 | 0.020 | 0.063 | 0.14610 |

**Supplementary table 2.25. Detailed information of instrumental SNPs for decTSH in the analysis of PPD.**

| **Chr** | **Position** | **SNP** | **EA** | **OA** | **beta.exposure** | **se.exposure** | **pval.exposure** | **R2** | **F_val** | **beta.outcome** | **se.outcome** | **pval.outcome** | **Eaf.outcome** |
| --- | --- | --- | --- | --- | --- | --- | --- | --- | --- | --- | --- | --- | --- |
| 11 | 45237858 | rs11038357 | A | T | 0.235 | 0.035 | 3.088e-11 | 0.0153 | 44.106 | -0.032 | 0.014 | 0.027 | 0.27930 |
| 1 | 19845279 | rs12410532 | T | C | 0.229 | 0.040 | 7.480e-09 | 0.0145 | 33.383 | 0.033 | 0.020 | 0.107 | 0.14210 |
| 5 | 76468398 | rs1479560 | T | G | 0.257 | 0.043 | 1.459e-09 | 0.0301 | 36.538 | 0.002 | 0.016 | 0.910 | 0.15810 |
| 1 | 230364466 | rs148591245 | A | G | 0.836 | 0.145 | 8.746e-09 | 0.0083 | 33.112 | -0.043 | 0.052 | 0.405 | 0.01093 |
| 15 | 49711185 | rs17477923 | T | C | -0.245 | 0.034 | 6.123e-13 | 0.0171 | 51.798 | 0.004 | 0.014 | 0.773 | 0.27530 |
| 5 | 76535811 | rs2046045 | T | G | 0.329 | 0.034 | 4.353e-22 | 0.0498 | 93.407 | -0.023 | 0.013 | 0.092 | 0.41250 |
| 6 | 166050119 | rs2983514 | A | G | -0.199 | 0.032 | 5.235e-10 | 0.0162 | 38.510 | -0.007 | 0.015 | 0.608 | 0.34290 |
| 4 | 153615676 | rs6535864 | A | G | 0.660 | 0.117 | 1.950e-08 | 0.1054 | 31.566 | -0.216 | 0.132 | 0.103 | 0.97810 |
| 6 | 43906255 | rs66760320 | T | C | 0.192 | 0.035 | 4.428e-08 | 0.0143 | 30.047 | 0.009 | 0.016 | 0.574 | 0.25050 |
| 8 | 133883905 | rs79676842 | T | G | 0.698 | 0.098 | 1.033e-12 | 0.0084 | 50.775 | 0.009 | 0.044 | 0.837 | 0.02286 |
| 17 | 70376177 | rs8077245 | T | G | 0.205 | 0.033 | 5.022e-10 | 0.0157 | 38.741 | -0.006 | 0.013 | 0.643 | 0.56960 |
| 9 | 100546391 | rs925488 | A | G | 0.190 | 0.033 | 1.142e-08 | 0.0127 | 32.658 | 0.005 | 0.014 | 0.706 | 0.66100 |

| **Supplementary table 2.26. Detailed information of instrumental SNPs for**  **Serum 25-Hydroxyvitamin D levels adjusted BMI in the analysis of PPD.** | | | | | | | | |  |  |  |  |  |
| --- | --- | --- | --- | --- | --- | --- | --- | --- | --- | --- | --- | --- | --- |
| Chr | Position | SNP | EA | OA | beta.exposure | se.exposure | pval.exposure | R2 | F_value | beta.outcome | se.outcome | pval.outcome | Eaf.outcome |
| ee2 | 211540507 | rs1047891 | C | A | 0.013 | 0.002 | 2.58370e-09 | 0.0001 | 35.475 | -0.004 | 0.014 | 0.752 | 0.30120 |
| 10 | 64934548 | rs10822145 | C | T | 0.013 | 0.002 | 1.60462e-10 | 0.0001 | 40.897 | 0.013 | 0.013 | 0.339 | 0.49500 |
| 12 | 96375682 | rs10859995 | T | C | 0.040 | 0.002 | 1.11049e-89 | 0.0008 | 403.195 | 0.003 | 0.014 | 0.845 | 0.57550 |
| 1 | 155468732 | rs10908469 | A | C | -0.016 | 0.002 | 6.66590e-13 | 0.0001 | 51.640 | -0.004 | 0.015 | 0.803 | 0.27630 |
| 11 | 13539344 | rs11022863 | C | T | -0.022 | 0.004 | 6.59408e-09 | 0.0001 | 33.651 | -0.002 | 0.024 | 0.926 | 0.04871 |
| 16 | 57006378 | rs11076175 | A | G | -0.024 | 0.003 | 9.46213e-21 | 0.0002 | 87.271 | -0.045 | 0.018 | 0.012 | 0.20580 |
| 1 | 230304930 | rs11122455 | C | G | -0.013 | 0.002 | 3.41591e-10 | 0.0001 | 39.421 | -0.008 | 0.013 | 0.551 | 0.60930 |
| 12 | 38526387 | rs11182428 | T | C | 0.012 | 0.002 | 3.16865e-10 | 0.0001 | 39.568 | -0.016 | 0.013 | 0.216 | 0.51290 |
| 11 | 76471616 | rs1149597 | C | T | -0.022 | 0.003 | 1.77792e-16 | 0.0001 | 67.834 | -0.028 | 0.018 | 0.121 | 0.14510 |
| 16 | 82033810 | rs11542462 | G | A | 0.023 | 0.003 | 5.78604e-16 | 0.0001 | 65.509 | -0.017 | 0.023 | 0.465 | 0.15110 |
| 19 | 54658102 | rs11606 | C | G | -0.011 | 0.002 | 2.76841e-08 | 0.0001 | 30.863 | -0.010 | 0.014 | 0.463 | 0.40260 |
| 4 | 72565508 | rs11730758 | C | T | -0.072 | 0.002 | 1.00000e-200 | 0.0023 | 1187.777 | 0.024 | 0.014 | 0.095 | 0.34290 |
| 6 | 32586236 | rs11751024 | C | A | 0.013 | 0.002 | 3.75952e-10 | 0.0001 | 39.234 | 0.014 | 0.014 | 0.306 | 0.43240 |
| 11 | 13210063 | rs117862422 | T | C | 0.054 | 0.009 | 1.91393e-10 | 0.0001 | 40.553 | -0.028 | 0.033 | 0.394 | 0.01491 |
| 8 | 116988527 | rs12056768 | T | G | 0.022 | 0.002 | 3.27304e-28 | 0.0002 | 121.308 | -0.024 | 0.013 | 0.076 | 0.57550 |
| 1 | 152179152 | rs12123821 | C | T | -0.077 | 0.005 | 3.70853e-63 | 0.0005 | 281.406 | -0.008 | 0.034 | 0.814 | 0.04374 |
| 12 | 21352541 | rs12317268 | A | G | 0.021 | 0.003 | 5.26505e-14 | 0.0001 | 56.628 | 0.016 | 0.015 | 0.278 | 0.17790 |
| 19 | 11955767 | rs12462826 | G | A | 0.013 | 0.002 | 1.51661e-10 | 0.0001 | 41.007 | -0.004 | 0.014 | 0.801 | 0.35490 |
| 2 | 27730940 | rs1260326 | T | C | -0.022 | 0.002 | 3.95221e-29 | 0.0002 | 125.502 | -0.004 | 0.014 | 0.762 | 0.58950 |
| 17 | 7570878 | rs12949853 | G | A | -0.014 | 0.003 | 1.22845e-08 | 0.0001 | 32.441 | 0.020 | 0.018 | 0.261 | 0.77530 |
| 2 | 21274167 | rs13011615 | A | T | -0.017 | 0.003 | 4.03978e-09 | 0.0001 | 34.604 | 0.006 | 0.019 | 0.768 | 0.16700 |
| 4 | 73034261 | rs13140922 | C | T | -0.054 | 0.004 | 2.56004e-49 | 0.0004 | 217.928 | 0.012 | 0.026 | 0.647 | 0.05567 |
| 9 | 107669073 | rs13284054 | T | C | -0.019 | 0.003 | 1.81680e-09 | 0.0001 | 36.161 | -0.019 | 0.021 | 0.368 | 0.12620 |
| 14 | 50829560 | rs142004400 | A | C | 0.033 | 0.005 | 6.42900e-10 | 0.0001 | 38.187 | 0.038 | 0.047 | 0.423 | 0.02883 |
| 19 | 11190534 | rs142158911 | G | A | -0.027 | 0.003 | 7.16666e-18 | 0.0001 | 74.170 | 0.021 | 0.022 | 0.344 | 0.11230 |
| 6 | 40962537 | rs143069752 | T | A | -0.022 | 0.004 | 1.16362e-08 | 0.0001 | 32.547 | -0.002 | 0.028 | 0.944 | 0.08250 |
| 15 | 58683366 | rs1532085 | A | G | -0.026 | 0.002 | 3.62404e-38 | 0.0003 | 166.841 | -0.022 | 0.013 | 0.098 | 0.63520 |
| 11 | 14918216 | rs1562902 | C | T | 0.061 | 0.002 | 1.00000e-200 | 0.0018 | 942.870 | 0.015 | 0.013 | 0.247 | 0.55860 |
| 7 | 21563471 | rs17144574 | T | C | 0.015 | 0.002 | 6.73000e-11 | 0.0001 | 42.596 | 0.011 | 0.016 | 0.486 | 0.22170 |
| 11 | 71135136 | rs1792283 | T | C | 0.097 | 0.003 | 1.00000e-200 | 0.0028 | 1473.627 | 0.000 | 0.014 | 0.987 | 0.26440 |
| 15 | 58723675 | rs1800588 | C | T | 0.033 | 0.002 | 1.49810e-43 | 0.0004 | 191.497 | -0.006 | 0.015 | 0.692 | 0.20870 |
| 7 | 107117447 | rs1858889 | A | C | -0.011 | 0.002 | 1.38169e-08 | 0.0001 | 32.213 | -0.009 | 0.013 | 0.488 | 0.50990 |
| 5 | 118627319 | rs1966478 | T | C | 0.012 | 0.002 | 6.91644e-09 | 0.0001 | 33.558 | -0.002 | 0.014 | 0.864 | 0.71870 |
| 2 | 234622379 | rs2012736 | C | A | 0.048 | 0.004 | 1.10147e-40 | 0.0003 | 178.367 | 0.009 | 0.025 | 0.734 | 0.09642 |
| 18 | 61366207 | rs2037511 | G | A | -0.017 | 0.003 | 1.06696e-10 | 0.0001 | 41.695 | -0.005 | 0.018 | 0.769 | 0.17790 |
| 22 | 31535872 | rs2074735 | G | C | -0.028 | 0.004 | 4.00251e-12 | 0.0001 | 48.123 | -0.012 | 0.019 | 0.521 | 0.08052 |
| 19 | 48376995 | rs212100 | T | C | 0.066 | 0.003 | 7.83577e-136 | 0.0012 | 615.307 | -0.019 | 0.018 | 0.295 | 0.84990 |
| 15 | 100231033 | rs2123930 | G | A | 0.014 | 0.002 | 1.30305e-10 | 0.0001 | 41.304 | -0.005 | 0.014 | 0.703 | 0.29820 |
| 1 | 63025942 | rs2131925 | G | T | 0.022 | 0.002 | 2.42863e-27 | 0.0002 | 117.332 | 0.021 | 0.015 | 0.160 | 0.69480 |
| 20 | 39142516 | rs2207132 | G | A | 0.039 | 0.005 | 1.96319e-12 | 0.0001 | 49.520 | -0.008 | 0.027 | 0.779 | 0.04076 |
| 21 | 16339172 | rs2229742 | G | C | 0.025 | 0.003 | 5.89653e-15 | 0.0001 | 60.936 | 0.029 | 0.021 | 0.175 | 0.09841 |
| 3 | 49881134 | rs2246832 | A | T | -0.017 | 0.002 | 1.86869e-18 | 0.0001 | 76.824 | 0.051 | 0.014 | 0.000 | 0.47320 |
| 10 | 113913222 | rs2297991 | T | C | -0.012 | 0.002 | 3.51448e-08 | 0.0001 | 30.401 | 0.017 | 0.014 | 0.233 | 0.67690 |
| 7 | 133536351 | rs2346264 | A | C | 0.015 | 0.002 | 8.86225e-10 | 0.0001 | 37.560 | 0.004 | 0.018 | 0.821 | 0.79520 |
| 6 | 131918839 | rs2608984 | A | T | 0.021 | 0.003 | 2.78567e-15 | 0.0001 | 62.413 | 0.015 | 0.016 | 0.347 | 0.16700 |
| 1 | 220970499 | rs2642439 | A | G | 0.015 | 0.002 | 6.17571e-12 | 0.0001 | 47.273 | 0.012 | 0.015 | 0.418 | 0.70580 |
| 17 | 79217478 | rs2659007 | G | A | -0.011 | 0.002 | 9.75225e-09 | 0.0001 | 32.890 | -0.037 | 0.013 | 0.006 | 0.45530 |
| 14 | 104001517 | rs2756119 | G | A | -0.012 | 0.002 | 2.35302e-09 | 0.0001 | 35.657 | 0.010 | 0.014 | 0.464 | 0.40060 |
| 11 | 120114421 | rs2847500 | G | A | 0.022 | 0.003 | 1.21523e-13 | 0.0001 | 54.984 | 0.026 | 0.019 | 0.171 | 0.14210 |
| 4 | 69574708 | rs293454 | G | A | 0.017 | 0.002 | 7.52429e-15 | 0.0001 | 60.456 | 0.020 | 0.013 | 0.145 | 0.31710 |
| 17 | 66464414 | rs2952289 | C | T | -0.018 | 0.002 | 3.22420e-13 | 0.0001 | 53.067 | 0.013 | 0.017 | 0.443 | 0.79520 |
| 16 | 89893375 | rs34177108 | C | A | 0.012 | 0.002 | 3.95618e-08 | 0.0001 | 30.171 | 0.024 | 0.017 | 0.153 | 0.25450 |
| 3 | 141720712 | rs34186890 | A | G | 0.015 | 0.002 | 2.25187e-11 | 0.0001 | 44.739 | 0.002 | 0.015 | 0.908 | 0.26140 |
| 13 | 55710231 | rs34284484 | T | G | 0.012 | 0.002 | 1.37258e-08 | 0.0001 | 32.226 | 0.029 | 0.015 | 0.053 | 0.28930 |
| 8 | 25889606 | rs34726834 | C | T | -0.015 | 0.002 | 4.49358e-11 | 0.0001 | 43.386 | -0.002 | 0.015 | 0.880 | 0.26940 |
| 1 | 17560195 | rs35408430 | C | T | 0.021 | 0.002 | 5.81643e-25 | 0.0002 | 106.470 | 0.017 | 0.014 | 0.215 | 0.36180 |
| 19 | 36342212 | rs3814995 | C | T | 0.013 | 0.002 | 2.33427e-09 | 0.0001 | 35.673 | -0.008 | 0.014 | 0.555 | 0.31110 |
| 4 | 166251068 | rs3890624 | A | G | -0.011 | 0.002 | 3.19364e-08 | 0.0001 | 30.586 | -0.016 | 0.014 | 0.265 | 0.38870 |
| 18 | 47144223 | rs4121823 | T | A | 0.019 | 0.003 | 1.32891e-11 | 0.0001 | 45.771 | -0.001 | 0.018 | 0.964 | 0.84890 |
| 7 | 75612803 | rs41301394 | C | T | -0.013 | 0.002 | 7.10925e-09 | 0.0001 | 33.505 | -0.014 | 0.014 | 0.304 | 0.29320 |
| 19 | 45411941 | rs429358 | T | C | 0.023 | 0.003 | 1.76054e-17 | 0.0001 | 72.396 | -0.032 | 0.017 | 0.069 | 0.15510 |
| 4 | 15892159 | rs4364259 | G | A | -0.017 | 0.002 | 2.23282e-11 | 0.0001 | 44.755 | 0.001 | 0.016 | 0.941 | 0.21470 |
| 10 | 94839724 | rs4418728 | G | T | -0.012 | 0.002 | 7.53657e-10 | 0.0001 | 37.877 | 0.013 | 0.013 | 0.342 | 0.48710 |
| 4 | 57745481 | rs4616820 | C | T | 0.012 | 0.002 | 5.31702e-10 | 0.0001 | 38.558 | 0.004 | 0.013 | 0.759 | 0.47510 |
| 4 | 70032525 | rs4694668 | C | T | 0.028 | 0.002 | 3.76409e-46 | 0.0004 | 203.411 | 0.016 | 0.013 | 0.241 | 0.52290 |
| 1 | 46027355 | rs512083 | T | C | -0.012 | 0.002 | 6.55690e-10 | 0.0001 | 38.148 | -0.038 | 0.013 | 0.004 | 0.47710 |
| 11 | 14263331 | rs56051708 | A | G | 0.057 | 0.005 | 1.11477e-31 | 0.0003 | 137.156 | -0.011 | 0.035 | 0.756 | 0.03976 |
| 8 | 61312205 | rs57459725 | C | G | 0.018 | 0.003 | 1.06936e-09 | 0.0001 | 37.194 | -0.029 | 0.018 | 0.094 | 0.14910 |
| 2 | 32579999 | rs58387006 | A | C | 0.014 | 0.002 | 6.33159e-09 | 0.0001 | 33.730 | -0.004 | 0.016 | 0.801 | 0.23560 |
| 22 | 23365501 | rs6003465 | T | C | 0.012 | 0.002 | 1.19014e-08 | 0.0001 | 32.503 | 0.010 | 0.015 | 0.490 | 0.34790 |
| 11 | 14421218 | rs61147618 | C | T | 0.127 | 0.004 | 1.00000e-200 | 0.0021 | 1068.859 | -0.009 | 0.028 | 0.737 | 0.07256 |
| 15 | 63852834 | rs62012766 | T | C | 0.018 | 0.003 | 1.61169e-11 | 0.0001 | 45.393 | -0.045 | 0.016 | 0.005 | 0.18790 |
| 19 | 51514874 | rs62115743 | C | T | -0.027 | 0.004 | 3.20575e-14 | 0.0001 | 57.604 | -0.032 | 0.022 | 0.142 | 0.09940 |
| 3 | 125148287 | rs6438900 | C | G | -0.013 | 0.002 | 3.43630e-09 | 0.0001 | 34.919 | 0.012 | 0.014 | 0.394 | 0.28630 |
| 2 | 21190209 | rs6547409 | C | T | -0.028 | 0.005 | 6.56801e-10 | 0.0001 | 38.145 | -0.033 | 0.025 | 0.184 | 0.06362 |
| 1 | 2339139 | rs6671730 | G | A | 0.015 | 0.002 | 2.25018e-14 | 0.0001 | 58.300 | 0.005 | 0.013 | 0.707 | 0.41950 |
| 2 | 58979783 | rs6723486 | C | T | 0.011 | 0.002 | 4.24314e-08 | 0.0001 | 30.035 | -0.001 | 0.013 | 0.932 | 0.59640 |
| 2 | 101440151 | rs6724965 | A | G | 0.017 | 0.003 | 1.76125e-10 | 0.0001 | 40.715 | 0.042 | 0.016 | 0.011 | 0.17790 |
| 4 | 88178919 | rs6834488 | C | T | 0.015 | 0.002 | 1.95870e-13 | 0.0001 | 54.046 | 0.012 | 0.014 | 0.413 | 0.40260 |
| 11 | 71041704 | rs7112442 | C | T | 0.078 | 0.008 | 4.29305e-25 | 0.0002 | 107.072 | 0.000 | 0.040 | 0.992 | 0.01690 |
| 11 | 70471414 | rs71467497 | T | C | 0.033 | 0.006 | 1.73800e-08 | 0.0001 | 31.767 | -0.010 | 0.040 | 0.808 | 0.02485 |
| 4 | 72703145 | rs72645665 | G | A | 0.088 | 0.002 | 1.00000e-200 | 0.0024 | 1245.646 | 0.008 | 0.016 | 0.617 | 0.16500 |
| 4 | 73177780 | rs72653995 | C | T | 0.062 | 0.008 | 6.90459e-16 | 0.0001 | 65.160 | -0.097 | 0.039 | 0.013 | 0.02187 |
| 6 | 22801858 | rs72834856 | T | G | 0.024 | 0.004 | 4.86222e-10 | 0.0001 | 38.732 | -0.040 | 0.027 | 0.144 | 0.07555 |
| 12 | 111582630 | rs73413596 | T | C | -0.024 | 0.004 | 2.88691e-10 | 0.0001 | 39.750 | -0.060 | 0.028 | 0.033 | 0.08549 |
| 1 | 41835685 | rs7522116 | C | T | 0.013 | 0.002 | 2.57687e-10 | 0.0001 | 39.971 | -0.003 | 0.014 | 0.835 | 0.54080 |
| 1 | 109817192 | rs7528419 | A | G | -0.020 | 0.002 | 1.33612e-17 | 0.0001 | 72.941 | 0.017 | 0.016 | 0.295 | 0.21270 |
| 11 | 15096436 | rs75461927 | T | C | -0.031 | 0.004 | 7.81815e-14 | 0.0001 | 55.851 | 0.021 | 0.029 | 0.479 | 0.05964 |
| 2 | 118648261 | rs7569755 | G | A | -0.013 | 0.002 | 1.21331e-09 | 0.0001 | 36.948 | -0.005 | 0.016 | 0.764 | 0.28030 |
| 16 | 20392332 | rs77924615 | G | A | 0.014 | 0.003 | 8.09141e-09 | 0.0001 | 33.253 | 0.014 | 0.016 | 0.379 | 0.20380 |
| 4 | 3482213 | rs78649910 | T | A | 0.019 | 0.003 | 1.56950e-09 | 0.0001 | 36.446 | 0.001 | 0.020 | 0.955 | 0.10540 |
| 11 | 15685570 | rs7943358 | T | C | -0.017 | 0.002 | 9.25767e-13 | 0.0001 | 50.995 | 0.017 | 0.015 | 0.266 | 0.19980 |
| 1 | 27284913 | rs79598313 | C | T | 0.037 | 0.006 | 1.13180e-08 | 0.0001 | 32.601 | 0.026 | 0.036 | 0.473 | 0.02485 |
| 1 | 214150821 | rs79687284 | G | C | 0.030 | 0.005 | 3.77203e-08 | 0.0001 | 30.263 | -0.042 | 0.051 | 0.412 | 0.05070 |
| 14 | 39556185 | rs8018720 | G | C | 0.038 | 0.003 | 1.94818e-50 | 0.0004 | 223.056 | -0.009 | 0.019 | 0.627 | 0.82700 |
| 8 | 11611865 | rs804281 | A | G | -0.016 | 0.002 | 2.48467e-15 | 0.0001 | 62.637 | -0.026 | 0.014 | 0.070 | 0.56660 |
| 16 | 30883965 | rs8063565 | G | C | -0.012 | 0.002 | 2.68070e-08 | 0.0001 | 30.926 | -0.003 | 0.015 | 0.843 | 0.71370 |
| 18 | 28919794 | rs8091117 | C | A | 0.025 | 0.004 | 2.34349e-10 | 0.0001 | 40.157 | 0.009 | 0.025 | 0.711 | 0.09046 |
| 19 | 19388500 | rs8107974 | A | T | -0.039 | 0.004 | 1.48701e-25 | 0.0002 | 109.173 | 0.016 | 0.027 | 0.552 | 0.06859 |
| 20 | 52742306 | rs8121940 | C | G | 0.038 | 0.002 | 1.68777e-52 | 0.0004 | 232.514 | 0.015 | 0.016 | 0.322 | 0.24160 |
| 12 | 68661596 | rs8181687 | G | A | -0.012 | 0.002 | 3.99621e-09 | 0.0001 | 34.626 | 0.027 | 0.013 | 0.047 | 0.54270 |
| 9 | 125745042 | rs9409266 | G | A | 0.018 | 0.003 | 8.73653e-11 | 0.0001 | 42.085 | -0.030 | 0.018 | 0.086 | 0.85090 |
| 6 | 25653401 | rs9467550 | A | G | 0.019 | 0.003 | 3.35618e-09 | 0.0001 | 34.966 | -0.026 | 0.021 | 0.210 | 0.08151 |
| 6 | 57767576 | rs9476310 | C | T | -0.012 | 0.002 | 2.67337e-09 | 0.0001 | 35.409 | 0.009 | 0.013 | 0.517 | 0.48710 |
| 11 | 116648917 | rs964184 | G | C | -0.043 | 0.003 | 3.36693e-50 | 0.0004 | 221.967 | 0.022 | 0.019 | 0.248 | 0.83800 |

**Supplementary table 2.27. Detailed information of instrumental SNPs for C-reactive protein** **in the analysis of PPD.**

| **Chr** | **Position** | **SNP** | **EA** | **OA** | **beta.exposure** | **se.exposure** | **pval.exposure** | **R2** | **F_val** | **beta.outcome** | **se.outcome** | **pval.outcome** | **Eaf.outcome** |
| --- | --- | --- | --- | --- | --- | --- | --- | --- | --- | --- | --- | --- | --- |
| 8 | 116974302 | rs10095930 | T | C | 0.029 | 0.002 | 8.234e-45 | 0.0004 | 204.490 | 0.024 | 0.013 | 0.072 | 0.57360 |
| 8 | 103688381 | rs10106298 | A | G | 0.015 | 0.002 | 1.637e-12 | 0.0001 | 51.703 | -0.037 | 0.014 | 0.010 | 0.35390 |
| 17 | 72702914 | rs1037170 | T | C | 0.028 | 0.002 | 3.563e-33 | 0.0003 | 149.265 | 0.002 | 0.014 | 0.866 | 0.72660 |
| 2 | 174847693 | rs10497423 | T | G | -0.013 | 0.002 | 4.684e-11 | 0.0001 | 44.890 | -0.013 | 0.014 | 0.360 | 0.61030 |
| 2 | 230734531 | rs10498240 | A | C | 0.013 | 0.002 | 4.955e-10 | 0.0001 | 40.111 | -0.020 | 0.014 | 0.169 | 0.34390 |
| 1 | 156206121 | rs1052067 | A | G | -0.015 | 0.002 | 9.575e-11 | 0.0001 | 43.440 | 0.015 | 0.015 | 0.308 | 0.27730 |
| 20 | 62370349 | rs1056441 | T | C | -0.021 | 0.002 | 2.283e-22 | 0.0002 | 98.104 | -0.010 | 0.015 | 0.517 | 0.72270 |
| 9 | 102281383 | rs10760691 | A | G | -0.017 | 0.002 | 8.654e-16 | 0.0001 | 67.084 | -0.022 | 0.014 | 0.112 | 0.38470 |
| 11 | 11820449 | rs10831676 | A | C | 0.012 | 0.002 | 6.836e-09 | 0.0001 | 34.810 | 0.031 | 0.013 | 0.019 | 0.49400 |
| 1 | 214392881 | rs10864088 | A | G | -0.016 | 0.002 | 7.246e-14 | 0.0001 | 58.050 | -0.003 | 0.014 | 0.806 | 0.69280 |
| 9 | 71069835 | rs10868852 | A | C | 0.034 | 0.006 | 1.024e-08 | 0.0000 | 32.871 | 0.036 | 0.045 | 0.419 | 0.01789 |
| 18 | 57851763 | rs10871777 | A | G | -0.020 | 0.002 | 5.764e-17 | 0.0001 | 72.620 | 0.023 | 0.017 | 0.181 | 0.24650 |
| 1 | 66215348 | rs10889580 | T | C | -0.065 | 0.002 | 1.000e-200 | 0.0020 | 1053.003 | 0.012 | 0.014 | 0.384 | 0.59540 |
| 1 | 236312326 | rs10924372 | T | C | -0.015 | 0.002 | 6.185e-12 | 0.0001 | 49.000 | 0.003 | 0.014 | 0.855 | 0.63220 |
| 9 | 29717279 | rs10969334 | A | C | -0.011 | 0.002 | 3.793e-08 | 0.0001 | 31.360 | -0.011 | 0.013 | 0.399 | 0.41250 |
| 12 | 24195042 | rs11047224 | C | G | -0.052 | 0.005 | 3.903e-22 | 0.0002 | 93.444 | -0.004 | 0.048 | 0.930 | 0.04573 |
| 12 | 11791029 | rs11054402 | T | C | -0.012 | 0.002 | 3.790e-09 | 0.0001 | 36.000 | 0.013 | 0.014 | 0.351 | 0.46120 |
| 12 | 120146824 | rs11064880 | T | C | -0.042 | 0.007 | 1.195e-09 | 0.0001 | 37.228 | 0.008 | 0.043 | 0.847 | 0.01491 |
| 13 | 110384432 | rs11069802 | A | C | 0.014 | 0.002 | 3.790e-09 | 0.0001 | 36.000 | 0.050 | 0.016 | 0.002 | 0.22170 |
| 17 | 1618363 | rs11078597 | T | C | 0.019 | 0.003 | 1.616e-12 | 0.0001 | 51.729 | -0.013 | 0.017 | 0.434 | 0.16900 |
| 1 | 221103388 | rs11118625 | A | G | 0.019 | 0.002 | 4.672e-18 | 0.0001 | 77.760 | 0.012 | 0.015 | 0.420 | 0.26040 |
| 14 | 73974531 | rs11159021 | T | C | -0.012 | 0.002 | 3.410e-08 | 0.0001 | 31.574 | -0.004 | 0.013 | 0.764 | 0.37970 |
| 5 | 131777234 | rs11242113 | A | G | -0.015 | 0.002 | 6.080e-09 | 0.0001 | 35.046 | -0.038 | 0.016 | 0.016 | 0.20280 |
| 7 | 105658927 | rs114947103 | T | C | -0.016 | 0.003 | 3.013e-09 | 0.0001 | 36.463 | -0.011 | 0.015 | 0.460 | 0.21370 |
| 1 | 65811770 | rs115291616 | T | C | 0.029 | 0.004 | 7.315e-12 | 0.0001 | 46.370 | 0.004 | 0.028 | 0.884 | 0.05268 |
| 1 | 222061973 | rs11577023 | T | C | 0.015 | 0.002 | 4.450e-12 | 0.0001 | 49.669 | -0.005 | 0.014 | 0.706 | 0.35690 |
| 17 | 76344918 | rs11658216 | T | C | 0.027 | 0.002 | 3.508e-29 | 0.0003 | 130.340 | 0.021 | 0.015 | 0.152 | 0.73960 |
| 2 | 178193316 | rs11674864 | T | C | -0.017 | 0.002 | 4.638e-12 | 0.0001 | 49.585 | 0.021 | 0.018 | 0.223 | 0.80120 |
| 2 | 25130440 | rs11689543 | A | T | -0.019 | 0.002 | 6.580e-20 | 0.0002 | 86.490 | -0.007 | 0.014 | 0.584 | 0.42740 |
| 3 | 25363163 | rs11711864 | A | G | 0.011 | 0.002 | 2.867e-08 | 0.0001 | 31.922 | -0.022 | 0.013 | 0.098 | 0.58950 |
| 19 | 45287103 | rs117326714 | A | G | -0.048 | 0.007 | 2.431e-12 | 0.0001 | 49.413 | -0.002 | 0.064 | 0.972 | 0.02386 |
| 7 | 99181096 | rs11772470 | A | G | -0.027 | 0.003 | 1.006e-21 | 0.0002 | 88.620 | 0.004 | 0.016 | 0.810 | 0.15610 |
| 19 | 45699187 | rs117783785 | A | G | 0.055 | 0.010 | 3.879e-08 | 0.0001 | 30.087 | 0.000 | 0.047 | 0.997 | 0.01590 |
| 8 | 22452357 | rs11782130 | T | G | 0.012 | 0.002 | 8.777e-09 | 0.0001 | 34.306 | 0.014 | 0.015 | 0.325 | 0.34190 |
| 8 | 9239958 | rs11786900 | C | G | -0.037 | 0.004 | 2.144e-26 | 0.0002 | 110.550 | 0.015 | 0.020 | 0.440 | 0.07555 |
| 17 | 76357874 | rs11868378 | A | G | -0.033 | 0.003 | 5.735e-34 | 0.0004 | 153.026 | 0.006 | 0.016 | 0.684 | 0.78630 |
| 1 | 159692537 | rs12068753 | A | T | 0.220 | 0.004 | 1.000e-200 | 0.0064 | 2624.775 | 0.010 | 0.027 | 0.709 | 0.07157 |
| 1 | 21820042 | rs12132412 | A | G | -0.018 | 0.002 | 1.705e-17 | 0.0002 | 75.111 | 0.003 | 0.014 | 0.854 | 0.40160 |
| 1 | 159388286 | rs12134665 | T | G | -0.031 | 0.002 | 1.201e-44 | 0.0004 | 203.711 | -0.010 | 0.015 | 0.503 | 0.23360 |
| 6 | 136019147 | rs12202212 | T | C | 0.012 | 0.002 | 2.611e-08 | 0.0001 | 32.111 | -0.005 | 0.016 | 0.747 | 0.28730 |
| 11 | 30361670 | rs1222209 | A | C | -0.015 | 0.002 | 8.578e-12 | 0.0001 | 48.336 | 0.000 | 0.014 | 0.975 | 0.67000 |
| 12 | 95857690 | rs12231235 | A | G | -0.028 | 0.002 | 9.943e-43 | 0.0004 | 194.602 | 0.002 | 0.013 | 0.882 | 0.40760 |
| 10 | 21821918 | rs12251016 | A | T | -0.014 | 0.002 | 1.639e-11 | 0.0001 | 47.020 | 0.005 | 0.014 | 0.721 | 0.36280 |
| 19 | 807442 | rs123698 | C | G | -0.013 | 0.002 | 3.790e-09 | 0.0001 | 36.000 | -0.001 | 0.014 | 0.938 | 0.63520 |
| 2 | 232324510 | rs12620844 | T | C | -0.013 | 0.002 | 6.509e-11 | 0.0001 | 44.222 | -0.033 | 0.013 | 0.015 | 0.47220 |
| 7 | 22833400 | rs12673996 | T | C | -0.027 | 0.002 | 2.202e-28 | 0.0003 | 126.562 | 0.004 | 0.015 | 0.815 | 0.22560 |
| 16 | 29936654 | rs12716972 | A | G | -0.012 | 0.002 | 1.627e-08 | 0.0001 | 33.062 | 0.036 | 0.013 | 0.007 | 0.51790 |
| 1 | 159647656 | rs12744244 | A | C | 0.106 | 0.002 | 1.000e-200 | 0.0040 | 1965.444 | -0.003 | 0.016 | 0.861 | 0.22560 |
| 11 | 72506324 | rs12795307 | A | G | 0.017 | 0.002 | 5.686e-15 | 0.0001 | 63.240 | -0.003 | 0.015 | 0.863 | 0.28130 |
| 17 | 57911230 | rs1292061 | A | G | -0.021 | 0.002 | 1.025e-24 | 0.0002 | 109.202 | 0.034 | 0.013 | 0.010 | 0.56760 |
| 16 | 27325021 | rs12927172 | A | G | 0.019 | 0.002 | 2.589e-21 | 0.0002 | 93.122 | 0.007 | 0.014 | 0.598 | 0.62820 |
| 16 | 83983775 | rs12933677 | T | C | -0.014 | 0.002 | 1.720e-11 | 0.0001 | 46.922 | 0.012 | 0.013 | 0.391 | 0.47910 |
| 2 | 632591 | rs13028310 | T | C | -0.026 | 0.003 | 9.064e-23 | 0.0002 | 100.000 | -0.019 | 0.018 | 0.299 | 0.82800 |
| 3 | 94075026 | rs13066686 | A | C | -0.013 | 0.002 | 3.258e-10 | 0.0001 | 40.960 | 0.008 | 0.014 | 0.539 | 0.41150 |
| 6 | 35054185 | rs13216424 | A | G | 0.015 | 0.003 | 9.355e-09 | 0.0001 | 34.178 | -0.006 | 0.026 | 0.825 | 0.16800 |
| 8 | 126352397 | rs13261587 | T | C | 0.020 | 0.002 | 1.266e-23 | 0.0002 | 104.040 | -0.008 | 0.013 | 0.566 | 0.52190 |
| 10 | 91011458 | rs1332328 | T | C | 0.026 | 0.002 | 2.807e-35 | 0.0003 | 159.240 | -0.020 | 0.013 | 0.129 | 0.35980 |
| 2 | 59298298 | rs13416992 | A | C | 0.012 | 0.002 | 1.132e-09 | 0.0001 | 38.440 | 0.007 | 0.014 | 0.594 | 0.62030 |
| 1 | 65405070 | rs1353595 | A | C | -0.028 | 0.003 | 4.508e-23 | 0.0002 | 94.559 | -0.015 | 0.015 | 0.305 | 0.15410 |
| 6 | 127439897 | rs141783576 | C | G | -0.041 | 0.005 | 3.593e-19 | 0.0002 | 79.442 | -0.035 | 0.024 | 0.149 | 0.07058 |
| 4 | 103401723 | rs141936164 | A | G | 0.013 | 0.002 | 2.852e-09 | 0.0001 | 36.574 | 0.001 | 0.014 | 0.963 | 0.33900 |
| 2 | 214033530 | rs1441169 | A | G | 0.021 | 0.002 | 6.157e-25 | 0.0002 | 110.250 | -0.009 | 0.014 | 0.501 | 0.53480 |
| 19 | 45629104 | rs145903417 | A | G | 0.056 | 0.008 | 1.678e-11 | 0.0001 | 45.848 | -0.036 | 0.045 | 0.426 | 0.01988 |
| 14 | 35621393 | rs146424514 | A | G | 0.016 | 0.003 | 7.295e-09 | 0.0001 | 34.679 | 0.004 | 0.017 | 0.796 | 0.16100 |
| 3 | 35670150 | rs1470560 | A | G | 0.012 | 0.002 | 1.520e-08 | 0.0001 | 33.200 | 0.026 | 0.014 | 0.062 | 0.35980 |
| 19 | 45337918 | rs147711004 | A | G | -0.212 | 0.006 | 1.000e-200 | 0.0025 | 1379.406 | 0.036 | 0.033 | 0.280 | 0.02883 |
| 6 | 126851160 | rs1490384 | T | C | -0.028 | 0.002 | 9.943e-43 | 0.0004 | 194.602 | 0.001 | 0.013 | 0.921 | 0.49300 |
| 3 | 170705693 | rs1514895 | A | G | -0.020 | 0.002 | 2.126e-18 | 0.0002 | 79.372 | 0.007 | 0.015 | 0.639 | 0.28930 |
| 8 | 144643169 | rs1545536 | T | C | -0.024 | 0.002 | 9.143e-22 | 0.0002 | 95.258 | -0.020 | 0.017 | 0.237 | 0.20580 |
| 17 | 36034365 | rs1557812 | A | G | 0.015 | 0.002 | 3.814e-10 | 0.0001 | 40.641 | -0.014 | 0.017 | 0.408 | 0.22270 |
| 2 | 27412283 | rs1659681 | A | G | -0.043 | 0.004 | 6.974e-25 | 0.0002 | 104.819 | -0.021 | 0.031 | 0.495 | 0.93640 |
| 5 | 56194768 | rs166169 | T | C | -0.019 | 0.003 | 4.262e-11 | 0.0001 | 42.026 | -0.008 | 0.018 | 0.659 | 0.84390 |
| 19 | 35553341 | rs1688043 | T | C | -0.030 | 0.004 | 6.931e-14 | 0.0001 | 55.339 | 0.027 | 0.028 | 0.331 | 0.93840 |
| 21 | 33122575 | rs16988471 | A | C | -0.034 | 0.005 | 6.185e-12 | 0.0001 | 47.020 | -0.008 | 0.024 | 0.745 | 0.05268 |
| 12 | 63455347 | rs17098829 | C | G | -0.013 | 0.002 | 3.613e-08 | 0.0001 | 31.457 | 0.000 | 0.015 | 0.973 | 0.29130 |
| 17 | 36073320 | rs17138478 | A | C | 0.034 | 0.003 | 9.234e-27 | 0.0002 | 111.566 | 0.000 | 0.018 | 0.999 | 0.11330 |
| 7 | 6418378 | rs17196595 | T | C | -0.014 | 0.002 | 1.731e-09 | 0.0001 | 37.582 | 0.011 | 0.016 | 0.491 | 0.22270 |
| 1 | 78623626 | rs17391694 | T | C | 0.017 | 0.003 | 1.479e-08 | 0.0001 | 31.144 | -0.003 | 0.020 | 0.873 | 0.09642 |
| 1 | 205216110 | rs17417252 | A | G | -0.015 | 0.002 | 2.131e-11 | 0.0001 | 46.488 | 0.011 | 0.014 | 0.451 | 0.28230 |
| 6 | 111904528 | rs174373 | T | C | 0.014 | 0.002 | 3.299e-08 | 0.0001 | 31.641 | -0.018 | 0.015 | 0.238 | 0.78930 |
| 16 | 51436882 | rs17616063 | A | G | 0.134 | 0.004 | 1.000e-200 | 0.0026 | 924.713 | 0.008 | 0.019 | 0.661 | 0.07853 |
| 14 | 73366493 | rs17781691 | A | G | 0.019 | 0.002 | 2.642e-20 | 0.0002 | 88.360 | 0.015 | 0.013 | 0.259 | 0.50600 |
| 17 | 16083684 | rs178826 | T | C | 0.016 | 0.002 | 8.541e-15 | 0.0001 | 62.410 | 0.023 | 0.014 | 0.085 | 0.56560 |
| 12 | 6440009 | rs1800693 | T | C | 0.022 | 0.002 | 9.556e-27 | 0.0002 | 118.810 | 0.009 | 0.013 | 0.505 | 0.42540 |
| 20 | 43042364 | rs1800961 | T | C | -0.107 | 0.006 | 6.495e-74 | 0.0008 | 331.363 | 0.000 | 0.032 | 0.997 | 0.03678 |
| 12 | 69744014 | rs1800973 | A | C | 0.029 | 0.004 | 6.185e-12 | 0.0001 | 46.747 | 0.003 | 0.031 | 0.930 | 0.04374 |
| 7 | 22759469 | rs1880241 | A | G | 0.025 | 0.002 | 3.544e-35 | 0.0003 | 158.760 | 0.008 | 0.014 | 0.575 | 0.48110 |
| 7 | 115906373 | rs1918912 | T | C | -0.012 | 0.002 | 3.095e-08 | 0.0001 | 31.769 | -0.010 | 0.014 | 0.459 | 0.30220 |
| 3 | 156915895 | rs1969066 | A | G | 0.013 | 0.002 | 2.889e-09 | 0.0001 | 36.548 | 0.017 | 0.016 | 0.289 | 0.30820 |
| 11 | 16251251 | rs2030291 | A | T | 0.013 | 0.002 | 2.372e-10 | 0.0001 | 41.602 | 0.012 | 0.014 | 0.405 | 0.41650 |
| 2 | 102756295 | rs2041748 | A | G | 0.022 | 0.002 | 1.683e-21 | 0.0002 | 94.006 | 0.039 | 0.020 | 0.049 | 0.75150 |
| 3 | 4769176 | rs2054872 | A | C | 0.018 | 0.003 | 1.940e-10 | 0.0001 | 42.010 | -0.027 | 0.020 | 0.178 | 0.14910 |
| 12 | 121431272 | rs2071190 | A | T | 0.050 | 0.002 | 3.740e-101 | 0.0009 | 472.590 | -0.023 | 0.016 | 0.150 | 0.23560 |
| 12 | 121600953 | rs208296 | A | G | 0.035 | 0.002 | 3.943e-54 | 0.0005 | 248.779 | -0.025 | 0.014 | 0.086 | 0.32410 |
| 12 | 21701300 | rs2126886 | T | C | -0.017 | 0.002 | 1.936e-12 | 0.0001 | 51.361 | 0.019 | 0.017 | 0.258 | 0.76940 |
| 2 | 169893419 | rs2161037 | A | G | 0.020 | 0.002 | 7.697e-24 | 0.0002 | 105.062 | 0.008 | 0.013 | 0.555 | 0.53280 |
| 5 | 172176886 | rs2161374 | T | C | -0.019 | 0.002 | 6.604e-21 | 0.0002 | 91.202 | -0.004 | 0.013 | 0.747 | 0.47810 |
| 20 | 39142516 | rs2207132 | A | G | 0.049 | 0.006 | 5.180e-16 | 0.0002 | 65.880 | 0.008 | 0.027 | 0.779 | 0.04076 |
| 14 | 73011885 | rs2239222 | A | G | -0.036 | 0.002 | 2.853e-63 | 0.0006 | 292.247 | -0.019 | 0.014 | 0.173 | 0.35390 |
| 2 | 37086197 | rs2245109 | C | G | 0.014 | 0.002 | 4.378e-12 | 0.0001 | 49.702 | 0.028 | 0.013 | 0.035 | 0.52090 |
| 20 | 25262789 | rs2261790 | T | C | -0.018 | 0.002 | 2.301e-18 | 0.0002 | 79.210 | 0.012 | 0.013 | 0.381 | 0.43340 |
| 3 | 49941436 | rs2280406 | A | G | 0.022 | 0.002 | 1.124e-27 | 0.0002 | 123.210 | 0.053 | 0.014 | 0.000 | 0.49110 |
| 5 | 156738540 | rs2289852 | A | G | -0.031 | 0.004 | 8.715e-13 | 0.0001 | 50.604 | -0.003 | 0.020 | 0.872 | 0.06262 |
| 6 | 88367635 | rs2307377 | A | G | -0.022 | 0.004 | 4.449e-09 | 0.0001 | 33.823 | -0.023 | 0.022 | 0.300 | 0.08847 |
| 3 | 131998851 | rs2310227 | T | G | -0.012 | 0.002 | 2.611e-08 | 0.0001 | 32.111 | -0.015 | 0.013 | 0.252 | 0.52680 |
| 18 | 23797853 | rs2339102 | T | C | 0.013 | 0.002 | 8.321e-10 | 0.0001 | 39.062 | -0.002 | 0.013 | 0.881 | 0.48810 |
| 12 | 47186260 | rs2465585 | A | G | 0.022 | 0.002 | 1.363e-18 | 0.0002 | 80.282 | -0.011 | 0.017 | 0.528 | 0.20080 |
| 18 | 12800820 | rs2542170 | T | C | -0.024 | 0.002 | 2.613e-31 | 0.0003 | 140.423 | -0.003 | 0.014 | 0.831 | 0.57160 |
| 3 | 9480906 | rs2596937 | T | C | -0.017 | 0.002 | 1.442e-12 | 0.0001 | 51.960 | 0.018 | 0.018 | 0.328 | 0.17690 |
| 12 | 56929694 | rs2657896 | T | C | -0.023 | 0.003 | 2.332e-17 | 0.0002 | 74.471 | 0.012 | 0.019 | 0.516 | 0.17890 |
| 7 | 36085142 | rs2700938 | T | C | -0.024 | 0.002 | 2.508e-28 | 0.0003 | 126.295 | 0.005 | 0.014 | 0.712 | 0.38170 |
| 16 | 28338039 | rs2726033 | A | G | -0.015 | 0.002 | 1.569e-11 | 0.0001 | 47.110 | 0.012 | 0.013 | 0.351 | 0.36780 |
| 16 | 53798319 | rs28429148 | A | G | 0.025 | 0.002 | 7.179e-31 | 0.0003 | 138.342 | 0.003 | 0.013 | 0.808 | 0.46020 |
| 5 | 107348180 | rs288183 | T | G | 0.015 | 0.003 | 2.259e-08 | 0.0001 | 32.402 | -0.005 | 0.017 | 0.796 | 0.16700 |
| 14 | 94844947 | rs28929474 | T | C | -0.100 | 0.007 | 1.674e-41 | 0.0003 | 183.712 | -0.007 | 0.048 | 0.891 | 0.01690 |
| 1 | 8488565 | rs301798 | A | G | -0.012 | 0.002 | 4.445e-08 | 0.0001 | 31.041 | 0.008 | 0.014 | 0.585 | 0.31410 |
| 10 | 30708441 | rs303429 | T | C | -0.015 | 0.002 | 8.354e-14 | 0.0001 | 57.760 | 0.000 | 0.013 | 0.995 | 0.59740 |
| 15 | 60883281 | rs339969 | A | C | 0.034 | 0.002 | 1.292e-56 | 0.0005 | 260.592 | 0.019 | 0.015 | 0.201 | 0.60830 |
| 1 | 247588053 | rs34298354 | T | C | -0.028 | 0.003 | 8.901e-20 | 0.0002 | 80.420 | 0.029 | 0.022 | 0.184 | 0.11430 |
| 1 | 22681214 | rs34761529 | T | C | -0.018 | 0.002 | 2.029e-12 | 0.0001 | 51.266 | 0.007 | 0.019 | 0.728 | 0.19090 |
| 8 | 129174624 | rs34820057 | A | G | -0.014 | 0.002 | 3.299e-08 | 0.0001 | 31.641 | -0.032 | 0.017 | 0.059 | 0.20870 |
| 18 | 13737452 | rs34866669 | C | G | 0.014 | 0.002 | 1.478e-10 | 0.0001 | 42.560 | -0.009 | 0.014 | 0.525 | 0.33300 |
| 7 | 150294632 | rs35462231 | A | T | 0.021 | 0.002 | 4.100e-18 | 0.0002 | 78.028 | -0.002 | 0.014 | 0.890 | 0.21570 |
| 3 | 119529113 | rs3732356 | T | G | -0.024 | 0.004 | 1.806e-08 | 0.0001 | 31.307 | 0.023 | 0.026 | 0.382 | 0.94230 |
| 1 | 40035928 | rs3768321 | T | G | 0.034 | 0.002 | 3.057e-40 | 0.0003 | 182.790 | 0.033 | 0.018 | 0.066 | 0.18790 |
| 7 | 101849396 | rs378011 | A | G | 0.014 | 0.002 | 1.639e-11 | 0.0001 | 47.020 | -0.004 | 0.014 | 0.793 | 0.67300 |
| 5 | 150435480 | rs3805433 | C | G | -0.015 | 0.002 | 1.569e-11 | 0.0001 | 47.110 | 0.018 | 0.015 | 0.234 | 0.25450 |
| 7 | 1028448 | rs3808348 | T | C | -0.022 | 0.002 | 1.363e-18 | 0.0002 | 80.282 | -0.024 | 0.015 | 0.105 | 0.21470 |
| 19 | 51727962 | rs3865444 | A | C | -0.020 | 0.002 | 8.426e-21 | 0.0002 | 90.703 | -0.007 | 0.014 | 0.624 | 0.31010 |
| 1 | 1564194 | rs3935032 | T | C | -0.015 | 0.002 | 6.417e-11 | 0.0001 | 44.251 | 0.017 | 0.014 | 0.231 | 0.35790 |
| 16 | 2169458 | rs4018180 | A | G | -0.032 | 0.004 | 3.102e-14 | 0.0001 | 56.967 | -0.008 | 0.021 | 0.707 | 0.07157 |
| 1 | 154395212 | rs4133213 | A | C | -0.085 | 0.002 | 1.000e-200 | 0.0034 | 1802.003 | 0.016 | 0.014 | 0.270 | 0.39260 |
| 4 | 89054667 | rs4148155 | A | G | 0.019 | 0.003 | 5.485e-09 | 0.0001 | 33.150 | 0.029 | 0.025 | 0.251 | 0.09245 |
| 9 | 14442595 | rs424539 | C | G | -0.012 | 0.002 | 1.994e-08 | 0.0001 | 32.653 | -0.004 | 0.014 | 0.770 | 0.41150 |
| 2 | 88438226 | rs4281907 | A | C | 0.013 | 0.002 | 4.684e-11 | 0.0001 | 44.890 | -0.027 | 0.014 | 0.052 | 0.47710 |
| 6 | 116327936 | rs4354188 | T | C | 0.018 | 0.002 | 9.599e-19 | 0.0002 | 81.000 | 0.007 | 0.014 | 0.603 | 0.40850 |
| 11 | 47245389 | rs4647725 | T | C | 0.033 | 0.003 | 8.943e-34 | 0.0003 | 152.111 | 0.026 | 0.015 | 0.084 | 0.15410 |
| 1 | 65932185 | rs4655598 | A | T | 0.062 | 0.002 | 1.000e-200 | 0.0019 | 951.722 | 0.010 | 0.013 | 0.447 | 0.54170 |
| 4 | 3447156 | rs4690098 | T | C | 0.017 | 0.002 | 8.236e-12 | 0.0001 | 48.418 | 0.014 | 0.015 | 0.362 | 0.23460 |
| 1 | 91530432 | rs469882 | A | C | 0.035 | 0.002 | 8.684e-43 | 0.0004 | 194.882 | 0.011 | 0.016 | 0.498 | 0.18990 |
| 6 | 41671677 | rs4714508 | A | G | -0.018 | 0.002 | 5.666e-17 | 0.0001 | 72.655 | -0.009 | 0.014 | 0.522 | 0.34490 |
| 11 | 43642130 | rs4755726 | T | G | 0.017 | 0.002 | 8.939e-14 | 0.0001 | 57.622 | 0.006 | 0.014 | 0.652 | 0.66300 |
| 12 | 103522952 | rs4764939 | T | C | -0.020 | 0.002 | 5.562e-23 | 0.0002 | 101.002 | 0.015 | 0.014 | 0.262 | 0.50600 |
| 15 | 53741628 | rs4776150 | A | G | -0.018 | 0.002 | 3.966e-19 | 0.0002 | 82.810 | -0.005 | 0.013 | 0.691 | 0.46920 |
| 21 | 40465066 | rs4817984 | A | C | -0.034 | 0.002 | 3.139e-53 | 0.0005 | 244.496 | 0.009 | 0.015 | 0.554 | 0.26940 |
| 22 | 38599364 | rs4821764 | A | G | -0.013 | 0.002 | 9.025e-11 | 0.0001 | 43.560 | 0.012 | 0.013 | 0.359 | 0.57550 |
| 4 | 38210173 | rs4832754 | T | C | 0.013 | 0.002 | 1.852e-08 | 0.0001 | 32.802 | 0.013 | 0.014 | 0.336 | 0.70480 |
| 2 | 113829236 | rs4849147 | A | T | -0.017 | 0.002 | 4.523e-14 | 0.0001 | 59.010 | 0.002 | 0.016 | 0.912 | 0.70680 |
| 4 | 55328931 | rs4864892 | T | G | 0.012 | 0.002 | 2.611e-08 | 0.0001 | 32.111 | 0.027 | 0.015 | 0.074 | 0.31010 |
| 12 | 24026288 | rs4963725 | T | C | 0.016 | 0.003 | 3.379e-08 | 0.0001 | 29.521 | -0.020 | 0.019 | 0.310 | 0.13320 |
| 16 | 1129010 | rs4988483 | A | C | 0.028 | 0.005 | 4.736e-08 | 0.0001 | 29.713 | -0.012 | 0.051 | 0.821 | 0.06461 |
| 6 | 18492350 | rs5017416 | T | G | 0.027 | 0.005 | 1.051e-08 | 0.0001 | 32.514 | 0.023 | 0.027 | 0.382 | 0.05865 |
| 18 | 55089715 | rs55855238 | T | C | -0.027 | 0.002 | 1.478e-36 | 0.0003 | 165.306 | 0.000 | 0.014 | 0.979 | 0.66800 |
| 2 | 72500685 | rs56045434 | C | G | -0.016 | 0.003 | 1.627e-08 | 0.0001 | 30.822 | -0.007 | 0.017 | 0.661 | 0.14020 |
| 14 | 73209388 | rs56047269 | T | C | 0.014 | 0.002 | 4.097e-10 | 0.0001 | 40.496 | -0.001 | 0.018 | 0.962 | 0.26740 |
| 15 | 63789479 | rs56187480 | A | G | -0.016 | 0.002 | 2.959e-13 | 0.0001 | 55.184 | 0.038 | 0.014 | 0.005 | 0.39660 |
| 5 | 92611208 | rs56821385 | T | C | -0.038 | 0.007 | 3.856e-08 | 0.0001 | 30.412 | -0.009 | 0.045 | 0.844 | 0.02783 |
| 1 | 10559598 | rs585406 | T | C | 0.012 | 0.002 | 1.222e-08 | 0.0001 | 33.640 | 0.008 | 0.013 | 0.528 | 0.50990 |
| 19 | 19379549 | rs58542926 | T | C | 0.036 | 0.004 | 3.528e-20 | 0.0002 | 83.324 | -0.014 | 0.027 | 0.602 | 0.06759 |
| 1 | 247601778 | rs58546652 | T | C | 0.040 | 0.002 | 1.340e-87 | 0.0007 | 408.040 | 0.024 | 0.014 | 0.079 | 0.64610 |
| 19 | 2671100 | rs59737437 | T | C | -0.015 | 0.002 | 1.498e-10 | 0.0001 | 42.533 | 0.010 | 0.014 | 0.498 | 0.23860 |
| 16 | 88528636 | rs60037105 | A | T | 0.018 | 0.002 | 3.084e-16 | 0.0001 | 69.192 | -0.014 | 0.014 | 0.337 | 0.30320 |
| 12 | 122022382 | rs60186830 | A | G | -0.041 | 0.006 | 2.325e-10 | 0.0002 | 40.371 | -0.031 | 0.028 | 0.271 | 0.04771 |
| 20 | 57295129 | rs6070563 | A | G | -0.013 | 0.002 | 1.605e-09 | 0.0001 | 37.735 | 0.003 | 0.014 | 0.836 | 0.61830 |
| 20 | 62692060 | rs6090040 | A | C | 0.015 | 0.002 | 3.944e-14 | 0.0001 | 59.290 | -0.003 | 0.013 | 0.828 | 0.52580 |
| 2 | 466003 | rs62104180 | A | G | -0.032 | 0.005 | 1.151e-10 | 0.0001 | 41.327 | 0.009 | 0.050 | 0.862 | 0.04771 |
| 7 | 36178374 | rs62451586 | A | G | -0.020 | 0.003 | 8.321e-10 | 0.0001 | 36.731 | -0.016 | 0.020 | 0.406 | 0.09841 |
| 11 | 32956492 | rs62618693 | T | C | -0.030 | 0.005 | 6.080e-09 | 0.0001 | 33.686 | -0.007 | 0.043 | 0.865 | 0.03380 |
| 1 | 177885762 | rs630372 | A | G | 0.015 | 0.002 | 4.820e-11 | 0.0001 | 44.832 | -0.007 | 0.017 | 0.683 | 0.22270 |
| 2 | 203425475 | rs6435156 | T | C | 0.014 | 0.002 | 4.903e-09 | 0.0001 | 35.480 | -0.008 | 0.014 | 0.585 | 0.25650 |
| 3 | 176869498 | rs6443429 | A | C | 0.013 | 0.002 | 3.674e-10 | 0.0001 | 40.717 | -0.019 | 0.015 | 0.200 | 0.69780 |
| 11 | 13361524 | rs6486122 | T | C | 0.030 | 0.002 | 1.380e-43 | 0.0004 | 198.676 | 0.026 | 0.013 | 0.048 | 0.70380 |
| 16 | 89848559 | rs6500446 | A | G | 0.011 | 0.002 | 2.163e-08 | 0.0001 | 32.490 | -0.016 | 0.013 | 0.236 | 0.41050 |
| 22 | 39096602 | rs6519133 | T | C | 0.031 | 0.002 | 1.578e-48 | 0.0005 | 222.152 | 0.001 | 0.014 | 0.959 | 0.40460 |
| 1 | 112328245 | rs653170 | T | C | 0.012 | 0.002 | 1.537e-09 | 0.0001 | 37.822 | 0.001 | 0.013 | 0.927 | 0.38770 |
| 6 | 138185422 | rs654912 | T | C | 0.013 | 0.002 | 6.481e-09 | 0.0001 | 34.917 | 0.014 | 0.014 | 0.304 | 0.70680 |
| 12 | 46416742 | rs6582586 | A | G | 0.011 | 0.002 | 2.163e-08 | 0.0001 | 32.490 | -0.012 | 0.013 | 0.359 | 0.41450 |
| 5 | 124139761 | rs6595549 | C | G | -0.016 | 0.003 | 4.760e-09 | 0.0001 | 35.540 | 0.012 | 0.017 | 0.470 | 0.17690 |
| 1 | 198442821 | rs6686560 | A | G | 0.026 | 0.003 | 3.919e-15 | 0.0002 | 60.180 | 0.041 | 0.018 | 0.019 | 0.13520 |
| 2 | 113841030 | rs6734238 | A | G | -0.045 | 0.002 | 9.530e-108 | 0.0010 | 504.002 | -0.025 | 0.014 | 0.082 | 0.41250 |
| 2 | 27752871 | rs6753534 | T | C | -0.062 | 0.002 | 1.000e-200 | 0.0019 | 951.722 | 0.015 | 0.014 | 0.272 | 0.55570 |
| 3 | 18758501 | rs6775319 | A | T | -0.013 | 0.002 | 4.961e-09 | 0.0001 | 35.457 | 0.036 | 0.014 | 0.011 | 0.70080 |
| 12 | 121565950 | rs67881993 | T | G | -0.035 | 0.005 | 9.207e-14 | 0.0001 | 55.139 | 0.035 | 0.045 | 0.434 | 0.04771 |
| 3 | 24520283 | rs6792725 | A | G | 0.018 | 0.002 | 1.465e-16 | 0.0002 | 70.713 | 0.015 | 0.013 | 0.253 | 0.67100 |
| 3 | 71632266 | rs6801781 | A | G | 0.013 | 0.002 | 8.452e-09 | 0.0001 | 34.382 | 0.007 | 0.015 | 0.617 | 0.72170 |
| 8 | 9918062 | rs685218 | A | C | 0.014 | 0.002 | 1.478e-10 | 0.0001 | 42.560 | 0.016 | 0.014 | 0.247 | 0.30120 |
| 3 | 135932359 | rs687339 | T | C | -0.025 | 0.002 | 2.193e-24 | 0.0002 | 107.641 | -0.037 | 0.019 | 0.047 | 0.77040 |
| 6 | 98411631 | rs6905544 | A | G | -0.015 | 0.002 | 1.753e-13 | 0.0001 | 56.250 | -0.009 | 0.014 | 0.504 | 0.59050 |
| 6 | 138006504 | rs6920220 | A | G | 0.022 | 0.002 | 3.175e-19 | 0.0001 | 83.266 | 0.019 | 0.017 | 0.260 | 0.16900 |
| 6 | 7112819 | rs6925389 | A | G | -0.015 | 0.002 | 3.195e-12 | 0.0001 | 50.342 | -0.010 | 0.013 | 0.466 | 0.62230 |
| 8 | 73435156 | rs6993128 | T | C | -0.014 | 0.002 | 1.670e-09 | 0.0001 | 37.655 | -0.003 | 0.015 | 0.834 | 0.72070 |
| 8 | 9173209 | rs7012637 | A | G | 0.050 | 0.002 | 1.290e-132 | 0.0012 | 622.503 | 0.002 | 0.013 | 0.852 | 0.47320 |
| 9 | 104111638 | rs7026000 | T | C | -0.020 | 0.003 | 5.903e-13 | 0.0001 | 53.778 | -0.015 | 0.022 | 0.494 | 0.15900 |
| 10 | 80819132 | rs704017 | A | G | 0.016 | 0.002 | 3.919e-15 | 0.0001 | 64.000 | -0.008 | 0.014 | 0.585 | 0.56160 |
| 10 | 133736636 | rs7084062 | A | G | -0.013 | 0.002 | 4.955e-10 | 0.0001 | 40.111 | 0.027 | 0.014 | 0.043 | 0.55860 |
| 11 | 27700125 | rs7103411 | T | C | 0.022 | 0.002 | 9.599e-19 | 0.0002 | 81.000 | -0.038 | 0.018 | 0.030 | 0.76540 |
| 17 | 76285388 | rs7217663 | T | C | -0.021 | 0.004 | 7.598e-10 | 0.0001 | 37.036 | -0.032 | 0.023 | 0.166 | 0.11630 |
| 5 | 132291157 | rs72799498 | A | G | 0.017 | 0.003 | 1.554e-09 | 0.0001 | 37.800 | -0.024 | 0.019 | 0.213 | 0.14510 |
| 2 | 172034785 | rs728455 | T | C | 0.014 | 0.002 | 2.007e-10 | 0.0001 | 41.941 | -0.031 | 0.014 | 0.032 | 0.36780 |
| 16 | 51205819 | rs728538 | T | G | -0.040 | 0.003 | 2.824e-47 | 0.0005 | 216.199 | -0.029 | 0.017 | 0.081 | 0.19680 |
| 12 | 90429028 | rs7311631 | A | G | 0.015 | 0.002 | 3.944e-14 | 0.0001 | 59.290 | -0.017 | 0.013 | 0.203 | 0.51190 |
| 7 | 74073590 | rs73137144 | A | G | 0.019 | 0.003 | 2.119e-12 | 0.0001 | 51.178 | -0.016 | 0.019 | 0.389 | 0.16800 |
| 7 | 150855952 | rs73476799 | A | G | -0.041 | 0.007 | 6.287e-09 | 0.0001 | 34.000 | 0.000 | 0.046 | 0.994 | 0.03479 |
| 9 | 92207308 | rs7357754 | A | G | -0.018 | 0.002 | 3.550e-18 | 0.0002 | 78.323 | -0.009 | 0.013 | 0.503 | 0.51590 |
| 19 | 45412079 | rs7412 | T | C | 0.091 | 0.004 | 8.440e-122 | 0.0010 | 542.054 | -0.026 | 0.030 | 0.373 | 0.06262 |
| 20 | 44615889 | rs74616372 | T | C | 0.023 | 0.004 | 1.572e-10 | 0.0001 | 40.111 | 0.038 | 0.027 | 0.153 | 0.09841 |
| 5 | 72979436 | rs750344 | A | G | 0.016 | 0.002 | 1.740e-12 | 0.0001 | 51.579 | -0.004 | 0.016 | 0.780 | 0.70780 |
| 7 | 72895946 | rs75229905 | T | C | 0.037 | 0.003 | 4.016e-34 | 0.0003 | 144.000 | 0.029 | 0.020 | 0.141 | 0.11430 |
| 1 | 109817192 | rs7528419 | A | G | -0.016 | 0.002 | 7.667e-11 | 0.0001 | 43.891 | 0.017 | 0.016 | 0.295 | 0.21270 |
| 1 | 27180088 | rs75460349 | A | C | 0.095 | 0.007 | 3.401e-45 | 0.0004 | 200.201 | 0.017 | 0.036 | 0.624 | 0.02485 |
| 1 | 63034363 | rs7555577 | A | C | -0.013 | 0.002 | 4.955e-10 | 0.0001 | 40.111 | 0.024 | 0.015 | 0.107 | 0.70380 |
| 2 | 102300940 | rs7594775 | T | C | -0.015 | 0.002 | 6.185e-12 | 0.0001 | 49.000 | 0.009 | 0.015 | 0.563 | 0.34990 |
| 14 | 21750929 | rs76014628 | T | C | -0.014 | 0.002 | 6.200e-09 | 0.0001 | 35.007 | -0.020 | 0.015 | 0.189 | 0.20680 |
| 4 | 45121873 | rs7662792 | A | T | -0.017 | 0.002 | 8.232e-15 | 0.0001 | 62.485 | -0.021 | 0.014 | 0.150 | 0.68090 |
| 2 | 113978183 | rs77143791 | A | G | 0.033 | 0.005 | 9.025e-11 | 0.0001 | 41.869 | -0.006 | 0.023 | 0.806 | 0.05268 |
| 15 | 53142829 | rs77405882 | T | G | 0.029 | 0.003 | 4.672e-18 | 0.0001 | 73.253 | -0.012 | 0.029 | 0.666 | 0.08250 |
| 6 | 153367347 | rs7763717 | T | G | -0.016 | 0.002 | 3.410e-13 | 0.0001 | 54.895 | 0.009 | 0.014 | 0.504 | 0.29320 |
| 5 | 52080909 | rs77704739 | T | C | 0.048 | 0.005 | 1.380e-20 | 0.0001 | 86.276 | -0.001 | 0.038 | 0.982 | 0.02883 |
| 12 | 100926084 | rs77738620 | T | C | -0.059 | 0.010 | 2.619e-09 | 0.0001 | 35.276 | 0.003 | 0.043 | 0.948 | 0.01093 |
| 7 | 128573994 | rs7801838 | T | C | -0.013 | 0.002 | 1.100e-08 | 0.0001 | 33.851 | 0.002 | 0.014 | 0.893 | 0.31010 |
| 9 | 139281847 | rs78428995 | T | C | -0.020 | 0.002 | 2.079e-23 | 0.0002 | 103.022 | -0.027 | 0.013 | 0.048 | 0.39360 |
| 1 | 91414243 | rs78511209 | C | G | 0.021 | 0.004 | 7.598e-10 | 0.0001 | 37.036 | -0.001 | 0.030 | 0.976 | 0.10240 |
| 10 | 75554541 | rs7908825 | C | G | -0.013 | 0.002 | 6.509e-11 | 0.0001 | 44.222 | 0.003 | 0.013 | 0.832 | 0.62520 |
| 11 | 59936926 | rs7933202 | A | C | 0.021 | 0.002 | 6.157e-25 | 0.0002 | 110.250 | 0.000 | 0.015 | 0.974 | 0.39360 |
| 1 | 93716974 | rs797680 | T | G | -0.014 | 0.002 | 1.530e-12 | 0.0001 | 51.840 | -0.002 | 0.014 | 0.896 | 0.36380 |
| 13 | 42573980 | rs7993752 | A | C | -0.013 | 0.002 | 4.684e-11 | 0.0001 | 44.890 | -0.004 | 0.014 | 0.781 | 0.57060 |
| 7 | 22876582 | rs79941637 | A | T | 0.029 | 0.004 | 7.118e-11 | 0.0001 | 42.106 | 0.011 | 0.034 | 0.742 | 0.06064 |
| 13 | 113927208 | rs9604045 | T | G | -0.023 | 0.003 | 8.057e-17 | 0.0002 | 71.936 | 0.023 | 0.018 | 0.197 | 0.25650 |
| 22 | 41339367 | rs9611441 | C | G | -0.016 | 0.002 | 8.541e-15 | 0.0001 | 62.410 | 0.033 | 0.013 | 0.012 | 0.47320 |
| 12 | 31997635 | rs9738365 | A | C | 0.021 | 0.002 | 1.388e-19 | 0.0002 | 84.960 | 0.014 | 0.015 | 0.355 | 0.30720 |
| 15 | 78802869 | rs9788721 | T | C | -0.012 | 0.002 | 1.520e-08 | 0.0001 | 33.200 | -0.009 | 0.014 | 0.507 | 0.61230 |
| 6 | 166329862 | rs991946 | T | C | 0.011 | 0.002 | 3.793e-08 | 0.0001 | 31.360 | 0.024 | 0.013 | 0.068 | 0.47020 |
| 17 | 68258208 | rs992072 | T | G | -0.015 | 0.002 | 3.910e-11 | 0.0001 | 45.256 | 0.018 | 0.016 | 0.253 | 0.67890 |
| 4 | 18459828 | rs994596 | T | C | 0.014 | 0.002 | 1.478e-10 | 0.0001 | 42.560 | -0.012 | 0.015 | 0.441 | 0.31810 |
| 18 | 40776606 | rs9965184 | A | T | 0.012 | 0.002 | 3.410e-08 | 0.0001 | 31.574 | 0.007 | 0.013 | 0.581 | 0.35190 |
| 21 | 46494995 | rs9977825 | T | C | 0.012 | 0.002 | 1.156e-08 | 0.0001 | 33.751 | 0.024 | 0.014 | 0.097 | 0.64310 |

**Supplementary table 2.28. Detailed information of instrumental SNPs for SI in the analysis of PPD.**

| **Chr** | **Position** | **SNP** | **EA** | **OA** | **beta.exposure** | **se.exposure** | **pval.exposure** | **R2** | **F_val** | **beta.outcome** | **se.outcome** | **pval.outcome** | **Eaf.outcome** |
| --- | --- | --- | --- | --- | --- | --- | --- | --- | --- | --- | --- | --- | --- |
| 4 | 147797214 | rs10001365 | A | G | -0.025 | 0.004 | 6.65e-12 | 3e-04 | 47.1 | -0.021 | 0.013 | 0.126 | 0.37870 |
| 2 | 45159091 | rs1004787 | A | G | 0.030 | 0.004 | 5.27e-17 | 4e-04 | 70.2 | 0.017 | 0.014 | 0.206 | 0.53980 |
| 10 | 21766969 | rs10159545 | G | C | 0.026 | 0.004 | 1.84e-12 | 3e-04 | 49.6 | 0.008 | 0.014 | 0.569 | 0.38170 |
| 7 | 117523709 | rs10233018 | G | A | 0.027 | 0.004 | 2.75e-14 | 4e-04 | 57.9 | 0.009 | 0.014 | 0.493 | 0.51290 |
| 7 | 1889773 | rs10260968 | A | G | -0.020 | 0.004 | 1.75e-08 | 2e-04 | 31.7 | -0.010 | 0.013 | 0.433 | 0.59940 |
| 7 | 133589846 | rs10279261 | A | G | -0.021 | 0.004 | 5.00e-09 | 2e-04 | 34.2 | -0.008 | 0.014 | 0.570 | 0.63020 |
| 6 | 67405337 | rs10498846 | T | C | 0.021 | 0.004 | 6.62e-09 | 2e-04 | 33.6 | 0.006 | 0.013 | 0.636 | 0.48510 |
| 16 | 87443734 | rs1050847 | T | C | -0.022 | 0.004 | 1.67e-09 | 2e-04 | 36.3 | 0.000 | 0.013 | 0.983 | 0.55070 |
| 10 | 8803551 | rs10905461 | C | T | -0.024 | 0.004 | 7.35e-09 | 2e-04 | 33.4 | -0.023 | 0.018 | 0.211 | 0.73660 |
| 8 | 92775386 | rs10956809 | C | G | -0.021 | 0.004 | 6.32e-09 | 2e-04 | 33.7 | 0.011 | 0.013 | 0.420 | 0.44930 |
| 12 | 16748721 | rs11057005 | G | A | -0.021 | 0.004 | 4.85e-09 | 2e-04 | 34.2 | -0.004 | 0.013 | 0.778 | 0.44730 |
| 17 | 7795972 | rs11078713 | G | A | -0.020 | 0.004 | 2.23e-08 | 2e-04 | 31.3 | -0.011 | 0.013 | 0.421 | 0.40760 |
| 3 | 117804154 | rs1154693 | G | A | 0.033 | 0.005 | 3.12e-11 | 3e-04 | 44.1 | 0.001 | 0.016 | 0.971 | 0.83700 |
| 4 | 94052854 | rs1160685 | G | C | 0.021 | 0.004 | 7.20e-09 | 2e-04 | 33.5 | 0.004 | 0.014 | 0.779 | 0.45330 |
| 17 | 2072949 | rs11658881 | G | A | 0.020 | 0.004 | 2.43e-08 | 2e-04 | 31.1 | 0.024 | 0.014 | 0.077 | 0.45630 |
| 3 | 75009019 | rs11712680 | C | A | -0.027 | 0.005 | 3.51e-09 | 2e-04 | 34.9 | 0.018 | 0.019 | 0.353 | 0.17590 |
| 21 | 40555561 | rs117143374 | C | T | 0.029 | 0.005 | 2.76e-08 | 2e-04 | 30.9 | 0.016 | 0.022 | 0.453 | 0.12620 |
| 3 | 5725560 | rs11721059 | T | C | 0.020 | 0.004 | 2.17e-08 | 2e-04 | 31.3 | 0.028 | 0.013 | 0.034 | 0.49010 |
| 7 | 96629103 | rs11768481 | A | C | -0.023 | 0.004 | 7.00e-10 | 2e-04 | 38.0 | -0.011 | 0.013 | 0.431 | 0.36080 |
| 18 | 72535282 | rs11872397 | A | G | -0.025 | 0.004 | 1.43e-09 | 2e-04 | 36.6 | -0.002 | 0.016 | 0.921 | 0.25550 |
| 1 | 91196176 | rs12042107 | C | T | -0.022 | 0.004 | 4.22e-10 | 2e-04 | 39.0 | -0.027 | 0.014 | 0.052 | 0.50200 |
| 7 | 69735251 | rs12112638 | G | A | -0.025 | 0.004 | 1.34e-09 | 2e-04 | 36.8 | 0.016 | 0.015 | 0.286 | 0.23360 |
| 5 | 103816655 | rs12186738 | T | G | -0.033 | 0.005 | 3.42e-11 | 3e-04 | 43.9 | -0.041 | 0.019 | 0.030 | 0.15710 |
| 7 | 99185406 | rs12333760 | C | T | -0.029 | 0.005 | 1.44e-09 | 3e-04 | 36.6 | 0.003 | 0.016 | 0.847 | 0.17200 |
| 10 | 104563808 | rs12356821 | C | G | 0.039 | 0.005 | 6.27e-15 | 4e-04 | 60.8 | 0.005 | 0.019 | 0.797 | 0.14610 |
| 15 | 83922387 | rs12441907 | A | C | -0.029 | 0.005 | 1.06e-10 | 3e-04 | 41.7 | 0.011 | 0.017 | 0.522 | 0.16700 |
| 2 | 162802993 | rs12474587 | T | G | 0.028 | 0.004 | 1.25e-14 | 4e-04 | 59.5 | -0.017 | 0.014 | 0.218 | 0.39860 |
| 8 | 65073605 | rs12545053 | G | A | 0.020 | 0.004 | 2.43e-08 | 2e-04 | 31.1 | 0.009 | 0.014 | 0.528 | 0.40760 |
| 3 | 50224225 | rs12632110 | G | A | -0.023 | 0.004 | 4.78e-10 | 2e-04 | 38.8 | -0.031 | 0.015 | 0.031 | 0.66300 |
| 16 | 17575065 | rs12923427 | T | C | -0.024 | 0.004 | 4.44e-08 | 2e-04 | 29.9 | -0.011 | 0.023 | 0.643 | 0.21970 |
| 2 | 146143090 | rs13030994 | A | G | 0.036 | 0.004 | 3.56e-24 | 7e-04 | 103.0 | -0.002 | 0.013 | 0.858 | 0.50890 |
| 4 | 140927812 | rs13145728 | C | G | -0.023 | 0.004 | 2.14e-10 | 2e-04 | 40.3 | -0.027 | 0.014 | 0.051 | 0.36080 |
| 8 | 59814666 | rs13261666 | T | G | -0.027 | 0.004 | 3.90e-14 | 4e-04 | 57.2 | -0.013 | 0.013 | 0.317 | 0.56060 |
| 22 | 28781758 | rs134529 | C | T | -0.020 | 0.004 | 4.85e-08 | 2e-04 | 29.8 | 0.001 | 0.014 | 0.968 | 0.37870 |
| 5 | 154839646 | rs1385108 | T | C | 0.025 | 0.004 | 3.00e-09 | 2e-04 | 35.2 | 0.036 | 0.015 | 0.015 | 0.23960 |
| 15 | 47935843 | rs1435741 | A | G | 0.029 | 0.004 | 2.64e-16 | 4e-04 | 67.1 | 0.003 | 0.014 | 0.852 | 0.42740 |
| 2 | 155682556 | rs1445649 | C | T | 0.024 | 0.004 | 1.68e-11 | 3e-04 | 45.3 | 0.003 | 0.013 | 0.810 | 0.51990 |
| 20 | 31175258 | rs1555445 | T | A | 0.023 | 0.004 | 3.65e-09 | 2e-04 | 34.8 | 0.010 | 0.013 | 0.469 | 0.27630 |
| 8 | 27426077 | rs1565735 | A | T | -0.038 | 0.004 | 3.42e-17 | 5e-04 | 71.1 | 0.035 | 0.019 | 0.067 | 0.19980 |
| 8 | 93201036 | rs1899896 | T | C | 0.026 | 0.004 | 1.04e-11 | 3e-04 | 46.3 | 0.014 | 0.015 | 0.340 | 0.30910 |
| 12 | 121389500 | rs1971318 | T | C | 0.029 | 0.005 | 7.06e-09 | 2e-04 | 33.5 | 0.005 | 0.021 | 0.792 | 0.12820 |
| 1 | 210304319 | rs2046850 | T | C | -0.025 | 0.004 | 3.03e-08 | 2e-04 | 30.7 | -0.035 | 0.015 | 0.018 | 0.22760 |
| 1 | 87905828 | rs2050586 | C | G | -0.021 | 0.004 | 3.00e-08 | 2e-04 | 30.7 | 0.006 | 0.014 | 0.654 | 0.38070 |
| 2 | 200937901 | rs2107300 | G | C | -0.027 | 0.005 | 3.27e-08 | 2e-04 | 30.5 | 0.021 | 0.019 | 0.274 | 0.84890 |
| 1 | 66470206 | rs2186122 | T | A | 0.026 | 0.004 | 3.61e-13 | 3e-04 | 52.8 | -0.011 | 0.014 | 0.438 | 0.56860 |
| 6 | 52916062 | rs222449 | T | A | -0.025 | 0.004 | 1.08e-08 | 2e-04 | 32.7 | -0.048 | 0.016 | 0.002 | 0.77240 |
| 9 | 86707289 | rs2378662 | A | G | 0.021 | 0.004 | 4.16e-09 | 2e-04 | 34.5 | 0.004 | 0.013 | 0.764 | 0.54470 |
| 6 | 111644332 | rs240963 | C | T | -0.041 | 0.005 | 2.16e-17 | 5e-04 | 72.0 | 0.030 | 0.017 | 0.066 | 0.84990 |
| 2 | 104088751 | rs266047 | A | G | -0.031 | 0.004 | 3.36e-16 | 5e-04 | 66.6 | 0.008 | 0.013 | 0.566 | 0.49800 |
| 4 | 28456089 | rs292071 | C | T | 0.024 | 0.004 | 4.08e-09 | 2e-04 | 34.6 | 0.035 | 0.017 | 0.038 | 0.28530 |
| 1 | 44037685 | rs3001723 | A | G | 0.034 | 0.004 | 8.12e-18 | 5e-04 | 73.9 | 0.009 | 0.015 | 0.549 | 0.32600 |
| 1 | 8481016 | rs301805 | G | T | 0.021 | 0.004 | 2.80e-09 | 2e-04 | 35.3 | 0.001 | 0.014 | 0.915 | 0.52780 |
| 2 | 137542847 | rs35702515 | T | G | 0.025 | 0.004 | 2.43e-09 | 2e-04 | 35.6 | 0.013 | 0.018 | 0.453 | 0.15610 |
| 6 | 108994161 | rs3800227 | G | A | 0.023 | 0.004 | 1.93e-08 | 2e-04 | 31.6 | 0.005 | 0.014 | 0.741 | 0.67990 |
| 13 | 38357471 | rs3904512 | A | G | -0.021 | 0.004 | 3.23e-09 | 2e-04 | 35.0 | -0.009 | 0.014 | 0.521 | 0.42050 |
| 5 | 166989513 | rs4044321 | G | A | -0.028 | 0.004 | 6.08e-14 | 4e-04 | 56.3 | -0.021 | 0.014 | 0.139 | 0.66900 |
| 7 | 1708080 | rs4236259 | G | T | -0.025 | 0.004 | 3.35e-12 | 3e-04 | 48.5 | -0.002 | 0.013 | 0.865 | 0.52190 |
| 11 | 7950797 | rs4523689 | G | A | -0.021 | 0.004 | 1.55e-08 | 2e-04 | 32.0 | -0.017 | 0.013 | 0.214 | 0.42540 |
| 9 | 3014254 | rs4543592 | C | T | 0.022 | 0.004 | 7.46e-10 | 2e-04 | 37.9 | 0.019 | 0.014 | 0.155 | 0.49110 |
| 5 | 87756918 | rs4571506 | T | C | -0.028 | 0.004 | 1.09e-14 | 4e-04 | 59.7 | 0.008 | 0.013 | 0.567 | 0.48110 |
| 2 | 226332033 | rs4674993 | G | A | -0.025 | 0.004 | 1.32e-08 | 2e-04 | 32.3 | -0.039 | 0.018 | 0.026 | 0.19090 |
| 12 | 56508409 | rs4759228 | C | G | -0.022 | 0.004 | 3.58e-08 | 2e-04 | 30.4 | 0.026 | 0.016 | 0.100 | 0.28330 |
| 16 | 65604652 | rs4785836 | C | T | -0.020 | 0.004 | 2.26e-08 | 2e-04 | 31.3 | -0.027 | 0.013 | 0.048 | 0.40060 |
| 20 | 54387374 | rs56820925 | T | C | -0.022 | 0.004 | 1.73e-08 | 2e-04 | 31.8 | 0.026 | 0.014 | 0.059 | 0.35490 |
| 11 | 27679916 | rs6265 | T | C | -0.032 | 0.005 | 3.77e-12 | 3e-04 | 48.2 | 0.040 | 0.018 | 0.031 | 0.19680 |
| 2 | 182034448 | rs6433897 | C | T | 0.022 | 0.004 | 3.16e-08 | 2e-04 | 30.6 | -0.014 | 0.015 | 0.350 | 0.75150 |
| 18 | 50026142 | rs6508144 | G | C | -0.021 | 0.004 | 7.97e-09 | 2e-04 | 33.3 | -0.007 | 0.013 | 0.612 | 0.56960 |
| 1 | 50625979 | rs6669839 | T | C | 0.026 | 0.004 | 3.36e-09 | 2e-04 | 35.0 | 0.018 | 0.017 | 0.279 | 0.18990 |
| 2 | 623976 | rs6728726 | C | T | 0.035 | 0.005 | 6.73e-14 | 4e-04 | 56.1 | 0.017 | 0.018 | 0.356 | 0.83000 |
| 3 | 85624131 | rs6788098 | T | A | -0.031 | 0.004 | 1.91e-17 | 5e-04 | 72.2 | -0.010 | 0.015 | 0.503 | 0.63020 |
| 5 | 60374912 | rs6893752 | G | A | -0.024 | 0.004 | 3.25e-09 | 2e-04 | 35.0 | 0.018 | 0.015 | 0.244 | 0.75250 |
| 16 | 717085 | rs7197072 | T | C | -0.025 | 0.004 | 2.77e-09 | 2e-04 | 35.3 | 0.006 | 0.014 | 0.674 | 0.25550 |
| 17 | 30657058 | rs7224742 | T | C | -0.021 | 0.004 | 1.43e-08 | 2e-04 | 32.1 | -0.009 | 0.014 | 0.534 | 0.63320 |
| 5 | 106834363 | rs72789632 | T | C | -0.033 | 0.005 | 5.02e-10 | 2e-04 | 38.7 | 0.034 | 0.024 | 0.164 | 0.12130 |
| 18 | 42632652 | rs72896886 | C | G | -0.027 | 0.005 | 2.75e-08 | 2e-04 | 30.9 | -0.036 | 0.018 | 0.047 | 0.14410 |
| 13 | 100548329 | rs7322872 | T | C | -0.026 | 0.004 | 3.58e-09 | 2e-04 | 34.8 | -0.001 | 0.017 | 0.959 | 0.78830 |
| 1 | 73766037 | rs7555507 | T | C | -0.024 | 0.004 | 1.14e-11 | 3e-04 | 46.1 | -0.031 | 0.013 | 0.021 | 0.45630 |
| 2 | 60024857 | rs7585579 | G | C | 0.022 | 0.004 | 1.88e-09 | 3e-04 | 36.1 | -0.002 | 0.013 | 0.885 | 0.51790 |
| 14 | 29500130 | rs76214862 | C | A | -0.025 | 0.005 | 3.99e-08 | 2e-04 | 30.2 | -0.033 | 0.016 | 0.032 | 0.21670 |
| 19 | 4474725 | rs76608582 | A | C | -0.050 | 0.008 | 1.94e-09 | 2e-04 | 36.0 | -0.037 | 0.029 | 0.192 | 0.04573 |
| 10 | 63674885 | rs7921378 | C | G | -0.025 | 0.004 | 8.26e-13 | 3e-04 | 51.2 | 0.023 | 0.013 | 0.078 | 0.49010 |
| 11 | 85980958 | rs7929518 | G | A | 0.024 | 0.004 | 1.56e-08 | 2e-04 | 32.0 | -0.012 | 0.015 | 0.442 | 0.77240 |
| 11 | 112911004 | rs7938812 | G | T | 0.044 | 0.004 | 2.71e-33 | 9e-04 | 145.0 | 0.028 | 0.014 | 0.040 | 0.43640 |
| 12 | 69655167 | rs7969559 | G | A | -0.024 | 0.004 | 7.31e-10 | 3e-04 | 37.9 | -0.012 | 0.014 | 0.372 | 0.68190 |
| 6 | 98748008 | rs9401770 | A | G | 0.028 | 0.004 | 3.47e-12 | 3e-04 | 48.4 | -0.010 | 0.014 | 0.498 | 0.29320 |
| 10 | 125680419 | rs9423279 | G | C | -0.021 | 0.004 | 3.21e-08 | 2e-04 | 30.6 | 0.001 | 0.014 | 0.965 | 0.63720 |
| 13 | 66947124 | rs9540729 | T | A | -0.020 | 0.004 | 3.82e-08 | 2e-04 | 30.2 | 0.023 | 0.013 | 0.089 | 0.50600 |
| 4 | 67825894 | rs993700 | C | T | -0.026 | 0.004 | 1.53e-09 | 2e-04 | 36.5 | -0.022 | 0.016 | 0.163 | 0.76740 |

**Supplementary table 2.29. Detailed information of instrumental SNPs for Cigarettes Per Day in the analysis of PPD.**

| **Chr** | **Position** | **SNP** | **EA** | **OA** | **beta.exposure** | **se.exposure** | **pval.exposure** | **R2** | **F_val** | **beta.outcome** | **se.outcome** | **pval.outcome** | **Eaf.outcome** |
| --- | --- | --- | --- | --- | --- | --- | --- | --- | --- | --- | --- | --- | --- |
| 1 | 154548521 | rs2072659 | G | C | -0.065 | 0.009 | 1.71e-12 | 0.0008 | 49.8 | 0.024 | 0.022 | 0.289 | 0.103 |
| 3 | 16872929 | rs2084533 | T | C | 0.034 | 0.006 | 1.22e-08 | 0.0005 | 32.5 | -0.001 | 0.014 | 0.972 | 0.306 |
| 3 | 48935583 | rs7431710 | A | G | -0.035 | 0.006 | 1.82e-09 | 0.0006 | 36.2 | -0.015 | 0.014 | 0.278 | 0.663 |
| 4 | 67053769 | rs11725618 | C | T | 0.036 | 0.006 | 4.67e-09 | 0.0005 | 34.3 | -0.010 | 0.015 | 0.518 | 0.272 |
| 4 | 67904931 | rs787362 | A | T | 0.030 | 0.006 | 4.50e-08 | 0.0005 | 29.9 | 0.007 | 0.013 | 0.583 | 0.478 |
| 6 | 26214473 | rs806798 | C | T | -0.031 | 0.006 | 2.48e-08 | 0.0005 | 31.1 | -0.007 | 0.014 | 0.588 | 0.592 |
| 7 | 32333642 | rs215600 | A | G | -0.049 | 0.006 | 1.10e-17 | 0.0011 | 73.3 | -0.016 | 0.015 | 0.281 | 0.720 |
| 8 | 42579203 | rs58379124 | C | T | 0.067 | 0.007 | 9.00e-25 | 0.0017 | 106.0 | -0.001 | 0.015 | 0.970 | 0.756 |
| 8 | 27442127 | rs73229090 | A | C | 0.055 | 0.009 | 2.44e-10 | 0.0006 | 40.1 | 0.042 | 0.023 | 0.063 | 0.094 |
| 8 | 64604218 | rs790564 | C | A | -0.041 | 0.006 | 3.97e-11 | 0.0007 | 43.6 | 0.006 | 0.016 | 0.688 | 0.759 |
| 9 | 136502369 | rs3025383 | C | T | -0.058 | 0.007 | 2.22e-16 | 0.0010 | 67.4 | -0.002 | 0.018 | 0.896 | 0.170 |
| 11 | 46465361 | rs75494138 | T | C | 0.060 | 0.011 | 1.45e-08 | 0.0004 | 32.1 | -0.009 | 0.033 | 0.795 | 0.042 |
| 11 | 113448762 | rs7928017 | A | C | -0.033 | 0.006 | 3.14e-09 | 0.0005 | 35.1 | -0.022 | 0.015 | 0.145 | 0.288 |
| 11 | 16377044 | rs7951365 | C | T | 0.039 | 0.006 | 6.63e-11 | 0.0006 | 42.6 | -0.022 | 0.016 | 0.153 | 0.242 |
| 15 | 78857939 | rs55853698 | G | T | 0.182 | 0.006 | 1.00e-200 | 0.0145 | 941.0 | 0.007 | 0.014 | 0.625 | 0.336 |
| 15 | 59155050 | rs632811 | G | A | -0.037 | 0.006 | 1.03e-08 | 0.0006 | 32.8 | -0.006 | 0.013 | 0.648 | 0.495 |
| 16 | 52074123 | rs2386571 | C | A | -0.032 | 0.006 | 1.03e-08 | 0.0005 | 32.8 | -0.007 | 0.014 | 0.637 | 0.663 |
| 16 | 89772619 | rs4785587 | A | G | -0.034 | 0.006 | 1.27e-09 | 0.0006 | 36.9 | 0.000 | 0.014 | 0.989 | 0.626 |
| 19 | 41305530 | rs34406232 | A | C | -0.147 | 0.017 | 1.33e-18 | 0.0011 | 77.5 | 0.012 | 0.042 | 0.773 | 0.026 |
| 19 | 41353107 | rs56113850 | C | T | 0.107 | 0.006 | 1.10e-81 | 0.0056 | 366.0 | -0.004 | 0.014 | 0.774 | 0.563 |
| 19 | 4060707 | rs895330 | G | C | -0.039 | 0.007 | 2.68e-08 | 0.0005 | 30.9 | -0.008 | 0.018 | 0.652 | 0.164 |
| 20 | 61986949 | rs2273500 | C | T | 0.068 | 0.008 | 2.47e-18 | 0.0012 | 76.3 | 0.029 | 0.016 | 0.074 | 0.216 |
| 20 | 31047533 | rs2424888 | A | G | 0.033 | 0.006 | 2.76e-09 | 0.0005 | 35.3 | 0.014 | 0.013 | 0.281 | 0.527 |

**Supplementary table 2.30. Detailed information of instrumental SNPs for Alcohol consumption in the analysis of PPD.**

| **Chr** | **Position** | **SNP** | **EA** | **OA** | **beta.exposure** | **se.exposure** | **pval.exposure** | **R2** | **F_val** | **beta.outcome** | **se.outcome** | **pval.outcome** | **Eaf.outcome** |
| --- | --- | --- | --- | --- | --- | --- | --- | --- | --- | --- | --- | --- | --- |
| 7 | 69783020 | rs10085696 | G | A | -0.016 | 0.002 | 1.24e-10 | 0.0001 | 41.4 | 0.028 | 0.016 | 0.089 | 0.18190 |
| 1 | 165119792 | rs10753661 | A | G | -0.011 | 0.002 | 4.24e-08 | 0.0001 | 30.0 | 0.004 | 0.014 | 0.768 | 0.73860 |
| 14 | 57274519 | rs1123285 | G | C | -0.012 | 0.002 | 1.36e-09 | 0.0001 | 36.7 | -0.027 | 0.014 | 0.049 | 0.34990 |
| 16 | 73912503 | rs11860773 | C | T | -0.015 | 0.002 | 8.35e-10 | 0.0001 | 37.7 | 0.004 | 0.017 | 0.799 | 0.18590 |
| 4 | 100239319 | rs1229984 | C | T | 0.188 | 0.006 | 1.00e-200 | 0.0032 | 927.0 | 0.180 | 0.096 | 0.060 | 0.97120 |
| 2 | 27730940 | rs1260326 | C | T | 0.024 | 0.002 | 3.33e-33 | 0.0003 | 144.0 | 0.004 | 0.014 | 0.762 | 0.58950 |
| 4 | 103188709 | rs13107325 | T | C | -0.036 | 0.004 | 1.23e-20 | 0.0002 | 86.8 | 0.122 | 0.058 | 0.035 | 0.07952 |
| 16 | 85721809 | rs13332432 | G | C | 0.014 | 0.002 | 5.94e-11 | 0.0001 | 42.8 | 0.057 | 0.015 | 0.000 | 0.29620 |
| 12 | 92081800 | rs1387766 | A | G | -0.011 | 0.002 | 4.79e-08 | 0.0001 | 29.8 | 0.017 | 0.014 | 0.236 | 0.61530 |
| 16 | 28526897 | rs153106 | C | T | -0.014 | 0.002 | 3.63e-12 | 0.0001 | 48.3 | -0.018 | 0.013 | 0.181 | 0.35490 |
| 4 | 42117559 | rs16854020 | A | G | 0.018 | 0.003 | 4.82e-10 | 0.0001 | 38.7 | -0.012 | 0.024 | 0.614 | 0.11830 |
| 11 | 113655696 | rs17542254 | G | A | 0.013 | 0.002 | 8.96e-10 | 0.0001 | 37.5 | 0.010 | 0.016 | 0.512 | 0.24550 |
| 11 | 27694241 | rs2049045 | C | G | -0.014 | 0.003 | 3.97e-08 | 0.0001 | 30.2 | 0.039 | 0.019 | 0.036 | 0.19090 |
| 7 | 103812171 | rs2299409 | A | G | -0.011 | 0.002 | 4.80e-08 | 0.0001 | 29.8 | 0.011 | 0.013 | 0.387 | 0.49800 |
| 8 | 126500031 | rs28601761 | G | C | 0.011 | 0.002 | 7.60e-09 | 0.0001 | 33.4 | 0.008 | 0.013 | 0.533 | 0.40760 |
| 1 | 173848808 | rs28680958 | A | G | -0.014 | 0.002 | 9.78e-09 | 0.0001 | 32.9 | 0.004 | 0.017 | 0.816 | 0.22170 |
| 4 | 39413780 | rs28712821 | A | G | 0.028 | 0.002 | 1.10e-46 | 0.0004 | 206.0 | -0.017 | 0.014 | 0.233 | 0.61330 |
| 3 | 85403892 | rs28732378 | G | A | -0.017 | 0.002 | 2.24e-14 | 0.0001 | 58.3 | -0.020 | 0.015 | 0.204 | 0.71870 |
| 14 | 94844947 | rs28929474 | T | C | -0.048 | 0.007 | 2.39e-11 | 0.0001 | 44.6 | -0.007 | 0.048 | 0.891 | 0.01690 |
| 4 | 143654889 | rs331939 | A | G | -0.012 | 0.002 | 4.50e-09 | 0.0001 | 34.4 | -0.019 | 0.015 | 0.202 | 0.36280 |
| 17 | 7733833 | rs34121753 | G | A | 0.011 | 0.002 | 1.39e-08 | 0.0001 | 32.2 | 0.001 | 0.013 | 0.922 | 0.59240 |
| 13 | 68117681 | rs34704785 | T | C | -0.011 | 0.002 | 4.52e-08 | 0.0001 | 29.9 | 0.002 | 0.013 | 0.905 | 0.52090 |
| 2 | 144272376 | rs4233567 | T | C | -0.013 | 0.002 | 3.83e-10 | 0.0001 | 39.2 | -0.019 | 0.014 | 0.172 | 0.34590 |
| 11 | 113412443 | rs4309187 | C | A | 0.015 | 0.002 | 1.37e-12 | 0.0001 | 50.2 | 0.014 | 0.018 | 0.440 | 0.69580 |
| 11 | 47428565 | rs4752999 | T | C | -0.015 | 0.002 | 2.03e-12 | 0.0001 | 49.5 | -0.013 | 0.014 | 0.357 | 0.32800 |
| 5 | 87854395 | rs4916723 | C | A | -0.011 | 0.002 | 8.07e-09 | 0.0001 | 33.3 | -0.007 | 0.013 | 0.576 | 0.41350 |
| 2 | 45154908 | rs528301 | A | G | 0.016 | 0.002 | 1.25e-15 | 0.0001 | 64.0 | 0.016 | 0.014 | 0.261 | 0.57060 |
| 5 | 155902003 | rs55872084 | T | G | 0.013 | 0.002 | 1.98e-08 | 0.0001 | 31.5 | 0.021 | 0.018 | 0.246 | 0.22660 |
| 9 | 108755622 | rs55932213 | G | A | 0.012 | 0.002 | 1.80e-08 | 0.0001 | 31.7 | 0.004 | 0.015 | 0.771 | 0.70180 |
| 20 | 25027630 | rs6106989 | A | G | 0.011 | 0.002 | 3.81e-08 | 0.0001 | 30.2 | 0.002 | 0.014 | 0.899 | 0.64310 |
| 2 | 44296002 | rs62135521 | T | G | -0.026 | 0.005 | 9.91e-09 | 0.0001 | 32.9 | -0.030 | 0.033 | 0.367 | 0.03678 |
| 2 | 63269604 | rs6739804 | C | T | -0.013 | 0.002 | 4.72e-10 | 0.0001 | 38.8 | -0.014 | 0.014 | 0.306 | 0.64210 |
| 17 | 44189858 | rs76640332 | A | G | -0.021 | 0.002 | 1.47e-18 | 0.0001 | 77.3 | 0.064 | 0.024 | 0.008 | 0.22960 |
| 4 | 100279889 | rs78234152 | A | G | 0.028 | 0.003 | 2.18e-19 | 0.0001 | 81.1 | -0.007 | 0.019 | 0.714 | 0.09245 |
| 16 | 72338507 | rs79616692 | C | G | 0.019 | 0.003 | 2.38e-09 | 0.0001 | 35.6 | -0.026 | 0.020 | 0.191 | 0.10540 |
| 19 | 49248730 | rs838145 | A | G | -0.016 | 0.002 | 3.87e-16 | 0.0001 | 66.3 | -0.009 | 0.014 | 0.535 | 0.58150 |

**Supplementary table 2.31. Detailed information of instrumental SNPs for coffee intake in the analysis of PPD.**

| **Chr** | **Position** | **SNP** | **EA** | **OA** | **beta.exposure** | **se.exposure** | **pval.exposure** | **R2** | **F_val** | **beta.outcome** | **se.outcome** | **pval.outcome** | **Eaf.outcome** |
| --- | --- | --- | --- | --- | --- | --- | --- | --- | --- | --- | --- | --- | --- |
| 9 | 27953724 | rs10119174 | C | G | -0.009 | 0.002 | 1.00000e-08 | 0e+00 | 32.774 | 0.014 | 0.014 | 0.337 | 0.60040 |
| 7 | 75615006 | rs1057868 | T | C | 0.020 | 0.002 | 5.40008e-29 | 2e-04 | 124.901 | 0.010 | 0.014 | 0.476 | 0.29520 |
| 15 | 75174251 | rs117968677 | A | G | -0.031 | 0.006 | 1.89998e-08 | 0e+00 | 31.645 | 0.003 | 0.036 | 0.937 | 0.02883 |
| 5 | 7391462 | rs12514566 | A | G | -0.011 | 0.002 | 2.39994e-11 | 1e-04 | 44.651 | 0.021 | 0.016 | 0.181 | 0.32110 |
| 2 | 49368391 | rs12989746 | T | G | 0.010 | 0.002 | 2.80001e-08 | 0e+00 | 30.821 | -0.004 | 0.015 | 0.809 | 0.26940 |
| 22 | 41215672 | rs13054099 | C | T | -0.011 | 0.002 | 4.30002e-09 | 0e+00 | 34.460 | 0.045 | 0.016 | 0.004 | 0.26240 |
| 5 | 87943710 | rs13163336 | A | C | 0.015 | 0.002 | 1.29987e-11 | 1e-04 | 45.742 | 0.001 | 0.021 | 0.949 | 0.17100 |
| 6 | 98312143 | rs1338549 | G | T | -0.009 | 0.002 | 5.60003e-09 | 0e+00 | 33.961 | 0.011 | 0.014 | 0.419 | 0.51390 |
| 2 | 637498 | rs13387939 | A | C | 0.017 | 0.002 | 9.79941e-15 | 1e-04 | 59.930 | 0.019 | 0.018 | 0.295 | 0.82600 |
| 16 | 53800954 | rs1421085 | C | T | 0.019 | 0.002 | 1.69981e-29 | 2e-04 | 127.159 | 0.008 | 0.013 | 0.529 | 0.43240 |
| 2 | 62780440 | rs1527961 | C | T | -0.013 | 0.002 | 1.70000e-08 | 0e+00 | 31.808 | 0.015 | 0.020 | 0.458 | 0.13520 |
| 18 | 55032486 | rs1942965 | C | T | -0.009 | 0.002 | 3.79997e-08 | 0e+00 | 30.236 | 0.010 | 0.013 | 0.481 | 0.50600 |
| 4 | 106075498 | rs2189234 | G | T | 0.010 | 0.002 | 1.79999e-09 | 0e+00 | 36.172 | 0.004 | 0.014 | 0.787 | 0.63020 |
| 6 | 51179260 | rs2465037 | A | C | -0.011 | 0.002 | 4.79999e-10 | 1e-04 | 38.772 | 0.005 | 0.015 | 0.711 | 0.35690 |
| 15 | 75027880 | rs2472297 | T | C | 0.046 | 0.002 | 1.09901e-142 | 8e-04 | 646.735 | 0.000 | 0.015 | 0.990 | 0.21470 |
| 4 | 17424930 | rs2597805 | T | C | 0.010 | 0.002 | 2.00000e-08 | 0e+00 | 31.489 | -0.015 | 0.014 | 0.262 | 0.65410 |
| 7 | 73037956 | rs34060476 | G | A | 0.018 | 0.002 | 7.50067e-15 | 1e-04 | 60.450 | -0.025 | 0.020 | 0.210 | 0.12330 |
| 7 | 17284577 | rs4410790 | C | T | 0.039 | 0.002 | 1.19950e-120 | 7e-04 | 545.509 | -0.006 | 0.014 | 0.663 | 0.61530 |
| 8 | 109128653 | rs442355 | C | G | -0.011 | 0.002 | 1.89998e-09 | 0e+00 | 36.096 | -0.031 | 0.015 | 0.041 | 0.26940 |
| 1 | 96274668 | rs4615895 | A | G | 0.012 | 0.002 | 4.19952e-11 | 1e-04 | 43.520 | -0.038 | 0.016 | 0.016 | 0.78730 |
| 18 | 57852587 | rs476828 | C | T | 0.017 | 0.002 | 5.60015e-20 | 1e-04 | 83.751 | -0.023 | 0.017 | 0.180 | 0.24650 |
| 1 | 177855517 | rs516636 | A | C | 0.012 | 0.002 | 4.00000e-09 | 0e+00 | 34.632 | -0.013 | 0.017 | 0.444 | 0.19180 |
| 19 | 41353107 | rs56113850 | C | T | 0.013 | 0.002 | 8.90020e-15 | 1e-04 | 60.129 | -0.004 | 0.014 | 0.774 | 0.59240 |
| 17 | 60150383 | rs57918684 | A | G | 0.013 | 0.002 | 8.60003e-09 | 0e+00 | 33.141 | 0.027 | 0.015 | 0.077 | 0.14810 |
| 20 | 62891820 | rs6062682 | T | C | 0.010 | 0.002 | 2.50000e-10 | 1e-04 | 40.020 | 0.004 | 0.014 | 0.792 | 0.43840 |
| 20 | 45840459 | rs6063085 | C | A | 0.010 | 0.002 | 4.49997e-10 | 1e-04 | 38.899 | -0.030 | 0.014 | 0.031 | 0.36680 |
| 12 | 11316437 | rs61928609 | C | A | -0.015 | 0.002 | 1.29987e-11 | 1e-04 | 45.854 | 0.001 | 0.024 | 0.982 | 0.86880 |
| 17 | 46155786 | rs62064918 | T | C | -0.010 | 0.002 | 4.09996e-08 | 0e+00 | 30.101 | -0.008 | 0.017 | 0.628 | 0.22470 |
| 18 | 40950954 | rs630194 | C | T | -0.011 | 0.002 | 2.29985e-11 | 1e-04 | 44.678 | 0.012 | 0.014 | 0.379 | 0.35590 |
| 8 | 110443480 | rs6469262 | C | T | -0.009 | 0.002 | 1.89998e-08 | 0e+00 | 31.576 | -0.027 | 0.013 | 0.046 | 0.54470 |
| 17 | 17845800 | rs7224815 | T | A | -0.011 | 0.002 | 3.69999e-11 | 1e-04 | 43.765 | -0.001 | 0.013 | 0.964 | 0.48610 |
| 7 | 17570479 | rs73075167 | T | A | -0.016 | 0.002 | 5.00035e-11 | 1e-04 | 43.191 | 0.005 | 0.021 | 0.804 | 0.11730 |
| 19 | 18495908 | rs75347775 | A | G | 0.010 | 0.002 | 2.69998e-08 | 0e+00 | 30.933 | -0.036 | 0.015 | 0.018 | 0.24550 |
| 2 | 27742603 | rs780093 | C | T | 0.013 | 0.002 | 1.00000e-15 | 1e-04 | 64.366 | 0.004 | 0.014 | 0.797 | 0.58850 |
| 7 | 32930597 | rs7811609 | T | C | 0.009 | 0.002 | 4.00000e-08 | 0e+00 | 30.137 | -0.006 | 0.014 | 0.643 | 0.36480 |
| 8 | 33790200 | rs78267637 | G | C | -0.025 | 0.004 | 3.89996e-09 | 0e+00 | 34.695 | 0.044 | 0.028 | 0.117 | 0.04771 |
| 16 | 70927078 | rs8056750 | T | C | 0.011 | 0.002 | 1.29999e-09 | 1e-04 | 36.776 | -0.010 | 0.014 | 0.440 | 0.37380 |
| 6 | 108983527 | rs9398171 | T | C | 0.011 | 0.002 | 1.09999e-09 | 0e+00 | 37.210 | 0.006 | 0.014 | 0.649 | 0.64510 |

**Supplementary table 2.32. Detailed information of instrumental SNPs for tea intake in the analysis of PPD.**

| **Chr** | **Position** | **SNP** | **EA** | **OA** | **beta.exposure** | **se.exposure** | **pval.exposure** | **R2** | **F_val** | **beta.outcome** | **se.outcome** | **pval.outcome** | **Eaf.outcome** |
| --- | --- | --- | --- | --- | --- | --- | --- | --- | --- | --- | --- | --- | --- |
| 1 | 150722844 | rs11587444 | G | A | 0.014 | 0.002 | 1.00000e-10 | 0.0001 | 41.789 | -0.014 | 0.013 | 0.286 | 0.404 |
| 1 | 93552187 | rs11164870 | G | C | -0.012 | 0.002 | 4.20001e-08 | 0.0001 | 30.037 | 0.002 | 0.014 | 0.859 | 0.628 |
| 1 | 174189269 | rs56188862 | C | T | -0.016 | 0.002 | 4.30031e-13 | 0.0001 | 52.497 | 0.009 | 0.014 | 0.510 | 0.366 |
| 2 | 58515375 | rs1156588 | G | A | -0.015 | 0.003 | 2.90001e-09 | 0.0001 | 35.241 | 0.007 | 0.016 | 0.675 | 0.227 |
| 3 | 50239803 | rs57462170 | A | G | 0.019 | 0.003 | 1.89998e-08 | 0.0001 | 31.620 | 0.006 | 0.019 | 0.737 | 0.142 |
| 3 | 89525505 | rs2117137 | G | A | 0.013 | 0.002 | 1.70000e-09 | 0.0001 | 36.338 | 0.003 | 0.013 | 0.813 | 0.485 |
| 4 | 89039082 | rs1481012 | G | A | -0.026 | 0.003 | 5.30029e-15 | 0.0001 | 61.148 | -0.027 | 0.025 | 0.282 | 0.075 |
| 5 | 60465365 | rs34619 | A | G | 0.012 | 0.002 | 4.30002e-08 | 0.0001 | 30.021 | 0.007 | 0.013 | 0.614 | 0.422 |
| 5 | 152031650 | rs72797284 | G | A | -0.017 | 0.002 | 7.00003e-13 | 0.0001 | 51.558 | -0.014 | 0.016 | 0.393 | 0.209 |
| 6 | 137222671 | rs7757102 | G | A | -0.012 | 0.002 | 3.09999e-08 | 0.0001 | 30.624 | -0.013 | 0.013 | 0.327 | 0.566 |
| 6 | 51283110 | rs2478875 | G | A | 0.022 | 0.003 | 5.10035e-17 | 0.0002 | 70.299 | 0.022 | 0.018 | 0.204 | 0.172 |
| 6 | 137095269 | rs149805207 | G | A | -0.072 | 0.013 | 1.09999e-08 | 0.0001 | 32.685 | 0.079 | 0.048 | 0.097 | 0.021 |
| 7 | 17284577 | rs4410790 | C | T | 0.041 | 0.002 | 3.40017e-76 | 0.0008 | 341.270 | -0.006 | 0.014 | 0.663 | 0.663 |
| 7 | 75616105 | rs17685 | A | G | 0.023 | 0.002 | 1.59993e-22 | 0.0002 | 95.364 | 0.012 | 0.014 | 0.382 | 0.380 |
| 7 | 17558580 | rs141071726 | A | G | 0.041 | 0.007 | 2.19999e-09 | 0.0001 | 35.754 | -0.010 | 0.057 | 0.857 | 0.015 |
| 7 | 39293033 | rs9648476 | A | G | 0.013 | 0.002 | 1.09999e-08 | 0.0001 | 32.722 | -0.008 | 0.014 | 0.568 | 0.638 |
| 7 | 141673345 | rs713598 | G | C | 0.013 | 0.002 | 5.19996e-10 | 0.0001 | 38.590 | 0.010 | 0.014 | 0.474 | 0.350 |
| 8 | 22088975 | rs13282783 | T | C | -0.014 | 0.002 | 7.90005e-09 | 0.0001 | 33.289 | -0.025 | 0.013 | 0.063 | 0.408 |
| 9 | 7054124 | rs56348300 | G | C | 0.016 | 0.003 | 6.10000e-09 | 0.0001 | 33.799 | 0.011 | 0.020 | 0.566 | 0.131 |
| 10 | 129152608 | rs10764990 | A | G | -0.012 | 0.002 | 1.89998e-08 | 0.0001 | 31.589 | -0.006 | 0.013 | 0.655 | 0.531 |
| 10 | 12692902 | rs10752269 | A | G | -0.013 | 0.002 | 1.29999e-09 | 0.0001 | 36.878 | -0.015 | 0.014 | 0.284 | 0.648 |
| 10 | 86850616 | rs2351187 | A | G | 0.013 | 0.002 | 1.60000e-08 | 0.0001 | 31.959 | -0.012 | 0.014 | 0.395 | 0.314 |
| 11 | 1679769 | rs17245213 | A | G | -0.015 | 0.003 | 2.00000e-08 | 0.0001 | 31.521 | 0.001 | 0.018 | 0.957 | 0.175 |
| 11 | 16286183 | rs10741694 | C | T | 0.015 | 0.002 | 7.89951e-12 | 0.0001 | 46.784 | 0.004 | 0.015 | 0.776 | 0.733 |
| 11 | 59192089 | rs1453548 | A | T | -0.013 | 0.002 | 2.99999e-09 | 0.0001 | 35.168 | 0.003 | 0.014 | 0.845 | 0.671 |
| 12 | 11284772 | rs977474 | T | C | 0.022 | 0.003 | 2.39994e-14 | 0.0001 | 58.180 | 0.002 | 0.024 | 0.949 | 0.915 |
| 13 | 80168720 | rs2783129 | G | C | -0.012 | 0.002 | 3.79997e-08 | 0.0001 | 30.254 | -0.021 | 0.013 | 0.115 | 0.449 |
| 13 | 100272019 | rs17576658 | A | G | -0.013 | 0.002 | 4.09996e-08 | 0.0001 | 30.117 | 0.002 | 0.016 | 0.901 | 0.230 |
| 13 | 111531264 | rs6829 | T | C | -0.012 | 0.002 | 3.69999e-08 | 0.0001 | 30.282 | -0.010 | 0.014 | 0.461 | 0.600 |
| 13 | 56444529 | rs2645929 | G | A | -0.015 | 0.003 | 3.50002e-08 | 0.0001 | 30.424 | 0.019 | 0.019 | 0.305 | 0.850 |
| 15 | 60902512 | rs12591786 | T | C | -0.018 | 0.003 | 3.69999e-10 | 0.0001 | 39.274 | -0.026 | 0.022 | 0.239 | 0.099 |
| 15 | 75027880 | rs2472297 | T | C | 0.053 | 0.002 | 2.30144e-109 | 0.0011 | 493.646 | 0.000 | 0.015 | 0.990 | 0.248 |
| 16 | 53799847 | rs9937354 | A | G | -0.014 | 0.002 | 4.90004e-11 | 0.0001 | 43.231 | 0.010 | 0.013 | 0.464 | 0.430 |
| 16 | 24717600 | rs9302428 | G | C | 0.012 | 0.002 | 2.59998e-08 | 0.0001 | 30.949 | 0.005 | 0.014 | 0.729 | 0.638 |
| 17 | 40819809 | rs2279844 | A | G | -0.012 | 0.002 | 4.00000e-08 | 0.0001 | 30.151 | 0.001 | 0.014 | 0.952 | 0.364 |
| 19 | 19410622 | rs4808193 | C | T | 0.015 | 0.002 | 1.69981e-11 | 0.0001 | 45.240 | -0.022 | 0.016 | 0.152 | 0.247 |
| 19 | 4338173 | rs57631352 | G | A | -0.013 | 0.002 | 1.70000e-08 | 0.0001 | 31.868 | -0.011 | 0.014 | 0.442 | 0.336 |
[truncated: 135,310 more chars]
