# Supplementary material for: Causal effects of potential risk factors on postpartum depression: a Mendelian randomization study
Source: Front Psychiatry. 2023 Dec 20;14:1275834. doi: 10.3389/fpsyt.2023.1275834 (PMC10761415; doi:10.3389/fpsyt.2023.1275834)
Supplement: Supplementary file 3 [file Table_3.docx]

**Supplementary table 3. IVW analysis of all potential factors on PPD.**

| Potential risk factors | Traits | n(SNP) | OR (95% CI) | *p* value | BH_corrected_ *p* |
| --- | --- | --- | --- | --- | --- |
| Psychiatric disorders | SCZ | 147 | 1.13 (1.08, 1.19) | 1.64E-06 | 1.64E-05 |
|  | Autism Spectrum Disorder | 32 | 1.04 (0.96, 1.12) | 0.346 | 0.585 |
|  | Bipolar disorder | 6 | 1.04 (0.96, 1.13) | 0.313 | 0.585 |
|  | MD | 46 | 2.05 (1.76, 2.39) | 1.26E-20 | 5.04E-19 |
|  | Attention deficit/hyperactivity disorder | 9 | 1.10 (0.95, 1.27) | 0.215 | 0.571 |
|  | Anxiety | 6 | 1.00 (0.93, 1.06) | 0.901 | 0.901 |
| Overweight | BMI | 406 | 1.13 (1.03, 1.24) | 0.010 | 0.050 |
| Blood pressure | Diastolic blood pressure | 194 | 1.08 (0.93, 1.25) | 0.296 | 0.585 |
|  | Pulse pressure | 199 | 1.05 (0.90, 1.22) | 0.514 | 0.734 |
|  | Systolic blood pressure | 204 | 1.05 (0.91, 1.20) | 0.507 | 0.734 |
| Glucose | Glycated hemoglobin | 70 | 0.86 (0.68, 1.09) | 0.213 | 0.571 |
|  | Fasting glucose | 60 | 0.97 (0.78, 1.20) | 0.752 | 0.813 |
|  | Fasting insulin | 37 | 0.94 (0.67, 1.32) | 0.715 | 0.813 |
|  | Two-hour glucose | 10 | 1.05 (0.92, 1.20) | 0.434 | 0.668 |
| Lipids | Triglycerides | 266 | 1.05 (0.98, 1.12) | 0.146 | 0.571 |
|  | LDL-c | 141 | 0.94 (0.85, 1.03) | 0.175 | 0.571 |
|  | HDL-c | 293 | 0.97 (0.97, 1.05) | 0.420 | 0.668 |
|  | Apolipoprotein A-I | 251 | 0.99 (0.91, 1.70) | 0.749 | 0.813 |
|  | Apolipoprotein B | 162 | 0.96 (0.89, 1.04) | 0.329 | 0.585 |
| Sex-hormones | Total Testosterone | 95 | 0.98 (0.88, 1.08) | 0.625 | 0.785 |
|  | Bioavailable Testosterone | 109 | 0.98 (0.88, 1.09) | 0.703 | 0.813 |
|  | Sex hormone binding globulin | 164 | 0.98 (0.91, 1.05) | 0.547 | 0.751 |
|  | Oestradiol | 15 | 1.05 (0.88, 1.26) | 0.563 | 0.751 |
| Thyroid function | Free thyroxine | 11 | 0.93 (0.81, 1.06) | 0.254 | 0.571 |
|  | TSH | 23 | 1.02 (0.94, 1.12) | 0.628 | 0.785 |
|  | Hypothyroidism | 8 | 1.01 (0.95, 1.07) | 0.840 | 0.863 |
|  | Hyperthyroidism | 12 | 0.98 (0.94, 1.02) | 0.232 | 0.571 |
| Inflammatory biomarkers | Serum 25-Hydroxyvitamin D levels adjusted BMI | 102 | 1.03 (0.91, 1.17) | 0.663 | 0.804 |
|  | C-reactive protein levels | 231 | 1.04 (0.96, 1.13) | 0.351 | 0.585 |
| Habits | SI | 80 | 1.33 (1.14, 1.54) | 2.00E-04 | 1.33E-03 |
|  | Cigarettes per day | 22 | 1.06 (0.96, 1.17) | 0.259 | 0.571 |
|  | Alcohol consumption | 36 | 1.26 (0.83, 1.90) | 0.271 | 0.571 |
|  | Coffee intake | 36 | 0.78 (0.54, 1.12) | 0.181 | 0.571 |
|  | Tea intake | 40 | 0.97 (0.76, 1.25) | 0.841 | 0.863 |
| Socioeconomic Factors | Years of schooling | 294 | 0.56 (0.48, 0.66) | 1.10E-12 | 1.47E-11 |
|  | Average total household income before tax | 40 | 0.62 (0.44, 0.88) | 0.008 | 4.57E-02 |
|  | Age at first sexual intercourse | 161 | 0.47 (0.40, 0.57) | 1.51E-16 | 3.02E-15 |
|  | Age at first birth | 56 | 0.86 (0.81, 0.93) | 2.64E-05 | 2.11E-04 |
|  | Age at menarche | 181 | 0.92 (0.81, 1.05) | 0.232 | 0.571 |
| Sleep | Insomnia | 37 | 1.70 (0.91, 3.19) | 0.096 | 0.427 |

Abbreviations: BH = Benjamin and Hochberg. CI = Confidence interval. SNP = Single nucleotide polymorphism. OR = Odds ratio. HDL-c = High-density lipoprotein cholesterol-c. LDL-c = Low-density lipoprotein cholesterol-c. MD = Major depression. BMI = Body mass index. SCZ = Schizophrenia. SI = Smoking initiation. TSH = Thyrotropin.
