# Supplementary material for: Causal effects of potential risk factors on postpartum depression: a Mendelian randomization study
Source: Front Psychiatry. 2023 Dec 20;14:1275834. doi: 10.3389/fpsyt.2023.1275834 (PMC10761415; doi:10.3389/fpsyt.2023.1275834)
Supplement: Supplementary file 4 [file Table_4.docx]

**Supplementary Table 4. An overview of the heterogeneity test and pleiotropy test in MR sensitivity analysis.**

| **Potential risk factors** | **Traits** | **Weighted median** | | **MR-Egger regression** | | **Heterogeneity** | **MR-PRESSO outlier detect** | | **Pleiotropy** |
| --- | --- | --- | --- | --- | --- | --- | --- | --- | --- |
|  |  | OR (95% CI) | P Value | OR (95% CI) | *p* value |  | OR (95% CI) | *p* value |  |
| Psychiatric disorders | SCZ | 1.13 (1.06, 1.20) | 7.24E-05 | 1.05 (0.86, 1.28) | 0.656 | I2 = 45.2%; Cochrane Q = 266; *p* = 4.61E-9 | 1.13 (1.08, 1.17) | 1.54E-06 | Intercept = 0.005; *p* = 0.443 |
|  | Autism Spectrum Disorder | 1.02 (0.92, 1.13) | 0.773 | 1.03 (0.81, 1.29) | 0.833 | I2 = 15.7%; Cochrane Q = 37; *p* = 0.219 | No significant outliers | No significant outliers | Intercept = 0.001; *p* = 0.924 |
|  | Bipolar disorder | 1.06 (0.97, 1.16) | 0.190 | 1.13 (0.94, 1.36) | 0.257 | I2 = 32.6%; Cochrane Q = 7; *p* = 0.192 | No significant outliers | No significant outliers | Intercept = -0.015; *p* = 0.387 |
|  | MD | 1.97 (1.60, 2.42) | 1.32E-10 | 1.72 (0.69, 4.28) | 0.247 | I2 = 6.9%; Cochrane Q = 48; *p* = 0.340 | No significant outliers | No significant outliers | Intercept = 0.005; *p* = 0.706 |
|  | Attention deficit/hyperactivity disorder | 1.01 (0.86, 1.19) | 0.898 | 1.57 (0.89, 2.76) | 0.164 | I2 = 42.9%; Cochrane Q = 14; *p* = 0.081 | No significant outliers | No significant outliers | Intercept = -0.035; *p* = 0.243 |
|  | Anxiety | 1.00 (0.92, 1.09) | 0.956 | 1.01 (0.82, 1.24) | 0.915 | I2 = 0%; Cochrane Q = 4; *p* = 0.483 | No significant outliers | No significant outliers | Intercept = -0.003; *p* = 0.878 |
| Overweight | BMI | 1.12 (0.96, 1.31) | 0.134 | 1.14 (0.89, 1.46) | 0.307 | I2 = 24.3%; Cochrane Q = 535; *p* = 1.46E-5 | 1.12 (1.01, 1.23) | 0.022 | Intercept = -1.39E-04; *p* = 0.952 |
| Blood pressure | Diastolic blood pressure | 1.10 (0.89, 1.35) | 0.373 | 1.13 (0.76, 1.67) | 0.557 | I2 = 17.4%; Cochrane Q = 234; *p* = 0.024 | No significant outliers | No significant outliers | Intercept = -0.001; *p* = 0.830 |
|  | Pulse pressure | 1.01 (0.82, 1.25) | 0.895 | 1.27 (0.86, 1.88) | 0.232 | I2 = 18.3%; Cochrane Q = 242; *p* = 0.017 | 1.06 (0.93, 1.22) | 0.404 | Intercept = -0.003; *p* = 0.306 |
|  | Systolic blood pressure | 1.23 (1.00, 1.51) | 0.05 | 1.39 (0.97, 1.99) | 0.078 | I2 = 6.4%; Cochrane Q = 217; *p* = 0.240 | No significant outliers | No significant outliers | Intercept = -0.005; *p* = 0.102 |
| Glucose | Glycated hemoglobin | 0.79 (0.54, 1.14) | 0.204 | 0.97 (0.61, 1.54) | 0.891 | I2 = 0%; Cochrane Q = 69; *p* = 0.481 | No significant outliers | No significant outliers | Intercept = -0.002; *p* = 0.559 |
|  | Fasting glucose | 1.12 (0.83, 1.51) | 0.457 | 1.29 (0.88, 1.90) | 0.200 | I2 = 42.4%; Cochrane Q = 102; *p* = 0.0004 | 0.90 (0.71, 1.13) | 0.325 | Intercept = -0.008; *p* = 0.08 |
|  | Fasting insulin | 0.89 (0.56, 1.41) | 0.628 | 1.65 (0.56, 4.81) | 0.368 | I2 = 15.6%; Cochrane Q = 43; *p* = 0.207 | No significant outliers | No significant outliers | Intercept = -0.010; *p* = 0.285 |
|  | 2-hour glucose | 1.04 (0.88, 1.24) | 0.624 | 1.05 (0.73, 1.51) | 0.792 | I2 = 0%; Cochrane Q = 5; *p* = 0.796 | No significant outliers | No significant outliers | Intercept = 1.13E-04; *p* = 0.994 |
| Lipids | Triglycerides | 1.08 (0.97, 1.19) | 0.143 | 0.99 (0.89, 1.09) | 0.791 | I2 = 10.1%; Cochrane Q = 295; *p* = 0.101 | No significant outliers | No significant outliers | Intercept = 0.003; *p* = 0.089 |
|  | LDL-c | 1.00 (0.88, 1.14) | 0.993 | 1.02 (0.89, 1.16) | 0.818 | I2 = 36.6%; Cochrane Q = 221; *p* = 1.58E-5 | 0.93 (0.85, 1.03) | 0.122 | Intercept = -0.004; *p* = 0.109 |
|  | HDL-c | 0.98 (0.86, 1.12) | 0.814 | 0.98 (0.87, 1.10) | 0.742 | I2 = 22.3%; Cochrane Q = 376; *p* = 0.001 | No significant outliers | No significant outliers | Intercept = -4.03E-04; *p* = 0.812 |
|  | Apolipoprotein A-I | 1.00 (0.89, 1.13) | 0.991 | 0.99 (0.88, 1.13) | 0.933 | I2 = 22.5%; Cochrane Q = 323; *p* = 0.001 | No significant outliers | No significant outliers | Intercept = -2.69E-04; *p* = 0.887 |
|  | Apolipoprotein B | 1.00 (0.88, 1.13) | 0.975 | 1.02 (0.92, 1.13) | 0.660 | I2 = 29.2%; Cochrane Q = 227; *p* = 4.49E-4 | 0.96 (0.89, 1.04) | 0.246 | Intercept = -0.004; *p* = 0.082 |
| Sex-hormones | Total Testosterone | 1.13 (0.96, 1.33) | 0.132 | 0.97 (0.81, 1.16) | 0.732 | I2 = 9.3%; Cochrane Q = 104; *p* = 0.232 | No significant outliers | No significant outliers | Intercept = 2.90E-04; *p* = 0.927 |
|  | Bioavailable Testosterone | 1.12 (0.95, 1.32) | 0.164 | 1.01 (0.81, 1.26) | 0.938 | I2 = 32.4%; Cochrane Q = 160; *p* = 0.001 | 0.96 (0.87, 1.06) | 0.485 | Intercept = -0.001; *p* = 0.766 |
|  | Sex hormone binding globulin | 0.97 (0.85, 1.11) | 0.677 | 0.94 (0.83, 1.07) | 0.376 | I2 = 17.7%; Cochrane Q = 198; *p* = 0.032 | No significant outliers | No significant outliers | Intercept = 0.002; *p* = 0.508 |
|  | Oestradiol | 0.97 (0.76, 1.24) | 0.807 | 1.19 (0.83, 1.71) | 0.370 | I2 = 0%; Cochrane Q = 12; *p* = 0.642 | No significant outliers | No significant outliers | Intercept = -0.007; *p* = 0.473 |
| Thyroid function | Free thyroxine | 0.88 (0.76, 1.02) | 0.080 | 0.83 (0.57, 1.22) | 0.370 | I2 = 33.8%; Cochrane Q = 15; *p* = 0.128 | No significant outliers | No significant outliers | Intercept = 0.010; *p* = 0.567 |
|  | TSH | 1.00 (0.89, 1.11) | 0.951 | 1.10 (0.85, 1.43) | 0.462 | I2 = 15.4%; Cochrane Q = 26; *p* = 0.251 | No significant outliers | No significant outliers | Intercept = -0.006; *p* = 0.540 |
|  | incTSH/hypothyroidism | 1.01 (0.94, 1.09) | 0.764 | 1.00 (0.86, 1.16) | 0.977 | I2 = 20.7%; Cochrane Q = 9; *p* = 0.265 | No significant outliers | No significant outliers | Intercept = 0.001; *p* = 0.953 |
|  | decTSH/hyperthyroidism | 0.98 (0.93, 1.03) | 0.371 | 0.92 (0.82, 1.04) | 0.213 | I2 = 15.7%; Cochrane Q = 13; *p* = 0.29 | No significant outliers | No significant outliers | Intercept = 0.015; *p* = 0.349 |
| Inflammatory biomarkers | Serum 25-Hydroxyvitamin D levels adjusted BMI | 1.00 (0.83, 1.20) | 0.995 | 0.90 (0.73, 1.10) | 0.297 | I2 = 23.7%; Cochrane Q = 132; *p* = 0.020 | No significant outliers | No significant outliers | Intercept = 0.005; *p* = 0.093 |
|  | C-reactive protein levels | 1.02 (0.90, 1.17) | 0.738 | 1.02 (0.89, 1.16) | 0.801 | I2 = 17.4%; Cochrane Q = 278; *p* = 0.016 | 1.03 (0.95, 1.11) | 0.464 | Intercept = 0.001; *p* = 0.675 |
| Habits | SI | 1.35 (1.12, 1.63) | 0.002 | 0.68 (0.32, 1.43) | 0.310 | I2 = 30.2%; Cochrane Q = 113; *p* = 0.007 | No significant outliers | No significant outliers | Intercept = 0.018; *p* = 0.076 |
|  | Cigarettes per day | 1.03 (0.90, 1.17) | 0.709 | 0.98 (0.83, 1.17) | 0.864 | I2 = 0%; Cochrane Q = 16; *p* = 0.760 | No significant outliers | No significant outliers | Intercept = 0.006; *p* = 0.332 |
|  | Alcohol consumption | 1.28 (0.79, 2.10) | 0.320 | 0.99 (0.37, 2.63) | 0.977 | I2 = 40.1%; Cochrane Q = 58; *p* = 0.008 | 1.15 (0.79, 1.67) | 0.455 | Intercept = 0.004; *p* = 0.591 |
|  | Coffee intake | 0.93 (0.60, 1.43) | 0.733 | 1.12 (0.53, 2.34) | 0.770 | I2 = 28.7%; Cochrane Q = 49; *p* = 0.057 | No significant outliers | No significant outliers | Intercept = -0.007; *p* = 0.278 |
|  | Tea intake | 0.95 (0.65, 1.39) | 0.799 | 0.80 (0.46, 1.39) | 0.436 | I2 = 0%; Cochrane Q = 30; *p* = 0.856 | No significant outliers | No significant outliers | Intercept = 0.004; *p* = 0.439 |
| Socioeconomic Factors | Years of schooling | 0.57 (0.47, 0.70) | 1.20E-07 | 0.54 (0.29, 1.00) | 0.051 | I2 = 35.7%; Cochrane Q = 455; P = 3.53E-9 | 0.57 (0.48, 0.66) | 5.21E-12 | Intercept = 0.001; *p* = 0.899 |
|  | Average total household income before tax | 0.62 (0.42, 0.92) | 0.017 | 0.73 (0.11, 4.97) | 0.746 | I2 = 51.3%; Cochrane Q = 80; P = 1.17E-4 | 0.67 (0.48, 0.94) | 0.023 | Intercept = -0.003; *p* = 0.875 |
|  | Age at first sexual intercourse | 0.52 (0.41, 0.66) | 7.33E-08 | 0.39 (0.17, 0.88) | 0.025 | I2 = 26.2%; Cochrane Q = 217; *p* = 0.002 | No significant outliers | No significant outliers | Intercept = 0.003; *p* = 0.634 |
|  | Age at first birth | 0.90 (0.82, 0.98) | 0.017 | 0.70 (0.53, 0.92) | 0.014 | I2 = 20.8%; Cochrane Q = 69; *p* = 0.091 | No significant outliers | No significant outliers | Intercept = 0.015; *p* = 0.125 |
|  | Age at menarche | 0.98 (0.81, 1.18) | 0.799 | 1.12 (0.76, 1.64) | 0.562 | I2 = 23.7%; Cochrane Q = 236; *p* = 0.003 | No significant outliers | No significant outliers | Intercept = -0.004; *p* = 0.288 |
| Sleep | Insomnia | 1.34 (0.65, 2.78) | 0.425 | 1.06 (0.15, 7.51) | 0.951 | I2 = 45.5%; Cochrane Q = 66; *p* = 0.002 | No significant outliers | No significant outliers | Intercept = 0.005; *p* = 0.621 |

Abbreviations: CI = Confidence interval. SNP = Single nucleotide polymorphism. OR = Odds ratio. HDL-c = High-density lipoprotein cholesterol-c. LDL-c = Low-density lipoprotein cholesterol-c. MD = Major depression. BMI = Body mass index. SCZ = Schizophrenia. SI = Smoking initiation. TSH = Thyrotropin.
