# Supplementary material for: Causal effects of potential risk factors on postpartum depression: a Mendelian randomization study
Source: Front Psychiatry. 2023 Dec 20;14:1275834. doi: 10.3389/fpsyt.2023.1275834 (PMC10761415; doi:10.3389/fpsyt.2023.1275834)
Supplement: Supplementary file 5 [file Table_5.docx]

**Supplementary table 5. IVs for MVMR analysis.**

| **SNP** | **exposure** | **effect_allele** | **other_allele** | **eaf** | **beta** | **se** | **pval** |
| --- | --- | --- | --- | --- | --- | --- | --- |
| rs1000237 | Age at first birth | A | T | NA | -0.0262 | 0.0094 | 0.005449 |
| rs10073890 | Age at first birth | G | A | NA | -0.022 | 0.0107 | 0.03983 |
| rs10160769 | Age at first birth | C | G | NA | 0.0501 | 0.011 | 5.36E-06 |
| rs1017529 | Age at first birth | A | C | NA | 0.0047 | 0.0125 | 0.7052 |
| rs10423928 | Age at first birth | A | T | NA | 0.0025 | 0.0116 | 0.8271 |
| rs10505836 | Age at first birth | C | A | NA | -0.0239 | 0.0131 | 0.068431 |
| rs10510025 | Age at first birth | T | C | NA | -0.0482 | 0.0105 | 4.20E-06 |
| rs1064213 | Age at first birth | A | G | NA | -0.0209 | 0.0091 | 0.02122 |
| rs10742752 | Age at first birth | C | T | NA | -0.0051 | 0.0093 | 0.5777 |
| rs10752613 | Age at first birth | A | T | NA | -0.0715 | 0.0102 | 1.89E-12 |
| rs10765775 | Age at first birth | A | G | NA | 0.0464 | 0.0095 | 1.05E-06 |
| rs10773002 | Age at first birth | T | A | NA | -0.0349 | 0.0106 | 0.001043 |
| rs10797055 | Age at first birth | G | A | NA | 0.0108 | 0.0096 | 0.2601 |
| rs10858054 | Age at first birth | T | G | NA | 9.00E-04 | 0.0121 | 0.941 |
| rs10861176 | Age at first birth | A | G | NA | 0.0144 | 0.0105 | 0.1709 |
| rs10887578 | Age at first birth | C | G | NA | -0.0086 | 0.0093 | 0.3564 |
| rs10887801 | Age at first birth | T | G | NA | 0.0139 | 0.0092 | 0.1321 |
| rs10922907 | Age at first birth | T | A | NA | 0.0468 | 0.0093 | 4.94E-07 |
| rs10938398 | Age at first birth | A | G | NA | 0.0063 | 0.0094 | 0.5014 |
| rs10963297 | Age at first birth | G | C | NA | 0.0338 | 0.0105 | 0.001312 |
| rs11000993 | Age at first birth | C | T | NA | 0.0026 | 0.0142 | 0.8556 |
| rs11012732 | Age at first birth | G | A | NA | -0.0341 | 0.01 | 0.000638 |
| rs1105307 | Age at first birth | A | G | NA | -0.0249 | 0.0103 | 0.01579 |
| rs11079849 | Age at first birth | T | C | NA | 0.0415 | 0.01 | 3.28E-05 |
| rs11081529 | Age at first birth | C | T | NA | -0.0595 | 0.0099 | 1.81E-09 |
| rs11099020 | Age at first birth | T | C | NA | -0.0157 | 0.0097 | 0.106 |
| rs11134679 | Age at first birth | G | A | NA | -0.0173 | 0.0098 | 0.07905 |
| rs11165643 | Age at first birth | T | C | NA | -0.0065 | 0.0092 | 0.483499 |
| rs11250094 | Age at first birth | C | G | NA | 0.0391 | 0.0092 | 2.29E-05 |
| rs112633616 | Age at first birth | C | A | NA | 0.0472 | 0.0273 | 0.083339 |
| rs112687095 | Age at first birth | A | G | NA | 0.0198 | 0.0135 | 0.1424 |
| rs113338260 | Age at first birth | C | T | NA | 0.0033 | 0.0118 | 0.781601 |
| rs113520408 | Age at first birth | A | G | NA | 0.0213 | 0.0104 | 0.04055 |
| rs113624107 | Age at first birth | A | G | NA | -0.0047 | 0.0109 | 0.6692 |
| rs1143770 | Age at first birth | T | C | NA | 0.0151 | 0.0092 | 0.1004 |
| rs115000530 | Age at first birth | T | A | NA | 0.0587 | 0.0209 | 0.005005 |
| rs115454970 | Age at first birth | T | G | NA | -0.0246 | 0.0109 | 0.02489 |
| rs11587347 | Age at first birth | G | C | NA | -0.005 | 0.0158 | 0.753401 |
| rs11620355 | Age at first birth | A | G | NA | 0.0197 | 0.0163 | 0.2274 |
| rs11635092 | Age at first birth | A | G | NA | -0.0222 | 0.0095 | 0.01866 |
| rs116377258 | Age at first birth | G | A | NA | -0.0059 | 0.0273 | 0.8281 |
| rs11693094 | Age at first birth | T | C | NA | -0.0033 | 0.0093 | 0.723401 |
| rs11696755 | Age at first birth | C | T | NA | 0.0168 | 0.0118 | 0.1561 |
| rs11709402 | Age at first birth | G | A | NA | -0.0112 | 0.0101 | 0.2651 |
| rs117118217 | Age at first birth | C | G | NA | 0.0113 | 0.0427 | 0.7915 |
| rs11732657 | Age at first birth | A | G | NA | -0.0078 | 0.0108 | 0.4669 |
| rs118136827 | Age at first birth | T | G | NA | -0.0121 | 0.0104 | 0.2434 |
| rs11866420 | Age at first birth | G | C | NA | -0.0319 | 0.0098 | 0.001084 |
| rs1198588 | Age at first birth | T | A | NA | 0.0161 | 0.0111 | 0.146 |
| rs12001437 | Age at first birth | C | T | NA | 0.0018 | 0.0096 | 0.8545 |
| rs12033257 | Age at first birth | G | A | NA | 0.0263 | 0.0095 | 0.005571 |
| rs12140153 | Age at first birth | T | G | NA | 0.0382 | 0.0167 | 0.02249 |
| rs12149660 | Age at first birth | A | G | NA | 0.0428 | 0.0146 | 0.003337 |
| rs12156160 | Age at first birth | G | A | NA | -0.01 | 0.0136 | 0.4629 |
| rs12204714 | Age at first birth | T | C | NA | 0.0804 | 0.0094 | 1.08E-17 |
| rs12293670 | Age at first birth | G | A | NA | -0.0067 | 0.0098 | 0.4934 |
| rs12303743 | Age at first birth | C | G | NA | 0.0171 | 0.0152 | 0.26 |
| rs12364470 | Age at first birth | G | T | NA | 0.0089 | 0.0125 | 0.4763 |
| rs12375949 | Age at first birth | C | T | NA | 0.0202 | 0.0094 | 0.03129 |
| rs12375985 | Age at first birth | A | G | NA | 0.0145 | 0.0096 | 0.1319 |
| rs12427047 | Age at first birth | T | C | NA | 0.0219 | 0.0103 | 0.03421 |
| rs12519073 | Age at first birth | T | C | NA | -0.0156 | 0.0107 | 0.1468 |
| rs12523398 | Age at first birth | A | T | NA | 0.0719 | 0.0127 | 1.32E-08 |
| rs12643771 | Age at first birth | T | C | NA | 0.0432 | 0.0104 | 3.06E-05 |
| rs1266874 | Age at first birth | G | A | NA | -0.0051 | 0.0096 | 0.5903 |
| rs12681792 | Age at first birth | A | C | NA | -0.0184 | 0.0114 | 0.1053 |
| rs12682775 | Age at first birth | C | T | NA | 0.0103 | 0.0109 | 0.3444 |
| rs12712510 | Age at first birth | C | T | NA | 0.0084 | 0.0091 | 0.358 |
| rs12714592 | Age at first birth | C | A | NA | -0.0424 | 0.0101 | 2.67E-05 |
| rs12714702 | Age at first birth | G | A | NA | -0.021 | 0.0124 | 0.09039 |
| rs12757779 | Age at first birth | A | G | NA | 0.0092 | 0.0109 | 0.3945 |
| rs12762034 | Age at first birth | C | T | NA | -0.0299 | 0.0168 | 0.074931 |
| rs1286058 | Age at first birth | A | T | NA | -0.0203 | 0.0101 | 0.04483 |
| rs12881629 | Age at first birth | G | A | NA | 0.0037 | 0.0175 | 0.8316 |
| rs12907546 | Age at first birth | A | G | NA | -0.052 | 0.0112 | 3.73E-06 |
| rs12919291 | Age at first birth | C | G | NA | -0.0153 | 0.0118 | 0.1976 |
| rs12940014 | Age at first birth | C | T | NA | 0.008 | 0.0095 | 0.3994 |
| rs12955211 | Age at first birth | A | T | NA | 0.0325 | 0.0097 | 0.000806 |
| rs12956148 | Age at first birth | A | C | NA | 0.0067 | 0.0103 | 0.5178 |
| rs1296328 | Age at first birth | C | A | NA | 0.0102 | 0.0094 | 0.2827 |
| rs12977787 | Age at first birth | A | G | NA | -0.0121 | 0.0095 | 0.2062 |
| rs13030994 | Age at first birth | A | G | NA | 0.0048 | 0.0092 | 0.6051 |
| rs13037326 | Age at first birth | T | C | NA | -0.0307 | 0.0103 | 0.002939 |
| rs13090388 | Age at first birth | T | C | NA | 0.0762 | 0.01 | 2.91E-14 |
| rs13107325 | Age at first birth | T | C | NA | -0.0293 | 0.019 | 0.1241 |
| rs13141210 | Age at first birth | T | C | NA | 0.02 | 0.0093 | 0.03101 |
| rs13145650 | Age at first birth | T | C | NA | -0.0312 | 0.0169 | 0.064991 |
| rs13175535 | Age at first birth | A | G | NA | 0.0143 | 0.0101 | 0.1577 |
| rs13195636 | Age at first birth | C | A | NA | -0.0049 | 0.0151 | 0.744201 |
| rs1320251 | Age at first birth | T | C | NA | 0.0339 | 0.0094 | 0.000309 |
| rs13218383 | Age at first birth | G | C | NA | 0.031 | 0.0095 | 0.001103 |
| rs13233308 | Age at first birth | T | C | NA | -9.00E-04 | 0.0093 | 0.9223 |
| rs13248187 | Age at first birth | C | T | NA | -0.0016 | 0.0103 | 0.8775 |
| rs13261666 | Age at first birth | T | G | NA | 0.0116 | 0.009 | 0.1984 |
| rs1327259 | Age at first birth | G | A | NA | 0.0105 | 0.0093 | 0.261 |
| rs13292699 | Age at first birth | C | A | NA | 0.0225 | 0.0093 | 0.01623 |
| rs1330199 | Age at first birth | T | G | NA | 0.0153 | 0.0093 | 0.1025 |
| rs13307225 | Age at first birth | A | G | NA | 0.0193 | 0.0158 | 0.2221 |
| rs13317303 | Age at first birth | A | C | NA | 0.0184 | 0.0131 | 0.1579 |
| rs1334297 | Age at first birth | A | G | NA | 0.03 | 0.0102 | 0.003278 |
| rs13422673 | Age at first birth | T | C | NA | -0.0099 | 0.0091 | 0.2779 |
| rs13427822 | Age at first birth | G | A | NA | 0.0274 | 0.0104 | 0.0087 |
| rs1346841 | Age at first birth | A | G | NA | -0.0086 | 0.0094 | 0.361 |
| rs1360201 | Age at first birth | T | C | NA | -0.0083 | 0.009 | 0.3563 |
| rs1363862 | Age at first birth | A | G | NA | -0.0274 | 0.0105 | 0.009108 |
| rs13642 | Age at first birth | T | A | NA | -9.00E-04 | 0.0094 | 0.9274 |
| rs1391438 | Age at first birth | C | T | NA | -0.0034 | 0.0098 | 0.7247 |
| rs1392816 | Age at first birth | T | C | NA | 0.0419 | 0.0093 | 7.30E-06 |
| rs140159717 | Age at first birth | T | C | NA | 0.0402 | 0.0193 | 0.03712 |
| rs1438945 | Age at first birth | A | T | NA | -0.0135 | 0.0103 | 0.1916 |
| rs1441264 | Age at first birth | A | G | NA | -0.0058 | 0.0094 | 0.538 |
| rs1450782 | Age at first birth | G | T | NA | -0.0256 | 0.0092 | 0.005587 |
| rs1455350 | Age at first birth | A | T | NA | -0.0378 | 0.0092 | 4.37E-05 |
| rs1458156 | Age at first birth | T | C | NA | 0.018 | 0.0091 | 0.04721 |
| rs1471740 | Age at first birth | C | T | NA | -0.0172 | 0.0111 | 0.1223 |
| rs147568678 | Age at first birth | C | T | NA | -0.0037 | 0.0113 | 0.7426 |
| rs1477290 | Age at first birth | C | T | NA | 0.0322 | 0.0136 | 0.01734 |
| rs1503526 | Age at first birth | C | T | NA | -0.0228 | 0.009 | 0.01116 |
| rs152603 | Age at first birth | G | A | NA | 0.0188 | 0.0094 | 0.04483 |
| rs1565735 | Age at first birth | A | T | NA | 0.0104 | 0.0115 | 0.3648 |
| rs1566085 | Age at first birth | T | G | NA | 0.032 | 0.0096 | 0.00085 |
| rs1582931 | Age at first birth | A | G | NA | -0.0261 | 0.0091 | 0.004144 |
| rs1584469 | Age at first birth | T | C | NA | -0.0197 | 0.0098 | 0.04599 |
| rs1609010 | Age at first birth | G | A | NA | -0.0013 | 0.0093 | 0.8922 |
| rs1671770 | Age at first birth | C | A | NA | -0.0216 | 0.0126 | 0.08623 |
| rs16846140 | Age at first birth | G | A | NA | -0.0308 | 0.0096 | 0.001282 |
| rs16846463 | Age at first birth | G | A | NA | -0.0585 | 0.0152 | 0.000124 |
| rs16854920 | Age at first birth | C | T | NA | 0.0138 | 0.0099 | 0.1628 |
| rs1689510 | Age at first birth | C | G | NA | 0.0588 | 0.0097 | 1.64E-09 |
| rs16916303 | Age at first birth | G | A | NA | 0.015 | 0.0143 | 0.2929 |
| rs16995054 | Age at first birth | T | C | NA | -0.0269 | 0.0112 | 0.01588 |
| rs17193211 | Age at first birth | T | C | NA | 0.0271 | 0.0183 | 0.1385 |
| rs17194490 | Age at first birth | T | G | NA | 0.0167 | 0.0125 | 0.1807 |
| rs17399739 | Age at first birth | G | A | NA | -0.0298 | 0.0177 | 0.09177 |
| rs17565975 | Age at first birth | A | G | NA | -0.0374 | 0.0092 | 4.74E-05 |
| rs17598675 | Age at first birth | C | T | NA | 0.0188 | 0.0091 | 0.03772 |
| rs176218 | Age at first birth | T | G | NA | 0.0528 | 0.0114 | 3.76E-06 |
| rs1778830 | Age at first birth | A | G | NA | -0.0189 | 0.0097 | 0.05229 |
| rs1788808 | Age at first birth | G | A | NA | 0.019 | 0.0092 | 0.03876 |
| rs1834144 | Age at first birth | A | C | NA | 0.0065 | 0.0096 | 0.498799 |
| rs1860002 | Age at first birth | T | C | NA | -0.0018 | 0.0094 | 0.8507 |
| rs1899896 | Age at first birth | T | C | NA | -0.0377 | 0.0098 | 0.000117 |
| rs1901512 | Age at first birth | C | T | NA | -0.0147 | 0.01 | 0.1387 |
| rs1915019 | Age at first birth | G | A | NA | -0.0082 | 0.0106 | 0.4406 |
| rs1919243 | Age at first birth | C | T | NA | -0.0131 | 0.0094 | 0.1637 |
| rs1950829 | Age at first birth | G | A | NA | 0.0061 | 0.0091 | 0.4987 |
| rs1967772 | Age at first birth | A | G | NA | 0.0022 | 0.01 | 0.8255 |
| rs2035936 | Age at first birth | T | G | NA | -0.083 | 0.0202 | 4.06E-05 |
| rs2076603 | Age at first birth | A | G | NA | 0.0096 | 0.0099 | 0.3289 |
| rs2084572 | Age at first birth | G | A | NA | 0.0271 | 0.0091 | 0.00293 |
| rs2102278 | Age at first birth | G | A | NA | -0.0183 | 0.01 | 0.06705 |
| rs2133561 | Age at first birth | T | A | NA | -4.00E-04 | 0.01 | 0.9659 |
| rs213518 | Age at first birth | C | T | NA | -3.00E-04 | 0.013 | 0.9834 |
| rs2153740 | Age at first birth | G | A | NA | 0.0128 | 0.0091 | 0.1593 |
| rs215634 | Age at first birth | G | A | NA | 0.0142 | 0.0094 | 0.1308 |
| rs2172131 | Age at first birth | C | T | NA | 0.0165 | 0.0097 | 0.09013 |
| rs217336 | Age at first birth | A | C | NA | 0.0099 | 0.0093 | 0.2835 |
| rs2174752 | Age at first birth | T | G | NA | -0.01 | 0.0091 | 0.2693 |
| rs2176337 | Age at first birth | T | A | NA | -0.0142 | 0.01 | 0.1533 |
| rs217672 | Age at first birth | C | A | NA | -0.0177 | 0.0107 | 0.097201 |
| rs2214123 | Age at first birth | G | A | NA | 0.0057 | 0.0098 | 0.559099 |
| rs2234458 | Age at first birth | T | C | NA | 0.0285 | 0.0099 | 0.00387 |
| rs2253310 | Age at first birth | G | C | NA | -0.0133 | 0.0093 | 0.1528 |
| rs2281819 | Age at first birth | A | T | NA | 0.0152 | 0.0108 | 0.1572 |
| rs2283076 | Age at first birth | G | A | NA | -0.0432 | 0.0109 | 7.19E-05 |
| rs2289379 | Age at first birth | T | C | NA | 0.0172 | 0.0095 | 0.069459 |
| rs2302761 | Age at first birth | T | C | NA | 0.0027 | 0.0115 | 0.8136 |
| rs2306593 | Age at first birth | T | C | NA | 0.0248 | 0.0094 | 0.008212 |
| rs2307111 | Age at first birth | C | T | NA | -0.0089 | 0.0093 | 0.341 |
| rs2332700 | Age at first birth | G | C | NA | -0.008 | 0.0107 | 0.4506 |
| rs2333321 | Age at first birth | G | A | NA | 0.0526 | 0.0112 | 2.85E-06 |
| rs2342892 | Age at first birth | G | T | NA | 0.0237 | 0.009 | 0.008677 |
| rs2347526 | Age at first birth | C | T | NA | 0.0344 | 0.01 | 0.000583 |
| rs2396625 | Age at first birth | A | T | NA | 0.0309 | 0.0094 | 0.001029 |
| rs2398861 | Age at first birth | G | A | NA | -0.038 | 0.0105 | 0.000307 |
| rs240963 | Age at first birth | C | T | NA | 0.0353 | 0.0126 | 0.004949 |
| rs242093 | Age at first birth | A | G | NA | -0.0246 | 0.0095 | 0.009309 |
| rs2433733 | Age at first birth | A | G | NA | -0.0168 | 0.0097 | 0.0816 |
| rs2456020 | Age at first birth | T | C | NA | -0.0017 | 0.0109 | 0.8798 |
| rs2482356 | Age at first birth | C | T | NA | -6.00E-04 | 0.0091 | 0.9446 |
| rs252761 | Age at first birth | T | G | NA | 0.0206 | 0.0095 | 0.03115 |
| rs2554835 | Age at first birth | A | G | NA | 0.0116 | 0.0098 | 0.2389 |
| rs2568958 | Age at first birth | A | G | NA | 0.0017 | 0.0093 | 0.859 |
| rs2606228 | Age at first birth | C | A | NA | 0.0216 | 0.0098 | 0.02691 |
| rs2612030 | Age at first birth | C | T | NA | 0.0449 | 0.0124 | 0.000301 |
| rs2616143 | Age at first birth | A | G | NA | 0.018 | 0.0096 | 0.06163 |
| rs266047 | Age at first birth | A | G | NA | 0.0533 | 0.0092 | 6.70E-09 |
| rs2678204 | Age at first birth | G | T | NA | -0.0282 | 0.0097 | 0.003692 |
| rs2781668 | Age at first birth | T | C | NA | 0.0054 | 0.0122 | 0.6595 |
| rs2787101 | Age at first birth | T | C | NA | 0.0241 | 0.0095 | 0.01105 |
| rs2819336 | Age at first birth | C | T | NA | -0.0295 | 0.0096 | 0.002136 |
| rs28373063 | Age at first birth | C | G | NA | 0.0453 | 0.0124 | 0.000267 |
| rs28404639 | Age at first birth | T | C | NA | -0.0104 | 0.0096 | 0.2821 |
| rs28489620 | Age at first birth | A | G | NA | 0.0038 | 0.0106 | 0.7177 |
| rs28513670 | Age at first birth | G | A | NA | 0.025 | 0.0121 | 0.03828 |
| rs2875762 | Age at first birth | C | G | NA | -0.0082 | 0.0107 | 0.4442 |
| rs2876520 | Age at first birth | G | C | NA | -0.0264 | 0.0094 | 0.004931 |
| rs2920503 | Age at first birth | T | C | NA | 0.0304 | 0.0105 | 0.003741 |
| rs2923431 | Age at first birth | C | G | NA | 0.0193 | 0.0096 | 0.04523 |
| rs293566 | Age at first birth | C | T | NA | -0.0694 | 0.0099 | 2.18E-12 |
| rs2962334 | Age at first birth | T | G | NA | 0.0069 | 0.0316 | 0.8285 |
| rs2971970 | Age at first birth | G | T | NA | 0.036 | 0.011 | 0.001026 |
| rs301800 | Age at first birth | C | T | NA | -0.0361 | 0.012 | 0.002532 |
| rs30266 | Age at first birth | A | G | NA | -0.022 | 0.0099 | 0.02629 |
| rs317656 | Age at first birth | A | T | NA | 0.0146 | 0.0104 | 0.159 |
| rs320693 | Age at first birth | C | G | NA | 0.0297 | 0.0092 | 0.001241 |
| rs3211995 | Age at first birth | A | G | NA | 0.0225 | 0.0133 | 0.09155 |
| rs329118 | Age at first birth | T | C | NA | 0.0495 | 0.0096 | 2.33E-07 |
| rs34025316 | Age at first birth | T | C | NA | 0.0058 | 0.0101 | 0.5662 |
| rs34045288 | Age at first birth | T | C | NA | -3.00E-04 | 0.0098 | 0.9739 |
| rs34234296 | Age at first birth | A | G | NA | -0.0102 | 0.01 | 0.3036 |
| rs34481751 | Age at first birth | A | C | NA | 8.00E-04 | 0.013 | 0.9506 |
| rs34517439 | Age at first birth | A | C | NA | -0.0799 | 0.0149 | 8.11E-08 |
| rs347551 | Age at first birth | G | C | NA | 0.0051 | 0.0099 | 0.6074 |
| rs34811474 | Age at first birth | A | G | NA | 0.0326 | 0.0117 | 0.005469 |
| rs35154326 | Age at first birth | G | A | NA | 0.0272 | 0.0107 | 0.01144 |
| rs35309068 | Age at first birth | G | T | NA | 0.0393 | 0.0093 | 2.34E-05 |
| rs354155 | Age at first birth | C | G | NA | 0.0179 | 0.0155 | 0.2461 |
| rs35417702 | Age at first birth | T | C | NA | -0.0271 | 0.0092 | 0.003251 |
| rs35475880 | Age at first birth | T | G | NA | -0.0284 | 0.0113 | 0.01163 |
| rs35532491 | Age at first birth | T | A | NA | 0.0433 | 0.0153 | 0.004528 |
| rs355777 | Age at first birth | C | G | NA | -0.0226 | 0.0092 | 0.01387 |
| rs35867081 | Age at first birth | G | A | NA | 0.0236 | 0.0096 | 0.01414 |
| rs36007635 | Age at first birth | A | G | NA | 0.0125 | 0.0134 | 0.3519 |
| rs36061954 | Age at first birth | T | C | NA | -0.0394 | 0.0097 | 4.64E-05 |
| rs36119825 | Age at first birth | A | G | NA | 0.0197 | 0.0093 | 0.03317 |
| rs363096 | Age at first birth | C | T | NA | 0.041 | 0.0093 | 9.43E-06 |
| rs3747631 | Age at first birth | C | G | NA | 0.0228 | 0.0113 | 0.04406 |
| rs3764625 | Age at first birth | G | T | NA | -0.0247 | 0.0094 | 0.008631 |
| rs3770754 | Age at first birth | G | C | NA | 0.006 | 0.0097 | 0.5377 |
| rs3784710 | Age at first birth | C | T | NA | 0.0257 | 0.0109 | 0.01797 |
| rs3800546 | Age at first birth | G | C | NA | -0.02 | 0.0103 | 0.05207 |
| rs3807865 | Age at first birth | A | G | NA | -0.0172 | 0.0093 | 0.065021 |
| rs3809634 | Age at first birth | G | A | NA | 0.0161 | 0.0101 | 0.1118 |
| rs3814883 | Age at first birth | T | C | NA | -0.0031 | 0.0094 | 0.746 |
| rs3845344 | Age at first birth | T | C | NA | -0.0373 | 0.0093 | 5.89E-05 |
| rs3851998 | Age at first birth | G | C | NA | 0.0324 | 0.0106 | 0.002315 |
| rs3896224 | Age at first birth | G | A | NA | 0.0494 | 0.0093 | 9.50E-08 |
| rs3897821 | Age at first birth | G | A | NA | -0.0337 | 0.0096 | 0.000464 |
| rs3901286 | Age at first birth | A | C | NA | 0.0538 | 0.0128 | 2.71E-05 |
| rs394608 | Age at first birth | C | T | NA | -0.0492 | 0.0093 | 1.42E-07 |
| rs40071 | Age at first birth | C | T | NA | 0.0128 | 0.0118 | 0.2799 |
| rs4044321 | Age at first birth | G | A | NA | 0.0286 | 0.0097 | 0.003022 |
| rs4055791 | Age at first birth | T | C | NA | 0.019 | 0.0093 | 0.04203 |
| rs406388 | Age at first birth | G | C | NA | -0.0174 | 0.0123 | 0.1586 |
| rs4148155 | Age at first birth | G | A | NA | 0.0151 | 0.0144 | 0.2942 |
| rs4261944 | Age at first birth | G | T | NA | -0.0184 | 0.0094 | 0.05106 |
| rs4267103 | Age at first birth | C | T | NA | -0.0109 | 0.012 | 0.3633 |
| rs429343 | Age at first birth | G | A | NA | 0.0136 | 0.0091 | 0.138 |
| rs429358 | Age at first birth | C | T | NA | 0.0112 | 0.0131 | 0.392 |
| rs4328757 | Age at first birth | T | C | NA | 0.0326 | 0.0093 | 0.000466 |
| rs4352658 | Age at first birth | T | C | NA | -0.0538 | 0.0164 | 0.001026 |
| rs4382592 | Age at first birth | G | T | NA | 0.0311 | 0.0099 | 0.001608 |
| rs4419475 | Age at first birth | T | A | NA | -0.0314 | 0.0094 | 0.000816 |
| rs4439537 | Age at first birth | C | T | NA | 0.0291 | 0.0093 | 0.001707 |
| rs4477562 | Age at first birth | T | C | NA | -0.0076 | 0.0136 | 0.577301 |
| rs4482463 | Age at first birth | A | C | NA | 0.0067 | 0.017 | 0.6945 |
| rs4613074 | Age at first birth | C | T | NA | 0.0177 | 0.0122 | 0.1455 |
| rs4648450 | Age at first birth | A | C | NA | 8.00E-04 | 0.0095 | 0.9317 |
| rs4653164 | Age at first birth | T | C | NA | -0.0219 | 0.01 | 0.02783 |
| rs4671328 | Age at first birth | G | T | NA | 0.0242 | 0.0093 | 0.009244 |
| rs4700393 | Age at first birth | G | A | NA | 0.025 | 0.0092 | 0.006528 |
| rs4702 | Age at first birth | A | G | NA | 0.0395 | 0.0095 | 3.40E-05 |
| rs4709807 | Age at first birth | C | T | NA | -0.0357 | 0.011 | 0.00112 |
| rs4737188 | Age at first birth | T | A | NA | -0.0136 | 0.0091 | 0.1328 |
| rs4757144 | Age at first birth | A | G | NA | -0.0217 | 0.0092 | 0.01888 |
| rs4757957 | Age at first birth | C | G | NA | 0.0368 | 0.0098 | 0.000169 |
| rs4764949 | Age at first birth | G | A | NA | -0.0051 | 0.0096 | 0.5942 |
| rs4790292 | Age at first birth | A | C | NA | 0.0394 | 0.0133 | 0.002932 |
| rs4810227 | Age at first birth | A | G | NA | 0.0323 | 0.0098 | 0.000974 |
| rs4812325 | Age at first birth | A | G | NA | -0.0011 | 0.0096 | 0.9088 |
| rs4832298 | Age at first birth | T | C | NA | 0.014 | 0.0102 | 0.1702 |
| rs4846724 | Age at first birth | A | G | NA | 0.0163 | 0.009 | 0.07169 |
| rs4876611 | Age at first birth | G | A | NA | -0.0047 | 0.0102 | 0.6466 |
| rs4895799 | Age at first birth | T | C | NA | 0.0133 | 0.0092 | 0.1484 |
| rs4958702 | Age at first birth | C | T | NA | -0.0058 | 0.0094 | 0.5383 |
| rs4961705 | Age at first birth | C | G | NA | 0.02 | 0.0096 | 0.03624 |
| rs512121 | Age at first birth | C | T | NA | -0.0117 | 0.0118 | 0.3186 |
| rs539515 | Age at first birth | C | A | NA | -0.0306 | 0.0115 | 0.007687 |
| rs55658481 | Age at first birth | A | G | NA | -0.0048 | 0.0099 | 0.6259 |
| rs55707359 | Age at first birth | G | T | NA | 0.0287 | 0.0406 | 0.4801 |
| rs55714539 | Age at first birth | C | A | NA | -0.0137 | 0.0102 | 0.1763 |
| rs55726687 | Age at first birth | A | G | NA | 0.0098 | 0.0115 | 0.3971 |
| rs55736314 | Age at first birth | G | C | NA | 0.0171 | 0.0094 | 0.06903 |
| rs558887 | Age at first birth | G | A | NA | 0.0274 | 0.0101 | 0.006466 |
| rs56094641 | Age at first birth | G | A | NA | 0.0131 | 0.0094 | 0.1639 |
| rs56133507 | Age at first birth | G | T | NA | -0.0202 | 0.0116 | 0.0809 |
| rs56161855 | Age at first birth | T | A | NA | -0.0339 | 0.0134 | 0.01171 |
| rs56203622 | Age at first birth | C | T | NA | -0.0228 | 0.0131 | 0.0823 |
| rs56335113 | Age at first birth | G | A | NA | -0.0177 | 0.0104 | 0.08711 |
| rs56356382 | Age at first birth | C | T | NA | 0.0195 | 0.0124 | 0.1155 |
| rs56391344 | Age at first birth | A | G | NA | 0.0194 | 0.0109 | 0.0758 |
| rs56399737 | Age at first birth | T | C | NA | 0.0103 | 0.0093 | 0.2672 |
| rs56858768 | Age at first birth | A | G | NA | -0.0212 | 0.01 | 0.03447 |
| rs57636386 | Age at first birth | C | T | NA | -0.0276 | 0.0166 | 0.09581 |
| rs58120505 | Age at first birth | C | T | NA | -0.0174 | 0.0096 | 0.0688 |
| rs59086897 | Age at first birth | A | T | NA | -0.0213 | 0.0096 | 0.02601 |
| rs59237168 | Age at first birth | C | T | NA | -6.00E-04 | 0.0112 | 0.9546 |
| rs5995843 | Age at first birth | G | A | NA | -0.0195 | 0.0095 | 0.03908 |
| rs60764613 | Age at first birth | T | G | NA | -0.0183 | 0.013 | 0.1592 |
| rs6123924 | Age at first birth | G | A | NA | -0.0262 | 0.0128 | 0.04154 |
| rs6134916 | Age at first birth | T | C | NA | 5.00E-04 | 0.009 | 0.9529 |
| rs61813324 | Age at first birth | T | C | NA | -0.0396 | 0.0149 | 0.007847 |
| rs61828088 | Age at first birth | A | G | NA | 0.0342 | 0.0148 | 0.02043 |
| rs61903695 | Age at first birth | G | A | NA | -0.0241 | 0.0107 | 0.02431 |
| rs61914045 | Age at first birth | A | G | NA | -0.023 | 0.0117 | 0.04966 |
| rs62097985 | Age at first birth | T | C | NA | -0.0289 | 0.0093 | 0.001966 |
| rs62107261 | Age at first birth | C | T | NA | 0.0429 | 0.0236 | 0.06907 |
| rs62134195 | Age at first birth | T | C | NA | 0.0627 | 0.0238 | 0.008406 |
| rs62176243 | Age at first birth | T | A | NA | 0.0529 | 0.0106 | 6.63E-07 |
| rs62176993 | Age at first birth | A | G | NA | -0.0218 | 0.0095 | 0.02149 |
| rs62190049 | Age at first birth | C | G | NA | 0.029 | 0.0098 | 0.003214 |
| rs62439690 | Age at first birth | A | G | NA | -0.0292 | 0.0109 | 0.00737 |
| rs6265 | Age at first birth | T | C | NA | 0.0167 | 0.0118 | 0.1557 |
| rs6531639 | Age at first birth | A | G | NA | 0.0061 | 0.0114 | 0.5943 |
| rs6545714 | Age at first birth | A | G | NA | -0.0147 | 0.0092 | 0.1111 |
| rs6546857 | Age at first birth | G | A | NA | -0.0175 | 0.0105 | 0.09679 |
| rs6560906 | Age at first birth | C | T | NA | 0.0051 | 0.0109 | 0.6402 |
| rs6567160 | Age at first birth | C | T | NA | -0.0016 | 0.0106 | 0.8798 |
| rs6575340 | Age at first birth | A | G | NA | -0.0377 | 0.0095 | 6.89E-05 |
| rs66511648 | Age at first birth | C | T | NA | -0.027 | 0.0105 | 0.01036 |
| rs66679256 | Age at first birth | T | C | NA | -0.0143 | 0.0093 | 0.1256 |
| rs6669341 | Age at first birth | G | A | NA | 0.0103 | 0.0093 | 0.2679 |
| rs6682438 | Age at first birth | C | T | NA | -0.0077 | 0.0096 | 0.4228 |
| rs6688826 | Age at first birth | C | T | NA | -0.0045 | 0.0099 | 0.6475 |
| rs6707827 | Age at first birth | G | A | NA | 0.0027 | 0.0102 | 0.7938 |
| rs6710091 | Age at first birth | G | C | NA | -0.0144 | 0.0094 | 0.1259 |
| rs6719762 | Age at first birth | C | T | NA | -0.0415 | 0.0092 | 6.63E-06 |
| rs6731967 | Age at first birth | C | G | NA | -0.0193 | 0.0106 | 0.068431 |
| rs6744646 | Age at first birth | G | A | NA | -0.0417 | 0.012 | 0.000536 |
| rs6744794 | Age at first birth | G | C | NA | -0.0424 | 0.0095 | 8.22E-06 |
| rs6752979 | Age at first birth | A | G | NA | -0.0165 | 0.0097 | 0.088589 |
| rs6774894 | Age at first birth | A | T | NA | -0.0061 | 0.0096 | 0.5265 |
| rs6798742 | Age at first birth | G | A | NA | 0.0124 | 0.0099 | 0.2112 |
| rs6803651 | Age at first birth | T | G | NA | 0.0268 | 0.0094 | 0.004483 |
| rs6805241 | Age at first birth | C | T | NA | -0.031 | 0.0109 | 0.004431 |
| rs6843852 | Age at first birth | T | C | NA | -1.00E-04 | 0.0092 | 0.9936 |
| rs6943762 | Age at first birth | C | T | NA | 0.0156 | 0.0136 | 0.2486 |
| rs6959891 | Age at first birth | G | A | NA | -0.0144 | 0.01 | 0.1507 |
| rs6962980 | Age at first birth | C | A | NA | 0.008 | 0.0096 | 0.4034 |
| rs6974218 | Age at first birth | C | A | NA | -0.0122 | 0.0095 | 0.196 |
| rs698147 | Age at first birth | G | A | NA | 0.0068 | 0.0092 | 0.4572 |
| rs7008955 | Age at first birth | G | T | NA | 0.0153 | 0.0092 | 0.09868 |
| rs7012546 | Age at first birth | T | C | NA | 0.0176 | 0.0094 | 0.06042 |
| rs7029718 | Age at first birth | A | G | NA | 0.0575 | 0.0095 | 1.38E-09 |
| rs7031698 | Age at first birth | C | T | NA | 0.0325 | 0.0109 | 0.00289 |
| rs7034554 | Age at first birth | G | A | NA | -0.002 | 0.0095 | 0.8325 |
| rs7038943 | Age at first birth | C | T | NA | 0.0247 | 0.0094 | 0.008959 |
| rs7079070 | Age at first birth | A | G | NA | -0.0214 | 0.0094 | 0.02223 |
| rs708228 | Age at first birth | T | C | NA | -0.0061 | 0.0096 | 0.527 |
| rs7124681 | Age at first birth | A | C | NA | -0.0029 | 0.0093 | 0.7567 |
| rs7132908 | Age at first birth | A | G | NA | 0.0168 | 0.0096 | 0.0808 |
| rs71646142 | Age at first birth | T | C | NA | 0.0172 | 0.0119 | 0.147 |
| rs7201895 | Age at first birth | A | G | NA | 0.0091 | 0.01 | 0.3614 |
| rs7206608 | Age at first birth | G | C | NA | -0.0143 | 0.0097 | 0.1375 |
| rs7218014 | Age at first birth | C | T | NA | -0.0288 | 0.0117 | 0.01407 |
| rs7233920 | Age at first birth | A | G | NA | -0.016 | 0.0108 | 0.1411 |
| rs7236339 | Age at first birth | A | G | NA | -0.0531 | 0.0119 | 8.22E-06 |
| rs723672 | Age at first birth | T | C | NA | -0.0125 | 0.0094 | 0.1823 |
| rs7250833 | Age at first birth | T | C | NA | -0.0087 | 0.01 | 0.3821 |
| rs7259070 | Age at first birth | C | T | NA | -0.0156 | 0.0102 | 0.1257 |
| rs72673947 | Age at first birth | G | A | NA | 0.0013 | 0.0149 | 0.9307 |
| rs7278859 | Age at first birth | T | A | NA | 0.0251 | 0.0101 | 0.01274 |
| rs72887338 | Age at first birth | C | T | NA | -0.0334 | 0.0095 | 0.000422 |
| rs72892910 | Age at first birth | T | G | NA | 0.0203 | 0.0121 | 0.09384 |
| rs72910629 | Age at first birth | G | A | NA | -0.0129 | 0.0136 | 0.3421 |
| rs72986630 | Age at first birth | T | C | NA | 0.0217 | 0.022 | 0.3234 |
| rs73026725 | Age at first birth | A | C | NA | 0.0035 | 0.0129 | 0.7843 |
| rs730384 | Age at first birth | A | G | NA | 0.0278 | 0.0091 | 0.002332 |
| rs73052033 | Age at first birth | C | T | NA | -0.0103 | 0.012 | 0.3913 |
| rs73142879 | Age at first birth | T | C | NA | 0.0102 | 0.0118 | 0.388 |
| rs73213484 | Age at first birth | T | A | NA | 0.0125 | 0.0132 | 0.3438 |
| rs7331420 | Age at first birth | A | G | NA | 0.0187 | 0.0102 | 0.06703 |
| rs7332724 | Age at first birth | T | C | NA | -0.0065 | 0.01 | 0.517201 |
| rs73344830 | Age at first birth | G | A | NA | -0.0358 | 0.0097 | 0.00022 |
| rs7357754 | Age at first birth | G | A | NA | -0.0366 | 0.0093 | 8.35E-05 |
| rs7359501 | Age at first birth | T | C | NA | 0.0565 | 0.0094 | 1.82E-09 |
| rs736282 | Age at first birth | C | T | NA | -0.0041 | 0.0093 | 0.657099 |
| rs7442137 | Age at first birth | T | C | NA | -0.0011 | 0.0099 | 0.9088 |
| rs7442885 | Age at first birth | G | C | NA | 0.0472 | 0.011 | 1.85E-05 |
| rs745249 | Age at first birth | T | C | NA | -0.0167 | 0.0102 | 0.1033 |
| rs7498044 | Age at first birth | A | G | NA | 0.027 | 0.0115 | 0.0195 |
| rs7498665 | Age at first birth | G | A | NA | 0.0069 | 0.0094 | 0.462 |
| rs75035127 | Age at first birth | G | A | NA | 0.0188 | 0.0283 | 0.507 |
| rs7551758 | Age at first birth | G | T | NA | -0.0103 | 0.009 | 0.2531 |
| rs7575189 | Age at first birth | A | G | NA | 0.0427 | 0.0091 | 2.98E-06 |
| rs7594904 | Age at first birth | C | T | NA | 0.0139 | 0.0092 | 0.1278 |
| rs76076331 | Age at first birth | T | C | NA | 0.0475 | 0.0135 | 0.000421 |
| rs76702514 | Age at first birth | G | C | NA | -0.0156 | 0.0117 | 0.1822 |
| rs76878669 | Age at first birth | G | C | NA | -0.0149 | 0.0114 | 0.1937 |
| rs76954012 | Age at first birth | A | T | NA | -0.0283 | 0.0161 | 0.078381 |
| rs7704530 | Age at first birth | A | G | NA | -0.0486 | 0.0102 | 1.79E-06 |
| rs7762794 | Age at first birth | G | A | NA | -0.0383 | 0.0102 | 0.000158 |
| rs77702622 | Age at first birth | A | G | NA | -0.0166 | 0.0197 | 0.4004 |
| rs7774 | Age at first birth | A | C | NA | -0.0405 | 0.0104 | 9.26E-05 |
| rs77835879 | Age at first birth | G | A | NA | -0.0159 | 0.0157 | 0.3105 |
| rs778371 | Age at first birth | G | A | NA | 0.0233 | 0.0105 | 0.02602 |
| rs7785195 | Age at first birth | A | G | NA | 0.0454 | 0.0096 | 2.48E-06 |
| rs7803932 | Age at first birth | A | G | NA | 0.041 | 0.0121 | 0.000681 |
| rs78086698 | Age at first birth | C | T | NA | 0.0397 | 0.0247 | 0.1084 |
| rs7852189 | Age at first birth | G | A | NA | -0.0035 | 0.0099 | 0.724499 |
| rs7893571 | Age at first birth | T | G | NA | -0.0162 | 0.0097 | 0.09498 |
| rs7909331 | Age at first birth | G | A | NA | -0.0294 | 0.0122 | 0.01573 |
| rs7920624 | Age at first birth | T | A | NA | -0.0154 | 0.009 | 0.087231 |
| rs7921378 | Age at first birth | C | G | NA | 0.003 | 0.0091 | 0.743599 |
| rs7924036 | Age at first birth | T | G | NA | 0.0425 | 0.0092 | 4.04E-06 |
| rs79265434 | Age at first birth | G | A | NA | 0.0353 | 0.0141 | 0.01221 |
| rs79269403 | Age at first birth | A | G | NA | 0.0328 | 0.0111 | 0.003262 |
| rs7927195 | Age at first birth | G | A | NA | -0.0169 | 0.0093 | 0.067909 |
| rs7928622 | Age at first birth | T | A | NA | 0.0132 | 0.0097 | 0.1745 |
| rs7938812 | Age at first birth | G | T | NA | -0.0037 | 0.0093 | 0.692599 |
| rs79445414 | Age at first birth | C | T | NA | -0.0186 | 0.0219 | 0.3965 |
| rs7944782 | Age at first birth | G | T | NA | 0.0056 | 0.0091 | 0.5386 |
| rs7952102 | Age at first birth | C | T | NA | 0.0175 | 0.0098 | 0.07415 |
| rs7996639 | Age at first birth | A | G | NA | -0.0089 | 0.0093 | 0.3406 |
| rs80153284 | Age at first birth | A | C | NA | 0.2314 | 0.0411 | 1.74E-08 |
| rs8015400 | Age at first birth | A | C | NA | -0.0116 | 0.0099 | 0.2413 |
| rs8020034 | Age at first birth | A | G | NA | 0.0458 | 0.0118 | 0.000107 |
| rs8112818 | Age at first birth | G | A | NA | 0.0144 | 0.01 | 0.1493 |
| rs8132491 | Age at first birth | A | G | NA | 0.0025 | 0.0107 | 0.8156 |
| rs815163 | Age at first birth | C | T | NA | 0.0031 | 0.0091 | 0.7366 |
| rs862320 | Age at first birth | T | C | NA | 0.0125 | 0.0093 | 0.1789 |
| rs879620 | Age at first birth | T | C | NA | -0.009 | 0.0096 | 0.3445 |
| rs892612 | Age at first birth | C | A | NA | 0.0107 | 0.0126 | 0.3973 |
| rs9294260 | Age at first birth | A | G | NA | -0.0022 | 0.0091 | 0.8113 |
| rs9296389 | Age at first birth | C | G | NA | 0.0095 | 0.0092 | 0.3019 |
| rs9320493 | Age at first birth | G | A | NA | -0.0209 | 0.0129 | 0.1043 |
| rs9349956 | Age at first birth | C | A | NA | 0.0526 | 0.0122 | 1.54E-05 |
| rs935166 | Age at first birth | A | G | NA | 0.0388 | 0.0092 | 2.67E-05 |
| rs9366863 | Age at first birth | C | T | NA | 0.0204 | 0.0098 | 0.03727 |
| rs9372625 | Age at first birth | A | G | NA | 0.0667 | 0.0096 | 3.32E-12 |
| rs9386319 | Age at first birth | G | A | NA | 0.0249 | 0.0092 | 0.007067 |
| rs9478496 | Age at first birth | C | T | NA | -0.0077 | 0.0122 | 0.5287 |
| rs9503598 | Age at first birth | A | G | NA | 0.0137 | 0.0094 | 0.1455 |
| rs9514600 | Age at first birth | G | C | NA | -0.0202 | 0.009 | 0.02563 |
| rs9522173 | Age at first birth | T | A | NA | 0.0133 | 0.0093 | 0.1509 |
| rs9529119 | Age at first birth | G | C | NA | -0.0322 | 0.0111 | 0.003739 |
| rs9571687 | Age at first birth | A | C | NA | 0.0036 | 0.0097 | 0.708 |
| rs9616906 | Age at first birth | A | G | NA | 0.0178 | 0.0094 | 0.058519 |
| rs9636107 | Age at first birth | G | A | NA | -0.0431 | 0.009 | 1.73E-06 |
| rs9643087 | Age at first birth | T | C | NA | -0.0173 | 0.0093 | 0.061511 |
| rs969512 | Age at first birth | T | A | NA | 0.0129 | 0.0097 | 0.1822 |
| rs9704097 | Age at first birth | A | C | NA | -0.0287 | 0.0093 | 0.001953 |
| rs9852062 | Age at first birth | A | T | NA | 0.0411 | 0.0095 | 1.55E-05 |
| rs9860326 | Age at first birth | G | C | NA | -0.0241 | 0.0096 | 0.01208 |
| rs9882532 | Age at first birth | C | T | NA | -0.0141 | 0.0094 | 0.1328 |
| rs9888533 | Age at first birth | T | C | NA | 0.0082 | 0.01 | 0.4109 |
| rs9951619 | Age at first birth | G | T | NA | 0.0101 | 0.0107 | 0.3453 |
| rs9964724 | Age at first birth | T | C | NA | 0.0475 | 0.0097 | 1.08E-06 |
| rs1000237 | Age at first sexual intercourse | A | T | NA | -0.0047 | 0.0022 | 0.023 |
| rs10073890 | Age at first sexual intercourse | G | A | NA | -0.0141 | 0.0024 | 2.80E-09 |
| rs10160769 | Age at first sexual intercourse | C | G | NA | 0.0068 | 0.0025 | 0.0086 |
| rs1017529 | Age at first sexual intercourse | A | C | NA | -0.0049 | 0.0028 | 0.072 |
| rs10423928 | Age at first sexual intercourse | A | T | NA | 0.0043 | 0.0026 | 0.1 |
| rs10505836 | Age at first sexual intercourse | C | A | NA | -0.0133 | 0.003 | 7.70E-06 |
| rs10510025 | Age at first sexual intercourse | T | C | NA | -0.0163 | 0.0024 | 8.90E-12 |
| rs1064213 | Age at first sexual intercourse | A | G | NA | -0.0077 | 0.0021 | 0.00012 |
| rs10742752 | Age at first sexual intercourse | C | T | NA | -9.00E-04 | 0.0021 | 0.73 |
| rs10752613 | Age at first sexual intercourse | A | T | NA | -0.012 | 0.0023 | 1.40E-07 |
| rs10765775 | Age at first sexual intercourse | A | G | NA | 0.0093 | 0.0021 | 1.10E-05 |
| rs10773002 | Age at first sexual intercourse | T | A | NA | -0.0067 | 0.0024 | 0.006 |
| rs10797055 | Age at first sexual intercourse | G | A | NA | 0.0042 | 0.0021 | 0.031 |
| rs10858054 | Age at first sexual intercourse | T | G | NA | 0.0149 | 0.0027 | 2.30E-08 |
| rs10861176 | Age at first sexual intercourse | A | G | NA | 0.0034 | 0.0023 | 0.18 |
| rs10887578 | Age at first sexual intercourse | C | G | NA | 0.0026 | 0.0021 | 0.25 |
| rs10887801 | Age at first sexual intercourse | T | G | NA | 0.0054 | 0.0021 | 0.011 |
| rs10922907 | Age at first sexual intercourse | T | A | NA | 0.0211 | 0.0021 | 2.10E-24 |
| rs10938398 | Age at first sexual intercourse | A | G | NA | -0.0014 | 0.0021 | 0.44 |
| rs10963297 | Age at first sexual intercourse | G | C | NA | 0.0116 | 0.0024 | 3.20E-06 |
| rs11000993 | Age at first sexual intercourse | C | T | NA | -0.0021 | 0.0031 | 0.47 |
| rs11012732 | Age at first sexual intercourse | G | A | NA | -0.0043 | 0.0022 | 0.049 |
| rs1105307 | Age at first sexual intercourse | A | G | NA | -0.0087 | 0.0024 | 0.00029 |
| rs11079849 | Age at first sexual intercourse | T | C | NA | -7.00E-04 | 0.0022 | 0.719999 |
| rs11081529 | Age at first sexual intercourse | C | T | NA | -3.00E-04 | 0.0023 | 0.99 |
| rs11099020 | Age at first sexual intercourse | T | C | NA | 5.00E-04 | 0.0022 | 0.82 |
| rs11134679 | Age at first sexual intercourse | G | A | NA | -0.0064 | 0.0022 | 0.0047 |
| rs11165643 | Age at first sexual intercourse | T | C | NA | -0.0022 | 0.0021 | 0.29 |
| rs11250094 | Age at first sexual intercourse | C | G | NA | 0.0153 | 0.0021 | 1.60E-13 |
| rs112633616 | Age at first sexual intercourse | C | A | NA | 0.0386 | 0.0058 | 5.20E-11 |
| rs112687095 | Age at first sexual intercourse | A | G | NA | 0.006 | 0.0028 | 0.042 |
| rs113338260 | Age at first sexual intercourse | C | T | NA | -0.0149 | 0.0025 | 2.20E-09 |
| rs113520408 | Age at first sexual intercourse | A | G | NA | 0.0088 | 0.0023 | 0.00019 |
| rs113624107 | Age at first sexual intercourse | A | G | NA | -0.0047 | 0.0025 | 0.075 |
| rs1143770 | Age at first sexual intercourse | T | C | NA | 0.0035 | 0.0021 | 0.1 |
| rs115000530 | Age at first sexual intercourse | T | A | NA | 0.0074 | 0.0046 | 0.11 |
| rs115454970 | Age at first sexual intercourse | T | G | NA | -6.00E-04 | 0.0024 | 0.709999 |
| rs11587347 | Age at first sexual intercourse | G | C | NA | -0.0061 | 0.0035 | 0.083 |
| rs11620355 | Age at first sexual intercourse | A | G | NA | 0.0063 | 0.0037 | 0.069 |
| rs11635092 | Age at first sexual intercourse | A | G | NA | -0.0042 | 0.0022 | 0.046 |
| rs116377258 | Age at first sexual intercourse | G | A | NA | 0.0034 | 0.0065 | 0.64 |
| rs11693094 | Age at first sexual intercourse | T | C | NA | -0.0069 | 0.0021 | 0.00081 |
| rs11696755 | Age at first sexual intercourse | C | T | NA | 9.00E-04 | 0.0027 | 0.780001 |
| rs11709402 | Age at first sexual intercourse | G | A | NA | -0.0064 | 0.0023 | 0.0054 |
| rs117118217 | Age at first sexual intercourse | C | G | NA | -8.00E-04 | 0.0082 | 0.98 |
| rs11732657 | Age at first sexual intercourse | A | G | NA | -0.0055 | 0.0024 | 0.024 |
| rs118136827 | Age at first sexual intercourse | T | G | NA | -0.0035 | 0.0023 | 0.14 |
| rs11866420 | Age at first sexual intercourse | G | C | NA | -0.0168 | 0.0021 | 7.60E-16 |
| rs1198588 | Age at first sexual intercourse | T | A | NA | 0.0082 | 0.0026 | 0.002 |
| rs12001437 | Age at first sexual intercourse | C | T | NA | 4.00E-04 | 0.0021 | 0.91 |
| rs12033257 | Age at first sexual intercourse | G | A | NA | 0.0058 | 0.0021 | 0.0048 |
| rs12140153 | Age at first sexual intercourse | T | G | NA | 0.0099 | 0.0036 | 0.0079 |
| rs12149660 | Age at first sexual intercourse | A | G | NA | 0.0102 | 0.0032 | 0.0013 |
| rs12156160 | Age at first sexual intercourse | G | A | NA | 8.00E-04 | 0.0029 | 0.85 |
| rs12204714 | Age at first sexual intercourse | T | C | NA | 0.0275 | 0.0021 | 8.30E-38 |
| rs12293670 | Age at first sexual intercourse | G | A | NA | 0.0026 | 0.0022 | 0.33 |
| rs12303743 | Age at first sexual intercourse | C | G | NA | 0.002 | 0.0035 | 0.54 |
| rs12364470 | Age at first sexual intercourse | G | T | NA | 0.0031 | 0.0028 | 0.23 |
| rs12375949 | Age at first sexual intercourse | C | T | NA | 0.0096 | 0.0021 | 5.40E-06 |
| rs12375985 | Age at first sexual intercourse | A | G | NA | 0.0019 | 0.0022 | 0.33 |
| rs12427047 | Age at first sexual intercourse | T | C | NA | 0.0039 | 0.0024 | 0.092001 |
| rs12519073 | Age at first sexual intercourse | T | C | NA | -0.0068 | 0.0025 | 0.0092 |
| rs12523398 | Age at first sexual intercourse | A | T | NA | 0.0219 | 0.0027 | 1.10E-15 |
| rs12643771 | Age at first sexual intercourse | T | C | NA | 0.0084 | 0.0022 | 8.10E-05 |
| rs1266874 | Age at first sexual intercourse | G | A | NA | 8.00E-04 | 0.0022 | 0.709999 |
| rs12681792 | Age at first sexual intercourse | A | C | NA | -1.00E-04 | 0.0026 | 0.94 |
| rs12682775 | Age at first sexual intercourse | C | T | NA | 0.0057 | 0.0025 | 0.024 |
| rs12712510 | Age at first sexual intercourse | C | T | NA | 0.0088 | 0.0021 | 1.40E-05 |
| rs12714592 | Age at first sexual intercourse | C | A | NA | -0.0217 | 0.0023 | 1.10E-20 |
| rs12714702 | Age at first sexual intercourse | G | A | NA | -0.0242 | 0.0028 | 1.40E-17 |
| rs12757779 | Age at first sexual intercourse | A | G | NA | 0.0134 | 0.0025 | 3.70E-08 |
| rs12762034 | Age at first sexual intercourse | C | T | NA | -0.0017 | 0.0039 | 0.67 |
| rs1286058 | Age at first sexual intercourse | A | T | NA | -7.00E-04 | 0.0023 | 0.79 |
| rs12881629 | Age at first sexual intercourse | G | A | NA | -0.0034 | 0.0038 | 0.3 |
| rs12907546 | Age at first sexual intercourse | A | G | NA | -0.0227 | 0.0025 | 2.10E-19 |
| rs12919291 | Age at first sexual intercourse | C | G | NA | -0.0073 | 0.0027 | 0.0046 |
| rs12940014 | Age at first sexual intercourse | C | T | NA | 0.0039 | 0.0021 | 0.056999 |
| rs12955211 | Age at first sexual intercourse | A | T | NA | 0.005 | 0.0022 | 0.03 |
| rs12956148 | Age at first sexual intercourse | A | C | NA | 0.0051 | 0.0023 | 0.035 |
| rs1296328 | Age at first sexual intercourse | C | A | NA | 0.0068 | 0.0021 | 0.0013 |
| rs12977787 | Age at first sexual intercourse | A | G | NA | -0.0015 | 0.0021 | 0.44 |
| rs13030994 | Age at first sexual intercourse | A | G | NA | -3.00E-04 | 0.0021 | 0.95 |
| rs13037326 | Age at first sexual intercourse | T | C | NA | -0.0083 | 0.0024 | 0.00029 |
| rs13090388 | Age at first sexual intercourse | T | C | NA | 0.0178 | 0.0022 | 1.20E-15 |
| rs13107325 | Age at first sexual intercourse | T | C | NA | 0.003 | 0.004 | 0.51 |
| rs13141210 | Age at first sexual intercourse | T | C | NA | 0.0098 | 0.0021 | 1.60E-06 |
| rs13145650 | Age at first sexual intercourse | T | C | NA | -0.0074 | 0.0037 | 0.047 |
| rs13175535 | Age at first sexual intercourse | A | G | NA | 0.0125 | 0.0023 | 4.40E-08 |
| rs13195636 | Age at first sexual intercourse | C | A | NA | 0.0087 | 0.0032 | 0.0081 |
| rs1320251 | Age at first sexual intercourse | T | C | NA | 0.0093 | 0.0021 | 1.20E-05 |
| rs13218383 | Age at first sexual intercourse | G | C | NA | 0.0036 | 0.0022 | 0.083 |
| rs13233308 | Age at first sexual intercourse | T | C | NA | -0.0014 | 0.0021 | 0.57 |
| rs13248187 | Age at first sexual intercourse | C | T | NA | 0.0048 | 0.0023 | 0.033 |
| rs13261666 | Age at first sexual intercourse | T | G | NA | 0.0076 | 0.0021 | 0.0003 |
| rs1327259 | Age at first sexual intercourse | G | A | NA | 3.00E-04 | 0.0021 | 0.79 |
| rs13292699 | Age at first sexual intercourse | C | A | NA | 0.0019 | 0.0021 | 0.35 |
| rs1330199 | Age at first sexual intercourse | T | G | NA | 0.0032 | 0.0021 | 0.13 |
| rs13307225 | Age at first sexual intercourse | A | G | NA | 0.0218 | 0.0034 | 4.70E-11 |
| rs13317303 | Age at first sexual intercourse | A | C | NA | -0.0035 | 0.0029 | 0.24 |
| rs1334297 | Age at first sexual intercourse | A | G | NA | 0.0083 | 0.0023 | 0.00043 |
| rs13422673 | Age at first sexual intercourse | T | C | NA | 0.0016 | 0.0021 | 0.47 |
| rs13427822 | Age at first sexual intercourse | G | A | NA | 0.0011 | 0.0023 | 0.7 |
| rs1346841 | Age at first sexual intercourse | A | G | NA | 7.00E-04 | 0.0021 | 0.719999 |
| rs1360201 | Age at first sexual intercourse | T | C | NA | -0.0073 | 0.0021 | 0.00059 |
| rs1363862 | Age at first sexual intercourse | A | G | NA | -0.0063 | 0.0023 | 0.0059 |
| rs13642 | Age at first sexual intercourse | T | A | NA | -2.00E-04 | 0.0021 | 0.93 |
| rs1391438 | Age at first sexual intercourse | C | T | NA | 0.0032 | 0.0022 | 0.15 |
| rs1392816 | Age at first sexual intercourse | T | C | NA | 0.017 | 0.0021 | 6.20E-16 |
| rs140159717 | Age at first sexual intercourse | T | C | NA | 0.0109 | 0.0039 | 0.0049 |
| rs1438945 | Age at first sexual intercourse | A | T | NA | -0.0022 | 0.0023 | 0.3 |
| rs1441264 | Age at first sexual intercourse | A | G | NA | 0.0036 | 0.0021 | 0.089 |
| rs1450782 | Age at first sexual intercourse | G | T | NA | -0.006 | 0.0021 | 0.0046 |
| rs1455350 | Age at first sexual intercourse | A | T | NA | -0.0047 | 0.0021 | 0.017 |
| rs1458156 | Age at first sexual intercourse | T | C | NA | 0.0101 | 0.0021 | 9.50E-07 |
| rs1471740 | Age at first sexual intercourse | C | T | NA | -0.009 | 0.0024 | 0.00016 |
| rs147568678 | Age at first sexual intercourse | C | T | NA | 7.00E-04 | 0.0024 | 0.8 |
| rs1477290 | Age at first sexual intercourse | C | T | NA | 0.0098 | 0.003 | 0.0011 |
| rs1503526 | Age at first sexual intercourse | C | T | NA | -7.00E-04 | 0.0021 | 0.649999 |
| rs152603 | Age at first sexual intercourse | G | A | NA | 0.0052 | 0.0021 | 0.019 |
| rs1565735 | Age at first sexual intercourse | A | T | NA | -0.0061 | 0.0026 | 0.016 |
| rs1566085 | Age at first sexual intercourse | T | G | NA | 0.0044 | 0.0021 | 0.031 |
| rs1582931 | Age at first sexual intercourse | A | G | NA | -0.0052 | 0.0021 | 0.014 |
| rs1584469 | Age at first sexual intercourse | T | C | NA | -0.008 | 0.0023 | 0.00033 |
| rs1609010 | Age at first sexual intercourse | G | A | NA | -0.001 | 0.0021 | 0.780001 |
| rs1671770 | Age at first sexual intercourse | C | A | NA | -0.0011 | 0.0028 | 0.68 |
| rs16846140 | Age at first sexual intercourse | G | A | NA | -0.0056 | 0.0022 | 0.0093 |
| rs16846463 | Age at first sexual intercourse | G | A | NA | -0.0161 | 0.0034 | 3.50E-06 |
| rs16854920 | Age at first sexual intercourse | C | T | NA | 0.0109 | 0.0022 | 2.20E-07 |
| rs1689510 | Age at first sexual intercourse | C | G | NA | 0.0123 | 0.0022 | 2.60E-08 |
| rs16916303 | Age at first sexual intercourse | G | A | NA | 0.0037 | 0.0032 | 0.29 |
| rs16995054 | Age at first sexual intercourse | T | C | NA | -0.0101 | 0.0026 | 1.00E-04 |
| rs17193211 | Age at first sexual intercourse | T | C | NA | -3.00E-04 | 0.0042 | 0.88 |
| rs17194490 | Age at first sexual intercourse | T | G | NA | 0.0031 | 0.0028 | 0.24 |
| rs17399739 | Age at first sexual intercourse | G | A | NA | -0.0013 | 0.0041 | 0.780001 |
| rs17565975 | Age at first sexual intercourse | A | G | NA | -0.0052 | 0.0021 | 0.017 |
| rs17598675 | Age at first sexual intercourse | C | T | NA | 0.0088 | 0.0021 | 2.30E-05 |
| rs176218 | Age at first sexual intercourse | T | G | NA | 0.0099 | 0.0026 | 0.00016 |
| rs1778830 | Age at first sexual intercourse | A | G | NA | -0.0088 | 0.0021 | 4.20E-05 |
| rs1788808 | Age at first sexual intercourse | G | A | NA | 0.0085 | 0.0021 | 5.90E-05 |
| rs1834144 | Age at first sexual intercourse | A | C | NA | 5.00E-04 | 0.0021 | 0.83 |
| rs1860002 | Age at first sexual intercourse | T | C | NA | 4.00E-04 | 0.0021 | 0.84 |
| rs1899896 | Age at first sexual intercourse | T | C | NA | -0.0101 | 0.0023 | 7.70E-06 |
| rs1901512 | Age at first sexual intercourse | C | T | NA | -0.0024 | 0.0023 | 0.23 |
| rs1915019 | Age at first sexual intercourse | G | A | NA | -2.00E-04 | 0.0024 | 0.88 |
| rs1919243 | Age at first sexual intercourse | C | T | NA | -2.00E-04 | 0.0021 | 1 |
| rs1950829 | Age at first sexual intercourse | G | A | NA | 2.00E-04 | 0.0021 | 0.92 |
| rs1967772 | Age at first sexual intercourse | A | G | NA | -0.0033 | 0.0023 | 0.19 |
| rs2035936 | Age at first sexual intercourse | T | G | NA | 0.0057 | 0.0046 | 0.2 |
| rs2076603 | Age at first sexual intercourse | A | G | NA | 0.0022 | 0.0022 | 0.26 |
| rs2084572 | Age at first sexual intercourse | G | A | NA | 0.014 | 0.0021 | 3.50E-11 |
| rs2102278 | Age at first sexual intercourse | G | A | NA | -7.00E-04 | 0.0022 | 0.75 |
| rs2133561 | Age at first sexual intercourse | T | A | NA | 1.00E-04 | 0.0021 | 0.95 |
| rs213518 | Age at first sexual intercourse | C | T | NA | 0.0075 | 0.0029 | 0.007 |
| rs2153740 | Age at first sexual intercourse | G | A | NA | 0.001 | 0.0021 | 0.6 |
| rs215634 | Age at first sexual intercourse | G | A | NA | 0.0103 | 0.0021 | 1.40E-06 |
| rs2172131 | Age at first sexual intercourse | C | T | NA | 0.0085 | 0.0021 | 5.50E-05 |
| rs217336 | Age at first sexual intercourse | A | C | NA | -0.0023 | 0.0021 | 0.25 |
| rs2174752 | Age at first sexual intercourse | T | G | NA | -0.0128 | 0.0021 | 5.90E-10 |
| rs2176337 | Age at first sexual intercourse | T | A | NA | -0.0151 | 0.0022 | 1.10E-11 |
| rs217672 | Age at first sexual intercourse | C | A | NA | -0.0033 | 0.0023 | 0.16 |
| rs2214123 | Age at first sexual intercourse | G | A | NA | 0.0055 | 0.0022 | 0.012 |
| rs2234458 | Age at first sexual intercourse | T | C | NA | 0.0031 | 0.0021 | 0.18 |
| rs2253310 | Age at first sexual intercourse | G | C | NA | -0.0054 | 0.0021 | 0.012 |
| rs2281819 | Age at first sexual intercourse | A | T | NA | 0.0036 | 0.0025 | 0.14 |
| rs2283076 | Age at first sexual intercourse | G | A | NA | -0.0057 | 0.0025 | 0.017 |
| rs2289379 | Age at first sexual intercourse | T | C | NA | 0.0033 | 0.0021 | 0.14 |
| rs2302761 | Age at first sexual intercourse | T | C | NA | -8.00E-04 | 0.0025 | 0.86 |
| rs2306593 | Age at first sexual intercourse | T | C | NA | 0.0028 | 0.0021 | 0.16 |
| rs2307111 | Age at first sexual intercourse | C | T | NA | 0.0023 | 0.0021 | 0.32 |
| rs2332700 | Age at first sexual intercourse | G | C | NA | -0.0019 | 0.0024 | 0.49 |
| rs2333321 | Age at first sexual intercourse | G | A | NA | 0.0023 | 0.0025 | 0.31 |
| rs2342892 | Age at first sexual intercourse | G | T | NA | 0.0074 | 0.0021 | 0.00034 |
| rs2347526 | Age at first sexual intercourse | C | T | NA | 0.0067 | 0.0022 | 0.0015 |
| rs2396625 | Age at first sexual intercourse | A | T | NA | 0.0042 | 0.0021 | 0.041 |
| rs2398861 | Age at first sexual intercourse | G | A | NA | -0.01 | 0.0024 | 2.20E-05 |
| rs240963 | Age at first sexual intercourse | C | T | NA | 0.0125 | 0.0028 | 6.30E-06 |
| rs242093 | Age at first sexual intercourse | A | G | NA | -0.0065 | 0.0021 | 0.0023 |
| rs2433733 | Age at first sexual intercourse | A | G | NA | 0.0046 | 0.0022 | 0.033 |
| rs2456020 | Age at first sexual intercourse | T | C | NA | -0.0062 | 0.0025 | 0.016 |
| rs2482356 | Age at first sexual intercourse | C | T | NA | -0.0037 | 0.0021 | 0.056999 |
| rs252761 | Age at first sexual intercourse | T | G | NA | 0.0017 | 0.0021 | 0.44 |
| rs2554835 | Age at first sexual intercourse | A | G | NA | 0.0069 | 0.0021 | 8.00E-04 |
| rs2568958 | Age at first sexual intercourse | A | G | NA | -0.0065 | 0.0021 | 0.002 |
| rs2606228 | Age at first sexual intercourse | C | A | NA | 0.0012 | 0.0022 | 0.55 |
| rs2612030 | Age at first sexual intercourse | C | T | NA | 0.024 | 0.0028 | 1.20E-17 |
| rs2616143 | Age at first sexual intercourse | A | G | NA | 0.004 | 0.0022 | 0.068 |
| rs266047 | Age at first sexual intercourse | A | G | NA | 0.0161 | 0.0021 | 9.00E-15 |
| rs2678204 | Age at first sexual intercourse | G | T | NA | -0.0058 | 0.0022 | 0.0073 |
| rs2781668 | Age at first sexual intercourse | T | C | NA | 0.0061 | 0.0028 | 0.027 |
| rs2787101 | Age at first sexual intercourse | T | C | NA | 0.0058 | 0.0021 | 0.0043 |
| rs2819336 | Age at first sexual intercourse | C | T | NA | -0.0104 | 0.0021 | 1.30E-06 |
| rs28373063 | Age at first sexual intercourse | C | G | NA | 0.0032 | 0.0027 | 0.23 |
| rs28404639 | Age at first sexual intercourse | T | C | NA | -0.0057 | 0.0021 | 0.011 |
| rs28489620 | Age at first sexual intercourse | A | G | NA | -0.002 | 0.0023 | 0.36 |
| rs28513670 | Age at first sexual intercourse | G | A | NA | 0.0088 | 0.0027 | 0.002 |
| rs2875762 | Age at first sexual intercourse | C | G | NA | -0.0041 | 0.0024 | 0.11 |
| rs2876520 | Age at first sexual intercourse | G | C | NA | -0.0074 | 0.0021 | 4.00E-04 |
| rs2920503 | Age at first sexual intercourse | T | C | NA | 0.0081 | 0.0023 | 0.00036 |
| rs2923431 | Age at first sexual intercourse | C | G | NA | 0.0117 | 0.0021 | 7.50E-08 |
| rs293566 | Age at first sexual intercourse | C | T | NA | -0.011 | 0.0022 | 7.90E-07 |
| rs2962334 | Age at first sexual intercourse | T | G | NA | 0.0065 | 0.0074 | 0.44 |
| rs2971970 | Age at first sexual intercourse | G | T | NA | 0.0173 | 0.0025 | 3.80E-12 |
| rs301800 | Age at first sexual intercourse | C | T | NA | 6.00E-04 | 0.0027 | 0.86 |
| rs30266 | Age at first sexual intercourse | A | G | NA | -0.012 | 0.0022 | 4.90E-08 |
| rs317656 | Age at first sexual intercourse | A | T | NA | 0.0066 | 0.0023 | 0.0053 |
| rs320693 | Age at first sexual intercourse | C | G | NA | 0.004 | 0.0021 | 0.039 |
| rs3211995 | Age at first sexual intercourse | A | G | NA | 0.0046 | 0.0028 | 0.1 |
| rs329118 | Age at first sexual intercourse | T | C | NA | 0.0074 | 0.0021 | 0.00046 |
| rs34025316 | Age at first sexual intercourse | T | C | NA | 0.0036 | 0.0022 | 0.12 |
| rs34045288 | Age at first sexual intercourse | T | C | NA | 1.00E-04 | 0.0022 | 1 |
| rs34234296 | Age at first sexual intercourse | A | G | NA | 0.0017 | 0.0021 | 0.39 |
| rs34481751 | Age at first sexual intercourse | A | C | NA | 0.0029 | 0.0028 | 0.33 |
| rs34517439 | Age at first sexual intercourse | A | C | NA | -0.0195 | 0.0032 | 8.20E-10 |
| rs347551 | Age at first sexual intercourse | G | C | NA | 9.00E-04 | 0.0021 | 0.630001 |
| rs34811474 | Age at first sexual intercourse | A | G | NA | 0.0155 | 0.0024 | 1.30E-10 |
| rs35154326 | Age at first sexual intercourse | G | A | NA | 0.0106 | 0.0023 | 9.50E-06 |
| rs35309068 | Age at first sexual intercourse | G | T | NA | 0.0058 | 0.0021 | 0.0043 |
| rs354155 | Age at first sexual intercourse | C | G | NA | 8.00E-04 | 0.0036 | 0.84 |
| rs35417702 | Age at first sexual intercourse | T | C | NA | -0.0048 | 0.0021 | 0.018 |
| rs35475880 | Age at first sexual intercourse | T | G | NA | -0.009 | 0.0025 | 0.00083 |
| rs35532491 | Age at first sexual intercourse | T | A | NA | 0.0044 | 0.0034 | 0.19 |
| rs355777 | Age at first sexual intercourse | C | G | NA | 0.0012 | 0.0021 | 0.58 |
| rs35867081 | Age at first sexual intercourse | G | A | NA | 0.0095 | 0.0021 | 6.00E-06 |
| rs36007635 | Age at first sexual intercourse | A | G | NA | 0.0054 | 0.003 | 0.11 |
| rs36061954 | Age at first sexual intercourse | T | C | NA | -0.0115 | 0.0021 | 2.80E-08 |
| rs36119825 | Age at first sexual intercourse | A | G | NA | 0.0027 | 0.0021 | 0.22 |
| rs363096 | Age at first sexual intercourse | C | T | NA | -9.00E-04 | 0.0021 | 0.62 |
| rs3747631 | Age at first sexual intercourse | C | G | NA | 0.014 | 0.0025 | 3.50E-08 |
| rs3764625 | Age at first sexual intercourse | G | T | NA | 0.0021 | 0.0021 | 0.32 |
| rs3770754 | Age at first sexual intercourse | G | C | NA | -6.00E-04 | 0.0022 | 0.86 |
| rs3784710 | Age at first sexual intercourse | C | T | NA | 0.0041 | 0.0025 | 0.088 |
| rs3800546 | Age at first sexual intercourse | G | C | NA | -0.0048 | 0.0024 | 0.038 |
| rs3807865 | Age at first sexual intercourse | A | G | NA | -0.0018 | 0.0021 | 0.35 |
| rs3809634 | Age at first sexual intercourse | G | A | NA | 0.0055 | 0.0022 | 0.01 |
| rs3814883 | Age at first sexual intercourse | T | C | NA | -0.0052 | 0.0021 | 0.014 |
| rs3845344 | Age at first sexual intercourse | T | C | NA | -0.0133 | 0.0021 | 3.00E-10 |
| rs3851998 | Age at first sexual intercourse | G | C | NA | 0.0129 | 0.0024 | 1.40E-08 |
| rs3896224 | Age at first sexual intercourse | G | A | NA | 0.0198 | 0.0021 | 2.70E-21 |
| rs3897821 | Age at first sexual intercourse | G | A | NA | -0.0023 | 0.0022 | 0.29 |
| rs3901286 | Age at first sexual intercourse | A | C | NA | 0.0225 | 0.0029 | 6.10E-15 |
| rs394608 | Age at first sexual intercourse | C | T | NA | -0.0079 | 0.0021 | 1.00E-04 |
| rs40071 | Age at first sexual intercourse | C | T | NA | 0.0058 | 0.0027 | 0.038 |
| rs4044321 | Age at first sexual intercourse | G | A | NA | 0.0125 | 0.0022 | 5.60E-09 |
| rs4055791 | Age at first sexual intercourse | T | C | NA | 0.0046 | 0.0021 | 0.018 |
| rs406388 | Age at first sexual intercourse | G | C | NA | -0.0035 | 0.0027 | 0.19 |
| rs4148155 | Age at first sexual intercourse | G | A | NA | 0.0014 | 0.0032 | 0.760001 |
| rs4261944 | Age at first sexual intercourse | G | T | NA | -0.0051 | 0.0022 | 0.016 |
| rs4267103 | Age at first sexual intercourse | C | T | NA | -0.0085 | 0.0027 | 0.0015 |
| rs429343 | Age at first sexual intercourse | G | A | NA | 0.0018 | 0.0021 | 0.39 |
| rs429358 | Age at first sexual intercourse | C | T | NA | 0.0025 | 0.0029 | 0.42 |
| rs4328757 | Age at first sexual intercourse | T | C | NA | 0.0037 | 0.0021 | 0.08 |
| rs4352658 | Age at first sexual intercourse | T | C | NA | -0.0145 | 0.0038 | 7.90E-05 |
| rs4382592 | Age at first sexual intercourse | G | T | NA | 0.0132 | 0.0023 | 3.10E-09 |
| rs4419475 | Age at first sexual intercourse | T | A | NA | -0.0055 | 0.0021 | 0.012 |
| rs4439537 | Age at first sexual intercourse | C | T | NA | 0.0132 | 0.0021 | 1.10E-10 |
| rs4477562 | Age at first sexual intercourse | T | C | NA | 0.0063 | 0.0031 | 0.043 |
| rs4482463 | Age at first sexual intercourse | A | C | NA | -0.0019 | 0.0039 | 0.67 |
| rs4613074 | Age at first sexual intercourse | C | T | NA | 0.0053 | 0.0027 | 0.052 |
| rs4648450 | Age at first sexual intercourse | A | C | NA | 5.00E-04 | 0.0021 | 0.81 |
| rs4653164 | Age at first sexual intercourse | T | C | NA | -2.00E-04 | 0.0022 | 0.99 |
| rs4671328 | Age at first sexual intercourse | G | T | NA | 0.0024 | 0.0021 | 0.25 |
| rs4700393 | Age at first sexual intercourse | G | A | NA | 0.0113 | 0.0021 | 3.20E-08 |
| rs4702 | Age at first sexual intercourse | A | G | NA | 0.0172 | 0.0021 | 1.10E-16 |
| rs4709807 | Age at first sexual intercourse | C | T | NA | -0.0138 | 0.0024 | 1.00E-08 |
| rs4737188 | Age at first sexual intercourse | T | A | NA | -0.0071 | 0.0021 | 0.00065 |
| rs4757144 | Age at first sexual intercourse | A | G | NA | -3.00E-04 | 0.0021 | 0.88 |
| rs4757957 | Age at first sexual intercourse | C | G | NA | 0.0138 | 0.0022 | 1.10E-09 |
| rs4764949 | Age at first sexual intercourse | G | A | NA | 0.0041 | 0.0022 | 0.073 |
| rs4790292 | Age at first sexual intercourse | A | C | NA | 0.0069 | 0.0029 | 0.019 |
| rs4810227 | Age at first sexual intercourse | A | G | NA | 0.0066 | 0.0021 | 0.0012 |
| rs4812325 | Age at first sexual intercourse | A | G | NA | -0.002 | 0.0021 | 0.39 |
| rs4832298 | Age at first sexual intercourse | T | C | NA | -0.0047 | 0.0022 | 0.036 |
| rs4846724 | Age at first sexual intercourse | A | G | NA | 0.0056 | 0.0021 | 0.0069 |
| rs4876611 | Age at first sexual intercourse | G | A | NA | -0.0076 | 0.0023 | 0.00072 |
| rs4895799 | Age at first sexual intercourse | T | C | NA | -0.0027 | 0.0021 | 0.22 |
| rs4958702 | Age at first sexual intercourse | C | T | NA | -0.0022 | 0.0021 | 0.26 |
| rs4961705 | Age at first sexual intercourse | C | G | NA | 0.013 | 0.0022 | 4.20E-09 |
| rs512121 | Age at first sexual intercourse | C | T | NA | -0.0016 | 0.0026 | 0.55 |
| rs539515 | Age at first sexual intercourse | C | A | NA | -0.0073 | 0.0025 | 0.0051 |
| rs55658481 | Age at first sexual intercourse | A | G | NA | -3.00E-04 | 0.0022 | 0.82 |
| rs55707359 | Age at first sexual intercourse | G | T | NA | 9.00E-04 | 0.0085 | 0.93 |
| rs55714539 | Age at first sexual intercourse | C | A | NA | -0.008 | 0.0022 | 0.00016 |
| rs55726687 | Age at first sexual intercourse | A | G | NA | -0.0019 | 0.0025 | 0.48 |
| rs55736314 | Age at first sexual intercourse | G | C | NA | 0.0058 | 0.0021 | 0.007 |
| rs558887 | Age at first sexual intercourse | G | A | NA | 0.0056 | 0.0022 | 0.014 |
| rs56094641 | Age at first sexual intercourse | G | A | NA | 0.0049 | 0.0021 | 0.019 |
| rs56133507 | Age at first sexual intercourse | G | T | NA | -0.0061 | 0.0026 | 0.02 |
| rs56161855 | Age at first sexual intercourse | T | A | NA | -0.0049 | 0.003 | 0.092001 |
| rs56203622 | Age at first sexual intercourse | C | T | NA | -0.0118 | 0.0029 | 5.60E-05 |
| rs56335113 | Age at first sexual intercourse | G | A | NA | -0.0025 | 0.0022 | 0.3 |
| rs56356382 | Age at first sexual intercourse | C | T | NA | 0.0075 | 0.0026 | 0.0033 |
| rs56391344 | Age at first sexual intercourse | A | G | NA | 0.0088 | 0.0024 | 0.0003 |
| rs56399737 | Age at first sexual intercourse | T | C | NA | 0.0031 | 0.0021 | 0.1 |
| rs56858768 | Age at first sexual intercourse | A | G | NA | -0.0024 | 0.0023 | 0.29 |
| rs57636386 | Age at first sexual intercourse | C | T | NA | -6.00E-04 | 0.0038 | 0.84 |
| rs58120505 | Age at first sexual intercourse | C | T | NA | 0.0099 | 0.0021 | 9.90E-07 |
| rs59086897 | Age at first sexual intercourse | A | T | NA | 0.005 | 0.0021 | 0.014 |
| rs59237168 | Age at first sexual intercourse | C | T | NA | -0.001 | 0.0025 | 0.7 |
| rs5995843 | Age at first sexual intercourse | G | A | NA | -0.0031 | 0.0022 | 0.17 |
| rs60764613 | Age at first sexual intercourse | T | G | NA | -0.0142 | 0.003 | 1.90E-06 |
| rs6123924 | Age at first sexual intercourse | G | A | NA | -0.0086 | 0.0029 | 0.003 |
| rs6134916 | Age at first sexual intercourse | T | C | NA | 0.001 | 0.0021 | 0.6 |
| rs61813324 | Age at first sexual intercourse | T | C | NA | -0.0121 | 0.0031 | 7.10E-05 |
| rs61828088 | Age at first sexual intercourse | A | G | NA | 0.0116 | 0.0033 | 0.00046 |
| rs61903695 | Age at first sexual intercourse | G | A | NA | -0.0059 | 0.0024 | 0.013 |
| rs61914045 | Age at first sexual intercourse | A | G | NA | -0.0055 | 0.0026 | 0.036 |
| rs62097985 | Age at first sexual intercourse | T | C | NA | -0.0107 | 0.0021 | 3.90E-07 |
| rs62107261 | Age at first sexual intercourse | C | T | NA | 0.0073 | 0.0048 | 0.14 |
| rs62134195 | Age at first sexual intercourse | T | C | NA | 0.0318 | 0.0052 | 5.30E-10 |
| rs62176243 | Age at first sexual intercourse | T | A | NA | 0.01 | 0.0024 | 2.40E-05 |
| rs62176993 | Age at first sexual intercourse | A | G | NA | -0.0043 | 0.0021 | 0.038 |
| rs62190049 | Age at first sexual intercourse | C | G | NA | 0.0052 | 0.0021 | 0.014 |
| rs62439690 | Age at first sexual intercourse | A | G | NA | -0.0155 | 0.0024 | 7.20E-11 |
| rs6265 | Age at first sexual intercourse | T | C | NA | 0.0135 | 0.0026 | 4.90E-07 |
| rs6531639 | Age at first sexual intercourse | A | G | NA | 0.0088 | 0.0024 | 0.0003 |
| rs6545714 | Age at first sexual intercourse | A | G | NA | 0.0043 | 0.0021 | 0.045 |
| rs6546857 | Age at first sexual intercourse | G | A | NA | -0.0086 | 0.0024 | 0.00024 |
| rs6560906 | Age at first sexual intercourse | C | T | NA | 0.004 | 0.0022 | 0.077 |
| rs6567160 | Age at first sexual intercourse | C | T | NA | 0.0113 | 0.0024 | 3.00E-06 |
| rs6575340 | Age at first sexual intercourse | A | G | NA | -0.0129 | 0.0022 | 5.60E-09 |
| rs66511648 | Age at first sexual intercourse | C | T | NA | -0.0028 | 0.0023 | 0.19 |
| rs66679256 | Age at first sexual intercourse | T | C | NA | -0.006 | 0.0021 | 0.004 |
| rs6669341 | Age at first sexual intercourse | G | A | NA | 0.0012 | 0.0021 | 0.66 |
| rs6682438 | Age at first sexual intercourse | C | T | NA | -0.0084 | 0.0022 | 0.00013 |
| rs6688826 | Age at first sexual intercourse | C | T | NA | -0.0103 | 0.0022 | 5.30E-06 |
| rs6707827 | Age at first sexual intercourse | G | A | NA | -0.0025 | 0.0023 | 0.25 |
| rs6710091 | Age at first sexual intercourse | G | C | NA | -0.0011 | 0.0022 | 0.55 |
| rs6719762 | Age at first sexual intercourse | C | T | NA | -0.0221 | 0.0021 | 7.20E-27 |
| rs6731967 | Age at first sexual intercourse | C | G | NA | -0.002 | 0.0024 | 0.44 |
| rs6744646 | Age at first sexual intercourse | G | A | NA | -0.0171 | 0.0027 | 7.50E-10 |
| rs6744794 | Age at first sexual intercourse | G | C | NA | -0.0191 | 0.0021 | 1.30E-19 |
| rs6752979 | Age at first sexual intercourse | A | G | NA | -0.0051 | 0.0022 | 0.014 |
| rs6774894 | Age at first sexual intercourse | A | T | NA | -0.0018 | 0.0021 | 0.43 |
| rs6798742 | Age at first sexual intercourse | G | A | NA | 0.0044 | 0.0023 | 0.045 |
| rs6803651 | Age at first sexual intercourse | T | G | NA | 0.0012 | 0.0021 | 0.5 |
| rs6805241 | Age at first sexual intercourse | C | T | NA | -0.0093 | 0.0024 | 0.00018 |
| rs6843852 | Age at first sexual intercourse | T | C | NA | -2.00E-04 | 0.0021 | 0.82 |
| rs6943762 | Age at first sexual intercourse | C | T | NA | -0.0056 | 0.0031 | 0.072 |
| rs6959891 | Age at first sexual intercourse | G | A | NA | -0.0031 | 0.0023 | 0.14 |
| rs6962980 | Age at first sexual intercourse | C | A | NA | 0.0056 | 0.0021 | 0.0071 |
| rs6974218 | Age at first sexual intercourse | C | A | NA | -0.0022 | 0.0021 | 0.28 |
| rs698147 | Age at first sexual intercourse | G | A | NA | 0.0053 | 0.0021 | 0.0063 |
| rs7008955 | Age at first sexual intercourse | G | T | NA | 0.0116 | 0.0021 | 3.40E-08 |
| rs7012546 | Age at first sexual intercourse | T | C | NA | -0.0014 | 0.0021 | 0.52 |
| rs7029718 | Age at first sexual intercourse | A | G | NA | 0.0125 | 0.0021 | 1.80E-09 |
| rs7031698 | Age at first sexual intercourse | C | T | NA | 0.0067 | 0.0025 | 0.0061 |
| rs7034554 | Age at first sexual intercourse | G | A | NA | -0.0073 | 0.0021 | 4.00E-04 |
| rs7038943 | Age at first sexual intercourse | C | T | NA | 0.0021 | 0.0022 | 0.3 |
| rs7079070 | Age at first sexual intercourse | A | G | NA | -0.0165 | 0.0021 | 1.20E-15 |
| rs708228 | Age at first sexual intercourse | T | C | NA | -4.00E-04 | 0.0022 | 0.7 |
| rs7124681 | Age at first sexual intercourse | A | C | NA | 0.0034 | 0.0021 | 0.11 |
| rs7132908 | Age at first sexual intercourse | A | G | NA | 0.0034 | 0.0021 | 0.1 |
| rs71646142 | Age at first sexual intercourse | T | C | NA | 0.0038 | 0.0026 | 0.16 |
| rs7201895 | Age at first sexual intercourse | A | G | NA | 0.0032 | 0.0022 | 0.15 |
| rs7206608 | Age at first sexual intercourse | G | C | NA | -0.0038 | 0.0022 | 0.087 |
| rs7218014 | Age at first sexual intercourse | C | T | NA | -0.0128 | 0.0026 | 6.30E-07 |
| rs7233920 | Age at first sexual intercourse | A | G | NA | -0.0049 | 0.0025 | 0.04 |
| rs7236339 | Age at first sexual intercourse | A | G | NA | -0.0201 | 0.0025 | 3.90E-16 |
| rs723672 | Age at first sexual intercourse | T | C | NA | -7.00E-04 | 0.0021 | 0.86 |
| rs7250833 | Age at first sexual intercourse | T | C | NA | 4.00E-04 | 0.0023 | 0.85 |
| rs7259070 | Age at first sexual intercourse | C | T | NA | -0.0054 | 0.0021 | 0.017 |
| rs72673947 | Age at first sexual intercourse | G | A | NA | 3.00E-04 | 0.0034 | 0.91 |
| rs7278859 | Age at first sexual intercourse | T | A | NA | 0.0077 | 0.0023 | 0.00055 |
| rs72887338 | Age at first sexual intercourse | C | T | NA | -0.0163 | 0.0021 | 1.60E-14 |
| rs72892910 | Age at first sexual intercourse | T | G | NA | -0.0035 | 0.0027 | 0.23 |
| rs72910629 | Age at first sexual intercourse | G | A | NA | -0.0104 | 0.003 | 8.00E-04 |
| rs72986630 | Age at first sexual intercourse | T | C | NA | 0.0033 | 0.0044 | 0.46 |
| rs73026725 | Age at first sexual intercourse | A | C | NA | 0.0079 | 0.0029 | 0.006 |
| rs730384 | Age at first sexual intercourse | A | G | NA | 0.0051 | 0.0021 | 0.013 |
| rs73052033 | Age at first sexual intercourse | C | T | NA | 0.0036 | 0.0027 | 0.2 |
| rs73142879 | Age at first sexual intercourse | T | C | NA | 3.00E-04 | 0.0026 | 0.94 |
| rs73213484 | Age at first sexual intercourse | T | A | NA | 0.0059 | 0.003 | 0.037 |
| rs7331420 | Age at first sexual intercourse | A | G | NA | 0.0015 | 0.0023 | 0.51 |
| rs7332724 | Age at first sexual intercourse | T | C | NA | -0.007 | 0.0023 | 0.0033 |
| rs73344830 | Age at first sexual intercourse | G | A | NA | -0.0111 | 0.0021 | 1.90E-07 |
| rs7357754 | Age at first sexual intercourse | G | A | NA | -0.0066 | 0.0021 | 0.00098 |
| rs7359501 | Age at first sexual intercourse | T | C | NA | 0.0099 | 0.0021 | 1.70E-06 |
| rs736282 | Age at first sexual intercourse | C | T | NA | -0.0048 | 0.0021 | 0.02 |
| rs7442137 | Age at first sexual intercourse | T | C | NA | 0.0023 | 0.0021 | 0.31 |
| rs7442885 | Age at first sexual intercourse | G | C | NA | 0.0134 | 0.0025 | 1.70E-07 |
| rs745249 | Age at first sexual intercourse | T | C | NA | -0.0048 | 0.0023 | 0.033 |
| rs7498044 | Age at first sexual intercourse | A | G | NA | 0.0074 | 0.0025 | 0.0037 |
| rs7498665 | Age at first sexual intercourse | G | A | NA | 0.002 | 0.0021 | 0.38 |
| rs75035127 | Age at first sexual intercourse | G | A | NA | 0.011 | 0.006 | 0.064 |
| rs7551758 | Age at first sexual intercourse | G | T | NA | -0.0054 | 0.0021 | 0.012 |
| rs7575189 | Age at first sexual intercourse | A | G | NA | 0.0164 | 0.0021 | 1.90E-15 |
| rs7594904 | Age at first sexual intercourse | C | T | NA | 0.0042 | 0.0021 | 0.034 |
| rs76076331 | Age at first sexual intercourse | T | C | NA | 0.0095 | 0.0031 | 0.0023 |
| rs76702514 | Age at first sexual intercourse | G | C | NA | 0.0021 | 0.0025 | 0.38 |
| rs76878669 | Age at first sexual intercourse | G | C | NA | -0.0023 | 0.0025 | 0.35 |
| rs76954012 | Age at first sexual intercourse | A | T | NA | -0.0072 | 0.0035 | 0.054 |
| rs7704530 | Age at first sexual intercourse | A | G | NA | -0.0128 | 0.0023 | 2.90E-08 |
| rs7762794 | Age at first sexual intercourse | G | A | NA | -0.0042 | 0.0023 | 0.068 |
| rs77702622 | Age at first sexual intercourse | A | G | NA | -0.0106 | 0.0042 | 0.008 |
| rs7774 | Age at first sexual intercourse | A | C | NA | -0.0081 | 0.0023 | 0.00023 |
| rs77835879 | Age at first sexual intercourse | G | A | NA | -0.0063 | 0.0034 | 0.064 |
| rs778371 | Age at first sexual intercourse | G | A | NA | 0.0039 | 0.0023 | 0.094999 |
| rs7785195 | Age at first sexual intercourse | A | G | NA | 0.0142 | 0.0022 | 9.50E-11 |
| rs7803932 | Age at first sexual intercourse | A | G | NA | 0.0099 | 0.0028 | 0.00043 |
| rs78086698 | Age at first sexual intercourse | C | T | NA | 0.0053 | 0.0053 | 0.29 |
| rs7852189 | Age at first sexual intercourse | G | A | NA | -0.0013 | 0.0022 | 0.69 |
| rs7893571 | Age at first sexual intercourse | T | G | NA | -1.00E-04 | 0.0022 | 0.94 |
| rs7909331 | Age at first sexual intercourse | G | A | NA | -0.0156 | 0.0028 | 2.90E-08 |
| rs7920624 | Age at first sexual intercourse | T | A | NA | -0.0026 | 0.0021 | 0.24 |
| rs7921378 | Age at first sexual intercourse | C | G | NA | 0.0075 | 0.0021 | 0.00049 |
| rs7924036 | Age at first sexual intercourse | T | G | NA | 0.0098 | 0.0021 | 1.20E-06 |
| rs79265434 | Age at first sexual intercourse | G | A | NA | 0.0061 | 0.0032 | 0.073 |
| rs79269403 | Age at first sexual intercourse | A | G | NA | 0.0206 | 0.0025 | 3.10E-17 |
| rs7927195 | Age at first sexual intercourse | G | A | NA | -0.0152 | 0.0021 | 4.90E-13 |
| rs7928622 | Age at first sexual intercourse | T | A | NA | 0.0015 | 0.0022 | 0.48 |
| rs7938812 | Age at first sexual intercourse | G | T | NA | -0.0152 | 0.0021 | 7.70E-13 |
| rs79445414 | Age at first sexual intercourse | C | T | NA | 5.00E-04 | 0.0049 | 0.95 |
| rs7944782 | Age at first sexual intercourse | G | T | NA | -0.0041 | 0.0021 | 0.038 |
| rs7952102 | Age at first sexual intercourse | C | T | NA | 0.0031 | 0.0021 | 0.14 |
| rs7996639 | Age at first sexual intercourse | A | G | NA | -0.0033 | 0.0021 | 0.16 |
| rs80153284 | Age at first sexual intercourse | A | C | NA | 0.0044 | 0.0083 | 0.52 |
| rs8015400 | Age at first sexual intercourse | A | C | NA | -0.0022 | 0.0022 | 0.31 |
| rs8020034 | Age at first sexual intercourse | A | G | NA | 0.0098 | 0.0027 | 0.00039 |
| rs8112818 | Age at first sexual intercourse | G | A | NA | 0.0014 | 0.0021 | 0.41 |
| rs8132491 | Age at first sexual intercourse | A | G | NA | -0.0021 | 0.0023 | 0.36 |
| rs815163 | Age at first sexual intercourse | C | T | NA | -0.0024 | 0.0021 | 0.26 |
| rs862320 | Age at first sexual intercourse | T | C | NA | 0.0096 | 0.0021 | 8.40E-06 |
| rs879620 | Age at first sexual intercourse | T | C | NA | -0.0073 | 0.0021 | 0.00065 |
| rs892612 | Age at first sexual intercourse | C | A | NA | 0.005 | 0.0029 | 0.068 |
| rs9294260 | Age at first sexual intercourse | A | G | NA | -0.003 | 0.0021 | 0.12 |
| rs9296389 | Age at first sexual intercourse | C | G | NA | -0.001 | 0.0021 | 0.6 |
| rs9320493 | Age at first sexual intercourse | G | A | NA | -0.0036 | 0.0029 | 0.2 |
| rs9349956 | Age at first sexual intercourse | C | A | NA | 0.012 | 0.0027 | 1.30E-05 |
| rs935166 | Age at first sexual intercourse | A | G | NA | 0.0132 | 0.0021 | 1.10E-10 |
| rs9366863 | Age at first sexual intercourse | C | T | NA | 0.0053 | 0.0022 | 0.016 |
| rs9372625 | Age at first sexual intercourse | A | G | NA | 0.0062 | 0.0021 | 0.0026 |
| rs9386319 | Age at first sexual intercourse | G | A | NA | 0.0087 | 0.0021 | 4.70E-05 |
| rs9478496 | Age at first sexual intercourse | C | T | NA | -0.0046 | 0.0028 | 0.06 |
| rs9503598 | Age at first sexual intercourse | A | G | NA | 0.0083 | 0.0021 | 6.60E-05 |
| rs9514600 | Age at first sexual intercourse | G | C | NA | -0.0114 | 0.0021 | 2.40E-08 |
| rs9522173 | Age at first sexual intercourse | T | A | NA | 6.00E-04 | 0.0021 | 0.7 |
| rs9529119 | Age at first sexual intercourse | G | C | NA | -0.004 | 0.0025 | 0.094999 |
| rs9571687 | Age at first sexual intercourse | A | C | NA | 0.0024 | 0.0022 | 0.29 |
| rs9616906 | Age at first sexual intercourse | A | G | NA | 0.0027 | 0.0021 | 0.16 |
| rs9636107 | Age at first sexual intercourse | G | A | NA | -0.0101 | 0.0021 | 8.40E-07 |
| rs9643087 | Age at first sexual intercourse | T | C | NA | -0.0135 | 0.0021 | 9.40E-11 |
| rs969512 | Age at first sexual intercourse | T | A | NA | 0.007 | 0.0022 | 0.00098 |
| rs9704097 | Age at first sexual intercourse | A | C | NA | -0.0071 | 0.0021 | 0.00052 |
| rs9852062 | Age at first sexual intercourse | A | T | NA | 0.0081 | 0.0021 | 0.00014 |
| rs9860326 | Age at first sexual intercourse | G | C | NA | -0.009 | 0.0022 | 2.90E-05 |
| rs9882532 | Age at first sexual intercourse | C | T | NA | -0.0049 | 0.0022 | 0.018 |
| rs9888533 | Age at first sexual intercourse | T | C | NA | -0.0016 | 0.0021 | 0.43 |
| rs9951619 | Age at first sexual intercourse | G | T | NA | 0.0054 | 0.0025 | 0.017 |
| rs9964724 | Age at first sexual intercourse | T | C | NA | 0.0083 | 0.0022 | 0.00035 |
| rs1000237 | Average total household income before tax | A | T | 0.354594 | -0.003247 | 0.002765 | 0.24 |
| rs10073890 | Average total household income before tax | G | A | 0.738433 | -0.004804 | 0.003013 | 0.11 |
| rs10160769 | Average total household income before tax | C | G | 0.217366 | 0.010616 | 0.003241 | 0.0011 |
| rs1017529 | Average total household income before tax | A | C | 0.174538 | -0.002929 | 0.003547 | 0.41 |
| rs10423928 | Average total household income before tax | A | T | 0.194269 | 0.000424 | 0.003336 | 0.9 |
| rs10505836 | Average total household income before tax | C | A | 0.860101 | -0.004677 | 0.003839 | 0.22 |
| rs10510025 | Average total household income before tax | T | C | 0.246898 | -0.007346 | 0.003074 | 0.017 |
| rs1064213 | Average total household income before tax | A | G | 0.478016 | -0.007391 | 0.002644 | 0.0052 |
| rs10742752 | Average total household income before tax | C | T | 0.612241 | 0.005589 | 0.002715 | 0.04 |
| rs10752613 | Average total household income before tax | A | T | 0.290919 | -0.004608 | 0.002936 | 0.12 |
| rs10765775 | Average total household income before tax | A | G | 0.391994 | 0.008952 | 0.002721 | 0.001 |
| rs10773002 | Average total household income before tax | T | A | 0.75154 | -0.008159 | 0.003061 | 0.0077 |
| rs10797055 | Average total household income before tax | G | A | 0.478407 | -0.001523 | 0.002646 | 0.56 |
| rs10858054 | Average total household income before tax | T | G | 0.183557 | 0.000259 | 0.00341 | 0.94 |
| rs10861176 | Average total household income before tax | A | G | 0.733221 | 0.004017 | 0.002991 | 0.18 |
| rs10887578 | Average total household income before tax | C | G | 0.497439 | -0.006276 | 0.002661 | 0.018 |
| rs10887801 | Average total household income before tax | T | G | 0.43262 | 0.003608 | 0.002674 | 0.18 |
| rs10922907 | Average total household income before tax | T | A | 0.550057 | 0.016629 | 0.002662 | 4.20E-10 |
| rs10938398 | Average total household income before tax | A | G | 0.433853 | -0.004023 | 0.002675 | 0.13 |
| rs10963297 | Average total household income before tax | G | C | 0.239471 | 0.016897 | 0.003093 | 4.70E-08 |
| rs11000993 | Average total household income before tax | C | T | 0.123869 | 0.005523 | 0.00401 | 0.17 |
| rs11012732 | Average total household income before tax | G | A | 0.331194 | -0.004073 | 0.002811 | 0.15 |
| rs1105307 | Average total household income before tax | A | G | 0.258157 | -0.008694 | 0.003025 | 0.0041 |
| rs11079849 | Average total household income before tax | T | C | 0.328668 | 0.007259 | 0.00282 | 0.01 |
| rs11081529 | Average total household income before tax | C | T | 0.292794 | -0.008373 | 0.002911 | 0.004 |
| rs11099020 | Average total household income before tax | T | C | 0.64112 | 0.003273 | 0.00276 | 0.24 |
| rs11134679 | Average total household income before tax | G | A | 0.684779 | -0.001342 | 0.002853 | 0.64 |
| rs11165643 | Average total household income before tax | T | C | 0.590337 | 0.003144 | 0.002686 | 0.24 |
| rs11250094 | Average total household income before tax | C | G | 0.547929 | -0.004964 | 0.002664 | 0.062 |
| rs112633616 | Average total household income before tax | C | A | 0.033272 | 0.03214 | 0.007436 | 1.50E-05 |
| rs112687095 | Average total household income before tax | A | G | 0.173374 | 0.004831 | 0.003555 | 0.17 |
| rs113338260 | Average total household income before tax | C | T | 0.215931 | -0.001304 | 0.003219 | 0.69 |
| rs113520408 | Average total household income before tax | A | G | 0.282607 | 0.009249 | 0.002946 | 0.0017 |
| rs113624107 | Average total household income before tax | A | G | 0.225956 | -0.014279 | 0.003164 | 6.40E-06 |
| rs1143770 | Average total household income before tax | T | C | 0.572797 | 0.007815 | 0.002681 | 0.0036 |
| rs115000530 | Average total household income before tax | T | A | 0.055328 | 0.008196 | 0.00583 | 0.16 |
| rs115454970 | Average total household income before tax | T | G | 0.25543 | -0.008146 | 0.003079 | 0.0082 |
| rs11587347 | Average total household income before tax | G | C | 0.098161 | -0.005537 | 0.004467 | 0.22 |
| rs11620355 | Average total household income before tax | A | G | 0.087683 | 0.018248 | 0.004724 | 0.00011 |
| rs11635092 | Average total household income before tax | A | G | 0.360105 | -0.00146 | 0.002772 | 0.6 |
| rs116377258 | Average total household income before tax | G | A | 0.025698 | -0.011949 | 0.008364 | 0.15 |
| rs11693094 | Average total household income before tax | T | C | 0.464503 | -0.001337 | 0.002654 | 0.61 |
| rs11696755 | Average total household income before tax | C | T | 0.184167 | -0.005104 | 0.003422 | 0.14 |
| rs11709402 | Average total household income before tax | G | A | 0.278952 | -0.001322 | 0.002957 | 0.649999 |
| rs117118217 | Average total household income before tax | C | G | 0.017739 | 0.008364 | 0.010525 | 0.43 |
| rs11732657 | Average total household income before tax | A | G | 0.740257 | -0.013112 | 0.00303 | 1.50E-05 |
| rs118136827 | Average total household income before tax | T | G | 0.281515 | 0.001096 | 0.002941 | 0.709999 |
| rs11866420 | Average total household income before tax | G | C | 0.57464 | 0.006176 | 0.002681 | 0.021 |
| rs1198588 | Average total household income before tax | T | A | 0.797113 | 0.00596 | 0.003284 | 0.07 |
| rs12001437 | Average total household income before tax | C | T | 0.36781 | 0.002879 | 0.002741 | 0.29 |
| rs12033257 | Average total household income before tax | G | A | 0.382489 | 0.009299 | 0.002738 | 0.00068 |
| rs12140153 | Average total household income before tax | T | G | 0.094353 | -0.0002 | 0.004634 | 0.97 |
| rs12149660 | Average total household income before tax | A | G | 0.11522 | 0.003385 | 0.004163 | 0.42 |
| rs12156160 | Average total household income before tax | G | A | 0.150072 | -0.006739 | 0.003711 | 0.069 |
| rs12204714 | Average total household income before tax | T | C | 0.632989 | 0.012404 | 0.002744 | 6.20E-06 |
| rs12293670 | Average total household income before tax | G | A | 0.334599 | -0.001883 | 0.002832 | 0.51 |
| rs12303743 | Average total household income before tax | C | G | 0.098107 | -0.006341 | 0.00445 | 0.15 |
| rs12364470 | Average total household income before tax | G | T | 0.164473 | 3.64E-05 | 0.003566 | 0.99 |
| rs12375949 | Average total household income before tax | C | T | 0.561507 | 0.012138 | 0.002662 | 5.10E-06 |
| rs12375985 | Average total household income before tax | A | G | 0.354537 | 0.007705 | 0.002767 | 0.0054 |
| rs12427047 | Average total household income before tax | T | C | 0.242999 | 0.001229 | 0.003081 | 0.69 |
| rs12519073 | Average total household income before tax | T | C | 0.225139 | -0.004638 | 0.003173 | 0.14 |
| rs12523398 | Average total household income before tax | A | T | 0.174401 | -0.003095 | 0.003503 | 0.38 |
| rs12643771 | Average total household income before tax | T | C | 0.315372 | 0.012455 | 0.002867 | 1.40E-05 |
| rs1266874 | Average total household income before tax | G | A | 0.349332 | -0.000499 | 0.002773 | 0.86 |
| rs12681792 | Average total household income before tax | A | C | 0.192748 | 0.000272 | 0.003363 | 0.94 |
| rs12682775 | Average total household income before tax | C | T | 0.219377 | 0.008439 | 0.003201 | 0.0084 |
| rs12712510 | Average total household income before tax | C | T | 0.536148 | 0.003301 | 0.002667 | 0.22 |
| rs12714592 | Average total household income before tax | C | A | 0.27285 | -0.004028 | 0.002965 | 0.17 |
| rs12714702 | Average total household income before tax | G | A | 0.841196 | 0.004886 | 0.003616 | 0.18 |
| rs12757779 | Average total household income before tax | A | G | 0.224943 | -0.001931 | 0.003167 | 0.54 |
| rs12762034 | Average total household income before tax | C | T | 0.076824 | -0.001311 | 0.004963 | 0.79 |
| rs1286058 | Average total household income before tax | A | T | 0.704037 | 0.000496 | 0.002899 | 0.86 |
| rs12881629 | Average total household income before tax | G | A | 0.082524 | -0.001707 | 0.004799 | 0.719999 |
| rs12907546 | Average total household income before tax | A | G | 0.211418 | -0.010524 | 0.003249 | 0.0012 |
| rs12919291 | Average total household income before tax | C | G | 0.189611 | -0.003539 | 0.003383 | 0.3 |
| rs12940014 | Average total household income before tax | C | T | 0.524212 | 0.003264 | 0.002645 | 0.22 |
| rs12955211 | Average total household income before tax | A | T | 0.329251 | 0.009091 | 0.002833 | 0.0013 |
| rs12956148 | Average total household income before tax | A | C | 0.277035 | 0.000193 | 0.002958 | 0.95 |
| rs1296328 | Average total household income before tax | C | A | 0.558914 | -0.001522 | 0.002675 | 0.57 |
| rs12977787 | Average total household income before tax | A | G | 0.541025 | -0.00542 | 0.002653 | 0.041 |
| rs13030994 | Average total household income before tax | A | G | 0.48033 | -0.000415 | 0.002646 | 0.88 |
| rs13037326 | Average total household income before tax | T | C | 0.259321 | -0.001502 | 0.003018 | 0.62 |
| rs13090388 | Average total household income before tax | T | C | 0.306846 | 0.022034 | 0.002866 | 1.50E-14 |
| rs13107325 | Average total household income before tax | T | C | 0.074647 | -0.02508 | 0.005035 | 6.30E-07 |
| rs13141210 | Average total household income before tax | T | C | 0.530939 | 0.010186 | 0.002657 | 0.00013 |
| rs13145650 | Average total household income before tax | T | C | 0.914208 | -0.011452 | 0.004728 | 0.015 |
| rs13175535 | Average total household income before tax | A | G | 0.295248 | 0.003449 | 0.002942 | 0.24 |
| rs13195636 | Average total household income before tax | C | A | 0.115004 | 0.005968 | 0.004137 | 0.15 |
| rs1320251 | Average total household income before tax | T | C | 0.455212 | 0.003214 | 0.002663 | 0.23 |
| rs13218383 | Average total household income before tax | G | C | 0.335075 | -0.002942 | 0.002801 | 0.29 |
| rs13233308 | Average total household income before tax | T | C | 0.483238 | 0.000464 | 0.002645 | 0.86 |
| rs13248187 | Average total household income before tax | C | T | 0.268428 | 0.000242 | 0.002997 | 0.94 |
| rs13261666 | Average total household income before tax | T | G | 0.50667 | -0.000851 | 0.002645 | 0.75 |
| rs1327259 | Average total household income before tax | G | A | 0.387736 | 0.003912 | 0.002723 | 0.15 |
| rs13292699 | Average total household income before tax | C | A | 0.433917 | 0.004545 | 0.002671 | 0.089 |
| rs1330199 | Average total household income before tax | T | G | 0.48344 | 0.005185 | 0.002655 | 0.051 |
| rs13307225 | Average total household income before tax | A | G | 0.893748 | 0.009064 | 0.004313 | 0.036 |
| rs13317303 | Average total household income before tax | A | C | 0.150415 | 0.00051 | 0.003704 | 0.89 |
| rs1334297 | Average total household income before tax | A | G | 0.736211 | 0.012007 | 0.003006 | 6.50E-05 |
| rs13422673 | Average total household income before tax | T | C | 0.460975 | -0.005751 | 0.002652 | 0.03 |
| rs13427822 | Average total household income before tax | G | A | 0.271426 | 0.009736 | 0.003005 | 0.0012 |
| rs1346841 | Average total household income before tax | A | G | 0.405155 | 0.006047 | 0.002703 | 0.025 |
| rs1360201 | Average total household income before tax | T | C | 0.481874 | -0.010573 | 0.002644 | 6.40E-05 |
| rs1363862 | Average total household income before tax | A | G | 0.277234 | -0.008544 | 0.002968 | 0.004 |
| rs13642 | Average total household income before tax | T | A | 0.360441 | 0.004378 | 0.002752 | 0.11 |
| rs1391438 | Average total household income before tax | C | T | 0.687317 | -0.011047 | 0.002852 | 0.00011 |
| rs1392816 | Average total household income before tax | T | C | 0.388358 | 0.0039 | 0.002735 | 0.15 |
| rs140159717 | Average total household income before tax | T | C | 0.082017 | 0.00479 | 0.004962 | 0.33 |
| rs1438945 | Average total household income before tax | A | T | 0.714744 | 0.001496 | 0.002941 | 0.61 |
| rs1441264 | Average total household income before tax | A | G | 0.593406 | 0.000859 | 0.00275 | 0.75 |
| rs1450782 | Average total household income before tax | G | T | 0.563423 | -0.004829 | 0.002667 | 0.07 |
| rs1455350 | Average total household income before tax | A | T | 0.477454 | -0.017234 | 0.00266 | 9.20E-11 |
| rs1458156 | Average total household income before tax | T | C | 0.489006 | 0.002439 | 0.002646 | 0.36 |
| rs1471740 | Average total household income before tax | C | T | 0.740486 | 0.000594 | 0.003018 | 0.84 |
| rs147568678 | Average total household income before tax | C | T | 0.237745 | 0.000952 | 0.003118 | 0.760001 |
| rs1477290 | Average total household income before tax | C | T | 0.137462 | 0.007601 | 0.003873 | 0.05 |
| rs1503526 | Average total household income before tax | C | T | 0.479974 | -0.006683 | 0.002645 | 0.012 |
| rs152603 | Average total household income before tax | G | A | 0.366013 | 0.010847 | 0.002741 | 7.60E-05 |
| rs1565735 | Average total household income before tax | A | T | 0.203041 | 0.005926 | 0.003312 | 0.074 |
| rs1566085 | Average total household income before tax | T | G | 0.547011 | 0.004792 | 0.002677 | 0.073 |
| rs1582931 | Average total household income before tax | A | G | 0.472892 | -0.004856 | 0.002671 | 0.069 |
| rs1584469 | Average total household income before tax | T | C | 0.29291 | -0.008219 | 0.002904 | 0.0046 |
| rs1609010 | Average total household income before tax | G | A | 0.565842 | -0.003219 | 0.002669 | 0.23 |
| rs1671770 | Average total household income before tax | C | A | 0.827956 | -0.002588 | 0.003502 | 0.46 |
| rs16846140 | Average total household income before tax | G | A | 0.337679 | -0.010874 | 0.002799 | 1.00E-04 |
| rs16846463 | Average total household income before tax | G | A | 0.10012 | -0.016876 | 0.004404 | 0.00013 |
| rs16854920 | Average total household income before tax | C | T | 0.344303 | 0.003021 | 0.002777 | 0.28 |
| rs1689510 | Average total household income before tax | C | G | 0.338491 | 0.010181 | 0.002795 | 0.00027 |
| rs16916303 | Average total household income before tax | G | A | 0.119688 | 0.003995 | 0.004116 | 0.33 |
| rs16995054 | Average total household income before tax | T | C | 0.207623 | -0.006459 | 0.003271 | 0.048 |
| rs17193211 | Average total household income before tax | T | C | 0.066802 | 0.007742 | 0.005385 | 0.15 |
| rs17194490 | Average total household income before tax | T | G | 0.164944 | 0.000103 | 0.003572 | 0.98 |
| rs17399739 | Average total household income before tax | G | A | 0.069015 | -0.003354 | 0.005226 | 0.52 |
| rs17565975 | Average total household income before tax | A | G | 0.562994 | -0.005343 | 0.002678 | 0.046 |
| rs17598675 | Average total household income before tax | C | T | 0.482457 | 0.004428 | 0.002665 | 0.097 |
| rs176218 | Average total household income before tax | T | G | 0.190918 | 0.015817 | 0.003375 | 2.80E-06 |
| rs1778830 | Average total household income before tax | A | G | 0.362142 | -0.004066 | 0.002758 | 0.14 |
| rs1788808 | Average total household income before tax | G | A | 0.495479 | 0.003628 | 0.002648 | 0.17 |
| rs1834144 | Average total household income before tax | A | C | 0.37227 | -0.007811 | 0.002743 | 0.0044 |
| rs1860002 | Average total household income before tax | T | C | 0.536867 | -0.00298 | 0.002665 | 0.26 |
| rs1899896 | Average total household income before tax | T | C | 0.297503 | -0.00137 | 0.002905 | 0.64 |
| rs1901512 | Average total household income before tax | C | T | 0.693997 | 0.006854 | 0.002889 | 0.018 |
| rs1915019 | Average total household income before tax | G | A | 0.760301 | -0.000326 | 0.003101 | 0.92 |
| rs1919243 | Average total household income before tax | C | T | 0.487021 | -0.003736 | 0.002681 | 0.16 |
| rs1950829 | Average total household income before tax | G | A | 0.518535 | 0.005163 | 0.002649 | 0.051 |
| rs1967772 | Average total household income before tax | A | G | 0.284927 | 0.005122 | 0.002945 | 0.081999 |
| rs2035936 | Average total household income before tax | T | G | 0.055843 | -0.00502 | 0.00584 | 0.39 |
| rs2076603 | Average total household income before tax | A | G | 0.644548 | 0.005919 | 0.002759 | 0.032 |
| rs2084572 | Average total household income before tax | G | A | 0.449935 | 0.006494 | 0.002657 | 0.015 |
| rs2102278 | Average total household income before tax | G | A | 0.322161 | -0.008599 | 0.002833 | 0.0024 |
| rs2133561 | Average total household income before tax | T | A | 0.610502 | 0.001019 | 0.00274 | 0.709999 |
| rs213518 | Average total household income before tax | C | T | 0.145753 | 0.005707 | 0.00375 | 0.13 |
| rs2153740 | Average total household income before tax | G | A | 0.480082 | -0.002806 | 0.00266 | 0.29 |
| rs215634 | Average total household income before tax | G | A | 0.612301 | 0.000239 | 0.002722 | 0.93 |
| rs2172131 | Average total household income before tax | C | T | 0.579031 | 0.005524 | 0.002679 | 0.039 |
| rs217336 | Average total household income before tax | A | C | 0.437536 | 0.004529 | 0.002665 | 0.089 |
| rs2174752 | Average total household income before tax | T | G | 0.450394 | -0.007338 | 0.002658 | 0.0058 |
| rs2176337 | Average total household income before tax | T | A | 0.315363 | -0.001865 | 0.002846 | 0.51 |
| rs217672 | Average total household income before tax | C | A | 0.271544 | -0.005667 | 0.002981 | 0.056999 |
| rs2214123 | Average total household income before tax | G | A | 0.647447 | 0.00014 | 0.002794 | 0.96 |
| rs2234458 | Average total household income before tax | T | C | 0.63924 | 0.005752 | 0.00275 | 0.036 |
| rs2253310 | Average total household income before tax | G | C | 0.626876 | 0.010572 | 0.002733 | 0.00011 |
| rs2281819 | Average total household income before tax | A | T | 0.230162 | 0.002269 | 0.003145 | 0.47 |
| rs2283076 | Average total household income before tax | G | A | 0.229089 | -0.017159 | 0.003158 | 5.50E-08 |
| rs2289379 | Average total household income before tax | T | C | 0.395999 | 0.006408 | 0.002714 | 0.018 |
| rs2302761 | Average total household income before tax | T | C | 0.212644 | 0.00898 | 0.003232 | 0.0055 |
| rs2306593 | Average total household income before tax | T | C | 0.488436 | 0.006845 | 0.002651 | 0.0098 |
| rs2307111 | Average total household income before tax | C | T | 0.394733 | 0.00623 | 0.002708 | 0.021 |
| rs2332700 | Average total household income before tax | G | C | 0.752223 | -0.00334 | 0.003069 | 0.28 |
| rs2333321 | Average total household income before tax | G | A | 0.792786 | 0.004976 | 0.003263 | 0.13 |
| rs2342892 | Average total household income before tax | G | T | 0.516205 | -0.00027 | 0.002645 | 0.92 |
| rs2347526 | Average total household income before tax | C | T | 0.657916 | 0.01137 | 0.002784 | 4.40E-05 |
| rs2396625 | Average total household income before tax | A | T | 0.421542 | 0.008477 | 0.002693 | 0.0016 |
| rs2398861 | Average total household income before tax | G | A | 0.258914 | -0.009959 | 0.003031 | 0.001 |
| rs240963 | Average total household income before tax | C | T | 0.840944 | 0.004868 | 0.003619 | 0.18 |
| rs242093 | Average total household income before tax | A | G | 0.569732 | -0.008763 | 0.002694 | 0.0011 |
| rs2433733 | Average total household income before tax | A | G | 0.677871 | 0.00063 | 0.002829 | 0.82 |
| rs2456020 | Average total household income before tax | T | C | 0.230216 | 0.012446 | 0.003143 | 7.50E-05 |
| rs2482356 | Average total household income before tax | C | T | 0.42939 | 0.001311 | 0.002667 | 0.62 |
| rs252761 | Average total household income before tax | T | G | 0.587828 | -0.002894 | 0.0027 | 0.28 |
| rs2554835 | Average total household income before tax | A | G | 0.396801 | 0.004388 | 0.00271 | 0.11 |
| rs2568958 | Average total household income before tax | A | G | 0.604059 | 0.008567 | 0.002698 | 0.0015 |
| rs2606228 | Average total household income before tax | C | A | 0.646596 | 0.00863 | 0.002792 | 0.002 |
| rs2612030 | Average total household income before tax | C | T | 0.838367 | 0.001927 | 0.003602 | 0.59 |
| rs2616143 | Average total household income before tax | A | G | 0.319851 | 0.004598 | 0.002842 | 0.11 |
| rs266047 | Average total household income before tax | A | G | 0.535447 | 0.002261 | 0.002648 | 0.39 |
| rs2678204 | Average total household income before tax | G | T | 0.340057 | -0.016219 | 0.002791 | 6.20E-09 |
| rs2781668 | Average total household income before tax | T | C | 0.165311 | -0.002803 | 0.003566 | 0.43 |
| rs2787101 | Average total household income before tax | T | C | 0.60419 | 0.009438 | 0.002719 | 0.00052 |
| rs2819336 | Average total household income before tax | C | T | 0.637837 | -0.01137 | 0.002755 | 3.70E-05 |
| rs28373063 | Average total household income before tax | C | G | 0.178115 | 0.008723 | 0.003452 | 0.012 |
| rs28404639 | Average total household income before tax | T | C | 0.366275 | -0.004652 | 0.002749 | 0.091 |
| rs28489620 | Average total household income before tax | A | G | 0.290283 | 0.006755 | 0.002936 | 0.021 |
| rs28513670 | Average total household income before tax | G | A | 0.171175 | 0.007706 | 0.003509 | 0.028 |
| rs2875762 | Average total household income before tax | C | G | 0.243029 | -0.007772 | 0.003095 | 0.012 |
| rs2876520 | Average total household income before tax | G | C | 0.467893 | -0.010371 | 0.002674 | 0.00011 |
| rs2920503 | Average total household income before tax | T | C | 0.28541 | 0.002921 | 0.002943 | 0.32 |
| rs2923431 | Average total household income before tax | C | G | 0.632608 | 0.006689 | 0.002744 | 0.015 |
| rs293566 | Average total household income before tax | C | T | 0.331312 | -0.005486 | 0.002817 | 0.051 |
| rs2962334 | Average total household income before tax | T | G | 0.020055 | 0.01099 | 0.00942 | 0.24 |
| rs2971970 | Average total household income before tax | G | T | 0.782411 | 0.011211 | 0.003208 | 0.00047 |
| rs301800 | Average total household income before tax | C | T | 0.824063 | -0.013505 | 0.003468 | 9.80E-05 |
| rs30266 | Average total household income before tax | A | G | 0.328268 | -0.011876 | 0.002819 | 2.50E-05 |
| rs317656 | Average total household income before tax | A | T | 0.724839 | 0.001 | 0.002962 | 0.74 |
| rs320693 | Average total household income before tax | C | G | 0.453746 | 0.004206 | 0.002652 | 0.11 |
| rs3211995 | Average total household income before tax | A | G | 0.160085 | 0.008163 | 0.003625 | 0.024 |
| rs329118 | Average total household income before tax | T | C | 0.419435 | 0.009871 | 0.00268 | 0.00023 |
| rs34025316 | Average total household income before tax | T | C | 0.33762 | -0.000548 | 0.002811 | 0.85 |
| rs34045288 | Average total household income before tax | T | C | 0.333964 | -0.008143 | 0.002805 | 0.0037 |
| rs34234296 | Average total household income before tax | A | G | 0.392549 | 0.000285 | 0.002737 | 0.92 |
| rs34481751 | Average total household income before tax | A | C | 0.165733 | -0.004834 | 0.003608 | 0.18 |
| rs34517439 | Average total household income before tax | A | C | 0.121793 | -0.012945 | 0.004089 | 0.0015 |
| rs347551 | Average total household income before tax | G | C | 0.472145 | 0.002253 | 0.002691 | 0.4 |
| rs34811474 | Average total household income before tax | A | G | 0.231597 | 0.017778 | 0.003134 | 1.40E-08 |
| rs35154326 | Average total household income before tax | G | A | 0.274354 | 0.009771 | 0.002989 | 0.0011 |
| rs35309068 | Average total household income before tax | G | T | 0.433378 | 0.010797 | 0.002664 | 5.10E-05 |
| rs354155 | Average total household income before tax | C | G | 0.088939 | -0.002931 | 0.004641 | 0.53 |
| rs35417702 | Average total household income before tax | T | C | 0.524522 | -0.007283 | 0.002647 | 0.0059 |
| rs35475880 | Average total household income before tax | T | G | 0.207325 | -0.012614 | 0.003271 | 0.00012 |
| rs35532491 | Average total household income before tax | T | A | 0.103595 | 0.017159 | 0.00434 | 7.70E-05 |
| rs355777 | Average total household income before tax | C | G | 0.407376 | -0.005081 | 0.002695 | 0.059 |
| rs35867081 | Average total household income before tax | G | A | 0.511712 | -0.005069 | 0.002659 | 0.056999 |
| rs36007635 | Average total household income before tax | A | G | 0.137907 | 0.005884 | 0.003837 | 0.13 |
| rs36061954 | Average total household income before tax | T | C | 0.398737 | -0.009077 | 0.0027 | 0.00077 |
| rs36119825 | Average total household income before tax | A | G | 0.442455 | -0.000971 | 0.002655 | 0.709999 |
| rs363096 | Average total household income before tax | C | T | 0.57559 | 0.010118 | 0.002677 | 0.00016 |
| rs3747631 | Average total household income before tax | C | G | 0.209719 | 0.021212 | 0.003244 | 6.20E-11 |
| rs3764625 | Average total household income before tax | G | T | 0.58771 | 0.001988 | 0.00269 | 0.46 |
| rs3770754 | Average total household income before tax | G | C | 0.359617 | 0.003621 | 0.002754 | 0.19 |
| rs3784710 | Average total household income before tax | C | T | 0.226387 | 0.001756 | 0.003152 | 0.58 |
| rs3800546 | Average total household income before tax | G | C | 0.255771 | -0.004221 | 0.003035 | 0.16 |
| rs3807865 | Average total household income before tax | A | G | 0.412376 | -0.008128 | 0.002681 | 0.0024 |
| rs3809634 | Average total household income before tax | G | A | 0.314159 | 0.011115 | 0.002875 | 0.00011 |
| rs3814883 | Average total household income before tax | T | C | 0.482806 | -0.00051 | 0.002654 | 0.85 |
| rs3845344 | Average total household income before tax | T | C | 0.391345 | -0.002931 | 0.002707 | 0.28 |
| rs3851998 | Average total household income before tax | G | C | 0.74282 | 0.005694 | 0.003037 | 0.061 |
| rs3896224 | Average total household income before tax | G | A | 0.414702 | 0.009544 | 0.002705 | 0.00042 |
| rs3897821 | Average total household income before tax | G | A | 0.333336 | -0.011297 | 0.002802 | 5.50E-05 |
| rs3901286 | Average total household income before tax | A | C | 0.152551 | 0.00446 | 0.003684 | 0.23 |
| rs394608 | Average total household income before tax | C | T | 0.537653 | -0.005593 | 0.002662 | 0.036 |
| rs40071 | Average total household income before tax | C | T | 0.179431 | 0.001408 | 0.003454 | 0.68 |
| rs4044321 | Average total household income before tax | G | A | 0.64087 | 0.002382 | 0.002761 | 0.39 |
| rs4055791 | Average total household income before tax | T | C | 0.416615 | 0.002761 | 0.002684 | 0.3 |
| rs406388 | Average total household income before tax | G | C | 0.176971 | -0.003747 | 0.003473 | 0.28 |
| rs4148155 | Average total household income before tax | G | A | 0.113165 | 0.004736 | 0.004165 | 0.26 |
| rs4261944 | Average total household income before tax | G | T | 0.365066 | 0.001891 | 0.002755 | 0.49 |
| rs4267103 | Average total household income before tax | C | T | 0.18606 | -0.004586 | 0.003402 | 0.18 |
| rs429343 | Average total household income before tax | G | A | 0.576777 | -0.000584 | 0.002679 | 0.83 |
| rs429358 | Average total household income before tax | C | T | 0.154217 | 0.004468 | 0.003663 | 0.22 |
| rs4328757 | Average total household income before tax | T | C | 0.61343 | 0.007728 | 0.002716 | 0.0044 |
| rs4352658 | Average total household income before tax | T | C | 0.080932 | -0.017456 | 0.004849 | 0.00032 |
| rs4382592 | Average total household income before tax | G | T | 0.69914 | 0.000315 | 0.002886 | 0.91 |
| rs4419475 | Average total household income before tax | T | A | 0.407226 | -0.005089 | 0.002691 | 0.059 |
| rs4439537 | Average total household income before tax | C | T | 0.525062 | 0.00314 | 0.002654 | 0.24 |
| rs4477562 | Average total household income before tax | T | C | 0.128581 | -0.00181 | 0.003981 | 0.649999 |
| rs4482463 | Average total household income before tax | A | C | 0.922886 | -0.003482 | 0.004972 | 0.48 |
| rs4613074 | Average total household income before tax | C | T | 0.184922 | -0.000105 | 0.003407 | 0.98 |
| rs4648450 | Average total household income before tax | A | C | 0.46725 | 0.001038 | 0.002664 | 0.7 |
| rs4653164 | Average total household income before tax | T | C | 0.690829 | 0.001803 | 0.002859 | 0.53 |
| rs4671328 | Average total household income before tax | G | T | 0.551291 | 0.004335 | 0.002679 | 0.11 |
| rs4700393 | Average total household income before tax | G | A | 0.519621 | 0.01848 | 0.002649 | 3.10E-12 |
| rs4702 | Average total household income before tax | A | G | 0.556321 | -0.005627 | 0.002663 | 0.035 |
| rs4709807 | Average total household income before tax | C | T | 0.759057 | 0.006991 | 0.003099 | 0.024 |
| rs4737188 | Average total household income before tax | T | A | 0.474369 | 0.005854 | 0.002651 | 0.027 |
| rs4757144 | Average total household income before tax | A | G | 0.590396 | 0.005106 | 0.00269 | 0.058 |
| rs4757957 | Average total household income before tax | C | G | 0.680294 | 0.010795 | 0.002842 | 0.00015 |
| rs4764949 | Average total household income before tax | G | A | 0.325639 | 0.00148 | 0.002822 | 0.6 |
| rs4790292 | Average total household income before tax | A | C | 0.154122 | 0.004138 | 0.003678 | 0.26 |
| rs4810227 | Average total household income before tax | A | G | 0.618974 | 0.010448 | 0.002743 | 0.00014 |
| rs4812325 | Average total household income before tax | A | G | 0.609807 | 0.001823 | 0.00273 | 0.5 |
| rs4832298 | Average total household income before tax | T | C | 0.686743 | 0.001267 | 0.002848 | 0.66 |
| rs4846724 | Average total household income before tax | A | G | 0.533524 | 0.007677 | 0.002659 | 0.0039 |
| rs4876611 | Average total household income before tax | G | A | 0.720459 | 0.000596 | 0.002948 | 0.84 |
| rs4895799 | Average total household income before tax | T | C | 0.585037 | 0.003711 | 0.002692 | 0.17 |
| rs4958702 | Average total household income before tax | C | T | 0.572405 | 0.001643 | 0.002669 | 0.54 |
| rs4961705 | Average total household income before tax | C | G | 0.347731 | 0.00472 | 0.002823 | 0.094999 |
| rs512121 | Average total household income before tax | C | T | 0.19183 | 0.005151 | 0.00337 | 0.13 |
| rs539515 | Average total household income before tax | C | A | 0.204829 | -0.015452 | 0.003276 | 2.40E-06 |
| rs55658481 | Average total household income before tax | A | G | 0.339265 | -0.006432 | 0.00279 | 0.021 |
| rs55707359 | Average total household income before tax | G | T | 0.015414 | -0.014177 | 0.010869 | 0.19 |
| rs55714539 | Average total household income before tax | C | A | 0.342964 | -0.004202 | 0.002807 | 0.13 |
| rs55726687 | Average total household income before tax | A | G | 0.209982 | 0.004631 | 0.003243 | 0.15 |
| rs55736314 | Average total household income before tax | G | C | 0.404337 | 0.011854 | 0.002701 | 1.10E-05 |
| rs558887 | Average total household income before tax | G | A | 0.307934 | 0.003956 | 0.002872 | 0.17 |
| rs56094641 | Average total household income before tax | G | A | 0.404584 | -0.001394 | 0.002694 | 0.6 |
| rs56133507 | Average total household income before tax | G | T | 0.196764 | -0.006348 | 0.003318 | 0.056 |
| rs56161855 | Average total household income before tax | T | A | 0.132686 | 0.005526 | 0.0039 | 0.16 |
| rs56203622 | Average total household income before tax | C | T | 0.145276 | -0.006179 | 0.003749 | 0.099001 |
| rs56335113 | Average total household income before tax | G | A | 0.690984 | 0.000388 | 0.002876 | 0.89 |
| rs56356382 | Average total household income before tax | C | T | 0.192132 | 0.009764 | 0.003371 | 0.0038 |
| rs56391344 | Average total household income before tax | A | G | 0.254322 | 0.014534 | 0.003042 | 1.80E-06 |
| rs56399737 | Average total household income before tax | T | C | 0.449554 | 0.004222 | 0.002667 | 0.11 |
| rs56858768 | Average total household income before tax | A | G | 0.296646 | -0.002209 | 0.002905 | 0.450001 |
| rs57636386 | Average total household income before tax | C | T | 0.083914 | 0.005339 | 0.004783 | 0.26 |
| rs58120505 | Average total household income before tax | C | T | 0.433761 | 0.004637 | 0.002665 | 0.081999 |
| rs59086897 | Average total household income before tax | A | T | 0.487327 | -0.004806 | 0.002642 | 0.069 |
| rs59237168 | Average total household income before tax | C | T | 0.215786 | 0.00272 | 0.003218 | 0.4 |
| rs5995843 | Average total household income before tax | G | A | 0.345597 | 0.005203 | 0.002782 | 0.061 |
| rs60764613 | Average total household income before tax | T | G | 0.144889 | -0.00129 | 0.003777 | 0.73 |
| rs6123924 | Average total household income before tax | G | A | 0.153338 | -0.009786 | 0.003682 | 0.0079 |
| rs6134916 | Average total household income before tax | T | C | 0.492985 | 0.001452 | 0.00265 | 0.58 |
| rs61813324 | Average total household income before tax | T | C | 0.135838 | -0.004461 | 0.003912 | 0.25 |
| rs61828088 | Average total household income before tax | A | G | 0.110499 | -0.000338 | 0.004215 | 0.94 |
| rs61903695 | Average total household income before tax | G | A | 0.254898 | -0.006239 | 0.003038 | 0.04 |
| rs61914045 | Average total household income before tax | A | G | 0.201971 | -0.006727 | 0.003307 | 0.042 |
| rs62097985 | Average total household income before tax | T | C | 0.418569 | -0.015383 | 0.002691 | 1.10E-08 |
| rs62107261 | Average total household income before tax | C | T | 0.048265 | 0.005581 | 0.006187 | 0.37 |
| rs62134195 | Average total household income before tax | T | C | 0.040934 | 0.015888 | 0.006757 | 0.019 |
| rs62176243 | Average total household income before tax | T | A | 0.24492 | 0.00424 | 0.003071 | 0.17 |
| rs62176993 | Average total household income before tax | A | G | 0.401301 | 0.001112 | 0.002707 | 0.68 |
| rs62190049 | Average total household income before tax | C | G | 0.39034 | 0.00159 | 0.00273 | 0.56 |
| rs62439690 | Average total household income before tax | A | G | 0.261907 | -0.003393 | 0.003042 | 0.26 |
| rs6265 | Average total household income before tax | T | C | 0.188599 | 0.009737 | 0.003379 | 0.004 |
| rs6531639 | Average total household income before tax | A | G | 0.247663 | 0.00914 | 0.003131 | 0.0035 |
| rs6545714 | Average total household income before tax | A | G | 0.601495 | 0.000359 | 0.002704 | 0.89 |
| rs6546857 | Average total household income before tax | G | A | 0.23775 | -0.014009 | 0.003112 | 6.70E-06 |
| rs6560906 | Average total household income before tax | C | T | 0.691888 | 0.000529 | 0.002863 | 0.85 |
| rs6567160 | Average total household income before tax | C | T | 0.232904 | -0.003651 | 0.003128 | 0.24 |
| rs6575340 | Average total household income before tax | A | G | 0.635895 | -0.003851 | 0.002754 | 0.16 |
| rs66511648 | Average total household income before tax | C | T | 0.284804 | 0.00232 | 0.002941 | 0.43 |
| rs66679256 | Average total household income before tax | T | C | 0.445408 | 0.001309 | 0.002662 | 0.62 |
| rs6669341 | Average total household income before tax | G | A | 0.58227 | -0.004811 | 0.002679 | 0.073 |
| rs6682438 | Average total household income before tax | C | T | 0.673097 | 0.002966 | 0.002816 | 0.29 |
| rs6688826 | Average total household income before tax | C | T | 0.298254 | -0.005986 | 0.002886 | 0.038 |
| rs6707827 | Average total household income before tax | G | A | 0.703067 | -0.010933 | 0.002913 | 0.00017 |
| rs6710091 | Average total household income before tax | G | C | 0.348034 | 0.001243 | 0.002773 | 0.649999 |
| rs6719762 | Average total household income before tax | C | T | 0.473383 | -0.009975 | 0.002655 | 0.00017 |
| rs6731967 | Average total household income before tax | C | G | 0.235168 | -0.009662 | 0.003126 | 0.002 |
| rs6744646 | Average total household income before tax | G | A | 0.828472 | -0.007328 | 0.003506 | 0.037 |
| rs6744794 | Average total household income before tax | G | C | 0.622487 | -0.011808 | 0.002723 | 1.50E-05 |
| rs6752979 | Average total household income before tax | A | G | 0.316904 | -0.004052 | 0.00284 | 0.15 |
| rs6774894 | Average total household income before tax | A | T | 0.358197 | 0.001773 | 0.002754 | 0.52 |
| rs6798742 | Average total household income before tax | G | A | 0.297973 | -0.000954 | 0.002892 | 0.74 |
| rs6803651 | Average total household income before tax | T | G | 0.434985 | 0.002147 | 0.002681 | 0.42 |
| rs6805241 | Average total household income before tax | C | T | 0.230572 | -0.010911 | 0.003144 | 0.00052 |
| rs6843852 | Average total household income before tax | T | C | 0.508323 | -0.004527 | 0.002644 | 0.087 |
| rs6943762 | Average total household income before tax | C | T | 0.126924 | 0.007336 | 0.003989 | 0.065999 |
| rs6959891 | Average total household income before tax | G | A | 0.281599 | -0.00484 | 0.002952 | 0.1 |
| rs6962980 | Average total household income before tax | C | A | 0.556092 | 0.006018 | 0.002659 | 0.024 |
| rs6974218 | Average total household income before tax | C | A | 0.382996 | 0.006327 | 0.002724 | 0.02 |
| rs698147 | Average total household income before tax | G | A | 0.543662 | 0.002537 | 0.002656 | 0.34 |
| rs7008955 | Average total household income before tax | G | T | 0.526996 | 0.000929 | 0.002662 | 0.73 |
| rs7012546 | Average total household income before tax | T | C | 0.414637 | 0.00552 | 0.002684 | 0.04 |
| rs7029718 | Average total household income before tax | A | G | 0.416497 | 0.026654 | 0.002683 | 3.00E-23 |
| rs7031698 | Average total household income before tax | C | T | 0.77599 | 0.011249 | 0.003173 | 0.00039 |
| rs7034554 | Average total household income before tax | G | A | 0.373542 | 0.000817 | 0.00273 | 0.760001 |
| rs7038943 | Average total household income before tax | C | T | 0.339004 | 0.007991 | 0.002788 | 0.0041 |
| rs7079070 | Average total household income before tax | A | G | 0.454601 | 0.000777 | 0.002656 | 0.77 |
| rs708228 | Average total household income before tax | T | C | 0.327511 | -0.014852 | 0.002818 | 1.40E-07 |
| rs7124681 | Average total household income before tax | A | C | 0.40801 | -0.000331 | 0.002683 | 0.9 |
| rs7132908 | Average total household income before tax | A | G | 0.384534 | -0.003941 | 0.00272 | 0.15 |
| rs71646142 | Average total household income before tax | T | C | 0.191826 | 0.009917 | 0.003379 | 0.0033 |
| rs7201895 | Average total household income before tax | A | G | 0.354154 | 0.002966 | 0.002783 | 0.29 |
| rs7206608 | Average total household income before tax | G | C | 0.321805 | -0.00257 | 0.002832 | 0.36 |
| rs7218014 | Average total household income before tax | C | T | 0.197019 | -0.007556 | 0.003332 | 0.023 |
| rs7233920 | Average total household income before tax | A | G | 0.226522 | -0.007946 | 0.003179 | 0.012 |
| rs7236339 | Average total household income before tax | A | G | 0.227737 | -0.00936 | 0.003172 | 0.0032 |
| rs723672 | Average total household income before tax | T | C | 0.431336 | 0.000146 | 0.002684 | 0.96 |
| rs7250833 | Average total household income before tax | T | C | 0.288618 | -0.004292 | 0.002924 | 0.14 |
| rs7259070 | Average total household income before tax | C | T | 0.595884 | -0.002249 | 0.002719 | 0.41 |
| rs72673947 | Average total household income before tax | G | A | 0.106797 | -0.011552 | 0.004302 | 0.0072 |
| rs7278859 | Average total household income before tax | T | A | 0.308721 | 0.006855 | 0.00288 | 0.017 |
| rs72887338 | Average total household income before tax | C | T | 0.386282 | -0.003147 | 0.00272 | 0.25 |
| rs72892910 | Average total household income before tax | T | G | 0.171942 | 0.004108 | 0.003509 | 0.24 |
| rs72910629 | Average total household income before tax | G | A | 0.135881 | -0.001371 | 0.003881 | 0.719999 |
| rs72986630 | Average total household income before tax | T | C | 0.064462 | 0.000918 | 0.005585 | 0.87 |
| rs73026725 | Average total household income before tax | A | C | 0.153802 | -0.008147 | 0.003671 | 0.026 |
| rs730384 | Average total household income before tax | A | G | 0.440676 | 0.008256 | 0.00268 | 0.0021 |
| rs73052033 | Average total household income before tax | C | T | 0.184933 | -0.002374 | 0.003408 | 0.49 |
| rs73142879 | Average total household income before tax | T | C | 0.191799 | 0.012785 | 0.003371 | 0.00015 |
| rs73213484 | Average total household income before tax | T | A | 0.141171 | -0.001163 | 0.0038 | 0.760001 |
| rs7331420 | Average total household income before tax | A | G | 0.285503 | 0.013414 | 0.002941 | 5.10E-06 |
| rs7332724 | Average total household income before tax | T | C | 0.272497 | -0.005648 | 0.00297 | 0.056999 |
| rs73344830 | Average total household income before tax | G | A | 0.578942 | -0.012789 | 0.002684 | 1.90E-06 |
| rs7357754 | Average total household income before tax | G | A | 0.500173 | -0.007225 | 0.002652 | 0.0064 |
| rs7359501 | Average total household income before tax | T | C | 0.394521 | 0.006444 | 0.002705 | 0.017 |
| rs736282 | Average total household income before tax | C | T | 0.520837 | -0.003877 | 0.002656 | 0.14 |
| rs7442137 | Average total household income before tax | T | C | 0.634965 | 0.006164 | 0.00275 | 0.025 |
| rs7442885 | Average total household income before tax | G | C | 0.214026 | 0.002865 | 0.00323 | 0.37 |
| rs745249 | Average total household income before tax | T | C | 0.281959 | -0.008886 | 0.002947 | 0.0026 |
| rs7498044 | Average total household income before tax | A | G | 0.217601 | 0.00632 | 0.003238 | 0.051 |
| rs7498665 | Average total household income before tax | G | A | 0.398829 | -0.015428 | 0.002702 | 1.10E-08 |
| rs75035127 | Average total household income before tax | G | A | 0.030535 | 0.015225 | 0.007687 | 0.048 |
| rs7551758 | Average total household income before tax | G | T | 0.533776 | -0.004252 | 0.002652 | 0.11 |
| rs7575189 | Average total household income before tax | A | G | 0.587656 | 0.01345 | 0.002686 | 5.50E-07 |
| rs7594904 | Average total household income before tax | C | T | 0.415408 | 0.005248 | 0.002689 | 0.051 |
| rs76076331 | Average total household income before tax | T | C | 0.123556 | 0.018243 | 0.004038 | 6.30E-06 |
| rs76702514 | Average total household income before tax | G | C | 0.210439 | 0.004307 | 0.003262 | 0.19 |
| rs76878669 | Average total household income before tax | G | C | 0.233938 | -0.005731 | 0.003144 | 0.068 |
| rs76954012 | Average total household income before tax | A | T | 0.093541 | -0.006588 | 0.004542 | 0.15 |
| rs7704530 | Average total household income before tax | A | G | 0.73147 | -0.007819 | 0.00299 | 0.0089 |
| rs7762794 | Average total household income before tax | G | A | 0.285121 | -0.003718 | 0.002927 | 0.2 |
| rs77702622 | Average total household income before tax | A | G | 0.06563 | -0.020215 | 0.005364 | 0.00016 |
| rs7774 | Average total household income before tax | A | C | 0.310025 | 0.001948 | 0.002876 | 0.5 |
| rs77835879 | Average total household income before tax | G | A | 0.100846 | -0.014123 | 0.004408 | 0.0014 |
| rs778371 | Average total household income before tax | G | A | 0.279208 | 0.001127 | 0.002946 | 0.7 |
| rs7785195 | Average total household income before tax | A | G | 0.659672 | 0.001364 | 0.002792 | 0.630001 |
| rs7803932 | Average total household income before tax | A | G | 0.17128 | 0.009542 | 0.003538 | 0.007 |
| rs78086698 | Average total household income before tax | C | T | 0.039627 | -0.001948 | 0.006805 | 0.77 |
| rs7852189 | Average total household income before tax | G | A | 0.315697 | -0.003674 | 0.002841 | 0.2 |
| rs7893571 | Average total household income before tax | T | G | 0.665898 | -0.004369 | 0.00281 | 0.12 |
| rs7909331 | Average total household income before tax | G | A | 0.164294 | -0.003134 | 0.003567 | 0.38 |
| rs7920624 | Average total household income before tax | T | A | 0.536238 | -0.008397 | 0.002662 | 0.0016 |
| rs7921378 | Average total household income before tax | C | G | 0.479803 | -0.00058 | 0.002655 | 0.83 |
| rs7924036 | Average total household income before tax | T | G | 0.50346 | 0.01451 | 0.002644 | 4.10E-08 |
| rs79265434 | Average total household income before tax | G | A | 0.119376 | 0.00211 | 0.004111 | 0.61 |
| rs79269403 | Average total household income before tax | A | G | 0.231311 | 0.003552 | 0.00315 | 0.26 |
| rs7927195 | Average total household income before tax | G | A | 0.614445 | 0.002873 | 0.002717 | 0.29 |
| rs7928622 | Average total household income before tax | T | A | 0.319368 | 0.003567 | 0.002843 | 0.21 |
| rs7938812 | Average total household income before tax | G | T | 0.387685 | -0.00735 | 0.002715 | 0.0068 |
| rs79445414 | Average total household income before tax | C | T | 0.046893 | -0.008809 | 0.006358 | 0.17 |
| rs7944782 | Average total household income before tax | G | T | 0.509959 | -0.004995 | 0.002658 | 0.06 |
| rs7952102 | Average total household income before tax | C | T | 0.38734 | 0.003882 | 0.00271 | 0.15 |
| rs7996639 | Average total household income before tax | A | G | 0.44922 | -0.003052 | 0.002676 | 0.25 |
| rs80153284 | Average total household income before tax | A | C | 0.015618 | 0.002806 | 0.010655 | 0.79 |
| rs8015400 | Average total household income before tax | A | C | 0.677017 | 0.001435 | 0.002829 | 0.61 |
| rs8020034 | Average total household income before tax | A | G | 0.181462 | 0.014682 | 0.003435 | 1.90E-05 |
| rs8112818 | Average total household income before tax | G | A | 0.399891 | 0.007838 | 0.002707 | 0.0038 |
| rs8132491 | Average total household income before tax | A | G | 0.312793 | 0.004237 | 0.002925 | 0.15 |
| rs815163 | Average total household income before tax | C | T | 0.562973 | 0.002246 | 0.002662 | 0.4 |
| rs862320 | Average total household income before tax | T | C | 0.409566 | 0.000375 | 0.002693 | 0.89 |
| rs879620 | Average total household income before tax | T | C | 0.613255 | -0.003292 | 0.002724 | 0.23 |
| rs892612 | Average total household income before tax | C | A | 0.844907 | 0.012452 | 0.003653 | 0.00065 |
| rs9294260 | Average total household income before tax | A | G | 0.476328 | 0.002569 | 0.002662 | 0.33 |
| rs9296389 | Average total household income before tax | C | G | 0.410366 | 0.000212 | 0.002685 | 0.94 |
| rs9320493 | Average total household income before tax | G | A | 0.848769 | -0.00435 | 0.003695 | 0.24 |
| rs9349956 | Average total household income before tax | C | A | 0.18544 | 0.011429 | 0.003405 | 0.00079 |
| rs935166 | Average total household income before tax | A | G | 0.507074 | -0.000273 | 0.002647 | 0.92 |
| rs9366863 | Average total household income before tax | C | T | 0.671808 | 0.004398 | 0.002814 | 0.12 |
| rs9372625 | Average total household income before tax | A | G | 0.382713 | 0.021104 | 0.002728 | 1.00E-14 |
| rs9386319 | Average total household income before tax | G | A | 0.390901 | 0.005704 | 0.002713 | 0.036 |
| rs9478496 | Average total household income before tax | C | T | 0.164259 | -0.004978 | 0.003581 | 0.16 |
| rs9503598 | Average total household income before tax | A | G | 0.44229 | 0.011236 | 0.002685 | 2.90E-05 |
| rs9514600 | Average total household income before tax | G | C | 0.501625 | -0.006987 | 0.002649 | 0.0083 |
| rs9522173 | Average total household income before tax | T | A | 0.394391 | 0.00341 | 0.002703 | 0.21 |
| rs9529119 | Average total household income before tax | G | C | 0.7771 | -0.012051 | 0.003187 | 0.00016 |
| rs9571687 | Average total household income before tax | A | C | 0.329464 | -0.007099 | 0.002817 | 0.012 |
| rs9616906 | Average total household income before tax | A | G | 0.441699 | 0.007381 | 0.002662 | 0.0056 |
| rs9636107 | Average total household income before tax | G | A | 0.470719 | -0.006454 | 0.002656 | 0.015 |
| rs9643087 | Average total household income before tax | T | C | 0.525569 | -0.004295 | 0.002674 | 0.11 |
| rs969512 | Average total household income before tax | T | A | 0.333816 | 0.008261 | 0.002804 | 0.0032 |
| rs9704097 | Average total household income before tax | A | C | 0.506601 | -0.009984 | 0.002645 | 0.00016 |
| rs9852062 | Average total household income before tax | A | T | 0.556954 | 0.00559 | 0.002674 | 0.037 |
| rs9860326 | Average total household income before tax | G | C | 0.328347 | -0.004585 | 0.002818 | 0.1 |
| rs9882532 | Average total household income before tax | C | T | 0.357061 | -0.010971 | 0.002769 | 7.40E-05 |
| rs9888533 | Average total household income before tax | T | C | 0.538266 | -0.001597 | 0.002698 | 0.55 |
| rs9951619 | Average total household income before tax | G | T | 0.767395 | 0.003941 | 0.003151 | 0.21 |
| rs9964724 | Average total household income before tax | T | C | 0.682034 | 0.019663 | 0.002844 | 4.70E-12 |
| rs1000237 | BMI | A | T | 0.355026 | -0.004183 | 0.002085 | 0.045 |
| rs10073890 | BMI | G | A | 0.738373 | 0.007399 | 0.002267 | 0.0011 |
| rs10160769 | BMI | C | G | 0.217493 | -0.015259 | 0.00244 | 4.00E-10 |
| rs1017529 | BMI | A | C | 0.17482 | 0.015237 | 0.002673 | 1.20E-08 |
| rs10423928 | BMI | A | T | 0.194435 | -0.033481 | 0.002517 | 2.20E-40 |
| rs10505836 | BMI | C | A | 0.860001 | 0.017795 | 0.002892 | 7.60E-10 |
| rs10510025 | BMI | T | C | 0.24708 | 0.017795 | 0.002316 | 5.00E-15 |
| rs1064213 | BMI | A | G | 0.478356 | 0.01489 | 0.001987 | 6.60E-14 |
| rs10742752 | BMI | C | T | 0.612246 | 0.011704 | 0.002045 | 1.00E-08 |
| rs10752613 | BMI | A | T | 0.291156 | -0.003141 | 0.002206 | 0.15 |
| rs10765775 | BMI | A | G | 0.391457 | -0.001725 | 0.00205 | 0.4 |
| rs10773002 | BMI | T | A | 0.752096 | 0.00216 | 0.002308 | 0.35 |
| rs10797055 | BMI | G | A | 0.478026 | -0.00617 | 0.001989 | 0.0019 |
| rs10858054 | BMI | T | G | 0.18337 | -0.001753 | 0.002563 | 0.49 |
| rs10861176 | BMI | A | G | 0.733169 | -0.000534 | 0.002254 | 0.81 |
| rs10887578 | BMI | C | G | 0.49753 | 0.013012 | 0.002004 | 8.40E-11 |
| rs10887801 | BMI | T | G | 0.432274 | 0.001357 | 0.002015 | 0.5 |
| rs10922907 | BMI | T | A | 0.548819 | -0.012638 | 0.002001 | 2.70E-10 |
| rs10938398 | BMI | A | G | 0.433498 | 0.029322 | 0.002012 | 4.00E-48 |
| rs10963297 | BMI | G | C | 0.238979 | -0.0035 | 0.002334 | 0.13 |
| rs11000993 | BMI | C | T | 0.123857 | 0.02128 | 0.00302 | 1.80E-12 |
| rs11012732 | BMI | G | A | 0.331714 | 0.021485 | 0.002117 | 3.30E-24 |
| rs1105307 | BMI | A | G | 0.258746 | -0.003627 | 0.002279 | 0.11 |
| rs11079849 | BMI | T | C | 0.328553 | -0.020477 | 0.002127 | 6.30E-22 |
| rs11081529 | BMI | C | T | 0.292954 | -0.000228 | 0.002196 | 0.92 |
| rs11099020 | BMI | T | C | 0.640524 | -0.014355 | 0.002077 | 4.90E-12 |
| rs11134679 | BMI | G | A | 0.684684 | 0.018569 | 0.002149 | 5.50E-18 |
| rs11165643 | BMI | T | C | 0.590138 | 0.019848 | 0.002018 | 7.90E-23 |
| rs11250094 | BMI | C | G | 0.548073 | -0.020777 | 0.002007 | 4.10E-25 |
| rs112633616 | BMI | C | A | 0.033222 | -0.01273 | 0.005607 | 0.023 |
| rs112687095 | BMI | A | G | 0.173036 | 0.000375 | 0.002675 | 0.89 |
| rs113338260 | BMI | C | T | 0.21559 | -0.002888 | 0.002425 | 0.23 |
| rs113520408 | BMI | A | G | 0.282298 | -0.002484 | 0.00222 | 0.26 |
| rs113624107 | BMI | A | G | 0.225895 | 0.014426 | 0.002386 | 1.50E-09 |
| rs1143770 | BMI | T | C | 0.572504 | -0.005924 | 0.002019 | 0.0033 |
| rs115000530 | BMI | T | A | 0.055328 | 0.010797 | 0.004385 | 0.014 |
| rs115454970 | BMI | T | G | 0.255274 | 0.010705 | 0.002316 | 3.80E-06 |
| rs11587347 | BMI | G | C | 0.098141 | -0.007567 | 0.003357 | 0.024 |
| rs11620355 | BMI | A | G | 0.087433 | -0.008252 | 0.003566 | 0.021 |
| rs11635092 | BMI | A | G | 0.360565 | -0.001877 | 0.00209 | 0.37 |
| rs116377258 | BMI | G | A | 0.025659 | 0.067055 | 0.006292 | 1.60E-26 |
| rs11693094 | BMI | T | C | 0.464609 | 0.003501 | 0.001993 | 0.079001 |
| rs11696755 | BMI | C | T | 0.184087 | -0.000325 | 0.002582 | 0.9 |
| rs11709402 | BMI | G | A | 0.278633 | 0.022855 | 0.002225 | 9.50E-25 |
| rs117118217 | BMI | C | G | 0.017706 | 0.044388 | 0.007939 | 2.30E-08 |
| rs11732657 | BMI | A | G | 0.740419 | 0.007194 | 0.002281 | 0.0016 |
| rs118136827 | BMI | T | G | 0.281137 | -0.013524 | 0.002218 | 1.10E-09 |
| rs11866420 | BMI | G | C | 0.574835 | -0.002584 | 0.002019 | 0.2 |
| rs1198588 | BMI | T | A | 0.796903 | -0.011808 | 0.002466 | 1.70E-06 |
| rs12001437 | BMI | C | T | 0.367748 | 0.012357 | 0.002066 | 2.20E-09 |
| rs12033257 | BMI | G | A | 0.382341 | -0.015502 | 0.002059 | 5.10E-14 |
| rs12140153 | BMI | T | G | 0.094285 | -0.033565 | 0.003484 | 5.70E-22 |
| rs12149660 | BMI | A | G | 0.114908 | -0.022198 | 0.00314 | 1.60E-12 |
| rs12156160 | BMI | G | A | 0.150003 | 0.015801 | 0.002798 | 1.60E-08 |
| rs12204714 | BMI | T | C | 0.63301 | -0.002777 | 0.002066 | 0.18 |
| rs12293670 | BMI | G | A | 0.334486 | 0.000439 | 0.002133 | 0.84 |
| rs12303743 | BMI | C | G | 0.097754 | 0.005104 | 0.003358 | 0.13 |
| rs12364470 | BMI | G | T | 0.164517 | 0.01912 | 0.002686 | 1.10E-12 |
| rs12375949 | BMI | C | T | 0.561153 | -0.01055 | 0.002005 | 1.40E-07 |
| rs12375985 | BMI | A | G | 0.354562 | -0.014738 | 0.002084 | 1.50E-12 |
| rs12427047 | BMI | T | C | 0.242759 | -0.017092 | 0.002323 | 1.90E-13 |
| rs12519073 | BMI | T | C | 0.22533 | 0.005 | 0.002388 | 0.036 |
| rs12523398 | BMI | A | T | 0.17451 | -0.005388 | 0.002635 | 0.041 |
| rs12643771 | BMI | T | C | 0.315093 | -0.011402 | 0.002159 | 1.30E-07 |
| rs1266874 | BMI | G | A | 0.349582 | 0.014147 | 0.002087 | 1.20E-11 |
| rs12681792 | BMI | A | C | 0.19265 | 0.014876 | 0.002535 | 4.40E-09 |
| rs12682775 | BMI | C | T | 0.219068 | -0.002946 | 0.002415 | 0.22 |
| rs12712510 | BMI | C | T | 0.536086 | 0.004273 | 0.002002 | 0.033 |
| rs12714592 | BMI | C | A | 0.272899 | 0.003734 | 0.002231 | 0.094001 |
| rs12714702 | BMI | G | A | 0.841319 | 0.016947 | 0.00272 | 4.60E-10 |
| rs12757779 | BMI | A | G | 0.224564 | 0.002537 | 0.002381 | 0.29 |
| rs12762034 | BMI | C | T | 0.076956 | 0.027678 | 0.003737 | 1.30E-13 |
| rs1286058 | BMI | A | T | 0.703745 | 0.014712 | 0.002186 | 1.70E-11 |
| rs12881629 | BMI | G | A | 0.082643 | 0.022201 | 0.003616 | 8.30E-10 |
| rs12907546 | BMI | A | G | 0.211536 | -0.004382 | 0.002449 | 0.074 |
| rs12919291 | BMI | C | G | 0.189322 | -0.002481 | 0.002549 | 0.33 |
| rs12940014 | BMI | C | T | 0.523614 | -1.04E-06 | 0.001994 | 1 |
| rs12955211 | BMI | A | T | 0.328962 | 0.000154 | 0.002137 | 0.94 |
| rs12956148 | BMI | A | C | 0.277653 | 0.013701 | 0.002229 | 7.90E-10 |
| rs1296328 | BMI | C | A | 0.558989 | -0.018145 | 0.002014 | 2.10E-19 |
| rs12977787 | BMI | A | G | 0.54117 | 0.01347 | 0.002002 | 1.70E-11 |
| rs13030994 | BMI | A | G | 0.480542 | 0.00084 | 0.001987 | 0.67 |
| rs13037326 | BMI | T | C | 0.259481 | 0.002119 | 0.002278 | 0.35 |
| rs13090388 | BMI | T | C | 0.306402 | -0.018859 | 0.002157 | 2.20E-18 |
| rs13107325 | BMI | T | C | 0.074863 | 0.047401 | 0.003784 | 5.40E-36 |
| rs13141210 | BMI | T | C | 0.530372 | -0.011612 | 0.001998 | 6.20E-09 |
| rs13145650 | BMI | T | C | 0.914137 | 0.010275 | 0.00356 | 0.0039 |
| rs13175535 | BMI | A | G | 0.295161 | -0.004545 | 0.002216 | 0.04 |
| rs13195636 | BMI | C | A | 0.11515 | 0.013616 | 0.003112 | 1.20E-05 |
| rs1320251 | BMI | T | C | 0.454748 | -0.017656 | 0.002009 | 1.50E-18 |
| rs13218383 | BMI | G | C | 0.335127 | -0.015121 | 0.002108 | 7.40E-13 |
| rs13233308 | BMI | T | C | 0.483087 | -0.004109 | 0.001992 | 0.039 |
| rs13248187 | BMI | C | T | 0.268482 | 0.015984 | 0.002259 | 1.50E-12 |
| rs13261666 | BMI | T | G | 0.506618 | -0.006496 | 0.001993 | 0.0011 |
| rs1327259 | BMI | G | A | 0.38766 | -0.015135 | 0.002049 | 1.50E-13 |
| rs13292699 | BMI | C | A | 0.433729 | -0.021288 | 0.002013 | 4.00E-26 |
| rs1330199 | BMI | T | G | 0.483141 | -0.011896 | 0.002001 | 2.80E-09 |
| rs13307225 | BMI | A | G | 0.893249 | -0.009953 | 0.003241 | 0.0021 |
| rs13317303 | BMI | A | C | 0.150191 | -0.015964 | 0.002786 | 1.00E-08 |
| rs1334297 | BMI | A | G | 0.735618 | -0.013501 | 0.002264 | 2.50E-09 |
| rs13422673 | BMI | T | C | 0.461309 | -0.000501 | 0.001993 | 0.8 |
| rs13427822 | BMI | G | A | 0.271195 | -0.018338 | 0.002258 | 4.70E-16 |
| rs1346841 | BMI | A | G | 0.405023 | -0.012809 | 0.002032 | 2.90E-10 |
| rs1360201 | BMI | T | C | 0.481644 | 0.013159 | 0.001992 | 4.00E-11 |
| rs1363862 | BMI | A | G | 0.277835 | 0.002951 | 0.002233 | 0.19 |
| rs13642 | BMI | T | A | 0.360771 | -0.016479 | 0.002072 | 1.80E-15 |
| rs1391438 | BMI | C | T | 0.687487 | 0.000129 | 0.002146 | 0.95 |
| rs1392816 | BMI | T | C | 0.388148 | -0.012561 | 0.002055 | 9.90E-10 |
| rs140159717 | BMI | T | C | 0.082346 | -0.024688 | 0.003736 | 3.90E-11 |
| rs1438945 | BMI | A | T | 0.715135 | -0.013779 | 0.002214 | 4.90E-10 |
| rs1441264 | BMI | A | G | 0.593699 | 0.018019 | 0.002074 | 3.70E-18 |
| rs1450782 | BMI | G | T | 0.563361 | -0.000566 | 0.002009 | 0.780001 |
| rs1455350 | BMI | A | T | 0.478125 | 0.000597 | 0.001998 | 0.77 |
| rs1458156 | BMI | T | C | 0.488475 | 0.01391 | 0.001994 | 3.10E-12 |
| rs1471740 | BMI | C | T | 0.74036 | 0.019472 | 0.002271 | 9.90E-18 |
| rs147568678 | BMI | C | T | 0.238042 | -0.014208 | 0.002348 | 1.40E-09 |
| rs1477290 | BMI | C | T | 0.136907 | 0.033868 | 0.00292 | 4.20E-31 |
| rs1503526 | BMI | C | T | 0.47996 | 0.015575 | 0.001992 | 5.30E-15 |
| rs152603 | BMI | G | A | 0.365386 | -0.006219 | 0.002065 | 0.0026 |
| rs1565735 | BMI | A | T | 0.203186 | 0.000507 | 0.002495 | 0.84 |
| rs1566085 | BMI | T | G | 0.546278 | -0.013291 | 0.002017 | 4.50E-11 |
| rs1582931 | BMI | A | G | 0.473261 | -0.012853 | 0.00201 | 1.60E-10 |
| rs1584469 | BMI | T | C | 0.293061 | 0.00386 | 0.002186 | 0.077 |
| rs1609010 | BMI | G | A | 0.565695 | 0.021588 | 0.002011 | 7.10E-27 |
| rs1671770 | BMI | C | A | 0.828147 | 0.0055 | 0.002641 | 0.037 |
| rs16846140 | BMI | G | A | 0.337825 | 0.013759 | 0.002103 | 6.10E-11 |
| rs16846463 | BMI | G | A | 0.100491 | 0.001047 | 0.003302 | 0.75 |
| rs16854920 | BMI | C | T | 0.344094 | 0.003612 | 0.002088 | 0.084 |
| rs1689510 | BMI | C | G | 0.338521 | -0.014801 | 0.002106 | 2.10E-12 |
| rs16916303 | BMI | G | A | 0.119778 | -0.019522 | 0.003102 | 3.10E-10 |
| rs16995054 | BMI | T | C | 0.207693 | -0.004482 | 0.002468 | 0.069 |
| rs17193211 | BMI | T | C | 0.066801 | -0.02476 | 0.004066 | 1.10E-09 |
| rs17194490 | BMI | T | G | 0.164631 | -0.001707 | 0.002689 | 0.53 |
| rs17399739 | BMI | G | A | 0.068921 | 0.027708 | 0.003938 | 2.00E-12 |
| rs17565975 | BMI | A | G | 0.563219 | -0.000961 | 0.002018 | 0.630001 |
| rs17598675 | BMI | C | T | 0.482299 | -0.000227 | 0.002007 | 0.91 |
| rs176218 | BMI | T | G | 0.190912 | -0.015026 | 0.002546 | 3.60E-09 |
| rs1778830 | BMI | A | G | 0.362123 | 0.014136 | 0.002071 | 8.80E-12 |
| rs1788808 | BMI | G | A | 0.494885 | -0.020534 | 0.001998 | 8.80E-25 |
| rs1834144 | BMI | A | C | 0.373121 | -0.014136 | 0.002068 | 8.10E-12 |
| rs1860002 | BMI | T | C | 0.536918 | 0.00342 | 0.002009 | 0.089 |
| rs1899896 | BMI | T | C | 0.297181 | 0.008488 | 0.002189 | 0.00011 |
| rs1901512 | BMI | C | T | 0.694462 | 0.002258 | 0.002175 | 0.3 |
| rs1915019 | BMI | G | A | 0.760293 | 0.005073 | 0.002337 | 0.03 |
| rs1919243 | BMI | C | T | 0.48751 | 0.011676 | 0.002017 | 7.10E-09 |
| rs1950829 | BMI | G | A | 0.518524 | 0.00044 | 0.001998 | 0.83 |
| rs1967772 | BMI | A | G | 0.2852 | -0.01686 | 0.00222 | 3.10E-14 |
| rs2035936 | BMI | T | G | 0.055879 | 0.036495 | 0.004391 | 9.40E-17 |
| rs2076603 | BMI | A | G | 0.643878 | -0.012309 | 0.002072 | 2.80E-09 |
| rs2084572 | BMI | G | A | 0.450548 | 0.005877 | 0.001998 | 0.0033 |
| rs2102278 | BMI | G | A | 0.32252 | 0.011834 | 0.002129 | 2.70E-08 |
| rs2133561 | BMI | T | A | 0.611104 | -0.014197 | 0.002063 | 5.90E-12 |
| rs213518 | BMI | C | T | 0.145601 | 0.016276 | 0.002826 | 8.40E-09 |
| rs2153740 | BMI | G | A | 0.479827 | -0.011452 | 0.002008 | 1.20E-08 |
| rs215634 | BMI | G | A | 0.611918 | -0.015494 | 0.002051 | 4.10E-14 |
| rs2172131 | BMI | C | T | 0.578558 | -0.015129 | 0.002019 | 6.70E-14 |
| rs217336 | BMI | A | C | 0.437264 | 0.007065 | 0.002006 | 0.00043 |
| rs2174752 | BMI | T | G | 0.451143 | 0.000431 | 0.002004 | 0.83 |
| rs2176337 | BMI | T | A | 0.315336 | 0.007354 | 0.002145 | 0.00061 |
| rs217672 | BMI | C | A | 0.271724 | 0.017084 | 0.002247 | 2.90E-14 |
| rs2214123 | BMI | G | A | 0.647192 | -0.002441 | 0.002102 | 0.25 |
| rs2234458 | BMI | T | C | 0.639603 | -0.02079 | 0.002071 | 1.10E-23 |
| rs2253310 | BMI | G | C | 0.626162 | 0.017969 | 0.002056 | 2.40E-18 |
| rs2281819 | BMI | A | T | 0.229877 | -0.01568 | 0.002369 | 3.60E-11 |
| rs2283076 | BMI | G | A | 0.229413 | 0.005766 | 0.002377 | 0.015 |
| rs2289379 | BMI | T | C | 0.395696 | -0.015048 | 0.002045 | 1.80E-13 |
| rs2302761 | BMI | T | C | 0.212696 | -0.008789 | 0.002436 | 0.00031 |
| rs2306593 | BMI | T | C | 0.488173 | -0.017015 | 0.001999 | 1.70E-17 |
| rs2307111 | BMI | C | T | 0.395098 | -0.027822 | 0.002037 | 1.80E-42 |
| rs2332700 | BMI | G | C | 0.752002 | 0.004864 | 0.002313 | 0.035 |
| rs2333321 | BMI | G | A | 0.793026 | -0.003207 | 0.002458 | 0.19 |
| rs2342892 | BMI | G | T | 0.516349 | -0.012726 | 0.001992 | 1.70E-10 |
| rs2347526 | BMI | C | T | 0.657748 | -0.008005 | 0.002096 | 0.00013 |
| rs2396625 | BMI | A | T | 0.421333 | -0.019597 | 0.002028 | 4.40E-22 |
| rs2398861 | BMI | G | A | 0.259135 | 0.018204 | 0.002285 | 1.60E-15 |
| rs240963 | BMI | C | T | 0.840798 | -0.012857 | 0.002723 | 2.30E-06 |
| rs242093 | BMI | A | G | 0.570036 | 0.007002 | 0.002031 | 0.00056 |
| rs2433733 | BMI | A | G | 0.677737 | -0.017642 | 0.002125 | 1.00E-16 |
| rs2456020 | BMI | T | C | 0.230208 | -0.000288 | 0.00237 | 0.9 |
| rs2482356 | BMI | C | T | 0.429075 | -0.011159 | 0.00201 | 2.80E-08 |
| rs252761 | BMI | T | G | 0.58811 | -0.011297 | 0.002033 | 2.70E-08 |
| rs2554835 | BMI | A | G | 0.396868 | 0.000518 | 0.002043 | 0.8 |
| rs2568958 | BMI | A | G | 0.603677 | 0.022467 | 0.002027 | 1.50E-28 |
| rs2606228 | BMI | C | A | 0.646353 | -0.013454 | 0.0021 | 1.50E-10 |
| rs2612030 | BMI | C | T | 0.838295 | -0.012412 | 0.002709 | 4.60E-06 |
| rs2616143 | BMI | A | G | 0.319763 | -0.013707 | 0.002142 | 1.60E-10 |
| rs266047 | BMI | A | G | 0.535326 | -0.01054 | 0.001989 | 1.20E-07 |
| rs2678204 | BMI | G | T | 0.340176 | 0.024184 | 0.002097 | 9.00E-31 |
| rs2781668 | BMI | T | C | 0.165574 | 0.014816 | 0.00268 | 3.20E-08 |
| rs2787101 | BMI | T | C | 0.603931 | -0.010734 | 0.00205 | 1.70E-07 |
| rs2819336 | BMI | C | T | 0.638049 | 0.004198 | 0.00207 | 0.043 |
| rs28373063 | BMI | C | G | 0.177799 | -0.000884 | 0.0026 | 0.73 |
| rs28404639 | BMI | T | C | 0.366039 | -0.012053 | 0.00207 | 5.80E-09 |
| rs28489620 | BMI | A | G | 0.290384 | -0.015169 | 0.002217 | 7.70E-12 |
| rs28513670 | BMI | G | A | 0.170626 | -0.009367 | 0.002651 | 0.00041 |
| rs2875762 | BMI | C | G | 0.242983 | 0.015077 | 0.002329 | 9.60E-11 |
| rs2876520 | BMI | G | C | 0.467945 | 0.000781 | 0.002013 | 0.7 |
| rs2920503 | BMI | T | C | 0.285443 | -0.014193 | 0.002213 | 1.40E-10 |
| rs2923431 | BMI | C | G | 0.632467 | -0.003615 | 0.002068 | 0.08 |
| rs293566 | BMI | C | T | 0.331312 | 0.002733 | 0.002125 | 0.2 |
| rs2962334 | BMI | T | G | 0.020066 | 0.043492 | 0.007088 | 8.40E-10 |
| rs2971970 | BMI | G | T | 0.782068 | -0.005884 | 0.002415 | 0.015 |
| rs301800 | BMI | C | T | 0.824081 | 0.00722 | 0.002608 | 0.0056 |
| rs30266 | BMI | A | G | 0.32839 | 0.010765 | 0.002121 | 3.90E-07 |
| rs317656 | BMI | A | T | 0.724546 | -0.01437 | 0.00223 | 1.20E-10 |
| rs320693 | BMI | C | G | 0.453463 | -0.007335 | 0.001998 | 0.00024 |
| rs3211995 | BMI | A | G | 0.160296 | -0.015376 | 0.002728 | 1.70E-08 |
| rs329118 | BMI | T | C | 0.419403 | -0.016759 | 0.002019 | 1.00E-16 |
| rs34025316 | BMI | T | C | 0.337633 | 0.011788 | 0.002118 | 2.60E-08 |
| rs34045288 | BMI | T | C | 0.334424 | 0.02353 | 0.00211 | 6.90E-29 |
| rs34234296 | BMI | A | G | 0.392475 | -0.015008 | 0.002055 | 2.80E-13 |
| rs34481751 | BMI | A | C | 0.165287 | -0.019136 | 0.002724 | 2.20E-12 |
| rs34517439 | BMI | A | C | 0.121683 | 0.038287 | 0.003074 | 1.30E-35 |
| rs347551 | BMI | G | C | 0.472309 | 0.013837 | 0.002026 | 8.40E-12 |
| rs34811474 | BMI | A | G | 0.230829 | -0.028337 | 0.00236 | 3.30E-33 |
| rs35154326 | BMI | G | A | 0.274043 | -0.013375 | 0.002251 | 2.80E-09 |
| rs35309068 | BMI | G | T | 0.433196 | -0.007918 | 0.002008 | 8.10E-05 |
| rs354155 | BMI | C | G | 0.089076 | 0.011052 | 0.003486 | 0.0015 |
| rs35417702 | BMI | T | C | 0.525093 | 0.003206 | 0.001995 | 0.11 |
| rs35475880 | BMI | T | G | 0.206989 | -3.94E-05 | 0.002463 | 0.99 |
| rs35532491 | BMI | T | A | 0.103097 | -0.002662 | 0.003285 | 0.42 |
| rs355777 | BMI | C | G | 0.407549 | 0.015506 | 0.002028 | 2.10E-14 |
| rs35867081 | BMI | G | A | 0.512198 | -0.01473 | 0.002005 | 2.00E-13 |
| rs36007635 | BMI | A | G | 0.137722 | -0.020519 | 0.002889 | 1.20E-12 |
| rs36061954 | BMI | T | C | 0.398777 | 0.012875 | 0.002034 | 2.50E-10 |
| rs36119825 | BMI | A | G | 0.442225 | -0.006463 | 0.002001 | 0.0012 |
| rs363096 | BMI | C | T | 0.575227 | -0.007445 | 0.002014 | 0.00022 |
| rs3747631 | BMI | C | G | 0.209375 | -0.003428 | 0.002438 | 0.16 |
| rs3764625 | BMI | G | T | 0.587524 | -0.011498 | 0.00203 | 1.50E-08 |
| rs3770754 | BMI | G | C | 0.359267 | 0.001429 | 0.00207 | 0.49 |
| rs3784710 | BMI | C | T | 0.226642 | -0.029512 | 0.002378 | 2.30E-35 |
| rs3800546 | BMI | G | C | 0.25598 | -0.001366 | 0.002283 | 0.55 |
| rs3807865 | BMI | A | G | 0.412243 | 0.007826 | 0.00202 | 0.00011 |
| rs3809634 | BMI | G | A | 0.31361 | 0.003682 | 0.002166 | 0.089 |
| rs3814883 | BMI | T | C | 0.482235 | 0.024051 | 0.001999 | 2.50E-33 |
| rs3845344 | BMI | T | C | 0.391167 | 0.01697 | 0.002035 | 7.40E-17 |
| rs3851998 | BMI | G | C | 0.74307 | -0.013897 | 0.002286 | 1.20E-09 |
| rs3896224 | BMI | G | A | 0.414721 | -0.009574 | 0.002039 | 2.60E-06 |
| rs3897821 | BMI | G | A | 0.333934 | 0.006771 | 0.002104 | 0.0013 |
| rs3901286 | BMI | A | C | 0.152427 | -0.022624 | 0.002777 | 3.70E-16 |
| rs394608 | BMI | C | T | 0.537722 | 0.01858 | 0.00201 | 2.30E-20 |
| rs40071 | BMI | C | T | 0.179493 | -0.026046 | 0.002601 | 1.30E-23 |
| rs4044321 | BMI | G | A | 0.641105 | -0.005046 | 0.002079 | 0.015 |
| rs4055791 | BMI | T | C | 0.416769 | -0.018063 | 0.002023 | 4.40E-19 |
| rs406388 | BMI | G | C | 0.177204 | 0.015881 | 0.002621 | 1.40E-09 |
| rs4148155 | BMI | G | A | 0.113281 | -0.022424 | 0.00313 | 7.80E-13 |
| rs4261944 | BMI | G | T | 0.364835 | 0.014174 | 0.002072 | 7.90E-12 |
| rs4267103 | BMI | C | T | 0.186356 | 0.015907 | 0.002562 | 5.40E-10 |
| rs429343 | BMI | G | A | 0.576563 | -0.017294 | 0.002012 | 8.40E-18 |
| rs429358 | BMI | C | T | 0.154048 | -0.026632 | 0.002765 | 5.90E-22 |
| rs4328757 | BMI | T | C | 0.612934 | -0.006959 | 0.002042 | 0.00065 |
| rs4352658 | BMI | T | C | 0.080846 | 0.00337 | 0.003651 | 0.36 |
| rs4382592 | BMI | G | T | 0.699005 | -0.008943 | 0.002174 | 3.90E-05 |
| rs4419475 | BMI | T | A | 0.407332 | 0.011853 | 0.002026 | 4.90E-09 |
| rs4439537 | BMI | C | T | 0.524826 | -0.001462 | 0.002 | 0.46 |
| rs4477562 | BMI | T | C | 0.128603 | 0.029763 | 0.003003 | 3.70E-23 |
| rs4482463 | BMI | A | C | 0.923052 | -0.030876 | 0.003736 | 1.40E-16 |
| rs4613074 | BMI | C | T | 0.185103 | -0.023585 | 0.002565 | 3.70E-20 |
| rs4648450 | BMI | A | C | 0.466827 | -0.014831 | 0.002003 | 1.30E-13 |
| rs4653164 | BMI | T | C | 0.690546 | 0.005943 | 0.002147 | 0.0056 |
| rs4671328 | BMI | G | T | 0.551247 | -0.020982 | 0.002012 | 1.80E-25 |
| rs4700393 | BMI | G | A | 0.519304 | 0.002326 | 0.001994 | 0.24 |
| rs4702 | BMI | A | G | 0.556608 | -0.005355 | 0.002008 | 0.0076 |
| rs4709807 | BMI | C | T | 0.759056 | 0.005054 | 0.002333 | 0.03 |
| rs4737188 | BMI | T | A | 0.473833 | -0.01301 | 0.001998 | 7.40E-11 |
| rs4757144 | BMI | A | G | 0.590102 | 0.016157 | 0.002026 | 1.50E-15 |
| rs4757957 | BMI | C | G | 0.679998 | -0.004698 | 0.00214 | 0.028 |
| rs4764949 | BMI | G | A | 0.325788 | -0.01797 | 0.002128 | 3.10E-17 |
| rs4790292 | BMI | A | C | 0.153741 | -0.026025 | 0.002776 | 6.90E-21 |
| rs4810227 | BMI | A | G | 0.61901 | 0.004392 | 0.002069 | 0.034 |
| rs4812325 | BMI | A | G | 0.609386 | -0.004227 | 0.00206 | 0.04 |
| rs4832298 | BMI | T | C | 0.686123 | -0.015969 | 0.002139 | 8.20E-14 |
| rs4846724 | BMI | A | G | 0.533148 | -0.008612 | 0.001998 | 1.60E-05 |
| rs4876611 | BMI | G | A | 0.720193 | 0.019307 | 0.002221 | 3.50E-18 |
| rs4895799 | BMI | T | C | 0.584972 | 0.011573 | 0.002026 | 1.10E-08 |
| rs4958702 | BMI | C | T | 0.572319 | -0.015568 | 0.00201 | 9.60E-15 |
| rs4961705 | BMI | C | G | 0.348357 | -0.003037 | 0.002126 | 0.15 |
| rs512121 | BMI | C | T | 0.19205 | -0.015682 | 0.002541 | 6.70E-10 |
| rs539515 | BMI | C | A | 0.204979 | 0.049741 | 0.00246 | 7.00E-91 |
| rs55658481 | BMI | A | G | 0.339221 | 0.013358 | 0.002096 | 1.80E-10 |
| rs55707359 | BMI | G | T | 0.015448 | 0.052837 | 0.00818 | 1.10E-10 |
| rs55714539 | BMI | C | A | 0.343621 | 0.017839 | 0.002116 | 3.50E-17 |
| rs55726687 | BMI | A | G | 0.209748 | 0.024856 | 0.002444 | 2.70E-24 |
| rs55736314 | BMI | G | C | 0.403647 | -0.005857 | 0.002032 | 0.004 |
| rs558887 | BMI | G | A | 0.307505 | -0.013302 | 0.002165 | 8.00E-10 |
| rs56094641 | BMI | G | A | 0.404637 | 0.073344 | 0.002029 | ######## |
| rs56133507 | BMI | G | T | 0.196867 | 0.014155 | 0.002493 | 1.40E-08 |
| rs56161855 | BMI | T | A | 0.132865 | 0.022726 | 0.002939 | 1.10E-14 |
| rs56203622 | BMI | C | T | 0.145543 | 0.017954 | 0.002823 | 2.00E-10 |
| rs56335113 | BMI | G | A | 0.691115 | 0.004291 | 0.002161 | 0.047 |
| rs56356382 | BMI | C | T | 0.192343 | -0.022567 | 0.002541 | 6.70E-19 |
| rs56391344 | BMI | A | G | 0.254146 | -0.01586 | 0.002296 | 4.90E-12 |
| rs56399737 | BMI | T | C | 0.44917 | -0.015827 | 0.002011 | 3.60E-15 |
| rs56858768 | BMI | A | G | 0.296903 | 0.015792 | 0.00219 | 5.60E-13 |
| rs57636386 | BMI | C | T | 0.083839 | -0.040867 | 0.003609 | 1.00E-29 |
| rs58120505 | BMI | C | T | 0.433932 | 0.001963 | 0.002008 | 0.33 |
| rs59086897 | BMI | A | T | 0.487574 | 0.033495 | 0.001985 | 6.60E-64 |
| rs59237168 | BMI | C | T | 0.215628 | -0.015771 | 0.002427 | 8.20E-11 |
| rs5995843 | BMI | G | A | 0.346053 | -0.017617 | 0.002101 | 5.00E-17 |
| rs60764613 | BMI | T | G | 0.14488 | 0.020132 | 0.002848 | 1.60E-12 |
| rs6123924 | BMI | G | A | 0.153877 | 0.010624 | 0.002773 | 0.00013 |
| rs6134916 | BMI | T | C | 0.49267 | -0.010937 | 0.002001 | 4.60E-08 |
| rs61813324 | BMI | T | C | 0.135701 | 0.029013 | 0.002942 | 6.20E-23 |
| rs61828088 | BMI | A | G | 0.110449 | 0.022126 | 0.003169 | 2.90E-12 |
| rs61903695 | BMI | G | A | 0.254893 | 0.016729 | 0.002289 | 2.70E-13 |
| rs61914045 | BMI | A | G | 0.202494 | 0.002012 | 0.00249 | 0.42 |
| rs62097985 | BMI | T | C | 0.418362 | 0.004124 | 0.002029 | 0.042 |
| rs62107261 | BMI | C | T | 0.048316 | -0.091532 | 0.004644 | 1.70E-86 |
| rs62134195 | BMI | T | C | 0.040979 | -0.000568 | 0.005073 | 0.91 |
| rs62176243 | BMI | T | A | 0.245042 | -0.015076 | 0.002306 | 6.20E-11 |
| rs62176993 | BMI | A | G | 0.401151 | 0.011228 | 0.002033 | 3.40E-08 |
| rs62190049 | BMI | C | G | 0.390421 | -0.011291 | 0.002051 | 3.70E-08 |
| rs62439690 | BMI | A | G | 0.262453 | 0.005006 | 0.002289 | 0.029 |
| rs6265 | BMI | T | C | 0.188495 | -0.040116 | 0.002546 | 5.90E-56 |
| rs6531639 | BMI | A | G | 0.247516 | -0.013986 | 0.002357 | 3.00E-09 |
| rs6545714 | BMI | A | G | 0.601485 | -0.020673 | 0.002031 | 2.50E-24 |
| rs6546857 | BMI | G | A | 0.238094 | 0.002064 | 0.002336 | 0.38 |
| rs6560906 | BMI | C | T | 0.691869 | -0.012159 | 0.002157 | 1.70E-08 |
| rs6567160 | BMI | C | T | 0.23266 | 0.053626 | 0.00236 | ######## |
| rs6575340 | BMI | A | G | 0.636028 | 0.020879 | 0.002077 | 9.10E-24 |
| rs66511648 | BMI | C | T | 0.284701 | 0.009217 | 0.002213 | 3.10E-05 |
| rs66679256 | BMI | T | C | 0.445806 | 0.01533 | 0.002003 | 1.90E-14 |
| rs6669341 | BMI | G | A | 0.58271 | -0.017286 | 0.002014 | 9.20E-18 |
| rs6682438 | BMI | C | T | 0.673173 | 0.012724 | 0.002116 | 1.80E-09 |
| rs6688826 | BMI | C | T | 0.29819 | 0.014105 | 0.002168 | 7.70E-11 |
| rs6707827 | BMI | G | A | 0.70356 | 0.012336 | 0.002189 | 1.70E-08 |
| rs6710091 | BMI | G | C | 0.348107 | -0.011573 | 0.002082 | 2.70E-08 |
| rs6719762 | BMI | C | T | 0.472932 | 0.006151 | 0.001996 | 0.0021 |
| rs6731967 | BMI | C | G | 0.235646 | 0.003722 | 0.002347 | 0.11 |
| rs6744646 | BMI | G | A | 0.828314 | 0.055431 | 0.002632 | 1.70E-98 |
| rs6744794 | BMI | G | C | 0.622846 | 0.010719 | 0.002047 | 1.60E-07 |
| rs6752979 | BMI | A | G | 0.316792 | 0.012176 | 0.002133 | 1.10E-08 |
| rs6774894 | BMI | A | T | 0.358077 | 0.01294 | 0.002072 | 4.30E-10 |
| rs6798742 | BMI | G | A | 0.298082 | -0.005928 | 0.002175 | 0.0064 |
| rs6803651 | BMI | T | G | 0.434807 | -0.001776 | 0.002015 | 0.38 |
| rs6805241 | BMI | C | T | 0.231019 | 0.007473 | 0.002363 | 0.0016 |
| rs6843852 | BMI | T | C | 0.507745 | 0.013401 | 0.001991 | 1.70E-11 |
| rs6943762 | BMI | C | T | 0.126923 | 0.005661 | 0.003005 | 0.06 |
| rs6959891 | BMI | G | A | 0.281791 | 0.004822 | 0.002224 | 0.03 |
| rs6962980 | BMI | C | A | 0.556058 | -0.015966 | 0.002003 | 1.60E-15 |
| rs6974218 | BMI | C | A | 0.383555 | -0.000105 | 0.002051 | 0.96 |
| rs698147 | BMI | G | A | 0.543488 | -0.013299 | 0.002 | 2.90E-11 |
| rs7008955 | BMI | G | T | 0.526482 | -0.005494 | 0.002005 | 0.0062 |
| rs7012546 | BMI | T | C | 0.413903 | -0.007531 | 0.002022 | 2.00E-04 |
| rs7029718 | BMI | A | G | 0.415439 | 0.010398 | 0.002023 | 2.80E-07 |
| rs7031698 | BMI | C | T | 0.775798 | -0.003933 | 0.00239 | 0.1 |
| rs7034554 | BMI | G | A | 0.373722 | -0.013538 | 0.002057 | 4.70E-11 |
| rs7038943 | BMI | C | T | 0.338794 | -0.014191 | 0.002102 | 1.50E-11 |
| rs7079070 | BMI | A | G | 0.454605 | 0.005651 | 0.002 | 0.0047 |
| rs708228 | BMI | T | C | 0.327986 | 0.006235 | 0.002121 | 0.0033 |
| rs7124681 | BMI | A | C | 0.408403 | 0.025898 | 0.002021 | 1.40E-37 |
| rs7132908 | BMI | A | G | 0.384452 | 0.029401 | 0.002049 | 1.10E-46 |
| rs71646142 | BMI | T | C | 0.191865 | -0.00632 | 0.002539 | 0.013 |
| rs7201895 | BMI | A | G | 0.354227 | -0.015007 | 0.002096 | 8.00E-13 |
| rs7206608 | BMI | G | C | 0.321564 | 0.013485 | 0.002134 | 2.60E-10 |
| rs7218014 | BMI | C | T | 0.197299 | 0.018762 | 0.002511 | 7.80E-14 |
| rs7233920 | BMI | A | G | 0.226818 | -0.001154 | 0.002397 | 0.630001 |
| rs7236339 | BMI | A | G | 0.227595 | 0.007161 | 0.002393 | 0.0028 |
| rs723672 | BMI | T | C | 0.431522 | 0.011108 | 0.002022 | 4.00E-08 |
| rs7250833 | BMI | T | C | 0.288875 | 0.013587 | 0.002205 | 7.10E-10 |
| rs7259070 | BMI | C | T | 0.596195 | 0.021704 | 0.002052 | 3.80E-26 |
| rs72673947 | BMI | G | A | 0.10707 | 0.022163 | 0.003239 | 7.70E-12 |
| rs7278859 | BMI | T | A | 0.308696 | -0.000822 | 0.002175 | 0.709999 |
| rs72887338 | BMI | C | T | 0.386154 | 0.003654 | 0.002047 | 0.074 |
| rs72892910 | BMI | T | G | 0.1722 | 0.038803 | 0.00264 | 6.60E-49 |
| rs72910629 | BMI | G | A | 0.136055 | 0.01626 | 0.002919 | 2.50E-08 |
| rs72986630 | BMI | T | C | 0.06448 | 0.003379 | 0.004214 | 0.42 |
| rs73026725 | BMI | A | C | 0.153577 | -0.022254 | 0.002771 | 9.70E-16 |
| rs730384 | BMI | A | G | 0.440565 | -0.002532 | 0.002021 | 0.21 |
| rs73052033 | BMI | C | T | 0.185068 | -0.030049 | 0.002565 | 1.00E-31 |
| rs73142879 | BMI | T | C | 0.192242 | -0.027215 | 0.002541 | 9.30E-27 |
| rs73213484 | BMI | T | A | 0.141232 | -0.022939 | 0.002858 | 1.00E-15 |
| rs7331420 | BMI | A | G | 0.285202 | -0.014173 | 0.002217 | 1.60E-10 |
| rs7332724 | BMI | T | C | 0.272586 | -0.000872 | 0.00224 | 0.7 |
| rs73344830 | BMI | G | A | 0.579077 | 0.001161 | 0.002023 | 0.57 |
| rs7357754 | BMI | G | A | 0.50012 | 0.014398 | 0.001998 | 5.80E-13 |
| rs7359501 | BMI | T | C | 0.394265 | -0.004636 | 0.00204 | 0.023 |
| rs736282 | BMI | C | T | 0.52103 | 0.004964 | 0.002001 | 0.013 |
| rs7442137 | BMI | T | C | 0.634233 | -0.012479 | 0.002067 | 1.60E-09 |
| rs7442885 | BMI | G | C | 0.214127 | -0.022855 | 0.00243 | 5.20E-21 |
| rs745249 | BMI | T | C | 0.281897 | 0.018069 | 0.002214 | 3.30E-16 |
| rs7498044 | BMI | A | G | 0.217396 | -0.01689 | 0.002443 | 4.70E-12 |
| rs7498665 | BMI | G | A | 0.399549 | 0.026502 | 0.002035 | 9.30E-39 |
| rs75035127 | BMI | G | A | 0.030385 | -0.041123 | 0.005791 | 1.20E-12 |
| rs7551758 | BMI | G | T | 0.533634 | -0.007296 | 0.001992 | 0.00025 |
| rs7575189 | BMI | A | G | 0.588214 | -0.006079 | 0.002018 | 0.0026 |
| rs7594904 | BMI | C | T | 0.415324 | 0.000361 | 0.002019 | 0.86 |
| rs76076331 | BMI | T | C | 0.123452 | -0.008166 | 0.003036 | 0.0072 |
| rs76702514 | BMI | G | C | 0.210646 | -0.016656 | 0.002451 | 1.10E-11 |
| rs76878669 | BMI | G | C | 0.234298 | -0.001296 | 0.002365 | 0.58 |
| rs76954012 | BMI | A | T | 0.093539 | 0.010316 | 0.003418 | 0.0025 |
| rs7704530 | BMI | A | G | 0.731943 | 4.02E-05 | 0.002252 | 0.99 |
| rs7762794 | BMI | G | A | 0.285511 | 0.014765 | 0.002202 | 2.00E-11 |
| rs77702622 | BMI | A | G | 0.065708 | 0.006952 | 0.004041 | 0.085 |
| rs7774 | BMI | A | C | 0.310472 | 0.015341 | 0.002168 | 1.50E-12 |
| rs77835879 | BMI | G | A | 0.100887 | 0.007927 | 0.003322 | 0.017 |
| rs778371 | BMI | G | A | 0.27925 | 0.001677 | 0.002212 | 0.450001 |
| rs7785195 | BMI | A | G | 0.659512 | 0.002051 | 0.002102 | 0.33 |
| rs7803932 | BMI | A | G | 0.171047 | -0.000816 | 0.002667 | 0.760001 |
| rs78086698 | BMI | C | T | 0.03983 | 0.032418 | 0.005115 | 2.30E-10 |
| rs7852189 | BMI | G | A | 0.315853 | 0.017152 | 0.00214 | 1.10E-15 |
| rs7893571 | BMI | T | G | 0.665931 | 0.01443 | 0.002117 | 9.40E-12 |
| rs7909331 | BMI | G | A | 0.16449 | 0.003018 | 0.002686 | 0.26 |
| rs7920624 | BMI | T | A | 0.536814 | 0.005964 | 0.002006 | 0.0029 |
| rs7921378 | BMI | C | G | 0.48004 | 0.003289 | 0.002 | 0.1 |
| rs7924036 | BMI | T | G | 0.503279 | -0.014489 | 0.001992 | 3.50E-13 |
| rs79265434 | BMI | G | A | 0.119318 | -0.009136 | 0.003096 | 0.0032 |
| rs79269403 | BMI | A | G | 0.231017 | -0.013429 | 0.00237 | 1.50E-08 |
| rs7927195 | BMI | G | A | 0.614671 | -0.000338 | 0.002046 | 0.87 |
| rs7928622 | BMI | T | A | 0.319429 | -0.000287 | 0.002141 | 0.89 |
| rs7938812 | BMI | G | T | 0.387739 | -0.006476 | 0.002044 | 0.0015 |
| rs79445414 | BMI | C | T | 0.046782 | 0.001236 | 0.004796 | 0.8 |
| rs7944782 | BMI | G | T | 0.509785 | 0.015982 | 0.002002 | 1.40E-15 |
| rs7952102 | BMI | C | T | 0.387816 | -0.01426 | 0.002041 | 2.80E-12 |
| rs7996639 | BMI | A | G | 0.44923 | 0.014345 | 0.002018 | 1.20E-12 |
| rs80153284 | BMI | A | C | 0.01565 | 0.012833 | 0.00799 | 0.11 |
| rs8015400 | BMI | A | C | 0.677163 | 0.021427 | 0.002133 | 9.60E-24 |
| rs8020034 | BMI | A | G | 0.181402 | -0.006406 | 0.002591 | 0.013 |
| rs8112818 | BMI | G | A | 0.400383 | -0.020334 | 0.002042 | 2.30E-23 |
| rs8132491 | BMI | A | G | 0.312982 | -0.015532 | 0.00221 | 2.10E-12 |
| rs815163 | BMI | C | T | 0.563215 | -0.016799 | 0.002001 | 4.60E-17 |
| rs862320 | BMI | T | C | 0.409791 | -0.023236 | 0.002028 | 2.20E-30 |
| rs879620 | BMI | T | C | 0.613274 | 0.02414 | 0.002051 | 5.70E-32 |
| rs892612 | BMI | C | A | 0.844821 | -0.012138 | 0.00275 | 1.00E-05 |
| rs9294260 | BMI | A | G | 0.476582 | 0.014714 | 0.002003 | 2.00E-13 |
| rs9296389 | BMI | C | G | 0.410625 | 0.011535 | 0.00202 | 1.10E-08 |
| rs9320493 | BMI | G | A | 0.848965 | -0.001983 | 0.002784 | 0.48 |
| rs9349956 | BMI | C | A | 0.18514 | 0.000789 | 0.002564 | 0.760001 |
| rs935166 | BMI | A | G | 0.506861 | -0.016083 | 0.001988 | 5.90E-16 |
| rs9366863 | BMI | C | T | 0.671952 | -0.028134 | 0.002117 | 2.60E-40 |
| rs9372625 | BMI | A | G | 0.381731 | -0.016837 | 0.002054 | 2.50E-16 |
| rs9386319 | BMI | G | A | 0.390721 | 0.000189 | 0.002043 | 0.93 |
| rs9478496 | BMI | C | T | 0.164208 | 0.017597 | 0.002695 | 6.60E-11 |
| rs9503598 | BMI | A | G | 0.441782 | -0.006367 | 0.002021 | 0.0016 |
| rs9514600 | BMI | G | C | 0.502123 | -0.004702 | 0.001996 | 0.018 |
| rs9522173 | BMI | T | A | 0.394176 | -0.012371 | 0.002038 | 1.30E-09 |
| rs9529119 | BMI | G | C | 0.777026 | 0.002212 | 0.002404 | 0.36 |
| rs9571687 | BMI | A | C | 0.329438 | -0.013248 | 0.002125 | 4.60E-10 |
| rs9616906 | BMI | A | G | 0.440993 | 0.002474 | 0.002011 | 0.22 |
| rs9636107 | BMI | G | A | 0.470978 | 0.002896 | 0.002003 | 0.15 |
| rs9643087 | BMI | T | C | 0.525568 | 0.0067 | 0.002016 | 0.00089 |
| rs969512 | BMI | T | A | 0.333627 | -0.006031 | 0.002111 | 0.0043 |
| rs9704097 | BMI | A | C | 0.506419 | 0.003773 | 0.001992 | 0.058 |
| rs9852062 | BMI | A | T | 0.556834 | -0.013965 | 0.002009 | 3.60E-12 |
| rs9860326 | BMI | G | C | 0.328353 | 0.01451 | 0.002119 | 7.50E-12 |
| rs9882532 | BMI | C | T | 0.357115 | 0.003255 | 0.002083 | 0.12 |
| rs9888533 | BMI | T | C | 0.538118 | 0.012162 | 0.002034 | 2.20E-09 |
| rs9951619 | BMI | G | T | 0.767282 | 0.014976 | 0.002375 | 2.90E-10 |
| rs9964724 | BMI | T | C | 0.681778 | -0.000568 | 0.002144 | 0.79 |
| rs1000237 | MD | A | T | 0.3556 | -0.0011 | 0.0045 | 0.8047 |
| rs10073890 | MD | G | A | 0.7409 | -0.0072 | 0.0049 | 0.1427 |
| rs10160769 | MD | C | G | 0.219 | -0.0104 | 0.0053 | 0.0473 |
| rs1017529 | MD | A | C | 0.1743 | -0.0047 | 0.0058 | 0.4222 |
| rs10423928 | MD | A | T | 0.1998 | 0.004 | 0.0054 | 0.4561 |
| rs10505836 | MD | C | A | 0.8592 | 0.0049 | 0.0062 | 0.4263 |
| rs10510025 | MD | T | C | 0.2475 | 0.0045 | 0.005 | 0.3662 |
| rs1064213 | MD | A | G | 0.4805 | -0.0042 | 0.0043 | 0.3346 |
| rs10742752 | MD | C | T | 0.6134 | -0.0017 | 0.0044 | 0.6989 |
| rs10752613 | MD | A | T | 0.2914 | 0.0038 | 0.0048 | 0.4249 |
| rs10765775 | MD | A | G | 0.3929 | -0.0024 | 0.0044 | 0.585901 |
| rs10773002 | MD | T | A | 0.7507 | 0.0143 | 0.005 | 0.004002 |
| rs10797055 | MD | G | A | 0.4786 | -0.001 | 0.0044 | 0.8173 |
| rs10858054 | MD | T | G | 0.1824 | -0.001 | 0.0056 | 0.8624 |
| rs10861176 | MD | A | G | 0.7346 | -0.0101 | 0.0049 | 0.03949 |
| rs10887578 | MD | C | G | 0.4987 | 0.0048 | 0.0048 | 0.3112 |
| rs10887801 | MD | T | G | 0.4317 | -2.00E-04 | 0.0043 | 0.9652 |
| rs10922907 | MD | T | A | 0.549 | -0.0104 | 0.0045 | 0.01955 |
| rs10938398 | MD | A | G | 0.4303 | 0.0061 | 0.0043 | 0.1612 |
| rs10963297 | MD | G | C | 0.2393 | 5.00E-04 | 0.005 | 0.9137 |
| rs11000993 | MD | C | T | 0.1231 | -0.0039 | 0.0065 | 0.5531 |
| rs11012732 | MD | G | A | 0.332 | 0.0049 | 0.0046 | 0.283 |
| rs1105307 | MD | A | G | 0.2595 | 0.0073 | 0.0049 | 0.1382 |
| rs11079849 | MD | T | C | 0.3268 | 0.0034 | 0.0046 | 0.4574 |
| rs11081529 | MD | C | T | 0.2911 | 0.0019 | 0.0047 | 0.6867 |
| rs11099020 | MD | T | C | 0.6417 | -0.0052 | 0.0045 | 0.2436 |
| rs11134679 | MD | G | A | 0.6837 | 1.00E-04 | 0.0046 | 0.9856 |
| rs11165643 | MD | T | C | 0.5894 | -0.0035 | 0.0045 | 0.431 |
| rs11250094 | MD | C | G | 0.5479 | 0.0087 | 0.0043 | 0.04395 |
| rs112633616 | MD | C | A | 0.0331 | -0.01 | 0.0123 | 0.4144 |
| rs112687095 | MD | A | G | 0.1699 | 0.0034 | 0.0061 | 0.5846 |
| rs113338260 | MD | C | T | 0.2139 | -0.001 | 0.0053 | 0.8479 |
| rs113520408 | MD | A | G | 0.2807 | 0.0018 | 0.0048 | 0.706801 |
| rs113624107 | MD | A | G | 0.2263 | 0.0022 | 0.0051 | 0.674899 |
| rs1143770 | MD | T | C | 0.5749 | -0.01 | 0.0044 | 0.02215 |
| rs115000530 | MD | T | A | 0.0553 | 0.0101 | 0.0095 | 0.2918 |
| rs115454970 | MD | T | G | 0.2572 | 0.0071 | 0.005 | 0.1614 |
| rs11587347 | MD | G | C | 0.0969 | 0.0227 | 0.0073 | 0.001924 |
| rs11620355 | MD | A | G | 0.0889 | -0.0216 | 0.0077 | 0.004746 |
| rs11635092 | MD | A | G | 0.3618 | -0.0102 | 0.0045 | 0.02496 |
| rs116377258 | MD | G | A | 0.0264 | 0.0341 | 0.0133 | 0.0104 |
| rs11693094 | MD | T | C | 0.4651 | -0.0127 | 0.0043 | 0.003292 |
| rs11696755 | MD | C | T | 0.1846 | 0.0072 | 0.0056 | 0.1969 |
| rs11709402 | MD | G | A | 0.2774 | 0.0071 | 0.0048 | 0.1399 |
| rs117118217 | MD | C | G | 0.0172 | -0.0068 | 0.0177 | 0.7001 |
| rs11732657 | MD | A | G | 0.7425 | 0.0051 | 0.0049 | 0.3039 |
| rs118136827 | MD | T | G | 0.2799 | 0.005 | 0.0048 | 0.2968 |
| rs11866420 | MD | G | C | 0.5816 | 8.00E-04 | 0.0044 | 0.8483 |
| rs1198588 | MD | T | A | 0.796 | -0.01 | 0.0055 | 0.06841 |
| rs12001437 | MD | C | T | 0.3692 | -0.009 | 0.0045 | 0.04235 |
| rs12033257 | MD | G | A | 0.3826 | -0.0052 | 0.0045 | 0.2481 |
| rs12140153 | MD | T | G | 0.094 | -3.00E-04 | 0.0076 | 0.9736 |
| rs12149660 | MD | A | G | 0.1164 | -0.0114 | 0.0069 | 0.097719 |
| rs12156160 | MD | G | A | 0.1487 | 0.0011 | 0.0062 | 0.8584 |
| rs12204714 | MD | T | C | 0.6383 | -0.0179 | 0.0045 | 5.96E-05 |
| rs12293670 | MD | G | A | 0.3356 | -0.0085 | 0.0046 | 0.06311 |
| rs12303743 | MD | C | G | 0.0979 | -0.008 | 0.0073 | 0.27 |
| rs12364470 | MD | G | T | 0.1654 | -0.0113 | 0.0058 | 0.0539 |
| rs12375949 | MD | C | T | 0.5642 | -0.0053 | 0.0043 | 0.2224 |
| rs12375985 | MD | A | G | 0.355 | -0.0193 | 0.0045 | 1.77E-05 |
| rs12427047 | MD | T | C | 0.2458 | 0.0032 | 0.005 | 0.5157 |
| rs12519073 | MD | T | C | 0.2265 | -0.0111 | 0.0052 | 0.03207 |
| rs12523398 | MD | A | T | 0.1724 | -0.016 | 0.0057 | 0.005224 |
| rs12643771 | MD | T | C | 0.3126 | -0.0023 | 0.0047 | 0.6282 |
| rs1266874 | MD | G | A | 0.3488 | 2.00E-04 | 0.0045 | 0.9646 |
| rs12681792 | MD | A | C | 0.1928 | -0.0076 | 0.0055 | 0.1667 |
| rs12682775 | MD | C | T | 0.22 | 0.0132 | 0.0052 | 0.01106 |
| rs12712510 | MD | C | T | 0.5372 | -0.0139 | 0.0043 | 0.001361 |
| rs12714592 | MD | C | A | 0.2728 | 0.0085 | 0.0048 | 0.078961 |
| rs12714702 | MD | G | A | 0.8433 | 6.00E-04 | 0.006 | 0.915 |
| rs12757779 | MD | A | G | 0.2231 | -0.0105 | 0.0052 | 0.0414 |
| rs12762034 | MD | C | T | 0.0775 | 0.0085 | 0.008 | 0.2909 |
| rs1286058 | MD | A | T | 0.7047 | 0.0168 | 0.0047 | 0.000387 |
| rs12881629 | MD | G | A | 0.0804 | 0.0108 | 0.008 | 0.1783 |
| rs12907546 | MD | A | G | 0.2118 | 0.0267 | 0.0052 | 3.52E-07 |
| rs12919291 | MD | C | G | 0.1884 | 0.0327 | 0.0055 | 3.09E-09 |
| rs12940014 | MD | C | T | 0.5226 | 1.00E-04 | 0.0043 | 0.9732 |
| rs12955211 | MD | A | T | 0.3264 | -4.00E-04 | 0.0046 | 0.9307 |
| rs12956148 | MD | A | C | 0.2815 | -0.0052 | 0.0048 | 0.2755 |
| rs1296328 | MD | C | A | 0.558 | 0.0029 | 0.0044 | 0.5036 |
| rs12977787 | MD | A | G | 0.5347 | -0.0037 | 0.0044 | 0.4083 |
| rs13030994 | MD | A | G | 0.4829 | 0.0058 | 0.0043 | 0.1745 |
| rs13037326 | MD | T | C | 0.2597 | 0.031 | 0.0049 | 2.40E-10 |
| rs13090388 | MD | T | C | 0.3064 | -0.0103 | 0.0047 | 0.02844 |
| rs13107325 | MD | T | C | 0.0726 | 0.0242 | 0.0084 | 0.003878 |
| rs13141210 | MD | T | C | 0.527 | -0.0052 | 0.0043 | 0.2273 |
| rs13145650 | MD | T | C | 0.9152 | 0.0076 | 0.0077 | 0.3279 |
| rs13175535 | MD | A | G | 0.2961 | -0.0134 | 0.0048 | 0.005164 |
| rs13195636 | MD | C | A | 0.1064 | -0.055 | 0.007 | 3.84E-15 |
| rs1320251 | MD | T | C | 0.4596 | -0.0021 | 0.0044 | 0.6363 |
| rs13218383 | MD | G | C | 0.3379 | 0.0067 | 0.0045 | 0.1415 |
| rs13233308 | MD | T | C | 0.4835 | -0.0084 | 0.0043 | 0.05052 |
| rs13248187 | MD | C | T | 0.2677 | -0.009 | 0.0049 | 0.0635 |
| rs13261666 | MD | T | G | 0.5091 | -0.0178 | 0.0043 | 3.19E-05 |
| rs1327259 | MD | G | A | 0.3897 | 0.01 | 0.0044 | 0.02298 |
| rs13292699 | MD | C | A | 0.4317 | -0.0096 | 0.0043 | 0.02709 |
| rs1330199 | MD | T | G | 0.4843 | 0.0038 | 0.0043 | 0.3775 |
| rs13307225 | MD | A | G | 0.8872 | -0.0061 | 0.007 | 0.379 |
| rs13317303 | MD | A | C | 0.1488 | 6.00E-04 | 0.0061 | 0.9235 |
| rs1334297 | MD | A | G | 0.7354 | -0.0118 | 0.0049 | 0.01529 |
| rs13422673 | MD | T | C | 0.462 | -0.0035 | 0.0043 | 0.4105 |
| rs13427822 | MD | G | A | 0.2723 | -0.0051 | 0.0049 | 0.3001 |
| rs1346841 | MD | A | G | 0.4028 | -1.00E-04 | 0.0044 | 0.9741 |
| rs1360201 | MD | T | C | 0.4824 | 0.0042 | 0.0043 | 0.3266 |
| rs1363862 | MD | A | G | 0.2745 | -0.0062 | 0.0049 | 0.2041 |
| rs13642 | MD | T | A | 0.3606 | 0.0035 | 0.0045 | 0.4396 |
| rs1391438 | MD | C | T | 0.684 | -0.0031 | 0.0046 | 0.510199 |
| rs1392816 | MD | T | C | 0.3865 | -0.0155 | 0.0044 | 0.000497 |
| rs140159717 | MD | T | C | 0.08 | -6.00E-04 | 0.0082 | 0.9425 |
| rs1438945 | MD | A | T | 0.7148 | -0.0086 | 0.0048 | 0.07254 |
| rs1441264 | MD | A | G | 0.5943 | 0.0014 | 0.0045 | 0.7604 |
| rs1450782 | MD | G | T | 0.5661 | 0.0015 | 0.0044 | 0.726001 |
| rs1455350 | MD | A | T | 0.4804 | 0.0017 | 0.0043 | 0.691301 |
| rs1458156 | MD | T | C | 0.489 | 0.0064 | 0.0043 | 0.1352 |
| rs1471740 | MD | C | T | 0.7356 | -0.0027 | 0.0049 | 0.5902 |
| rs147568678 | MD | C | T | 0.2373 | -0.0039 | 0.0051 | 0.4423 |
| rs1477290 | MD | C | T | 0.1366 | 0.0234 | 0.0063 | 0.000197 |
| rs1503526 | MD | C | T | 0.4811 | 0.0026 | 0.0043 | 0.548399 |
| rs152603 | MD | G | A | 0.3649 | -0.0054 | 0.0045 | 0.2284 |
| rs1565735 | MD | A | T | 0.2033 | -0.0019 | 0.0054 | 0.725499 |
| rs1566085 | MD | T | G | 0.545 | -0.0013 | 0.0044 | 0.7681 |
| rs1582931 | MD | A | G | 0.4722 | 0.0047 | 0.0043 | 0.2832 |
| rs1584469 | MD | T | C | 0.294 | 0.0144 | 0.0047 | 0.00228 |
| rs1609010 | MD | G | A | 0.5651 | 0.002 | 0.0043 | 0.649 |
| rs1671770 | MD | C | A | 0.8209 | 0.0065 | 0.0057 | 0.2539 |
| rs16846140 | MD | G | A | 0.3374 | 0.0141 | 0.0046 | 0.001962 |
| rs16846463 | MD | G | A | 0.1 | 0.0092 | 0.0071 | 0.1965 |
| rs16854920 | MD | C | T | 0.3425 | -0.0063 | 0.0046 | 0.1674 |
| rs1689510 | MD | C | G | 0.3369 | -5.00E-04 | 0.0046 | 0.9194 |
| rs16916303 | MD | G | A | 0.1202 | 0.0066 | 0.0067 | 0.3245 |
| rs16995054 | MD | T | C | 0.2091 | -0.0027 | 0.0053 | 0.6058 |
| rs17193211 | MD | T | C | 0.0679 | -0.0152 | 0.0087 | 0.079721 |
| rs17194490 | MD | T | G | 0.1647 | 0.0072 | 0.0059 | 0.2208 |
| rs17399739 | MD | G | A | 0.0692 | -0.0052 | 0.0088 | 0.5571 |
| rs17565975 | MD | A | G | 0.5619 | 0.0057 | 0.0044 | 0.1885 |
| rs17598675 | MD | C | T | 0.4856 | -0.0041 | 0.0043 | 0.3457 |
| rs176218 | MD | T | G | 0.1926 | -0.0017 | 0.0055 | 0.7596 |
| rs1778830 | MD | A | G | 0.364 | 0.0012 | 0.0045 | 0.788 |
| rs1788808 | MD | G | A | 0.493 | 0.0074 | 0.0043 | 0.086181 |
| rs1834144 | MD | A | C | 0.3734 | 0.0015 | 0.0045 | 0.741 |
| rs1860002 | MD | T | C | 0.5393 | -0.0077 | 0.0043 | 0.074781 |
| rs1899896 | MD | T | C | 0.2986 | 0.0081 | 0.0047 | 0.085349 |
| rs1901512 | MD | C | T | 0.6961 | -0.0057 | 0.0047 | 0.2282 |
| rs1915019 | MD | G | A | 0.7589 | 5.00E-04 | 0.005 | 0.9158 |
| rs1919243 | MD | C | T | 0.4846 | -0.0017 | 0.0044 | 0.7032 |
| rs1950829 | MD | G | A | 0.5173 | -0.0297 | 0.0043 | 4.74E-12 |
| rs1967772 | MD | A | G | 0.2843 | 7.00E-04 | 0.0048 | 0.8771 |
| rs2035936 | MD | T | G | 0.0572 | -0.0076 | 0.0094 | 0.419 |
| rs2076603 | MD | A | G | 0.6413 | 0.0017 | 0.0045 | 0.706999 |
| rs2084572 | MD | G | A | 0.4493 | 0.0074 | 0.0043 | 0.086551 |
| rs2102278 | MD | G | A | 0.3191 | -2.00E-04 | 0.0046 | 0.9723 |
| rs2133561 | MD | T | A | 0.6124 | -0.0019 | 0.0045 | 0.6699 |
| rs213518 | MD | C | T | 0.1437 | 0.0029 | 0.0061 | 0.6392 |
| rs2153740 | MD | G | A | 0.4798 | 0.0021 | 0.0043 | 0.6305 |
| rs215634 | MD | G | A | 0.6138 | -0.0091 | 0.0044 | 0.0407 |
| rs2172131 | MD | C | T | 0.5746 | -0.0116 | 0.0045 | 0.009182 |
| rs217336 | MD | A | C | 0.4378 | 0.0029 | 0.0043 | 0.5083 |
| rs2174752 | MD | T | G | 0.454 | 0.0076 | 0.0043 | 0.078921 |
| rs2176337 | MD | T | A | 0.3159 | 0.004 | 0.0046 | 0.3842 |
| rs217672 | MD | C | A | 0.2704 | 0.0017 | 0.0049 | 0.7302 |
| rs2214123 | MD | G | A | 0.6466 | -0.0261 | 0.0045 | 8.56E-09 |
| rs2234458 | MD | T | C | 0.6421 | -0.008 | 0.0045 | 0.07536 |
| rs2253310 | MD | G | C | 0.6267 | 0.0034 | 0.0044 | 0.4383 |
| rs2281819 | MD | A | T | 0.2299 | 0.002 | 0.0051 | 0.7009 |
| rs2283076 | MD | G | A | 0.2269 | 0.0154 | 0.0052 | 0.002762 |
| rs2289379 | MD | T | C | 0.3971 | 0.0122 | 0.0044 | 0.005672 |
| rs2302761 | MD | T | C | 0.2113 | -0.0043 | 0.0053 | 0.4126 |
| rs2306593 | MD | T | C | 0.4826 | 0.0039 | 0.0043 | 0.3715 |
| rs2307111 | MD | C | T | 0.395 | -0.0021 | 0.0044 | 0.633 |
| rs2332700 | MD | G | C | 0.7523 | -0.0163 | 0.005 | 0.001061 |
| rs2333321 | MD | G | A | 0.7923 | -0.0229 | 0.0053 | 1.51E-05 |
| rs2342892 | MD | G | T | 0.5177 | -0.0026 | 0.0043 | 0.547 |
| rs2347526 | MD | C | T | 0.6585 | -0.0148 | 0.0045 | 0.001151 |
| rs2396625 | MD | A | T | 0.4231 | -0.0025 | 0.0044 | 0.574101 |
| rs2398861 | MD | G | A | 0.26 | 0.0031 | 0.0049 | 0.5247 |
| rs240963 | MD | C | T | 0.8394 | -0.0185 | 0.0059 | 0.001632 |
| rs242093 | MD | A | G | 0.5709 | 0.0091 | 0.0044 | 0.03821 |
| rs2433733 | MD | A | G | 0.6768 | -0.005 | 0.0046 | 0.2782 |
| rs2456020 | MD | T | C | 0.2306 | -0.0076 | 0.0051 | 0.1379 |
| rs2482356 | MD | C | T | 0.4259 | -0.0056 | 0.0044 | 0.2012 |
| rs252761 | MD | T | G | 0.5896 | -0.0094 | 0.0044 | 0.03287 |
| rs2554835 | MD | A | G | 0.4002 | 0.0034 | 0.0044 | 0.4379 |
| rs2568958 | MD | A | G | 0.6042 | 0.0382 | 0.0044 | 2.90E-18 |
| rs2606228 | MD | C | A | 0.6449 | -0.003 | 0.0045 | 0.5107 |
| rs2612030 | MD | C | T | 0.8402 | -0.006 | 0.0059 | 0.3063 |
| rs2616143 | MD | A | G | 0.3214 | -0.0147 | 0.0046 | 0.001425 |
| rs266047 | MD | A | G | 0.5347 | -0.0089 | 0.0043 | 0.03982 |
| rs2678204 | MD | G | T | 0.3375 | 0.0032 | 0.0046 | 0.4851 |
| rs2781668 | MD | T | C | 0.1646 | -0.0012 | 0.0058 | 0.838 |
| rs2787101 | MD | T | C | 0.605 | -0.0085 | 0.0044 | 0.05464 |
| rs2819336 | MD | C | T | 0.6376 | 0.0095 | 0.0045 | 0.03332 |
| rs28373063 | MD | C | G | 0.1778 | -0.0248 | 0.0057 | 1.31E-05 |
| rs28404639 | MD | T | C | 0.3614 | -0.007 | 0.0045 | 0.1163 |
| rs28489620 | MD | A | G | 0.2912 | -0.0068 | 0.0048 | 0.1553 |
| rs28513670 | MD | G | A | 0.1719 | -0.0047 | 0.0057 | 0.4097 |
| rs2875762 | MD | C | G | 0.2424 | -0.0022 | 0.0051 | 0.6593 |
| rs2876520 | MD | G | C | 0.4688 | 0.026 | 0.0043 | 2.24E-09 |
| rs2920503 | MD | T | C | 0.2865 | -0.0141 | 0.0048 | 0.003312 |
| rs2923431 | MD | C | G | 0.6306 | -0.0054 | 0.0045 | 0.2243 |
| rs293566 | MD | C | T | 0.3353 | 0.0073 | 0.0046 | 0.1101 |
| rs2962334 | MD | T | G | 0.0206 | -0.0152 | 0.0151 | 0.3152 |
| rs2971970 | MD | G | T | 0.7827 | 6.00E-04 | 0.0052 | 0.9082 |
| rs301800 | MD | C | T | 0.8233 | -0.0085 | 0.0056 | 0.1325 |
| rs30266 | MD | A | G | 0.3271 | 0.0366 | 0.0046 | 1.43E-15 |
| rs317656 | MD | A | T | 0.7231 | -0.0092 | 0.0048 | 0.05599 |
| rs320693 | MD | C | G | 0.4566 | -0.0053 | 0.0043 | 0.2205 |
| rs3211995 | MD | A | G | 0.1594 | -0.0065 | 0.0061 | 0.2913 |
| rs329118 | MD | T | C | 0.4196 | -0.0121 | 0.0044 | 0.005742 |
| rs34025316 | MD | T | C | 0.3366 | -0.0017 | 0.0046 | 0.708401 |
| rs34045288 | MD | T | C | 0.3323 | 0.0017 | 0.0046 | 0.7035 |
| rs34234296 | MD | A | G | 0.3901 | -0.0104 | 0.0045 | 0.02074 |
| rs34481751 | MD | A | C | 0.164 | 0.0077 | 0.0059 | 0.1939 |
| rs34517439 | MD | A | C | 0.1208 | -9.00E-04 | 0.0067 | 0.8906 |
| rs347551 | MD | G | C | 0.4694 | 0.0026 | 0.0044 | 0.5643 |
| rs34811474 | MD | A | G | 0.23 | -0.0071 | 0.0051 | 0.1665 |
| rs35154326 | MD | G | A | 0.2761 | -3.00E-04 | 0.0049 | 0.953 |
| rs35309068 | MD | G | T | 0.4369 | 0.0037 | 0.0043 | 0.3876 |
| rs354155 | MD | C | G | 0.0923 | -0.0449 | 0.0075 | 1.75E-09 |
| rs35417702 | MD | T | C | 0.5248 | 0.0072 | 0.0043 | 0.096661 |
| rs35475880 | MD | T | G | 0.2073 | 8.00E-04 | 0.0053 | 0.8732 |
| rs35532491 | MD | T | A | 0.103 | -0.0081 | 0.0071 | 0.259 |
| rs355777 | MD | C | G | 0.4079 | 0.0073 | 0.0044 | 0.09533 |
| rs35867081 | MD | G | A | 0.5151 | -0.001 | 0.0044 | 0.823 |
| rs36007635 | MD | A | G | 0.1375 | -0.0104 | 0.0062 | 0.09643 |
| rs36061954 | MD | T | C | 0.3982 | 0.0079 | 0.0044 | 0.072979 |
| rs36119825 | MD | A | G | 0.4437 | -0.0046 | 0.0043 | 0.2825 |
| rs363096 | MD | C | T | 0.5749 | -0.0145 | 0.0045 | 0.001306 |
| rs3747631 | MD | C | G | 0.2102 | -0.0075 | 0.0053 | 0.1538 |
| rs3764625 | MD | G | T | 0.5871 | -0.0013 | 0.0044 | 0.7635 |
| rs3770754 | MD | G | C | 0.3599 | 0.003 | 0.0045 | 0.5017 |
| rs3784710 | MD | C | T | 0.2268 | -0.0121 | 0.0051 | 0.01794 |
| rs3800546 | MD | G | C | 0.2583 | 0.0082 | 0.0049 | 0.095821 |
| rs3807865 | MD | A | G | 0.4105 | 0.031 | 0.0044 | 1.09E-12 |
| rs3809634 | MD | G | A | 0.3142 | 0.0023 | 0.0047 | 0.6145 |
| rs3814883 | MD | T | C | 0.4811 | -0.0145 | 0.0043 | 0.000845 |
| rs3845344 | MD | T | C | 0.3898 | 0.0098 | 0.0044 | 0.02551 |
| rs3851998 | MD | G | C | 0.7439 | -0.0012 | 0.0049 | 0.8033 |
| rs3896224 | MD | G | A | 0.4167 | -0.0268 | 0.0044 | 1.29E-09 |
| rs3897821 | MD | G | A | 0.3335 | -0.0062 | 0.0046 | 0.1745 |
| rs3901286 | MD | A | C | 0.1514 | -0.0106 | 0.006 | 0.076611 |
| rs394608 | MD | C | T | 0.5366 | 0.0195 | 0.0045 | 1.32E-05 |
| rs40071 | MD | C | T | 0.179 | -0.0149 | 0.0057 | 0.008704 |
| rs4044321 | MD | G | A | 0.6414 | -0.0159 | 0.0045 | 0.000407 |
| rs4055791 | MD | T | C | 0.4174 | 0.003 | 0.0044 | 0.4888 |
| rs406388 | MD | G | C | 0.1783 | -0.0129 | 0.0057 | 0.02202 |
| rs4148155 | MD | G | A | 0.1121 | 0.007 | 0.0068 | 0.3055 |
| rs4261944 | MD | G | T | 0.3642 | 0.0043 | 0.0045 | 0.3316 |
| rs4267103 | MD | C | T | 0.1845 | -0.0045 | 0.0056 | 0.414 |
| rs429343 | MD | G | A | 0.5784 | 0.0136 | 0.0044 | 0.00176 |
| rs429358 | MD | C | T | 0.1514 | -5.00E-04 | 0.0062 | 0.9408 |
| rs4328757 | MD | T | C | 0.6128 | 0.0032 | 0.0044 | 0.4693 |
| rs4352658 | MD | T | C | 0.0811 | -5.00E-04 | 0.0079 | 0.9527 |
| rs4382592 | MD | G | T | 0.6982 | -0.0076 | 0.0047 | 0.1036 |
| rs4419475 | MD | T | A | 0.4065 | 0.0035 | 0.0044 | 0.4242 |
| rs4439537 | MD | C | T | 0.5222 | 0.0047 | 0.0043 | 0.273 |
| rs4477562 | MD | T | C | 0.1283 | 0.0079 | 0.0065 | 0.2216 |
| rs4482463 | MD | A | C | 0.9217 | 0.0073 | 0.0081 | 0.3631 |
| rs4613074 | MD | C | T | 0.1833 | 0.0114 | 0.0056 | 0.03954 |
| rs4648450 | MD | A | C | 0.471 | -0.0012 | 0.0044 | 0.788199 |
| rs4653164 | MD | T | C | 0.6878 | 0.0113 | 0.0046 | 0.01487 |
| rs4671328 | MD | G | T | 0.551 | 0.0111 | 0.0044 | 0.01166 |
| rs4700393 | MD | G | A | 0.5218 | 0.0055 | 0.0043 | 0.1983 |
| rs4702 | MD | A | G | 0.5585 | -0.0178 | 0.0044 | 4.98E-05 |
| rs4709807 | MD | C | T | 0.7604 | -3.00E-04 | 0.005 | 0.9501 |
| rs4737188 | MD | T | A | 0.4713 | 0.0158 | 0.0043 | 0.000244 |
| rs4757144 | MD | A | G | 0.5903 | -0.0059 | 0.0044 | 0.1798 |
| rs4757957 | MD | C | G | 0.684 | 0.0041 | 0.0046 | 0.3776 |
| rs4764949 | MD | G | A | 0.3267 | 0.0032 | 0.0046 | 0.4869 |
| rs4790292 | MD | A | C | 0.1536 | 0.0071 | 0.006 | 0.238 |
| rs4810227 | MD | A | G | 0.6195 | 0.0056 | 0.0045 | 0.2124 |
| rs4812325 | MD | A | G | 0.6104 | 0.0151 | 0.0044 | 0.000666 |
| rs4832298 | MD | T | C | 0.6887 | 0.0042 | 0.0047 | 0.3646 |
| rs4846724 | MD | A | G | 0.5338 | -0.0025 | 0.0043 | 0.5592 |
| rs4876611 | MD | G | A | 0.7207 | 0.0021 | 0.0048 | 0.6549 |
| rs4895799 | MD | T | C | 0.5837 | -0.004 | 0.0044 | 0.3592 |
| rs4958702 | MD | C | T | 0.5723 | 0.0139 | 0.0043 | 0.001353 |
| rs4961705 | MD | C | G | 0.3475 | -0.0057 | 0.0046 | 0.2112 |
| rs512121 | MD | C | T | 0.1925 | -0.0079 | 0.0055 | 0.1496 |
| rs539515 | MD | C | A | 0.2032 | 0.0013 | 0.0053 | 0.8144 |
| rs55658481 | MD | A | G | 0.3391 | 0.0032 | 0.0045 | 0.4806 |
| rs55707359 | MD | G | T | 0.0147 | 0.0532 | 0.0186 | 0.004235 |
| rs55714539 | MD | C | A | 0.3415 | -0.0026 | 0.0046 | 0.5784 |
| rs55726687 | MD | A | G | 0.2091 | 0.0066 | 0.0053 | 0.2104 |
| rs55736314 | MD | G | C | 0.4035 | -0.0057 | 0.0044 | 0.1947 |
| rs558887 | MD | G | A | 0.3067 | -0.0082 | 0.0047 | 0.078741 |
| rs56094641 | MD | G | A | 0.4068 | 0.0104 | 0.0044 | 0.01688 |
| rs56133507 | MD | G | T | 0.1966 | -0.0014 | 0.0054 | 0.8002 |
| rs56161855 | MD | T | A | 0.1333 | 0.0082 | 0.0063 | 0.1954 |
| rs56203622 | MD | C | T | 0.1452 | -5.00E-04 | 0.0061 | 0.9382 |
| rs56335113 | MD | G | A | 0.6921 | -0.004 | 0.0047 | 0.3991 |
| rs56356382 | MD | C | T | 0.189 | -0.0061 | 0.0056 | 0.2815 |
| rs56391344 | MD | A | G | 0.2555 | -0.0036 | 0.0049 | 0.4654 |
| rs56399737 | MD | T | C | 0.4482 | -0.0055 | 0.0043 | 0.2047 |
| rs56858768 | MD | A | G | 0.297 | 0.0106 | 0.0047 | 0.02508 |
| rs57636386 | MD | C | T | 0.085 | 0.0122 | 0.0077 | 0.1158 |
| rs58120505 | MD | C | T | 0.4327 | 0.0037 | 0.0044 | 0.3983 |
| rs59086897 | MD | A | T | 0.4758 | 0.0091 | 0.0043 | 0.03531 |
| rs59237168 | MD | C | T | 0.2165 | -0.0064 | 0.0052 | 0.2193 |
| rs5995843 | MD | G | A | 0.348 | 0.0136 | 0.0045 | 0.002485 |
| rs60764613 | MD | T | G | 0.1458 | 0.0193 | 0.0061 | 0.001577 |
| rs6123924 | MD | G | A | 0.1542 | 0.0023 | 0.006 | 0.6975 |
| rs6134916 | MD | T | C | 0.4917 | 0.0069 | 0.0043 | 0.1085 |
| rs61813324 | MD | T | C | 0.1349 | -0.0076 | 0.0065 | 0.2383 |
| rs61828088 | MD | A | G | 0.1111 | 0.0012 | 0.0068 | 0.8619 |
| rs61903695 | MD | G | A | 0.2523 | 0.0109 | 0.0049 | 0.02775 |
| rs61914045 | MD | A | G | 0.2034 | 0.0309 | 0.0054 | 7.96E-09 |
| rs62097985 | MD | T | C | 0.4222 | 0.0242 | 0.0044 | 3.13E-08 |
| rs62107261 | MD | C | T | 0.0481 | -0.0014 | 0.0103 | 0.8942 |
| rs62134195 | MD | T | C | 0.0414 | -3.00E-04 | 0.0109 | 0.9815 |
| rs62176243 | MD | T | A | 0.2464 | -0.0091 | 0.005 | 0.069091 |
| rs62176993 | MD | A | G | 0.3978 | 0.0021 | 0.0044 | 0.632199 |
| rs62190049 | MD | C | G | 0.3906 | -0.0024 | 0.0045 | 0.6005 |
| rs62439690 | MD | A | G | 0.2607 | 0.0028 | 0.005 | 0.5718 |
| rs6265 | MD | T | C | 0.1891 | 0.0173 | 0.0055 | 0.001589 |
| rs6531639 | MD | A | G | 0.2464 | -0.0045 | 0.0051 | 0.3815 |
| rs6545714 | MD | A | G | 0.6047 | -0.0025 | 0.0044 | 0.5758 |
| rs6546857 | MD | G | A | 0.2383 | 0.0019 | 0.0051 | 0.706401 |
| rs6560906 | MD | C | T | 0.6884 | -0.0058 | 0.0046 | 0.2097 |
| rs6567160 | MD | C | T | 0.2347 | -0.0158 | 0.0051 | 0.001818 |
| rs6575340 | MD | A | G | 0.6373 | 0.0133 | 0.0045 | 0.0029 |
| rs66511648 | MD | C | T | 0.284 | 0.0297 | 0.0048 | 6.03E-10 |
| rs66679256 | MD | T | C | 0.447 | -0.0096 | 0.0043 | 0.02631 |
| rs6669341 | MD | G | A | 0.5857 | -0.0181 | 0.0044 | 3.15E-05 |
| rs6682438 | MD | C | T | 0.6738 | 0.0113 | 0.0046 | 0.01405 |
| rs6688826 | MD | C | T | 0.2948 | 0.0156 | 0.0047 | 0.000928 |
| rs6707827 | MD | G | A | 0.7032 | 0.0132 | 0.0048 | 0.005596 |
| rs6710091 | MD | G | C | 0.3499 | -0.0061 | 0.0045 | 0.1769 |
| rs6719762 | MD | C | T | 0.4735 | 0.0118 | 0.0043 | 0.006138 |
| rs6731967 | MD | C | G | 0.236 | -0.0023 | 0.0051 | 0.651199 |
| rs6744646 | MD | G | A | 0.8281 | 0.006 | 0.0057 | 0.2913 |
| rs6744794 | MD | G | C | 0.6208 | 0.0054 | 0.0044 | 0.2225 |
| rs6752979 | MD | A | G | 0.3157 | 0.0058 | 0.0046 | 0.2098 |
| rs6774894 | MD | A | T | 0.3589 | -9.00E-04 | 0.0045 | 0.8366 |
| rs6798742 | MD | G | A | 0.2982 | -0.0042 | 0.0047 | 0.3662 |
| rs6803651 | MD | T | G | 0.4342 | 0.0084 | 0.0044 | 0.053361 |
| rs6805241 | MD | C | T | 0.2291 | -0.0037 | 0.0051 | 0.4722 |
| rs6843852 | MD | T | C | 0.5109 | 0.0082 | 0.0043 | 0.05691 |
| rs6943762 | MD | C | T | 0.1269 | -0.014 | 0.0065 | 0.03089 |
| rs6959891 | MD | G | A | 0.2833 | 0.0038 | 0.0048 | 0.4217 |
| rs6962980 | MD | C | A | 0.555 | -0.0041 | 0.0043 | 0.3394 |
| rs6974218 | MD | C | A | 0.3822 | -0.0131 | 0.0044 | 0.003076 |
| rs698147 | MD | G | A | 0.5446 | 0.0045 | 0.0043 | 0.2969 |
| rs7008955 | MD | G | T | 0.5263 | -0.0074 | 0.0043 | 0.0875 |
| rs7012546 | MD | T | C | 0.4158 | 0.0038 | 0.0044 | 0.3884 |
| rs7029718 | MD | A | G | 0.4145 | 1.00E-04 | 0.0044 | 0.9744 |
| rs7031698 | MD | C | T | 0.7758 | -0.0195 | 0.0052 | 0.000163 |
| rs7034554 | MD | G | A | 0.3738 | -0.017 | 0.0044 | 0.000131 |
| rs7038943 | MD | C | T | 0.3429 | -0.0101 | 0.0045 | 0.02535 |
| rs7079070 | MD | A | G | 0.4516 | 0.0051 | 0.0044 | 0.2433 |
| rs708228 | MD | T | C | 0.3269 | 0.0208 | 0.0046 | 5.18E-06 |
| rs7124681 | MD | A | C | 0.4084 | -0.0145 | 0.0044 | 0.000878 |
| rs7132908 | MD | A | G | 0.3859 | 0.0041 | 0.0044 | 0.3519 |
| rs71646142 | MD | T | C | 0.1908 | -0.007 | 0.0055 | 0.2048 |
| rs7201895 | MD | A | G | 0.3486 | -0.0087 | 0.0046 | 0.060239 |
| rs7206608 | MD | G | C | 0.3195 | 0.0088 | 0.0046 | 0.05786 |
| rs7218014 | MD | C | T | 0.198 | 0.0109 | 0.0054 | 0.04213 |
| rs7233920 | MD | A | G | 0.2277 | 0.0041 | 0.0051 | 0.4252 |
| rs7236339 | MD | A | G | 0.2244 | 0.0286 | 0.0052 | 3.28E-08 |
| rs723672 | MD | T | C | 0.4311 | 0.0023 | 0.0044 | 0.597301 |
| rs7250833 | MD | T | C | 0.2892 | 0.0017 | 0.0047 | 0.7123 |
| rs7259070 | MD | C | T | 0.5917 | 0.0037 | 0.0045 | 0.4109 |
| rs72673947 | MD | G | A | 0.1071 | -0.0309 | 0.007 | 1.11E-05 |
| rs7278859 | MD | T | A | 0.3099 | -0.0052 | 0.0047 | 0.2607 |
| rs72887338 | MD | C | T | 0.3852 | 0.0115 | 0.0044 | 0.009061 |
| rs72892910 | MD | T | G | 0.1738 | 0.0061 | 0.0057 | 0.2857 |
| rs72910629 | MD | G | A | 0.1353 | -0.0105 | 0.0063 | 0.098601 |
| rs72986630 | MD | T | C | 0.0636 | -0.013 | 0.0092 | 0.1583 |
| rs73026725 | MD | A | C | 0.1532 | -0.0145 | 0.006 | 0.01506 |
| rs730384 | MD | A | G | 0.4407 | -0.0093 | 0.0044 | 0.03339 |
| rs73052033 | MD | C | T | 0.1841 | -0.0106 | 0.0056 | 0.05693 |
| rs73142879 | MD | T | C | 0.1914 | -0.0195 | 0.0055 | 0.000393 |
| rs73213484 | MD | T | A | 0.1436 | -0.0111 | 0.0061 | 0.070821 |
| rs7331420 | MD | A | G | 0.2879 | -0.0105 | 0.0048 | 0.02687 |
| rs7332724 | MD | T | C | 0.2747 | 0.0066 | 0.0048 | 0.1683 |
| rs73344830 | MD | G | A | 0.5783 | -0.0013 | 0.0044 | 0.767499 |
| rs7357754 | MD | G | A | 0.5014 | -0.0048 | 0.0043 | 0.264 |
| rs7359501 | MD | T | C | 0.3958 | 0.005 | 0.0044 | 0.2511 |
| rs736282 | MD | C | T | 0.5238 | 0.0061 | 0.0043 | 0.1547 |
| rs7442137 | MD | T | C | 0.6371 | -0.0056 | 0.0045 | 0.2102 |
| rs7442885 | MD | G | C | 0.2152 | 0.0122 | 0.0052 | 0.01936 |
| rs745249 | MD | T | C | 0.2826 | 0.0194 | 0.0048 | 5.32E-05 |
| rs7498044 | MD | A | G | 0.218 | -0.0088 | 0.0053 | 0.09383 |
| rs7498665 | MD | G | A | 0.3995 | -7.00E-04 | 0.0044 | 0.8757 |
| rs75035127 | MD | G | A | 0.0302 | -0.0097 | 0.0127 | 0.4445 |
| rs7551758 | MD | G | T | 0.5329 | 0.0283 | 0.0043 | 5.11E-11 |
| rs7575189 | MD | A | G | 0.5879 | 9.00E-04 | 0.0044 | 0.8352 |
| rs7594904 | MD | C | T | 0.4141 | -0.0055 | 0.0044 | 0.2043 |
| rs76076331 | MD | T | C | 0.1268 | -0.0079 | 0.0066 | 0.2257 |
| rs76702514 | MD | G | C | 0.2088 | 2.00E-04 | 0.0053 | 0.971 |
| rs76878669 | MD | G | C | 0.2359 | -0.0105 | 0.0051 | 0.04026 |
| rs76954012 | MD | A | T | 0.0931 | 0.0412 | 0.0074 | 2.41E-08 |
| rs7704530 | MD | A | G | 0.7308 | 0.0064 | 0.0049 | 0.1844 |
| rs7762794 | MD | G | A | 0.2872 | -0.0147 | 0.0047 | 0.001885 |
| rs77702622 | MD | A | G | 0.0637 | 0.0121 | 0.0089 | 0.1746 |
| rs7774 | MD | A | C | 0.3114 | 0.001 | 0.0047 | 0.8386 |
| rs77835879 | MD | G | A | 0.0994 | 0.0136 | 0.0073 | 0.0618 |
| rs778371 | MD | G | A | 0.2812 | -0.0076 | 0.0048 | 0.1143 |
| rs7785195 | MD | A | G | 0.6574 | -0.009 | 0.0045 | 0.04573 |
| rs7803932 | MD | A | G | 0.1699 | -0.0073 | 0.0058 | 0.2065 |
| rs78086698 | MD | C | T | 0.0396 | -0.0159 | 0.0113 | 0.16 |
| rs7852189 | MD | G | A | 0.3177 | 0.0111 | 0.0046 | 0.01547 |
| rs7893571 | MD | T | G | 0.6664 | -0.0057 | 0.0047 | 0.2291 |
| rs7909331 | MD | G | A | 0.1656 | 0.0057 | 0.006 | 0.3398 |
| rs7920624 | MD | T | A | 0.5336 | -0.0091 | 0.0043 | 0.03464 |
| rs7921378 | MD | C | G | 0.4805 | 0.0057 | 0.0043 | 0.1832 |
| rs7924036 | MD | T | G | 0.5072 | -0.0044 | 0.0043 | 0.3046 |
| rs79265434 | MD | G | A | 0.1182 | 0.0116 | 0.0067 | 0.08336 |
| rs79269403 | MD | A | G | 0.2277 | -8.00E-04 | 0.0052 | 0.8727 |
| rs7927195 | MD | G | A | 0.6148 | -0.0052 | 0.0044 | 0.2398 |
| rs7928622 | MD | T | A | 0.3201 | 5.00E-04 | 0.0046 | 0.9059 |
| rs7938812 | MD | G | T | 0.3884 | 0.0203 | 0.0044 | 3.96E-06 |
| rs79445414 | MD | C | T | 0.0465 | -0.0042 | 0.0104 | 0.6832 |
| rs7944782 | MD | G | T | 0.5118 | -0.008 | 0.0043 | 0.063281 |
| rs7952102 | MD | C | T | 0.3895 | 4.00E-04 | 0.0045 | 0.9314 |
| rs7996639 | MD | A | G | 0.4494 | -0.0033 | 0.0043 | 0.449 |
| rs80153284 | MD | A | C | 0.0149 | 0.015 | 0.0185 | 0.4161 |
| rs8015400 | MD | A | C | 0.6758 | 0.0019 | 0.0046 | 0.684301 |
| rs8020034 | MD | A | G | 0.1823 | 0.0022 | 0.0056 | 0.6957 |
| rs8112818 | MD | G | A | 0.4033 | 0 | 0.0044 | 0.9929 |
| rs8132491 | MD | A | G | 0.3118 | -0.0063 | 0.005 | 0.2032 |
| rs815163 | MD | C | T | 0.5673 | -0.0043 | 0.0043 | 0.316 |
| rs862320 | MD | T | C | 0.4139 | -0.0104 | 0.0044 | 0.01697 |
| rs879620 | MD | T | C | 0.6118 | -0.0102 | 0.0045 | 0.0233 |
| rs892612 | MD | C | A | 0.8465 | -0.0026 | 0.006 | 0.659601 |
| rs9294260 | MD | A | G | 0.4745 | 0.0065 | 0.0043 | 0.1355 |
| rs9296389 | MD | C | G | 0.4113 | 0.0049 | 0.0044 | 0.257 |
| rs9320493 | MD | G | A | 0.8504 | -0.0033 | 0.006 | 0.5856 |
| rs9349956 | MD | C | A | 0.1838 | -4.00E-04 | 0.0056 | 0.9416 |
| rs935166 | MD | A | G | 0.5084 | 0 | 0.0043 | 0.9974 |
| rs9366863 | MD | C | T | 0.6713 | -0.0027 | 0.0046 | 0.551099 |
| rs9372625 | MD | A | G | 0.381 | 0.0017 | 0.0044 | 0.6998 |
| rs9386319 | MD | G | A | 0.3908 | 0.0012 | 0.0044 | 0.7926 |
| rs9478496 | MD | C | T | 0.1652 | 0.0082 | 0.0058 | 0.158 |
| rs9503598 | MD | A | G | 0.4412 | 0.0019 | 0.0044 | 0.6666 |
| rs9514600 | MD | G | C | 0.5024 | 0.0028 | 0.0043 | 0.5209 |
| rs9522173 | MD | T | A | 0.3931 | -0.0157 | 0.0044 | 0.000355 |
| rs9529119 | MD | G | C | 0.7782 | 0.0305 | 0.0052 | 4.64E-09 |
| rs9571687 | MD | A | C | 0.3271 | -0.0129 | 0.0046 | 0.004806 |
| rs9616906 | MD | A | G | 0.4403 | -4.00E-04 | 0.0044 | 0.9185 |
| rs9636107 | MD | G | A | 0.4705 | 0.0215 | 0.0043 | 6.48E-07 |
| rs9643087 | MD | T | C | 0.5246 | 0.0138 | 0.0043 | 0.001474 |
| rs969512 | MD | T | A | 0.336 | -0.009 | 0.0046 | 0.04729 |
| rs9704097 | MD | A | C | 0.5073 | 0.0047 | 0.0043 | 0.2728 |
| rs9852062 | MD | A | T | 0.5576 | -0.0076 | 0.0043 | 0.08007 |
| rs9860326 | MD | G | C | 0.3291 | 0.0058 | 0.0046 | 0.2044 |
| rs9882532 | MD | C | T | 0.3582 | 0.0105 | 0.0045 | 0.01959 |
| rs9888533 | MD | T | C | 0.5398 | 0.0085 | 0.0044 | 0.05462 |
| rs9951619 | MD | G | T | 0.7666 | 3.00E-04 | 0.0051 | 0.9566 |
| rs9964724 | MD | T | C | 0.6823 | -0.0279 | 0.0046 | 1.79E-09 |
| rs1000237 | SCZ | A | T | 0.355 | 0.073205 | 0.0089 | 2.80E-16 |
| rs10073890 | SCZ | G | A | 0.746 | -0.011602 | 0.0099 | 0.2372 |
| rs10160769 | SCZ | C | G | 0.234 | 0.014698 | 0.0102 | 0.1486 |
| rs1017529 | SCZ | A | C | 0.161 | -0.031198 | 0.0121 | 0.009719 |
| rs10423928 | SCZ | A | T | 0.213 | 0.013095 | 0.0108 | 0.2268 |
| rs10505836 | SCZ | C | A | 0.852 | -0.003703 | 0.0121 | 0.7634 |
| rs10510025 | SCZ | T | C | 0.253 | 0.006199 | 0.0099 | 0.5284 |
| rs1064213 | SCZ | A | G | 0.487 | -0.0444 | 0.0086 | 2.40E-07 |
| rs10742752 | SCZ | C | T | 0.613 | -0.017801 | 0.0088 | 0.04342 |
| rs10752613 | SCZ | A | T | 0.304 | 0.037598 | 0.0094 | 6.48E-05 |
| rs10765775 | SCZ | A | G | 0.387 | 0.010697 | 0.0089 | 0.2309 |
| rs10773002 | SCZ | T | A | 0.743 | -0.070896 | 0.0098 | 3.77E-13 |
| rs10797055 | SCZ | G | A | 0.484 | -0.003604 | 0.0087 | 0.6822 |
| rs10858054 | SCZ | T | G | 0.18 | 0.024395 | 0.0112 | 0.0294 |
| rs10861176 | SCZ | A | G | 0.737 | 0.055502 | 0.0098 | 1.59E-08 |
| rs10887578 | SCZ | C | G | 0.491 | -0.008504 | 0.0087 | 0.3318 |
| rs10887801 | SCZ | T | G | 0.433 | -0.0005 | 0.0087 | 0.9538 |
| rs10922907 | SCZ | T | A | 0.53 | -0.034604 | 0.0087 | 6.67E-05 |
| rs10938398 | SCZ | A | G | 0.431 | -0.023297 | 0.0087 | 0.007717 |
| rs10963297 | SCZ | G | C | 0.242 | 0.010505 | 0.0101 | 0.2985 |
| rs11000993 | SCZ | C | T | 0.122 | -0.020401 | 0.0133 | 0.1234 |
| rs11012732 | SCZ | G | A | 0.339 | -0.010703 | 0.0091 | 0.2377 |
| rs1105307 | SCZ | A | G | 0.262 | 0.031305 | 0.0098 | 0.001333 |
| rs11079849 | SCZ | T | C | 0.325 | -0.020596 | 0.0093 | 0.02765 |
| rs11081529 | SCZ | C | T | 0.278 | 0.01 | 0.0097 | 0.2992 |
| rs11099020 | SCZ | T | C | 0.634 | -0.007899 | 0.009 | 0.3785 |
| rs11134679 | SCZ | G | A | 0.679 | -0.002297 | 0.0092 | 0.8045 |
| rs11165643 | SCZ | T | C | 0.577 | -0.013903 | 0.0087 | 0.1101 |
| rs11250094 | SCZ | C | G | 0.545 | -0.015302 | 0.0087 | 0.077951 |
| rs112633616 | SCZ | C | A | 0.032 | -0.078497 | 0.0263 | 0.002828 |
| rs112687095 | SCZ | A | G | 0.166 | 0.008799 | 0.0124 | 0.4793 |
| rs113338260 | SCZ | C | T | 0.211 | 0.042198 | 0.0122 | 0.000541 |
| rs113520408 | SCZ | A | G | 0.27 | -0.0278 | 0.0098 | 0.00456 |
| rs113624107 | SCZ | A | G | 0.223 | -0.008603 | 0.0105 | 0.4114 |
| rs1143770 | SCZ | T | C | 0.584 | -0.007899 | 0.0088 | 0.3706 |
| rs115000530 | SCZ | T | A | 0.053 | -0.001898 | 0.0199 | 0.9222 |
| rs115454970 | SCZ | T | G | 0.267 | 0.009101 | 0.01 | 0.3635 |
| rs11587347 | SCZ | G | C | 0.094 | 0.103895 | 0.0147 | 1.53E-12 |
| rs11620355 | SCZ | A | G | 0.092 | -0.032099 | 0.0153 | 0.03579 |
| rs11635092 | SCZ | A | G | 0.356 | 0.003105 | 0.0093 | 0.741899 |
| rs116377258 | SCZ | G | A | 0.031 | 0.078502 | 0.0249 | 0.001603 |
| rs11693094 | SCZ | T | C | 0.461 | -0.054403 | 0.0087 | 4.29E-10 |
| rs11696755 | SCZ | C | T | 0.184 | 0.063696 | 0.011 | 7.26E-09 |
| rs11709402 | SCZ | G | A | 0.268 | -0.014701 | 0.0097 | 0.1327 |
| rs117118217 | SCZ | C | G | 0.018 | 0.037297 | 0.0398 | 0.3485 |
| rs11732657 | SCZ | A | G | 0.747 | -0.009505 | 0.01 | 0.3401 |
| rs118136827 | SCZ | T | G | 0.277 | 0.014403 | 0.0097 | 0.1377 |
| rs11866420 | SCZ | G | C | 0.607 | 0.030397 | 0.009 | 0.000748 |
| rs1198588 | SCZ | T | A | 0.788 | 0.102598 | 0.0108 | 1.73E-21 |
| rs12001437 | SCZ | C | T | 0.374 | -0.003404 | 0.0089 | 0.7036 |
| rs12033257 | SCZ | G | A | 0.384 | -0.023902 | 0.0092 | 0.009292 |
| rs12140153 | SCZ | T | G | 0.091 | -0.018704 | 0.0156 | 0.2318 |
| rs12149660 | SCZ | A | G | 0.121 | 0.013602 | 0.0137 | 0.3203 |
| rs12156160 | SCZ | G | A | 0.142 | -0.031101 | 0.0135 | 0.0213 |
| rs12204714 | SCZ | T | C | 0.654 | 0.005002 | 0.0091 | 0.5815 |
| rs12293670 | SCZ | G | A | 0.34 | -0.070496 | 0.0092 | 1.56E-14 |
| rs12303743 | SCZ | C | G | 0.094 | 0.087499 | 0.0145 | 1.59E-09 |
| rs12364470 | SCZ | G | T | 0.168 | -0.042197 | 0.0119 | 0.000388 |
| rs12375949 | SCZ | C | T | 0.575 | -0.001699 | 0.0087 | 0.8463 |
| rs12375985 | SCZ | A | G | 0.347 | 0.005596 | 0.0091 | 0.5352 |
| rs12427047 | SCZ | T | C | 0.255 | -0.009802 | 0.0099 | 0.3246 |
| rs12519073 | SCZ | T | C | 0.237 | -0.002397 | 0.0102 | 0.8116 |
| rs12523398 | SCZ | A | T | 0.172 | 0.0455 | 0.0115 | 7.30E-05 |
| rs12643771 | SCZ | T | C | 0.31 | 0.032895 | 0.0094 | 0.000487 |
| rs1266874 | SCZ | G | A | 0.36 | -0.0063 | 0.009 | 0.4826 |
| rs12681792 | SCZ | A | C | 0.197 | -0.011296 | 0.011 | 0.3022 |
| rs12682775 | SCZ | C | T | 0.224 | -0.002497 | 0.0103 | 0.8094 |
| rs12712510 | SCZ | C | T | 0.527 | -0.057401 | 0.0087 | 5.14E-11 |
| rs12714592 | SCZ | C | A | 0.277 | 0.011597 | 0.0096 | 0.2273 |
| rs12714702 | SCZ | G | A | 0.847 | 0.025 | 0.0121 | 0.03948 |
| rs12757779 | SCZ | A | G | 0.209 | 0.025297 | 0.0106 | 0.01679 |
| rs12762034 | SCZ | C | T | 0.081 | -0.076498 | 0.0162 | 2.29E-06 |
| rs1286058 | SCZ | A | T | 0.712 | -0.016798 | 0.0095 | 0.076849 |
| rs12881629 | SCZ | G | A | 0.08 | 0.004098 | 0.0162 | 0.802 |
| rs12907546 | SCZ | A | G | 0.216 | 0.019805 | 0.0104 | 0.05774 |
| rs12919291 | SCZ | C | G | 0.194 | 0.025205 | 0.0109 | 0.02048 |
| rs12940014 | SCZ | C | T | 0.513 | -0.024 | 0.0086 | 0.005289 |
| rs12955211 | SCZ | A | T | 0.316 | 0.007095 | 0.0093 | 0.4465 |
| rs12956148 | SCZ | A | C | 0.304 | 0.017899 | 0.0094 | 0.05571 |
| rs1296328 | SCZ | C | A | 0.551 | -0.012304 | 0.0088 | 0.161 |
| rs12977787 | SCZ | A | G | 0.529 | 0.011 | 0.009 | 0.2182 |
| rs13030994 | SCZ | A | G | 0.485 | -0.032903 | 0.0086 | 0.000135 |
| rs13037326 | SCZ | T | C | 0.263 | 0.043398 | 0.0097 | 8.44E-06 |
| rs13090388 | SCZ | T | C | 0.317 | 0.020397 | 0.0093 | 0.02775 |
| rs13107325 | SCZ | T | C | 0.067 | 0.158703 | 0.0168 | 2.90E-21 |
| rs13141210 | SCZ | T | C | 0.506 | -0.014504 | 0.0087 | 0.094591 |
| rs13145650 | SCZ | T | C | 0.9183 | -0.047999 | 0.0157 | 0.002229 |
| rs13175535 | SCZ | A | G | 0.302 | 0.006501 | 0.0096 | 0.4984 |
| rs13195636 | SCZ | C | A | 0.091 | -0.210504 | 0.0159 | 6.55E-40 |
| rs1320251 | SCZ | T | C | 0.47 | -0.008097 | 0.0087 | 0.3521 |
| rs13218383 | SCZ | G | C | 0.345 | 0.007397 | 0.009 | 0.4135 |
| rs13233308 | SCZ | T | C | 0.484 | -0.048704 | 0.0086 | 1.75E-08 |
| rs13248187 | SCZ | C | T | 0.267 | -0.015696 | 0.0098 | 0.1102 |
| rs13261666 | SCZ | T | G | 0.522 | -0.016503 | 0.0086 | 0.05544 |
| rs1327259 | SCZ | G | A | 0.402 | 0.0001 | 0.0088 | 0.9902 |
| rs13292699 | SCZ | C | A | 0.428 | 0.001701 | 0.0087 | 0.8491 |
| rs1330199 | SCZ | T | G | 0.484 | -0.015302 | 0.0086 | 0.077441 |
| rs13307225 | SCZ | A | G | 0.9046 | -0.036997 | 0.0149 | 0.01278 |
| rs13317303 | SCZ | A | C | 0.143 | 0.012002 | 0.0123 | 0.3306 |
| rs1334297 | SCZ | A | G | 0.737 | -0.002297 | 0.0098 | 0.8145 |
| rs13422673 | SCZ | T | C | 0.466 | 0.001902 | 0.0086 | 0.8244 |
| rs13427822 | SCZ | G | A | 0.274 | 0.041395 | 0.0097 | 2.00E-05 |
| rs1346841 | SCZ | A | G | 0.399 | 0.030098 | 0.0088 | 0.000594 |
| rs1360201 | SCZ | T | C | 0.482 | -0.014297 | 0.0086 | 0.09849 |
| rs1363862 | SCZ | A | G | 0.272 | -0.024702 | 0.0097 | 0.01094 |
| rs13642 | SCZ | T | A | 0.359 | 0.032399 | 0.0089 | 0.000287 |
| rs1391438 | SCZ | C | T | 0.668 | -0.018596 | 0.0091 | 0.04164 |
| rs1392816 | SCZ | T | C | 0.381 | -0.018498 | 0.0089 | 0.03824 |
| rs140159717 | SCZ | T | C | 0.082 | 0.046997 | 0.017 | 0.005682 |
| rs1438945 | SCZ | A | T | 0.721 | 0.024395 | 0.0097 | 0.01214 |
| rs1441264 | SCZ | A | G | 0.608 | 0.008395 | 0.0089 | 0.3483 |
| rs1450782 | SCZ | G | T | 0.577 | 0.008698 | 0.0088 | 0.3234 |
| rs1455350 | SCZ | A | T | 0.491 | 0.015205 | 0.0086 | 0.07773 |
| rs1458156 | SCZ | T | C | 0.484 | -0.015401 | 0.0086 | 0.073529 |
| rs1471740 | SCZ | C | T | 0.75 | -0.042197 | 0.0099 | 2.07E-05 |
| rs147568678 | SCZ | C | T | 0.233 | 0.008799 | 0.0104 | 0.3967 |
| rs1477290 | SCZ | C | T | 0.141 | 0.023903 | 0.0123 | 0.05157 |
| rs1503526 | SCZ | C | T | 0.492 | -0.020704 | 0.0086 | 0.01652 |
| rs152603 | SCZ | G | A | 0.356 | 0.030995 | 0.009 | 0.000569 |
| rs1565735 | SCZ | A | T | 0.2 | -0.0718 | 0.0109 | 4.83E-11 |
| rs1566085 | SCZ | T | G | 0.549 | 0.017696 | 0.0087 | 0.04287 |
| rs1582931 | SCZ | A | G | 0.48 | 0.004601 | 0.0087 | 0.596401 |
| rs1584469 | SCZ | T | C | 0.297 | 0.003105 | 0.0095 | 0.741199 |
| rs1609010 | SCZ | G | A | 0.555 | -0.010604 | 0.0087 | 0.2215 |
| rs1671770 | SCZ | C | A | 0.825 | 0.015703 | 0.0114 | 0.1681 |
| rs16846140 | SCZ | G | A | 0.337 | -0.035599 | 0.0092 | 0.000101 |
| rs16846463 | SCZ | G | A | 0.099 | 0.006501 | 0.0144 | 0.6536 |
| rs16854920 | SCZ | C | T | 0.336 | 0.026303 | 0.0093 | 0.004471 |
| rs1689510 | SCZ | C | G | 0.327 | -0.006201 | 0.0092 | 0.502 |
| rs16916303 | SCZ | G | A | 0.123 | 0.006099 | 0.0134 | 0.6488 |
| rs16995054 | SCZ | T | C | 0.204 | 0.006099 | 0.0106 | 0.5668 |
| rs17193211 | SCZ | T | C | 0.07 | -0.002597 | 0.0176 | 0.8825 |
| rs17194490 | SCZ | T | G | 0.161 | 0.078199 | 0.0116 | 1.80E-11 |
| rs17399739 | SCZ | G | A | 0.064 | 0.001701 | 0.0177 | 0.9238 |
| rs17565975 | SCZ | A | G | 0.549 | 0.004199 | 0.0087 | 0.6313 |
| rs17598675 | SCZ | C | T | 0.488 | 0.005002 | 0.0086 | 0.5603 |
| rs176218 | SCZ | T | G | 0.2 | -0.023599 | 0.0109 | 0.02999 |
| rs1778830 | SCZ | A | G | 0.361 | -0.016198 | 0.009 | 0.07131 |
| rs1788808 | SCZ | G | A | 0.483 | -0.001998 | 0.0087 | 0.8169 |
| rs1834144 | SCZ | A | C | 0.385 | 0.005596 | 0.0089 | 0.5283 |
| rs1860002 | SCZ | T | C | 0.543 | -0.083799 | 0.0087 | 1.04E-21 |
| rs1899896 | SCZ | T | C | 0.298 | 0.021397 | 0.0094 | 0.02325 |
| rs1901512 | SCZ | C | T | 0.699 | -0.058401 | 0.0094 | 5.72E-10 |
| rs1915019 | SCZ | G | A | 0.75 | -0.057098 | 0.0098 | 6.57E-09 |
| rs1919243 | SCZ | C | T | 0.484 | 0.011101 | 0.0087 | 0.2013 |
| rs1950829 | SCZ | G | A | 0.506 | -0.017899 | 0.0086 | 0.03798 |
| rs1967772 | SCZ | A | G | 0.273 | 0.023596 | 0.0097 | 0.01453 |
| rs2035936 | SCZ | T | G | 0.054 | -0.014603 | 0.0195 | 0.453799 |
| rs2076603 | SCZ | A | G | 0.623 | -0.0109 | 0.0089 | 0.2222 |
| rs2084572 | SCZ | G | A | 0.448 | -0.045298 | 0.0086 | 1.66E-07 |
| rs2102278 | SCZ | G | A | 0.31 | -0.010999 | 0.0095 | 0.2474 |
| rs2133561 | SCZ | T | A | 0.609 | -0.033203 | 0.009 | 0.000239 |
| rs213518 | SCZ | C | T | 0.141 | -0.009802 | 0.0124 | 0.4296 |
| rs2153740 | SCZ | G | A | 0.484 | 0.015002 | 0.0086 | 0.083311 |
| rs215634 | SCZ | G | A | 0.622 | -0.010099 | 0.0088 | 0.2514 |
| rs2172131 | SCZ | C | T | 0.562 | 0.0185 | 0.0088 | 0.03491 |
| rs217336 | SCZ | A | C | 0.434 | -0.050303 | 0.0087 | 8.05E-09 |
| rs2174752 | SCZ | T | G | 0.458 | 0.0001 | 0.0086 | 0.993 |
| rs2176337 | SCZ | T | A | 0.33 | 0.012295 | 0.0092 | 0.1819 |
| rs217672 | SCZ | C | A | 0.262 | -0.023502 | 0.0098 | 0.01687 |
| rs2214123 | SCZ | G | A | 0.638 | -0.006995 | 0.0091 | 0.4386 |
| rs2234458 | SCZ | T | C | 0.653 | -0.0161 | 0.0091 | 0.07576 |
| rs2253310 | SCZ | G | C | 0.616 | -0.021302 | 0.0088 | 0.01618 |
| rs2281819 | SCZ | A | T | 0.234 | 0.036695 | 0.0101 | 0.000284 |
| rs2283076 | SCZ | G | A | 0.213 | 0.023801 | 0.0105 | 0.02386 |
| rs2289379 | SCZ | T | C | 0.404 | -0.003902 | 0.0088 | 0.6608 |
| rs2302761 | SCZ | T | C | 0.208 | 0.0063 | 0.0106 | 0.5512 |
| rs2306593 | SCZ | T | C | 0.493 | 0.0382 | 0.0086 | 9.99E-06 |
| rs2307111 | SCZ | C | T | 0.402 | 0.012103 | 0.0088 | 0.1665 |
| rs2332700 | SCZ | G | C | 0.753 | -0.075098 | 0.0099 | 3.88E-14 |
| rs2333321 | SCZ | G | A | 0.792 | -0.071204 | 0.0105 | 1.25E-11 |
| rs2342892 | SCZ | G | T | 0.523 | 0.005002 | 0.0086 | 0.5635 |
| rs2347526 | SCZ | C | T | 0.666 | 0.016302 | 0.0091 | 0.074059 |
| rs2396625 | SCZ | A | T | 0.426 | 0.010596 | 0.0088 | 0.2269 |
| rs2398861 | SCZ | G | A | 0.264 | 0.023402 | 0.0098 | 0.01654 |
| rs240963 | SCZ | C | T | 0.836 | 0.029295 | 0.0117 | 0.01261 |
| rs242093 | SCZ | A | G | 0.586 | 0.0045 | 0.0088 | 0.610401 |
| rs2433733 | SCZ | A | G | 0.663 | 0.027803 | 0.0091 | 0.002344 |
| rs2456020 | SCZ | T | C | 0.241 | -0.081598 | 0.0102 | 1.13E-15 |
| rs2482356 | SCZ | C | T | 0.418 | -0.010505 | 0.0087 | 0.2282 |
| rs252761 | SCZ | T | G | 0.589 | 0.003295 | 0.009 | 0.716699 |
| rs2554835 | SCZ | A | G | 0.402 | -0.015401 | 0.0091 | 0.0926 |
| rs2568958 | SCZ | A | G | 0.618 | 0.022399 | 0.0089 | 0.01179 |
| rs2606228 | SCZ | C | A | 0.656 | 0.002102 | 0.0092 | 0.8189 |
| rs2612030 | SCZ | C | T | 0.852 | 0.0142 | 0.0122 | 0.2424 |
| rs2616143 | SCZ | A | G | 0.318 | -0.006896 | 0.0093 | 0.4578 |
| rs266047 | SCZ | A | G | 0.535 | 0.0009 | 0.0086 | 0.9187 |
| rs2678204 | SCZ | G | T | 0.326 | 0.013805 | 0.0092 | 0.1328 |
| rs2781668 | SCZ | T | C | 0.166 | 0.014698 | 0.0117 | 0.2094 |
| rs2787101 | SCZ | T | C | 0.604 | -0.041497 | 0.0088 | 2.61E-06 |
| rs2819336 | SCZ | C | T | 0.644 | 0.037505 | 0.009 | 3.37E-05 |
| rs28373063 | SCZ | C | G | 0.17 | -0.006499 | 0.012 | 0.5877 |
| rs28404639 | SCZ | T | C | 0.349 | -0.002397 | 0.0091 | 0.786799 |
| rs28489620 | SCZ | A | G | 0.289 | -0.005196 | 0.0098 | 0.5962 |
| rs28513670 | SCZ | G | A | 0.18 | 0.024897 | 0.0112 | 0.02643 |
| rs2875762 | SCZ | C | G | 0.236 | -0.008097 | 0.0103 | 0.4312 |
| rs2876520 | SCZ | G | C | 0.473 | 0.015205 | 0.0088 | 0.08209 |
| rs2920503 | SCZ | T | C | 0.284 | -0.013903 | 0.0097 | 0.1525 |
| rs2923431 | SCZ | C | G | 0.627 | 0.014099 | 0.009 | 0.1165 |
| rs293566 | SCZ | C | T | 0.355 | -0.012897 | 0.0091 | 0.1576 |
| rs2962334 | SCZ | T | G | 0.023 | 0.028502 | 0.0292 | 0.3282 |
| rs2971970 | SCZ | G | T | 0.791 | -0.011504 | 0.0106 | 0.2766 |
| rs301800 | SCZ | C | T | 0.817 | 0.029903 | 0.0112 | 0.007675 |
| rs30266 | SCZ | A | G | 0.323 | 0.026098 | 0.0092 | 0.00443 |
| rs317656 | SCZ | A | T | 0.713 | 0.017502 | 0.0096 | 0.06749 |
| rs320693 | SCZ | C | G | 0.459 | 0.041395 | 0.0086 | 1.57E-06 |
| rs3211995 | SCZ | A | G | 0.162 | -0.0109 | 0.0121 | 0.3647 |
| rs329118 | SCZ | T | C | 0.426 | 0.008799 | 0.0087 | 0.3084 |
| rs34025316 | SCZ | T | C | 0.349 | -0.025405 | 0.0093 | 0.006007 |
| rs34045288 | SCZ | T | C | 0.327 | 0.010404 | 0.0092 | 0.2571 |
| rs34234296 | SCZ | A | G | 0.378 | -0.003703 | 0.0092 | 0.6857 |
| rs34481751 | SCZ | A | C | 0.166 | -0.009802 | 0.012 | 0.4122 |
| rs34517439 | SCZ | A | C | 0.109 | -0.040095 | 0.0149 | 0.007166 |
| rs347551 | SCZ | G | C | 0.481 | -0.025297 | 0.009 | 0.004767 |
| rs34811474 | SCZ | A | G | 0.219 | -0.027897 | 0.0114 | 0.01446 |
| rs35154326 | SCZ | G | A | 0.281 | 0.019703 | 0.0097 | 0.04234 |
| rs35309068 | SCZ | G | T | 0.456 | 0.013896 | 0.0087 | 0.1108 |
| rs354155 | SCZ | C | G | 0.104 | 0.002403 | 0.0142 | 0.8665 |
| rs35417702 | SCZ | T | C | 0.522 | -0.047799 | 0.0086 | 2.78E-08 |
| rs35475880 | SCZ | T | G | 0.206 | -0.021801 | 0.0109 | 0.04422 |
| rs35532491 | SCZ | T | A | 0.097 | -0.006399 | 0.0152 | 0.670601 |
| rs355777 | SCZ | C | G | 0.414 | 0.013501 | 0.0087 | 0.1215 |
| rs35867081 | SCZ | G | A | 0.511 | 0.0003 | 0.0089 | 0.9756 |
| rs36007635 | SCZ | A | G | 0.134 | -0.005196 | 0.0127 | 0.6791 |
| rs36061954 | SCZ | T | C | 0.393 | 0.029995 | 0.0088 | 0.000679 |
| rs36119825 | SCZ | A | G | 0.447 | 0.014505 | 0.0086 | 0.092611 |
| rs363096 | SCZ | C | T | 0.581 | -0.016297 | 0.0087 | 0.0622 |
| rs3747631 | SCZ | C | G | 0.212 | 0.037899 | 0.0104 | 0.000285 |
| rs3764625 | SCZ | G | T | 0.585 | 0.028502 | 0.0088 | 0.001212 |
| rs3770754 | SCZ | G | C | 0.368 | -0.052896 | 0.0091 | 5.35E-09 |
| rs3784710 | SCZ | C | T | 0.225 | -0.023101 | 0.0104 | 0.02562 |
| rs3800546 | SCZ | G | C | 0.271 | 0.014302 | 0.0097 | 0.1396 |
| rs3807865 | SCZ | A | G | 0.407 | 0.016201 | 0.0088 | 0.065671 |
| rs3809634 | SCZ | G | A | 0.317 | -0.002896 | 0.0092 | 0.752501 |
| rs3814883 | SCZ | T | C | 0.467 | -0.067098 | 0.0087 | 1.58E-14 |
| rs3845344 | SCZ | T | C | 0.385 | 0.013704 | 0.0089 | 0.122 |
| rs3851998 | SCZ | G | C | 0.748 | 0.0142 | 0.01 | 0.1552 |
| rs3896224 | SCZ | G | A | 0.428 | -0.027401 | 0.0087 | 0.001697 |
| rs3897821 | SCZ | G | A | 0.334 | -0.0435 | 0.0092 | 2.20E-06 |
| rs3901286 | SCZ | A | C | 0.157 | -0.017005 | 0.0119 | 0.1525 |
| rs394608 | SCZ | C | T | 0.533 | -1.00E-04 | 0.0086 | 0.9928 |
| rs40071 | SCZ | C | T | 0.179 | -0.003095 | 0.0112 | 0.783401 |
| rs4044321 | SCZ | G | A | 0.644 | 0.011101 | 0.009 | 0.2168 |
| rs4055791 | SCZ | T | C | 0.411 | 0.006904 | 0.0087 | 0.4333 |
| rs406388 | SCZ | G | C | 0.18 | -0.021703 | 0.0115 | 0.058621 |
| rs4148155 | SCZ | G | A | 0.106 | -0.0089 | 0.0141 | 0.5291 |
| rs4261944 | SCZ | G | T | 0.363 | 0.002603 | 0.009 | 0.7696 |
| rs4267103 | SCZ | C | T | 0.178 | -0.004301 | 0.0113 | 0.7059 |
| rs429343 | SCZ | G | A | 0.574 | 0.0262 | 0.0087 | 0.002653 |
| rs429358 | SCZ | C | T | 0.149 | 0.013501 | 0.0124 | 0.2768 |
| rs4328757 | SCZ | T | C | 0.605 | 0.029202 | 0.0089 | 0.000986 |
| rs4352658 | SCZ | T | C | 0.08 | -0.009098 | 0.0159 | 0.565999 |
| rs4382592 | SCZ | G | T | 0.693 | -0.0002 | 0.0093 | 0.9805 |
| rs4419475 | SCZ | T | A | 0.411 | 0.001501 | 0.0088 | 0.8613 |
| rs4439537 | SCZ | C | T | 0.522 | 0.008698 | 0.0086 | 0.3135 |
| rs4477562 | SCZ | T | C | 0.128 | -0.002397 | 0.0129 | 0.8553 |
| rs4482463 | SCZ | A | C | 0.9205 | -0.015903 | 0.0161 | 0.3238 |
| rs4613074 | SCZ | C | T | 0.182 | 0.020805 | 0.0111 | 0.06142 |
| rs4648450 | SCZ | A | C | 0.474 | -0.005704 | 0.0088 | 0.5197 |
| rs4653164 | SCZ | T | C | 0.668 | 0.051104 | 0.0092 | 3.08E-08 |
| rs4671328 | SCZ | G | T | 0.555 | 0.028101 | 0.0087 | 0.00123 |
| rs4700393 | SCZ | G | A | 0.529 | -0.040499 | 0.0086 | 2.42E-06 |
| rs4702 | SCZ | A | G | 0.56 | -0.084304 | 0.0089 | 2.79E-21 |
| rs4709807 | SCZ | C | T | 0.768 | 0.029604 | 0.0103 | 0.003898 |
| rs4737188 | SCZ | T | A | 0.48 | 0.030903 | 0.0086 | 0.000346 |
| rs4757144 | SCZ | A | G | 0.578 | -0.017702 | 0.0087 | 0.04207 |
| rs4757957 | SCZ | C | G | 0.695 | 0.009697 | 0.0094 | 0.2994 |
| rs4764949 | SCZ | G | A | 0.334 | 0.027104 | 0.0092 | 0.003091 |
| rs4790292 | SCZ | A | C | 0.154 | 0.040697 | 0.0123 | 0.000982 |
| rs4810227 | SCZ | A | G | 0.615 | -0.008405 | 0.0089 | 0.3471 |
| rs4812325 | SCZ | A | G | 0.616 | 0.071904 | 0.0089 | 8.96E-16 |
| rs4832298 | SCZ | T | C | 0.698 | 0.009202 | 0.0094 | 0.3268 |
| rs4846724 | SCZ | A | G | 0.532 | 0.013896 | 0.0086 | 0.1069 |
| rs4876611 | SCZ | G | A | 0.729 | -0.020998 | 0.0097 | 0.03038 |
| rs4895799 | SCZ | T | C | 0.573 | 0.021101 | 0.0088 | 0.01627 |
| rs4958702 | SCZ | C | T | 0.571 | 0.032895 | 0.0087 | 0.00016 |
| rs4961705 | SCZ | C | G | 0.346 | 0.0064 | 0.0091 | 0.4791 |
| rs512121 | SCZ | C | T | 0.199 | 0.009202 | 0.0108 | 0.3952 |
| rs539515 | SCZ | C | A | 0.194 | -0.0007 | 0.0109 | 0.9464 |
| rs55658481 | SCZ | A | G | 0.334 | 0.008496 | 0.0092 | 0.3562 |
| rs55707359 | SCZ | G | T | 0.017 | 0.001601 | 0.0414 | 0.969 |
| rs55714539 | SCZ | C | A | 0.348 | -0.001699 | 0.0093 | 0.8543 |
| rs55726687 | SCZ | A | G | 0.21 | 0.022297 | 0.0107 | 0.03675 |
| rs55736314 | SCZ | G | C | 0.392 | -0.037402 | 0.0089 | 2.60E-05 |
| rs558887 | SCZ | G | A | 0.304 | -0.015903 | 0.0095 | 0.094591 |
| rs56094641 | SCZ | G | A | 0.415 | -0.002996 | 0.0087 | 0.727301 |
| rs56133507 | SCZ | G | T | 0.19 | -0.019999 | 0.0111 | 0.07233 |
| rs56161855 | SCZ | T | A | 0.142 | -0.002198 | 0.0125 | 0.8584 |
| rs56203622 | SCZ | C | T | 0.151 | -0.004102 | 0.0122 | 0.734199 |
| rs56335113 | SCZ | G | A | 0.697 | -0.064701 | 0.0094 | 6.02E-12 |
| rs56356382 | SCZ | C | T | 0.191 | -0.027002 | 0.0117 | 0.02114 |
| rs56391344 | SCZ | A | G | 0.247 | -0.003095 | 0.0101 | 0.7573 |
| rs56399737 | SCZ | T | C | 0.449 | 0.0008 | 0.0087 | 0.9244 |
| rs56858768 | SCZ | A | G | 0.293 | 0.007498 | 0.0095 | 0.4296 |
| rs57636386 | SCZ | C | T | 0.086 | -0.021595 | 0.0157 | 0.1674 |
| rs58120505 | SCZ | C | T | 0.418 | -0.089603 | 0.0088 | 2.24E-24 |
| rs59086897 | SCZ | A | T | 0.475 | -0.003803 | 0.0086 | 0.6569 |
| rs59237168 | SCZ | C | T | 0.221 | 0.004701 | 0.0104 | 0.649101 |
| rs5995843 | SCZ | G | A | 0.345 | -0.010198 | 0.0091 | 0.2628 |
| rs60764613 | SCZ | T | G | 0.151 | 0.002704 | 0.0121 | 0.8267 |
| rs6123924 | SCZ | G | A | 0.161 | 0.009 | 0.0119 | 0.4485 |
| rs6134916 | SCZ | T | C | 0.491 | -0.027897 | 0.0086 | 0.00124 |
| rs61813324 | SCZ | T | C | 0.138 | -0.026798 | 0.0136 | 0.04876 |
| rs61828088 | SCZ | A | G | 0.111 | -0.050303 | 0.0138 | 0.00027 |
| rs61903695 | SCZ | G | A | 0.249 | 0 | 0.01 | 0.9994 |
| rs61914045 | SCZ | A | G | 0.205 | 0.022205 | 0.0107 | 0.03751 |
| rs62097985 | SCZ | T | C | 0.425 | 0.037795 | 0.0087 | 1.51E-05 |
| rs62107261 | SCZ | C | T | 0.043 | -0.017496 | 0.0229 | 0.4441 |
| rs62134195 | SCZ | T | C | 0.04 | 0.012903 | 0.0231 | 0.5761 |
| rs62176243 | SCZ | T | A | 0.25 | 0.008304 | 0.01 | 0.408 |
| rs62176993 | SCZ | A | G | 0.391 | 0.013095 | 0.0089 | 0.1402 |
| rs62190049 | SCZ | C | G | 0.39 | -0.012097 | 0.0091 | 0.1818 |
| rs62439690 | SCZ | A | G | 0.253 | -0.004997 | 0.01 | 0.621001 |
| rs6265 | SCZ | T | C | 0.191 | -0.047198 | 0.011 | 1.87E-05 |
| rs6531639 | SCZ | A | G | 0.231 | 0.005304 | 0.0107 | 0.616 |
| rs6545714 | SCZ | A | G | 0.61 | -0.005196 | 0.0088 | 0.5517 |
| rs6546857 | SCZ | G | A | 0.232 | 0.060398 | 0.0102 | 2.74E-09 |
| rs6560906 | SCZ | C | T | 0.693 | -0.003703 | 0.0094 | 0.6889 |
| rs6567160 | SCZ | C | T | 0.234 | 0.012599 | 0.0102 | 0.2156 |
| rs6575340 | SCZ | A | G | 0.639 | -0.019597 | 0.009 | 0.02895 |
| rs66511648 | SCZ | C | T | 0.276 | -0.0005 | 0.0097 | 0.9565 |
| rs66679256 | SCZ | T | C | 0.445 | -0.002696 | 0.0087 | 0.7558 |
| rs6669341 | SCZ | G | A | 0.598 | 0.007901 | 0.0088 | 0.3675 |
| rs6682438 | SCZ | C | T | 0.671 | -0.007998 | 0.0092 | 0.3819 |
| rs6688826 | SCZ | C | T | 0.279 | 0.011496 | 0.0096 | 0.2286 |
| rs6707827 | SCZ | G | A | 0.706 | 0.013095 | 0.0096 | 0.1731 |
| rs6710091 | SCZ | G | C | 0.349 | 0.0078 | 0.009 | 0.3878 |
| rs6719762 | SCZ | C | T | 0.482 | 0.0142 | 0.0086 | 0.1001 |
| rs6731967 | SCZ | C | G | 0.243 | 0.022103 | 0.0101 | 0.02845 |
| rs6744646 | SCZ | G | A | 0.825 | 0.031305 | 0.0113 | 0.005747 |
| rs6744794 | SCZ | G | C | 0.621 | 0.0127 | 0.0089 | 0.1537 |
| rs6752979 | SCZ | A | G | 0.315 | -0.019705 | 0.0093 | 0.03416 |
| rs6774894 | SCZ | A | T | 0.354 | -0.026798 | 0.009 | 0.002966 |
| rs6798742 | SCZ | G | A | 0.307 | 0.061099 | 0.0093 | 4.57E-11 |
| rs6803651 | SCZ | T | G | 0.43 | -0.009703 | 0.0088 | 0.2748 |
| rs6805241 | SCZ | C | T | 0.221 | -0.008296 | 0.0104 | 0.4243 |
| rs6843852 | SCZ | T | C | 0.529 | 0.005998 | 0.0087 | 0.4935 |
| rs6943762 | SCZ | C | T | 0.13 | -0.105098 | 0.0132 | 1.57E-15 |
| rs6959891 | SCZ | G | A | 0.284 | 0.005002 | 0.0096 | 0.6037 |
| rs6962980 | SCZ | C | A | 0.544 | -0.031101 | 0.0087 | 0.000351 |
| rs6974218 | SCZ | C | A | 0.382 | -0.054895 | 0.0089 | 6.80E-10 |
| rs698147 | SCZ | G | A | 0.534 | 0.0064 | 0.0086 | 0.4586 |
| rs7008955 | SCZ | G | T | 0.525 | -0.020303 | 0.0086 | 0.01855 |
| rs7012546 | SCZ | T | C | 0.413 | -0.006698 | 0.0088 | 0.4416 |
| rs7029718 | SCZ | A | G | 0.404 | -0.018203 | 0.0088 | 0.03921 |
| rs7031698 | SCZ | C | T | 0.781 | -0.006399 | 0.0105 | 0.543501 |
| rs7034554 | SCZ | G | A | 0.364 | 0.042396 | 0.009 | 2.37E-06 |
| rs7038943 | SCZ | C | T | 0.36 | 0.019999 | 0.009 | 0.02609 |
| rs7079070 | SCZ | A | G | 0.44 | 0.020397 | 0.0088 | 0.02008 |
| rs708228 | SCZ | T | C | 0.329 | 0.0528 | 0.0091 | 6.56E-09 |
| rs7124681 | SCZ | A | C | 0.409 | -0.0004 | 0.0088 | 0.9594 |
| rs7132908 | SCZ | A | G | 0.396 | -0.005296 | 0.0088 | 0.5456 |
| rs71646142 | SCZ | T | C | 0.188 | -0.017801 | 0.0111 | 0.1077 |
| rs7201895 | SCZ | A | G | 0.346 | 0.001902 | 0.0091 | 0.8339 |
| rs7206608 | SCZ | G | C | 0.316 | -0.021399 | 0.0093 | 0.02139 |
| rs7218014 | SCZ | C | T | 0.207 | 0.008698 | 0.0107 | 0.4177 |
| rs7233920 | SCZ | A | G | 0.229 | 0.008103 | 0.0103 | 0.4294 |
| rs7236339 | SCZ | A | G | 0.213 | 0.068097 | 0.0104 | 6.38E-11 |
| rs723672 | SCZ | T | C | 0.436 | -0.006797 | 0.0088 | 0.4408 |
| rs7250833 | SCZ | T | C | 0.284 | -0.019401 | 0.0096 | 0.04252 |
| rs7259070 | SCZ | C | T | 0.592 | -0.026204 | 0.0094 | 0.005599 |
| rs72673947 | SCZ | G | A | 0.114 | -0.001798 | 0.0136 | 0.8927 |
| rs7278859 | SCZ | T | A | 0.314 | 0.008002 | 0.0093 | 0.3899 |
| rs72887338 | SCZ | C | T | 0.378 | -0.024302 | 0.009 | 0.006826 |
| rs72892910 | SCZ | T | G | 0.181 | -0.011998 | 0.0112 | 0.2851 |
| rs72910629 | SCZ | G | A | 0.134 | 0.021898 | 0.0127 | 0.08493 |
| rs72986630 | SCZ | T | C | 0.068 | 0.112296 | 0.0179 | 3.59E-10 |
| rs73026725 | SCZ | A | C | 0.145 | 0.056401 | 0.0122 | 3.88E-06 |
| rs730384 | SCZ | A | G | 0.44 | -0.0223 | 0.0087 | 0.0105 |
| rs73052033 | SCZ | C | T | 0.177 | -0.019705 | 0.0113 | 0.081031 |
| rs73142879 | SCZ | T | C | 0.187 | -0.002397 | 0.0111 | 0.8316 |
| rs73213484 | SCZ | T | A | 0.153 | -0.039298 | 0.0121 | 0.001167 |
| rs7331420 | SCZ | A | G | 0.295 | 0.013197 | 0.0095 | 0.165 |
| rs7332724 | SCZ | T | C | 0.28 | 0.0277 | 0.0096 | 0.003846 |
| rs73344830 | SCZ | G | A | 0.593 | -0.009604 | 0.0089 | 0.2781 |
| rs7357754 | SCZ | G | A | 0.501 | -0.010703 | 0.0087 | 0.2162 |
| rs7359501 | SCZ | T | C | 0.394 | -0.009604 | 0.0088 | 0.2756 |
| rs736282 | SCZ | C | T | 0.533 | -0.006002 | 0.0086 | 0.4853 |
| rs7442137 | SCZ | T | C | 0.642 | -0.013203 | 0.009 | 0.1428 |
| rs7442885 | SCZ | G | C | 0.222 | 0.029697 | 0.0104 | 0.004179 |
| rs745249 | SCZ | T | C | 0.28 | 0.003898 | 0.0096 | 0.683701 |
| rs7498044 | SCZ | A | G | 0.213 | -0.009396 | 0.0108 | 0.3835 |
| rs7498665 | SCZ | G | A | 0.387 | 0.023699 | 0.0089 | 0.007378 |
| rs75035127 | SCZ | G | A | 0.029 | 0.034602 | 0.0256 | 0.1756 |
| rs7551758 | SCZ | G | T | 0.535 | 0.003105 | 0.0087 | 0.7191 |
| rs7575189 | SCZ | A | G | 0.581 | -0.031402 | 0.0087 | 0.000316 |
| rs7594904 | SCZ | C | T | 0.417 | -0.002996 | 0.0088 | 0.730099 |
| rs76076331 | SCZ | T | C | 0.138 | -0.048104 | 0.0129 | 0.000186 |
| rs76702514 | SCZ | G | C | 0.212 | 0.020397 | 0.0106 | 0.05501 |
| rs76878669 | SCZ | G | C | 0.241 | -0.020998 | 0.0101 | 0.03813 |
| rs76954012 | SCZ | A | T | 0.087 | 0.024395 | 0.0155 | 0.114 |
| rs7704530 | SCZ | A | G | 0.727 | -0.013696 | 0.0096 | 0.1557 |
| rs7762794 | SCZ | G | A | 0.297 | -0.025502 | 0.0094 | 0.006589 |
| rs77702622 | SCZ | A | G | 0.058 | 0.021602 | 0.0188 | 0.25 |
| rs7774 | SCZ | A | C | 0.33 | -0.010396 | 0.0094 | 0.2642 |
| rs77835879 | SCZ | G | A | 0.096 | -0.023003 | 0.015 | 0.1248 |
| rs778371 | SCZ | G | A | 0.286 | 0.080603 | 0.0095 | 1.49E-17 |
| rs7785195 | SCZ | A | G | 0.66 | -0.009396 | 0.0091 | 0.3 |
| rs7803932 | SCZ | A | G | 0.163 | -0.036602 | 0.0118 | 0.001887 |
| rs78086698 | SCZ | C | T | 0.038 | -0.022505 | 0.0233 | 0.3329 |
| rs7852189 | SCZ | G | A | 0.334 | -0.001399 | 0.0092 | 0.8783 |
| rs7893571 | SCZ | T | G | 0.675 | -0.002098 | 0.0092 | 0.8203 |
| rs7909331 | SCZ | G | A | 0.164 | -0.003803 | 0.0118 | 0.7486 |
| rs7920624 | SCZ | T | A | 0.518 | -0.005505 | 0.0086 | 0.5201 |
| rs7921378 | SCZ | C | G | 0.48 | 0.008204 | 0.0087 | 0.3413 |
| rs7924036 | SCZ | T | G | 0.512 | 0.016597 | 0.0086 | 0.05379 |
| rs79265434 | SCZ | G | A | 0.11 | 0.084001 | 0.0136 | 6.63E-10 |
| rs79269403 | SCZ | A | G | 0.223 | -0.005505 | 0.0105 | 0.602101 |
| rs7927195 | SCZ | G | A | 0.628 | -0.001998 | 0.0089 | 0.8243 |
| rs7928622 | SCZ | T | A | 0.326 | 0.009101 | 0.0093 | 0.3267 |
| rs7938812 | SCZ | G | T | 0.416 | 0.026498 | 0.0088 | 0.002463 |
| rs79445414 | SCZ | C | T | 0.04 | 0.1234 | 0.0222 | 2.80E-08 |
| rs7944782 | SCZ | G | T | 0.514 | -0.049304 | 0.0087 | 1.25E-08 |
| rs7952102 | SCZ | C | T | 0.389 | 0.0168 | 0.009 | 0.06275 |
| rs7996639 | SCZ | A | G | 0.469 | 0.001501 | 0.0086 | 0.8607 |
| rs80153284 | SCZ | A | C | 0.016 | 0.022604 | 0.0372 | 0.5432 |
| rs8015400 | SCZ | A | C | 0.673 | -0.012995 | 0.0092 | 0.1583 |
| rs8020034 | SCZ | A | G | 0.181 | -0.017899 | 0.0113 | 0.1129 |
| rs8112818 | SCZ | G | A | 0.405 | -0.012995 | 0.0089 | 0.1428 |
| rs8132491 | SCZ | A | G | 0.311 | -0.025297 | 0.0097 | 0.0092 |
| rs815163 | SCZ | C | T | 0.572 | -0.007204 | 0.0087 | 0.4083 |
| rs862320 | SCZ | T | C | 0.42 | -0.002297 | 0.0087 | 0.7888 |
| rs879620 | SCZ | T | C | 0.61 | 0.005897 | 0.009 | 0.513701 |
| rs892612 | SCZ | C | A | 0.848 | 0.028996 | 0.0121 | 0.0163 |
| rs9294260 | SCZ | A | G | 0.469 | 0.005998 | 0.0087 | 0.4896 |
| rs9296389 | SCZ | C | G | 0.417 | 0.0007 | 0.0088 | 0.9378 |
| rs9320493 | SCZ | G | A | 0.853 | 0.019703 | 0.0122 | 0.1068 |
| rs9349956 | SCZ | C | A | 0.179 | -0.004898 | 0.0114 | 0.666801 |
| rs935166 | SCZ | A | G | 0.51 | -0.019999 | 0.0086 | 0.02023 |
| rs9366863 | SCZ | C | T | 0.665 | -0.0063 | 0.0091 | 0.4864 |
| rs9372625 | SCZ | A | G | 0.372 | 0.017299 | 0.0089 | 0.05147 |
| rs9386319 | SCZ | G | A | 0.395 | -0.0002 | 0.0088 | 0.9833 |
| rs9478496 | SCZ | C | T | 0.168 | 0.017401 | 0.0115 | 0.1306 |
| rs9503598 | SCZ | A | G | 0.434 | -0.008603 | 0.0087 | 0.3236 |
| rs9514600 | SCZ | G | C | 0.505 | 0.028204 | 0.0086 | 0.001059 |
| rs9522173 | SCZ | T | A | 0.393 | -0.011504 | 0.0088 | 0.1925 |
| rs9529119 | SCZ | G | C | 0.778 | -0.001499 | 0.0104 | 0.8883 |
| rs9571687 | SCZ | A | C | 0.322 | 0.006904 | 0.0092 | 0.4505 |
| rs9616906 | SCZ | A | G | 0.448 | 0.024395 | 0.0087 | 0.004815 |
| rs9636107 | SCZ | G | A | 0.471 | 0.069897 | 0.0086 | 5.11E-16 |
| rs9643087 | SCZ | T | C | 0.523 | 0.017004 | 0.0087 | 0.05122 |
| rs969512 | SCZ | T | A | 0.338 | -0.015204 | 0.0091 | 0.09435 |
| rs9704097 | SCZ | A | C | 0.505 | -0.007303 | 0.0086 | 0.3936 |
| rs9852062 | SCZ | A | T | 0.571 | 0.007599 | 0.0087 | 0.382 |
| rs9860326 | SCZ | G | C | 0.331 | -0.019597 | 0.0092 | 0.03266 |
| rs9882532 | SCZ | C | T | 0.361 | 0.053601 | 0.0089 | 2.03E-09 |
| rs9888533 | SCZ | T | C | 0.534 | 0.006803 | 0.0091 | 0.4522 |
| rs9951619 | SCZ | G | T | 0.76 | -0.021096 | 0.0101 | 0.03713 |
| rs9964724 | SCZ | T | C | 0.685 | -0.017702 | 0.0092 | 0.05578 |
| rs1000237 | SI | A | T | 0.361 | 0.009445 | 0.003725 | 0.0112 |
| rs10073890 | SI | G | A | 0.739 | 0.003734 | 0.004106 | 0.363 |
| rs10160769 | SI | C | G | 0.244 | -0.007483 | 0.004306 | 0.081999 |
| rs1017529 | SI | A | C | 0.143 | -0.004016 | 0.004733 | 0.396 |
| rs10423928 | SI | A | T | 0.203 | 0.002952 | 0.00442 | 0.504001 |
| rs10505836 | SI | C | A | 0.827 | 0.012199 | 0.005078 | 0.0163 |
| rs10510025 | SI | T | C | 0.254 | 0.018644 | 0.004128 | 6.31E-06 |
| rs1064213 | SI | A | G | 0.482 | 0.007601 | 0.00356 | 0.0328 |
| rs10742752 | SI | C | T | 0.621 | -0.002161 | 0.003654 | 0.554 |
| rs10752613 | SI | A | T | 0.296 | 0.002386 | 0.003902 | 0.541 |
| rs10765775 | SI | A | G | 0.373 | -0.012847 | 0.003648 | 0.000423 |
| rs10773002 | SI | T | A | 0.73 | 0.002072 | 0.004111 | 0.615 |
| rs10797055 | SI | G | A | 0.477 | -0.005866 | 0.003557 | 0.0992 |
| rs10858054 | SI | T | G | 0.181 | -0.005597 | 0.004648 | 0.228 |
| rs10861176 | SI | A | G | 0.742 | 0.000984 | 0.004053 | 0.808 |
| rs10887578 | SI | C | G | 0.47 | -0.001827 | 0.003556 | 0.608001 |
| rs10887801 | SI | T | G | 0.433 | -0.004944 | 0.003587 | 0.168 |
| rs10922907 | SI | T | A | 0.526 | -0.022251 | 0.003568 | 4.38E-10 |
| rs10938398 | SI | A | G | 0.424 | -0.002981 | 0.003596 | 0.407 |
| rs10963297 | SI | G | C | 0.227 | -0.00407 | 0.004128 | 0.324 |
| rs11000993 | SI | C | T | 0.128 | 0.010771 | 0.005511 | 0.0506 |
| rs11012732 | SI | G | A | 0.333 | 0.024139 | 0.003784 | 1.76E-10 |
| rs1105307 | SI | A | G | 0.275 | 0.005332 | 0.004089 | 0.193 |
| rs11079849 | SI | T | C | 0.313 | -0.004549 | 0.003817 | 0.233 |
| rs11081529 | SI | C | T | 0.275 | -0.011001 | 0.003887 | 0.00466 |
| rs11099020 | SI | T | C | 0.611 | -0.000689 | 0.003706 | 0.852 |
| rs11134679 | SI | G | A | 0.687 | 0.014062 | 0.003827 | 0.000235 |
| rs11165643 | SI | T | C | 0.57 | 0.014077 | 0.003611 | 9.89E-05 |
| rs11250094 | SI | C | G | 0.525 | -0.010331 | 0.003584 | 0.00394 |
| rs112633616 | SI | C | A | 0.0269 | -0.007701 | 0.009967 | 0.44 |
| rs112687095 | SI | A | G | 0.168 | -0.000316 | 0.00479 | 0.947 |
| rs113338260 | SI | C | T | 0.207 | 0.009435 | 0.00438 | 0.0313 |
| rs113520408 | SI | A | G | 0.261 | -0.008955 | 0.003977 | 0.0243 |
| rs113624107 | SI | A | G | 0.212 | 0.00098 | 0.004237 | 0.817 |
| rs1143770 | SI | T | C | 0.578 | -0.01396 | 0.003604 | 0.000109 |
| rs115000530 | SI | T | A | 0.0509 | -0.014504 | 0.007832 | 0.0641 |
| rs115454970 | SI | T | G | 0.263 | 0.002019 | 0.004276 | 0.636999 |
| rs11587347 | SI | G | C | 0.0966 | 0.001459 | 0.006101 | 0.811 |
| rs11620355 | SI | A | G | 0.089 | -0.014941 | 0.006348 | 0.0186 |
| rs11635092 | SI | A | G | 0.341 | 0.000856 | 0.003713 | 0.818 |
| rs116377258 | SI | G | A | 0.0269 | 0.020655 | 0.010473 | 0.0486 |
| rs11693094 | SI | T | C | 0.44 | -0.00212 | 0.003578 | 0.553 |
| rs11696755 | SI | C | T | 0.182 | 0.001108 | 0.00461 | 0.81 |
| rs11709402 | SI | G | A | 0.251 | 0.001222 | 0.003968 | 0.758 |
| rs117118217 | SI | C | G | 0.0138 | 0.011875 | 0.013833 | 0.391 |
| rs11732657 | SI | A | G | 0.716 | 0.007503 | 0.004063 | 0.065 |
| rs118136827 | SI | T | G | 0.271 | 0.015283 | 0.004 | 0.000136 |
| rs11866420 | SI | G | C | 0.63 | 0.012705 | 0.003608 | 0.000437 |
| rs1198588 | SI | T | A | 0.792 | 0.004644 | 0.00435 | 0.286 |
| rs12001437 | SI | C | T | 0.392 | 0.002757 | 0.003678 | 0.453 |
| rs12033257 | SI | G | A | 0.394 | -0.0084 | 0.003659 | 0.0218 |
| rs12140153 | SI | T | G | 0.0715 | -0.015774 | 0.005958 | 0.00811 |
| rs12149660 | SI | A | G | 0.111 | -0.004482 | 0.005451 | 0.411 |
| rs12156160 | SI | G | A | 0.127 | 0.004563 | 0.004952 | 0.357 |
| rs12204714 | SI | T | C | 0.63 | -0.008384 | 0.003701 | 0.0235 |
| rs12293670 | SI | G | A | 0.343 | -0.01197 | 0.003767 | 0.0015 |
| rs12303743 | SI | C | G | 0.0888 | 0.012654 | 0.005926 | 0.0328 |
| rs12364470 | SI | G | T | 0.15 | -0.007686 | 0.004767 | 0.107 |
| rs12375949 | SI | C | T | 0.571 | -0.010083 | 0.003594 | 0.00504 |
| rs12375985 | SI | A | G | 0.333 | 0.004685 | 0.003727 | 0.208 |
| rs12427047 | SI | T | C | 0.241 | 0.007 | 0.004048 | 0.083701 |
| rs12519073 | SI | T | C | 0.234 | 0.009922 | 0.00446 | 0.0261 |
| rs12523398 | SI | A | T | 0.183 | -0.01067 | 0.004772 | 0.0253 |
| rs12643771 | SI | T | C | 0.315 | -0.009305 | 0.003844 | 0.0154 |
| rs1266874 | SI | G | A | 0.381 | -0.006342 | 0.003737 | 0.089499 |
| rs12681792 | SI | A | C | 0.2 | 0.000831 | 0.004668 | 0.859 |
| rs12682775 | SI | C | T | 0.222 | -0.003614 | 0.004292 | 0.4 |
| rs12712510 | SI | C | T | 0.503 | -0.006911 | 0.003564 | 0.052601 |
| rs12714592 | SI | C | A | 0.269 | 0.016979 | 0.003991 | 2.13E-05 |
| rs12714702 | SI | G | A | 0.831 | 0.01333 | 0.004813 | 0.00562 |
| rs12757779 | SI | A | G | 0.191 | -0.015961 | 0.004251 | 0.000174 |
| rs12762034 | SI | C | T | 0.0824 | 0.004741 | 0.006625 | 0.474 |
| rs1286058 | SI | A | T | 0.71 | -0.008455 | 0.003904 | 0.0304 |
| rs12881629 | SI | G | A | 0.075 | 0.005268 | 0.00657 | 0.423 |
| rs12907546 | SI | A | G | 0.215 | 0.032118 | 0.004335 | 1.29E-13 |
| rs12919291 | SI | C | G | 0.194 | 0.010227 | 0.004569 | 0.0253 |
| rs12940014 | SI | C | T | 0.486 | -6.66E-05 | 0.003558 | 0.985 |
| rs12955211 | SI | A | T | 0.31 | 0.002021 | 0.003799 | 0.593999 |
| rs12956148 | SI | A | C | 0.324 | 0.010343 | 0.003926 | 0.00844 |
| rs1296328 | SI | C | A | 0.519 | -0.011361 | 0.003575 | 0.00147 |
| rs12977787 | SI | A | G | 0.524 | 0.005276 | 0.003565 | 0.139 |
| rs13030994 | SI | A | G | 0.485 | 0.036093 | 0.003556 | 3.56E-24 |
| rs13037326 | SI | T | C | 0.27 | 0.008667 | 0.004068 | 0.0332 |
| rs13090388 | SI | T | C | 0.313 | -0.009217 | 0.003841 | 0.0164 |
| rs13107325 | SI | T | C | 0.0654 | -0.017541 | 0.007258 | 0.0156 |
| rs13141210 | SI | T | C | 0.48 | -0.01043 | 0.003561 | 0.0034 |
| rs13145650 | SI | T | C | 0.92 | 0.01451 | 0.006444 | 0.0244 |
| rs13175535 | SI | A | G | 0.302 | -0.009372 | 0.003898 | 0.0162 |
| rs13195636 | SI | C | A | 0.0767 | -0.024483 | 0.005869 | 3.02E-05 |
| rs1320251 | SI | T | C | 0.486 | -0.000722 | 0.003572 | 0.84 |
| rs13218383 | SI | G | C | 0.346 | -0.012955 | 0.00374 | 0.00053 |
| rs13233308 | SI | T | C | 0.471 | -0.003352 | 0.003557 | 0.346 |
| rs13248187 | SI | C | T | 0.248 | -0.008314 | 0.004033 | 0.0391 |
| rs13261666 | SI | T | G | 0.522 | -0.026895 | 0.003556 | 3.90E-14 |
| rs1327259 | SI | G | A | 0.423 | 0.005684 | 0.003654 | 0.119 |
| rs13292699 | SI | C | A | 0.432 | -0.001622 | 0.003592 | 0.651 |
| rs1330199 | SI | T | G | 0.488 | -0.003297 | 0.003557 | 0.354 |
| rs13307225 | SI | A | G | 0.905 | -0.005905 | 0.005926 | 0.319 |
| rs13317303 | SI | A | C | 0.137 | -0.00295 | 0.004965 | 0.553 |
| rs1334297 | SI | A | G | 0.751 | -0.006204 | 0.004004 | 0.121 |
| rs13422673 | SI | T | C | 0.457 | 0.001172 | 0.003566 | 0.743 |
| rs13427822 | SI | G | A | 0.265 | 0.004223 | 0.003973 | 0.289 |
| rs1346841 | SI | A | G | 0.41 | 0.006976 | 0.003647 | 0.0559 |
| rs1360201 | SI | T | C | 0.474 | 0.002002 | 0.003556 | 0.573 |
| rs1363862 | SI | A | G | 0.29 | 0.005443 | 0.004023 | 0.176 |
| rs13642 | SI | T | A | 0.351 | -0.008092 | 0.003713 | 0.0293 |
| rs1391438 | SI | C | T | 0.666 | -0.002773 | 0.003827 | 0.469 |
| rs1392816 | SI | T | C | 0.372 | -0.025769 | 0.003659 | 1.92E-12 |
| rs140159717 | SI | T | C | 0.074 | -0.00622 | 0.006534 | 0.341 |
| rs1438945 | SI | A | T | 0.739 | 0.004264 | 0.003942 | 0.279 |
| rs1441264 | SI | A | G | 0.612 | 0.001804 | 0.003623 | 0.617999 |
| rs1450782 | SI | G | T | 0.582 | 0.01228 | 0.00359 | 0.00062 |
| rs1455350 | SI | A | T | 0.493 | -0.016758 | 0.003557 | 2.50E-06 |
| rs1458156 | SI | T | C | 0.476 | -0.002796 | 0.003556 | 0.432 |
| rs1471740 | SI | C | T | 0.749 | 0.004867 | 0.004084 | 0.233 |
| rs147568678 | SI | C | T | 0.224 | -0.002789 | 0.004199 | 0.507 |
| rs1477290 | SI | C | T | 0.149 | 0.004434 | 0.005219 | 0.395 |
| rs1503526 | SI | C | T | 0.501 | 0.009377 | 0.003557 | 0.00838 |
| rs152603 | SI | G | A | 0.365 | -0.009617 | 0.003715 | 0.00962 |
| rs1565735 | SI | A | T | 0.212 | -0.037618 | 0.004461 | 3.42E-17 |
| rs1566085 | SI | T | G | 0.535 | 0.006964 | 0.003568 | 0.0511 |
| rs1582931 | SI | A | G | 0.478 | -0.000112 | 0.003559 | 0.975 |
| rs1584469 | SI | T | C | 0.285 | -0.001882 | 0.003883 | 0.628001 |
| rs1609010 | SI | G | A | 0.549 | -0.011171 | 0.003583 | 0.00182 |
| rs1671770 | SI | C | A | 0.817 | 0.018194 | 0.004778 | 0.00014 |
| rs16846140 | SI | G | A | 0.331 | 0.001845 | 0.003767 | 0.624001 |
| rs16846463 | SI | G | A | 0.0964 | -0.006463 | 0.0059 | 0.274 |
| rs16854920 | SI | C | T | 0.329 | -0.001569 | 0.00375 | 0.676 |
| rs1689510 | SI | C | G | 0.314 | -0.019072 | 0.003769 | 4.19E-07 |
| rs16916303 | SI | G | A | 0.129 | 0.007169 | 0.005531 | 0.195 |
| rs16995054 | SI | T | C | 0.188 | -0.006815 | 0.004372 | 0.119 |
| rs17193211 | SI | T | C | 0.0673 | -0.003658 | 0.007201 | 0.611999 |
| rs17194490 | SI | T | G | 0.166 | 0.005316 | 0.005046 | 0.293 |
| rs17399739 | SI | G | A | 0.0732 | 0.017661 | 0.006895 | 0.0104 |
| rs17565975 | SI | A | G | 0.53 | 0.01155 | 0.003581 | 0.00129 |
| rs17598675 | SI | C | T | 0.45 | 0.005846 | 0.003558 | 0.101 |
| rs176218 | SI | T | G | 0.203 | -0.015824 | 0.004494 | 0.000435 |
| rs1778830 | SI | A | G | 0.365 | 0.008228 | 0.003691 | 0.0257 |
| rs1788808 | SI | G | A | 0.486 | 0.005262 | 0.003556 | 0.139 |
| rs1834144 | SI | A | C | 0.379 | 0.001923 | 0.003661 | 0.599 |
| rs1860002 | SI | T | C | 0.54 | -0.012089 | 0.003565 | 0.000715 |
| rs1899896 | SI | T | C | 0.286 | 0.026448 | 0.003887 | 1.04E-11 |
| rs1901512 | SI | C | T | 0.685 | -0.009472 | 0.003883 | 0.0147 |
| rs1915019 | SI | G | A | 0.746 | 0.009309 | 0.004205 | 0.0269 |
| rs1919243 | SI | C | T | 0.488 | 0.009292 | 0.003558 | 0.00902 |
| rs1950829 | SI | G | A | 0.481 | -0.00706 | 0.003561 | 0.0476 |
| rs1967772 | SI | A | G | 0.264 | -0.011907 | 0.00393 | 0.00244 |
| rs2035936 | SI | T | G | 0.0533 | 0.010995 | 0.007914 | 0.165 |
| rs2076603 | SI | A | G | 0.601 | 0.00086 | 0.003713 | 0.817 |
| rs2084572 | SI | G | A | 0.439 | -0.007309 | 0.003575 | 0.0409 |
| rs2102278 | SI | G | A | 0.326 | 8.61E-05 | 0.003811 | 0.982 |
| rs2133561 | SI | T | A | 0.577 | 0.002821 | 0.003657 | 0.44 |
| rs213518 | SI | C | T | 0.139 | -0.003846 | 0.005064 | 0.448 |
| rs2153740 | SI | G | A | 0.493 | -4.15E-05 | 0.003572 | 0.991 |
| rs215634 | SI | G | A | 0.601 | -0.011613 | 0.003672 | 0.00154 |
| rs2172131 | SI | C | T | 0.576 | -0.002336 | 0.003601 | 0.516 |
| rs217336 | SI | A | C | 0.413 | 0.013953 | 0.003579 | 9.91E-05 |
| rs2174752 | SI | T | G | 0.435 | 0.012718 | 0.003569 | 0.000359 |
| rs2176337 | SI | T | A | 0.341 | 0.007156 | 0.00383 | 0.0619 |
| rs217672 | SI | C | A | 0.257 | 0.003537 | 0.004049 | 0.382 |
| rs2214123 | SI | G | A | 0.632 | -0.009749 | 0.003706 | 0.0085 |
| rs2234458 | SI | T | C | 0.648 | -0.003209 | 0.003713 | 0.387 |
| rs2253310 | SI | G | C | 0.595 | 0.010533 | 0.003678 | 0.00419 |
| rs2281819 | SI | A | T | 0.236 | -0.010329 | 0.004199 | 0.0139 |
| rs2283076 | SI | G | A | 0.2 | 0.013024 | 0.004292 | 0.00241 |
| rs2289379 | SI | T | C | 0.392 | 0.000959 | 0.003627 | 0.792 |
| rs2302761 | SI | T | C | 0.208 | -0.002644 | 0.004365 | 0.544999 |
| rs2306593 | SI | T | C | 0.496 | -0.007279 | 0.003556 | 0.0407 |
| rs2307111 | SI | C | T | 0.418 | -0.004621 | 0.003642 | 0.204 |
| rs2332700 | SI | G | C | 0.755 | 0.005219 | 0.004101 | 0.204 |
| rs2333321 | SI | G | A | 0.797 | -0.017344 | 0.00435 | 6.58E-05 |
| rs2342892 | SI | G | T | 0.515 | -0.00205 | 0.003558 | 0.564 |
| rs2347526 | SI | C | T | 0.669 | 0.006091 | 0.003756 | 0.105 |
| rs2396625 | SI | A | T | 0.416 | -0.001601 | 0.003608 | 0.656999 |
| rs2398861 | SI | G | A | 0.281 | -0.00317 | 0.004079 | 0.437 |
| rs240963 | SI | C | T | 0.836 | -0.041044 | 0.004837 | 2.16E-17 |
| rs242093 | SI | A | G | 0.593 | 0.008377 | 0.003608 | 0.0203 |
| rs2433733 | SI | A | G | 0.657 | -0.00255 | 0.003802 | 0.503 |
| rs2456020 | SI | T | C | 0.254 | 0.001566 | 0.004231 | 0.711001 |
| rs2482356 | SI | C | T | 0.44 | 0.001628 | 0.003604 | 0.651 |
| rs252761 | SI | T | G | 0.601 | 0.001846 | 0.0036 | 0.608001 |
| rs2554835 | SI | A | G | 0.391 | -0.001223 | 0.003623 | 0.736 |
| rs2568958 | SI | A | G | 0.628 | 0.005395 | 0.003629 | 0.137 |
| rs2606228 | SI | C | A | 0.676 | 0.010203 | 0.003708 | 0.00593 |
| rs2612030 | SI | C | T | 0.86 | -0.017868 | 0.004899 | 0.000268 |
| rs2616143 | SI | A | G | 0.296 | -0.001351 | 0.00379 | 0.721 |
| rs266047 | SI | A | G | 0.529 | -0.03051 | 0.003739 | 3.36E-16 |
| rs2678204 | SI | G | T | 0.306 | 0.00664 | 0.003753 | 0.076901 |
| rs2781668 | SI | T | C | 0.162 | 0.006561 | 0.004711 | 0.164 |
| rs2787101 | SI | T | C | 0.599 | 0.003797 | 0.003637 | 0.297 |
| rs2819336 | SI | C | T | 0.654 | 0.029805 | 0.003708 | 9.18E-16 |
| rs28373063 | SI | C | G | 0.18 | -0.010297 | 0.0047 | 0.0285 |
| rs28404639 | SI | T | C | 0.35 | 0.00204 | 0.003713 | 0.582 |
| rs28489620 | SI | A | G | 0.264 | 0.007289 | 0.00393 | 0.0637 |
| rs28513670 | SI | G | A | 0.2 | -0.00256 | 0.004627 | 0.58 |
| rs2875762 | SI | C | G | 0.253 | 0.001923 | 0.004205 | 0.647 |
| rs2876520 | SI | G | C | 0.48 | 0.012897 | 0.003563 | 0.000303 |
| rs2920503 | SI | T | C | 0.291 | 8.86E-05 | 0.003914 | 0.982 |
| rs2923431 | SI | C | G | 0.63 | -0.01176 | 0.003682 | 0.0014 |
| rs293566 | SI | C | T | 0.377 | 0.020982 | 0.00375 | 2.21E-08 |
| rs2962334 | SI | T | G | 0.0214 | -0.000899 | 0.012486 | 0.943 |
| rs2971970 | SI | G | T | 0.801 | -0.018473 | 0.004342 | 2.07E-05 |
| rs301800 | SI | C | T | 0.807 | 0.017959 | 0.0047 | 0.000132 |
| rs30266 | SI | A | G | 0.311 | 0.013388 | 0.003817 | 0.000446 |
| rs317656 | SI | A | T | 0.697 | -0.022742 | 0.003977 | 1.07E-08 |
| rs320693 | SI | C | G | 0.461 | -0.000226 | 0.003567 | 0.949 |
| rs3211995 | SI | A | G | 0.18 | -0.000748 | 0.004899 | 0.879 |
| rs329118 | SI | T | C | 0.425 | -0.01336 | 0.003596 | 0.000207 |
| rs34025316 | SI | T | C | 0.345 | -0.007797 | 0.003756 | 0.0378 |
| rs34045288 | SI | T | C | 0.323 | 0.010254 | 0.003769 | 0.00651 |
| rs34234296 | SI | A | G | 0.373 | -0.000102 | 0.003659 | 0.978 |
| rs34481751 | SI | A | C | 0.143 | -0.002921 | 0.00479 | 0.542 |
| rs34517439 | SI | A | C | 0.1 | 0.0233 | 0.005432 | 1.81E-05 |
| rs347551 | SI | G | C | 0.476 | -0.003219 | 0.003561 | 0.366 |
| rs34811474 | SI | A | G | 0.198 | -0.004478 | 0.004251 | 0.291 |
| rs35154326 | SI | G | A | 0.294 | -0.009739 | 0.003973 | 0.0142 |
| rs35309068 | SI | G | T | 0.446 | -0.006385 | 0.003586 | 0.0749 |
| rs354155 | SI | C | G | 0.111 | -0.005282 | 0.006128 | 0.389 |
| rs35417702 | SI | T | C | 0.527 | -0.000507 | 0.003558 | 0.887 |
| rs35475880 | SI | T | G | 0.187 | -0.005932 | 0.00435 | 0.172 |
| rs35532491 | SI | T | A | 0.0988 | -0.00662 | 0.005874 | 0.26 |
| rs355777 | SI | C | G | 0.418 | 0.010014 | 0.003623 | 0.00572 |
| rs35867081 | SI | G | A | 0.51 | -0.010555 | 0.003556 | 0.003 |
| rs36007635 | SI | A | G | 0.133 | -0.004276 | 0.00517 | 0.408 |
| rs36061954 | SI | T | C | 0.382 | 0.014811 | 0.003635 | 4.60E-05 |
| rs36119825 | SI | A | G | 0.446 | -0.004564 | 0.003575 | 0.201 |
| rs363096 | SI | C | T | 0.583 | 0.001551 | 0.003596 | 0.666 |
| rs3747631 | SI | C | G | 0.201 | -0.014553 | 0.004388 | 0.000917 |
| rs3764625 | SI | G | T | 0.593 | 0.002606 | 0.003603 | 0.47 |
| rs3770754 | SI | G | C | 0.373 | -0.003021 | 0.003701 | 0.415 |
| rs3784710 | SI | C | T | 0.231 | -0.010984 | 0.004292 | 0.0105 |
| rs3800546 | SI | G | C | 0.291 | 0.012542 | 0.004033 | 0.00187 |
| rs3807865 | SI | A | G | 0.415 | 0.0122 | 0.003613 | 0.000734 |
| rs3809634 | SI | G | A | 0.318 | -0.007904 | 0.003834 | 0.0393 |
| rs3814883 | SI | T | C | 0.44 | 0.008247 | 0.003559 | 0.0204 |
| rs3845344 | SI | T | C | 0.381 | 0.019055 | 0.003654 | 1.79E-07 |
| rs3851998 | SI | G | C | 0.761 | -0.01674 | 0.004084 | 4.08E-05 |
| rs3896224 | SI | G | A | 0.446 | -0.013587 | 0.003783 | 0.000323 |
| rs3897821 | SI | G | A | 0.335 | -0.005058 | 0.003781 | 0.181 |
| rs3901286 | SI | A | C | 0.196 | -0.028846 | 0.004925 | 4.82E-09 |
| rs394608 | SI | C | T | 0.534 | 0.013857 | 0.003566 | 9.94E-05 |
| rs40071 | SI | C | T | 0.197 | -0.016128 | 0.004617 | 0.000483 |
| rs4044321 | SI | G | A | 0.642 | -0.027842 | 0.003711 | 6.08E-14 |
| rs4055791 | SI | T | C | 0.396 | 0.014764 | 0.003602 | 4.08E-05 |
| rs406388 | SI | G | C | 0.158 | 0.009111 | 0.004608 | 0.048101 |
| rs4148155 | SI | G | A | 0.1 | 0.010929 | 0.005682 | 0.0543 |
| rs4261944 | SI | G | T | 0.389 | 0.004498 | 0.003697 | 0.224 |
| rs4267103 | SI | C | T | 0.196 | 0.007078 | 0.004617 | 0.125 |
| rs429343 | SI | G | A | 0.563 | 0.000672 | 0.003599 | 0.852 |
| rs429358 | SI | C | T | 0.141 | -0.006225 | 0.005206 | 0.232 |
| rs4328757 | SI | T | C | 0.61 | -0.003503 | 0.003813 | 0.358 |
| rs4352658 | SI | T | C | 0.0794 | 0.017999 | 0.006416 | 0.00503 |
| rs4382592 | SI | G | T | 0.677 | -0.018815 | 0.003865 | 1.15E-06 |
| rs4419475 | SI | T | A | 0.414 | 0.008016 | 0.003617 | 0.0266 |
| rs4439537 | SI | C | T | 0.516 | -0.011799 | 0.003557 | 0.00093 |
| rs4477562 | SI | T | C | 0.13 | -0.010373 | 0.005321 | 0.0512 |
| rs4482463 | SI | A | C | 0.908 | -0.005242 | 0.006553 | 0.424 |
| rs4613074 | SI | C | T | 0.203 | -0.00212 | 0.004637 | 0.648 |
| rs4648450 | SI | A | C | 0.471 | -0.002296 | 0.003565 | 0.519 |
| rs4653164 | SI | T | C | 0.651 | 0.004343 | 0.003824 | 0.256 |
| rs4671328 | SI | G | T | 0.557 | 0.00633 | 0.003566 | 0.075701 |
| rs4700393 | SI | G | A | 0.548 | -0.015636 | 0.003559 | 1.09E-05 |
| rs4702 | SI | A | G | 0.552 | -0.007577 | 0.003576 | 0.0341 |
| rs4709807 | SI | C | T | 0.76 | -0.00476 | 0.004175 | 0.254 |
| rs4737188 | SI | T | A | 0.491 | 0.01244 | 0.003562 | 0.000489 |
| rs4757144 | SI | A | G | 0.561 | -0.012705 | 0.003608 | 0.000426 |
| rs4757957 | SI | C | G | 0.681 | -0.012478 | 0.003851 | 0.00121 |
| rs4764949 | SI | G | A | 0.365 | 0.001773 | 0.00379 | 0.64 |
| rs4790292 | SI | A | C | 0.151 | -0.002832 | 0.004952 | 0.567 |
| rs4810227 | SI | A | G | 0.61 | 0.002621 | 0.00367 | 0.475 |
| rs4812325 | SI | A | G | 0.602 | 0.010211 | 0.003656 | 0.00522 |
| rs4832298 | SI | T | C | 0.692 | -0.001462 | 0.003841 | 0.704001 |
| rs4846724 | SI | A | G | 0.532 | -0.002922 | 0.003564 | 0.412 |
| rs4876611 | SI | G | A | 0.735 | -0.000189 | 0.003942 | 0.962 |
| rs4895799 | SI | T | C | 0.574 | -0.001185 | 0.003604 | 0.743 |
| rs4958702 | SI | C | T | 0.564 | 0.009592 | 0.0036 | 0.00769 |
| rs4961705 | SI | C | G | 0.349 | -0.006415 | 0.003748 | 0.087201 |
| rs512121 | SI | C | T | 0.231 | 0.00561 | 0.00455 | 0.217 |
| rs539515 | SI | C | A | 0.179 | 0.012114 | 0.004412 | 0.00604 |
| rs55658481 | SI | A | G | 0.322 | 0.002525 | 0.003748 | 0.501 |
| rs55707359 | SI | G | T | 0.0145 | 0.007104 | 0.015078 | 0.638 |
| rs55714539 | SI | C | A | 0.354 | -0.006142 | 0.003745 | 0.101 |
| rs55726687 | SI | A | G | 0.187 | -0.003988 | 0.004404 | 0.365 |
| rs55736314 | SI | G | C | 0.387 | -0.010215 | 0.003632 | 0.0049 |
| rs558887 | SI | G | A | 0.298 | -0.003561 | 0.003865 | 0.357 |
| rs56094641 | SI | G | A | 0.401 | 0.006957 | 0.003622 | 0.0547 |
| rs56133507 | SI | G | T | 0.187 | 0.003526 | 0.004478 | 0.431 |
| rs56161855 | SI | T | A | 0.14 | 0.010679 | 0.005186 | 0.0394 |
| rs56203622 | SI | C | T | 0.166 | 0.002633 | 0.005021 | 0.6 |
| rs56335113 | SI | G | A | 0.675 | -0.00744 | 0.003847 | 0.0532 |
| rs56356382 | SI | C | T | 0.199 | -0.001966 | 0.004523 | 0.664 |
| rs56391344 | SI | A | G | 0.237 | -0.013862 | 0.004106 | 0.000741 |
| rs56399737 | SI | T | C | 0.444 | -0.003695 | 0.003572 | 0.3 |
| rs56858768 | SI | A | G | 0.284 | 0.000103 | 0.003876 | 0.979 |
| rs57636386 | SI | C | T | 0.0951 | -0.01416 | 0.006365 | 0.0261 |
| rs58120505 | SI | C | T | 0.404 | -0.009749 | 0.003599 | 0.00673 |
| rs59086897 | SI | A | T | 0.478 | 0.013872 | 0.003558 | 9.93E-05 |
| rs59237168 | SI | C | T | 0.229 | 0.000727 | 0.004327 | 0.867 |
| rs5995843 | SI | G | A | 0.342 | 0.007515 | 0.003725 | 0.0437 |
| rs60764613 | SI | T | G | 0.152 | 0.01424 | 0.005006 | 0.00445 |
| rs6123924 | SI | G | A | 0.176 | -0.00641 | 0.004916 | 0.192 |
| rs6134916 | SI | T | C | 0.48 | -0.004069 | 0.003569 | 0.254 |
| rs61813324 | SI | T | C | 0.122 | 0.00312 | 0.005252 | 0.552 |
| rs61828088 | SI | A | G | 0.116 | 0.000856 | 0.006005 | 0.887 |
| rs61903695 | SI | G | A | 0.246 | 0.011853 | 0.004109 | 0.00393 |
| rs61914045 | SI | A | G | 0.206 | 0.011334 | 0.004412 | 0.0102 |
| rs62097985 | SI | T | C | 0.394 | 0.01447 | 0.003595 | 5.83E-05 |
| rs62107261 | SI | C | T | 0.042 | -0.035815 | 0.008513 | 2.58E-05 |
| rs62134195 | SI | T | C | 0.0347 | -0.022383 | 0.009161 | 0.0145 |
| rs62176243 | SI | T | A | 0.246 | -0.007758 | 0.0041 | 0.0584 |
| rs62176993 | SI | A | G | 0.393 | 0.001343 | 0.003642 | 0.712 |
| rs62190049 | SI | C | G | 0.381 | -0.003358 | 0.003647 | 0.357 |
| rs62439690 | SI | A | G | 0.249 | 0.020174 | 0.004084 | 7.84E-07 |
| rs6265 | SI | T | C | 0.203 | -0.031786 | 0.004578 | 3.77E-12 |
| rs6531639 | SI | A | G | 0.22 | 0.010689 | 0.004145 | 0.00989 |
| rs6545714 | SI | A | G | 0.607 | -0.014957 | 0.003638 | 3.97E-05 |
| rs6546857 | SI | G | A | 0.234 | 0.008386 | 0.004157 | 0.0437 |
| rs6560906 | SI | C | T | 0.722 | -0.004893 | 0.003821 | 0.2 |
| rs6567160 | SI | C | T | 0.225 | 0.002383 | 0.004193 | 0.57 |
| rs6575340 | SI | A | G | 0.624 | 0.011543 | 0.003702 | 0.00182 |
| rs66511648 | SI | C | T | 0.272 | -0.001338 | 0.003964 | 0.736 |
| rs66679256 | SI | T | C | 0.45 | 0.011884 | 0.003583 | 0.000913 |
| rs6669341 | SI | G | A | 0.581 | -0.004537 | 0.003609 | 0.209 |
| rs6682438 | SI | C | T | 0.656 | 0.018218 | 0.003799 | 1.64E-06 |
| rs6688826 | SI | C | T | 0.292 | 0.016185 | 0.003914 | 3.55E-05 |
| rs6707827 | SI | G | A | 0.719 | 0.005452 | 0.003894 | 0.162 |
| rs6710091 | SI | G | C | 0.331 | -0.012028 | 0.00373 | 0.00128 |
| rs6719762 | SI | C | T | 0.462 | 0.011913 | 0.00356 | 0.000806 |
| rs6731967 | SI | C | G | 0.246 | 0.013102 | 0.004175 | 0.0017 |
| rs6744646 | SI | G | A | 0.827 | 0.03465 | 0.004733 | 2.52E-13 |
| rs6744794 | SI | G | C | 0.623 | 0.003485 | 0.003659 | 0.341 |
| rs6752979 | SI | A | G | 0.324 | 0.010522 | 0.003814 | 0.00581 |
| rs6774894 | SI | A | T | 0.362 | 0.009053 | 0.003708 | 0.0146 |
| rs6798742 | SI | G | A | 0.323 | -0.00198 | 0.003891 | 0.611 |
| rs6803651 | SI | T | G | 0.406 | 0.001427 | 0.003591 | 0.691 |
| rs6805241 | SI | C | T | 0.209 | 0.0092 | 0.004257 | 0.0307 |
| rs6843852 | SI | T | C | 0.528 | 0.002695 | 0.003557 | 0.449 |
| rs6943762 | SI | C | T | 0.118 | -0.007557 | 0.005357 | 0.159 |
| rs6959891 | SI | G | A | 0.275 | -0.007734 | 0.003947 | 0.0501 |
| rs6962980 | SI | C | A | 0.528 | -0.000987 | 0.003573 | 0.782 |
| rs6974218 | SI | C | A | 0.378 | -0.003458 | 0.003657 | 0.344 |
| rs698147 | SI | G | A | 0.532 | -0.008477 | 0.003573 | 0.0177 |
| rs7008955 | SI | G | T | 0.509 | -0.009576 | 0.003559 | 0.00712 |
| rs7012546 | SI | T | C | 0.403 | -0.002273 | 0.003603 | 0.528 |
| rs7029718 | SI | A | G | 0.406 | -0.001086 | 0.003611 | 0.763999 |
| rs7031698 | SI | C | T | 0.775 | -0.011318 | 0.004251 | 0.00773 |
| rs7034554 | SI | G | A | 0.367 | 0.008424 | 0.00368 | 0.0221 |
| rs7038943 | SI | C | T | 0.35 | -0.01225 | 0.003745 | 0.0011 |
| rs7079070 | SI | A | G | 0.443 | 0.012184 | 0.003577 | 0.000675 |
| rs708228 | SI | T | C | 0.309 | -0.000101 | 0.00379 | 0.979 |
| rs7124681 | SI | A | C | 0.397 | -0.008122 | 0.003629 | 0.0252 |
| rs7132908 | SI | A | G | 0.367 | -0.004087 | 0.003655 | 0.264 |
| rs71646142 | SI | T | C | 0.177 | -0.001147 | 0.004588 | 0.803 |
| rs7201895 | SI | A | G | 0.347 | -0.005238 | 0.003722 | 0.16 |
| rs7206608 | SI | G | C | 0.303 | -0.005341 | 0.003796 | 0.159 |
| rs7218014 | SI | C | T | 0.233 | 0.01282 | 0.004444 | 0.00392 |
| rs7233920 | SI | A | G | 0.218 | -0.002583 | 0.004218 | 0.54 |
| rs7236339 | SI | A | G | 0.203 | 0.01785 | 0.004231 | 2.41E-05 |
| rs723672 | SI | T | C | 0.46 | -0.001604 | 0.003587 | 0.655 |
| rs7250833 | SI | T | C | 0.297 | 0.002124 | 0.003918 | 0.588 |
| rs7259070 | SI | C | T | 0.578 | 0.017912 | 0.003619 | 7.50E-07 |
| rs72673947 | SI | G | A | 0.12 | -0.000838 | 0.005728 | 0.884 |
| rs7278859 | SI | T | A | 0.315 | 0.000224 | 0.003865 | 0.954 |
| rs72887338 | SI | C | T | 0.354 | 0.018845 | 0.003661 | 2.63E-07 |
| rs72892910 | SI | T | G | 0.168 | -0.001045 | 0.004711 | 0.824 |
| rs72910629 | SI | G | A | 0.136 | -0.001656 | 0.005252 | 0.753 |
| rs72986630 | SI | T | C | 0.0628 | -0.003802 | 0.0074 | 0.607 |
| rs73026725 | SI | A | C | 0.132 | -0.012034 | 0.004993 | 0.016 |
| rs730384 | SI | A | G | 0.425 | -0.004322 | 0.003589 | 0.229 |
| rs73052033 | SI | C | T | 0.165 | -0.007399 | 0.004598 | 0.108 |
| rs73142879 | SI | T | C | 0.183 | -0.004741 | 0.00452 | 0.295 |
| rs73213484 | SI | T | A | 0.152 | -0.002659 | 0.005108 | 0.603 |
| rs7331420 | SI | A | G | 0.281 | -0.004319 | 0.003926 | 0.271 |
| rs7332724 | SI | T | C | 0.273 | 0.009513 | 0.003964 | 0.0164 |
| rs73344830 | SI | G | A | 0.59 | 0.010011 | 0.003603 | 0.00546 |
| rs7357754 | SI | G | A | 0.487 | -0.002427 | 0.003556 | 0.495 |
| rs7359501 | SI | T | C | 0.385 | -0.00474 | 0.003635 | 0.192 |
| rs736282 | SI | C | T | 0.543 | -0.007333 | 0.003566 | 0.0396 |
| rs7442137 | SI | T | C | 0.624 | -0.001042 | 0.003708 | 0.778999 |
| rs7442885 | SI | G | C | 0.235 | -0.013213 | 0.00438 | 0.00256 |
| rs745249 | SI | T | C | 0.292 | 0.00201 | 0.003934 | 0.609 |
| rs7498044 | SI | A | G | 0.197 | 0.010147 | 0.004292 | 0.018 |
| rs7498665 | SI | G | A | 0.381 | -0.002964 | 0.003622 | 0.413 |
| rs75035127 | SI | G | A | 0.0298 | -0.023472 | 0.010702 | 0.0282 |
| rs7551758 | SI | G | T | 0.535 | 0.008194 | 0.003562 | 0.0214 |
| rs7575189 | SI | A | G | 0.553 | -0.012034 | 0.003612 | 0.000862 |
| rs7594904 | SI | C | T | 0.422 | 0.002721 | 0.003613 | 0.451 |
| rs76076331 | SI | T | C | 0.123 | -0.006434 | 0.005219 | 0.218 |
| rs76702514 | SI | G | C | 0.208 | -0.000561 | 0.004327 | 0.897 |
| rs76878669 | SI | G | C | 0.233 | -0.005885 | 0.004193 | 0.16 |
| rs76954012 | SI | A | T | 0.087 | 0.015062 | 0.006452 | 0.0196 |
| rs7704530 | SI | A | G | 0.711 | 0.015073 | 0.003986 | 0.000152 |
| rs7762794 | SI | G | A | 0.311 | 0.009724 | 0.003918 | 0.0131 |
| rs77702622 | SI | A | G | 0.0548 | -0.003926 | 0.007253 | 0.588 |
| rs7774 | SI | A | C | 0.351 | 0.006727 | 0.003821 | 0.0781 |
| rs77835879 | SI | G | A | 0.0894 | 0.008334 | 0.005999 | 0.164 |
| rs778371 | SI | G | A | 0.289 | 0.002807 | 0.003942 | 0.476 |
| rs7785195 | SI | A | G | 0.656 | -0.017833 | 0.003735 | 1.75E-06 |
| rs7803932 | SI | A | G | 0.157 | 0.005593 | 0.004744 | 0.238 |
| rs78086698 | SI | C | T | 0.038 | 0.008854 | 0.009493 | 0.351 |
| rs7852189 | SI | G | A | 0.357 | 0.006744 | 0.00383 | 0.0785 |
| rs7893571 | SI | T | G | 0.684 | 0.006004 | 0.00379 | 0.113 |
| rs7909331 | SI | G | A | 0.167 | 0.015145 | 0.004801 | 0.00161 |
| rs7920624 | SI | T | A | 0.502 | -0.005504 | 0.00356 | 0.122 |
| rs7921378 | SI | C | G | 0.463 | -0.02546 | 0.003558 | 8.26E-13 |
| rs7924036 | SI | T | G | 0.505 | -0.004744 | 0.003556 | 0.182 |
| rs79265434 | SI | G | A | 0.101 | 0.006046 | 0.005451 | 0.268 |
| rs79269403 | SI | A | G | 0.226 | -0.016977 | 0.004231 | 5.97E-05 |
| rs7927195 | SI | G | A | 0.637 | 0.015291 | 0.003655 | 2.94E-05 |
| rs7928622 | SI | T | A | 0.317 | -0.0086 | 0.00379 | 0.0233 |
| rs7938812 | SI | G | T | 0.424 | 0.043791 | 0.003637 | 2.71E-33 |
| rs79445414 | SI | C | T | 0.0394 | 0.016936 | 0.008284 | 0.041 |
| rs7944782 | SI | G | T | 0.511 | 0.006768 | 0.003557 | 0.057101 |
| rs7952102 | SI | C | T | 0.396 | -0.008463 | 0.003659 | 0.0207 |
| rs7996639 | SI | A | G | 0.485 | 7.21E-05 | 0.003577 | 0.984 |
| rs80153284 | SI | A | C | 0.0144 | 0.008595 | 0.014169 | 0.544 |
| rs8015400 | SI | A | C | 0.655 | -0.000936 | 0.003804 | 0.805 |
| rs8020034 | SI | A | G | 0.186 | -0.010624 | 0.004654 | 0.0225 |
| rs8112818 | SI | G | A | 0.415 | -0.001524 | 0.003632 | 0.675 |
| rs8132491 | SI | A | G | 0.312 | 0.006282 | 0.003837 | 0.102 |
| rs815163 | SI | C | T | 0.563 | -0.003193 | 0.003592 | 0.374 |
| rs862320 | SI | T | C | 0.407 | -0.013515 | 0.003612 | 0.000182 |
| rs879620 | SI | T | C | 0.585 | 0.008395 | 0.00365 | 0.0214 |
| rs892612 | SI | C | A | 0.853 | -0.007009 | 0.004956 | 0.157 |
| rs9294260 | SI | A | G | 0.466 | -0.00036 | 0.00356 | 0.92 |
| rs9296389 | SI | C | G | 0.429 | -0.006224 | 0.003617 | 0.0854 |
| rs9320493 | SI | G | A | 0.851 | 0.009592 | 0.005021 | 0.056201 |
| rs9349956 | SI | C | A | 0.188 | -0.007699 | 0.004668 | 0.099001 |
| rs935166 | SI | A | G | 0.482 | -0.001679 | 0.003556 | 0.636 |
| rs9366863 | SI | C | T | 0.65 | -0.006634 | 0.003793 | 0.0801 |
| rs9372625 | SI | A | G | 0.367 | -0.000937 | 0.003666 | 0.798 |
| rs9386319 | SI | G | A | 0.403 | -0.002616 | 0.003645 | 0.473 |
| rs9478496 | SI | C | T | 0.167 | 0.011888 | 0.004778 | 0.0128 |
| rs9503598 | SI | A | G | 0.431 | -0.005272 | 0.003587 | 0.142 |
| rs9514600 | SI | G | C | 0.51 | -0.008535 | 0.003556 | 0.0164 |
| rs9522173 | SI | T | A | 0.373 | 0.001977 | 0.003652 | 0.589 |
| rs9529119 | SI | G | C | 0.784 | 0.004593 | 0.004264 | 0.281 |
| rs9571687 | SI | A | C | 0.332 | 0.004699 | 0.003824 | 0.219 |
| rs9616906 | SI | A | G | 0.44 | 0.006389 | 0.003577 | 0.0741 |
| rs9636107 | SI | G | A | 0.49 | 0.01035 | 0.003562 | 0.00368 |
| rs9643087 | SI | T | C | 0.541 | 0.008523 | 0.00356 | 0.0167 |
| rs969512 | SI | T | A | 0.34 | -0.024684 | 0.003756 | 4.93E-11 |
| rs9704097 | SI | A | C | 0.499 | 0.002759 | 0.003556 | 0.438 |
| rs9852062 | SI | A | T | 0.581 | -0.002489 | 0.003581 | 0.487 |
| rs9860326 | SI | G | C | 0.318 | 0.003448 | 0.00379 | 0.363 |
| rs9882532 | SI | C | T | 0.354 | 0.018585 | 0.003695 | 4.83E-07 |
| rs9888533 | SI | T | C | 0.516 | 0.002157 | 0.003566 | 0.544999 |
| rs9951619 | SI | G | T | 0.751 | -0.003818 | 0.004181 | 0.361 |
| rs9964724 | SI | T | C | 0.674 | -0.005102 | 0.003824 | 0.182 |
| rs1000237 | Years of schooling | A | T | 0.3673 | -0.00953 | 0.00178 | 9.12E-08 |
| rs10073890 | Years of schooling | G | A | 0.7364 | -0.01262 | 0.00196 | 1.11E-10 |
| rs10160769 | Years of schooling | C | G | 0.2364 | 0.01247 | 0.00207 | 1.75E-09 |
| rs1017529 | Years of schooling | A | C | 0.1854 | -0.00326 | 0.00225 | 0.148 |
| rs10423928 | Years of schooling | A | T | 0.1956 | 0.00312 | 0.00214 | 0.145 |
| rs10505836 | Years of schooling | C | A | 0.852 | -0.00297 | 0.00245 | 0.226 |
| rs10510025 | Years of schooling | T | C | 0.2364 | -0.00659 | 0.00197 | 0.000844 |
| rs1064213 | Years of schooling | A | G | 0.517 | -0.00255 | 0.0017 | 0.133 |
| rs10742752 | Years of schooling | C | T | 0.6207 | 0.00261 | 0.00174 | 0.134 |
| rs10752613 | Years of schooling | A | T | 0.2517 | -0.00748 | 0.00187 | 6.19E-05 |
| rs10765775 | Years of schooling | A | G | 0.3963 | 0.01488 | 0.00176 | 2.62E-17 |
| rs10773002 | Years of schooling | T | A | 0.7211 | -0.02191 | 0.00197 | 8.68E-29 |
| rs10797055 | Years of schooling | G | A | 0.5102 | 0.00986 | 0.00172 | 1.04E-08 |
| rs10858054 | Years of schooling | T | G | 0.1871 | 0.00119 | 0.0022 | 0.588 |
| rs10861176 | Years of schooling | A | G | 0.7296 | 0.00503 | 0.00193 | 0.00917 |
| rs10887578 | Years of schooling | C | G | 0.4847 | -0.00187 | 0.0017 | 0.272 |
| rs10887801 | Years of schooling | T | G | 0.4371 | 0.01087 | 0.00171 | 2.27E-10 |
| rs10922907 | Years of schooling | T | A | 0.5289 | 0.01609 | 0.00171 | 4.26E-21 |
| rs10938398 | Years of schooling | A | G | 0.415 | -0.00663 | 0.00172 | 0.000114 |
| rs10963297 | Years of schooling | G | C | 0.2517 | 0.01904 | 0.00198 | 7.36E-22 |
| rs11000993 | Years of schooling | C | T | 0.1259 | -0.00267 | 0.00262 | 0.308 |
| rs11012732 | Years of schooling | G | A | 0.3486 | -0.00673 | 0.00182 | 0.000222 |
| rs1105307 | Years of schooling | A | G | 0.2449 | -0.01173 | 0.00195 | 1.67E-09 |
| rs11079849 | Years of schooling | T | C | 0.3078 | 0.00736 | 0.00183 | 5.57E-05 |
| rs11081529 | Years of schooling | C | T | 0.2568 | -0.01311 | 0.00186 | 1.82E-12 |
| rs11099020 | Years of schooling | T | C | 0.6531 | 0.00051 | 0.00177 | 0.772999 |
| rs11134679 | Years of schooling | G | A | 0.6786 | -0.00253 | 0.00183 | 0.166 |
| rs11165643 | Years of schooling | T | C | 0.5884 | -0.00236 | 0.00173 | 0.171 |
| rs11250094 | Years of schooling | C | G | 0.5527 | 0.00261 | 0.00171 | 0.126 |
| rs112633616 | Years of schooling | C | A | 0.03401 | 0.00743 | 0.00493 | 0.132 |
| rs112687095 | Years of schooling | A | G | 0.165 | 0.01325 | 0.00238 | 2.42E-08 |
| rs113338260 | Years of schooling | C | T | 0.2211 | 0.00279 | 0.00212 | 0.188 |
| rs113520408 | Years of schooling | A | G | 0.2857 | 0.01304 | 0.00192 | 1.02E-11 |
| rs113624107 | Years of schooling | A | G | 0.2415 | -0.00135 | 0.00202 | 0.505 |
| rs1143770 | Years of schooling | T | C | 0.5918 | 0.01136 | 0.00172 | 4.31E-11 |
| rs115000530 | Years of schooling | T | A | 0.06122 | 0.02892 | 0.00381 | 3.30E-14 |
| rs115454970 | Years of schooling | T | G | 0.3044 | -0.01185 | 0.00199 | 2.76E-09 |
| rs11587347 | Years of schooling | G | C | 0.1037 | 0.0041 | 0.00292 | 0.16 |
| rs11620355 | Years of schooling | A | G | 0.1156 | 0.01756 | 0.003 | 4.77E-09 |
| rs11635092 | Years of schooling | A | G | 0.3639 | -0.01231 | 0.00177 | 3.89E-12 |
| rs116377258 | Years of schooling | G | A | 0.02551 | 0.00478 | 0.00505 | 0.344 |
| rs11693094 | Years of schooling | T | C | 0.4082 | -0.003 | 0.00171 | 0.078401 |
| rs11696755 | Years of schooling | C | T | 0.199 | -5.00E-05 | 0.0022 | 0.983 |
| rs11709402 | Years of schooling | G | A | 0.2857 | -0.00373 | 0.0019 | 0.0501 |
| rs117118217 | Years of schooling | C | G | 0.01701 | -0.00657 | 0.00747 | 0.38 |
| rs11732657 | Years of schooling | A | G | 0.7007 | -0.01274 | 0.00197 | 9.54E-11 |
| rs118136827 | Years of schooling | T | G | 0.2738 | 0.00073 | 0.0019 | 0.702 |
| rs11866420 | Years of schooling | G | C | 0.631 | 0.0013 | 0.00174 | 0.452 |
| rs1198588 | Years of schooling | T | A | 0.7891 | 0.01112 | 0.00209 | 9.71E-08 |
| rs12001437 | Years of schooling | C | T | 0.3861 | 0.00145 | 0.00178 | 0.415 |
| rs12033257 | Years of schooling | G | A | 0.3639 | 0.00673 | 0.00177 | 0.000149 |
| rs12140153 | Years of schooling | T | G | 0.07653 | 0.0042 | 0.00293 | 0.152 |
| rs12149660 | Years of schooling | A | G | 0.1003 | 0.00328 | 0.00267 | 0.22 |
| rs12156160 | Years of schooling | G | A | 0.1259 | -0.00044 | 0.00246 | 0.857 |
| rs12204714 | Years of schooling | T | C | 0.6956 | 0.01494 | 0.00177 | 3.51E-17 |
| rs12293670 | Years of schooling | G | A | 0.3503 | -0.00455 | 0.0018 | 0.0115 |
| rs12303743 | Years of schooling | C | G | 0.08333 | -0.001 | 0.00284 | 0.726001 |
| rs12364470 | Years of schooling | G | T | 0.1327 | -0.005 | 0.00227 | 0.0274 |
| rs12375949 | Years of schooling | C | T | 0.5697 | 0.01447 | 0.00172 | 3.31E-17 |
| rs12375985 | Years of schooling | A | G | 0.3282 | 0.00489 | 0.00178 | 0.00594 |
| rs12427047 | Years of schooling | T | C | 0.2585 | -0.00408 | 0.00195 | 0.0367 |
| rs12519073 | Years of schooling | T | C | 0.2381 | -0.01221 | 0.00202 | 1.60E-09 |
| rs12523398 | Years of schooling | A | T | 0.1565 | 0.00946 | 0.00229 | 3.51E-05 |
| rs12643771 | Years of schooling | T | C | 0.3112 | 0.01518 | 0.00184 | 1.61E-16 |
| rs1266874 | Years of schooling | G | A | 0.3418 | -0.00226 | 0.00178 | 0.203 |
| rs12681792 | Years of schooling | A | C | 0.1871 | -0.00338 | 0.00213 | 0.112 |
| rs12682775 | Years of schooling | C | T | 0.2143 | 0.01187 | 0.00204 | 5.99E-09 |
| rs12712510 | Years of schooling | C | T | 0.5272 | 0.00113 | 0.00171 | 0.509 |
| rs12714592 | Years of schooling | C | A | 0.2704 | -0.0033 | 0.0019 | 0.0823 |
| rs12714702 | Years of schooling | G | A | 0.8316 | -0.00671 | 0.00233 | 0.00403 |
| rs12757779 | Years of schooling | A | G | 0.2075 | 0.00268 | 0.00204 | 0.187 |
| rs12762034 | Years of schooling | C | T | 0.07823 | -0.00587 | 0.00317 | 0.0638 |
| rs1286058 | Years of schooling | A | T | 0.75 | -0.00206 | 0.00187 | 0.27 |
| rs12881629 | Years of schooling | G | A | 0.05612 | 0.00582 | 0.00317 | 0.066599 |
| rs12907546 | Years of schooling | A | G | 0.2143 | -0.013 | 0.00208 | 4.11E-10 |
| rs12919291 | Years of schooling | C | G | 0.199 | 0.00372 | 0.00219 | 0.088699 |
| rs12940014 | Years of schooling | C | T | 0.5204 | 0.00936 | 0.0017 | 3.73E-08 |
| rs12955211 | Years of schooling | A | T | 0.3554 | 0.01097 | 0.00182 | 1.59E-09 |
| rs12956148 | Years of schooling | A | C | 0.3044 | -0.00214 | 0.00189 | 0.256 |
| rs1296328 | Years of schooling | C | A | 0.5646 | 0.00242 | 0.00173 | 0.162 |
| rs12977787 | Years of schooling | A | G | 0.5391 | -0.00279 | 0.00172 | 0.105 |
| rs13030994 | Years of schooling | A | G | 0.5034 | 0.00181 | 0.0017 | 0.286 |
| rs13037326 | Years of schooling | T | C | 0.2619 | -0.00993 | 0.00194 | 2.88E-07 |
| rs13090388 | Years of schooling | T | C | 0.3095 | 0.02852 | 0.00184 | 4.29E-54 |
| rs13107325 | Years of schooling | T | C | 0.09014 | -0.0188 | 0.00344 | 4.45E-08 |
| rs13141210 | Years of schooling | T | C | 0.5085 | 0.01361 | 0.00172 | 2.26E-15 |
| rs13145650 | Years of schooling | T | C | 0.90816 | -0.01918 | 0.00306 | 3.80E-10 |
| rs13175535 | Years of schooling | A | G | 0.2891 | 0.00266 | 0.00187 | 0.155 |
| rs13195636 | Years of schooling | C | A | 0.08844 | 0.00263 | 0.00278 | 0.345 |
| rs1320251 | Years of schooling | T | C | 0.4881 | 0.00377 | 0.00172 | 0.028 |
| rs13218383 | Years of schooling | G | C | 0.3469 | 0.00139 | 0.00179 | 0.438 |
| rs13233308 | Years of schooling | T | C | 0.4745 | 0.0033 | 0.0017 | 0.0522 |
| rs13248187 | Years of schooling | C | T | 0.2636 | -0.00143 | 0.00193 | 0.457 |
| rs13261666 | Years of schooling | T | G | 0.5187 | 0.00254 | 0.0017 | 0.135 |
| rs1327259 | Years of schooling | G | A | 0.381 | 0.00306 | 0.00174 | 0.079201 |
| rs13292699 | Years of schooling | C | A | 0.4609 | 0.00488 | 0.00172 | 0.00442 |
| rs1330199 | Years of schooling | T | G | 0.4167 | 0.00453 | 0.0017 | 0.00766 |
| rs13307225 | Years of schooling | A | G | 0.8929 | 0.01181 | 0.00282 | 2.76E-05 |
| rs13317303 | Years of schooling | A | C | 0.1395 | 2.00E-05 | 0.00243 | 0.993 |
| rs1334297 | Years of schooling | A | G | 0.784 | 0.02449 | 0.00192 | 3.06E-37 |
| rs13422673 | Years of schooling | T | C | 0.4847 | -0.01201 | 0.0017 | 1.74E-12 |
| rs13427822 | Years of schooling | G | A | 0.2687 | 0.00525 | 0.0019 | 0.00569 |
| rs1346841 | Years of schooling | A | G | 0.4337 | 0.00564 | 0.00174 | 0.00119 |
| rs1360201 | Years of schooling | T | C | 0.4592 | -0.00308 | 0.0017 | 0.0701 |
| rs1363862 | Years of schooling | A | G | 0.2602 | -0.01171 | 0.00192 | 1.02E-09 |
| rs13642 | Years of schooling | T | A | 0.3622 | -0.00236 | 0.00177 | 0.183 |
| rs1391438 | Years of schooling | C | T | 0.6854 | -0.0167 | 0.00183 | 5.79E-20 |
| rs1392816 | Years of schooling | T | C | 0.3895 | 0.0066 | 0.00175 | 0.000163 |
| rs140159717 | Years of schooling | T | C | 0.05612 | 0.00347 | 0.00328 | 0.291 |
| rs1438945 | Years of schooling | A | T | 0.733 | -0.001 | 0.00189 | 0.593999 |
| rs1441264 | Years of schooling | A | G | 0.5901 | -0.00177 | 0.00174 | 0.311 |
| rs1450782 | Years of schooling | G | T | 0.602 | -0.00945 | 0.00173 | 4.93E-08 |
| rs1455350 | Years of schooling | A | T | 0.4711 | -0.01614 | 0.0017 | 2.61E-21 |
| rs1458156 | Years of schooling | T | C | 0.4524 | 0.00668 | 0.0017 | 8.45E-05 |
| rs1471740 | Years of schooling | C | T | 0.7109 | -0.00193 | 0.00198 | 0.328 |
| rs147568678 | Years of schooling | C | T | 0.2313 | 0.00178 | 0.00201 | 0.377 |
| rs1477290 | Years of schooling | C | T | 0.1616 | 0.01723 | 0.00248 | 3.47E-12 |
| rs1503526 | Years of schooling | C | T | 0.4711 | -0.01051 | 0.0017 | 6.25E-10 |
| rs152603 | Years of schooling | G | A | 0.3861 | 0.01019 | 0.00177 | 9.47E-09 |
| rs1565735 | Years of schooling | A | T | 0.1871 | 0.00266 | 0.00212 | 0.21 |
| rs1566085 | Years of schooling | T | G | 0.5697 | 0.01645 | 0.00171 | 6.90E-22 |
| rs1582931 | Years of schooling | A | G | 0.449 | -0.0074 | 0.0017 | 1.39E-05 |
| rs1584469 | Years of schooling | T | C | 0.3112 | -0.01303 | 0.00185 | 2.10E-12 |
| rs1609010 | Years of schooling | G | A | 0.5697 | 0.00282 | 0.00171 | 0.0993 |
| rs1671770 | Years of schooling | C | A | 0.8061 | -0.01342 | 0.00223 | 1.91E-09 |
| rs16846140 | Years of schooling | G | A | 0.3401 | -0.00694 | 0.0018 | 0.00011 |
| rs16846463 | Years of schooling | G | A | 0.1173 | -0.02256 | 0.00283 | 1.38E-15 |
| rs16854920 | Years of schooling | C | T | 0.3554 | 0.01007 | 0.00181 | 2.51E-08 |
| rs1689510 | Years of schooling | C | G | 0.3435 | 0.01761 | 0.0018 | 1.40E-22 |
| rs16916303 | Years of schooling | G | A | 0.09524 | 0.00262 | 0.00265 | 0.323 |
| rs16995054 | Years of schooling | T | C | 0.2007 | -0.0139 | 0.00208 | 2.52E-11 |
| rs17193211 | Years of schooling | T | C | 0.07993 | 0.0056 | 0.0034 | 0.099701 |
| rs17194490 | Years of schooling | T | G | 0.1599 | 0.00929 | 0.0023 | 5.57E-05 |
| rs17399739 | Years of schooling | G | A | 0.05952 | 0.00354 | 0.00332 | 0.286 |
| rs17565975 | Years of schooling | A | G | 0.5306 | -0.01142 | 0.00171 | 2.56E-11 |
| rs17598675 | Years of schooling | C | T | 0.5187 | 0.01199 | 0.0017 | 1.75E-12 |
| rs176218 | Years of schooling | T | G | 0.2007 | 0.01883 | 0.00215 | 1.85E-18 |
| rs1778830 | Years of schooling | A | G | 0.3912 | -0.00556 | 0.00177 | 0.00165 |
| rs1788808 | Years of schooling | G | A | 0.5255 | 0.01358 | 0.0017 | 1.30E-15 |
| rs1834144 | Years of schooling | A | C | 0.335 | -0.00068 | 0.00175 | 0.697999 |
| rs1860002 | Years of schooling | T | C | 0.5153 | 0.00112 | 0.0017 | 0.512 |
| rs1899896 | Years of schooling | T | C | 0.3265 | -0.00186 | 0.00186 | 0.317 |
| rs1901512 | Years of schooling | C | T | 0.6701 | -0.0019 | 0.00185 | 0.304 |
| rs1915019 | Years of schooling | G | A | 0.7517 | -0.0035 | 0.00199 | 0.079001 |
| rs1919243 | Years of schooling | C | T | 0.5187 | -0.00564 | 0.0017 | 0.000919 |
| rs1950829 | Years of schooling | G | A | 0.517 | 0.00548 | 0.0017 | 0.00126 |
| rs1967772 | Years of schooling | A | G | 0.2296 | 0.00354 | 0.00189 | 0.0609 |
| rs2035936 | Years of schooling | T | G | 0.05952 | -0.01331 | 0.00367 | 0.000285 |
| rs2076603 | Years of schooling | A | G | 0.6446 | 0.00225 | 0.00177 | 0.204 |
| rs2084572 | Years of schooling | G | A | 0.4779 | 2.00E-05 | 0.00171 | 0.992 |
| rs2102278 | Years of schooling | G | A | 0.3027 | -1.00E-04 | 0.00183 | 0.954 |
| rs2133561 | Years of schooling | T | A | 0.5986 | -0.00139 | 0.00177 | 0.433 |
| rs213518 | Years of schooling | C | T | 0.1565 | -0.00098 | 0.00244 | 0.687 |
| rs2153740 | Years of schooling | G | A | 0.5068 | 0.00228 | 0.0017 | 0.181 |
| rs215634 | Years of schooling | G | A | 0.6344 | 0.00949 | 0.00175 | 5.98E-08 |
| rs2172131 | Years of schooling | C | T | 0.5425 | 0.00215 | 0.00173 | 0.214 |
| rs217336 | Years of schooling | A | C | 0.4473 | 7.00E-05 | 0.00171 | 0.967 |
| rs2174752 | Years of schooling | T | G | 0.4167 | -0.00669 | 0.00171 | 8.65E-05 |
| rs2176337 | Years of schooling | T | A | 0.3265 | -0.00523 | 0.00182 | 0.00415 |
| rs217672 | Years of schooling | C | A | 0.2568 | -0.00707 | 0.00197 | 0.000328 |
| rs2214123 | Years of schooling | G | A | 0.6548 | 0.00232 | 0.00178 | 0.193 |
| rs2234458 | Years of schooling | T | C | 0.6173 | 0.0074 | 0.00178 | 3.09E-05 |
| rs2253310 | Years of schooling | G | C | 0.5918 | 0.00933 | 0.00175 | 1.03E-07 |
| rs2281819 | Years of schooling | A | T | 0.2738 | 0.00648 | 0.00201 | 0.00124 |
| rs2283076 | Years of schooling | G | A | 0.2143 | -0.01143 | 0.00204 | 2.07E-08 |
| rs2289379 | Years of schooling | T | C | 0.415 | 0.00734 | 0.00174 | 2.33E-05 |
| rs2302761 | Years of schooling | T | C | 0.1905 | 0.01354 | 0.00209 | 1.00E-10 |
| rs2306593 | Years of schooling | T | C | 0.5 | 0.00583 | 0.0017 | 0.000625 |
| rs2307111 | Years of schooling | C | T | 0.381 | 0.00521 | 0.00174 | 0.00273 |
| rs2332700 | Years of schooling | G | C | 0.7585 | -0.00548 | 0.00196 | 0.00515 |
| rs2333321 | Years of schooling | G | A | 0.7874 | 0.00812 | 0.0021 | 0.000111 |
| rs2342892 | Years of schooling | G | T | 0.5323 | 0.0039 | 0.00171 | 0.0221 |
| rs2347526 | Years of schooling | C | T | 0.6378 | 0.01395 | 0.00179 | 6.84E-15 |
| rs2396625 | Years of schooling | A | T | 0.4133 | 0.00533 | 0.00172 | 0.0019 |
| rs2398861 | Years of schooling | G | A | 0.3027 | -0.00852 | 0.00194 | 1.11E-05 |
| rs240963 | Years of schooling | C | T | 0.8622 | -9.00E-04 | 0.00231 | 0.696 |
| rs242093 | Years of schooling | A | G | 0.5476 | -0.01031 | 0.00172 | 2.07E-09 |
| rs2433733 | Years of schooling | A | G | 0.6599 | 0.00572 | 0.00182 | 0.00162 |
| rs2456020 | Years of schooling | T | C | 0.2245 | 0.00421 | 0.00202 | 0.0367 |
| rs2482356 | Years of schooling | C | T | 0.4133 | 0.00294 | 0.00172 | 0.0877 |
| rs252761 | Years of schooling | T | G | 0.6139 | 0.00176 | 0.00174 | 0.313 |
| rs2554835 | Years of schooling | A | G | 0.398 | 0.00974 | 0.00175 | 2.69E-08 |
| rs2568958 | Years of schooling | A | G | 0.6327 | 0.01363 | 0.00174 | 4.22E-15 |
| rs2606228 | Years of schooling | C | A | 0.682 | 0.00531 | 0.00178 | 0.00292 |
| rs2612030 | Years of schooling | C | T | 0.8469 | 0.01024 | 0.00234 | 1.19E-05 |
| rs2616143 | Years of schooling | A | G | 0.3333 | 0.00048 | 0.00181 | 0.793 |
| rs266047 | Years of schooling | A | G | 0.4983 | 0.00882 | 0.0017 | 2.29E-07 |
| rs2678204 | Years of schooling | G | T | 0.3197 | -0.01094 | 0.00179 | 1.08E-09 |
| rs2781668 | Years of schooling | T | C | 0.1531 | 0.00484 | 0.0023 | 0.0348 |
| rs2787101 | Years of schooling | T | C | 0.6173 | 0.00968 | 0.00174 | 2.50E-08 |
| rs2819336 | Years of schooling | C | T | 0.6616 | -0.01828 | 0.00177 | 5.46E-25 |
| rs28373063 | Years of schooling | C | G | 0.2075 | 0.01389 | 0.00229 | 1.23E-09 |
| rs28404639 | Years of schooling | T | C | 0.3452 | -0.00198 | 0.00177 | 0.265 |
| rs28489620 | Years of schooling | A | G | 0.267 | 0.00386 | 0.00187 | 0.0389 |
| rs28513670 | Years of schooling | G | A | 0.1531 | 0.01477 | 0.00225 | 5.06E-11 |
| rs2875762 | Years of schooling | C | G | 0.2619 | -0.0012 | 0.00201 | 0.55 |
| rs2876520 | Years of schooling | G | C | 0.4796 | -0.00362 | 0.0017 | 0.0335 |
| rs2920503 | Years of schooling | T | C | 0.2687 | 0.00249 | 0.00188 | 0.185 |
| rs2923431 | Years of schooling | C | G | 0.6446 | 0.0114 | 0.00176 | 9.84E-11 |
| rs293566 | Years of schooling | C | T | 0.3112 | -0.00737 | 0.00179 | 3.75E-05 |
| rs2962334 | Years of schooling | T | G | 0.02211 | -0.00392 | 0.00594 | 0.509 |
| rs2971970 | Years of schooling | G | T | 0.7721 | 0.01654 | 0.00207 | 1.25E-15 |
| rs301800 | Years of schooling | C | T | 0.8197 | -0.01516 | 0.00224 | 1.33E-11 |
| rs30266 | Years of schooling | A | G | 0.3418 | -0.01128 | 0.00181 | 4.84E-10 |
| rs317656 | Years of schooling | A | T | 0.7143 | -0.00153 | 0.0019 | 0.422 |
| rs320693 | Years of schooling | C | G | 0.4728 | 0.01204 | 0.0017 | 1.58E-12 |
| rs3211995 | Years of schooling | A | G | 0.1497 | 0.00901 | 0.00238 | 0.000151 |
| rs329118 | Years of schooling | T | C | 0.415 | 0.0081 | 0.00172 | 2.53E-06 |
| rs34025316 | Years of schooling | T | C | 0.3265 | 0.00088 | 0.00182 | 0.626 |
| rs34045288 | Years of schooling | T | C | 0.3146 | -0.0064 | 0.0018 | 0.00038 |
| rs34234296 | Years of schooling | A | G | 0.3554 | 0.00083 | 0.00177 | 0.641 |
| rs34481751 | Years of schooling | A | C | 0.1395 | 0.00376 | 0.00229 | 0.102 |
| rs34517439 | Years of schooling | A | C | 0.1054 | -0.01112 | 0.00266 | 2.91E-05 |
| rs347551 | Years of schooling | G | C | 0.4643 | 0.00102 | 0.00174 | 0.557 |
| rs34811474 | Years of schooling | A | G | 0.2296 | 0.00868 | 0.00214 | 4.89E-05 |
| rs35154326 | Years of schooling | G | A | 0.2755 | 0.00537 | 0.0019 | 0.00464 |
| rs35309068 | Years of schooling | G | T | 0.466 | 0.01321 | 0.00171 | 1.15E-14 |
| rs354155 | Years of schooling | C | G | 0.08844 | -0.00709 | 0.00289 | 0.0141 |
| rs35417702 | Years of schooling | T | C | 0.5765 | -0.01445 | 0.0017 | 1.93E-17 |
| rs35475880 | Years of schooling | T | G | 0.1837 | -0.01511 | 0.00208 | 3.80E-13 |
| rs35532491 | Years of schooling | T | A | 0.1071 | 0.02007 | 0.00286 | 2.42E-12 |
| rs355777 | Years of schooling | C | G | 0.4252 | -0.00382 | 0.00173 | 0.027 |
| rs35867081 | Years of schooling | G | A | 0.4881 | -0.0048 | 0.00172 | 0.00538 |
| rs36007635 | Years of schooling | A | G | 0.1616 | 0.00753 | 0.00249 | 0.00249 |
| rs36061954 | Years of schooling | T | C | 0.381 | -0.00351 | 0.00174 | 0.0434 |
| rs36119825 | Years of schooling | A | G | 0.4694 | 0.01063 | 0.00171 | 4.82E-10 |
| rs363096 | Years of schooling | C | T | 0.5748 | 0.01363 | 0.00172 | 2.04E-15 |
| rs3747631 | Years of schooling | C | G | 0.2279 | 0.02207 | 0.00208 | 2.97E-26 |
| rs3764625 | Years of schooling | G | T | 0.6088 | 0.0018 | 0.00172 | 0.296 |
| rs3770754 | Years of schooling | G | C | 0.3776 | -0.00208 | 0.00177 | 0.241 |
| rs3784710 | Years of schooling | C | T | 0.2466 | 0.00048 | 0.00205 | 0.817 |
| rs3800546 | Years of schooling | G | C | 0.2704 | -0.01183 | 0.00194 | 9.73E-10 |
| rs3807865 | Years of schooling | A | G | 0.415 | -0.00341 | 0.00173 | 0.0488 |
| rs3809634 | Years of schooling | G | A | 0.335 | 0.01058 | 0.00185 | 1.09E-08 |
| rs3814883 | Years of schooling | T | C | 0.449 | -0.00045 | 0.00171 | 0.792 |
| rs3845344 | Years of schooling | T | C | 0.3827 | -0.00065 | 0.00176 | 0.711001 |
| rs3851998 | Years of schooling | G | C | 0.7568 | 0.00198 | 0.00195 | 0.311 |
| rs3896224 | Years of schooling | G | A | 0.4269 | 0.0088 | 0.00173 | 3.40E-07 |
| rs3897821 | Years of schooling | G | A | 0.3503 | -0.01502 | 0.0018 | 8.25E-17 |
| rs3901286 | Years of schooling | A | C | 0.1463 | 0.00421 | 0.00235 | 0.0732 |
| rs394608 | Years of schooling | C | T | 0.5034 | -0.00439 | 0.0017 | 0.01 |
| rs40071 | Years of schooling | C | T | 0.1837 | 0.00026 | 0.00222 | 0.905 |
| rs4044321 | Years of schooling | G | A | 0.6769 | -0.0012 | 0.00177 | 0.497 |
| rs4055791 | Years of schooling | T | C | 0.3861 | 0.00213 | 0.00172 | 0.217 |
| rs406388 | Years of schooling | G | C | 0.1769 | -0.00426 | 0.0022 | 0.053099 |
| rs4148155 | Years of schooling | G | A | 0.1037 | 0.00038 | 0.00271 | 0.887 |
| rs4261944 | Years of schooling | G | T | 0.3435 | 0.00072 | 0.00179 | 0.685001 |
| rs4267103 | Years of schooling | C | T | 0.1905 | -0.00031 | 0.0022 | 0.888 |
| rs429343 | Years of schooling | G | A | 0.5714 | 0.00063 | 0.00172 | 0.715 |
| rs429358 | Years of schooling | C | T | 0.165 | 0.00707 | 0.00238 | 0.003 |
| rs4328757 | Years of schooling | T | C | 0.6514 | 0.01067 | 0.00174 | 9.39E-10 |
| rs4352658 | Years of schooling | T | C | 0.09014 | -0.0212 | 0.00308 | 5.55E-12 |
| rs4382592 | Years of schooling | G | T | 0.699 | 0.01636 | 0.00185 | 1.01E-18 |
| rs4419475 | Years of schooling | T | A | 0.3776 | -0.00551 | 0.00174 | 0.00157 |
| rs4439537 | Years of schooling | C | T | 0.5391 | 0.00522 | 0.0017 | 0.00212 |
| rs4477562 | Years of schooling | T | C | 0.1003 | -0.00013 | 0.00255 | 0.96 |
| rs4482463 | Years of schooling | A | C | 0.90646 | -0.00428 | 0.00323 | 0.185 |
| rs4613074 | Years of schooling | C | T | 0.199 | -0.00316 | 0.00223 | 0.156 |
| rs4648450 | Years of schooling | A | C | 0.4949 | 0.00368 | 0.00171 | 0.0312 |
| rs4653164 | Years of schooling | T | C | 0.6803 | 0.00024 | 0.00183 | 0.896 |
| rs4671328 | Years of schooling | G | T | 0.5663 | 0.00336 | 0.00175 | 0.0545 |
| rs4700393 | Years of schooling | G | A | 0.5289 | 0.02086 | 0.0017 | 1.51E-34 |
| rs4702 | Years of schooling | A | G | 0.5935 | -9.00E-05 | 0.00173 | 0.961 |
| rs4709807 | Years of schooling | C | T | 0.7483 | 0.00191 | 0.00202 | 0.345 |
| rs4737188 | Years of schooling | T | A | 0.4745 | 0.00628 | 0.0017 | 0.00023 |
| rs4757144 | Years of schooling | A | G | 0.6037 | 0.00143 | 0.00172 | 0.409 |
| rs4757957 | Years of schooling | C | G | 0.6514 | 0.0141 | 0.00184 | 1.81E-14 |
| rs4764949 | Years of schooling | G | A | 0.3282 | -0.00104 | 0.00181 | 0.568 |
| rs4790292 | Years of schooling | A | C | 0.1344 | 0.00775 | 0.00239 | 0.00119 |
| rs4810227 | Years of schooling | A | G | 0.6344 | 0.01272 | 0.00175 | 3.57E-13 |
| rs4812325 | Years of schooling | A | G | 0.5578 | 0.00523 | 0.00174 | 0.00267 |
| rs4832298 | Years of schooling | T | C | 0.6548 | 0.00426 | 0.00188 | 0.0236 |
| rs4846724 | Years of schooling | A | G | 0.4915 | 0.01018 | 0.0017 | 2.26E-09 |
| rs4876611 | Years of schooling | G | A | 0.7738 | -0.00319 | 0.00189 | 0.0923 |
| rs4895799 | Years of schooling | T | C | 0.5833 | 0.00436 | 0.00172 | 0.0113 |
| rs4958702 | Years of schooling | C | T | 0.5731 | -0.00065 | 0.00172 | 0.704001 |
| rs4961705 | Years of schooling | C | G | 0.3588 | 0.00367 | 0.00179 | 0.0402 |
| rs512121 | Years of schooling | C | T | 0.2024 | 0.00114 | 0.00217 | 0.6 |
| rs539515 | Years of schooling | C | A | 0.199 | -0.00467 | 0.00213 | 0.0281 |
| rs55658481 | Years of schooling | A | G | 0.3316 | -0.00698 | 0.00179 | 9.81E-05 |
| rs55707359 | Years of schooling | G | T | 0.0102 | -0.01525 | 0.0074 | 0.0394 |
| rs55714539 | Years of schooling | C | A | 0.3265 | -0.00603 | 0.0018 | 0.000786 |
| rs55726687 | Years of schooling | A | G | 0.2007 | 0.00754 | 0.00211 | 0.000346 |
| rs55736314 | Years of schooling | G | C | 0.4167 | 0.01431 | 0.00174 | 1.63E-16 |
| rs558887 | Years of schooling | G | A | 0.3282 | 0.00356 | 0.00185 | 0.0541 |
| rs56094641 | Years of schooling | G | A | 0.4201 | 0.00286 | 0.00173 | 0.0982 |
| rs56133507 | Years of schooling | G | T | 0.1956 | -0.0125 | 0.00215 | 6.31E-09 |
| rs56161855 | Years of schooling | T | A | 0.1548 | -0.00431 | 0.00248 | 0.082499 |
| rs56203622 | Years of schooling | C | T | 0.1718 | -0.00348 | 0.00241 | 0.149 |
| rs56335113 | Years of schooling | G | A | 0.6769 | -0.00292 | 0.00185 | 0.114 |
| rs56356382 | Years of schooling | C | T | 0.199 | 0.00347 | 0.00223 | 0.119 |
| rs56391344 | Years of schooling | A | G | 0.2381 | 0.01571 | 0.00197 | 1.34E-15 |
| rs56399737 | Years of schooling | T | C | 0.4847 | 0.00288 | 0.00171 | 0.0925 |
| rs56858768 | Years of schooling | A | G | 0.3027 | -0.006 | 0.00185 | 0.00118 |
| rs57636386 | Years of schooling | C | T | 0.07993 | 0.00491 | 0.00307 | 0.11 |
| rs58120505 | Years of schooling | C | T | 0.4439 | -0.0056 | 0.00172 | 0.00112 |
| rs59086897 | Years of schooling | A | T | 0.4949 | -0.00098 | 0.0017 | 0.565999 |
| rs59237168 | Years of schooling | C | T | 0.1973 | 0.0029 | 0.00205 | 0.158 |
| rs5995843 | Years of schooling | G | A | 0.3316 | -0.00434 | 0.00178 | 0.0147 |
| rs60764613 | Years of schooling | T | G | 0.131 | -0.00254 | 0.00239 | 0.288 |
| rs6123924 | Years of schooling | G | A | 0.1599 | -0.01528 | 0.00235 | 7.55E-11 |
| rs6134916 | Years of schooling | T | C | 0.4898 | -0.00015 | 0.0017 | 0.928 |
| rs61813324 | Years of schooling | T | C | 0.1259 | -0.00985 | 0.00256 | 0.000122 |
| rs61828088 | Years of schooling | A | G | 0.09524 | 0.0022 | 0.00273 | 0.421 |
| rs61903695 | Years of schooling | G | A | 0.2959 | -0.0082 | 0.00196 | 2.98E-05 |
| rs61914045 | Years of schooling | A | G | 0.2228 | -0.0013 | 0.00211 | 0.54 |
| rs62097985 | Years of schooling | T | C | 0.4116 | -0.01288 | 0.00172 | 6.06E-14 |
| rs62107261 | Years of schooling | C | T | 0.04932 | -0.00316 | 0.00427 | 0.46 |
| rs62134195 | Years of schooling | T | C | 0.04932 | 0.00571 | 0.00432 | 0.186 |
| rs62176243 | Years of schooling | T | A | 0.2466 | 0.01044 | 0.00195 | 9.26E-08 |
| rs62176993 | Years of schooling | A | G | 0.3793 | -0.00279 | 0.00174 | 0.108 |
| rs62190049 | Years of schooling | C | G | 0.3963 | 0.00342 | 0.00176 | 0.0514 |
| rs62439690 | Years of schooling | A | G | 0.267 | -0.01087 | 0.00194 | 2.18E-08 |
| rs6265 | Years of schooling | T | C | 0.1939 | 0.00974 | 0.00218 | 8.05E-06 |
| rs6531639 | Years of schooling | A | G | 0.2279 | 0.00607 | 0.002 | 0.00244 |
| rs6545714 | Years of schooling | A | G | 0.6241 | 0.00445 | 0.00174 | 0.0104 |
| rs6546857 | Years of schooling | G | A | 0.2058 | -0.00829 | 0.00199 | 3.05E-05 |
| rs6560906 | Years of schooling | C | T | 0.7364 | 0.00314 | 0.00183 | 0.086399 |
| rs6567160 | Years of schooling | C | T | 0.2517 | 0.00535 | 0.00201 | 0.00782 |
| rs6575340 | Years of schooling | A | G | 0.631 | -0.00744 | 0.00177 | 2.65E-05 |
| rs66511648 | Years of schooling | C | T | 0.2568 | 0.00203 | 0.00189 | 0.284 |
| rs66679256 | Years of schooling | T | C | 0.4677 | -0.0028 | 0.00171 | 0.102 |
| rs6669341 | Years of schooling | G | A | 0.5765 | 0.00014 | 0.00173 | 0.936 |
| rs6682438 | Years of schooling | C | T | 0.6327 | -0.00268 | 0.00181 | 0.138 |
| rs6688826 | Years of schooling | C | T | 0.3231 | -0.00574 | 0.00187 | 0.00217 |
| rs6707827 | Years of schooling | G | A | 0.7092 | -0.00878 | 0.00186 | 2.32E-06 |
| rs6710091 | Years of schooling | G | C | 0.3299 | 0.00377 | 0.00178 | 0.0339 |
| rs6719762 | Years of schooling | C | T | 0.4286 | -0.00935 | 0.0017 | 3.83E-08 |
| rs6731967 | Years of schooling | C | G | 0.2092 | -0.01186 | 0.00199 | 2.36E-09 |
| rs6744646 | Years of schooling | G | A | 0.8401 | -0.00487 | 0.00226 | 0.0313 |
| rs6744794 | Years of schooling | G | C | 0.6071 | -0.01423 | 0.00175 | 3.71E-16 |
| rs6752979 | Years of schooling | A | G | 0.3027 | -0.00726 | 0.00183 | 7.04E-05 |
| rs6774894 | Years of schooling | A | T | 0.3844 | -0.00733 | 0.00177 | 3.48E-05 |
| rs6798742 | Years of schooling | G | A | 0.3299 | -0.00019 | 0.00186 | 0.919 |
| rs6803651 | Years of schooling | T | G | 0.415 | 0.01131 | 0.00172 | 4.36E-11 |
| rs6805241 | Years of schooling | C | T | 0.1973 | -0.01413 | 0.00203 | 3.09E-12 |
| rs6843852 | Years of schooling | T | C | 0.5068 | -0.00275 | 0.0017 | 0.105 |
| rs6943762 | Years of schooling | C | T | 0.131 | -0.00297 | 0.00253 | 0.241 |
| rs6959891 | Years of schooling | G | A | 0.2959 | -0.01136 | 0.00189 | 1.74E-09 |
| rs6962980 | Years of schooling | C | A | 0.5306 | 0.00199 | 0.00177 | 0.259 |
| rs6974218 | Years of schooling | C | A | 0.4099 | -0.00589 | 0.00174 | 0.000728 |
| rs698147 | Years of schooling | G | A | 0.5238 | 0.00445 | 0.00171 | 0.00907 |
| rs7008955 | Years of schooling | G | T | 0.5289 | 0.0026 | 0.0017 | 0.127 |
| rs7012546 | Years of schooling | T | C | 0.4201 | 0.01009 | 0.00172 | 4.93E-09 |
| rs7029718 | Years of schooling | A | G | 0.4354 | 0.02439 | 0.00174 | 1.85E-44 |
| rs7031698 | Years of schooling | C | T | 0.7755 | 0.01248 | 0.00206 | 1.26E-09 |
| rs7034554 | Years of schooling | G | A | 0.3759 | -0.00123 | 0.00178 | 0.489 |
| rs7038943 | Years of schooling | C | T | 0.3554 | 0.0075 | 0.00179 | 2.65E-05 |
| rs7079070 | Years of schooling | A | G | 0.4507 | -0.0046 | 0.00171 | 0.0073 |
| rs708228 | Years of schooling | T | C | 0.3197 | -0.00967 | 0.00181 | 8.65E-08 |
| rs7124681 | Years of schooling | A | C | 0.4371 | 0.00019 | 0.00173 | 0.911 |
| rs7132908 | Years of schooling | A | G | 0.3588 | -0.00048 | 0.00176 | 0.786001 |
| rs71646142 | Years of schooling | T | C | 0.1735 | 0.01286 | 0.00217 | 3.11E-09 |
| rs7201895 | Years of schooling | A | G | 0.3707 | 0.00257 | 0.00178 | 0.149 |
| rs7206608 | Years of schooling | G | C | 0.318 | -0.00668 | 0.00182 | 0.000239 |
| rs7218014 | Years of schooling | C | T | 0.2143 | -0.0033 | 0.00215 | 0.125 |
| rs7233920 | Years of schooling | A | G | 0.216 | -0.01315 | 0.00202 | 7.13E-11 |
| rs7236339 | Years of schooling | A | G | 0.1956 | -0.01232 | 0.00204 | 1.43E-09 |
| rs723672 | Years of schooling | T | C | 0.4422 | 0.00184 | 0.00172 | 0.283 |
| rs7250833 | Years of schooling | T | C | 0.3061 | -0.00191 | 0.00188 | 0.308 |
| rs7259070 | Years of schooling | C | T | 0.5833 | 0.00154 | 0.00182 | 0.397 |
| rs72673947 | Years of schooling | G | A | 0.1088 | -0.00276 | 0.00273 | 0.311 |
| rs7278859 | Years of schooling | T | A | 0.3078 | 0.01013 | 0.00185 | 4.15E-08 |
| rs72887338 | Years of schooling | C | T | 0.4014 | -0.00247 | 0.00175 | 0.158 |
| rs72892910 | Years of schooling | T | G | 0.1599 | -0.00386 | 0.00222 | 0.0828 |
| rs72910629 | Years of schooling | G | A | 0.1224 | -0.00549 | 0.00248 | 0.0267 |
| rs72986630 | Years of schooling | T | C | 0.05782 | 0.01283 | 0.00365 | 0.000446 |
| rs73026725 | Years of schooling | A | C | 0.1582 | -0.00446 | 0.00237 | 0.0604 |
| rs730384 | Years of schooling | A | G | 0.4558 | 0.01016 | 0.00171 | 3.01E-09 |
| rs73052033 | Years of schooling | C | T | 0.2041 | -0.0076 | 0.00222 | 0.000612 |
| rs73142879 | Years of schooling | T | C | 0.1956 | 0.00698 | 0.00217 | 0.00128 |
| rs73213484 | Years of schooling | T | A | 0.1803 | -0.00109 | 0.00242 | 0.653 |
| rs7331420 | Years of schooling | A | G | 0.3044 | 0.00896 | 0.00187 | 1.68E-06 |
| rs7332724 | Years of schooling | T | C | 0.2619 | -0.01149 | 0.00189 | 1.26E-09 |
| rs73344830 | Years of schooling | G | A | 0.602 | -0.0172 | 0.00172 | 1.95E-23 |
| rs7357754 | Years of schooling | G | A | 0.5238 | -0.00779 | 0.0017 | 4.74E-06 |
| rs7359501 | Years of schooling | T | C | 0.369 | 0.00701 | 0.00173 | 5.16E-05 |
| rs736282 | Years of schooling | C | T | 0.5153 | -0.01082 | 0.0017 | 2.07E-10 |
| rs7442137 | Years of schooling | T | C | 0.6463 | 0.00351 | 0.00177 | 0.0475 |
| rs7442885 | Years of schooling | G | C | 0.216 | 0.01402 | 0.00208 | 1.58E-11 |
| rs745249 | Years of schooling | T | C | 0.2381 | 0.00111 | 0.00188 | 0.555 |
| rs7498044 | Years of schooling | A | G | 0.2075 | 0.00398 | 0.00206 | 0.0533 |
| rs7498665 | Years of schooling | G | A | 0.3367 | -0.01662 | 0.00174 | 1.09E-21 |
| rs75035127 | Years of schooling | G | A | 0.03571 | 0.01863 | 0.00513 | 0.000281 |
| rs7551758 | Years of schooling | G | T | 0.5544 | -0.00395 | 0.0017 | 0.0203 |
| rs7575189 | Years of schooling | A | G | 0.5476 | 0.00828 | 0.00173 | 1.62E-06 |
| rs7594904 | Years of schooling | C | T | 0.4184 | 0.00969 | 0.00173 | 2.05E-08 |
| rs76076331 | Years of schooling | T | C | 0.131 | 0.01873 | 0.00248 | 4.40E-14 |
| rs76702514 | Years of schooling | G | C | 0.2449 | 0.0018 | 0.00208 | 0.386 |
| rs76878669 | Years of schooling | G | C | 0.2534 | -0.01399 | 0.00205 | 8.67E-12 |
| rs76954012 | Years of schooling | A | T | 0.119 | -0.00379 | 0.00296 | 0.201 |
| rs7704530 | Years of schooling | A | G | 0.6973 | -0.00834 | 0.0019 | 1.10E-05 |
| rs7762794 | Years of schooling | G | A | 0.2687 | -0.00744 | 0.00187 | 6.82E-05 |
| rs77702622 | Years of schooling | A | G | 0.07653 | -0.02447 | 0.00351 | 2.99E-12 |
| rs7774 | Years of schooling | A | C | 0.3231 | -0.0029 | 0.00185 | 0.116 |
| rs77835879 | Years of schooling | G | A | 0.09014 | -0.01601 | 0.00288 | 2.68E-08 |
| rs778371 | Years of schooling | G | A | 0.318 | 0.00998 | 0.00189 | 1.22E-07 |
| rs7785195 | Years of schooling | A | G | 0.6463 | 0.00807 | 0.00179 | 6.47E-06 |
| rs7803932 | Years of schooling | A | G | 0.1565 | 0.0143 | 0.00226 | 2.44E-10 |
| rs78086698 | Years of schooling | C | T | 0.04082 | -0.00693 | 0.00456 | 0.128 |
| rs7852189 | Years of schooling | G | A | 0.3299 | -0.00476 | 0.00182 | 0.00899 |
| rs7893571 | Years of schooling | T | G | 0.6769 | 0.0024 | 0.00181 | 0.185 |
| rs7909331 | Years of schooling | G | A | 0.1599 | -0.00519 | 0.00229 | 0.0234 |
| rs7920624 | Years of schooling | T | A | 0.5068 | 0.01181 | 0.0017 | 3.97E-12 |
| rs7921378 | Years of schooling | C | G | 0.4898 | 0.00212 | 0.0017 | 0.214 |
| rs7924036 | Years of schooling | T | G | 0.5391 | 0.01501 | 0.0017 | 1.07E-18 |
| rs79265434 | Years of schooling | G | A | 0.1173 | 0.02331 | 0.00262 | 6.08E-19 |
| rs79269403 | Years of schooling | A | G | 0.2228 | 0.01447 | 0.00204 | 1.17E-12 |
| rs7927195 | Years of schooling | G | A | 0.6003 | 0.00029 | 0.00174 | 0.869 |
| rs7928622 | Years of schooling | T | A | 0.3078 | 0.01011 | 0.00181 | 2.52E-08 |
| rs7938812 | Years of schooling | G | T | 0.3997 | -0.00739 | 0.00173 | 2.02E-05 |
| rs79445414 | Years of schooling | C | T | 0.05612 | -0.00269 | 0.00401 | 0.503 |
| rs7944782 | Years of schooling | G | T | 0.5085 | -0.00493 | 0.0017 | 0.00369 |
| rs7952102 | Years of schooling | C | T | 0.3861 | 0.00518 | 0.00176 | 0.00317 |
| rs7996639 | Years of schooling | A | G | 0.4694 | -0.00293 | 0.00171 | 0.086099 |
| rs80153284 | Years of schooling | A | C | 0.01361 | 0.01741 | 0.00728 | 0.0168 |
| rs8015400 | Years of schooling | A | C | 0.6701 | -0.00215 | 0.00182 | 0.237 |
| rs8020034 | Years of schooling | A | G | 0.2058 | 0.01782 | 0.00223 | 1.17E-15 |
| rs8112818 | Years of schooling | G | A | 0.4201 | 0.00063 | 0.00178 | 0.724001 |
| rs8132491 | Years of schooling | A | G | 0.2755 | -0.00013 | 0.00188 | 0.945 |
| rs815163 | Years of schooling | C | T | 0.5986 | -9.00E-05 | 0.00171 | 0.957 |
| rs862320 | Years of schooling | T | C | 0.4252 | 0.001 | 0.00172 | 0.562 |
| rs879620 | Years of schooling | T | C | 0.6054 | -0.00081 | 0.00176 | 0.645 |
| rs892612 | Years of schooling | C | A | 0.8418 | 0.01464 | 0.00237 | 6.63E-10 |
| rs9294260 | Years of schooling | A | G | 0.449 | -0.00544 | 0.0017 | 0.0014 |
| rs9296389 | Years of schooling | C | G | 0.415 | -0.00124 | 0.00172 | 0.472 |
| rs9320493 | Years of schooling | G | A | 0.8639 | -0.01394 | 0.0024 | 6.13E-09 |
| rs9349956 | Years of schooling | C | A | 0.2398 | 0.01881 | 0.00225 | 6.28E-17 |
| rs935166 | Years of schooling | A | G | 0.4847 | 0.00777 | 0.0017 | 4.74E-06 |
| rs9366863 | Years of schooling | C | T | 0.6429 | 0.0031 | 0.00181 | 0.0875 |
| rs9372625 | Years of schooling | A | G | 0.4133 | 0.02383 | 0.00176 | 6.76E-42 |
| rs9386319 | Years of schooling | G | A | 0.4269 | 0.00991 | 0.00174 | 1.27E-08 |
| rs9478496 | Years of schooling | C | T | 0.1582 | -0.00131 | 0.00227 | 0.562999 |
| rs9503598 | Years of schooling | A | G | 0.4388 | 0.01079 | 0.00171 | 3.12E-10 |
| rs9514600 | Years of schooling | G | C | 0.517 | 0.0027 | 0.0017 | 0.113 |
| rs9522173 | Years of schooling | T | A | 0.3844 | 0.00374 | 0.00174 | 0.0315 |
| rs9529119 | Years of schooling | G | C | 0.8027 | -0.01295 | 0.00204 | 2.13E-10 |
| rs9571687 | Years of schooling | A | C | 0.3384 | -0.00161 | 0.00182 | 0.378 |
| rs9616906 | Years of schooling | A | G | 0.4235 | 0.01497 | 0.00172 | 2.92E-18 |
| rs9636107 | Years of schooling | G | A | 0.4762 | -0.00372 | 0.0017 | 0.0287 |
| rs9643087 | Years of schooling | T | C | 0.5612 | -0.00254 | 0.0017 | 0.136 |
| rs969512 | Years of schooling | T | A | 0.2959 | 0.01249 | 0.00179 | 3.22E-12 |
| rs9704097 | Years of schooling | A | C | 0.4728 | -0.0103 | 0.00171 | 1.61E-09 |
| rs9852062 | Years of schooling | A | T | 0.568 | 0.00455 | 0.00171 | 0.00782 |
| rs9860326 | Years of schooling | G | C | 0.3197 | -0.00302 | 0.00181 | 0.0948 |
| rs9882532 | Years of schooling | C | T | 0.3639 | -0.01208 | 0.00177 | 8.17E-12 |
| rs9888533 | Years of schooling | T | C | 0.5119 | -0.00027 | 0.00175 | 0.879 |
| rs9951619 | Years of schooling | G | T | 0.7755 | 0.00519 | 0.00202 | 0.0101 |
| rs9964724 | Years of schooling | T | C | 0.6599 | 0.01978 | 0.00183 | 2.66E-27 |

Abbreviations: MVMR = Multivariable Mendelian randomization. SNP = single nucleotide polymorphism. MD = Major depression. BMI = Body mass index. SCZ = Schizophrenia. SI = Smoking initiation.
